# Direct (LC-)MS Identification of Regioisomers from C–H Functionalization by Partial Isotopic Labeling

**DOI:** 10.1021/acscentsci.4c01765

**Published:** 2025-02-14

**Authors:** Christopher A. Sojdak, David A. Polefrone, Hriday M. Shah, Cassandra D. Vu, Brandon J. Orzolek, Pedro M. Jimenez Antenucci, Micah Valadez Bush, Marisa C. Kozlowski

**Affiliations:** † Department of Chemistry, Roy and Diana Vagelos Laboratories, University of Pennsylvania, Philadelphia, Pennsylvania 19104-6323, United States

## Abstract

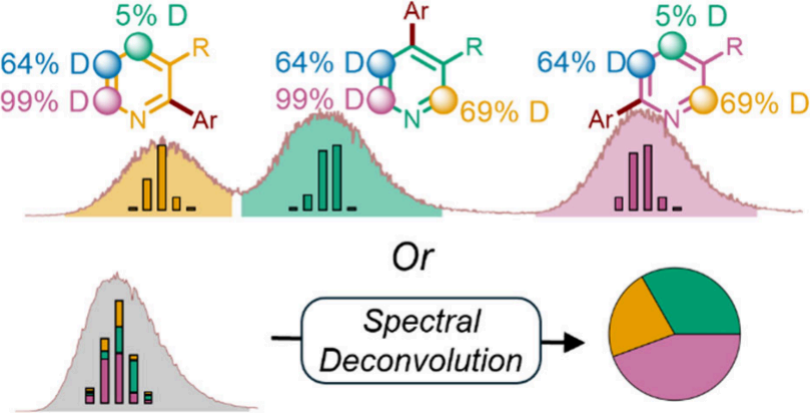

C–H functionalization of complex substrates is highly enabling in total synthesis and in the development of late-stage drug candidates. Much work has been dedicated to developing new methods as well as predictive modeling to accelerate route scouting. However, workflows to identify regioisomeric products are arduous, typically requiring chromatographic separation and/or nuclear magnetic resonance spectroscopy analysis. In addition, most reports focus on major products or do not assign regioisomeric products, which biases predictive models constructed from such data. Herein, we present a novel approach to complex reaction analysis utilizing partial deuterium labels, which enables direct product identification via liquid chromatography–mass spectrometry. When combined with spectral deconvolution, the method generates product ratios while circumventing chromatography altogether. Competitive kinetic isotope effects can also be determined. The resultant data are expected to be useful in the construction of predictive models across several dimensions including reaction selectivity, the impact of structure on mechanism, and mass spectral ionization patterns and expedite the identification of drug metabolites.

## Abstract 2

Partial isotopic labels allow direct identification of regioisomers via their distinct isotopic distributions. Alternately, spectral deconvolution of unseparated mixtures delivers regioisomer ratios.

## Introduction

The use of late-stage functionalization (LSF) to alter the molecular properties of drug candidates late in development has become an essential part of modern medicinal chemistry and drug discovery. Optimizing the potency and pharmacokinetic characteristics of preexisting or drugs late in development can represent a more cost-effective approach compared to initiating the development process anew with a novel compound. Of particular interest is C–H functionalization and to this end, a large body of work now exists allowing for the functionalization of complex and structurally diverse compounds.^1−3^

**Figure 1:**
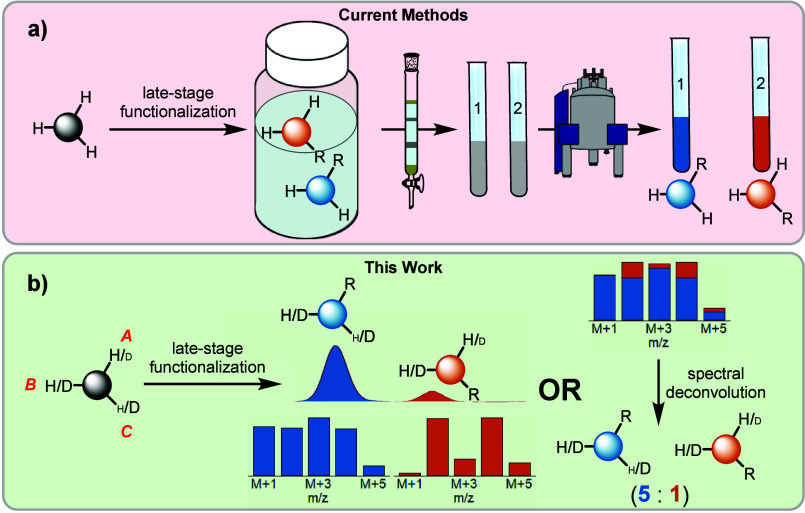
Methods for product identification. **a)** Traditional workflow to identify mixtures of regioisomers. **b)** Our work using isotopic labels to identify separated and unseparated mixtures of regioisomers.

Although significant progress has been made in developing LSF, predicting outcomes when regioisomeric products are possible is often difficult. To this end, efforts have been devoted to developing predictive models. For example, in 2023 the Hartwig group developed a model which could predict the major reactive site of an iridium-catalyzed C–H borylation of six arenes.^4^ The underlying data for such modeling relies on identifying the major product isomers in a large number of transformations. While high-throughput experimentation (HTE) is well-suited to rapidly conducting a large number of reactions to collect such data, the identification of products when isomers can form is challenging. Typically, such data is obtained by isolation of the relevant products and subsequent nuclear magnetic resonance (NMR) spectroscopic analysis. When multiple products are obtained, this workflow rapidly becomes more difficult as isomers are difficult to separate, and NMR analysis of multiple products is required (Figure 1a). Once identities are secured, quantitation can be undertaken via liquid chromatography (LC) or gas chromatography (GC) coupled with UV–vis or mass spectral (MS) detection typically requiring calibrated standards.^5^ While evaporative light scattering detection (ELSD) and charged aerosol detection (CAD) can provide improved quantitation after LC without standards, and MS techniques such as matrix-assisted laser desorption ionization (MALDI) or desorption electrospray ionization (DESI) enable highly sensitive chromatography-free detection of analytes, none of these approaches directly overcome the challenge of isomer identification.^6^ The current methods for quantitation of isomeric ratios are slow, cost prohibitive such as in-line LC-NMR,^7^ molecular rotational resonance spectroscopy (MRR),^8^ ion mobility spectrometry–mass spectrometry (IMS–MS),^9^ or niche in their application, such as DNA or peptide analysis via mass fragmentation.^10^

With the goal of rapidly generating data sets for machine learning to generate predictive models of C–H functionalization where regioisomeric outcomes are possible, we propose utilizing deuterium (^2^H or D) labels to identify regioisomers. Distinct partial isotopic labeling of reactive sites on a substrate allows direct identification of different regiosiomeric products. Product ratios can even be successfully measured without the need for LC separation by using spectral deconvolution (Figure 1b).

## Results and Discussion

### Method Development

To identify regioisomers via their unique isotopic distributions, potential reactive sites were labeled with differing amounts of deuterium. For example, a conventional C–H functionalization having three reactive sites A, B, and C with different amounts of deuterium at each position (A = 25% ^2^H; B = 50% ^2^H; C = 75% ^2^H) would lead to each unique product exhibiting a distinct isotopic fingerprint. In this case, a reaction at position A forms a noticeably heavier product compared to that formed at position C. Consequently, direct regioisomer identification can be accomplished by uncalibrated LC-MS analysis. The incorporation of deuterium into drugs has been used as a strategy to improve pharmacokinetic properties or reduce toxicity relative to their protio counterparts.^11^ In addition, the greater use of analytical mass spectrometry has created a demand for internal deuterated standards^12^ and tritiated compounds are used in many aspects of drug discovery and development.^13^ Altogether, these needs have driven substantial development in undirected deuterium labeling of sp, sp^2^, and sp^3^ centered C–H bonds, often utilizing D_2_O as an inexpensive deuterium source.^14−18^ Leveraging these well-established methods allows for the rapid generation of the deuterated analogs needed for this approach with a broad range of molecules.

**Figure 2:**
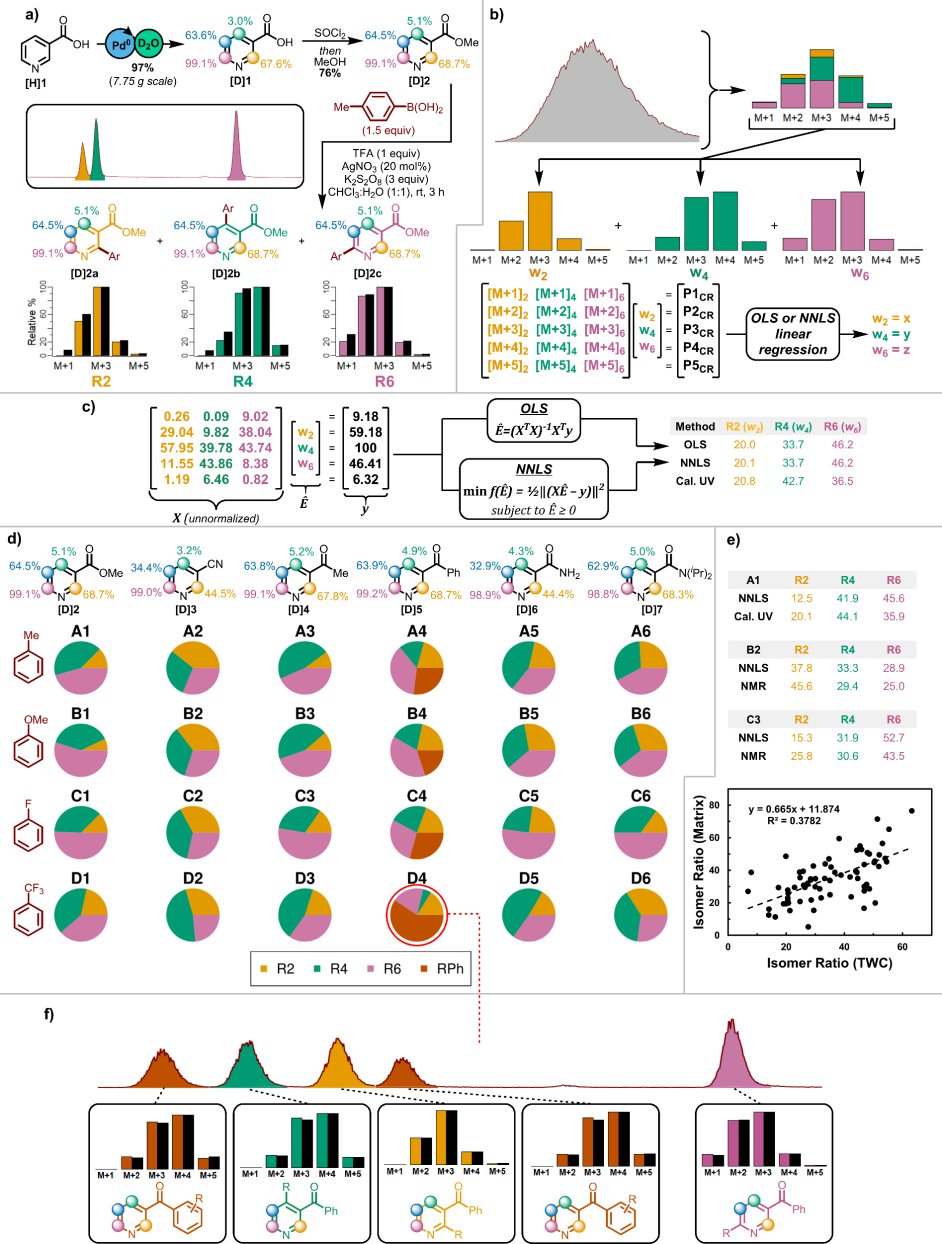
Method development and parallel microscale reactions. **a)** Proof-of-concept Minisci reaction. **b)** Depiction of matrix deconvolution. **c)** Matrix deconvolution of proof-of-concept reaction via OLS and NNLS regression. **d)** 24-well plate depicting Minisci coupling reactions between **[D]2**–**[D]7** and four aryl radicals. Product ratios are determined via NNLS deconvolution. **e)** (top) Select examples comparing the matrix deconvoluted product ratios and product ratios from protio trials for wells: A1; B2; C3. (bottom) Comparison of matrix deconvolution results with total wavelength chromatogram (TWC) ratios. **f)** LC trace showcasing the isotopic patterns of the five products formed in well D4.

Minisci reactions of *N*-heterocycles are highly enabling in medicinal chemistry discovery.^19^ However, multiple isomers can form as in the case of 3-substituted pyridines.^20^ Using a modified literature procedure, [D]-methyl nicotinate (**[D]2**) with different levels of deuterium labels at the C2, C4, C5, and C6 positions was generated by a straightforward palladium catalyzed exchange with D_2_O^21^ followed by esterification (Figure 2a). Labeled **[D]2** was then subjected to conventional Minisci coupling conditions using *para*-tolylboronic acid as a radical source.^22^ The unpurified reaction mixture was analyzed by LC-MS analysis and the experimental isotopic distributions (M+1 to M+5) of the LC product peaks (colored bars = experimental) were compared with the predicted values from the deuterium labels measured in the starting material (black bars) allowing assignment of the individual peaks *without isolation* (Figure 2a). No C5 product was observed in line with prior literature.^22,23^ The identity of these products was later confirmed by isolating the individual peaks, securing the identity of each by ^1^H NMR spectroscopy, and comparing LC retention times with the isolated standards (see Supporting Information, SI).

Determining product ratios from unseparated mixtures can be done by first predicting the unique isotopic distribution (ranging from M+1 to M+5 in the case of Figure 2a) of each product formed using the deuterium incorporation values determined via ^1^H NMR of the starting material. Direct injection of the sample to the mass detector provides five unique *m*/*z* values (M+1 to M+5) which can be expressed as a vector, P_CR_, and correlated to the weights of the three isomers (w_2_, w_4_, w_6_) expressed as vector w_*x*_ (Figure 2b, see SI for details). After constructing these matrices, linear regression (OLS or NNLS) can be done in order to solve for the relative weights of each regioisomers present in the unseparated mixture (Figure 2b).^24^Figure 2c illustrates that this matrix deconvolution gives rise to similar product ratios compared to that from conventional LC UV–vis analysis using calibrated standards (Figure 2c). In this instance, similar product ratios are obtained whether using OLS or NNLS regression, but the NNLS should be utilized if OLS delivers negative values, as negative percent contributions are nonsensical.

### Parallel Microscale Reactions

With these promising results (Figure 2a–c), six 3-substituted pyridines (**[D]2**–**7**) were prepared from a common labeled intermediate **[D]1** which simplifies the introduction of the appropriate levels of labeling. A 24-well plate was designed for Minisci coupling on a 1 μmol scale of these six 3-substituted pyridines (**[D]2**–**7**) with four electronically diverse aryl radicals (Figure 2d). Each sample was analyzed by MS without separation in triplicate and product ratios were deconvoluted using isotopic distributions from the starting substrate (Figure 2d). Deconvolution of wells A4-D4 via OLS regression resulted in negative values (see SI). As such, NNLS regression results for the 24-well plate are shown in Figure 2d.

Importantly, with a run time of ∼0.3 min for a loop injection vs a standard 5 min LC method, a 24-well plate can be analyzed in 7.2 min vs 120 min which represents a 17-fold decrease in chromatography time, in addition to the time saved in method development.

These data were compared to additional analysis of each sample by LC-MS where each product peak was identified solely based on the MS isotopic distribution and was quantified via uncalibrated UV–vis.

Notably, the isotopic labeling readily identified when the elution order of the isomers was changed, which was observed in several cases (see SI). Additional product peaks were observed for the substrate **[D]5** arising from radical addition to the phenyl ring (labeled as RPh), and in the case of well D4 five unique products were identified from the LC-separated isotopic distributions (Figure 2f). Thus, it is best practice to incorporate a reaction from an unlabeled site in the matrix deconvolution if it is possible for the reaction to occur at other sites anywhere in the molecule. Uncalibrated total wavelength chromatograms (TWC) of product ratios are currently standard for rapid analysis of isomeric product ratios but do require LC separation and are subject to error if isomers have meaningfully different absorption values (1.7–1.9× measured for well A1). Notably, the matrix method gives fair agreement relative to TWC values (Figure 2e). While different ionization efficiencies of the isomers can cause errors in the matrix method, such differences are typically small (1.2–1.5× measured in the case of well A1, see SI) as the isomers have similar molecular volumes and charge distributions.^25^ Calibrated LC/UV or ^1^H NMR spectroscopic analysis of larger-scale reactions corresponding to wells A1, B2, and C3, show the results from the matrix MS values are as good or better relative to those obtained via TWC analysis (Figure 2e).

### Other sp^2^ and sp^3^ LSFs

**Figure 3:**
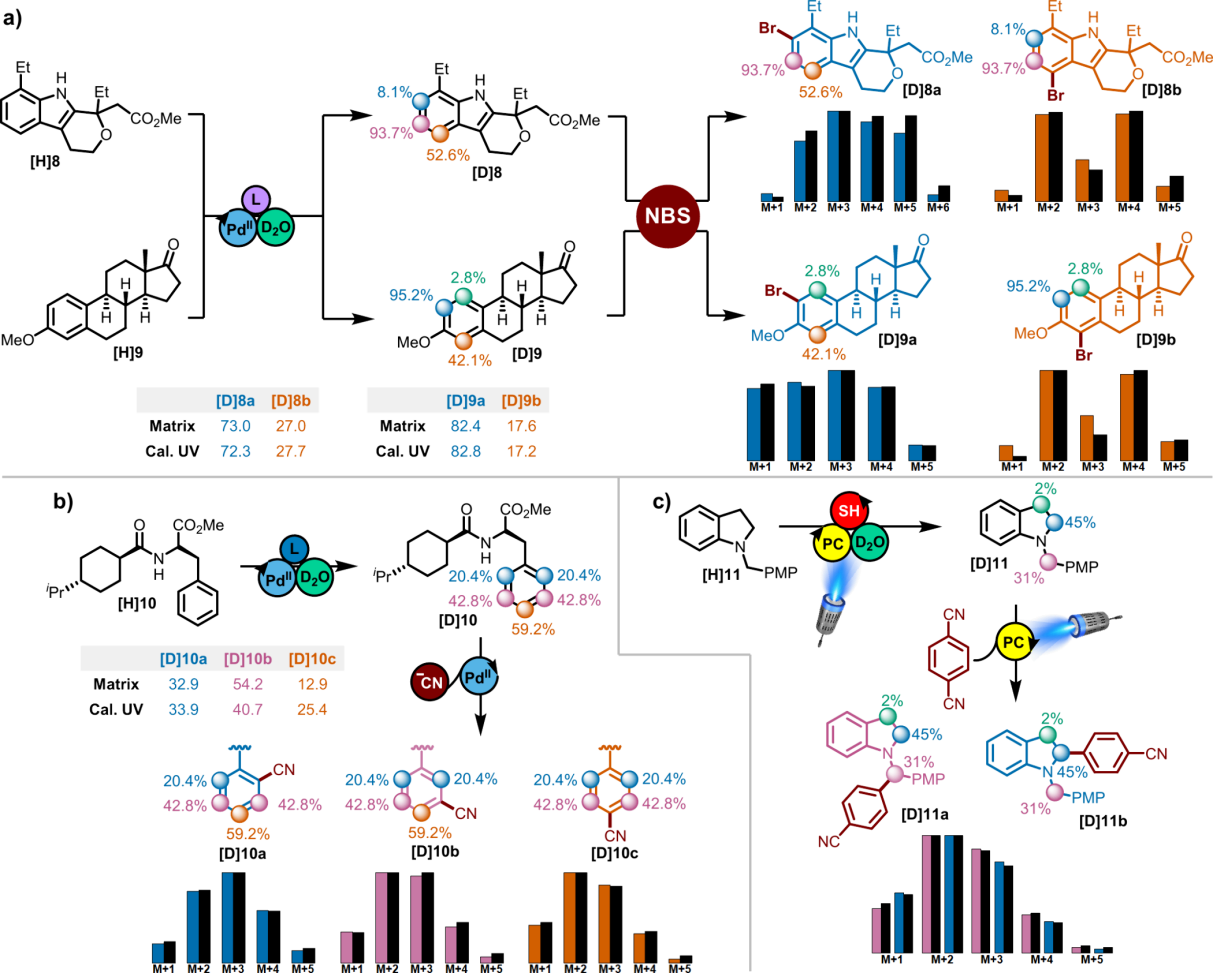
Other examples of sp^2^ and sp^3^ C–H functionalization. **a)** NBS bromination of **[D]8,9**. **b)** Pd^II^-catalyzed cyanation of **[D]10**. **c)** Photocatalytic arylation of **[D]11**. In all figures, predicted isotope distributions are depicted in black, while experimentally observed are shown with respective colored bars.

To further establish the utility of the method, a variety of targets for LSF were selected including **[H]8**–**11** (Figure 3). Isotopically labeled substrates **[D]8**–**10** were readily synthesized via a reversible Pd^II^ catalyzed C–H insertion in the presence of D_2_O.^26^ In the case of **[D]10** an alternate, more sterically encumbering, ligand was needed to provide different levels of deuteration. **[D]11**, was obtained using a photocatalyzed HAT mediated deuteration.^27^

Bromination of etodolac and estrone derivatives **[D]8**,**9** was accomplished using N-bromosuccinimide (NBS).^28^ The distinctive isotopic patterns easily allow the resultant products to be distinguished from one another and agree well with their predicted isotopic patterns (Figure 3a). Moreover, the deconvolution of unseparated materials exhibited excellent agreement with the calibrated UV–vis ratios. Increasingly complex LSFs were performed on nateglinide derivative **[D]10** (Figure 3b), and benzyl protected indoline **[D]11** (Figure 3c). Palladium catalyzed C–H cyanation^29^ of the sp^2^ centers in **[D]10** resulted in the formation of three products **[D]10a**–**10c**, with the LC peak for each being readily identified by their MS isotopic distributions. Furthermore, deconvolution of unseparated material led to excellent agreement with calibrated UV–vis ratios, despite **[D]10** having less distinction between positions compared to **[D]2**–**7** (∼16/22/39% vs ∼30/64/94%). The method was also effective with sp^3^ centers. Specifically, photocatalytic arylation of **[D]11**, via an α-amino radical intermediate,^30^ led to the formation of two products **[D]11a**,**11b**, both of which were identified through their MS isotopic distributions.

### Kinetic Isotope Effects

**Figure 4:**
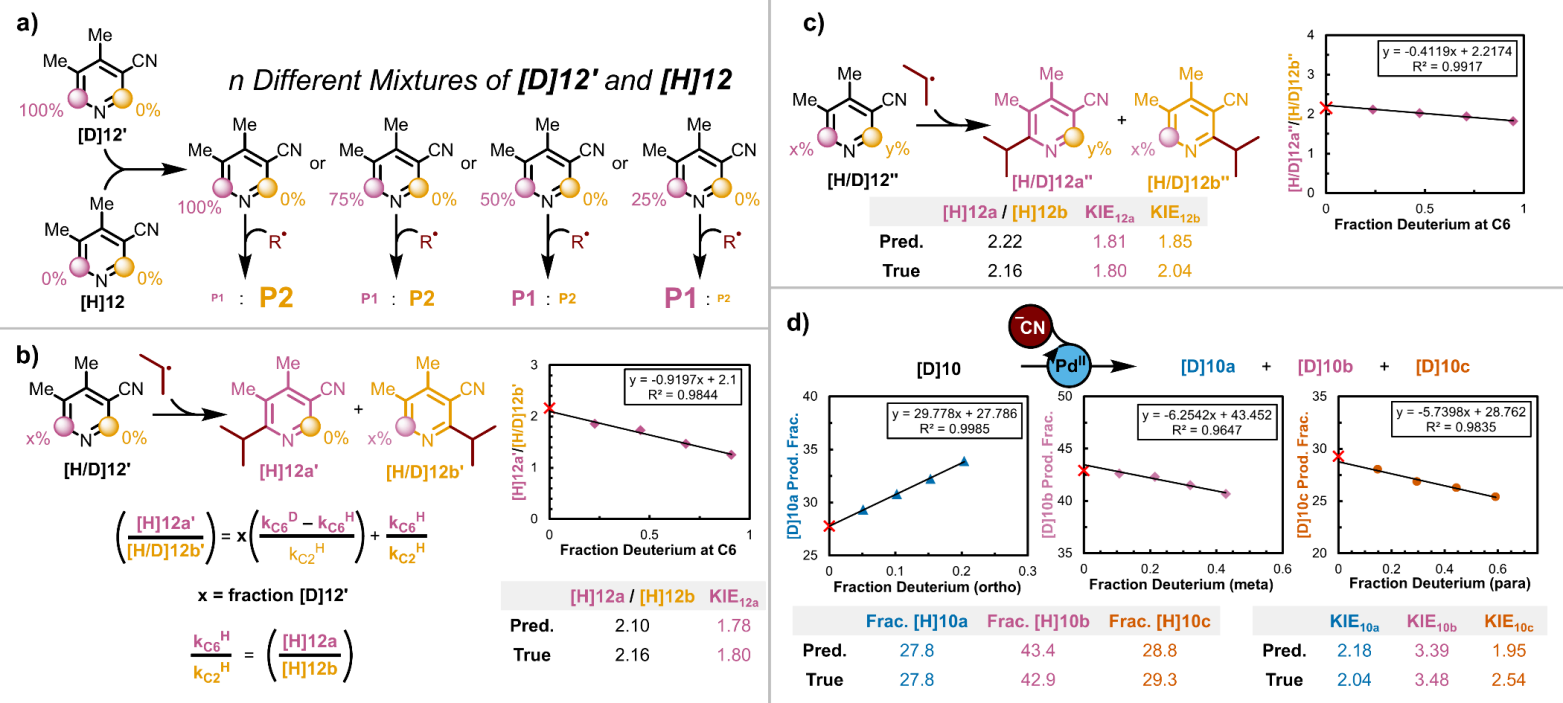
Kinetic isotope experiments. **a)** Example of dilution experiments. **b)** Simplified model system with one position partially labeled. **c)** More complex model with two positions partially labeled. **d)** Analysis of cyanation of **[D]10**. In all plots the red X marks the experimentally obtained product fraction or ratio for protio material.

When utilizing partially labeled materials, the product ratios from reactions exhibiting a kinetic isotope effect (KIE) are not representative of those observed with protio material. As a result of the decreased rate observed at deuterated centers, positions with a greater degree of deuterium incorporation will be underrepresented in the final product ratio. Depending on the degree of accuracy required, this effect need not be considered in many systems (if competitive KIE < 3) as the consequences of such isotope effects at low conversion are small (see SI). However, this technique can also be used to both obtain the inherent product ratios of the protio substrates as well as the competitive KIE values providing an opportunity to collect mechanistic information in a high throughput manner. Determination of the presence of a KIE in these systems is easily determined if there is a change in the MS isotope pattern of residual starting material. Furthermore, by diluting the original deutero substrate with unlabeled material as shown in Figure 4a, the effect of a KIE will diminish. As shown with the system of equations and graphically in Figure 4b, the data can be linearly extrapolated to identify the regioselectivity ratio of the unlabeled material (*y*-intercept, for **[H]12a**/**[H]12b**, this method = 2.10, standard from nondeuterated material = 2.16) and the slope can be used to determine the KIE value (this method = 1.78, standard competitive KIE method = 1.80). Similar analysis of multiple positions can also be done as in the case of pyridine **[H/D]12’’** (Figure 4c) and nateglinide derivative **[D]10** (Figure 4d). In both cases predicted product ratios show excellent agreement with experimentally determined values. Additionally, fair to excellent agreement is observed with KIE values determined using this method with those determined using traditional competitive KIE experiments. While this method is robust at determining product ratios, care should be taken with competitive isotope effects (KIE < 4) which can be sensitive to small changes in product ratios as was the case for Figure 4c−d.

## Outlook

The novel mass spectral approach to reaction analysis developed herein utilizing partially isotopically labeled substrates allows direct identification of multiple regioisomers from C–H functionalization reactions in LC workflows and is effective with both sp^2^ and sp^3^ hybridized centers. Alternative methods for regioisomer identification require time-consuming methods development, molecular modeling, lengthy acquisition times, the use of standards, or costly instrumentation. In contrast, this method may be implemented using conventional LC–MS methods with a range of detectors (single quadrupole and triple quadrupole) and rudimentary isotopic pattern calculations.^31^ Furthermore, the use of spectral deconvolution allows for the quantitation of regioisomeric ratios as well as relative reactivity without recourse to chromatographic separation, enabling rapid analysis. This method sets the stage for collection of both larger and richer data sets containing information about minor isomers that are typically disregarded when a “major” product is isolated. In doing so, opportunities will abound to develop methods that target “minor” isomers selectively. Additionally, competitive KIE values can also be collected by dilution with unlabeled substrates allowing facile mechanistic interrogation of large portions of reaction space. The resultant data is expected to be useful in the construction of predictive models across several dimensions including predicting reaction selectivity, predicting mass spectral ionization efficiencies, developing methods to identify isomers from mass spectral fragmentation patterns,^32^ and to expedite drug metabolite identification.^33^

## Supporting Information Available

Techniques for calculating deuterium incorporation, methods for analysis of isotopically labeled products, experimental procedures, product characterization, HRMS, NMR, and LC-MS spectral data (PDF)
Code S1 to S5 (ZIP)
FAIR data including the primary NMR FID files for compounds (**[H]1**-**12**, **[D]1**-**12**, **[H]2a-2c**, **[D]2a**-**2c**, **[H]3a**-**3c**, **[H]4a**-**4c**, **[H]8a**-**8c**, **[H]9a**-**9b**, **[H]10a-10c**, **[H]11a**-**11b**, **[D]11a**-**11b**, **[H]12a**-**12c**) (ZIP)
