# Supplementary material for: Direct (LC-)MS Identification of Regioisomers from C–H Functionalization by Partial Isotopic Labeling
Source: ACS Cent Sci. 2025 Feb 14;11(2):272–8. doi: 10.1021/acscentsci.4c01765 (PMC11868960; doi:10.1021/acscentsci.4c01765)
Supplement: Supplementary file 1 — oc4c01765_si_001.pdf [file oc4c01765_si_001.pdf]

Direct (LC-)MS Identification of Regioisomers from C-H Functionalization by Partial Isotopic Labeling

Authors: Christopher A. Sojdak[1], David A. Polefrone[1], Hriday M. Shah[1], Cassandra D. Vu[1], Brandon J. Orzolek[1], Pedro M. Jimenez Antenucci[1], Micah Valadez Bush[1], and Marisa C. Kozlowski[1]*

Affiliations: *Department of Chemistry, Roy and Diana Vagelos Laboratories, University of Pennsylvania, Philadelphia, Pennsylvania 19104-6323, United States*. *Corresponding author. Email: marisa@sas.upenn.edu (M. C. K)

This PDF file includes:

- Materials and Methods
- Figures S1 to S279
- Tables S1 to S30
- References

Other Supplementary Material for this manuscript includes the following:

- Code S1 to S5
- NMR Fair Data

1. General

1.1 Experimental Considerations and Instrumentation

1.2 LC/MS and MS Methods

1.2.1 UPLC-MS Parameters

1.2.2 MS-Only Parameters

1.2.3 MS Parameters for Both UPLC-MS and MS-Only

1.2.4 MS Data Analysis for Both UPLC-MS and MS-Only

1.2.5 Calculation of Isotope Patterns For Separated Materials

1.2.6 Deconvolution of Regioselectivity From MS-Only Analysis

1.2.6.1 Excel Ordinary Least Squares Deconvolution of Regioselectivity From MS-Only Analysis

1.2.6.2 Excel Non-Negative Least Squares Deconvolution of Regioselectivity From MS-Only Analysis

2. Methods for Deuterium Incorporation Calculation

Method 2.1 ^{1}H NMR Only

Method 2.2 ^{1}H NMR with MS Correction

Method 2.3 ^{13}C NMR

3. Reporting of ^{13}C Peaks in Deuterated Materials

4. Synthesis of Deuterated Pyridines

5. Reactions of Pyridines

5.1 Proof of Concept Minisci Reaction of [H]2 and [D]2 with *para*-Tolylboronic Acid

5.1.1 Reaction Conditions and Compound Characterization

5.1.2 UPLC-MS Analysis and Identification of Regioisomers from MS Isotope Patterns

5.1.3 Quantitation of Regioisomers from MS-Only Analysis

5.1.4 Quantitation of Regioisomers By Calibrated UPLC via TWC and TIC

5.2 24-Well HTE Plate: Pyridine Substrates [D]2–[D]7

5.2.1 General Procedure for HTE-Scale Minisci Arylations

5.2.2 UPLC Analysis of Regioselectivities for the 24-Well Plate

5.2.3 MS-Only Analysis of Regioselectivity for the 24-Well Plate

5.2.3.1 Description of MS-Only Analytical Methods

5.2.3.2 Selected MS-ONLY Data for 24-Well Plate

5.2.3.3 Variance in Isotope Patterns in MS-Only Measurements for 24-Well Plate

5.2.3.4 MS-Only Deconvolution Methods for 24-Well Plate

5.2.3.5 MS-Only Deconvolution Data for 24-Well Plate

5.2.4 Validation of Regioisomer Identities in 24-Well Plate

6. Synthesis of Deuterated of Arenes

7. Reactions of Arenes

7.1.1 Bromination of Etodolac Methyl Ester

7.1.2 MS-Only Analysis Etodolac Methyl Ester

7.2.1 Bromination of Estrone Methyl Ether

7.2.2 MS-Only Analysis Estrone Methyl Ether

7.3 Cyanation of Nateglinide Methyl Ester

8. Deuteration of sp^3 Centers

8.1 Deuteration of 1-(4-Methoxybenzyl)indoline

9. Reaction at sp^3 Centers

9.1 Reaction of 1-(4-Methoxybenzyl)indoline

10. Kinetic Isotope Effects

10.1 Pyridine Kinetic Isotope Effects

10.1.1 Synthesis of Pyridine Substrates

10.1.2 Synthesis of Pyridine Product Standards

10.1.3 Intermolecular Kinetic Isotope Experiments [D]12

10.1.4 Reactions with Singly Deuterated Pyridine

10.1.5 Reactions with Doubly Deuterated Pyridine

10.1.6 Derivations of KIE with Singly Deuterated Pyridine

10.1.7 Derivations of KIE with Doubly Deuterated Pyridine

10.2 Nateglinide Methyl Ester Kinetic Isotope Effects

10.2.1 Intermolecular Kinetic Isotope Experiment Nateglinide Methyl Ester

10.2.2 Reactions of Triply Deuterated Nateglinide Methyl Ester

10.2.3 MS-Only Analysis Nateglinide Methyl Ester

10.2.4 Derivations of KIE with Triply Deuterated Nateglinide Methyl Ester
10.3 Effect of Conversion on Regioselectivity
11. NMR Spectra

1. General

1.1 Experimental Considerations and Instrumentation

All reactions were performed under an argon atmosphere unless otherwise noted with oven-dried glassware using standard Schlenk or vacuum line techniques. All reactions mixtures were heated using RCT Digital IKA Magnetic Stirrer Plates. Dry and degassed CH_2Cl_2, THF, and Et_2O were obtained using a Glass Contour Solvent System manufactured by Pure Process Technology, LLC. Commercially available reagents were obtained from Sigma-Aldrich, TCI America, or Acros Organics, and solvents were obtained from Fisher Scientific. Ligands **L1**, and **L2** were synthesized using previously established literature.[1] Analytical thin-layer chromatography (TLC) was used to monitor reaction progress and was performed using Silicycle 250 μm precoated 60 Å silica gel plates with F254 indicator using shortwave ultraviolet light to visualize. Automated flash chromatography was performed using a Teledyne ISCO CombiFlash® (254 nm & 280 nm UV detector) with RediSep Rf Gold® disposable columns (60 Å porosity, 20–40 μm) or flash chromatography with forced flow of the indicated solvent system on Silica-P flash silica gel (50-63 μm mesh particle size).

NMR spectra were recorded on a Fourier transform (Bruker NEO) NMR spectrometer equipped with SampleCase at 298 K. ^{1}H NMR spectra were obtained at 400 or 600 MHz. Decoupled ^{13}C{^{1}H} NMR spectra were recorded at 101 or 151 MHz. Chemical shifts are reported in parts per million (ppm) from the solvent resonance, $CDCl_3$ 7.26 ppm, acetone-d_6 2.05 ppm, DMSO-d_6 2.50 ppm for ^{1}H NMR, and $CDCl_3$ 77.16 ppm, acetone-d_6 29.84 ppm, DMSO-d_6 39.52 ppm for ^{13}C{^{1}H} NMR. To ensure accurate deuterium incorporation ^{1}H NMR spectra of deuterated materials were performed with a relaxation delay (d1) time of 20–30s, the spectra for the corresponding protio materials were performed with a d1 time of 1s unless otherwise noted. Data are reported as follows: chemical shift, multiplicity (s = singlet, d = doublet, t = triplet, dd = doublet of doublets, ddd = doublet of doublets of doublets, dt = doublet of triplets, q = quartet, m = multiplet), coupling constants, and number or protons. In isotopically labeled species, peaks in the ^{13}C{^{1}H} NMR can experience isotopic shifting as well as splitting (see **Section 3. ^{13}C{^{1}H} NMR** for further discussion). Deuterated solvents, $CDCl_3$, DMSO-d_6, and acetone-d_6 were obtained from Cambridge Isotope Laboratories, Inc., while D_2O was provided by Thermo Fisher Scientific. Infrared (IR) absorption spectra were taken on a FT-IR spectrometer (ATR, neat).

High-resolution accurate mass measurement analyses were conducted on either a GCMS with electron ionization (EI) or an LCMS with electrospray ionization (ESI). The signals were mass measured (TOF) against an internal lock mass reference of perfluorotributylamine (PFTBA) for EI-GCMS, and leucine enkephalin for ESI-LCMS. Waters software calibrates the instruments, and reports measurements, by use of neutral atomic masses; the mass of the electron is not subtracted (positive ions) or added (negative ions).

The isotopically labeled materials were analyzed on a Waters Aquity SQ (single-quadropole) or a Waters Aquity TQ (triple-quadropole). Mass spectral patterns of substrates and products were calculated for isotopically labeled materials using a modified version of EnviPat[2] where placeholder elements (a, b, c, etc.) added to the isotope library were used for each unique hydrogen reflecting the level of deuterium incorporation.

1.2 LC/MS and MS Methods

1.2.1 UPLC-MS Parameters

Liquid chromatography-mass spectrometry was performed on a Waters ACQUITY SQ (single-quadrupole) mass detector equipped with a Waters Photodiode Array (PDA) detector or on a Waters ACQUITY TQ (triple-quadrupole) mass detector equipped with a Waters Photodiode Array (PDA) detector.

Analytical-scale liquid chromatography was performed using a Waters ACQUITY Premier BEH columns (Aquity Premier BEH C18; 2.4 µm; 50mm x 2.1mm) or Waters AQUITY UPLC HSS column (AQUITY UPLC HSS C18; 1.8 µm; 50mm x 2.1mm or AQUITY UPLC HSS C18; 1.8 µm; 150mm x 2.1 mm). Custom chromatographic gradients were developed for individual reactions and will be noted on a case-by-case basis. The default UPLC inlet flow rate was 0.75 mL/min. Mobile phase: MeCN:H_2O:0.1% formic acid (FA). Detection wavelength: 190-400 nm. Positive-mode electrospray ionization (ESI+); nebulizer: 700 L/hr; cone gas (N_2): 30 L/hr; source 150 °C; desolvation 450 °C.

Unless otherwise stated gradient curves were set to 6 (linear). A summary of curve settings is shown below.

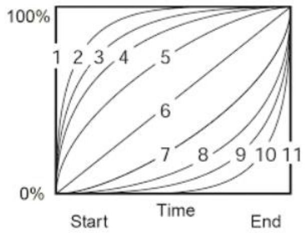

Curve Setting	2	3	4	5	6	7	8	9	10
Formula	$X^{1/5}$	$X^{1/4}$	$X^{1/3}$	$X^{1/2}$	X	X^2	X^2	X^4	X^5

For collection of mass spectral from analytical chromatograms, it is necessary to integrate across the entire peak as deuterated isomers elute more quickly.[3] Alternately, use of the peak centroid was found to give consistent results in cases where peaks overlap.

Where possible, replicate trials were performed for UPLC-MS analytical runs. For any peak intensity measured across replicate trials, precision was assessed by the relative standard deviation (RSD) of peak intensities, defined as the standard deviation divided by the average. For normalized intensities (base peak 100.00) this results in RSD values of 0.00 by convention. Generally, RSD values of <5% were achievable, with RSD values as low as 1% consistently observed for the 24-well arylation plate described below.

1.2.2 MS-Only Parameters

When applicable, loop injections were performed in column bypass mode ("MS-only"). Standard inlet conditions for loop injections: 0.25 mL/min flow rate with a 0.3-minute-long isocratic run (50:50 MeCN:H_2O:0.1% FA). Positive-mode electrospray ionization (ESI+); nebulizer: 700 L/hr; cone gas (N_2): 30 L/hr; source 150 °C; desolvation 450 °C.

Minimal differences have been observed between mass spectral peak intensities obtained via loop injection versus those obtained via liquid chromatography, so long as mass spectra are appropriately combined across the full width of a peak.

1.2.3 MS Parameters for Both UPLC-MS and MS-Only

Electrospray-positive (ESI+) ionization was used with quadrupole MS detectors. Similar principles apply to the collection of data using ESI- and should extend to other atmospheric pressure ionization sources as well as higher-resolution mass detectors. Stronger ionization sources (electron/chemical sources) may require additional consideration of fragmentation patterns.

The **cone voltage** for a given compound should be set so that compound fragmentation is minimized while maximizing ion counts. Cone voltages of 10-60 V have been employed, depending on the specific analyte. The standard operating cone voltage is 30 V and was the default setting.

"**Peak splitting**" can occur such that a MS peak has a meaningful satellite within ~1 *m/z* (for example, a peak at *m/z* 300.50 with normalized intensity 100.00, with another peak at *m/z* 300.81 with normalized intensity 9.00). This is usually a consequence of detector overload and was mitigated by optimizing analytical parameters for a given run. Satellites with minor normalized intensities (≤ 2.00 normalized) were disregarded. For runs with especially low overall ionization, the largest peak in each decimal cluster was selected.

The **scan time** should be set at 0.1 sec or less (corresponding to scan rates of ≥ 10 Hz) to maximize the number of points collected by the MS detector, 0.05 sec (20 Hz) was used as a standard for quantitative analysis. Additionally, for quantitative analysis of isotopic distributions, the mass-to-charge (*m/z*) range was confined to ~20 Da. This enables additional scans in the *m/z* region of interest. Note that total ion count (TIC) peak heights will be altered by both scan time and *m/z* range, and additional care should be taken to avoid detector overload when changing these parameters. While selective ion recording (SIR) methods may be employed instead of narrow *m/z* ranges, such methods were usually not employed here unless otherwise noted.

Optimal **TIC peak heights** are between 1E7 and 1.5E8, but this is not always feasible depending on the compound/conversion. For our instrumental setups, we determined that TIC peak height of >1.5E8 led to potential detector overload and less reliable quantitative results. Reducing the injection volume and/or sample concentration alleviated this where necessary. Signal-to-noise (S/N) of isotopic clusters appeared to worsen below 1E7 TIC peak height due to low sensitivity.

Standardized sample concentrations and volumes were used where possible to maintain reproducibility. For a neutral compound, more basic moieties improve the ESI+ response[4] and allow for either lower sample concentrations or smaller injection volumes of sample. Injection volume (IV) could be varied between 0.1 and 7.0 µL given our analytical configurations, depending on sample concentration, generally between 0.100 mM – 4 mM with more concentrated samples being used for poorly ionizing substrates.

1.2.4 MS Data Analysis for Both UPLC-MS and MS-Only

Mass spectral data is acquired in the centroided mode. Manual inspection of mass chromatograms was performed using MestReNova (MNova). When using MNova, crosshair selection was applied across a full peak of interest (baseline to baseline) to obtain the full isotopic distribution for a given analyte. Isotopic peaks were selected in table view under the Analysis tab of MNova and copied into Excel/R (two decimal places for normalized intensities).

Alternatively, **automated extraction of mass chromatograms** was performed by converting raw MS output file directories to mzML[5] files using the MSConvert[6] functionality within the ProteoWizard[7] suite, followed by data processing developed with custom workflows in R. Key dependencies for this workflow include mzR[8], msdata[9] and MSnbase[10]. Minimal differences were observed between manual and automated routines for extracting MS data. Alternative means of processing continuum data may yield slightly different numerical results and were ruled out due to excessive file sizes.

Ionization differences between labeled/unlabeled species are assumed to be negligible. Since regioisomeric species have similar molecular volume and pK_a, they are expected to exhibit similar relative ionization efficiency (RIE) in the ESI+ mode.[4] We have observed small differences in ionization efficiencies between regioisomers via calibration of a model reaction, although inclusion of these values for deconvolution of regioselectivity was found not to improve quantitative agreement.

Raw intensities are relevant when benchmarking absolute ionization efficiency. We refer to the “unnormalized intensities” for a given cluster of mass peaks as the set of intensities for each respective peak, divided by the sum of all relevant peaks. By doing this, the unnormalized intensities sum to 100%.

1.2.5 Calculation of Isotope Patterns For Separated Materials

The calculation of predicted isotopic distribution patterns for partially deuterated materials is done via a modified version of the R package enviPat[2] named IsotopicDistributionCode.R (included as Supplementary Information). To accommodate the partial deuterium labels, custom elements are added to the list of default isotopic abundances in enviPat. For example, if one position contains 10% deuterium and 90% proton, a new element with this distribution is created and added to the list of isotopic abundances that enviPat. When inputting the molecular formula, one hydrogen is then replaced with this custom element.

An example for calculating the isotopic distribution of partially deuterated nicotinic acid (**[D]1**) is shown below.

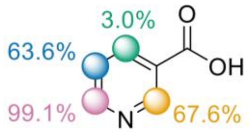

1) On line 206, input the chemical formula of the parent protio substrate to be analyzed in the quotation marks. Every atom must be explicitly numbered even if there is just one in the structure. For **[D]1** the parent protio chemical formula is: C6H5N1O2 and can be input as follows.

```
205 ##########################################
206 SM_formula <- "C6H5N1O2"
207 ##########################################
```

2) On line 218, input the chemical formula of the isotopically labeled substrate in the quotation marks. To do this remove however many isotopically labeled positions from the amount of hydrogen in the protio chemical formula. For example, **[D]1** has 4 labeled positions so H5 will be set to H1. Now placeholder values will be used for these labeled positions as follows: a1, b1, c1, d1, etc. Each letter denotes a unique deuterium label, while the number corresponds to the number of labels at that position. For example -CH_3 would be labeled as a3. For **[D]1** we will input "C6H1a1b1c1d1N1O2".

```
217 ##########################################
218 SM_isotopic_formula <- "C6H1a1b1c1d1N1O2"
219 ##########################################
```

3) On line 198, the fractional deuterium levels are input (100% should be entered as 1). From left to right each value input will correspond to the placeholder values used in the previous step in alphabetical order (1st value = a#, 2nd value = b#, 3rd value = c#, etc.). For **[D]1** these values and the corresponding placeholders will be: 0.676, 0.030, 0.636, 0.991, corresponding to a1, b1, c1, and d1 respectively.

```
197 ##########################################
198 SM_D_levels <- c(0.676,0.030,0636,0.991)
199 ##########################################
```

4) Highlight line 1-233 and run the code. The isotopic distribution will now appear in the console.

```
[[1]]
[1] 124 125 126 127 128

[[2]]
[1]   0.4154698  46.6146907 100.0000000   9.6828812   0.7513503
```

1.2.6 Deconvolution of Regioselectivity From MS-Only Analysis

The isotopic distribution for a mixture of labeled species may be represented as a weighted combination of the isotopic distributions for each component:

$$\boldsymbol{Cw} = \boldsymbol{p}$$

Where **C** represents "components" of the isotopic distributions for each individual product, **w** represents "weight" of each product, and **p** represents the "product" vector distribution from the mixture of products. For a compound that is deuterated at four positions and that undergoes reaction at three of those sites (positions 2, 4, 6), MH+ will consist of M+1 to M+5 isotopes resulting in the following matrix:

$$\begin{bmatrix} C1_2 & C1_4 & C1_6 \\ C2_2 & C2_4 & C2_6 \\ C3_2 & C3_4 & C3_6 \\ C4_2 & C4_4 & C4_6 \\ C5_2 & C5_4 & C5_6 \end{bmatrix} \begin{bmatrix} w_2 \\ w_4 \\ w_6 \end{bmatrix} = \begin{bmatrix} P1_{CR} \\ P2_{CR} \\ P3_{CR} \\ P4_{CR} \\ P5_{CR} \end{bmatrix}$$

The **C** matrix is obtained by using NMR measurements of site-specific deuterium incorporation values using the IsotopicDistributionCode described above. These values are referred to as $\mathbf{C_{NMR}}$.

In cases where the mass spectra have been measured for separated product isomers (such as those obtained in Section 5.2.2), the resultant isotopic distributions can be used instead. These values are referred to as $\mathbf{C_{LC}}$. The differences between $\mathbf{C_{NMR}}$ and $\mathbf{C_{LC}}$ values arise from small errors in the NMR measurements. As the $\mathbf{C_{LC}}$ values represent the actual measured values that are aggregated in the loop analyses, slightly enhanced accuracy is obtained. Use of these values is especially useful if multiple runs will be done with the same substrates; for example, looking at many different reaction conditions.

For any give reaction, the isotopic distributions (**C**) can thus be calculated or measured for each respective regioisomer. These values were copied into Excel for manual ordinary least squares (OLS) and non-negative least squares (NNLS) regression analysis. Deconvolution was also performed using OLS and NNLS in R (**Section 5.2.3.4**). These methods typically gave identical results unless noted (**Section 5.2.3.4**). NNLS is recommended as negative weights have no physical meaning.

Other regression specifications such as weighted least squares (WLS) regression, with or without non-negativity as a constraint, may also be used.

1.2.6.1 Excel Ordinary Least Squares Deconvolution of Regioselectivity From MS-Only Analysis

The deconvolution workflow in Excel is illustrated in file OLS_NNLS_Deconvolution.xlsx for reaction well **A1** from **Section 5.2** below. The MS peak intensities for M+1 to M+5 obtained in the MS-Only analysis (LOOP) are used to populate the **p** vector (O7:O10). Values are the average of the normalized intensities for the reaction mixture of reaction well **A1** across triplicate measurements.

A1 Loop Injection Replicate 1		
A1	R1	IV 0.2 μL
monosub	m/z	norm
M+1	228.48	8.77
M+2	229.46	57.87
M+3	230.46	100.00
M+4	231.46	53.89
M+5	232.48	7.65
TIC height	9.49E+07	

A1 Loop Injection Replicate 2		
A1	R2	IV 0.2 μL
monosub	m/z	norm
M+1	228.48	8.82
M+2	229.46	57.57
M+3	230.46	100.00
M+4	231.46	54.07
M+5	232.48	7.60
TIC height	1.03E+08	

A1 Loop Injection Replicate 2		
A1	R3	IV 0.2 μL
monosub	m/z	norm
M+1	228.48	8.91
M+2	229.46	57.84
M+3	230.46	100.00
M+4	231.46	54.30
M+5	232.48	7.74
TIC height	9.63E+07	

A1 Average of Three Replicates			
A1			
	AVG	SD	RSD
M+1	8.83	0.07	0.8%
M+2	57.76	0.17	0.3%
M+3	100.00	0.00	0.0%
M+4	54.09	0.21	0.4%
M+5	7.66	0.07	0.9%
TIC height	9.81E+07	4.33E+06	4.4%

The 5x3 matrix T7:V11 is the $\mathbf{C_{NMR}}$ matrix. Unnormalized intensity values must be used for deconvolution rather than normalized intensity values. This is necessary since base peaks reflect meaningfully different intensities for different regioisomers. Renormalized values are shown (W7:Y11) for comparison. Total D values (W12:Y12) were calculated using an implementation of

IsoPat[2] (see below) to verify that the values agreed with those expected from the NMR measurements of the starting material used.

AB7 {=MMULT(MMULT(MINVERSE(MMULT(TRANSPOSE($T$7:$V$11),$T$7:$V$11)),TRANSPOSE($T$7:$V$11)),O7:O11)}

	T	U	V	W	X	Y	Z	AA	AB	AC	AD
5	C-NMR 1			*renormalized*				A1		NMR, no correction	
6	R2	R4	R6						*OLS*	rr	rel%
7	0.26%	0.09%	9.02%	0.45	0.21	20.62		R2	28.50	1.00	12.5%
8	29.04%	9.82%	38.04%	50.11	22.39	86.97		R4	95.73	3.36	41.9%
9	57.95%	39.78%	43.74%	100.00	90.70	100.00		R6	104.31	3.66	45.6%
10	11.55%	43.86%	8.38%	19.93	100.00	19.16					
11	1.19%	6.46%	0.82%	2.05	14.73	1.87					
12			*total D*	1.63	2.28	1.34					

The highlighted cell displays the formula used for matrix OLS regression in Excel. Comparable results could be obtained using LINEST or other built-in functionalities. Solving the matrix linear regression for this case give the **w** vector (amounts of each isomer):

$$\begin{bmatrix} C1_2 & C1_4 & C1_6 \\ C2_2 & C2_4 & C2_6 \\ C3_2 & C3_4 & C3_6 \\ C4_2 & C4_4 & C4_6 \\ C5_2 & C5_4 & C5_6 \end{bmatrix} \begin{bmatrix} w_2 \\ w_4 \\ w_6 \end{bmatrix} = \begin{bmatrix} P1_{CR} \\ P2_{CR} \\ P3_{CR} \\ P4_{CR} \\ P5_{CR} \end{bmatrix}$$

$$\begin{bmatrix} 0.26\% & 0.09\% & 9.02\% \\ 29.04\% & 9.82\% & 38.04\% \\ 57.95\% & 39.78\% & 43.74\% \\ 11.55\% & 43.86\% & 8.38\% \\ 1.19\% & 6.46\% & 0.82\% \end{bmatrix} \begin{bmatrix} w_2 \\ w_4 \\ w_6 \end{bmatrix} = \begin{bmatrix} 8.83\% \\ 57.76\% \\ 100.00\% \\ 54.09\% \\ 7.66\% \end{bmatrix}$$

$$\begin{bmatrix} w_2 \\ w_4 \\ w_6 \end{bmatrix} = \begin{bmatrix} 28.50 \\ 95.73 \\ 104.31 \end{bmatrix}$$

Given these raw coefficients from OLS regression in **w** (AB7:AB10), renormalization (minimum = 1.00) yields the corresponding regioselectivity ratio (AC7:AC10). Product percentages (out of 100%) can also be generated (AD7:AD10). A corollary to the renormalization procedure for **w** is that the choice of unnormalized versus normalized intensities for the **p** vector does not affect the numerical results of the deconvolution procedure.

This system was also analyzed using CLC values from **Section 5.2.2**. Given the observed values in AI7:AK11 (taken directly from the corresponding UPLC-MS run for A1), unnormalization was then performed to obtain the appropriate $\mathbf{C}_{\mathbf{LC}}$ matrix (AF7:AH11). Application of OLS generates the results in AN7-AP9. The relative percentages line up well with those from the $\mathbf{C}_{\mathbf{NMR}}$ matrix above.

AN7 {=MMULT(MMULT(MINVERSE(MMULT(TRANSPOSE($AF$7:$AH$11),$AF$7:$AH$11)),TRANSPOSE($AF$7:$AH$11)),O7:O11)}

	AF	AG	AH	AI	AJ	AK	AL	AM	AN	AO	AP	AQ
5	C-LC 1 (A1)			*renormalized*				A1		LC, no correction		
6	R2	R4	R6						*OLS*	rr	rel%	
7	0.17%	0.34%	11.90%	0.32	0.80	28.10		R2	54.70	1.00	24.0%	
8	33.44%	11.65%	36.49%	61.31	27.54	86.18		R4	98.41	1.80	43.1%	
9	54.54%	39.39%	42.34%	100.00	93.10	100.00		R6	75.11	1.37	32.9%	
10	10.81%	42.31%	8.47%	19.82	100.00	20.01						
11	1.04%	6.30%	0.80%	1.91	14.89	1.88						
12			*total D*	1.63	2.28	1.34						

1.2.6.2 Excel Non-Negative Least Squares Deconvolution of Regioselectivity From MS-Only Analysis

In some instances, such as the reaction illustrated below (well **A4** from **Section 5.2**), OLS analysis returns negative values for **w**. For these cases, non-negative least squares analysis can be performed to constrain **w** such that the values cannot be negative. The deconvolution workflow in Excel is illustrated in file OLS_NNLS_Deconvolution.xlsx and is described below.

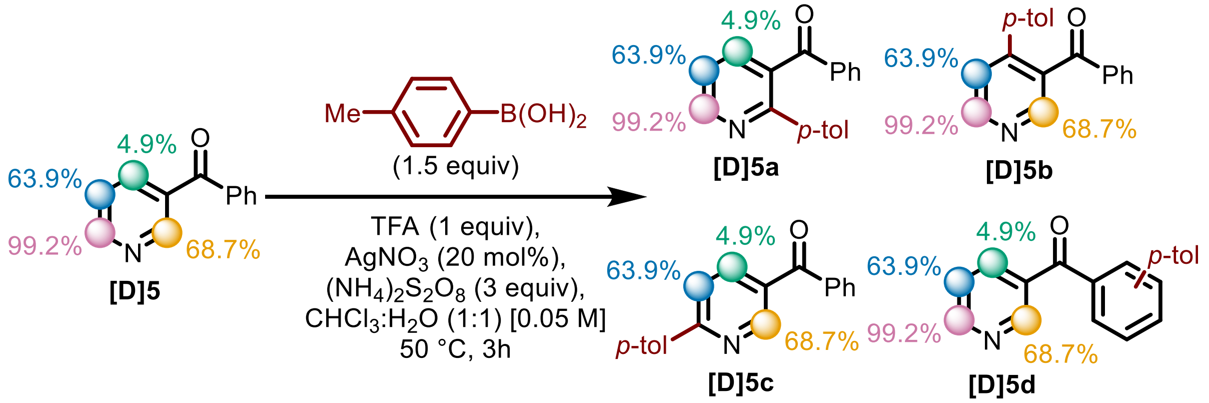

First, the predicted isotopic patterns for all isomers are obtained using methods previously discussed. In this case, $\mathbf{C}_{\mathbf{NMR}}$ values are entered into C10:F14.. These values must be unnormalized (sum of peaks = 100) to obtain I10:L14:

I10 | =100*C10/(SUM(C$10:C$14))

	B	C	D	E	F	G	H	I	J	K	L
7											
8											
9	normalized	[D]5a	[D]5b	[D]5c	[D]5d		unnormalized	[D]5a	[D]5b	[D]5c	[D]5d
10	M+1	0.397	0.170	20.205	0.163		M+1	0.223	0.074	8.740	0.071
11	M+2	49.874	21.768	85.305	20.824		M+2	28.094	9.481	36.899	9.058
12	M+3	100.000	88.630	100.000	85.826		M+3	56.330	38.604	43.255	37.334
13	M+4	24.414	100.000	23.113	100.000		M+4	13.752	43.556	9.998	43.500
14	M+5	2.842	19.021	2.563	23.074		M+5	1.601	8.285	1.109	10.037

The data gathered from the LOOP injection are then entered into O10-O14. Next, arbitrary values (1) are entered initially for the weight of each isomers in cells R10-V10.

	N	O	P	Q	R	S	T	U	V
8									
9		LOOP			w[D]5a	w[D]5b	w[D]5c	w[D]5d	weight sum
10	M+1	7.06		weights	1	1	1	1	4
11	M+2	53.89		normalized weights	25	25	25	25	100
12	M+3	100							
13	M+4	57.4							
14	M+5	10.79							

Because each M+x value is a weighted average of all regioisomers it can be expressed as:

$$(M+x) = (M+x)_a w_a + (M+x)_b w_b + (M+x)_c w_c + (M+x)_d w_d$$

A formula is made for each M+x value to represent the right-hand side of the above equation (T14:T18).

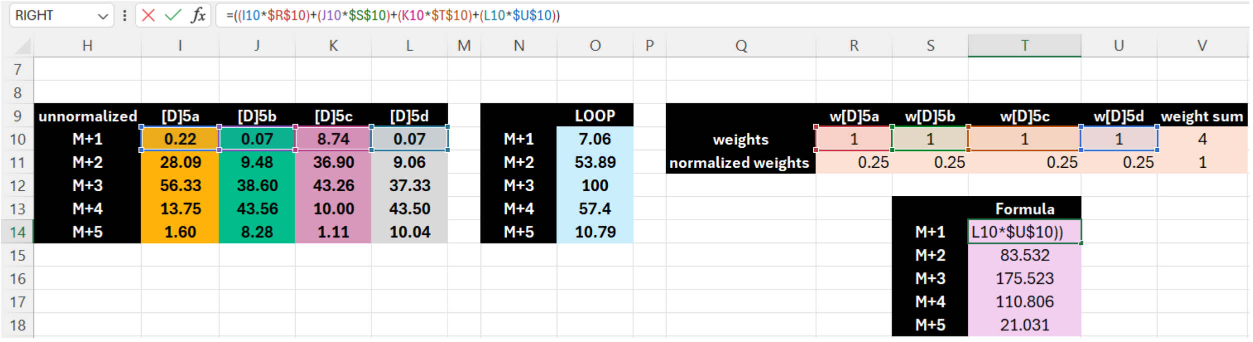

With this formula in hand, the Solver function in Excel found in the Data tab is selected.

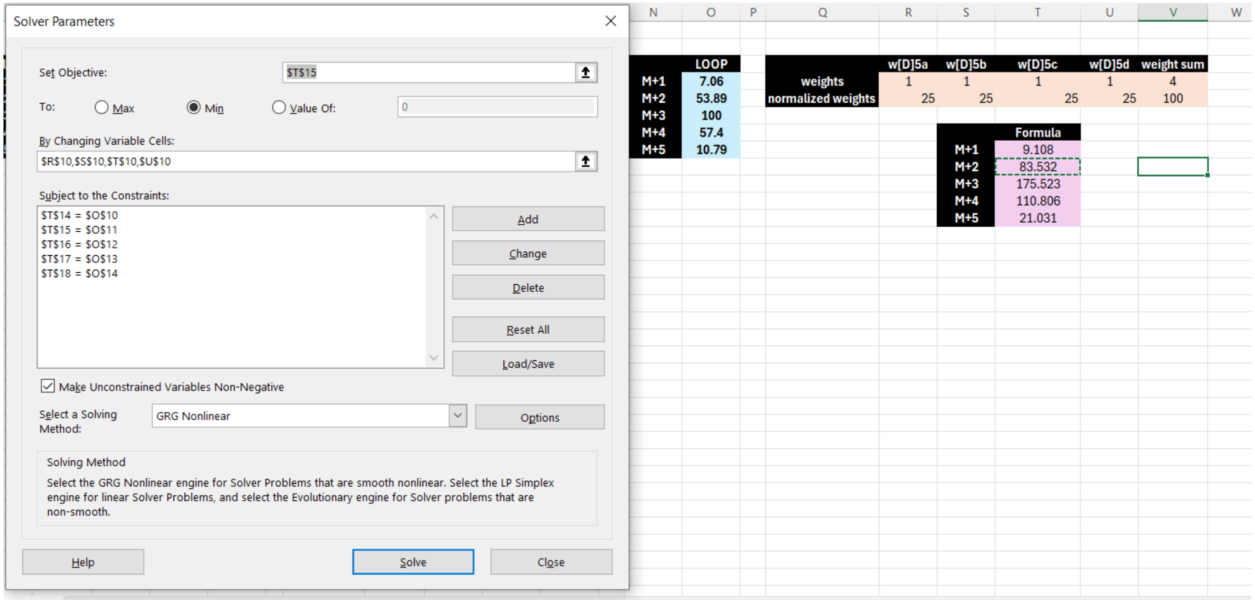

The objective is one of the cells in the “Formula” column and it is set to “Min” so the solver will attempt to minimize values in the formula column and prevent overestimation. The “Changing Variables Cells” are the variables that will be calculated, in this case the cells corresponding to the amount (weight) of each regioisomer: **w[D]5a**, **w[D]5b**, **w[D]5c**, and **w[D]5d** (cells: R10:U10). To cause the values in the formula cell to be equal to the experimentally observed values, constraints are added so that each M+x value in the formula cell must equal the equivalent M+x value in the experimental data cells. When all the constraints are input, the “Make unconstrained variables non-negative” box is checked, which will prevent negative values for the regioisomer weights. GRG Nonlinear was used as the solving method.

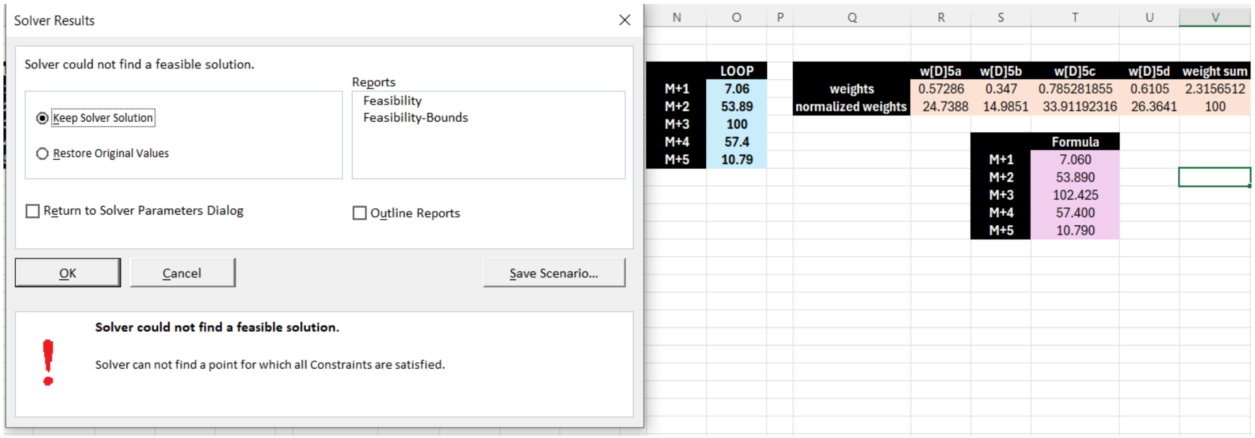

After clicking solve, a window will appear and if a perfect match cannot be found it will give whichever values result in the closest fit. The variables input previously will automatically update to the solved values (R10:U10), which can be renormalized to give regioisomeric ratios(R11:U11).

2. Methods for Deuterium Incorporation Calculation

isoPat[2]

The total amount of deuterium in a compound (D value) can be calculated from the MS isotope patterns of the labeled material. Using normalized ratios (largest peak = 100), D values were calculated using an implementation of IsoPat[2] [11, 12] written in R and then copied over to Excel. This value does not account for positional information. This self-contained program is included in the Supplementary Information (isoPat2modified.xlsm).

An example of use of the program is shown below for partially deuterated nicotinic acid:

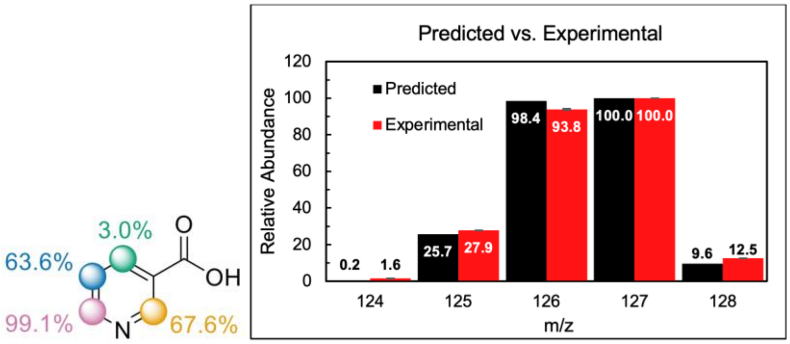

1) In B14-B16, the normalized MS abundance pattern for unlabeled nicotinic acid is entered (largest peak = 100): 100.00, 6.85, 0.59.

2) In E14-E18, the normalized MS abundance pattern for labeled nicotinic acid (red bars in bar plot above) is entered.

3) In K12, the number of possible isotopologues for the labeled material is entered which is the number of positions that can be deuterated plus one. Since the carboxylic acid is not deuterated in this instance, there are four possible positions that can be labeled resulting in five possible isotopologues (D_0, D_1, D_2, D_3, D_4).

4) Press the “Calculate” button. The output appears in T30 as 2.334 for this data.

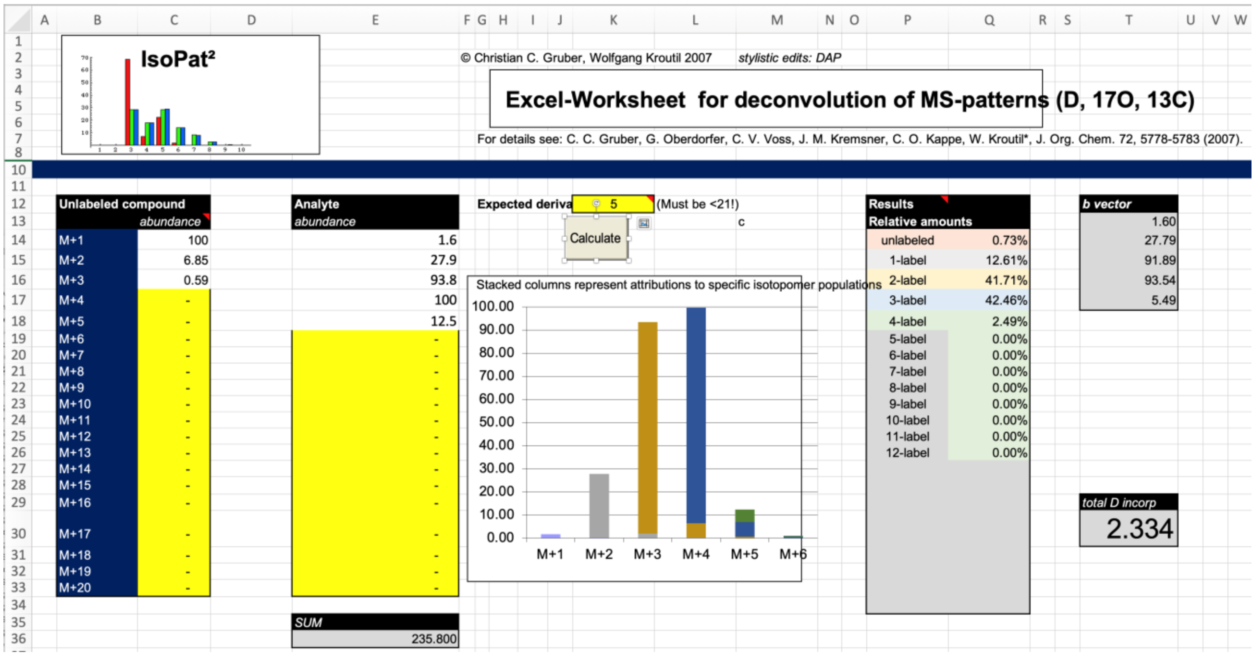

Method 2.1 ^{1}H NMR Only

By integrating an internal reference peak which is known to have no deuterium incorporated on that position (confirmed by ^{13}C {^{1}H} NMR)[13-15], for example, the methyl ester in **D-methyl nicotinate**. The readout from the integration corresponds to the amount of hydrogen remaining, percent deuterium is then calculated as follows: $\%D = 100 * (1 - integration)$. The ^{13}C satellites are only included in the integration if they are unobstructed in both the reference peak and all deuterated peaks. The total amount of deuterium present is equal to the number of hydrogens integrated in the ^{1}H NMR spectrum of the nondeuterated sample minus the number of hydrogens integrated in the ^{1}H NMR spectrum of deuterated sample.

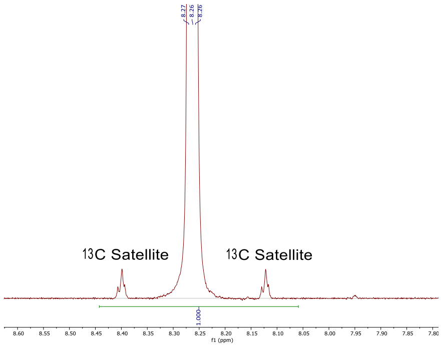

Method 2.2 ^{1}H NMR with MS Correction

Due to the inherent error present in the gravimetric addition of an internal standard an alternative method for determining %D was used for compounds which do not have an internal reference peak. In the example below, positions H2, H4, H5, and H6 are quantitated where the integration of each corresponds to 1H in nondeuterated material. If the integration is higher (e.g. methylenes, methyls), the analysis below needs to be modified accordingly:

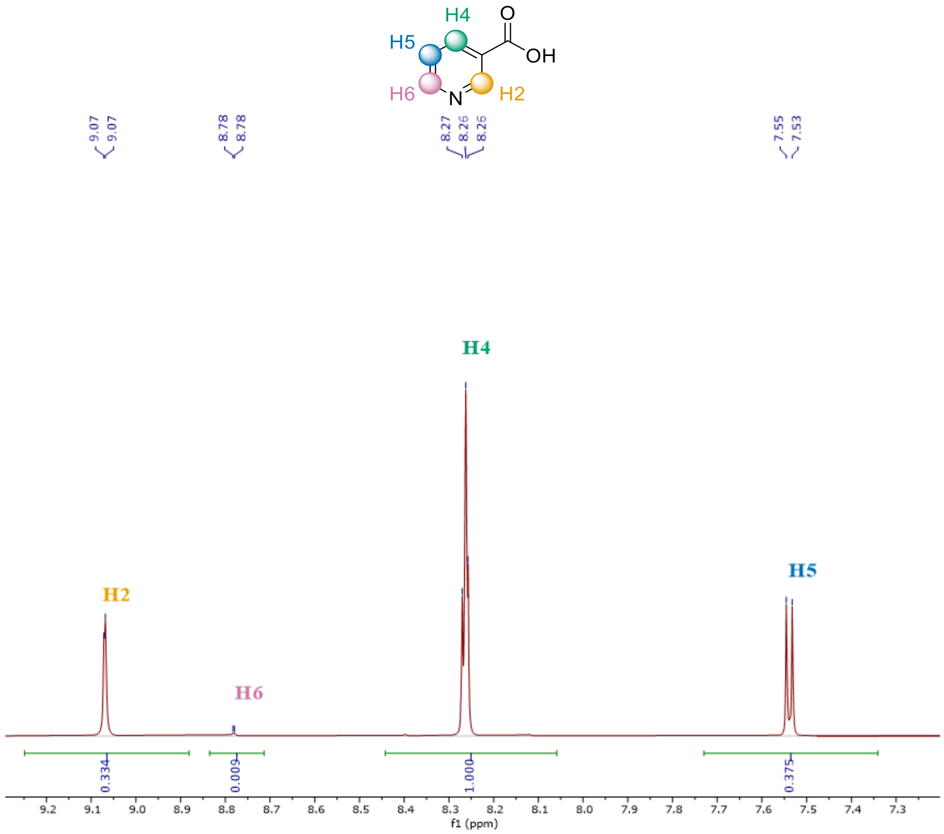

a. Set one of the integrals equal to 1 (this is done for convenience; however, it can be set to any number).

b. Obtain the sum of all the raw integrations (*Hx*) for deuterated positions: $\Sigma = H2 + H4 + H5 + H6$

c. Find the fraction each position makes up of the total sum: $\Phi_{H2} = \frac{H2}{\Sigma}$

    i. Φ_{H2}=0.194, Φ_{H4}=0.582, Φ_{H5}=0.218, Φ_{H6}=0.005

d. Obtain $\#D_{isopat}$ (total number of proton across the entire molecule) from the mass spectrum of the material. Loop injections were performed in triplicate as described above in section 1. Using these data, a modified version of Isopat[2] program was applied to obtain *#H* (total number of hydrogens in deuterated sample across the positions being considered). $\#H = \#H_{undeuterated\ sample} - \#D_{isopat} = 4 - 2.335D = 1.665H$

e. From the weight fraction (Φ_x), the absolute amount of hydrogen, *Hx'* (and by substraction, the amount deuterium) at position x can be solved using *#H*:

    i. $\Phi_{H2} = \frac{H2'}{1.665H} = 0.194 * 1.665H = H2' = 0.323H \therefore 67.6\%\ D\ at\ postion\ 2$

f. As a check, the amount deuterium was also recalculated from these corrected NMR values, S(1-Hx'), which should equal $\#D_{isopat}$.

Method 2.3 ^{13}C NMR

In some cases proton peaks could not be resolved entirely even with the use of various NMR solvents. As a result, $^{13}C\{^1H\}$ NMR was used to determine %D at a position in question and these values were cross-checked with ^{1}H NMR in various solvents. It is well established that ^{13}C signals

experience an upfield shift when deuterium is incorporated adjacent to the carbon in question (generally 1-2 carbons away). As more deuterium is incorporated, this upfield shift is increased.[15] With this knowledge in hand deuterium incorporation of a position adjacent to the carbon being examined can be determined as follows.

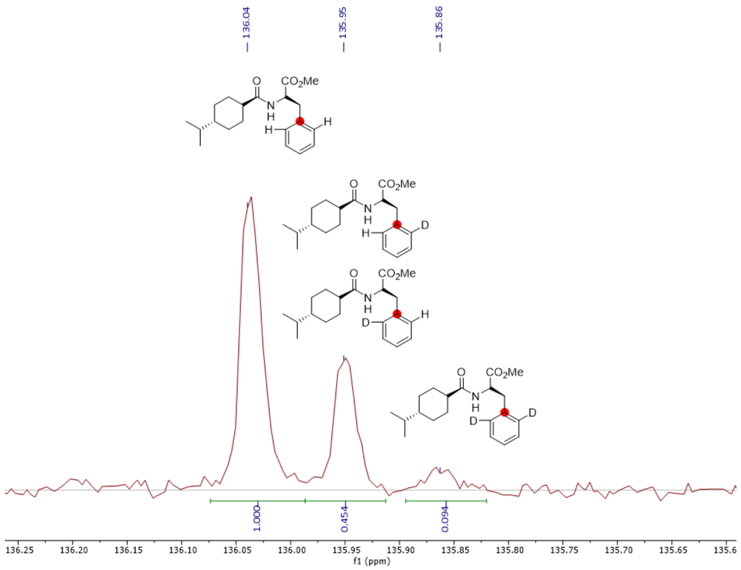

a. Integrate all peaks corresponding to a carbon adjacent to the deuterated position. Ideally this is a carbon that only experiences an isotopic shift from the position being examined.
b. The following equation can then be used to determine the %D at this position:[15]

$$\%D = 100 - \left[\frac{\sum(Area\ C\ \times \#H_{Cx})}{\sum(Area\ C\ \times \#H_{Cx}) + \sum(Area\ C\ \times \#D_{Cx})} \times 100\right]$$

Area C= the integration value for a single peak
$\#H_{Cx}$= the number of hydrogens at the adjacent carbon
$\#D_{Cx}$= the number of deuteriums at the adjacent carbon

c. For the above example we then have: 3.096

$$100 - \left[\frac{(1.000 \times 2) + (0.454 \times 1)}{(1.000 \times 2) + (0.454 \times 1) + (0.454 \times 1) + (0.094 \times 2)} \times 100\right] = \mathbf{20.7\%\ D}$$

d. This method is most accurate when adjacent positions have moderate levels of deuterium incorporation. Too much deuterium incorporation may make the first peak (adjacent to only H) unnoticeable, and conversely too little deuterium incorporation may make the last peak (adjacent to only D) unnoticeable.
e. If possible, this method should not be the only method used to determine deuterium incorporation and should be used to correct previously stated methods (2.1 or 2.2) when not all proton peaks can be resolved in ^{1}H NMR.

3. Reporting of ^{13}C Peaks in Deuterated Materials.

Peaks labelled as (m, *adjacent*) correspond to carbons which are not labeled with deuterium but experience an upfield chemical shift due to neighboring carbons (generally 1-2 bonds away) being labeled with deuterium. Peaks labeled as (m, *labeled*) correspond to carbons that have a

deuterium label, but exact coupling constants are not able to be determined (often due to low deuterium incorporation).

For partially deuterated materials, carbons bearing deuterium appear at different chemical shifts relative to carbons bearing protons. A combination of peaks was observed arising from a given position being deuterated (coupling to ^{13}C) and not deuterated (no coupling to ^{13}C). In addition, attachment of deuterium can alter chemical shifts up to two positions away. In some cases, overlapping signals could not be discerned leading to fewer signals than expected for a given structure.

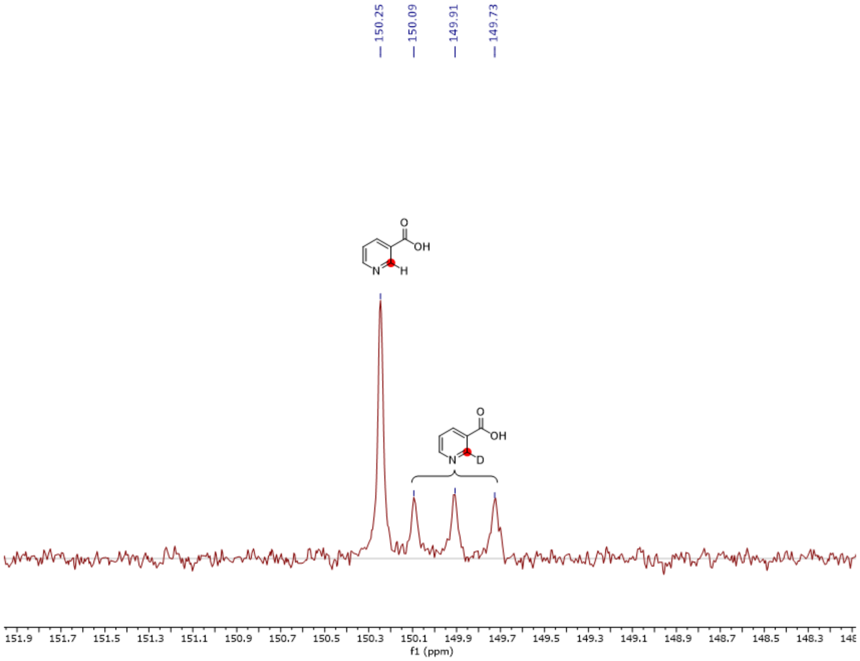

Fig. S1. Examples for ^{13}C spectra labelling. Isotope shift and triplet visible, denoted (*t*, 28.9 Hz).

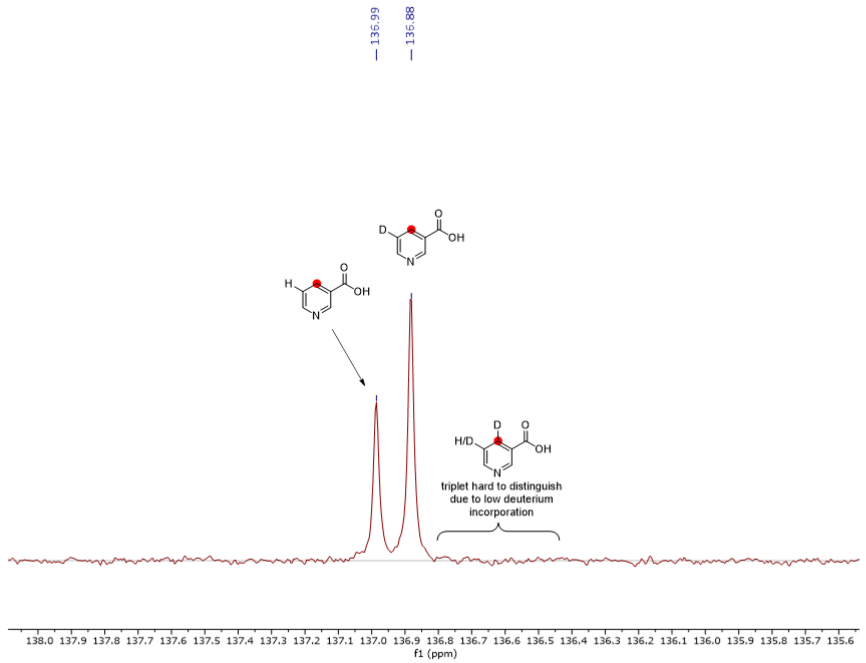

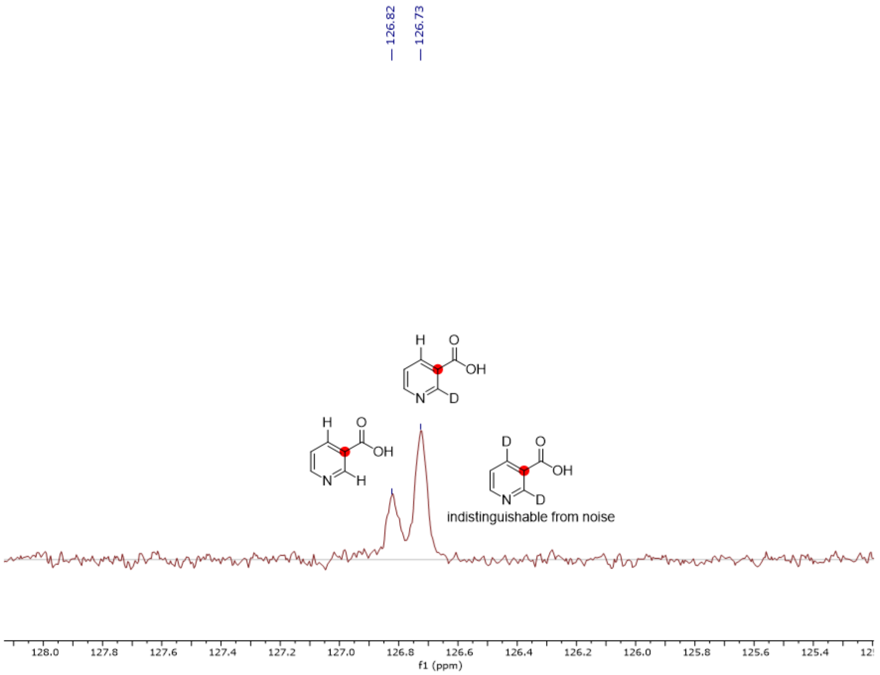

Fig. S2. Examples for ^{13}C spectra labelling. Isotope shift visible but triplet not seen, denoted (*m*, labeled) (top). Isotope shift visible but no deuterium incorporated at this position, denoted (*m*, adjacent) (bottom).

4. Synthesis of Deuterated Pyridines

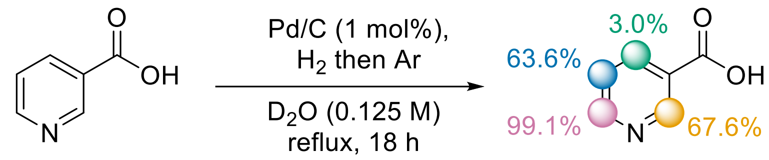

D-Nicotinic Acid ([D]1). A dry 1 L 2-neck flask was purged with argon and 10% Pd/C (0.804 g, 0.775 mmol) was added followed by D_2O (500 mL). Nicotinic acid (7.75 g, 62.9 mmol) was added to the flask and a reflux condenser stoppered with a septum was added and the side arm sealed with a rubber septum. An outlet needle was attached to the top of the condenser and through the side arm a long metal needle was used to bubble argon vigorously through the solution for 10 min. The outlet needle was removed followed by the argon needle. The palladium was treated with H2 to ensure complete reduction to Pd(0): a balloon filled with H_2 was added to the side arm using a long metal needle, an outlet needle was attached to the condenser and H_2 was bubbled through the solution for 10 min ensuring positive pressure. The outlet needle was removed followed by the H_2 needle and once again argon was bubbled through the solution for 10 min before changing to an argon inlet at the top of the condenser. Using a heating mantle the mixture was heated to a vigorous reflux. After 3 h, the reaction was cooled to rt and purged with H_2 followed by argon as was done previously. The mixture was again heated to a vigorous reflux and after 15 h (total reaction time of 18 h) the flask was removed from heat and allowed to cool. The mixture was filtered through Celite^{TM} and the filtrate separated to recover the D_2O. To ensure complete recovery of material the Celite was washed with DI water and this material was concentrated directly to yield pure product. The initial filtrate containing D_2O was distilled using a heating mantle to recover ~400 mL D_2O which can be used for subsequent deuterations. The residue in the distillation pot was also pure product. The two sets of isolated products were combined yielding [D]-nicotinic acid as a white powder (7.50 g, 60.9 mmol, 97%). Multiple replicates of this procedure were undertaken resulting in batches of product with slightly different deuterated levels which were determined as described below.

Protio Material:

^{1}H NMR (600 MHz, DMSO) δ 9.07 (dd, *J* = 2.2, 0.9 Hz, 1H), 8.78 (dd, *J* = 4.8, 1.7 Hz, 1H), 8.26 (ddd, *J* = 7.9, 2.2, 1.7 Hz, 1H), 7.54 (ddd, *J* = 7.9, 4.8, 0.9 Hz, 1H).

^{13}C{^{1}H} NMR (151 MHz, DMSO) δ 166.3, 153.3, 150.2, 137.0, 126.6, 123.8.

Spectral data are of commercial material and agree with those reported.[16]

Deutero Material:

^{1}H NMR (600 MHz, d1=30s, DMSO) δ 9.08 – 9.01 (m, 0.334H), 8.81 – 8.72 (m, 0.009H), 8.41 – 7.91 (m, 1.000H), 7.54 (d, J = 7.9 Hz, 0.375H). Integrations relative to largest peak (see method 2 above).

^{13}C{^{1}H} NMR (151 MHz, DMSO) δ 166.4, 153.0 – 152.7 (m, *labeled*), 150.2, 149.9 (t, 28.9 Hz), 137.0 – 136.9 (m, *labeled*), 126.8 – 126.7 (m, *adjacent*), 123.70, 123.68, 123.4 (t, 26.2 Hz).

MS (ESI, SQ): loop injection, concentration 100 μM, injection vol 1 μL, cone voltage 30 V.

Isopat: 2.334 D (NMR: 2.333 D).

%D Calculation: Method 2.2.

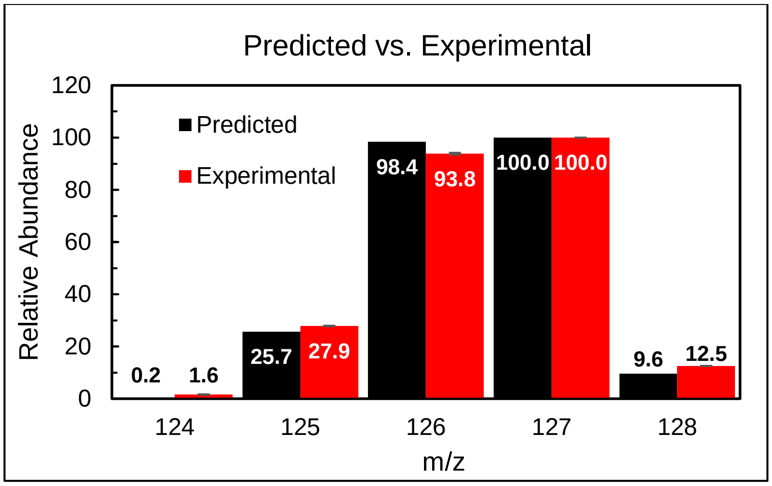

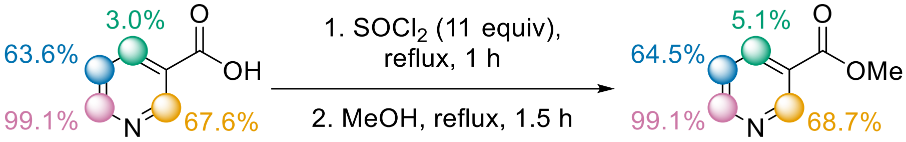

D-Methyl Nicotinate ([D]2). To a dry 25 mL Schlenk flask was added nicotinic acid (500 mg, 4.06 mmol). A reflux condenser was attached, and the flask was purged with argon and cooled to 0 °C using an ice bath before slowly adding thionyl chloride (6.2 mL, 85 mmol) over the course of ~5 min. The mixture was heated to reflux using an oil bath at 80 °C. After 1 h, the solution became clear and was cooled to rt. Excess thionyl chloride was removed under reduced pressure and the residual solids were dissolved in dry MeOH (10 mL). A reflux condenser was attached, and the flask purged with argon before heating to reflux with an oil bath at 70 °C. After 1.5 h the reaction was allowed to cool to rt and the volatiles removed under reduced pressure. The resulting solids were dissolved in CH_2Cl_2 (100 mL). The organic layer was washed with satd $NaHCO_3$ (3 x 5 mL), satd NaCl (10 mL), dried using anhydrous $MgSO_4$, filtered, and concentrated to yield pure **D-methyl nicotinate** (**[D]2**) as an off-white powder (0.522 g, 3.08 mmol, 76%). Deuteration levels were determined as described below.

Protio Material:

^{1}H NMR (600 MHz, $CDCl_3$) δ 9.19 (d, *J* = 1.8 Hz, 1H), 8.74 (dd, *J* = 4.9, 1.8 Hz, 1H), 8.26 (dt, *J* = 7.9, 2.0 Hz, 1H), 7.36 (dd, *J* = 7.9, 4.9 Hz, 1H), 3.92 (s, 3H).

^{13}C{^{1}H} NMR (151 MHz, $CDCl_3$) δ 165.8, 153.5, 151.0, 137.1, 126.1, 123.4, 52.5.

Spectral data are of commercial material and agree with those reported.[17]

Deutero Material:

^{1}H NMR (600 MHz, d1=30s, $CDCl_3$) δ 9.22 (s, 0.313H, *C2*), 8.77 (s, 0.009H, *C6*), 8.36 – 8.24 (m, 0.949H, *C4*), 7.39 (d, *J* = 7.9 Hz, 0.355H, *C5*), 3.95 (s, 3H).

$^{13}C\{^1H\}$ NMR (151 MHz, $CDCl_3$) δ 165.9, 153.15 (t, 27.4 Hz), 153.08 (t, 27.2 Hz), 151.0, 150.7 (t, 27.8 Hz), 137.2 – 137.1 (m, *adjacent*), 126.2 – 126.1 (m, *adjacent*), 123.3, 123.0 (t, 25.2 Hz), 52.6.

MS (ESI, SQ): loop injection, concentration 100 μM, injection vol 1 μL, cone voltage 30 V.

Isopat: 2.368 (NMR: 2.374 D).

%D Calculation: Method 2.1.

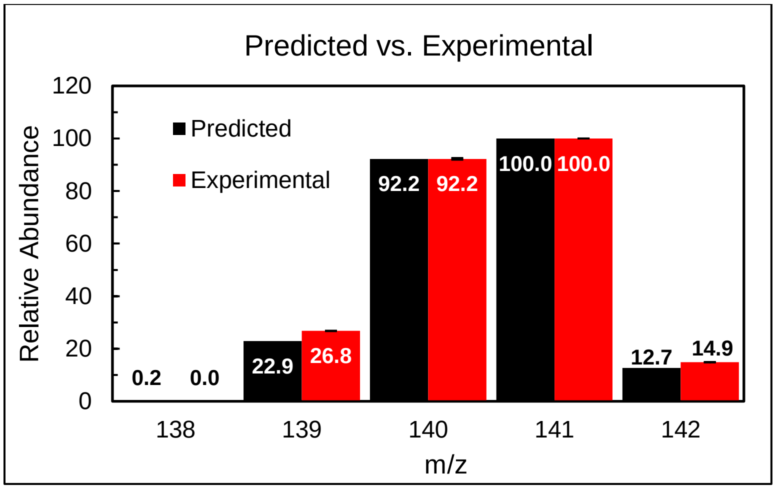

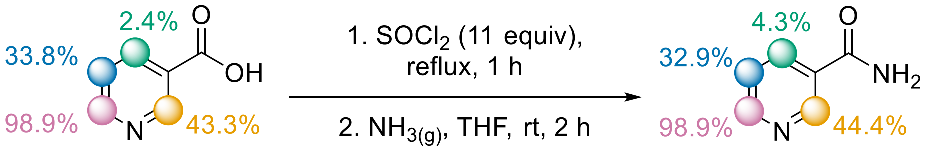

D-Nicotinamide ([D]6). To a dry 50 mL Schlenk flask was added nicotinic acid (1.00 g, 8.12 mmol). A reflux condenser was attached and sealed with a rubber septum prior to vacuum purging with argon 3x. The flask was cooled to 0 °C using an ice bath and thionyl chloride (12.5 mL, 171 mmol) was added slowly over the course of ~2 min. The mixture was heated to reflux using an oil bath at 80 °C. After 1 h, the solution became clear and was cooled to rt. The excess thionyl chloride was removed under reduced pressure. The remaining solids were dissolved in anhydrous THF (20 mL). NH_3 gas (generated from aqueous 35% NH_4OH by heating to 50 °C and passing the evolved gas through a CaO drying tube) was bubbled through the solution for 2 h. After 2 h, the volatiles were removed and the solids redissolved in $CHCl_3$ (200 mL). The organic layer was washed with satd $NaHCO_3$ (3 x 50 mL) followed by satd NaCl (50 mL). The organic layer was dried using anhydrous $MgSO_4$, filtered, and concentrated to yield pure **D-nicotinamide** (**[D]6**) as a beige solid (0.922 mg, 7.55 mmol, 93%).

Protio Material:

^1H NMR (600 MHz, MeOD) δ 9.02 (dd, *J* = 2.3, 0.9 Hz, 1H), 8.69 (dd, *J* = 4.9, 1.7 Hz, 1H), 8.29 (ddd, *J* = 8.0, 2.3, 1.6 Hz, 1H), 7.55 (ddd, *J* = 8.0, 4.9, 0.9 Hz, 1H).

$^{13}C\{^1H\}$ NMR (151 MHz, MeOD) δ 169.8, 152.8, 149.5, 137.3, 131.4, 125.1.

Spectral data are of commercial material and agree with those reported.[18]

Deutero Material:

^1H NMR (600 MHz, d1=30s, MeOD) δ 9.04 – 9.00 (m, 0.581H), 8.72 – 8.67 (m, 0.011H), 8.31 – 8.27 (m, 1.000H), 7.57 – 7.52 (m, 0.701H). Integrations relative to largest peak (see method 2 above).

$^{13}C\{^1H\}$ NMR (151 MHz, MeOD) δ 169.82 — 169.80 (m, *adjacent*), 152.5 (t, 27.4 Hz), 152.4 (t, 27.7 Hz), 149.5, 149.1 (t, 27.8 Hz), 137.3 – 137.2 (m, *labeled*), 131.4 – 131.3 (m, *adjacent*), 125.1, 124.98, 124.97, 124.7 (t, 26.4 Hz).

MS (ESI, SQ): loop injection, concentration 100 μM, injection vol 1 μL, cone voltage 30 V.

IsoPat: 1.806 D (NMR: 1.805 D).

%D Calculation: Method 2.2.

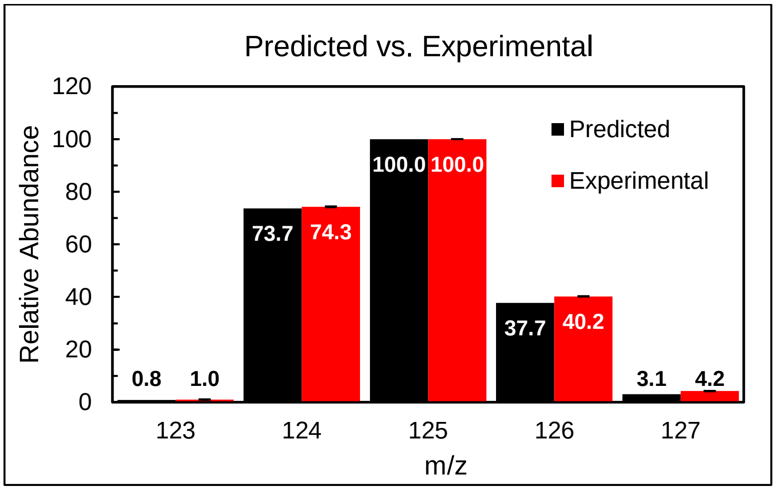

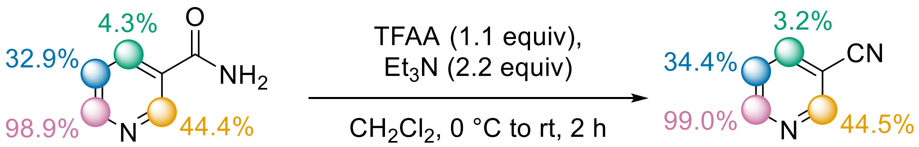

D-3-Cyanopyridine ([D]3). To a dry 100 mL rbf was added nicotinamide (0.500 g, 4.09 mmol) followed by dry CH_2Cl_2 (20 mL). The flask was cooled to 0 °C with an ice bath and purged with argon. Freshly distilled triethylamine (1.26 mL, 9.01 mmol, distilled from CaH_2) was added to the flask followed by the dropwise addition of trifluoroacetic anhydride (0.64 mL, 4.5 mmol, TFAA) over the course of ~1 min. The mixture was allowed to warm to rt and after 2 h DI water (50 mL) was added. The organic phase was separated and the aqueous layer washed with CH_2Cl_2 (3 x 20 mL). The organic phases were combined, washed with satd NaCl (10 mL), dried with anhydrous $MgSO_4$, filtered, and concentrated. The resultant material was chromatographed using 10-20% EtOAc in hexanes to yield pure **D-3-cyanopyridine** (**[D]3**) as an off-white amorphous solid (0.222 g, 2.14 mmol, 52%).

Protio Material:

^{1}H NMR (600 MHz, CDCl$_3$) δ 8.92 – 8.88 (m, 1H), 8.82 (dt, *J* = 4.9, 1.6, 1.6 Hz, 1H), 8.00 – 7.94 (m, 1H), 7.44 (dd, *J* = 8.0, 4.9 Hz, 1H).

^{13}C{^{1}H} NMR (151 MHz, CDCl$_3$) δ 153.1, 152.6, 139.4, 123.8, 116.6, 110.3.

Spectral data are of commercial material and agree with those reported.[19]

Deutero Material:

^{1}H NMR (600 MHz, d1=30s, CDCl$_3$) δ 8.93 – 8.89 (m, 0.573H), 8.85 – 8.82 (m, 0.010H), 8.01 – 7.96 (m, 1.000H), 7.45 (d, *J* = 7.8 Hz, 0.678H). Integrations relative to largest peak (see method 2 above).

^{13}C{^{1}H} NMR (151 MHz, CDCl$_3$) δ 152.6, 152.5, 152.25 (t, 28.0 Hz), 152.18 (t, 28.1 Hz), 152.0, 151.7 (t, 28.4 Hz), 139.2 – 138.7 (m, *labeled*), 123.4, 123.2, 123.0 (t, 25.9 Hz), 116.2, 109.7 – 109.4 (m, *adjacent*).

MS (ESI, SQ): loop injection, concentration 100 μM, injection vol 1 μL, cone voltage 30 V.

IsoPat: 1.811 D (NMR: 1.811 D).

%D Calculation: Method 2.2.

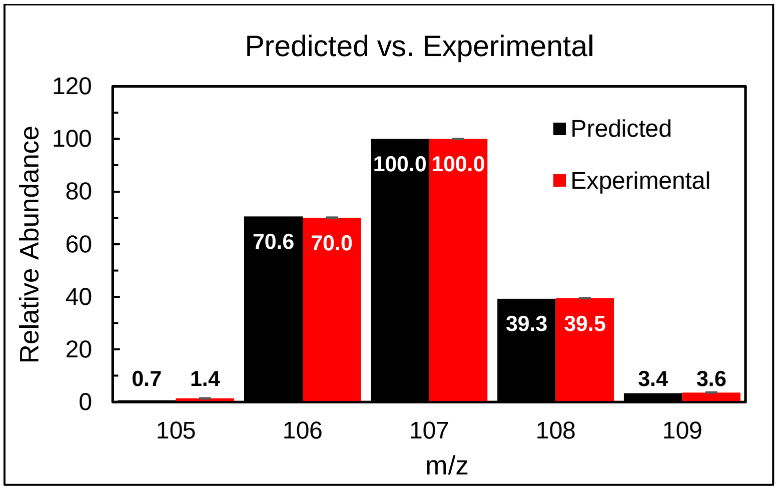

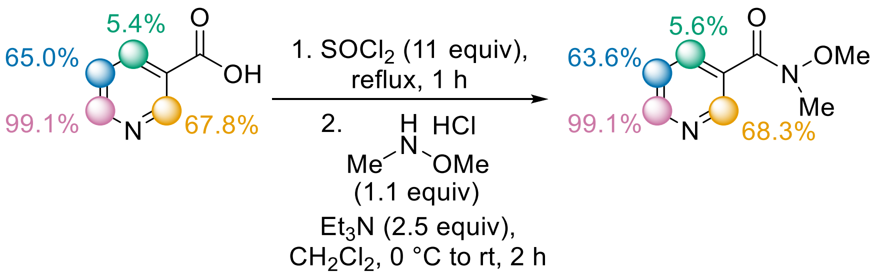

D-*N*-Methoxy-*N*-methylnicotinamide. To a dry 50 mL Schlenk flask was added nicotinic acid (2.50 g, 20.3 mmol). A reflux condenser was attached, and the flask was purged with argon and cooled to 0 °C using an ice bath before slowly adding thionyl chloride (17 mL, 230 mmol) over the course of ~5 min. The mixture was heated to reflux using an oil bath at 80 °C and after 1 h of heating the solution became clear and was cooled to rt. Excess thionyl chloride was removed under reduced pressure and the residual solids were dissolved in dry CH_2Cl_2 (16 mL). *N,N*-dimethylhydroxylamine hydrochloride (2.18 g, 22.3 mmol) was added and the flask was purged with argon and cooled to 0 °C using an ice bath. Freshly distilled triethylamine (7.1 mL, 51 mmol) was added to the stirring solution over the course of ~2 min. After 1 h of stirring, the flask was removed from the ice bath and left to warm to rt over the course of 1 h. DI water (25 mL) was added to the flask and the organic layer separated. The remaining aqueous layer was washed with CH_2Cl_2 (3 x 15 mL), the organic phases were combined and washed with satd $NaHCO_3$ (10 mL) followed by satd NaCl (10 mL), dried with anhydrous $MgSO_4$, filtered, and concentrated. The resultant material was chromatographed using 20-60% EtOAc in hexanes to yield pure **D-*N*-methoxy-*N*-methylnicotinamide** as a brown oil (2.52 g, 15.2 mmol, 75%).

Protio Material:

^{1}H NMR (600 MHz, $CDCl_3$) δ 8.96 (s, 1H), 8.69 (d, *J* = 4.9 Hz, 1H), 8.03 (dq, *J* = 7.9, 1.8 Hz, 1H), 7.37 (dd, *J* = 7.8, 4.9 Hz, 1H), 3.56 (s, 3H), 3.40 (s, 3H).

^{13}C{^{1}H} NMR (151 MHz, $CDCl_3$) δ 167.4, 151.4, 149.3, 136.1, 129.9, 123.0, 61.3, 33.2.

The protio compound was generated using the same protocol described above for the deutero material. The spectral data agree with those reported.[20]

Deutero Material:

^{1}H NMR (600 MHz, d1=30s, $CDCl_3$) δ 8.94 (s, 0.317H), 8.67 (s, 0.009H), 8.02 (td, *J* = 3.7, 3.4, 1.0 Hz, 0.944H), 7.35 (d, *J* = 7.9 Hz, 0.364H), 3.54 (s, 3H), 3.38 (s, 3H).

^{13}C{^{1}H} NMR (151 MHz, $CDCl_3$) δ 167.48 – 167.46 (m, *adjacent*), 151.06 (t, 27.6 Hz), 151.00 (t, 27.5 Hz), 149.3, 149.0 (t, 28.0 Hz), 136.3 – 136.1 (m, *adjacent*), 130.0 – 129.8 (m, *adjacent*), 122.97 – 122.94 (m, *adjacent*), 122.7 (t, 25.2 Hz), 61.4, 33.3.

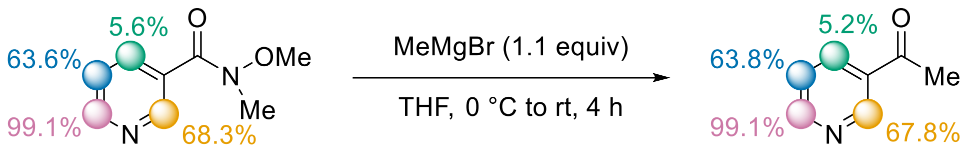

D-3-Acetylpyridine ([D]4). To a dry 25 mL Schlenk flask was added **D-*N*-methoxy-*N*-methylnicotinamide** (0.500 g, 3.01 mmol). The flask was purged with argon and dry THF (10 mL) was added. The flask was cooled to 0 °C with an ice bath, and MeMgBr (3.0 M in Et_2O, 1.2 mL, 3.6 mmol) was added over the course of ~1 min. The stirring solution was removed from the ice bath and allowed to warm to rt. After 4 h, the reaction was deemed complete by TLC and satd NH_4Cl (15 mL) was added slowly over the course of ~1 min. After stirring for 15 min, the mixture was extracted with CH_2Cl_2 (3 x 10 mL). The organic washings were combined, washed with satd NaCl (10 mL), dried with anhydrous $MgSO_4$, filtered, and concentrated. The resultant material was chromatographed using 5-20% EtOAc in hexanes to yield pure **D-3-acetylpyridine** (**[D]4**) as a pale yellow oil (0.328 g, 2.71 mmol, 90%).

Protio Material:

^{1}H NMR (600 MHz, $CDCl_3$) δ 9.14 (d, *J* = 1.1 Hz, 1H), 8.76 (dd, *J* = 4.8, 1.2 Hz, 1H), 8.21 (dt, *J* = 8.0, 1.8 Hz, 1H), 7.40 (dd, *J* = 7.7, 4.8 Hz, 1H), 2.62 (s, 3H).

^{13}C{^{1}H} NMR (151 MHz, $CDCl_3$) δ 196.8, 153.7, 150.1, 135.5, 132.4, 123.7, 26.8.

Spectral data are of commercial material and agree with those reported.[21]

Deutero Material:

^{1}H NMR (600 MHz, d1=30s, $CDCl_3$) δ 9.17 (s, 0.322H), 8.78 (s, 0.009H), 8.23 (s, 0.948H), 7.42 (d, *J* = 7.9 Hz, 0.362H), 2.64 (s, 3H).

^{13}C{^{1}H} NMR (151 MHz, $CDCl_3$) δ 196.8, 153.32 (t, 27.4 Hz), 153.26 (t, 27.4 Hz), 150.1, 149.7 (t, 27.2 Hz), 135.56 – 135.45 (m, *labeled*), 132.4 – 132.3 (m, *adjacent*), 123.6, 123.3 (t, 25.6 Hz), 26.8.

MS (ESI, SQ): loop injection, concentration 100 μM, injection vol 1 μL, cone voltage 30 V.

Isopat: 2.370 D (NMR: 2.359 D).

%D Calculation: Method 2.1.

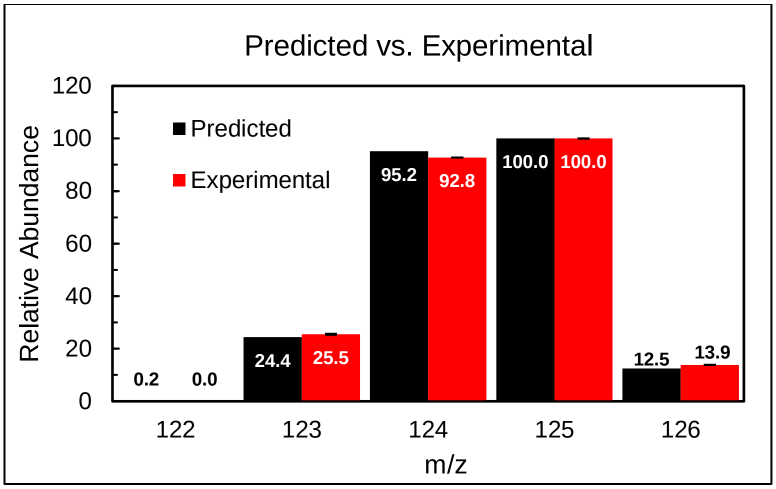

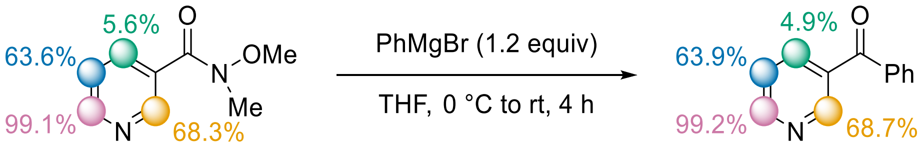

D-3-Benzoylpyridine ([D]5). To a dry 25 mL Schlenk flask was added **D-*N*-methoxy-*N*-methylnicotinamide** (0.500 g, 3.01 mmol). The flask was purged with argon and dry THF (10 mL) was added. The flask was cooled to 0 °C with an ice bath, and PhMgBr (1.6 M in CPME, 2.3 mL, 3.7 mmol) was added over the course of ~1 min. The stirring solution was removed from the ice bath and allowed to warm to rt. After 4 h, the reaction was deemed complete by TLC and satd NH_4Cl (15 mL) was added slowly over the course of ~1 min. After stirring for 15 min, the mixture was extracted with CH_2Cl_2 (3 x 10 mL). The organic washings were combined, washed with satd NaCl (10 mL), dried with anhydrous $MgSO_4$, filtered, and concentrated. The resultant material was chromatographed using 5-20% EtOAc in hexanes to yield pure **D-3-benzoylpyridine** (**[D]5**) as an off-white solid (0.424 g, 2.31 mmol, 77%).

Protio Material:

^{1}H NMR (600 MHz, $CDCl_3$) δ 8.98 (d, *J* = 1.3 Hz, 1H), 8.80 (dd, *J* = 4.8, 1.8 Hz, 1H), 8.10 (dddd, *J* = 7.8, 2.2, 1.7, 0.5 Hz, 1H), 7.86 – 7.75 (m, 2H), 7.68 – 7.57 (m, 1H), 7.55 – 7.48 (m, 2H), 7.44 (dddd, *J* = 7.9, 4.9, 0.9, 0.4 Hz, 1H).

^{13}C{^{1}H} NMR (151 MHz, $CDCl_3$) δ 195.0, 153.0, 151.1, 137.3, 136.85, 136.84, 133.3, 130.2, 128.8, 123.5.

Spectral data are of commercial material and agree with those reported.[22]

Deutero Material:

^{1}H NMR (600 MHz, d1=30s, $CDCl_3$) δ 9.00 (d, *J* = 2.2 Hz, 0.313H), 8.82 (s, 0.008H), 8.18 – 8.09 (m, 0.951H), 7.87 – 7.78 (m, 2H), 7.69 – 7.60 (m, 1H), 7.58 – 7.47 (m, 2H), 7.48 – 7.44 (m, 0.357H).

$^{13}C\{^1H\}$ NMR (151 MHz, $CDCl_3$) δ 195.03, 195.01, 152.63 (t, 27.4 Hz), 152.57 (t, 27.4 Hz), 151.1, 150.1 (t, 27.6 Hz), 137.3, 137.2, 136.9, 133.33, 133.31, 133.2, 130.2, 128.8, 123.38, 123.36, 123.1 (t, 25.7 Hz).

MS (ESI, SQ): loop injection, concentration 100 μM, injection vol 1 μL, cone voltage 30 V.

Isopat: 2.353 D (NMR: 2.367).

%D Calculation: Method 2.1.

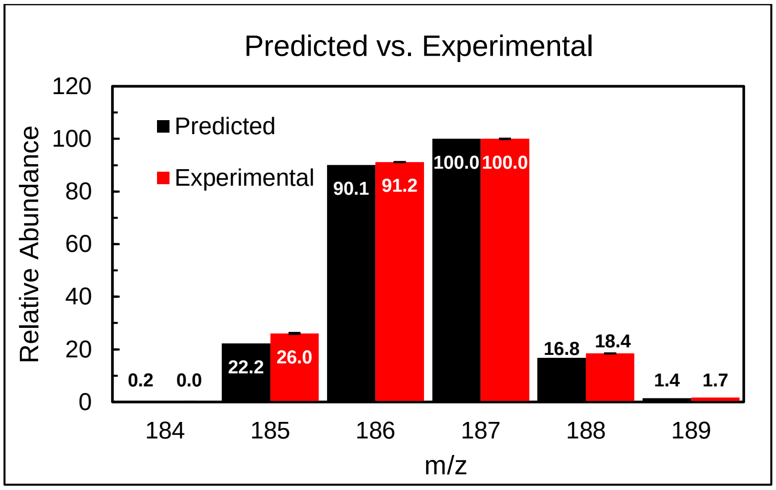

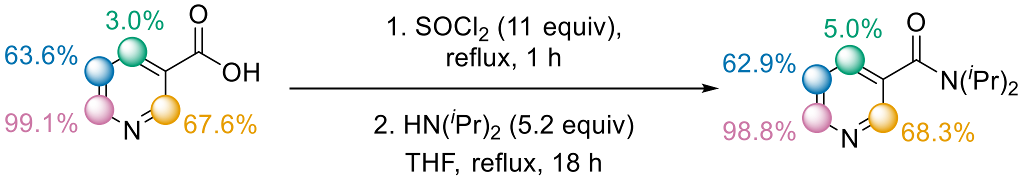

D-*N,N*-diisopropylnicotinamide ([D]7). To a dry 25 mL Schlenk flask was added nicotinic acid (0.500 g, 4.06 mmol). A reflux condenser was attached and the flask was purged with argon and cooled to 0 °C using an ice bath before slowly adding thionyl chloride (6.2 mL, 85 mmol) over the course of ~5 min. The mixture was heated to reflux using an oil bath and after 1 h of heating the solution became clear and was cooled to rt. Excess thionyl chloride was removed under reduced pressure and the residual solids were dissolved in dry THF (10 mL) and a reflux condenser was attached. The flask was purged with argon and freshly distilled diisopropylamine (2.9 mL, 21 mmol) was added. The flask was heated to reflux using an oil bath. After 18 h the mixture was cooled to rt, poured into DI water (100 mL), and the aqueous mixture was extracted with CH_2Cl_2 (3 x 25 mL). The organic washings were combined, washed with satd NaCl (15 mL), dried using anhydrous $MgSO_4$, filtered, and concentrated. The resultant material was purified via column chromatography using 5-20% EtOAc in hexanes to yield pure **D-*N,N*-diisopropylnicotinamide** (**[D]7**) as a tan solid (0.563 g, 2.73 mmol, 67%).

Protio Material:

^1H NMR (600 MHz, $CDCl_3$) δ 8.61 (d, J = 4.8 Hz, 1H), 8.62 – 8.56 (m, 1H), 7.67 – 7.62 (m, 1H), 7.32 (dd, J = 7.8, 4.9 Hz, 1H), 4.08 – 3.31 (m, 2H), 2.00 – 0.76 (m, 12H).

$^{13}C\{^1H\}$ NMR (151 MHz, $CDCl_3$) δ 168.4, 150.0, 146.8, 134.7, 133.7, 123.6, 51.3, 46.4, 20.9.

The protio compound was generated using the same protocol described above for the deutero material. The spectral data agree with those reported.[23]

Deutero Material:

^1H NMR (600 MHz, d1=30s, $CDCl_3$) δ 8.62 (s, 0.013H), 8.60 – 8.57 (m, 0.334H), 7.68 – 7.63 (m, 1.000H), 7.33 (d, *J* = 7.7 Hz, 0.391H), 3.95 – 3.41 (m, 2H), 1.68 – 0.93 (m, 12H).

$^{13}C\{^1H\}$ NMR (151 MHz, $CDCl_3$) δ 168.4, 149.7 (t, 28.2 Hz), 149.6 (t, 28.0 Hz), 146.8, 146.5 (t, 27.4 Hz), 134.7 – 134.5 (m, *adjacent*), 133.7 – 133.6 (m, *labeled*), 123.48, 123.47, 123.2 (t, 25.6 Hz), 51.3, 46.3, 20.9.

MS (ESI, SQ): loop injection, concentration 100 µM, injection vol 1 µL, cone voltage 30 V.

Isopat: 2.350 D (NMR: 2.350 D).

%D Calculation: Method 2.2.

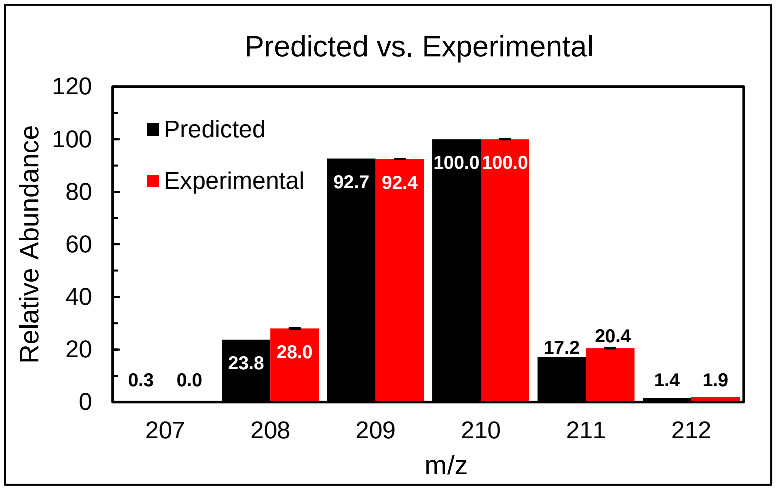

5. Reactions of Pyridines

5.1 Proof of Concept Minisci Reaction of [H]2 and [D]2 with *para*-Tolylboronic Acid

5.1.1 Reaction Conditions and Compound Characterization

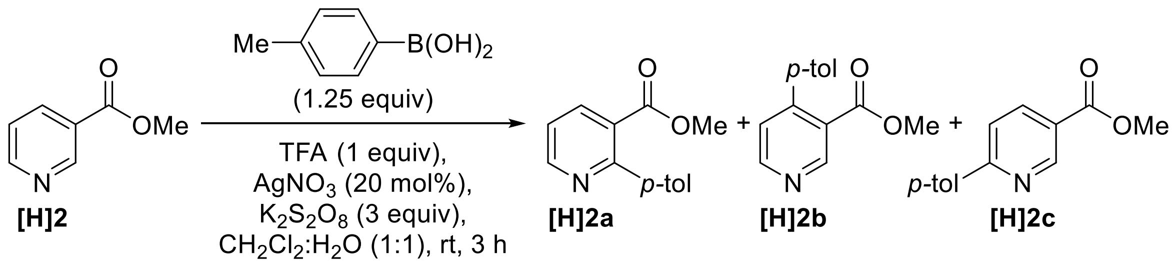

Reaction of Methyl Nicotinate with *para*-Tolylboronic Acid Using $K_2S_2O_8$. To a solution of methyl nicotinate (1.00 g, 7.29 mmol) in 42.5 mL CH_2Cl_2 was added trifluoroacetic acid (0.56 mL, 7.3 mmol, 1.0 equiv). The solution was stirred in a 300 mL rbf under air. Then, *para*-tolylboronic acid (1.24 mg, 9.12 mmol, 1.25 equiv) was added, followed by water (42.5 mL), solid $AgNO_3$ (249 mg, 20 mol %) and finally $K_2S_2O_8$ (5.91 g, 21.9 mmol, 3.00 equiv) over 1 min. After stirring for 3 h at rt, the reaction mixture was quenched with 2 M NaOH (150 mL) and further diluted with CH_2Cl_2 (90 mL). The aqueous layer was extracted with CH_2Cl_2 (3 x 75 mL). The combined organic layers were dried over Na_2SO_4, filtered and concentrated. The resultant material was chromatographed using 20-40% EtOAc in hexanes to affording **[H]2a** as a yellow oil (277 mg, 1.22 mmol, 17%), **[H]2b** as a yellow oil (440 mg, 1.94 mmol, 27%), and **[H]2c** as a white amorphous solid (396 mg, 1.74 mmol, 24%).

Methyl 2-(*para*-tolyl)nicotinate ([H]2a)
^{1}H NMR (600 MHz, $CDCl_3$) δ 8.78 – 8.74 (m, 1H), 8.06 (d, *J* = 7.8 Hz, 1H), 7.45 (d, *J* = 7.6 Hz, 2H), 7.29 (t, *J* = 6.3 Hz, 1H), 7.24 (d, *J* = 7.8 Hz, 2H), 3.72 (s, 3H), 2.40 (s, 3H).

^{13}C{^{1}H} NMR (101 MHz, $CDCl_3$) δ 168.9, 158.9, 151.4, 138.8, 137.9, 137.2, 129.1, 128.6, 127.0, 121.4, 52.5, 21.5.

Methyl 4-(*para*-tolyl)nicotinate ([H]2b)
^{1}H NMR (600 MHz, $CDCl_3$) δ 8.99 (s, 1H), 8.69 (d, *J* = 5.1 Hz, 1H), 7.29 (d, *J* = 5.2 Hz, 1H), 7.26 – 7.20 (m, 4H), 3.73 (s, 3H), 2.40 (s, 3H).

^{13}C{^{1}H} NMR (151 MHz, $CDCl_3$) δ 167.5, 152.0, 150.9, 150.2, 138.7, 135.5, 129.3, 128.0, 126.5, 125.0, 52.4, 21.4.

Methyl 6-(*para*-tolyl)nicotinate ([H]2c)
^{1}H NMR (400 MHz, $CDCl_3$) δ 9.26 (dd, *J* = 2.2, 0.9 Hz, 1H), 8.32 (dd, *J* = 8.3, 2.2 Hz, 1H), 7.97 (d, *J* = 8.2 Hz, 2H), 7.79 (dd, *J* = 8.3, 0.9 Hz, 1H), 7.31 (d, *J* = 8.4 Hz, 2H), 3.97 (s, 3H), 2.42 (s, 3H).

^{13}C{^{1}H} NMR (151 MHz, $CDCl_3$) δ 165.9, 160.8, 150.9, 140.2, 137.8, 135.4, 129.6, 127.2, 123.8, 119.4, 52.3, 21.3.

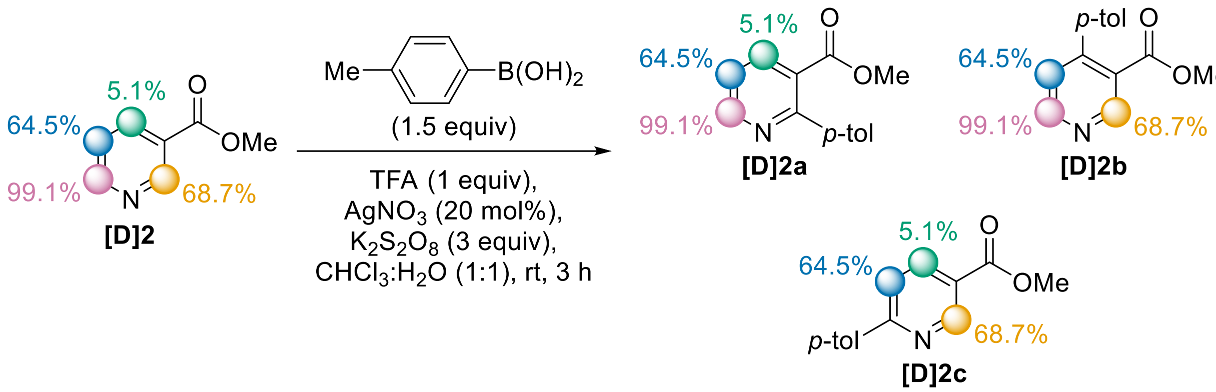

Reaction of [D]2 with *para*-Tolylboronic acid Using $K_2S_2O_8$. To a solution of **[D]2** (137 mg, 1.00 mmol) in 1:1 H_2O:$CHCl_3$ (20 mL) was added trifluoroacetic acid (77 µL, 1.00 mmol, 1.00 equiv). The solution was stirred in a 100 mL rbf under air. Then, *para*-tolylboronic acid (204 mg, 1.50 mmol, 1.50 equiv) was added, followed by silver nitrate (34 mg, 0.20 mmol, 20 mol %) and finally $K_2S_2O_8$ (811 mg, 3.00 mmol, 3.00 equiv). After stirring for 3 h at rt, the reaction mixture was quenched with satd $NaHCO_3$ (20 mL) and the aqueous layer was extracted with $CHCl_3$ (3 x 20 mL). The combined organic layers were dried over Na_2SO_4, filtered, and concentrated. The resultant crude material was purified via column chromatography using 20-40% EtOAc in hexanes to afforded labeled products: **[D]2a** (25 mg, 0.11 mmol, 11%) as a yellow oil, **[D]2b** (54 mg, 0.24 mmol, 24%) as a yellow oil, and **[D]2c** (36 mg, 0.16 mmol, 16%) as a white amorphous solid. The unpurified reaction mixture of labeled regioisomers was also analyzed directly by UPLC-MS, using selected ion monitoring (SIM)[24] across five *m/z* channels corresponding to each monoisotopic mass. Injection volumes of 1 µL of a 100 µM solution were preferred for UPLC-MS analysis whereas injection volumes 0.1/0.2 µL of a 100 µM solution were preferred for MS-only analysis. Less ionizable analytes may require sample concentrations of 1 mM.

[D]2a:
^{1}H NMR (600 MHz, $CDCl_3$) δ 8.78 (s, 0.008H), 8.12 – 8.07 (m, 0.943H), 7.46 (d, *J* = 8.1 Hz, 2H), 7.33 (d, *J* = 7.8 Hz, 0.496H), 7.25 (d, *J* = 7.7 Hz, 2H), 3.73 (s, 3H), 2.41 (s, 3H).

^{13}C{^{1}H} NMR (151 MHz, $CDCl_3$) δ 168.9, 158.8, 151.1 – 150.7, (m, labeled), 138.9, 138.1 – 138.0 (m, *labeled*), 137.1, 129.1, 128.6, 127.0, 121.3, 121.0 (t, 25.5 Hz), 52.5, 21.5.

[D]2b:
^{1}H NMR (600 MHz, $CDCl_3$) δ 9.01 (s, 0.327H), 8.71 (s, 0.008H), 7.33 (s, 0.402H), 7.28 – 7.19 (m, 4H), 3.74 (s, 3H), 2.41 (s, 3H).

^{13}C{^{1}H} NMR (151 MHz, $CDCl_3$) δ 167.54 – 167.52 (m, *adjacent*), 151.8 – 151.3 (m, *labeled*), 150.8 – 150.5 (m, *labeled*), 150.4 – 150.3 (m, *adjacent*), 138.8, 135.53 – 135.49 (m, *adjacent*), 129.3, 128.1, 126.6 – 126.4 (m, *adjacent*), 125.0, 124.8–124.5 (m, *labeled*), 52.4, 21.4.

[D]2c:
^{1}H NMR (600 MHz, $CDCl_3$) δ 9.27 (d, *J* = 2.2 Hz, 0.310H), 8.35 (t, *J* = 3.8 Hz, 0.903H), 7.98 (d, *J* = 8.2 Hz, 2H), 7.81 (d, *J* = 8.2 Hz, 0.334H), 7.32 (d, *J* = 7.9 Hz, 2H), 3.97 (s, 3H), 2.43 (s, 3H).

$^{13}C\{^1H\}$ NMR (151 MHz, $CDCl_3$) δ 166.1, 161.1 – 161.0 (m, *adjacent*), 151.1, 150.7 (t, 27.5 Hz), 140.4, 138.0 – 137.9 (m, *labeled*), 135.62 – 135.60 (m, *adjacent*), 129.8, 127.4, 124.0 – 123.9 (m, *adjacent*), 119.65, 119.63, 119.4 (t, 25.7 Hz), 52.5, 21.5.

Due to exchange during work up or column chromatography, the deuterium incorporation of the isolated regioisomers may not agree exactly with expected values from starting materials (typically <1% variance in these cases).

5.1.2 UPLC-MS Analysis and Identification of Regioisomers from MS Isotope Patterns

The unpurified reaction mixture of labeled regioisomers was analyzed directly by UPLC-MS in triplicate, using selected ion monitoring (SIM)[24] across five *m/z* channels corresponding to each monoisotopic mass. Injection volumes of 1 µL of a 100 µM solution were preferred for UPLC-MS analysis whereas injection volumes 0.1/0.2 µL of a 100 µM solution were preferred for MS-only analysis. Less ionizable analytes may require sample concentrations of 1 mM.

Generally, intensities obtained from narrow mass ranges (10-20 Da) were found to be comparable to those obtained from SIM. As such, narrow mass ranges were preferred for all other reported quantitative MS results due to operational ease.

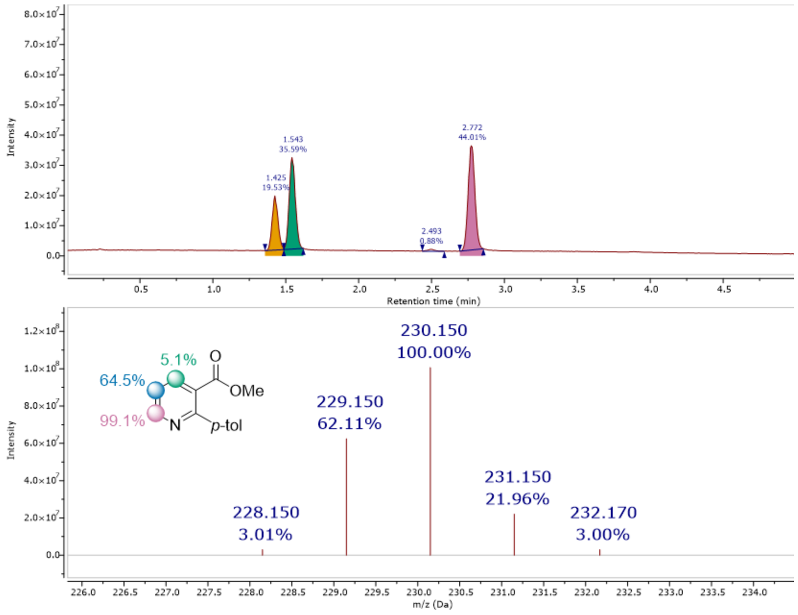

[D]2a	M+1	M+2	M+3	M+4	M+5
Exp.	8.5 ± 4.0	60.5±1.2	100 ± 0	21.8 ±0.6	3.0 ± 0.1
Pred.	0.4	50.1	100.0	19.9	2.0

Fig. S3. (top) Isotopic distribution observed for **[D]2a**. Direct analysis of the unpurified reaction mixture via UPLC-MS with SIM as described above. Full crosshair selection from t_R 1.36 – 1.48 min. (bottom) Summary of predicted (Pred.) vs. experimental (Exp.) isotopic distributions for, **[D]2a**. Experimental values are shown as the average of triplicate UPLC/MS runs of the same sample. Contamination in M+1 (228 m/z) results in a larger abundance than expected and increased error.

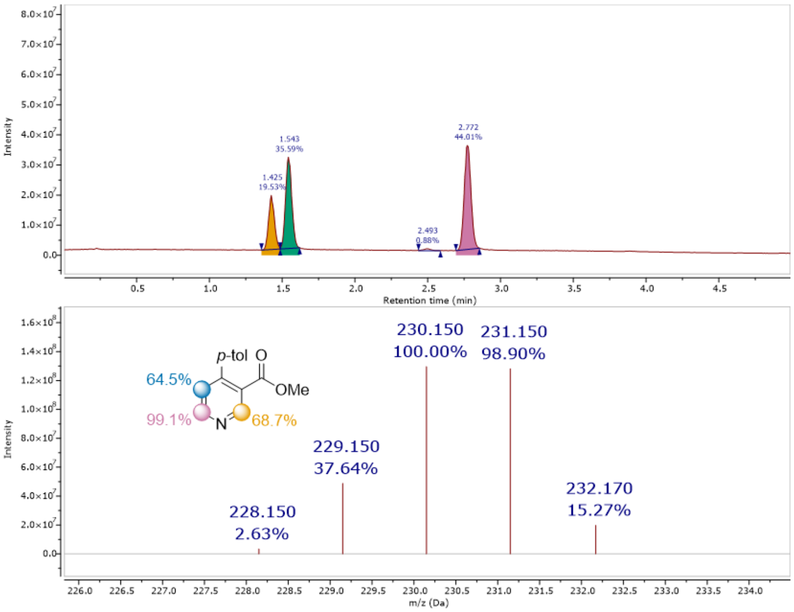

[D]2b	M+1	M+2	M+3	M+4	M+5
Exp.	7.6 ± 3.8	34.8 ± 1.9	98.0 ± 1.6	99.8 ±0.3	15.5 ± 0.1
Pred.	0.20	22.38	90.70	100.00	14.73

Fig. S4. (top) Isotopic distribution observed for **[D]2b**. Direct analysis of the unpurified reaction mixture via UPLC-MS with SIM as described above. Full crosshair selection from t_R 1.49 – 1.62 min. (bottom) Summary of predicted (Pred.) vs. experimental (Exp.) isotopic distributions for, **[D]2b**. Experimental values are shown as the average of triplicate UPLC/MS runs of the same sample. Contamination in M+1 (228 m/z) results in a larger abundance than expected and increased error.

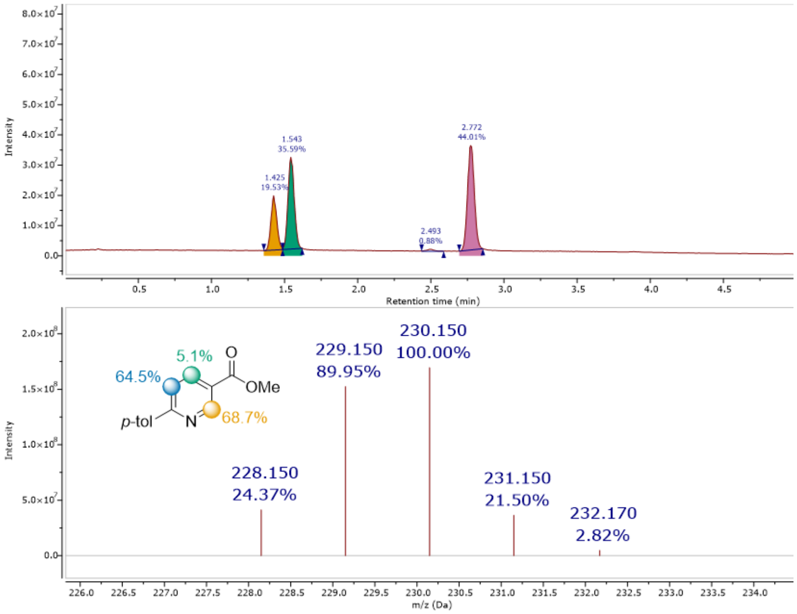

[D]2c	M+1	M+2	M+3	M+4	M+5

**Exp.**	30.7 ± 4.7	88.7 ± 0.9	100 ± 0	21.15 ± 0.2	2.7 ± 0.02
**Pred.**	20.62	86.97	100.00	19.16	1.88

Fig. S5. (top) Isotopic distribution observed for **[D]2c**. Direct analysis of the unpurified reaction mixture via UPLC-MS with SIM as described above. Full crosshair selection from t_R 2.69 – 2.85 min. (bottom) Table summarizing Predicted (Pred.) vs. Experimental (Exp.) isotopic distributions for, **[D]2c**. Experimental values are shown as the average of triplicate UPLC/MS runs of the same sample. Contamination in M+1 (228 m/z) results in a larger abundance than expected and increased error.

5.1.3 Quantitation of Regioisomers from MS-Only Analysis

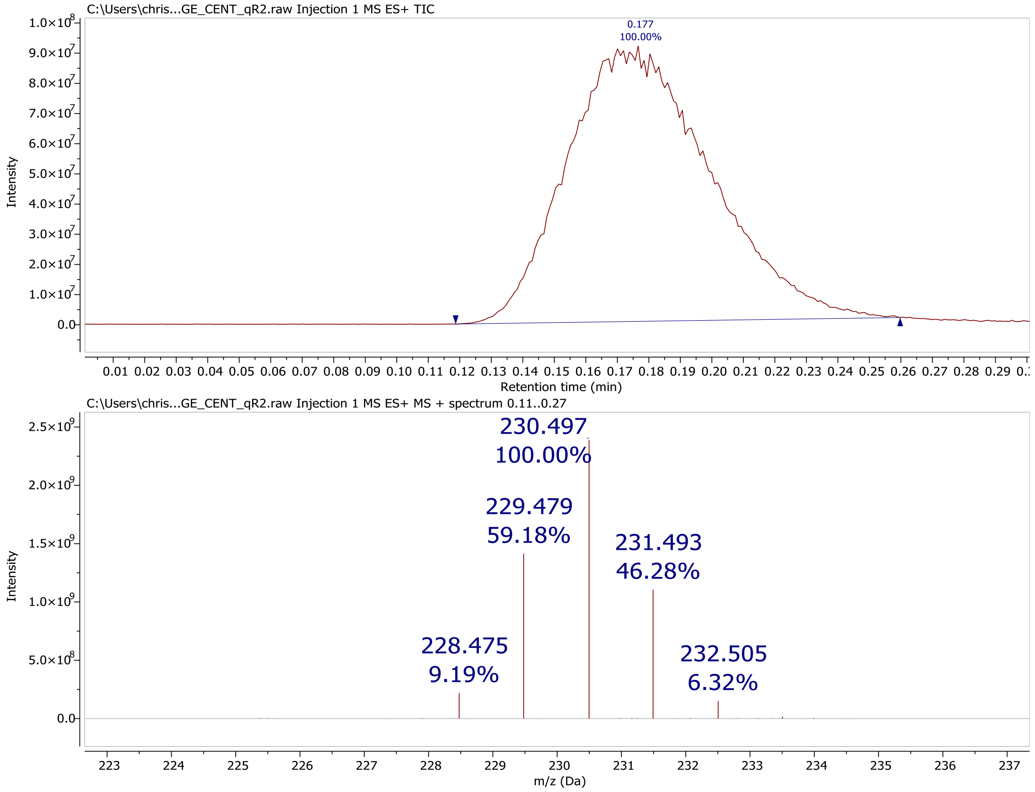

Trial	M+1	M+2	M+3	M+4	M+5
t1	9.19	59.07	100.00	46.47	6.31
t2	9.19	59.18	100.00	46.28	6.32
t3	9.16	59.28	100.00	46.48	6.32
Avg.	9.18	59.18	100.00	46.41	6.32
Std. Dev.	0.014	0.086	0.000	0.092	0.005

Fig. S6. (top) Representative example of one of the triplicate LOOP injections performed. (bottom) Table summarizing triplicate LOOP injections.

	[D]2a	[D]2b	[D]2c
**OLS**	20.1 (0.035)	33.7 (0.0098)	46.2 (0.016)
**Calib. UV**	20.8	42.7	36.5

Table S1. OLS deconvoluted product ratios vs calibrated UV of deutero trial, for the OLS values p-values are shown in parentheses.

5.1.4 Quantitation of Regioisomers By Calibrated UPLC via TWC and TIC

Calibration with Unlabeled Regioisomers. Methyl 2-(*para*-tolyl)nicotinate (**[H]2a**) (4.6 mg), methyl 4-(*para*-tolyl)nicotinate(**[H]2b**) (4.6 mg), and methyl 6-(*para*-tolyl)nicotinate (**[H]2c**) (4.6 mg) were combined and dried under high vacuum overnight. After dissolution in $CDCl_3$ (1 mL), their relative amounts were determined ratiometrically from methyl ester integrations in the ^1H NMR spectrum (600 MHz, d1 = 30 s). After removal of solvent, MeCN (10 mL) was added to form a stock solution (2 mM in each regioisomer). Correction factors for UV activity (total wavelength chromatogram, 190-400 nm) as well as relative ionization efficiency (sum of raw peak intensities in the M+1/M+2 cluster) were determined from duplicate runs at multiple dilutions.

UV correction factors for **[H]2a**, **[H]2b**, and **[H]2c** were: 1.90 ± 0.05, 1.72 ± 0.08, and 1.00, respectively. Ionization correction factors for **[H]2a**, **[H]2b**, and **[H]2c** were: 1.18 ± 0.04, 1.00, and 1.42 ± 0.06, respectively.

Calibration with Deuterated Regioisomers. D-Methyl 2-(*para*-tolyl)nicotinate (**[D]2a**) (2.3 mg), D-methyl 4-(*para*-tolyl)nicotinate (**[D]2b**) (2.3 mg), and D-methyl 6-(*para*-tolyl)nicotinate (**[H]2c**) (2.3 mg) were mixed and dried under high vacuum overnight. After dissolution in $CDCl_3$ (1 mL), their relative amounts were determined ratiometrically from methyl ester integrations in the ^1H NMR spectrum (600 MHz, d1 = 30 s). After removal of solvent, MeCN (10 mL) was added to form a stock solution (1 mM in each regioisomer). Correction factors for UV activity (total wavelength chromatogram, 190-400 nm) as well as relative ionization efficiency (sum of raw peak intensities in the M+1 to M+5 cluster) were determined from duplicate runs at multiple dilutions.

UV correction factors for **[D]2a**, **[D]2b**, and **[D]2c** were: 1.91 ± 0.15, 1.82 ± 0.08, and 1.00 respectively. Ionization correction factors for **[D]2a**, **[D]2b**, and **[D]2c** were: 1.16 ± 0.02, 1.00, and 1.54 ± 0.07, respectively.

Given that the relative correction factors for each set of regioisomers (labeled/unlabeled) were within statistical & experimental errors, we conclude that deuteration does not affect the LC-UV or MS responses meaningfully in terms of quantitation.

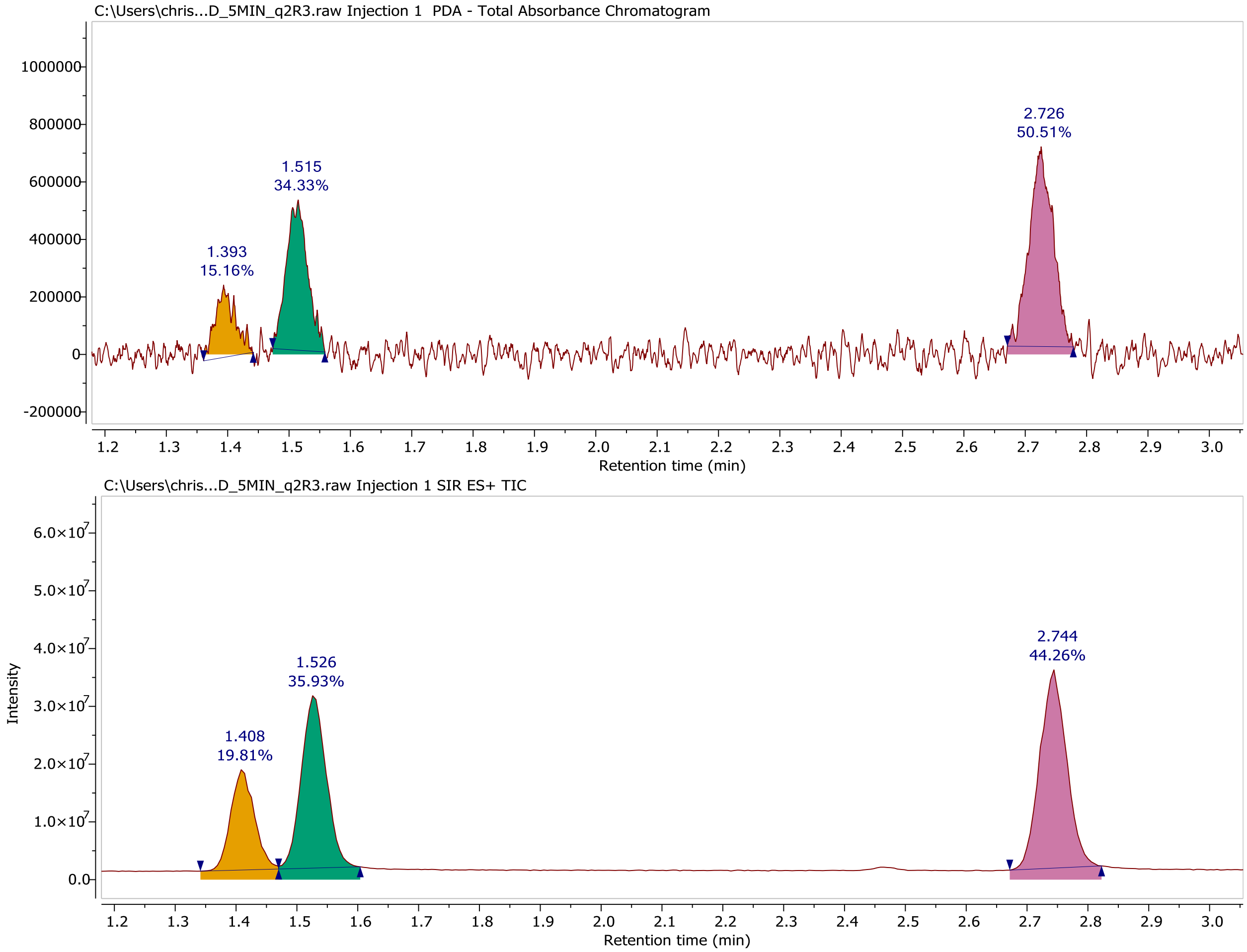

Fig. S7. UPLC trace (top) and TIC trace (bottom) of the above mixture of **[D]2a**, **[D]2b**, and **[D]2c**.

5.2 24-Well HTE Plate: Pyridine Substrates [D]2–[D]7

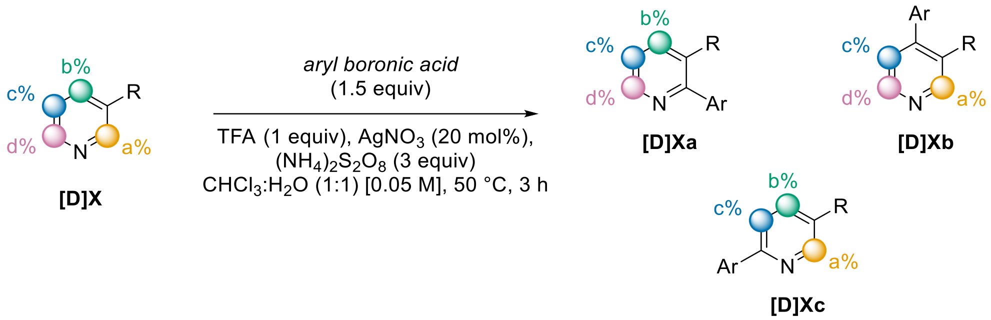

5.2.1 General Procedure for HTE-Scale Minisci Arylations

Screening reactions were carried out in 1 mL vials (30 mm height x 8 mm diameter) in a 24-well plate aluminum reactor block. Liquid solutions were dosed manually via micropipette. Reactions were heated and stirred on a heating block with a tumble-stirrer (V&P Scientific) using 1.98 mm diameter x 4.80 mm length parylene stir bars. Reaction vials were sealed in the 24-well plate during reaction. Below each reactor vial in the aluminum 24-well plate was a 0.062 mm thick silicon-rubber gasket. Directly above the glass vial reactor tops was a Teflon perfluoroalkoxy copolymer resin sealing gasket and above that, two more 0.062 mm silicon-rubber gaskets. The entire assembly was compressed between an aluminum top and the reactor base with 9 evenly placed screws.

Experiments were set up inside a hood under air. The 24-well aluminum block containing 1 mL glass vials were first dosed with solutions of isotopically labeled heterocycles in $CHCl_3$ (10 μmol per 25 μL), followed by freshly prepared aqueous solutions of TFA (10 μmol per 10 μL) and solutions of aryl boronic acids in $CHCl_3$ (15 μmol per 75 μL). An aqueous solution of $AgNO_3$ (2 μmol per 10 μL) was dosed into each vial followed by an aqueous solution of $(NH_4)_2S_2O_8$ (30 μmol per 80 μL). The plate was sealed with screws and stirred at 50 ºC for 3 h.

After cooling to ambient temperature, the vials were quenched with satd $NaHCO_3$ (200 μL) and diluted with $CHCl_3$ (400 μL). The plate was allowed to stir for 10 min at rt on an IKA stirplate. After separation of the biphasic layers, 10 μL aliquots from the organic layer of each diluted well were transferred to a 96-well LC plate, followed by 990 μL of a 4,4'-di-tert-butylbiphenyl (dtbbp) solution in MeCN (10.1 μM). The mixture was then analyzed using Waters MassLynx on a Waters ACQUITY UPLC-MS SQ system as described below.

Note that HTE-scale Minisci reactions were conducted at 0.05 M concentration, rather than 0.1 M, to allow for better solubility of arylboronic acids in $CHCl_3$ stock solutions.

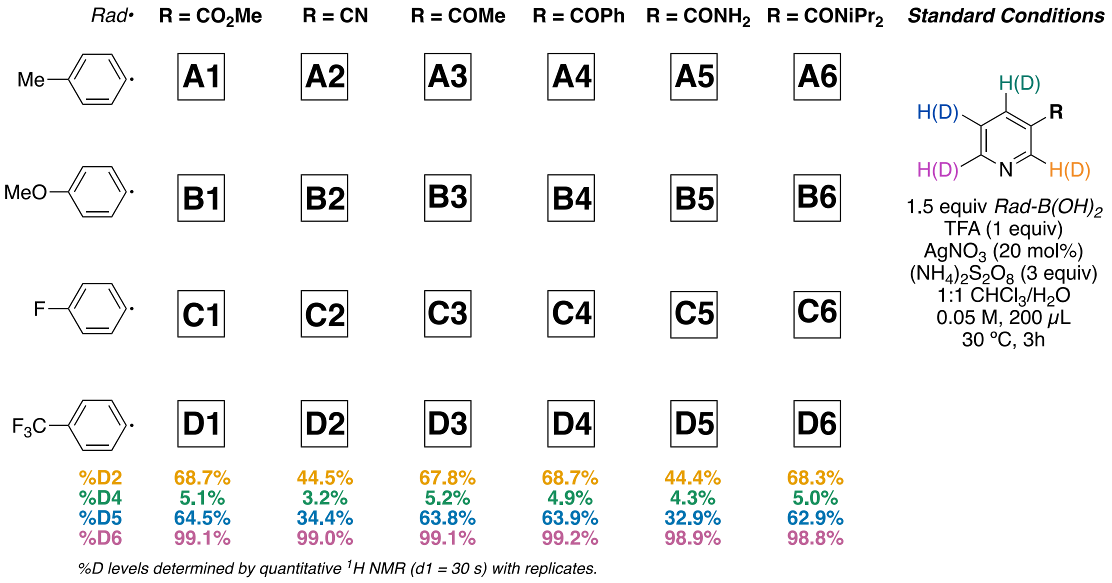

Fig. S8. Representation of 24-well HTE plat. Rows are represented by letters A-D and columns are represented by numbers 1-6.

5.2.2 UPLC Analysis of Regioselectivities for the 24-Well Plate

UPLC conditions (Acquity SQ): Premier BEH C18; 2.4 µm; 50mm x 2.1mm; Column T = 40 °C; 0.75 mL/min. Gradient (5 min, 0.1% formic acid additive): ***A or B***. Detection: UV-Vis (TWC 210 – 400 nm); MS (ESI+, *m/z* = ***varied***, 30 V, 20 Hz).

Gradient A: 0.55 min at 75% MeCN/H_2O; 2.45 min gradient to 65% MeCN/H_2O; 1.85 min gradient to 25% MeCN/H_2O; 0.05 min gradient to 95% MeCN/H_2O; 0.10 min at 95% MeCN/H_2O.

Gradient B: 0.55 min at 100% MeCN/H_2O (0.1% FA); 2.45 min gradient to 75% MeCN/H_2O (0.1% FA); 1.85 min gradient to 25% MeCN/H_2O (0.1% FA); 0.05 min gradient to 95% MeCN/H_2O (0.1% FA); 0.10 min at 95% MeCN/H_2O (0.1% FA).

Gradient A was employed for wells: A1, A2, A4, A6, B2, B3, B4, B6, C1, C2, C3, C4, C6, D1, D2, D3, D4, D6. Gradient B was employed for wells: A3, A5, B1, B5, C5, D5.

Custom MS files were written to capture narrow *m/z* ranges of 15 Da to improve signal-to-noise for quantitative measurements and are noted for each reaction well. Total absorbance chromatograms (TACs) have been processed with trace baseline corrections.

Although the R5 regioisomer may in principle form under these arylative Minisci conditions, it is so minor (≤ 5% relative) that corresponding UPLC-MS distributions could not be cleanly extracted. The presence of this regioisomer has a minor effect on deconvoluted selectivities as discussed later.

UPLC separations between all three heteroaryl regioisomers (R2/R4/R6) were attained for 17 of the 24 reaction wells. Separation of the R2/R4 regioisomers was infeasible for the remaining 7.

UPLC-MS Analysis, A1

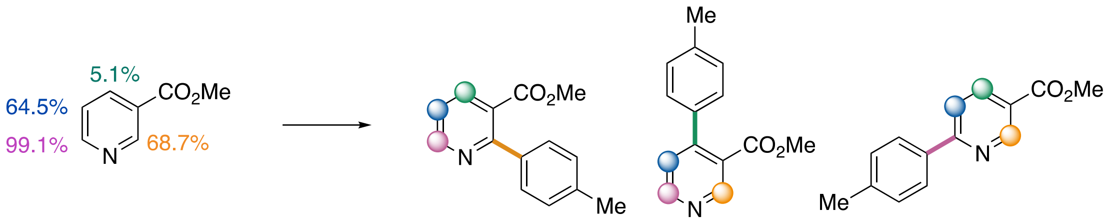

Fig. S9. Reaction **A1**, following the HTE general procedure detailed above.

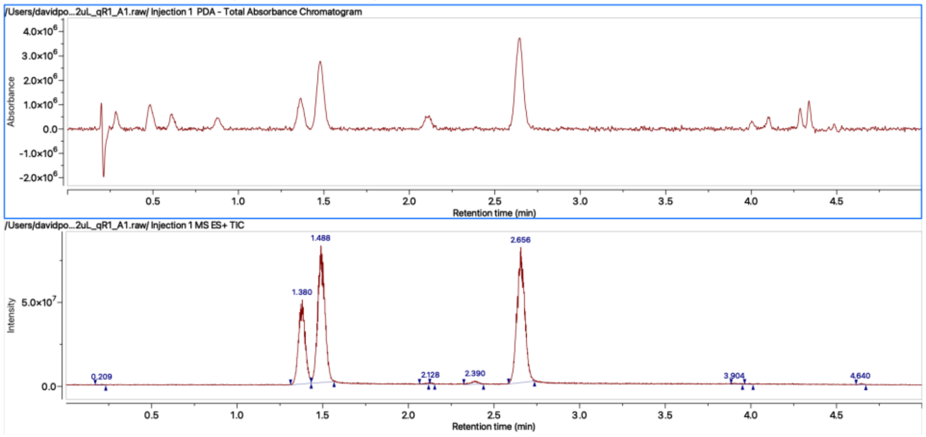

Fig. S10. Direct UPLC-MS analysis of reaction well **A1**, with *m/z* range 225-240 Da.

UPLC, A1												
		rt				rt				rt		
	m/z	1.31..1.43	R2		m/z	1.43..1.56	R4		m/z	2.59..2.73	R6	
M+1	228.461	4.08E+06	0.32		228.425	1.40E+07	0.80		228.452	5.41E+08	28.10	
M+2	229.478	7.73E+08	61.31		229.471	4.86E+08	27.54		229.471	1.66E+09	86.18	
M+3	230.481	1.26E+09	100.00		230.480	1.64E+09	93.10		230.479	1.93E+09	100.00	
M+4	231.490	2.50E+08	19.82		231.490	1.77E+09	100.00		231.489	3.85E+08	20.01	
M+5	232.500	2.41E+07	1.91		232.500	2.63E+08	14.89		232.498	3.63E+07	1.88	

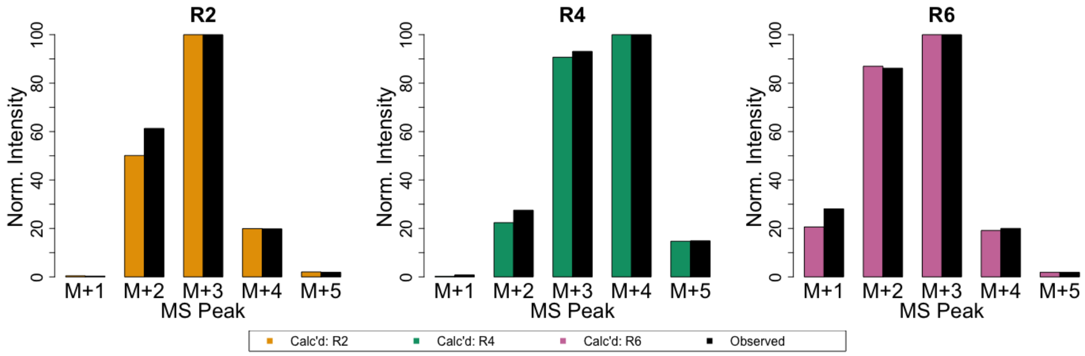

Fig. S11. Observed isotopic distributions for each of the three product regioisomers, with retention times noted for crosshair selections (top). Comparison of expected isotopic distributions (respective colors) versus observed isotopic distributions (black) for each of the three product regioisomers (bottom).

UPLC-MS Analysis, A2

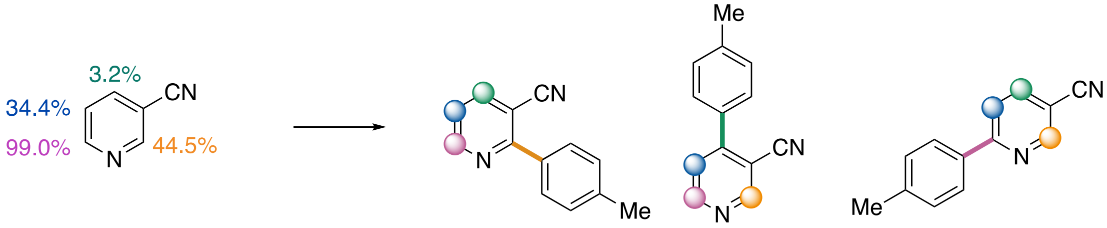

Fig. S12. Reaction **A2**, following the HTE general procedure detailed above.

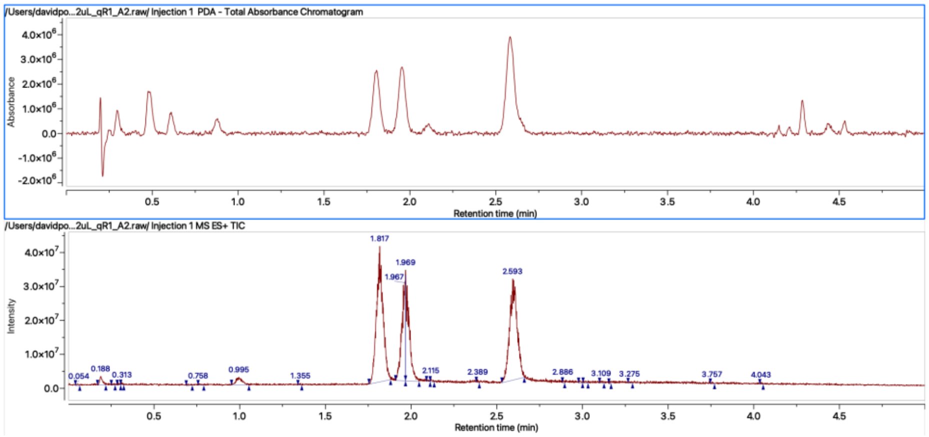

Fig. S13. Direct UPLC-MS analysis of reaction well **A2**, with *m/z* range 190-205 Da.

UPLC, A2									
		rt			rt			rt	
	m/z	1.75..1.88	R2	m/z	1.91..2.04	R4	m/z	2.53..2.66	R6
M+1	195.422	6.54E+06	0.64	195.430	2.10E+06	0.29	195.421	5.08E+08	64.68
M+2	196.436	1.02E+09	100.00	196.430	5.49E+08	74.86	196.430	7.86E+08	100.00
M+3	197.447	7.10E+08	69.59	197.443	7.33E+08	100.00	197.438	3.43E+08	43.66
M+4	198.443	1.17E+08	11.45	198.449	3.11E+08	42.40	198.431	5.35E+07	6.81
M+5	199.444	8.37E+06	0.82	199.443	3.78E+07	5.16	199.410	5.89E+06	0.75

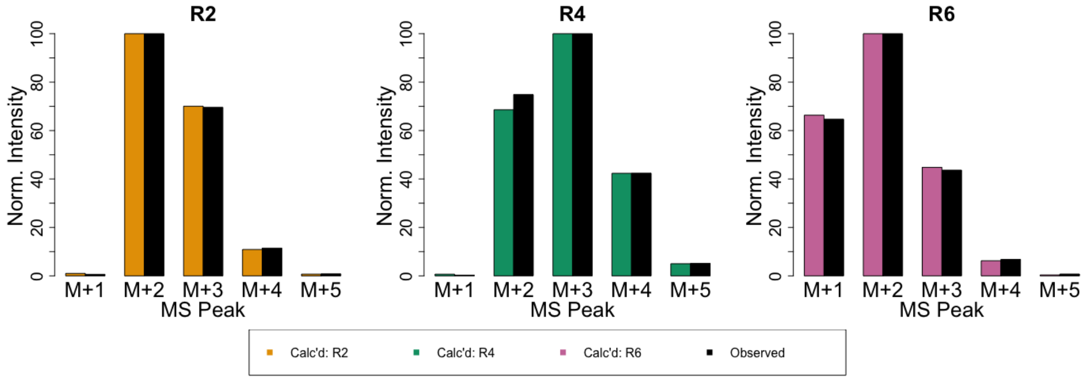

Fig. S14. Observed isotopic distributions for each of the three product regioisomers, with retention times noted for crosshair selections (top). Comparison of expected isotopic distributions (respective colors) versus observed isotopic distributions (black) for each of the three product regioisomers (bottom).

UPLC-MS Analysis, A3

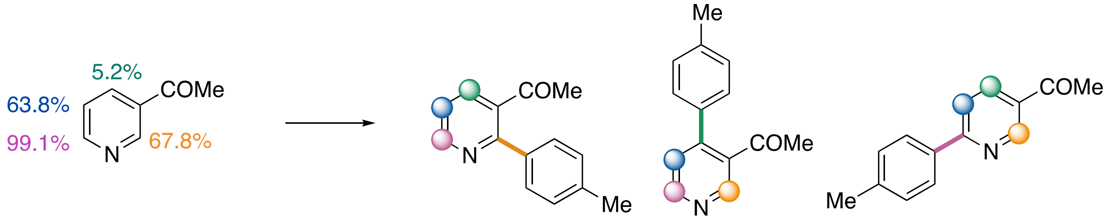

Fig. S15. Reaction **A3**, following the HTE general procedure detailed above.

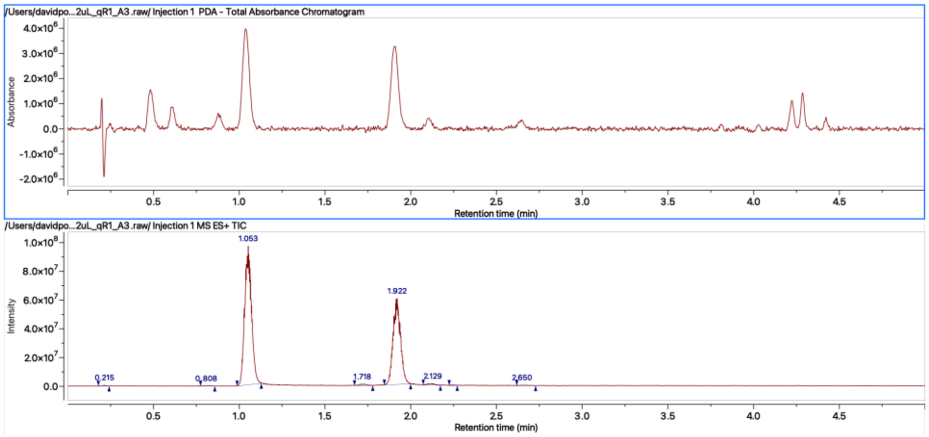

Fig. S16. Direct UPLC-MS analysis of reaction well **A3**, with *m/z* range 210-225 Da.

UPLC, A3							
		rt		rt			rt
	m/z	0.99..1.13	R2R4	m/z	1.84..2.00	R6	
M+1	212.471	4.25E+06	0.20	212.456	3.37E+08	24.16	
M+2	213.471	8.71E+08	41.80	213.462	1.24E+09	89.28	
M+3	214.479	2.08E+09	100.00	214.470	1.39E+09	100.00	
M+4	215.486	1.52E+09	72.86	215.480	2.73E+08	19.61	
M+5	216.491	2.11E+08	10.14	216.488	2.08E+07	1.49	

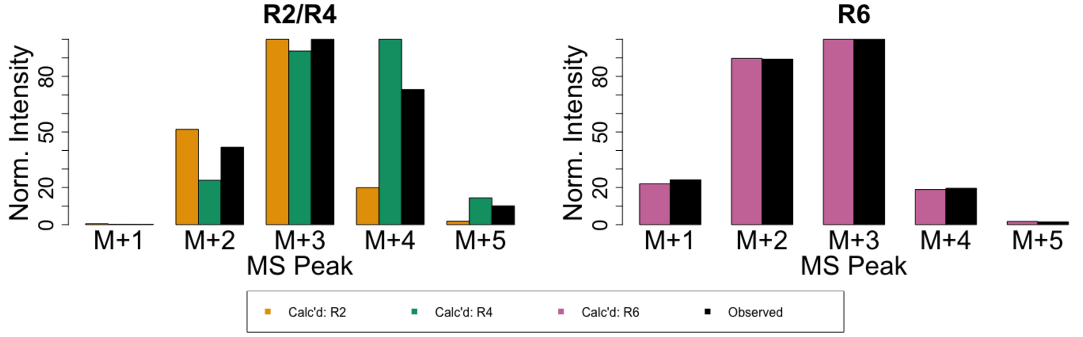

Fig. S17. Observed isotopic distributions for each of the two observed UPLC-MS distributions, with retention times noted for crosshair selections (top). Comparison of expected isotopic distributions (respective colors) versus observed isotopic distributions (black) for each of the two observed UPLC-MS distributions (bottom).

The first major peak (ESI+ t_R peak 1.05) can be clearly assigned as a mixture of the R2/R4 regioisomers. Deconvolution via ordinary least-squares (OLS) regression yields **R2**:**R4** ratios of **1.0**:**1.9** and **1.0**:**1.7** (**C_{NMR}** and **C_{LC}**, respectively).
See above section for details on OLS deconvolution.

UPLC-MS Analysis, A4

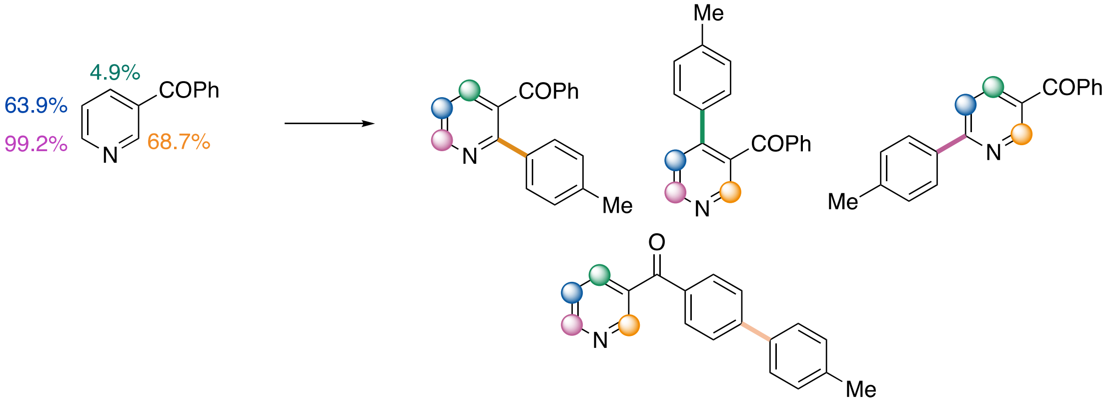

Fig. S18. Reaction **A4**, following the HTE general procedure detailed above.

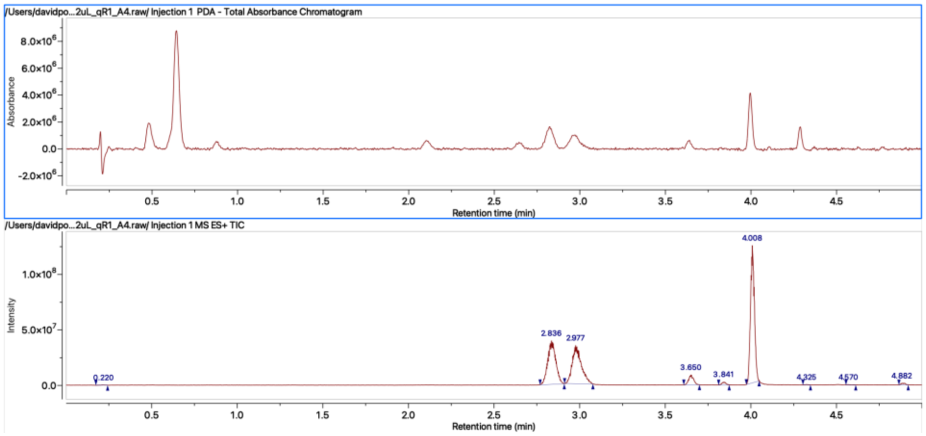

Fig. S19. Direct UPLC-MS analysis of reaction well **A4**, with *m/z* range 270-285 Da.

**UPLC, A4**		rt			rt			rt			rt	
	m/z	2.77..2.91	R4	m/z	2.91..3.08	R2	m/z	3.61..3.70	R-Ph	m/z	3.97..4.05	R6
M+1	274.579	1.38E+06	0.14	274.579	3.13E+06	0.26	274.617	7.87E+05	0.60	274.522	3.47E+08	22.43
M+2	275.528	2.27E+08	23.16	275.530	6.30E+08	51.64	275.530	2.72E+07	20.84	275.531	1.29E+09	83.53
M+3	276.539	8.63E+08	87.92	276.539	1.22E+09	100.00	276.534	1.17E+08	89.99	276.541	1.55E+09	100.00
M+4	277.550	9.82E+08	100.00	277.545	4.28E+08	35.10	277.550	1.30E+08	100.00	277.551	3.71E+08	23.98
M+5	278.544	1.90E+08	19.32	278.537	6.88E+07	5.64	278.538	3.04E+07	23.33	278.545	4.72E+07	3.05

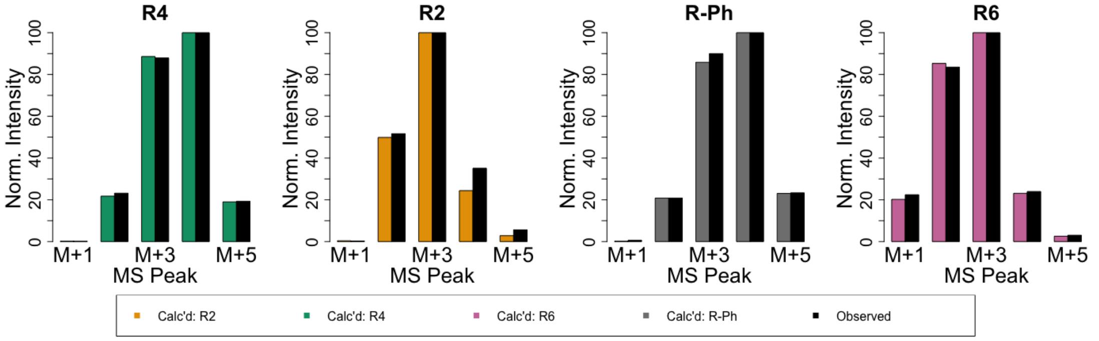

Fig. S20. Observed isotopic distributions for each of the four observed UPLC-MS distributions, with retention times noted for crosshair selections (top). Comparison of expected isotopic distributions (respective colors) versus observed isotopic distributions (blue) for each of the four observed UPLC-MS distributions (bottom).

Given the close similarity of expected distributions for **R4** and **R-Ph**, these isomers cannot be definitively distinguished based on their isotopic distributions alone. The small relative amount of the t_R 3.65 peak makes assignment as **R-Ph** a more reasonable assumption.

Additionally, the t_R 3.65 peak cannot be reasonably assigned as the **R5** regioisomer (which appears to correspond to the very minor ESI+ peak at t_R 3.84) based on an inadequate match with the expected **R5** isotopic distribution.

UPLC-MS Analysis, A5

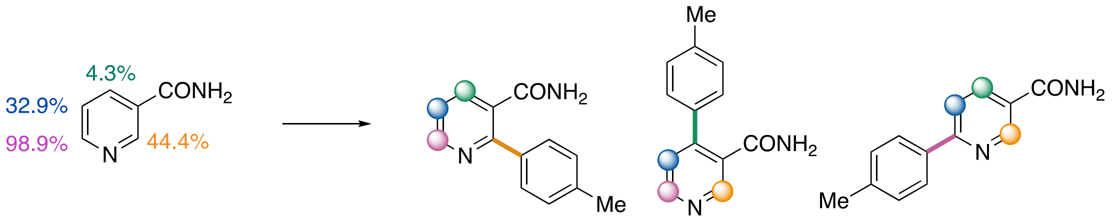

Fig. S21. Reaction **A5**, following the HTE general procedure detailed above.

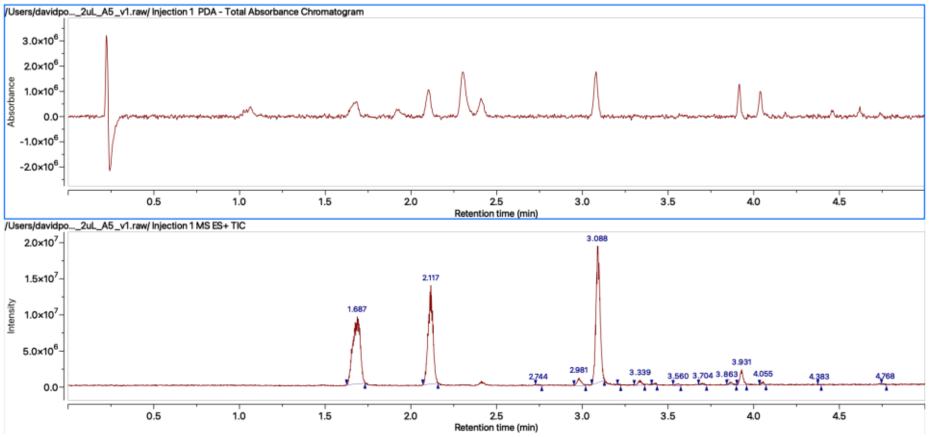

Fig. S22. Direct UPLC-MS analysis of reaction well **A5**, with *m/z* range 210-225 Da.

**UPLC, A5**									
		rt			rt			rt	
	m/z	1.62..1.73	R2	m/z	2.07..2.16	R4	m/z	3.06..3.13	R6
M+1	213.495	8.42E+05	0.30	213.413	6.54E+05	0.32	213.460	1.79E+08	68.91
M+2	214.474	2.82E+08	100.00	214.473	1.51E+08	73.38	214.466	2.60E+08	100.00
M+3	215.481	1.83E+08	64.88	215.481	2.06E+08	100.00	215.481	1.12E+08	43.17
M+4	216.491	2.38E+07	8.46	216.491	8.26E+07	40.17	216.480	1.18E+07	4.54
M+5	217.503	5.09E+05	0.18	217.495	6.81E+06	3.31	217.486	4.67E+05	0.18

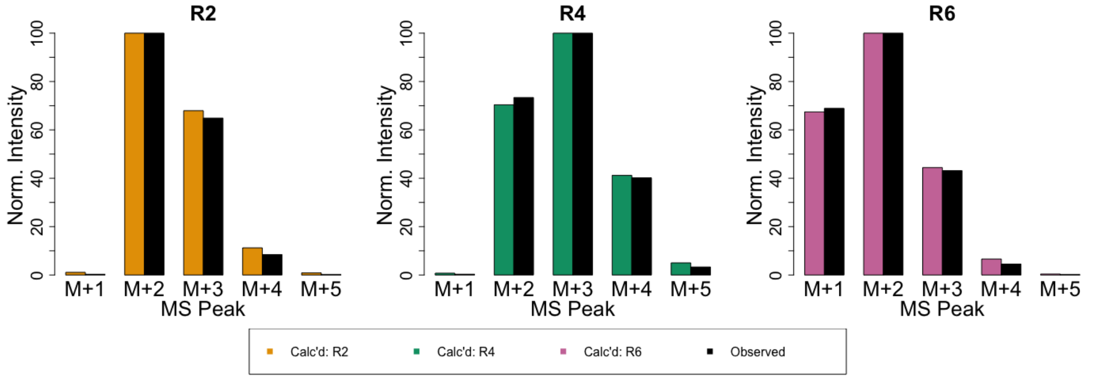

Fig. S23. Observed isotopic distributions for each of the three product regioisomers, with retention times noted for crosshair selections (top). Comparison of expected isotopic distributions (respective colors) versus observed isotopic distributions (black) for each of the three product regioisomers (bottom).

UPLC-MS Analysis, A6

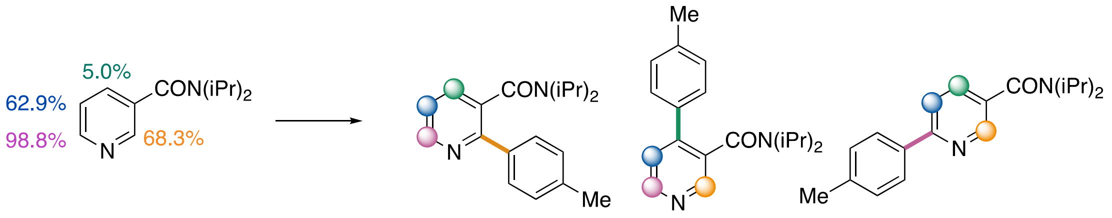

Fig. S24. Reaction **A6**, following the HTE general procedure detailed above.

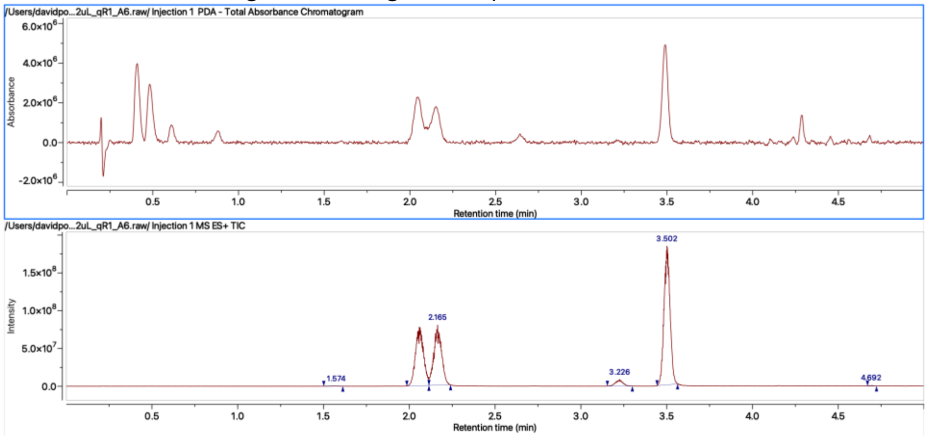

Fig. S25. Direct UPLC-MS analysis of reaction well **A6**, with *m/z* range 295-310 Da.

UPLC, A6									
		rt			rt			rt	
	m/z	1.99..2.12	R4	m/z	2.12..2.24	R2	m/z	3.45..3.56	R6
M+1	297.628	1.77E+06	0.09	297.601	7.40E+06	0.31	297.603	7.83E+08	23.02
M+2	298.610	4.43E+08	22.88	298.613	1.24E+09	51.84	298.613	2.90E+09	85.27
M+3	299.622	1.66E+09	85.67	299.622	2.38E+09	100.00	299.622	3.40E+09	100.00
M+4	300.632	1.93E+09	100.00	300.631	6.18E+08	25.94	300.631	8.50E+08	25.01
M+5	301.639	3.76E+08	19.44	301.625	7.36E+07	3.09	301.635	1.07E+08	3.14

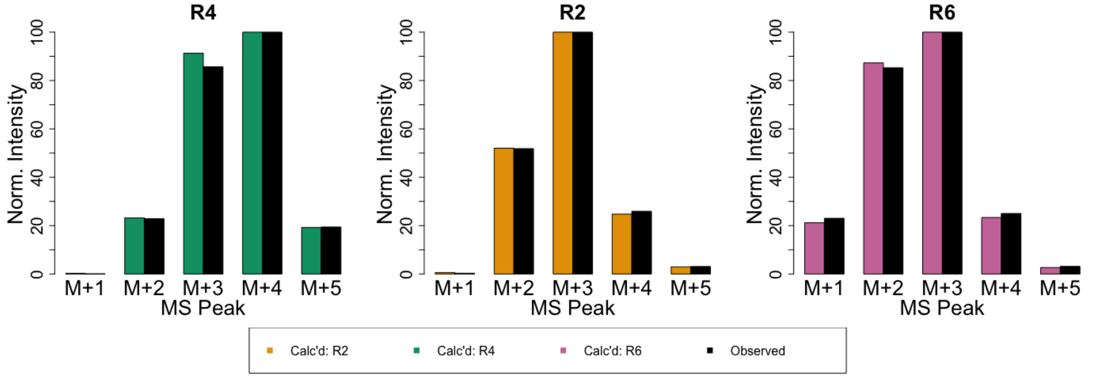

Fig. S26. Observed isotopic distributions for each of the three product regioisomers, with retention times noted for crosshair selections (top). Comparison of expected isotopic distributions (respective colors) versus observed isotopic distributions (black) for each of the three product regioisomers (bottom).

UPLC-MS Analysis, B1

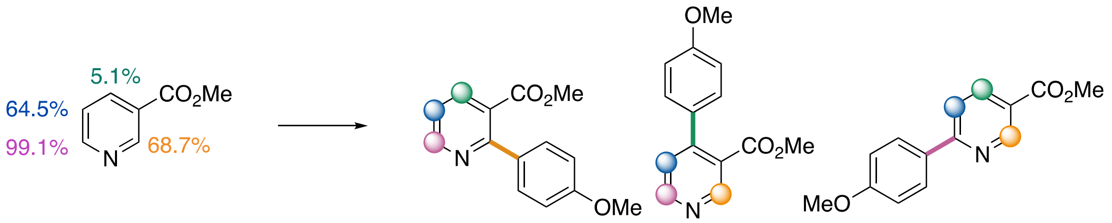

Fig. S27. Reaction **B1**, following the HTE general procedure detailed above.

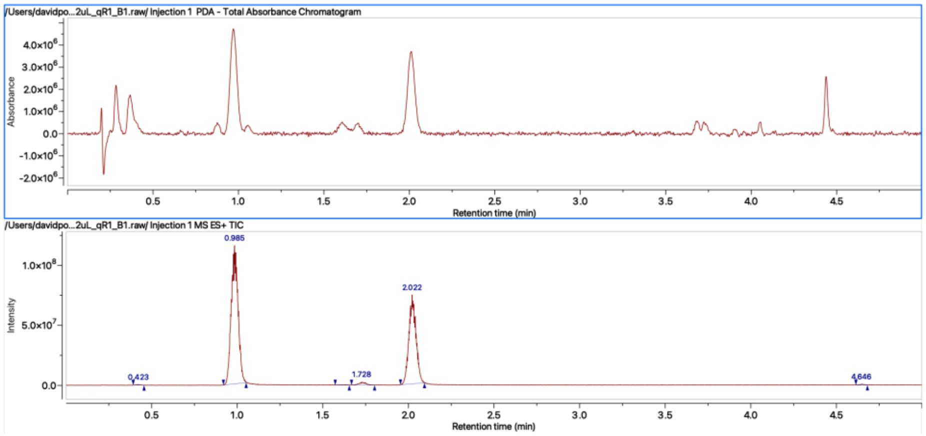

Fig. S28. Direct UPLC-MS analysis of reaction well **B1**, with *m/z* range 240-255 Da.

UPLC, B1						
		rt			rt	
	m/z	0.92..1.05	R2R4	m/z	1.95..2.10	R6
M+1	244.473	6.47E+06	0.26	244.479	3.82E+08	23.25
M+2	245.486	1.19E+09	48.47	245.487	1.43E+09	86.95
M+3	246.496	2.46E+09	100.00	246.496	1.64E+09	100.00
M+4	247.507	1.74E+09	70.83	247.505	3.26E+08	19.86
M+5	248.512	2.60E+08	10.54	248.506	3.41E+07	2.08

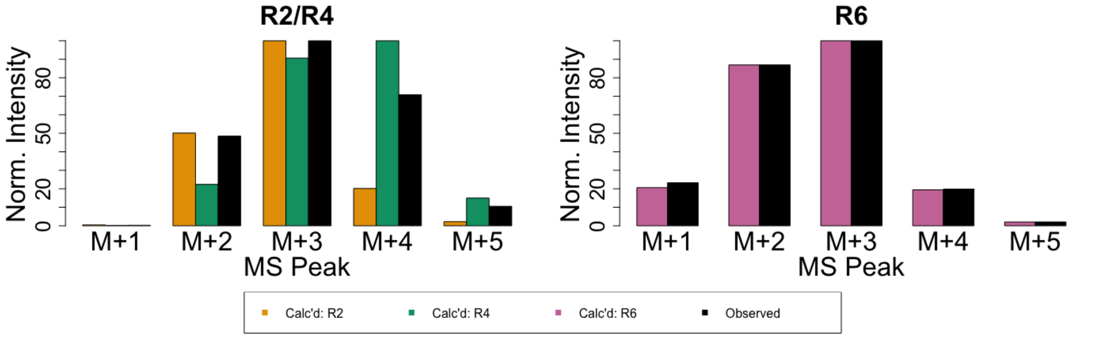

Fig. S29. Observed isotopic distributions for each of the two observed UPLC-MS distributions, with retention times noted for crosshair selections (top). Comparison of expected isotopic distributions (respective colors) versus observed isotopic distributions (black) for each of the three product regioisomers (bottom).

The first major peak (ESI+ t_R peak 0.99) can be clearly assigned as a mixture of the R2/R4 regioisomers. Deconvolution via ordinary least-squares (OLS) regression yields **R2**:**R4** ratios of **1.0**:**1.5** and **1.0**:**1.7** (**C**$_{NMR}$ and **C**$_{LC}$, respectively).

See above section for details on OLS deconvolution.

UPLC-MS Analysis, B2

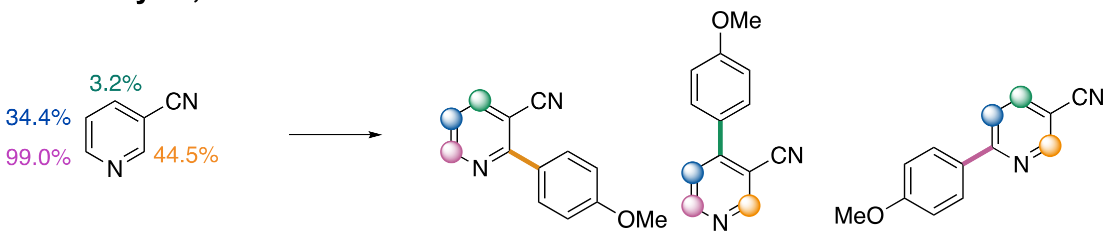

Fig. S30. Reaction **B2**, following the HTE general procedure detailed above.

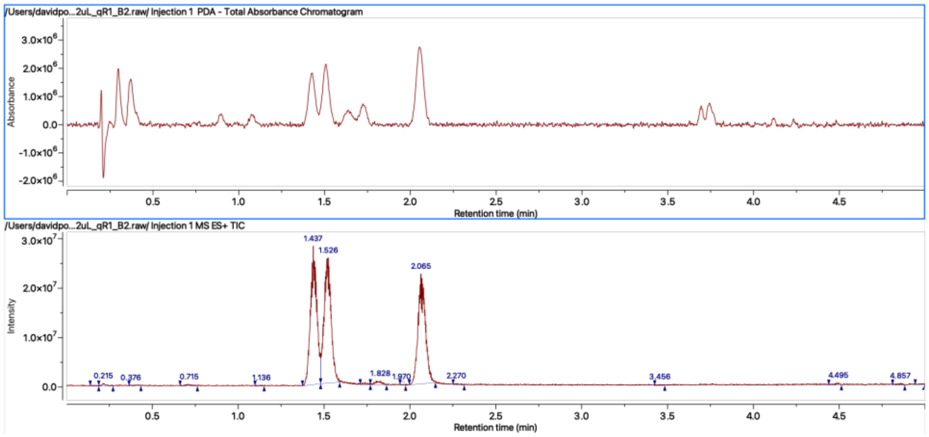

Fig. S31. Direct UPLC-MS analysis of reaction well **B2**, with *m/z* range 205-220 Da.

UPLC, B2									
		rt			rt			rt	
	m/z	1.37..1.48	R2	m/z	1.48..1.59	R4	m/z	2.00..2.15	R6
M+1	211.477	3.91E+06	0.58	211.463	1.16E+06	0.20	211.448	3.58E+08	68.74
M+2	212.462	6.75E+08	100.00	212.461	4.45E+08	76.46	212.457	5.21E+08	100.00
M+3	213.474	4.57E+08	67.71	213.472	5.82E+08	100.00	213.468	2.31E+08	44.36
M+4	214.481	7.55E+07	11.18	214.481	2.37E+08	40.71	214.459	2.85E+07	5.47
M+5	215.491	3.76E+06	0.56	215.488	2.42E+07	4.16	215.489	1.55E+06	0.30

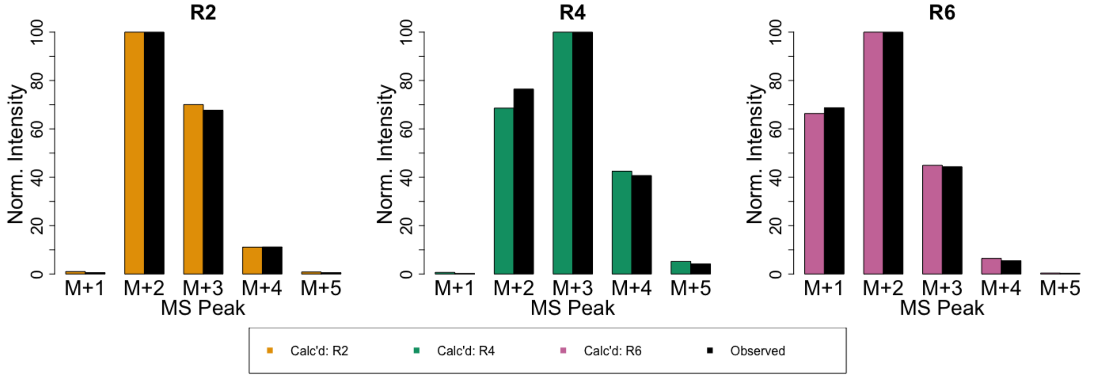

Fig. S32. Observed isotopic distributions for each of the three product regioisomers, with retention times noted for crosshair selections (top). Comparison of expected isotopic distributions (respective colors) versus observed isotopic distributions (black) for each of the three product regioisomers (bottom).

UPLC-MS Analysis, B3

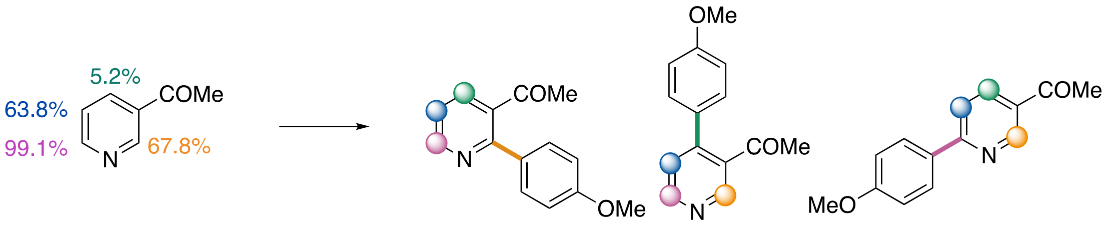

Fig. S33. Reaction **B3**, following the HTE general procedure detailed above.

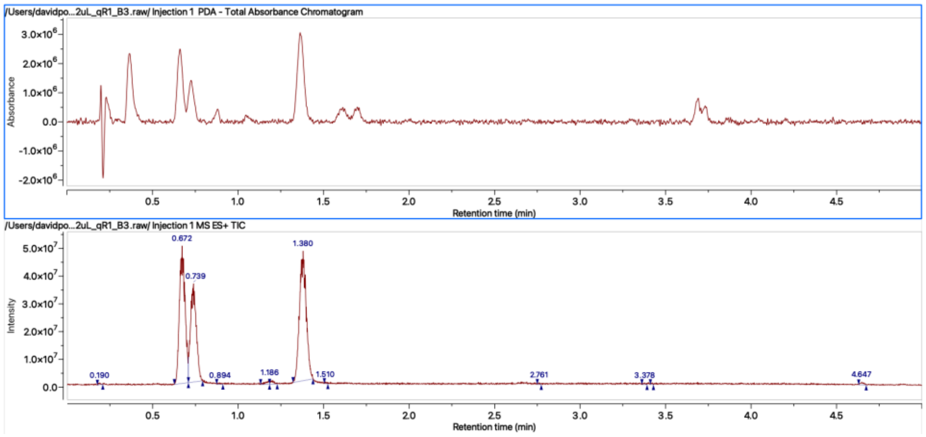

Fig. S34. Direct UPLC-MS analysis of reaction well **B3**, with *m/z* range 225-240 Da.

UPLC, B3		rt				rt				rt	
	m/z	0.63..0.71	R4	m/z	0.71..0.79	R2	m/z	1.32..1.44	R6
M+1	228.361	5.07E+07	5.83	228.367	5.38E+07	6.97	228.451	2.93E+08	28.91
M+2	229.480	2.24E+08	25.74	229.482	5.93E+08	76.75	229.478	8.95E+08	88.25
M+3	230.490	8.05E+08	92.39	230.489	7.72E+08	100.00	230.483	1.01E+09	100.00
M+4	231.498	8.71E+08	100.00	231.494	1.75E+08	22.71	231.496	1.98E+08	19.51
M+5	232.508	1.32E+08	15.20	232.506	1.55E+07	2.01	232.499	1.79E+07	1.77

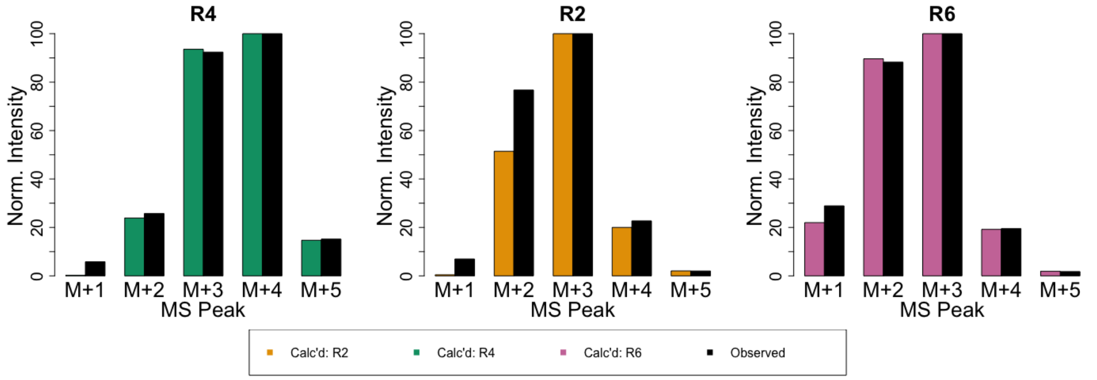

Fig. S35. Observed isotopic distributions for each of the three product regioisomers, with retention times noted for crosshair selections (top). Comparison of expected isotopic distributions (respective colors) versus observed isotopic distributions (black) for each of the three product regioisomers (bottom).

UPLC-MS Analysis, B4

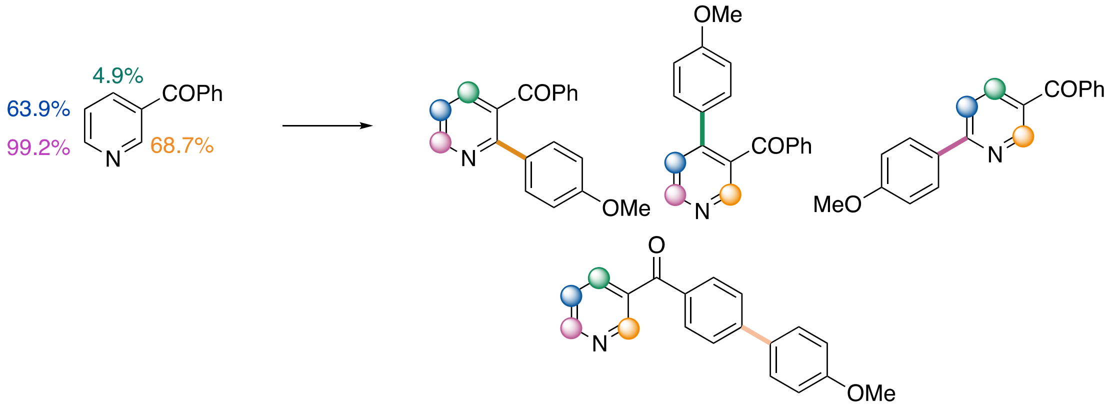

Fig. S36. Reaction **B4**, following the HTE general procedure detailed above.

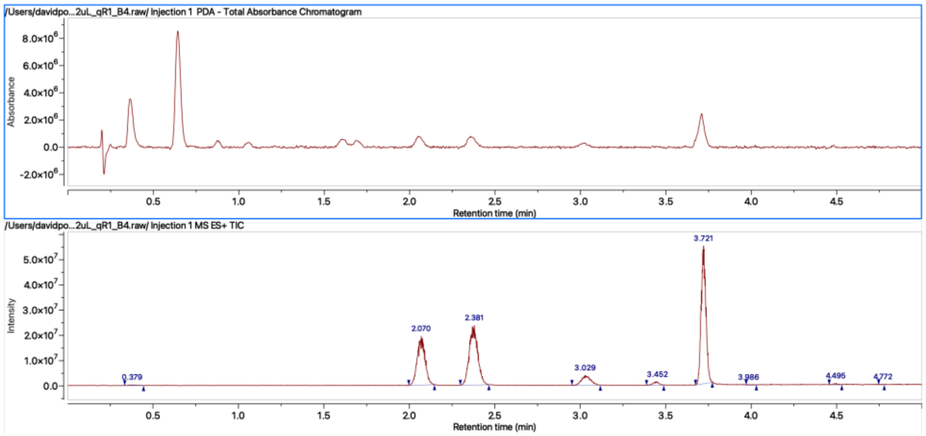

Fig. S37. Direct UPLC-MS analysis of reaction well **B4**, with *m/z* range 285-300 Da.

UPLC, B4												
		rt			rt			rt			rt	
	m/z	2.00..2.15	R4	m/z	2.30..2.47	R2	m/z	2.96..3.12	R-Ph	m/z	3.68..3.77	R6
M+1	290.556	2.35E+05	0.05	290.501	1.33E+06	0.19	290.501	1.75E+05	0.21	290.529	1.77E+08	22.08
M+2	291.536	1.01E+08	22.71	291.536	3.79E+08	55.25	291.540	1.54E+07	18.69	291.536	6.85E+08	85.23
M+3	292.543	3.88E+08	87.65	292.540	6.86E+08	100.00	292.536	7.72E+07	93.94	292.541	8.03E+08	100.00
M+4	293.554	4.43E+08	100.00	293.554	2.57E+08	37.52	293.555	8.22E+07	100.00	293.549	1.93E+08	24.06
M+5	294.551	8.74E+07	19.73	294.547	4.67E+07	6.81	294.527	2.14E+07	26.05	294.537	2.73E+07	3.40

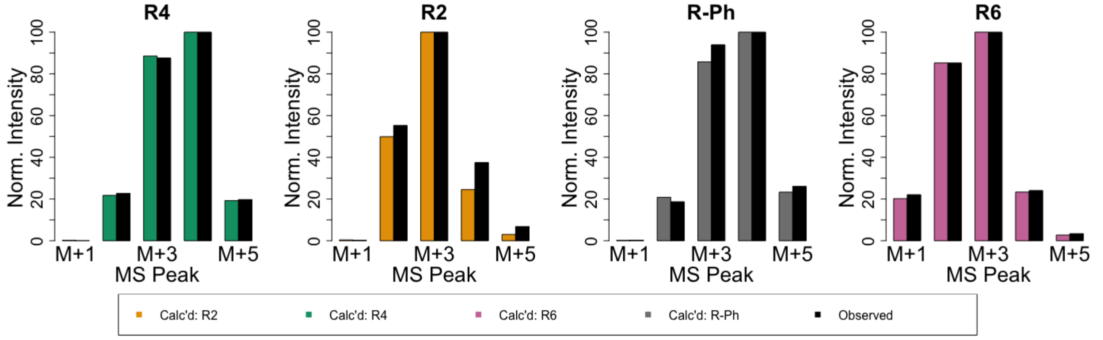

Fig. S38. Observed isotopic distributions for each of the four observed UPLC-MS distributions, with retention times noted for crosshair selections (top). Comparison of expected isotopic distributions (respective colors) versus observed isotopic distributions (black) for each of the four observed UPLC-MS distributions (bottom).

Given the close similarity of expected distributions for **R4** and **R-Ph**, these isomers cannot be definitively distinguished based on their isotopic distributions alone. The small relative amount of the t_R 3.03 peak makes assignment as **R-Ph** a more reasonable assumption. As above for well **A4**, the MS ion pattern of this peak does not agree well with the expected **R5** MS ion pattern.

UPLC-MS Analysis, B5

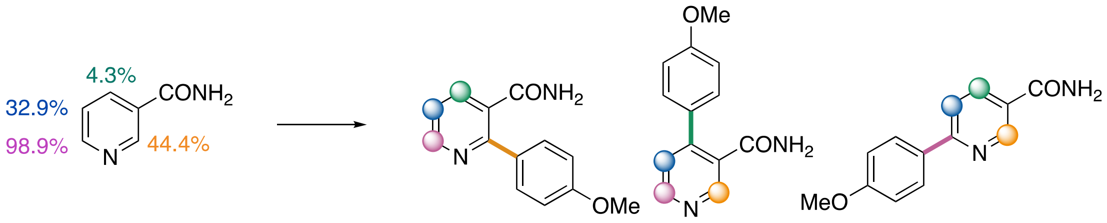

Fig. S39. Reaction **B5**, following the HTE general procedure detailed above.

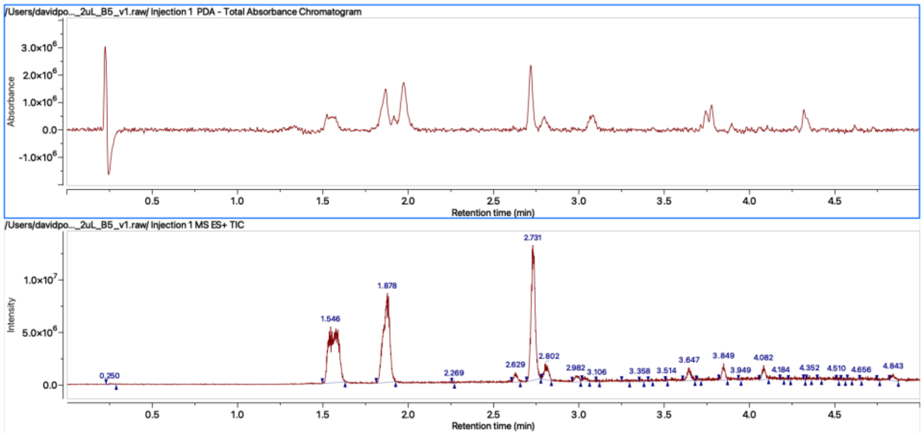

Fig. S40. Direct UPLC-MS analysis of reaction well **B5**, with *m/z* range 225-240 Da.

UPLC, B5									
		rt			rt			rt	
	m/z	1.50..1.63	R2	m/z	1.82..1.93	R4	m/z	2.69..2.78	R6
M+1	229.499	3.22E+05	0.16	229.501	6.49E+05	0.40	229.476	1.19E+08	70.63
M+2	230.491	2.00E+08	100.00	230.494	1.16E+08	70.66	230.488	1.68E+08	100.00
M+3	231.497	1.29E+08	64.60	231.497	1.63E+08	100.00	231.497	7.33E+07	43.61
M+4	232.503	1.51E+07	7.54	232.512	6.91E+07	42.27	232.503	8.54E+06	5.08
M+5	233.518	7.68E+05	0.38	233.536	4.89E+06	2.99	233.491	2.40E+05	0.14

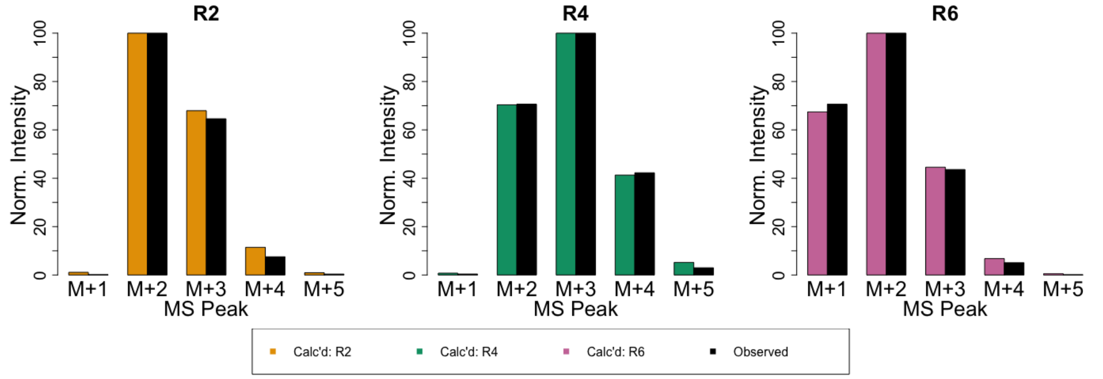

Fig. S41. Observed isotopic distributions for each of the three product regioisomers, with retention times noted for crosshair selections (top). Comparison of expected isotopic distributions (respective colors) versus observed isotopic distributions (black) for each of the three product regioisomers (bottom).

UPLC-MS Analysis, B6

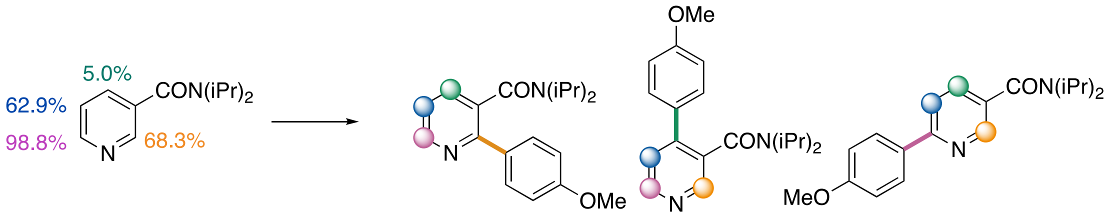

Fig. S42. Reaction **B6**, following the HTE general procedure detailed above.

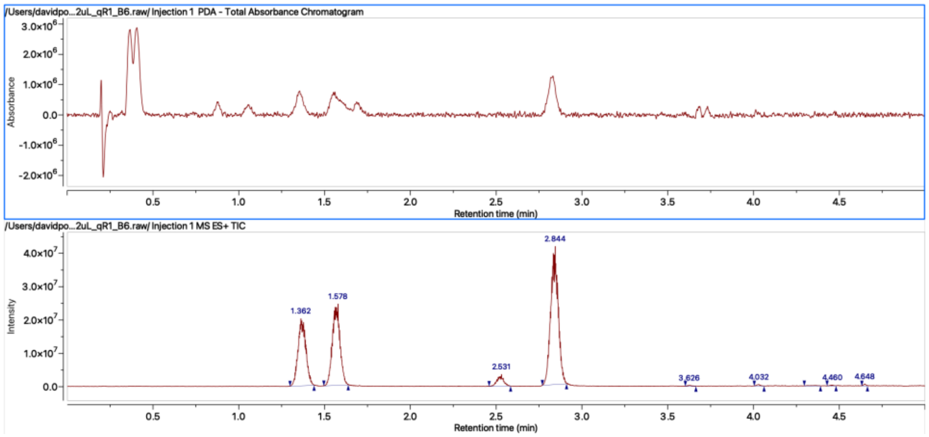

Fig. S43. Direct UPLC-MS analysis of reaction well **B6**, with *m/z* range 310-325 Da.

UPLC, B6									
		rt			rt			rt	
	m/z	1.30..1.44	R4	m/z	1.49..1.64	R2	m/z	2.77..2.91	R6
M+1	313.619	3.20E+05	0.07	313.615	1.10E+06	0.16	313.611	1.99E+08	21.20
M+2	314.622	1.06E+08	22.73	314.622	3.72E+08	53.45	314.625	7.74E+08	82.49
M+3	315.635	4.06E+08	87.45	315.634	6.97E+08	100.00	315.635	9.38E+08	100.00
M+4	316.641	4.64E+08	100.00	316.636	1.75E+08	25.09	316.635	2.24E+08	23.83
M+5	317.642	9.10E+07	19.60	317.628	2.15E+07	3.08	317.627	2.68E+07	2.85

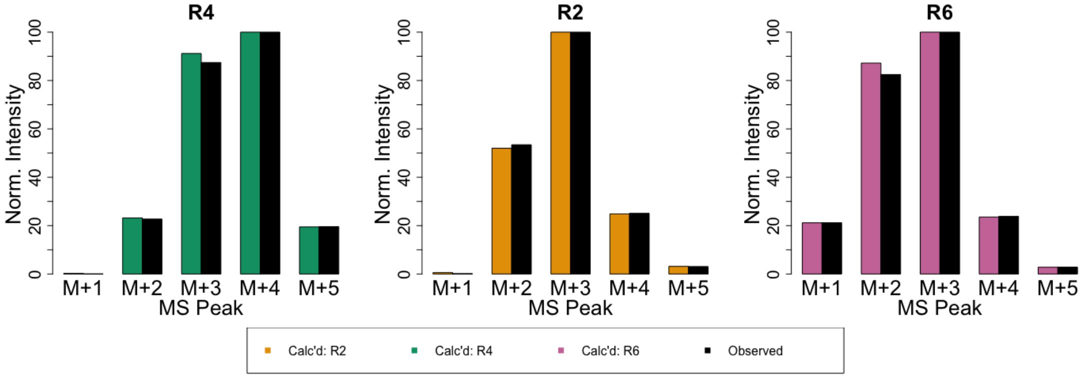

Fig. S44. Observed isotopic distributions for each of the three product regioisomers, with retention times noted for crosshair selections (top). Comparison of expected isotopic distributions (respective colors) versus observed isotopic distributions (black) for each of the three product regioisomers (bottom).

UPLC-MS Analysis, C1

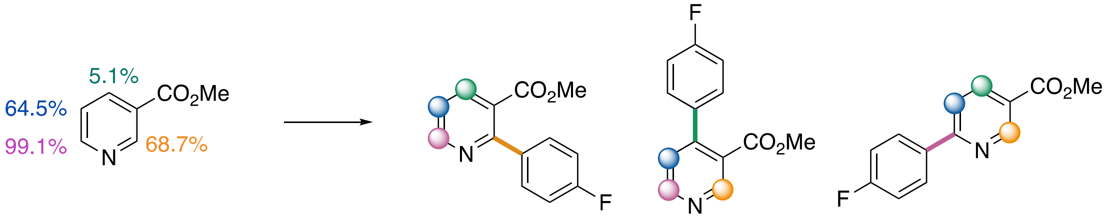

Fig. S45. Reaction **C1**, following the HTE general procedure detailed above.

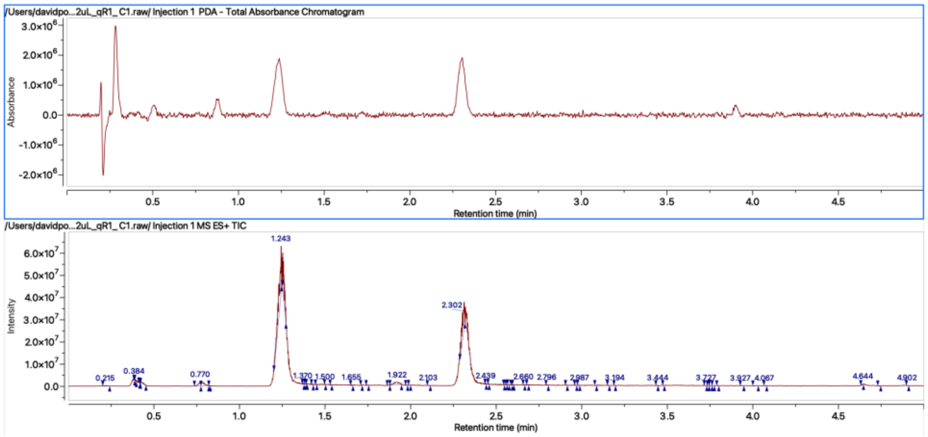

Fig. S46. Direct UPLC-MS analysis of reaction well **C1**, with *m/z* range 230-245 Da.

UPLC, C1						
		rt			rt	
	m/z	1.19..1.32	R2R4	m/z	2.26..2.39	R6
M+1	232.446	2.27E+06	0.14	232.444	2.07E+08	23.59
M+2	233.458	6.46E+08	41.23	233.457	7.63E+08	87.10
M+3	234.469	1.57E+09	100.00	234.467	8.77E+08	100.00
M+4	235.478	1.03E+09	65.99	235.476	1.65E+08	18.82
M+5	236.481	1.32E+08	8.43	236.465	1.07E+07	1.22

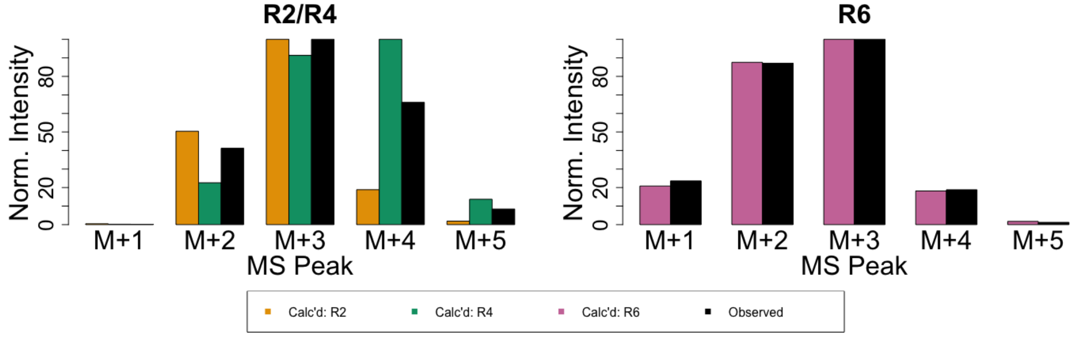

Fig. S47. Observed isotopic distributions for each of the two observed UPLC-MS distributions, with retention times noted for crosshair selections (top). Comparison of expected isotopic distributions (respective colors) versus observed isotopic distributions (black) for each of the three product regioisomers (bottom).

The first major peak (ESI+ t_R peak 1.24) can be clearly assigned as a mixture of the R2/R4 regioisomers. Deconvolution via ordinary least-squares (OLS) regression yields **R2**:**R4** ratios of **1.0**:**1.4** and **1.0**:**1.7** (**C**$_{\textbf{NMR}}$ and **C**$_{\textbf{LC}}$, respectively).

See section above for details on OLS deconvolution.

UPLC-MS Analysis, C2

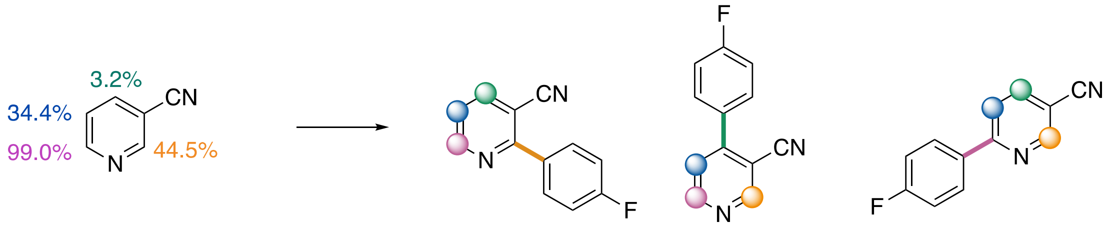

Fig. S48. Reaction **C2**, following the HTE general procedure detailed above.

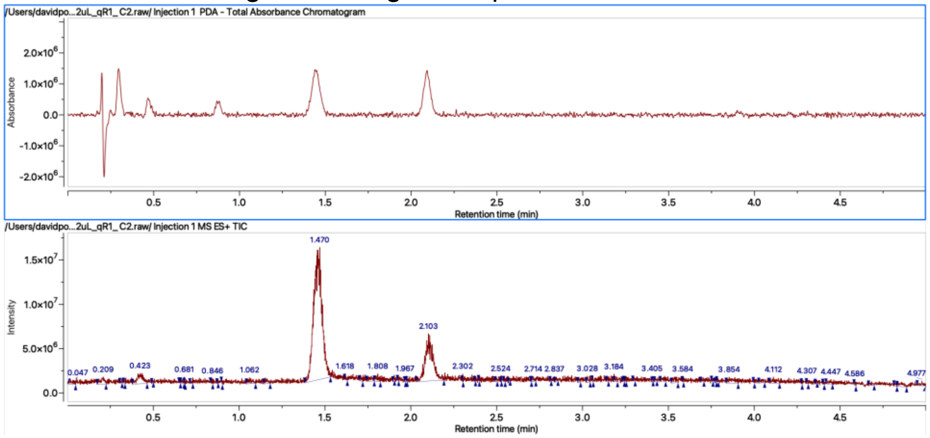

Fig. S49. Direct UPLC-MS analysis of reaction well **C2**, with *m/z* range 195-210 Da.

UPLC, C2						
		rt			rt	
	m/z	1.38..1.53	R2R4	m/z	2.03..2.19	R6
M+1	199.361	3.98E+06	0.91	199.396	6.97E+07	41.37
M+2	200.402	4.39E+08	100.00	200.386	1.68E+08	100.00
M+3	201.428	3.68E+08	83.70	201.416	5.50E+07	32.66
M+4	202.433	1.08E+08	24.49	202.422	6.09E+06	3.62
M+5	203.459	5.72E+06	1.30	203.416	2.90E+05	0.17

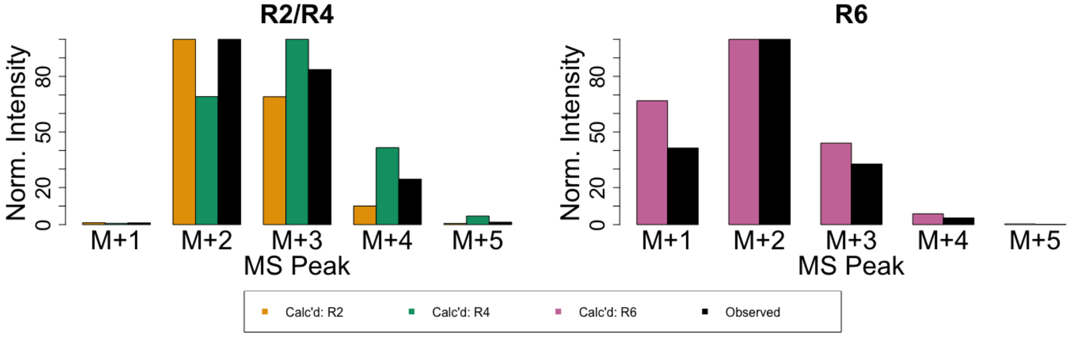

Fig. S50. Observed isotopic distributions for each of the two observed UPLC-MS distributions, with retention times noted for crosshair selections (top). Comparison of expected isotopic distributions (respective colors) versus observed isotopic distributions (black) for each of the three product regioisomers (bottom).

The first major peak (ESI+ t_R peak 1.47) can be clearly assigned as a mixture of the R2/R4 regioisomers. Deconvolution via ordinary least-squares (OLS) regression yields **R2**:**R4** ratios of **1.9**:**1.0** and **1.8**:**1.0** (**C**$_{NMR}$ and **C**$_{LC}$, respectively).

See above section for details on OLS deconvolution.

UPLC-MS Analysis, C3

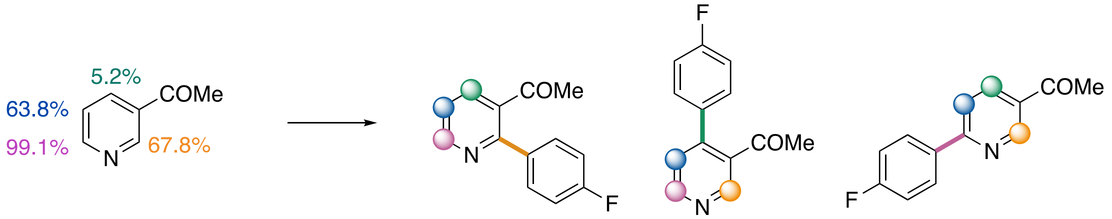

Fig. S51. Reaction **C3**, following the HTE general procedure detailed above.

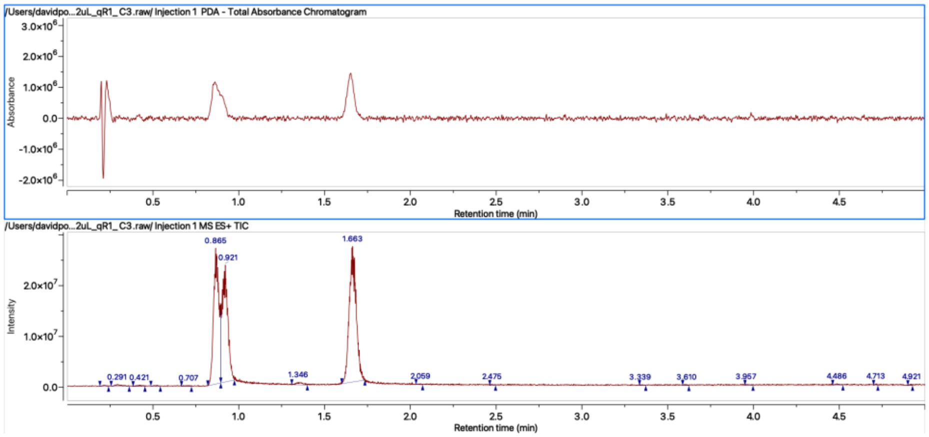

Fig. S52. Direct UPLC-MS analysis of reaction well **C3**, with *m/z* range 210-225 Da.

UPLC, C3									
		rt			rt			rt	
	m/z	0.82..0.89	R4	m/z	0.90..0.97	R2	m/z	1.60..1.74	R6
M+1	216.474	5.37E+05	0.12	216.477	1.02E+06	0.19	216.447	1.43E+08	24.23
M+2	217.465	1.03E+08	22.92	217.461	3.10E+08	58.11	217.461	5.23E+08	88.58
M+3	218.473	4.08E+08	90.74	218.471	5.34E+08	100.00	218.471	5.90E+08	100.00
M+4	219.484	4.49E+08	100.00	219.479	1.29E+08	24.15	219.473	1.10E+08	18.68
M+5	220.487	6.11E+07	13.60	220.471	1.10E+07	2.06	220.466	6.82E+06	1.16

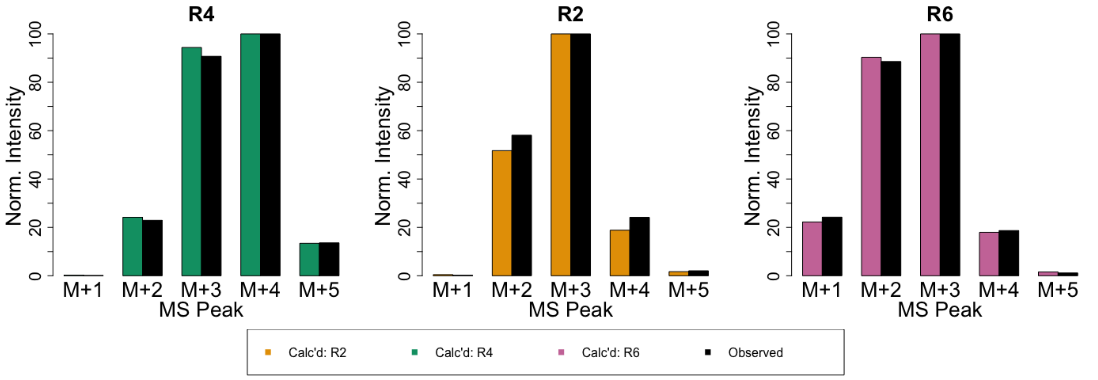

Fig. S53. Observed isotopic distributions for each of the three product regioisomers, with retention times noted for crosshair selections (top). Comparison of expected isotopic distributions (respective colors) versus observed isotopic distributions (black) for each of the three product regioisomers (bottom).

UPLC-MS Analysis, C4

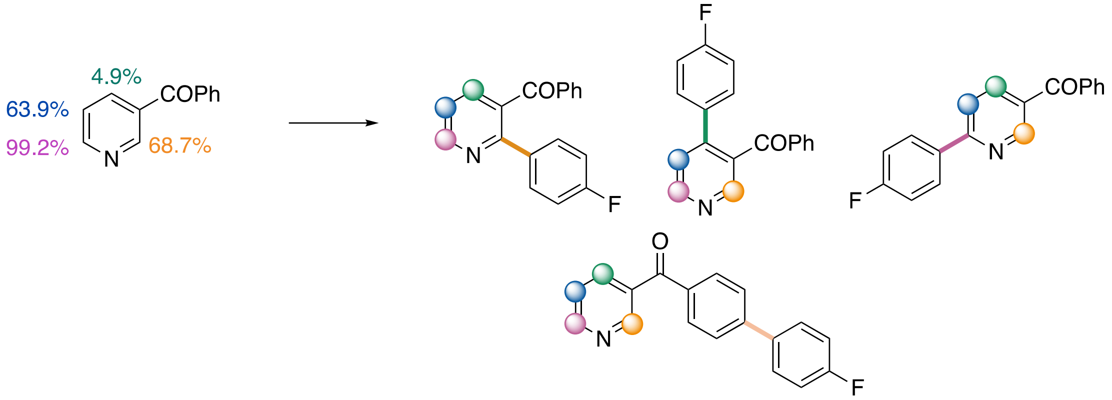

Fig. S54. Reaction **C4**, following the HTE general procedure detailed above.

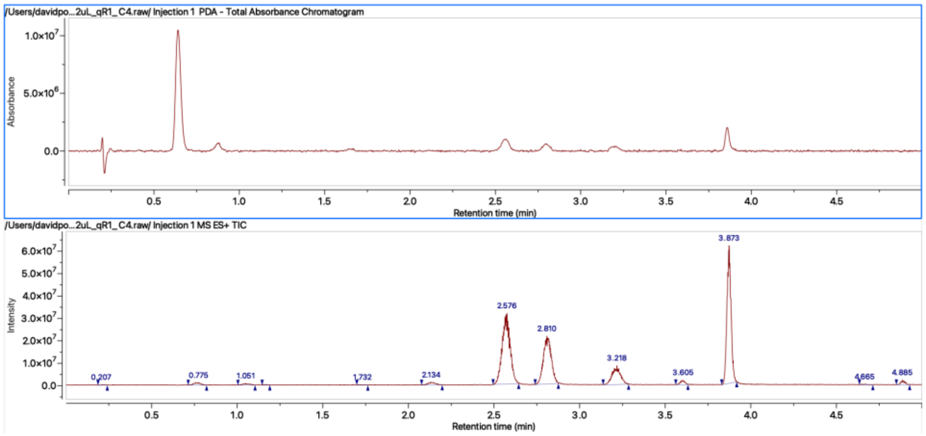

Fig. S55. Direct UPLC-MS analysis of reaction well **C4**, with *m/z* range 270-285 Da.

UPLC, C4												
		rt			rt			rt			rt	
	m/z	2.50..2.65	R4	m/z	2.74..2.88	R2	m/z	3.14..3.29	R-Ph	m/z	3.83..3.92	R6
M+1	278.493	2.20E+06	0.29	278.474	2.11E+06	0.31	278.377	3.89E+06	2.12	278.508	1.84E+08	23.50
M+2	279.514	1.73E+08	23.10	279.515	3.52E+08	52.18	279.515	4.05E+07	22.05	279.512	6.62E+08	84.48
M+3	280.522	6.51E+08	87.12	280.523	6.75E+08	100.00	280.520	1.59E+08	86.59	280.522	7.83E+08	100.00
M+4	281.534	7.48E+08	100.00	281.533	1.60E+08	23.78	281.531	1.84E+08	100.00	281.534	1.83E+08	23.38
M+5	282.541	1.43E+08	19.10	282.518	1.16E+07	1.72	282.535	3.60E+07	19.59	282.539	2.01E+07	2.57

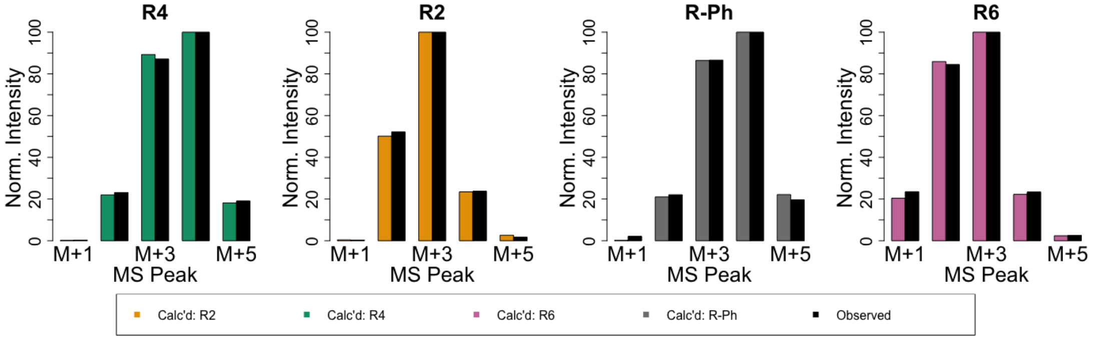

Fig. S56. Observed isotopic distributions for each of the four observed UPLC-MS distributions, with retention times noted for crosshair selections (top). Comparison of expected isotopic distributions (respective colors) versus observed isotopic distributions (black) for each of the three product regioisomers (bottom).

Given the close similarity of expected distributions for **R4** and **R-Ph**, these isomers cannot be definitively distinguished based on their isotopic distributions alone. The small relative amount of the t_R 3.22 peak makes assignment as **R-Ph** a more reasonable assumption. As above for well **A4**, the MS ion pattern of this peak does not agree well with the expected **R5** MS ion pattern.

UPLC-MS Analysis, C5

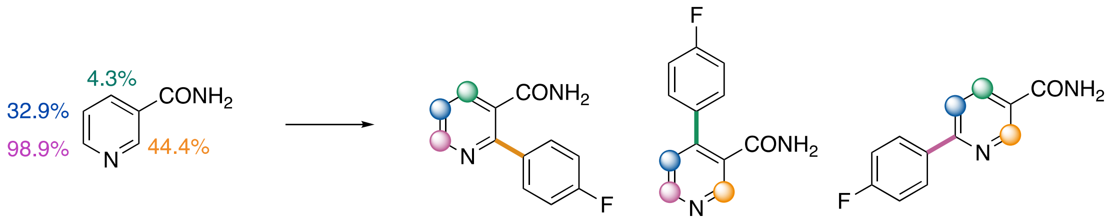

Fig. S57. Reaction **C5**, following the HTE general procedure detailed above.

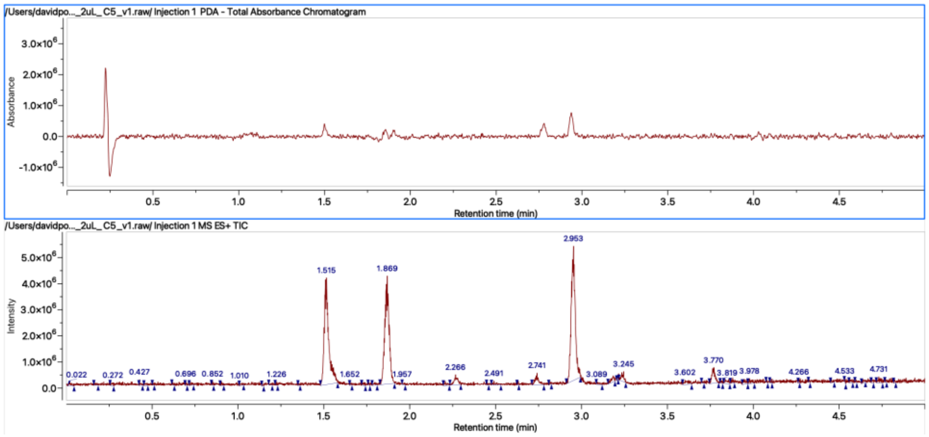

Fig. S58. Direct UPLC-MS analysis of reaction well **C5**, with *m/z* range 210-225 Da.

**UPLC, C5**									
		rt			rt			rt	
	m/z	1.48..1.58	R2	m/z	1.82..1.91	R4	m/z	2.91..3.00	R6
M+1	217.404	2.24E+05	0.37	217.543	8.73E+04	0.18	217.464	3.81E+07	64.15
M+2	218.475	6.10E+07	100.00	218.474	3.39E+07	70.72	218.468	5.93E+07	100.00
M+3	219.477	3.90E+07	63.89	219.484	4.80E+07	100.00	219.480	2.41E+07	40.67
M+4	220.465	2.66E+06	4.37	220.490	1.84E+07	38.30	220.475	2.56E+06	4.31
M+5	221.452	1.50E+05	0.25	221.471	6.74E+05	1.40	221.504	4.02E+05	0.68

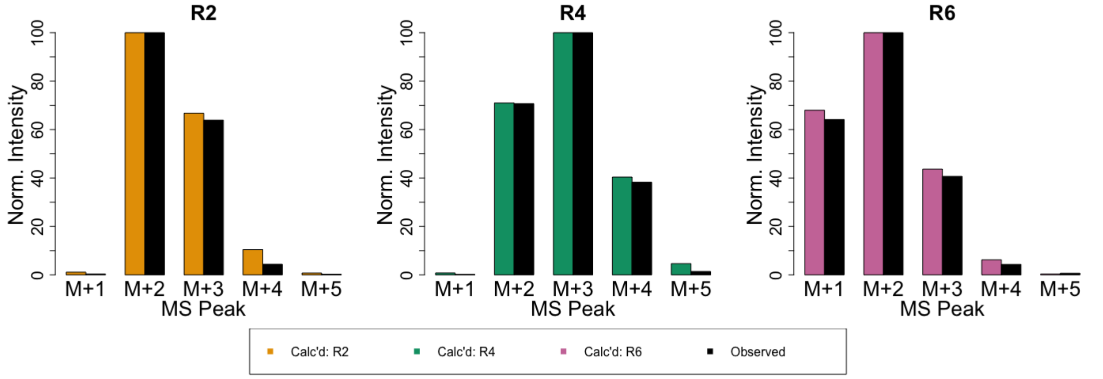

Fig. S59. Observed isotopic distributions for each of the three product regioisomers, with retention times noted for crosshair selections (top). Comparison of expected isotopic distributions (respective colors) versus observed isotopic distributions (black) for each of the three product regioisomers (bottom).

UPLC-MS Analysis, C6

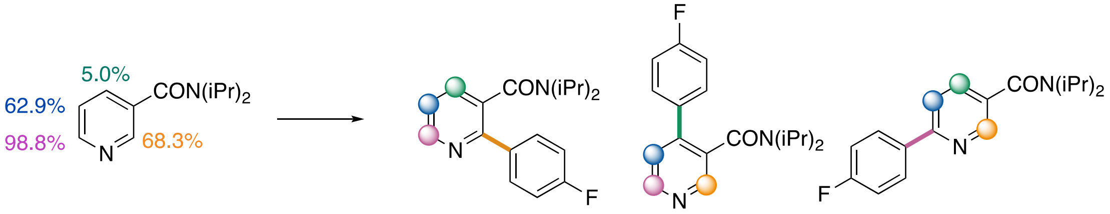

Fig. S60. Reaction **C6**, following the HTE general procedure detailed above.

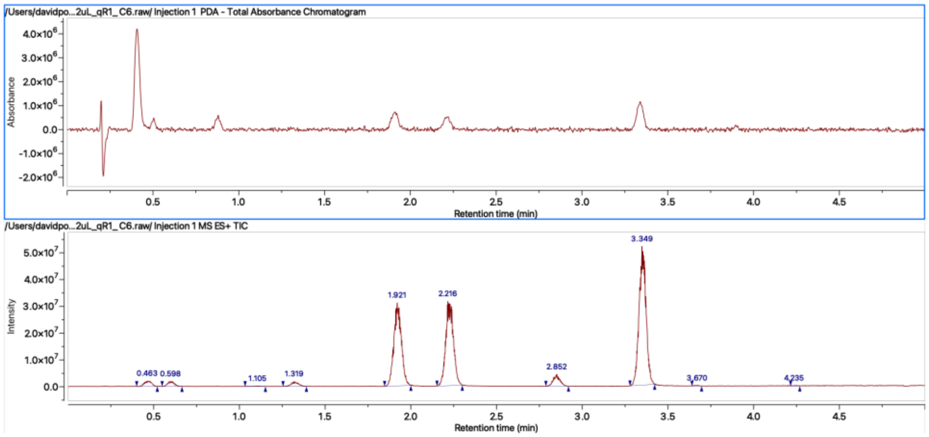

Fig. S61. Direct UPLC-MS analysis of reaction well **C6**, with *m/z* range 295-310 Da.

**UPLC, C6**									
		rt			rt			rt	
	m/z	1.85..2.00	R4	m/z	2.15..2.29	R2	m/z	3.28..3.42	R6
M+1	301.566	5.28E+05	0.08	301.554	1.60E+06	0.17	301.590	2.44E+08	22.40
M+2	302.598	1.63E+08	23.60	302.596	4.79E+08	51.43	302.595	9.35E+08	85.70
M+3	303.606	6.12E+08	88.70	303.606	9.31E+08	100.00	303.606	1.09E+09	100.00
M+4	304.616	6.90E+08	100.00	304.612	2.24E+08	24.04	304.613	2.54E+08	23.30
M+5	305.622	1.24E+08	17.89	305.620	2.12E+07	2.28	305.619	2.60E+07	2.38

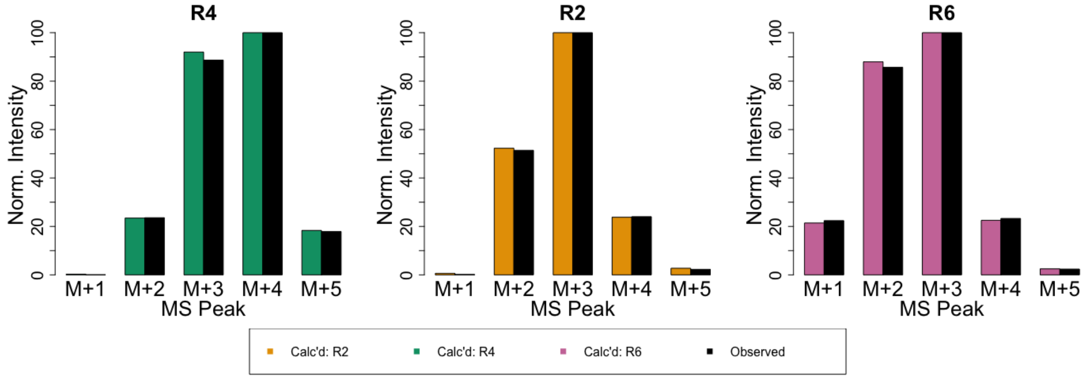

Fig. S62. Observed isotopic distributions for each of the three product regioisomers, with retention times noted for crosshair selections (top). Comparison of expected isotopic distributions (respective colors) versus observed isotopic distributions (black) for each of the three product regioisomers (bottom).

UPLC-MS Analysis, D1

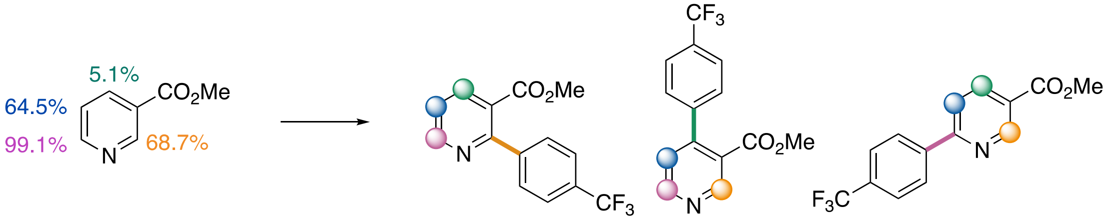

Fig. S63. Reaction **D1**, following the HTE general procedure detailed above.

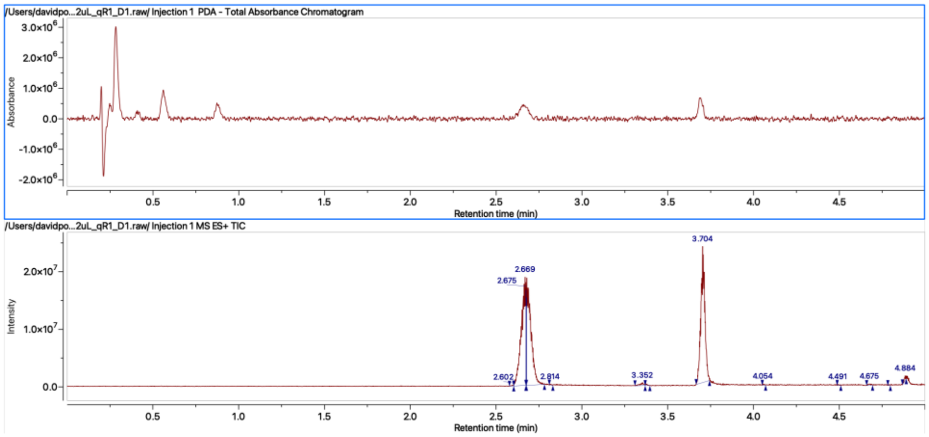

Fig. S64. Direct UPLC-MS analysis of reaction well **D1**, with *m/z* range 280-295 Da.

UPLC, D1						
	m/z	2.60..2.79	R2R4	m/z	3.66..3.75	R6
M+1	282.478	7.82E+05	0.14	282.468	6.64E+07	22.60
M+2	283.478	2.13E+08	39.09	283.481	2.54E+08	86.59
M+3	284.486	5.46E+08	100.00	284.485	2.94E+08	100.00
M+4	285.498	3.51E+08	64.42	285.492	5.34E+07	18.18
M+5	286.492	4.10E+07	7.52	286.453	3.46E+06	1.18

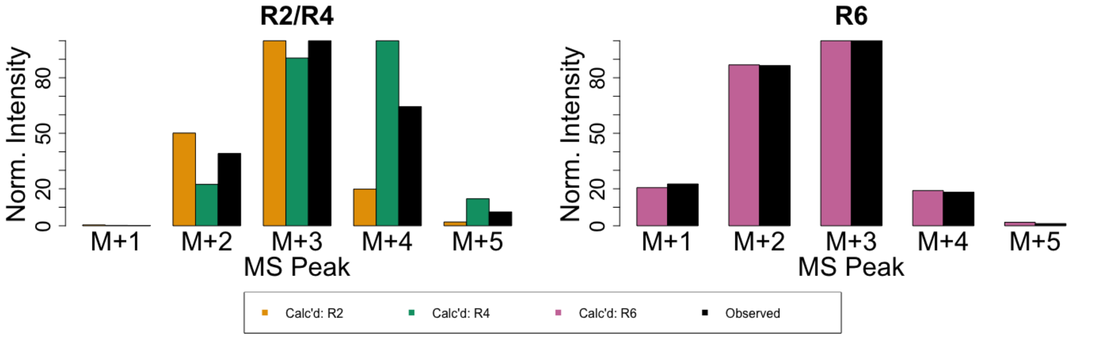

Fig. S65. Observed isotopic distributions for each of the two observed UPLC-MS distributions, with retention times noted for crosshair selections (top). Comparison of expected isotopic distributions (respective colors) versus observed isotopic distributions (black) for each of the three product regioisomers (bottom).

The first major peak (ESI+ t_R peak 2.67) can be clearly assigned as a mixture of the R2/R4 regioisomers. Deconvolution via ordinary least-squares (OLS) regression yields R2:R4 ratios of 1.0:1.4 and 1.0:1.6 (**C**$_{NMR}$ and **C**$_{LC}$, respectively).

See above section for details on OLS deconvolution.

UPLC-MS Analysis, D2

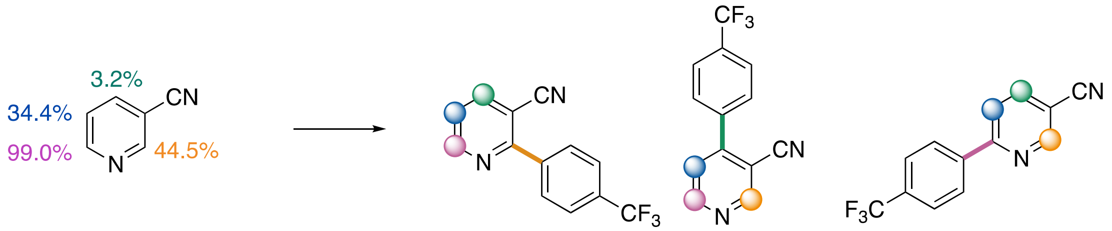

Fig. S66. Reaction **D2**, following the HTE general procedure detailed above.

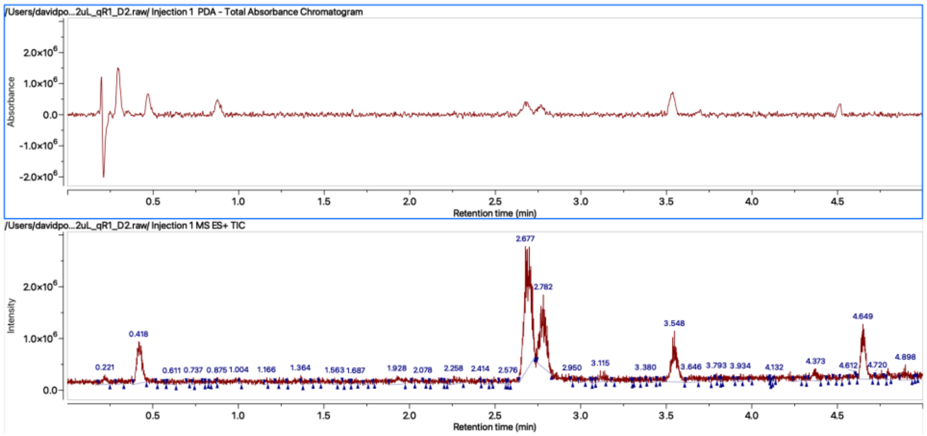

Fig. S67. Direct UPLC-MS analysis of reaction well **D2**, with *m/z* range 245-260 Da.

Due to the low intensity of all ESI+ peaks for this well, it was not feasible to obtain isotopic distributions from the separated peaks. To obtain product ratios of unseparated mixtures using experimentally observed LC values (C_{LC}) isotopic distributions obtained from A2 were used.

UPLC-MS Analysis, D3

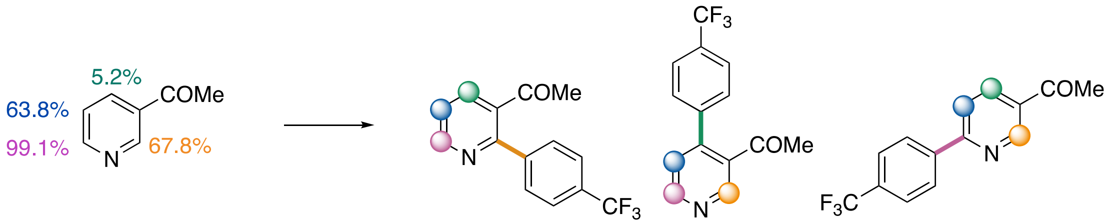

Fig. S68. Reaction **D3**, following the HTE general procedure detailed above.

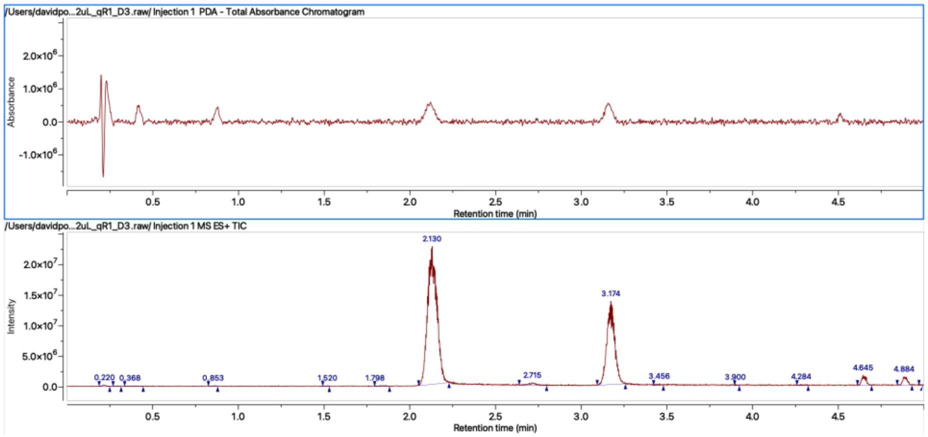

Fig. S69. Direct UPLC-MS analysis of reaction well **D3**, with *m/z* range 260-275 Da.

UPLC, D3		rt			rt	
	m/z	2.05..2.23	R2R4	m/z	3.09..3.26	R6
M+1	266.348	7.45E+05	0.11	266.478	6.62E+07	21.30
M+2	267.486	2.74E+08	39.69	267.486	2.72E+08	87.54
M+3	268.495	6.91E+08	100	268.493	3.11E+08	100.00
M+4	269.502	4.42E+08	63.95	269.500	5.51E+07	17.75
M+5	270.509	5.51E+07	7.98	270.484	3.21E+06	1.03

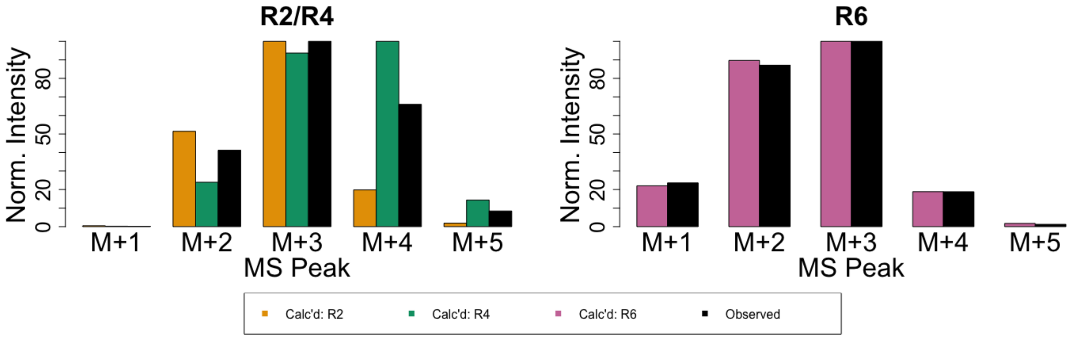

Fig. S70. Observed isotopic distributions for each of the two observed UPLC-MS distributions, with retention times noted for crosshair selections (top). Comparison of expected isotopic distributions (respective colors) versus observed isotopic distributions (black) for each of the three product regioisomers (bottom).

The first major peak (ESI+ t_R peak 2.13) can be clearly assigned as a mixture of the R2/R4 regioisomers. Deconvolution via ordinary least-squares (OLS) regression yields **R2**:**R4** ratios of **1.0**:**1.4** and **1.0**:**1.6** (**C**$_{\mathbf{NMR}}$ and **C**$_{\mathbf{LC}}$, respectively).

See above section for details on OLS deconvolution.

UPLC-MS Analysis, D4

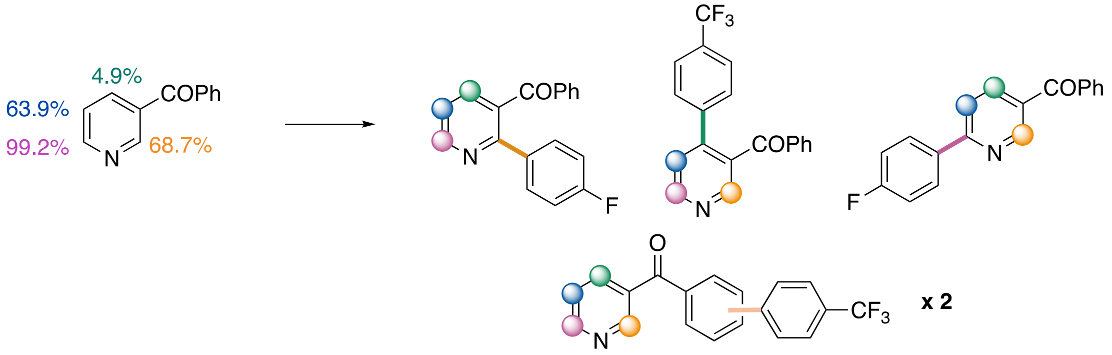

Fig. S71. Reaction **D4**, following the HTE general procedure detailed above.

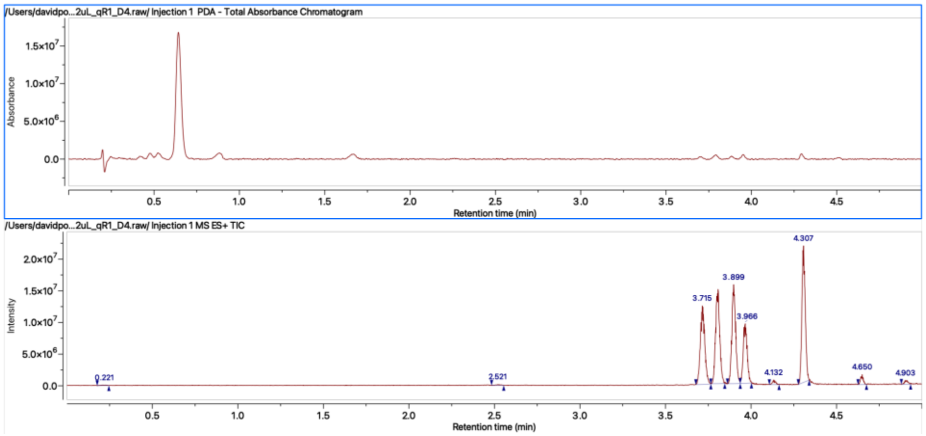

Fig. S72. Direct UPLC-MS analysis of reaction well **D4**, with *m/z* range 325-340 Da.

UPLC, D4		rt			rt			rt			rt			rt	
	m/z	3.68..3.77	R-Ph1	m/z	3.77..3.85	R4	m/z	3.86..3.94	R2	m/z	3.94..4.00	R-Ph2	m/z	4.28..4.34	R6
M+1	328.531	2.50E+05	0.16	328.502	2.27E+05	0.13	328.506	2.65E+05	0.11	328.489	2.30E+05	0.22	328.524	4.90E+07	22.42
M+2	329.541	3.51E+07	22.69	329.537	4.22E+07	23.47	329.537	1.19E+08	50.24	329.538	2.31E+07	22.26	329.535	1.85E+08	84.60
M+3	330.548	1.35E+08	87.05	330.549	1.64E+08	91.47	330.549	2.37E+08	100.00	330.545	9.30E+07	89.62	330.548	2.19E+08	100.00
M+4	331.558	1.55E+08	100.00	331.555	1.80E+08	100.00	331.553	5.80E+07	24.46	331.556	1.04E+08	100.00	331.551	5.13E+07	23.45
M+5	332.553	3.14E+07	20.34	332.560	3.34E+07	18.61	332.569	4.42E+06	1.86	332.564	2.32E+07	22.36	332.544	3.96E+06	1.81

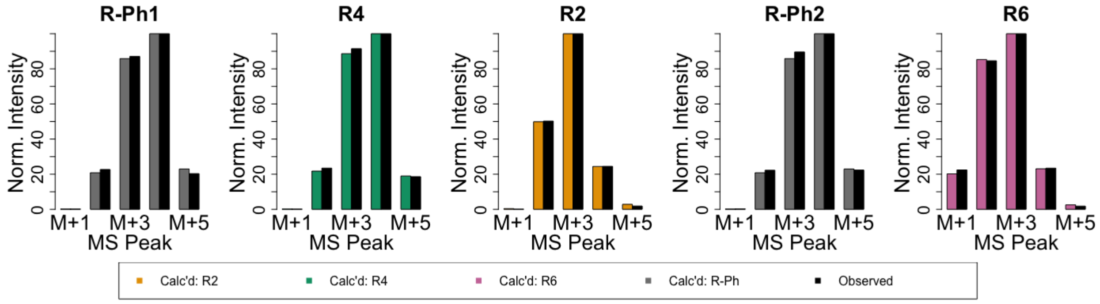

Fig. S73. Observed isotopic distributions for each of the five observed UPLC-MS distributions, with retention times noted for crosshair selections (top). Comparison of expected isotopic distributions (respective colors) versus observed isotopic distributions (black) for each of the three product regioisomers (bottom).

UPLC-MS Analysis, D5

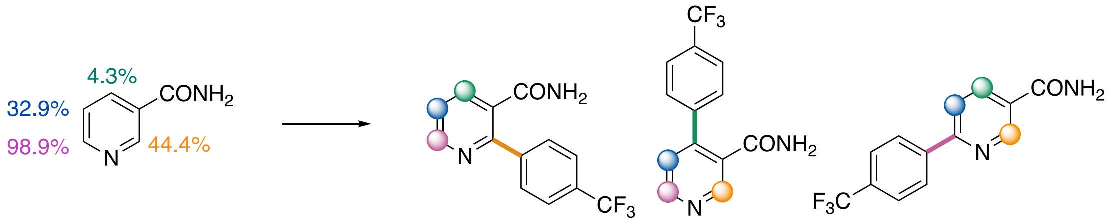

Fig. S74. Reaction **D5**, following the HTE general procedure detailed above.

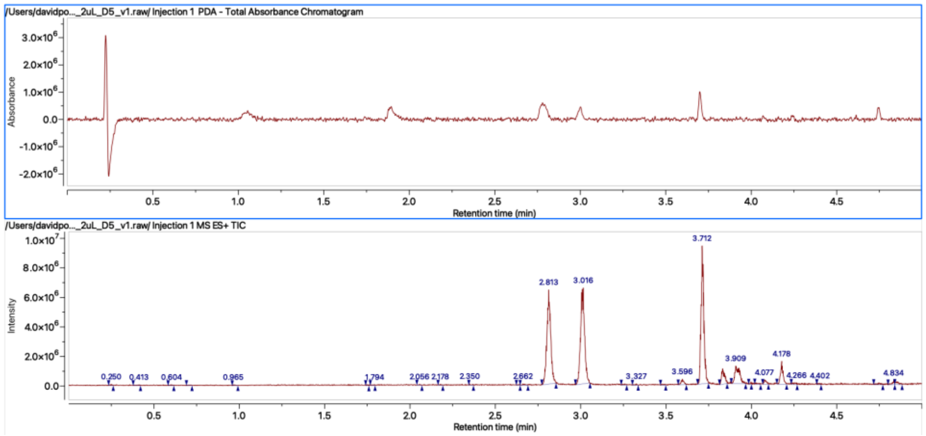

Fig. S75. Direct UPLC-MS analysis of reaction well **D5**, with *m/z* range 260-275 Da.

UPLC, D5									
		rt			rt			rt	
	m/z	2.77..2.85	R2	m/z	2.97..3.06	R4	m/z	3.68..3.75	R6
M+1	267.494	2.01E+05	0.23	267.417	2.72E+05	0.35	267.482	5.22E+07	67.82
M+2	268.489	8.94E+07	100.00	268.490	5.77E+07	74.19	268.489	7.69E+07	100.00
M+3	269.494	5.80E+07	64.91	269.495	7.78E+07	100.00	269.494	3.24E+07	42.13
M+4	270.469	5.16E+06	5.77	270.504	3.07E+07	39.41	270.514	3.23E+06	4.20
M+5	271.519	2.65E+05	0.30	271.467	1.80E+06	2.32	271.566	2.38E+05	0.31

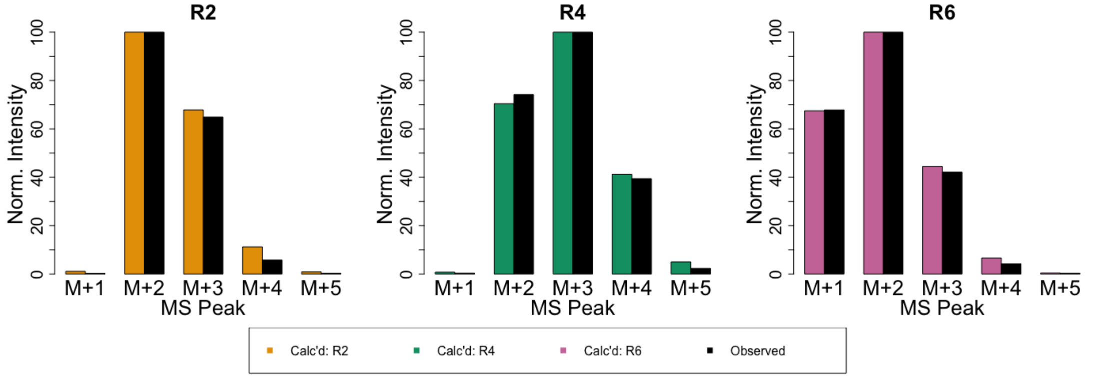

Fig. S76. Observed isotopic distributions for each of the three product regioisomers, with retention times noted for crosshair selections (top). Comparison of expected isotopic distributions (respective colors) versus observed isotopic distributions (black) for each of the three product regioisomers (bottom).

UPLC-MS Analysis, D6

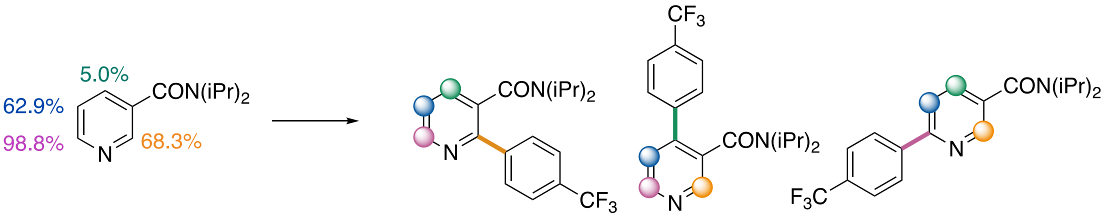

Fig. S77. Reaction **D6**, following the HTE general procedure detailed above.

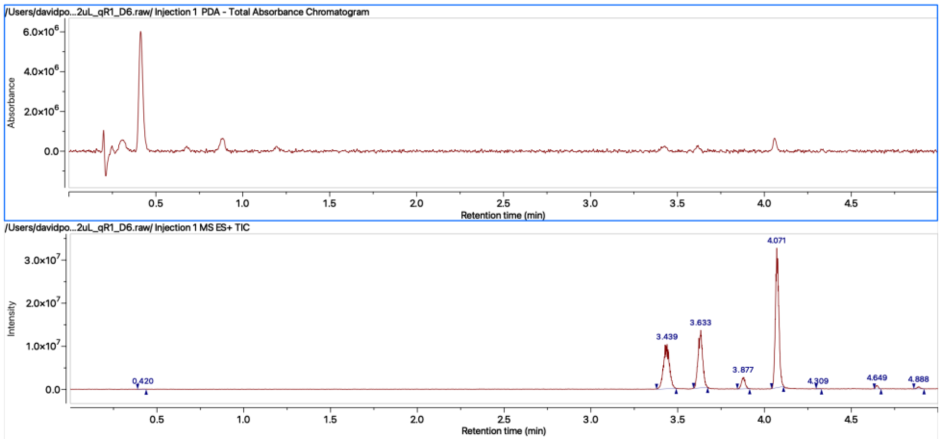

Fig. S78. Direct UPLC-MS analysis of reaction well **D6**, with *m/z* range 345-360 Da.

UPLC, D6									
		rt			rt			rt	
	m/z	3.38..3.49	R4	m/z	3.59..3.67	R2	m/z	4.04..4.11	R6
M+1	351.644	8.84E+04	0.05	351.663	3.90E+05	0.17	351.627	7.42E+07	22.04
M+2	352.637	3.82E+07	22.91	352.636	1.19E+08	50.56	352.634	2.85E+08	84.57
M+3	353.647	1.47E+08	88.35	353.649	2.35E+08	100.00	353.643	3.37E+08	100.00
M+4	354.656	1.67E+08	100.00	354.654	5.61E+07	23.89	354.645	8.02E+07	23.84
M+5	355.649	2.67E+07	16.02	355.670	4.34E+06	1.85	355.670	7.56E+06	2.25

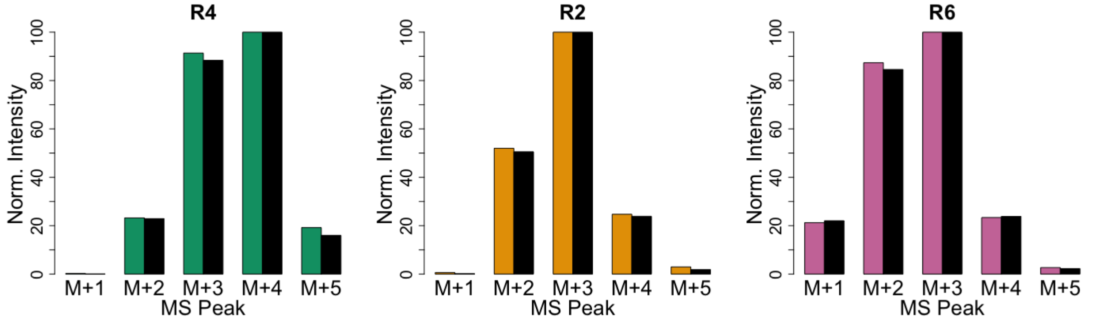

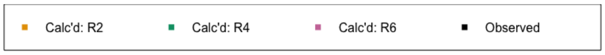

Fig. S79. Observed isotopic distributions for each of the three product regioisomers, with retention times noted for crosshair selections (top). Comparison of expected isotopic distributions (respective colors) versus observed isotopic distributions (black) for each of the three product regioisomers (bottom).

5.2.3 MS-Only Analysis of Regioselectivity for the 24-Well Plate

5.2.3.1 Description of MS-Only Analytical Methods

UPLC (Acquity SQ): Column bypass; 0.25 mL/min. Inlet: 0.5 min at 50% MeCN/H_2O (0.1% formic acid additive). Detection: UV-Vis (TWC 210 – 400 nm); MS (ESI+, *m/z* = ***varied***, 30 V, 20 Hz).

Injection volume and sample loading were varied to achieve a desired ESI+ maximum TIC height in the range of 1E7 to 1.5E8 where possible. Below 1E7, signal-to-noise became suboptimal, whereas above 1.5E8 detector overload impeded quantitation. Injection volume is noted in the screenshots directly.

Loop injections were performed initially for all wells with *m/z* range 175-375 Da to observe all monosubstituted product clusters. In some cases, minor isotopic clusters corresponding to starting material and/or diarylated products were visible.

After the initial round of observational MS-only analysis, custom *m/z* ranges for each well were set with spans of 15 Da to capture monosubstituted product clusters with additional signal-to-noise improvements. These runs were performed in triplicate to benchmark precision. Normalized intensity measurements were highly reproducible from injection to injection for each well, as discussed below. Injection volumes were varied between reaction wells to compensate for variable analyte concentrations and ionization efficiencies and are noted run-by-run.

Averages and standard deviations (SDs) were calculated from the normalized MS intensities at each of five respective *m/z* values, as well as relative standard deviations (RSDs) defined as (SD / mean). By convention, the SDs and RSDs for base peaks are thus calculated as 0.00 and 0.0%, respectively. Peak total ion count (TIC) height values were also recorded for each run with corresponding RSD calculations.

Only the first of three MS-only chromatograms are displayed for each of the 24 reaction wells. These normalized intensities directly correspond to “R1” (“Run 1”) in each of the summary tables.

5.2.3.2 Selected MS-ONLY Data for 24-Well Plate

MS-only, A1

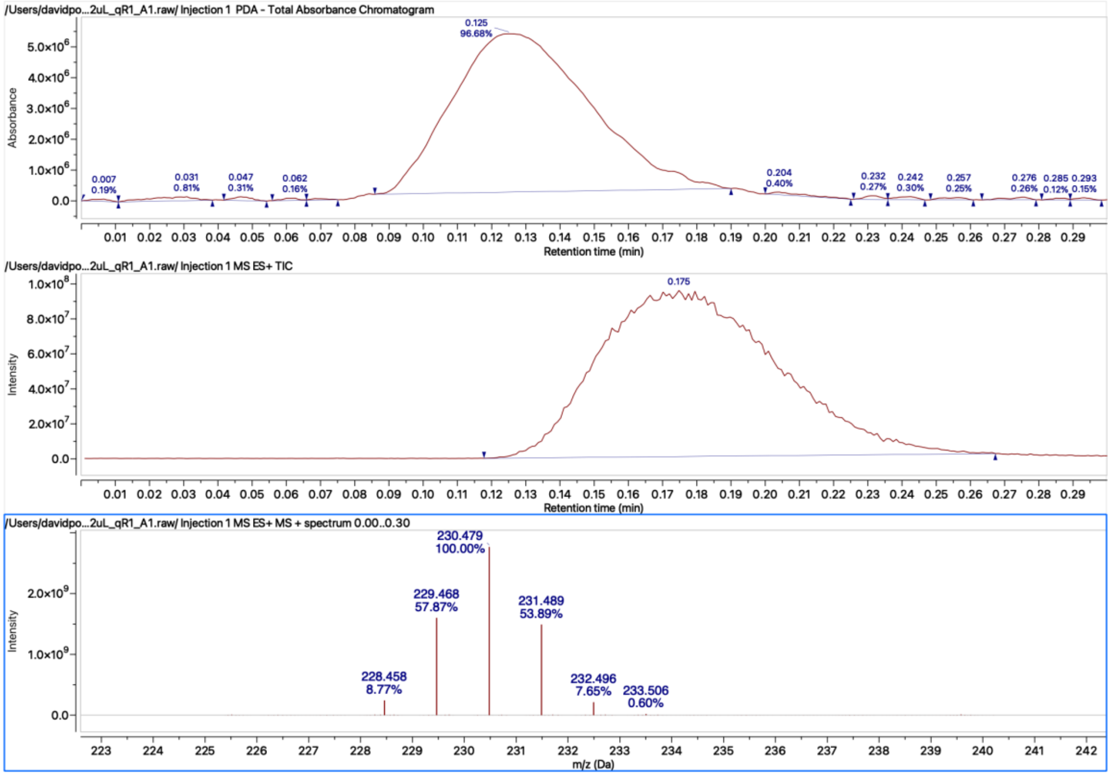

Fig. S80. *m/z* 225-240 Da used in MS file; 0.2 µL injection volume.

	B	C	D	E	F	G	H	I	J	K	L	M	N	O	P	Q
4	A1	R1	IV 0.2 µL		A1	R2	IV 0.2 µL		A1	R3	IV 0.2 µL		A1			
5	*monosub*	m/z	norm		*monosub*	m/z	norm		*monosub*	m/z	norm			AVG	SD	RSD
6	M+1	228.48	8.77		M+1	228.48	8.82		M+1	228.48	8.91		M+1	8.83	0.07	0.8%
7	M+2	229.46	57.87		M+2	229.46	57.57		M+2	229.46	57.84		M+2	57.76	0.17	0.3%
8	M+3	230.46	100.00		M+3	230.46	100.00		M+3	230.46	100.00		M+3	100.00	0.00	0.0%
9	M+4	231.46	53.89		M+4	231.46	54.07		M+4	231.46	54.30		M+4	54.09	0.21	0.4%
10	M+5	232.48	7.65		M+5	232.48	7.60		M+5	232.48	7.74		M+5	7.66	0.07	0.9%
11	*TIC height*	9.49E+07			*TIC height*	1.03E+08			*TIC height*	9.63E+07			*TIC height*	9.81E+07	4.33E+06	4.4%

Fig. S81. Numerical summary of triplicate runs for **A1**.

MS-only, A2

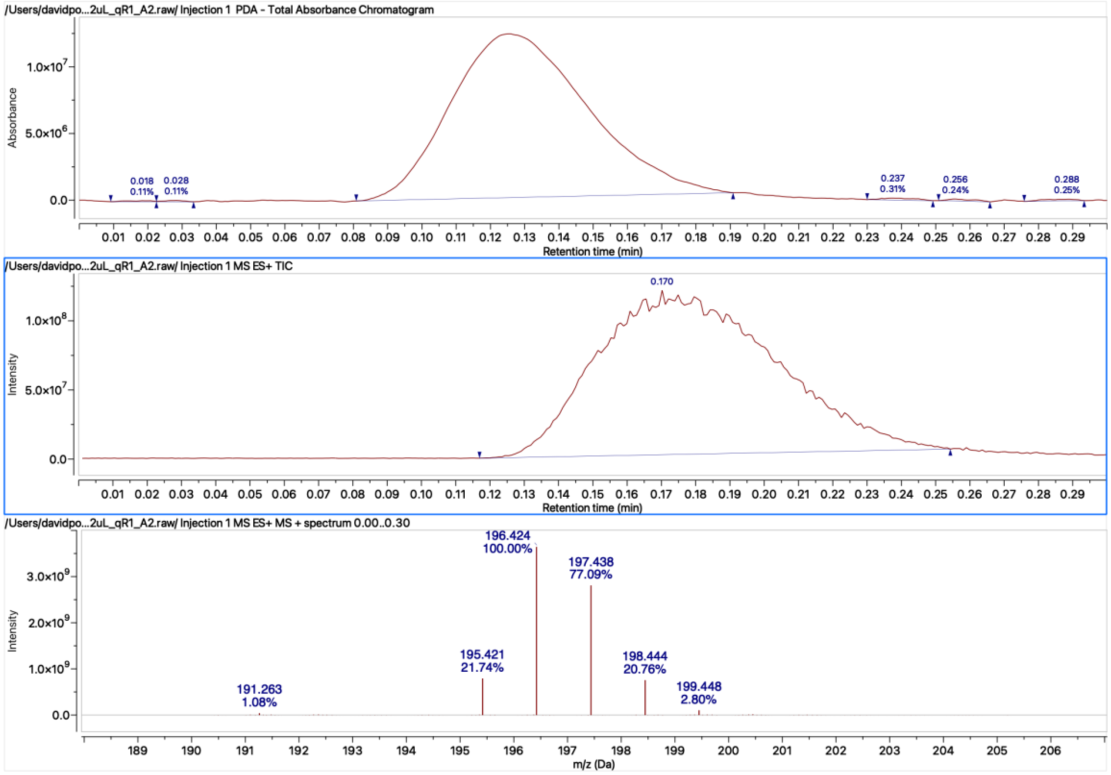

Fig. S82. *m/z* 190-205 Da used in MS file.

A2	R1	IV 0.2 μL
monosub	m/z	norm
M+1	195.38	21.74
M+2	196.39	100.00
M+3	197.39	77.09
M+4	198.39	20.76
M+5	199.43	2.80
TIC height	1.19E+08	

A2	R2	IV 0.2 μL
monosub	m/z	norm
M+1	195.38	21.34
M+2	196.39	100.00
M+3	197.39	77.21
M+4	198.39	20.08
M+5	199.43	2.71
TIC height	8.54E+07	

A2	R3	IV 0.2 μL
monosub	m/z	norm
M+1	195.38	21.57
M+2	196.39	100.00
M+3	197.39	77.33
M+4	198.39	20.18
M+5	199.43	2.83
TIC height	8.14E+07	

A2			
	AVG	SD	RSD
M+1	21.55	0.20	0.9%
M+2	100.00	0.00	0.0%
M+3	77.21	0.12	0.2%
M+4	20.34	0.37	1.8%
M+5	2.78	0.06	2.2%
TIC height	9.53E+07	2.07E+07	21.7%

Fig. S83. Numerical summary of triplicate runs for **A2**.

MS-only, A3

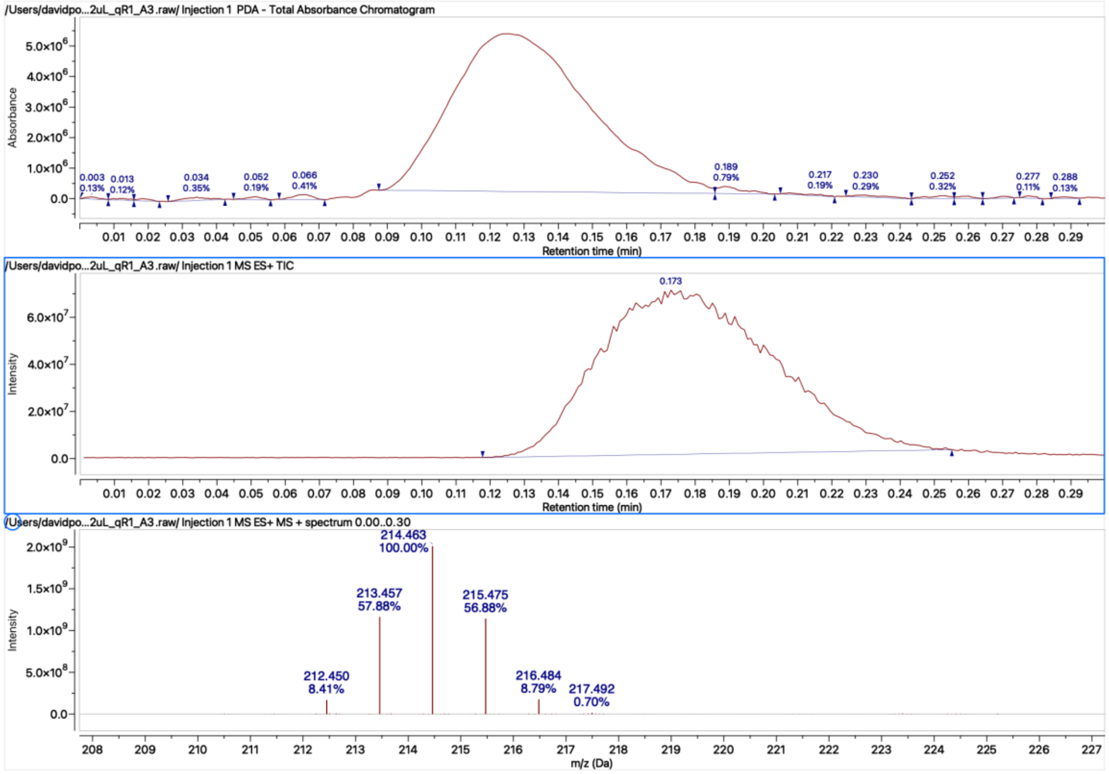

Fig. S84. *m/z* 210-225 Da used in MS file.

A3	R1	IV 0.2 μL
monosub	m/z	norm
M+1	212.44	8.41
M+2	213.44	57.88
M+3	214.44	100.00
M+4	215.45	56.88
M+5	216.48	8.79
TIC height	6.99E+07	

A3	R2	IV 0.2 μL
monosub	m/z	norm
M+1	212.44	8.39
M+2	213.44	57.44
M+3	214.44	100.00
M+4	215.45	57.55
M+5	216.48	8.94
TIC height	7.57E+07	

A3	R3	IV 0.2 μL
monosub	m/z	norm
M+1	212.44	8.57
M+2	213.44	57.92
M+3	214.44	100.00
M+4	215.45	57.37
M+5	216.48	8.91
TIC height	7.35E+07	

A3			
	AVG	SD	RSD
M+1	8.46	0.10	1.2%
M+2	57.75	0.27	0.5%
M+3	100.00	0.00	0.0%
M+4	57.27	0.35	0.6%
M+5	8.88	0.08	0.9%
TIC height	7.30E+07	2.93E+06	4.0%

Fig. S85. Numerical summary of triplicate runs for **A3**.

MS-only, A4

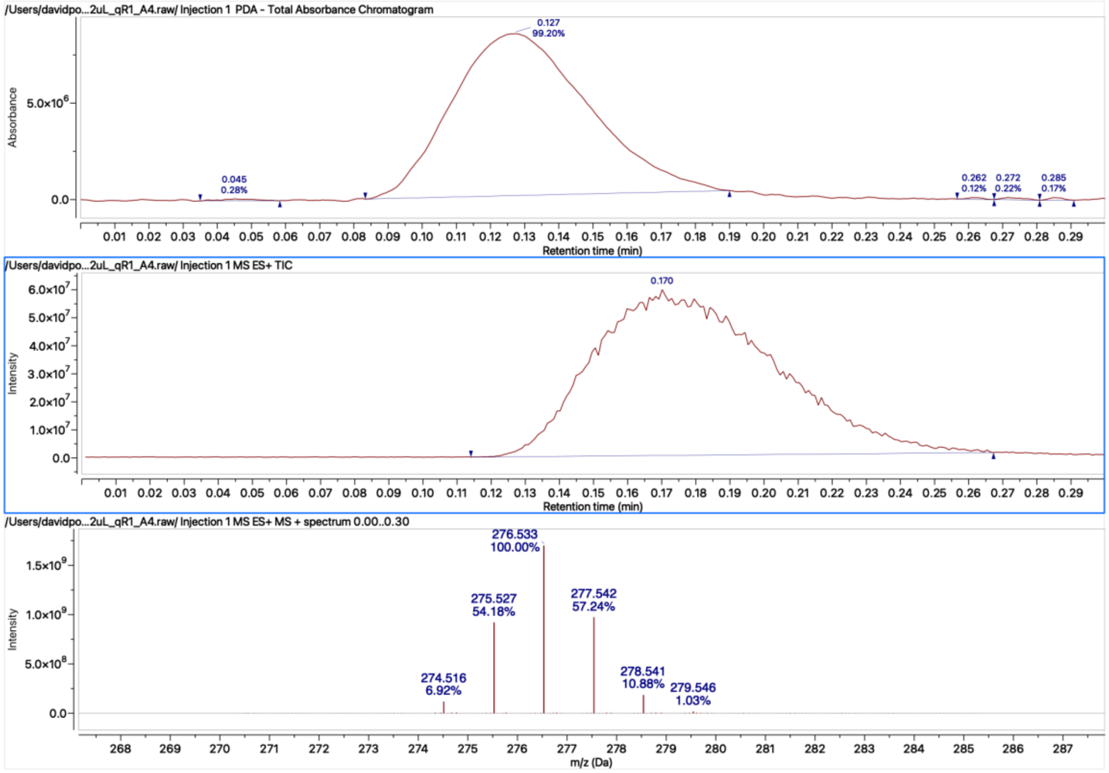

Fig. S86. *m/z* 270-285 Da used in MS file.

A4	R1	IV 0.2 μL
monosub	m/z	norm
M+1	274.48	6.92
M+2	275.51	54.18
M+3	276.51	100.00
M+4	277.52	57.24
M+5	278.51	10.88
TIC height	5.92E+07	

A4	R2	IV 0.2 μL
monosub	m/z	norm
M+1	274.48	7.13
M+2	275.51	53.55
M+3	276.51	100.00
M+4	277.52	57.50
M+5	278.51	10.89
TIC height	6.07E+07	

A4	R3	IV 0.2 μL
monosub	m/z	norm
M+1	274.48	7.12
M+2	275.51	53.95
M+3	276.51	100.00
M+4	277.52	57.46
M+5	278.51	10.60
TIC height	7.35E+07	

A4			
	AVG	SD	RSD
M+1	7.06	0.12	1.7%
M+2	53.89	0.32	0.6%
M+3	100.00	0.00	0.0%
M+4	57.40	0.14	0.2%
M+5	10.79	0.16	1.5%
TIC height	6.45E+07	7.86E+06	12.2%

Fig. S87. Numerical summary of triplicate runs for **A4**.

MS-only, A5

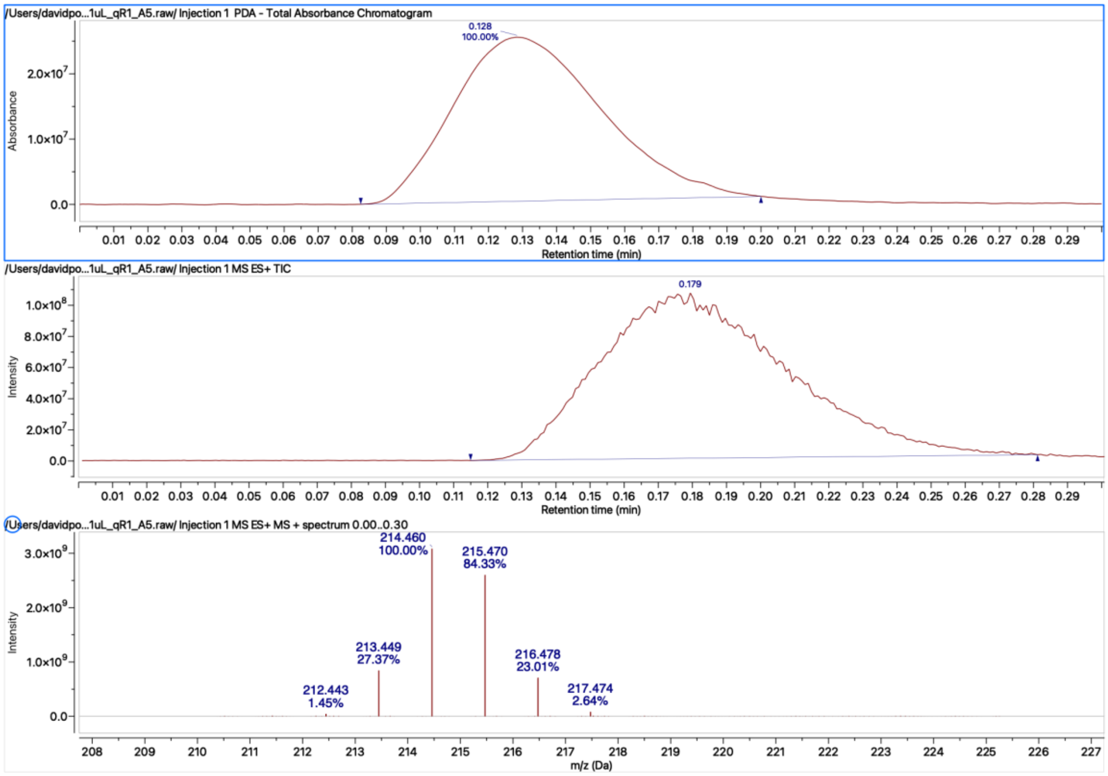

Fig. S88. *m/z* 210-225 Da used in MS file.

A5	R1	IV 1.0 μL
monosub	m/z	norm
M+1	213.44	27.37
M+2	214.45	100.00
M+3	215.44	84.33
M+4	216.44	23.01
M+5	218.47	2.64
TIC height	1.06E+08	

A5	R2	IV 1.0 μL
monosub	m/z	norm
M+1	213.44	27.29
M+2	214.45	100.00
M+3	215.44	85.35
M+4	216.44	23.09
M+5	218.47	2.88
TIC height	1.06E+08	

A5	R3	IV 1.0 μL
monosub	m/z	norm
M+1	213.44	27.54
M+2	214.45	100.00
M+3	215.44	85.21
M+4	216.44	23.19
M+5	218.47	2.72
TIC height	1.03E+08	

A5			
	AVG	SD	RSD
M+1	27.40	0.13	0.5%
M+2	100.00	0.00	0.0%
M+3	84.96	0.55	0.7%
M+4	23.10	0.09	0.4%
M+5	2.75	0.12	4.4%
TIC height	1.05E+08	1.73E+06	1.6%

Fig. S89. Numerical summary of triplicate runs for **A5**.

MS-only, A6

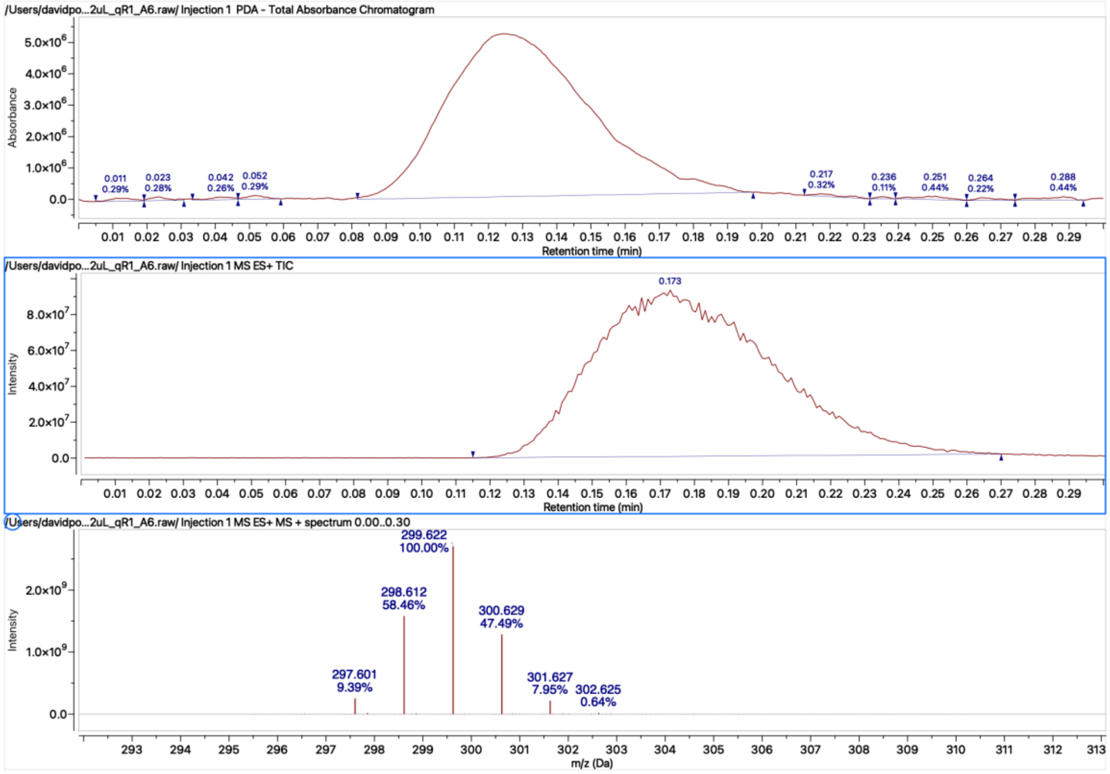

Fig. S90. *m/z* 295-310 Da used in MS file.

A6	R1	IV 0.2 μL
monosub	m/z	norm
M+1	297.57	9.39
M+2	298.58	58.46
M+3	299.58	100.00
M+4	300.57	47.49
M+5	301.60	7.95
TIC height	9.28E+07	

A6	R2	IV 0.2 μL
monosub	m/z	norm
M+1	297.57	9.41
M+2	298.58	58.41
M+3	299.58	100.00
M+4	300.57	47.38
M+5	301.60	8.08
TIC height	9.04E+07	

A6	R3	IV 0.2 μL
monosub	m/z	norm
M+1	297.57	9.44
M+2	298.58	58.24
M+3	299.58	100.00
M+4	300.57	47.60
M+5	301.60	7.95
TIC height	8.82E+07	

A6			
	AVG	SD	RSD
M+1	9.41	0.03	0.3%
M+2	58.37	0.12	0.2%
M+3	100.00	0.00	0.0%
M+4	47.49	0.11	0.2%
M+5	7.99	0.08	0.9%
TIC height	9.05E+07	2.30E+06	2.5%

Fig. S91. Numerical summary of triplicate runs for **A6**.

MS-only, B1

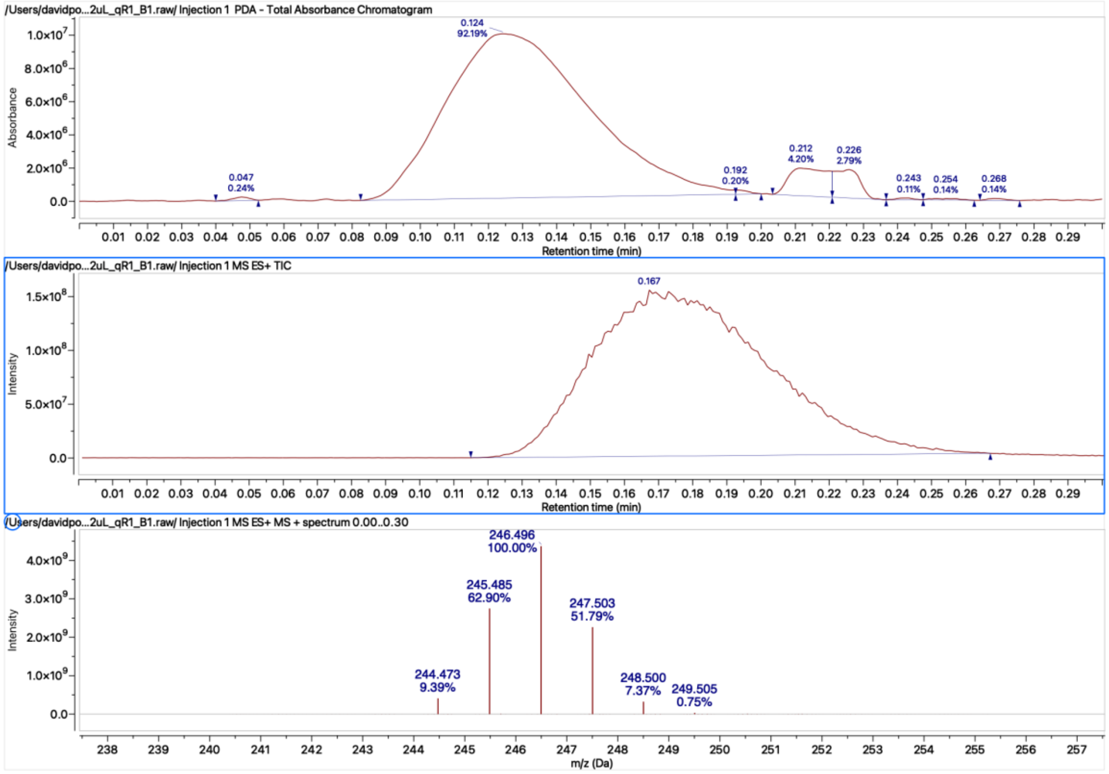

Fig. S92. *m/z* 240-255 Da used in MS file.

B1	R1	IV 0.2 μL
monosub	m/z	norm
M+1	244.45	9.39
M+2	245.47	62.90
M+3	246.47	100.00
M+4	247.47	51.79
M+5	248.49	7.37
TIC height	1.54E+08	

B1	R2	IV 0.2 μL
monosub	m/z	norm
M+1	244.45	9.37
M+2	245.47	63.07
M+3	246.47	100.00
M+4	247.47	51.76
M+5	248.49	7.33
TIC height	1.61E+08	

B1	R3	IV 0.2 μL
monosub	m/z	norm
M+1	244.45	9.36
M+2	245.47	63.59
M+3	246.47	100.00
M+4	247.47	51.84
M+5	248.49	7.38
TIC height	1.46E+08	

B1			
	AVG	SD	RSD
M+1	9.37	0.02	0.2%
M+2	63.19	0.36	0.6%
M+3	100.00	0.00	0.0%
M+4	51.80	0.04	0.1%
M+5	7.36	0.03	0.4%
TIC height	1.54E+08	7.51E+06	4.9%

Fig. S93. Numerical summary of triplicate runs for **B1**.

MS-only, B2

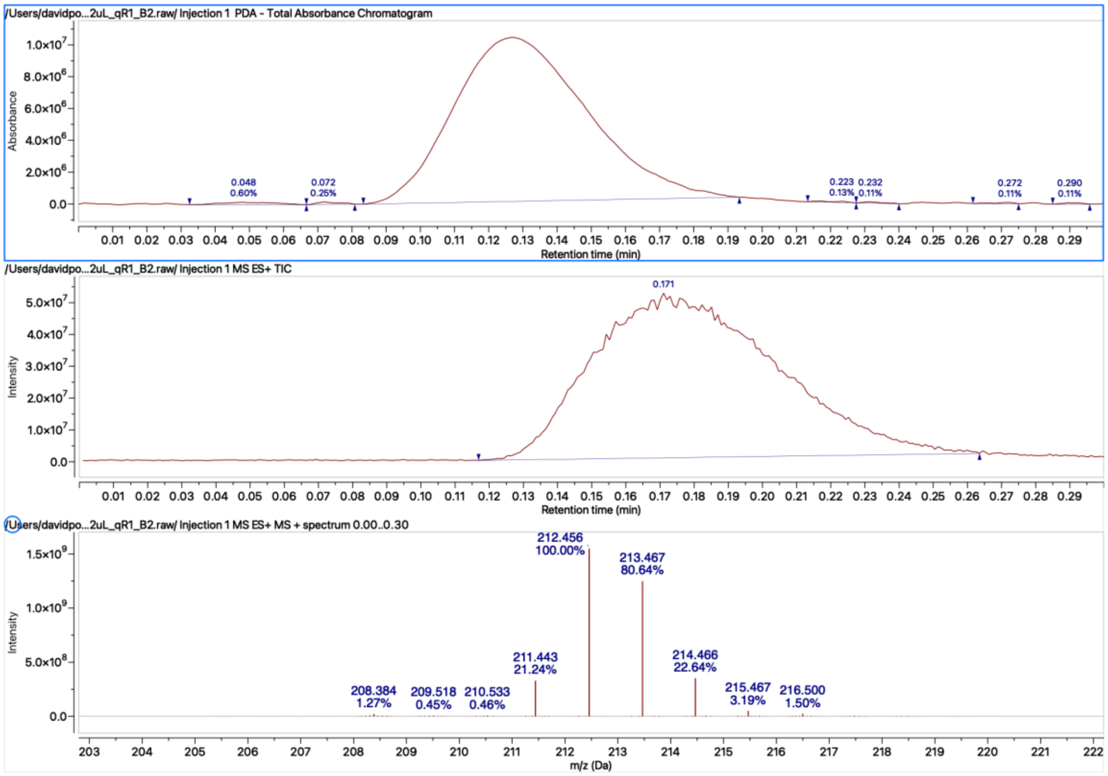

Fig. S94. *m/z* 205-220 Da used in MS file.

B2	R1	IV 0.2 μL
monosub	m/z	norm
M+1	211.41	21.24
M+2	212.41	100.00
M+3	213.43	80.64
M+4	214.44	22.64
M+5	215.45	3.19
TIC height	5.18E+07	

B2	R2	IV 0.2 μL
monosub	m/z	norm
M+1	211.41	21.23
M+2	212.41	100.00
M+3	213.43	80.11
M+4	214.44	22.62
M+5	215.45	3.21
TIC height	5.59E+07	

B2	R3	IV 0.2 μL
monosub	m/z	norm
M+1	211.41	21.33
M+2	212.41	100.00
M+3	213.43	80.94
M+4	214.44	22.66
M+5	215.45	3.20
TIC height	5.05E+07	

B2			
	AVG	SD	RSD
M+1	21.27	0.06	0.3%
M+2	100.00	0.00	0.0%
M+3	80.56	0.42	0.5%
M+4	22.64	0.02	0.1%
M+5	3.20	0.01	0.3%
TIC height	5.27E+07	2.82E+06	5.3%

Fig. S95. Numerical summary of triplicate runs for **B2**.

MS-only, B3

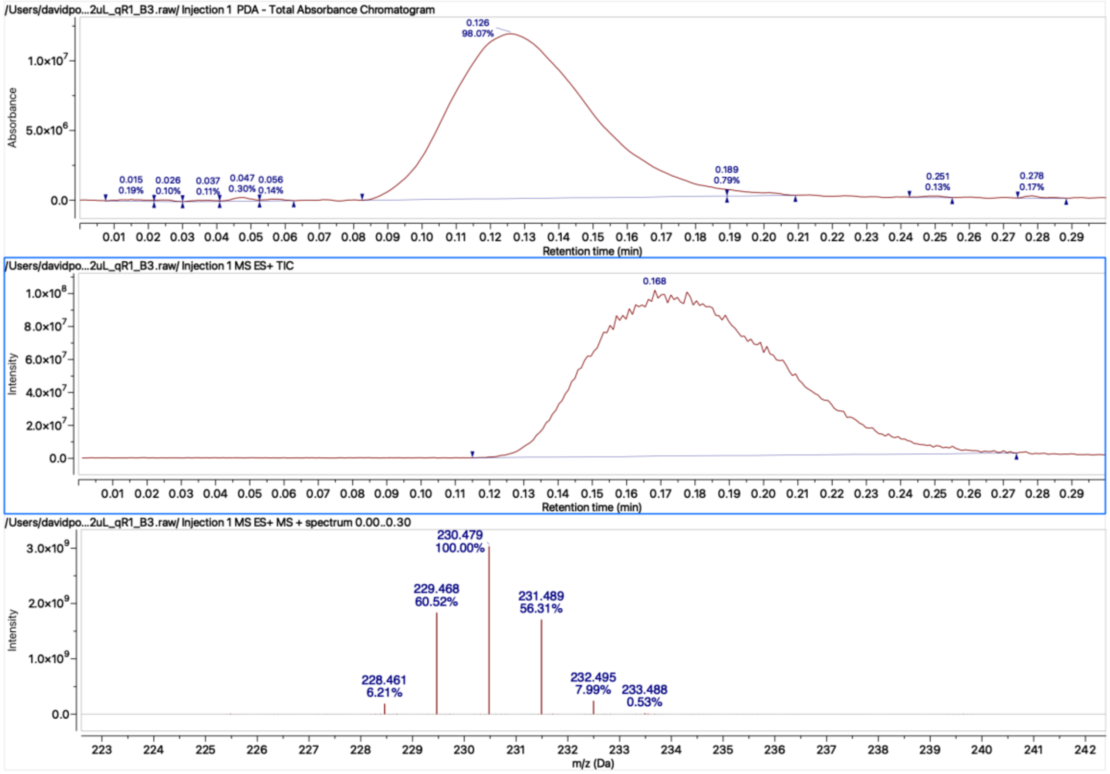

Fig. S96. *m/z* 225-240 Da used in MS file.

B3	R1	IV 0.2 μL
monosub	m/z	norm
M+1	228.48	6.21
M+2	229.45	60.52
M+3	230.45	100.00
M+4	231.46	56.31
M+5	232.49	7.99
TIC height	1.01E+08	

B3	R2	IV 0.2 μL
monosub	m/z	norm
M+1	228.48	6.26
M+2	229.45	60.82
M+3	230.45	100.00
M+4	231.46	56.13
M+5	232.49	8.00
TIC height	1.00E+08	

B3	R3	IV 0.2 μL
monosub	m/z	norm
M+1	228.48	6.25
M+2	229.45	60.58
M+3	230.45	100.00
M+4	231.46	56.40
M+5	232.49	7.87
TIC height	9.37E+07	

B3			
	AVG	SD	RSD
M+1	6.24	0.03	0.4%
M+2	60.64	0.16	0.3%
M+3	100.00	0.00	0.0%
M+4	56.28	0.14	0.2%
M+5	7.95	0.07	0.9%
TIC height	9.82E+07	3.96E+06	4.0%

Fig. S97. Numerical summary of triplicate runs for **B3**.

MS-only, B4

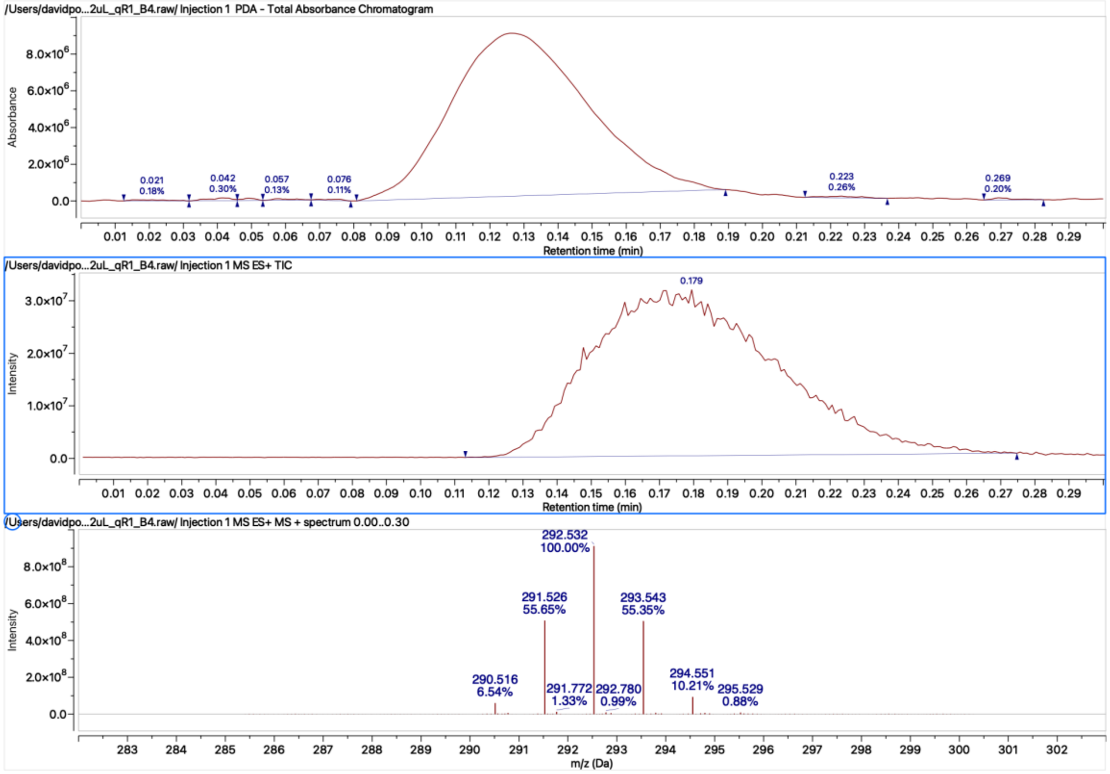

Fig. S98. *m/z* 285-300 Da used in MS file.

B4	R1	IV 0.2 μL
monosub	m/z	norm
M+1	290.46	6.54
M+2	291.50	55.65
M+3	292.49	100.00
M+4	293.49	55.35
M+5	294.49	10.21
TIC height	3.16E+07	

B4	R2	IV 0.2 μL
monosub	m/z	norm
M+1	290.46	6.06
M+2	291.50	55.79
M+3	292.49	100.00
M+4	293.49	55.45
M+5	294.49	10.07
TIC height	3.21E+07	

B4	R3	IV 0.2 μL
monosub	m/z	norm
M+1	290.46	6.32
M+2	291.50	55.68
M+3	292.49	100.00
M+4	293.49	55.85
M+5	294.49	10.16
TIC height	2.97E+07	

B4			
	AVG	SD	RSD
M+1	6.31	0.24	3.8%
M+2	55.71	0.07	0.1%
M+3	100.00	0.00	0.0%
M+4	55.55	0.26	0.5%
M+5	10.15	0.07	0.7%
TIC height	3.11E+07	1.27E+06	4.1%

Fig. S99. Numerical summary of triplicate runs for **B4**.

MS-only, B5

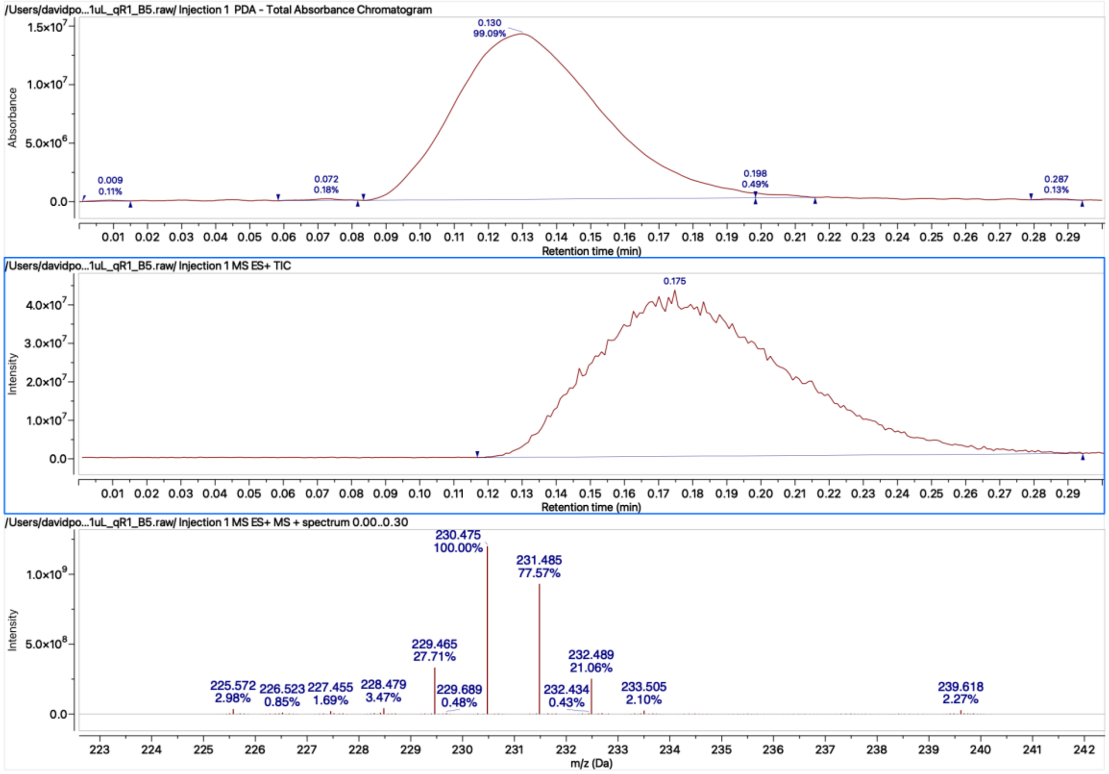

Fig. S100. *m/z* 225-240 Da used in MS file.

B5	R1	IV 1.0 μL
monosub	m/z	norm
M+1	229.45	27.71
M+2	230.44	100.00
M+3	231.45	77.57
M+4	232.50	21.06
M+5	233.49	2.10
TIC height	4.32E+07	

B5	R2	IV 1.0 μL
monosub	m/z	norm
M+1	229.45	27.64
M+2	230.44	100.00
M+3	231.45	76.84
M+4	232.50	21.07
M+5	233.49	1.98
TIC height	4.06E+07	

B5	R3	IV 1.0 μL
monosub	m/z	norm
M+1	229.45	28.23
M+2	230.44	100.00
M+3	231.45	76.92
M+4	232.50	21.22
M+5	233.49	2.01
TIC height	4.36E+07	

B5			
	AVG	SD	RSD
M+1	27.86	0.32	1.2%
M+2	100.00	0.00	0.0%
M+3	77.11	0.40	0.5%
M+4	21.12	0.09	0.4%
M+5	2.03	0.06	3.1%
TIC height	4.25E+07	1.63E+06	3.8%

Fig. S101. Numerical summary of triplicate runs for **B5**.

MS-only, B6

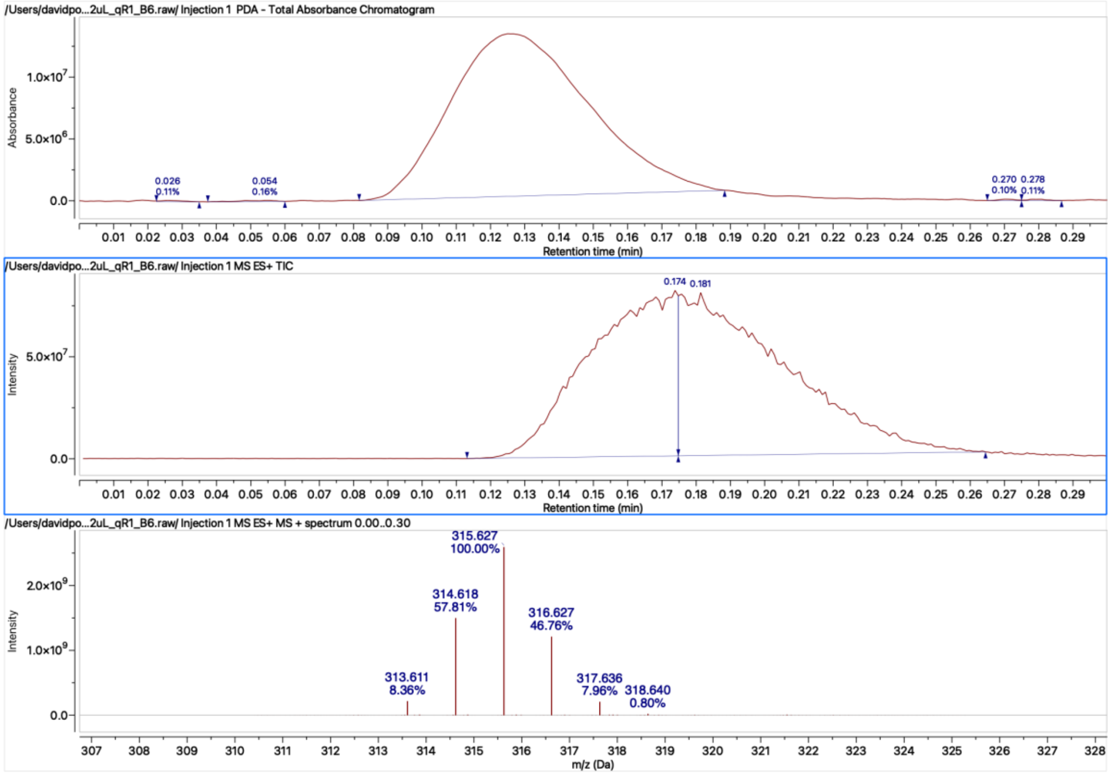

Fig. S102. *m/z* 310-325 Da used in MS file.

B6	R1	IV 0.2 µL
monosub	m/z	norm
M+1	313.57	8.36
M+2	314.57	57.81
M+3	315.57	100.00
M+4	316.57	46.76
M+5	317.57	7.96
TIC height	8.11E+07	

B6	R2	IV 0.2 µL
monosub	m/z	norm
M+1	313.57	8.49
M+2	314.57	58.07
M+3	315.57	100.00
M+4	316.57	46.75
M+5	317.57	7.95
TIC height	8.08E+07	

B6	R3	IV 0.2 µL
monosub	m/z	norm
M+1	313.57	8.41
M+2	314.57	57.61
M+3	315.57	100.00
M+4	316.57	46.51
M+5	317.57	7.91
TIC height	7.88E+07	

B6			
	AVG	SD	RSD
M+1	8.42	0.07	0.8%
M+2	57.83	0.23	0.4%
M+3	100.00	0.00	0.0%
M+4	46.67	0.14	0.3%
M+5	7.94	0.03	0.3%
TIC height	8.02E+07	1.25E+06	1.6%

Fig. S103. Numerical summary of triplicate runs for **B6**.

MS-only, C1

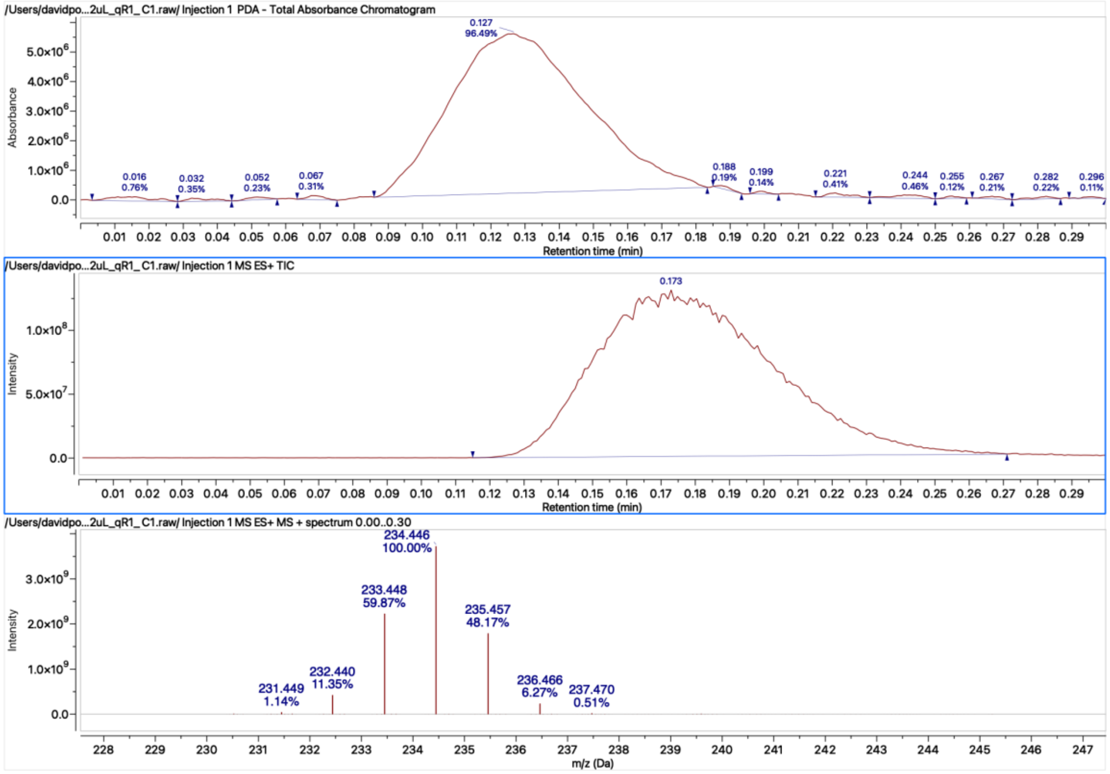

Fig. S104. *m/z* 230-245 Da used in MS file.

C1	R1	IV 0.2 μL
monosub	m/z	norm
M+1	232.41	11.35
M+2	233.42	59.87
M+3	234.45	100.00
M+4	235.42	48.17
M+5	236.45	6.27
TIC height	1.30E+08	

C1	R2	IV 0.2 μL
monosub	m/z	norm
M+1	232.41	11.39
M+2	233.42	59.80
M+3	234.45	100.00
M+4	235.42	48.85
M+5	236.45	6.26
TIC height	7.74E+07	

C1	R3	IV 0.2 μL
monosub	m/z	norm
M+1	232.41	11.46
M+2	233.42	59.94
M+3	234.45	100.00
M+4	235.42	48.56
M+5	236.45	6.12
TIC height	8.01E+07	

C1			
	AVG	SD	RSD
M+1	11.40	0.06	0.5%
M+2	59.87	0.07	0.1%
M+3	100.00	0.00	0.0%
M+4	48.53	0.34	0.7%
M+5	6.22	0.08	1.3%
TIC height	9.58E+07	2.96E+07	30.9%

Fig. S105. Numerical summary of triplicate runs for **C1**.

MS-only, C2

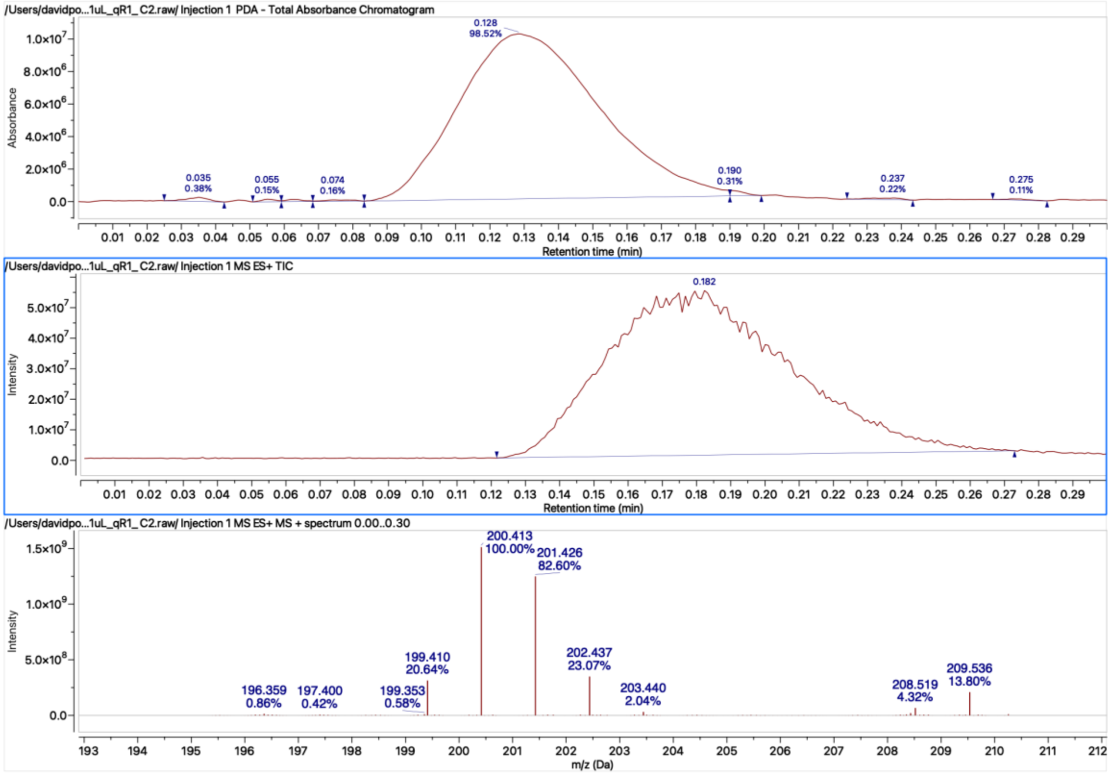

Fig. S106. *m/z* 195-210 Da used in MS file.

C2	R1	IV 1.0 μL
monosub	m/z	norm
M+1	199.31	20.64
M+2	200.36	100.00
M+3	201.36	82.60
M+4	202.40	23.07
M+5	203.39	2.04
TIC height	5.39E+07	

C2	R2	IV 1.0 μL
monosub	m/z	norm
M+1	199.31	21.06
M+2	200.36	100.00
M+3	201.36	82.83
M+4	202.40	23.09
M+5	203.39	2.04
TIC height	5.66E+07	

C2	R3	IV 1.0 μL
monosub	m/z	norm
M+1	199.31	20.73
M+2	200.36	100.00
M+3	201.36	82.37
M+4	202.40	23.22
M+5	203.39	2.12
TIC height	5.54E+07	

C2			
	AVG	SD	RSD
M+1	20.81	0.22	1.1%
M+2	100.00	0.00	0.0%
M+3	82.60	0.23	0.3%
M+4	23.13	0.08	0.4%
M+5	2.07	0.05	2.2%
TIC height	5.53E+07	1.35E+06	2.4%

Fig. S107. Numerical summary of triplicate runs for **C2**.

MS-only, C3

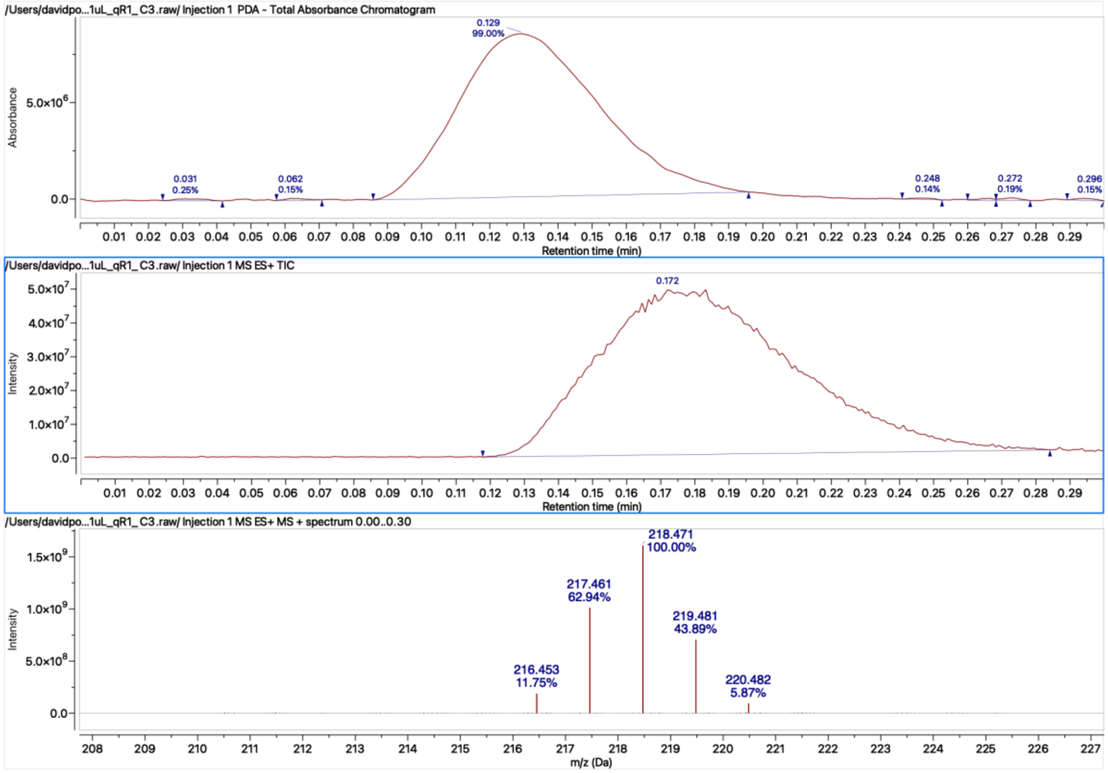

Fig. S108. *m/z* 210-225 Da used in MS file.

C3	R1	IV 1.0 μL
monosub	m/z	norm
M+1	216.40	11.75
M+2	217.42	62.94
M+3	218.42	100.00
M+4	219.43	43.89
M+5	220.47	5.87
TIC height	4.88E+07	

C3	R2	IV 1.0 μL
monosub	m/z	norm
M+1	216.40	11.76
M+2	217.42	63.65
M+3	218.42	100.00
M+4	219.43	43.32
M+5	220.47	6.07
TIC height	4.90E+07	

C3	R3	IV 1.0 μL
monosub	m/z	norm
M+1	216.40	12.04
M+2	217.42	63.92
M+3	218.42	100.00
M+4	219.43	43.39
M+5	220.47	6.15
TIC height	5.11E+07	

C3			
	AVG	SD	RSD
M+1	11.85	0.16	1.4%
M+2	63.50	0.51	0.8%
M+3	100.00	0.00	0.0%
M+4	43.53	0.31	0.7%
M+5	6.03	0.14	2.4%
TIC height	4.96E+07	1.27E+06	2.6%

Fig. S109. Numerical summary of triplicate runs for **C3**.

MS-only, C4

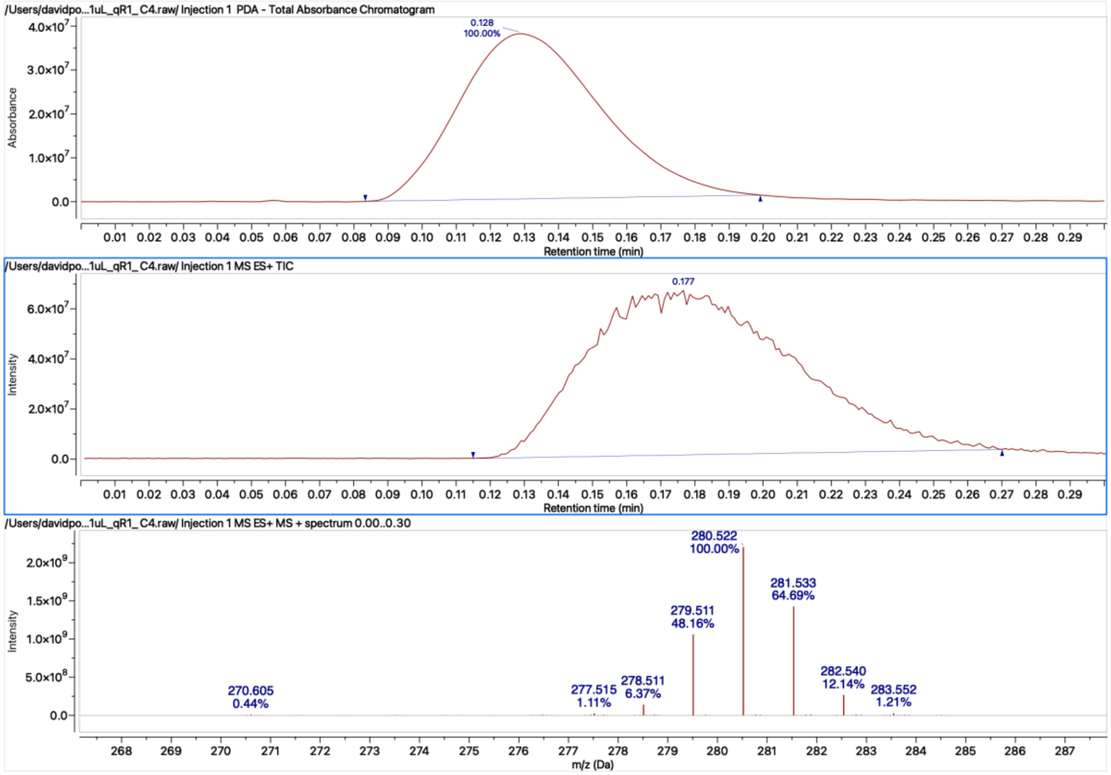

Fig. S110. *m/z* 270-285 Da used in MS file.

C4	R1	IV 1.0 μL
monosub	m/z	norm
M+1	278.47	6.37
M+2	279.48	48.16
M+3	280.48	100.00
M+4	281.48	64.69
M+5	282.52	12.14
TIC height	6.57E+07	

C4	R2	IV 1.0 μL
monosub	m/z	norm
M+1	278.47	6.41
M+2	279.48	48.05
M+3	280.48	100.00
M+4	281.48	64.98
M+5	282.52	12.23
TIC height	6.74E+07	

C4	R3	IV 1.0 μL
monosub	m/z	norm
M+1	278.47	6.40
M+2	279.48	48.42
M+3	280.48	100.00
M+4	281.48	64.66
M+5	282.52	11.86
TIC height	6.31E+07	

C4			
	AVG	SD	RSD
M+1	6.39	0.02	0.3%
M+2	48.21	0.19	0.4%
M+3	100.00	0.00	0.0%
M+4	64.78	0.18	0.3%
M+5	12.08	0.19	1.6%
TIC height	6.54E+07	2.17E+06	3.3%

Fig. S111. Numerical summary of triplicate runs for **C4**.

MS-only, C5

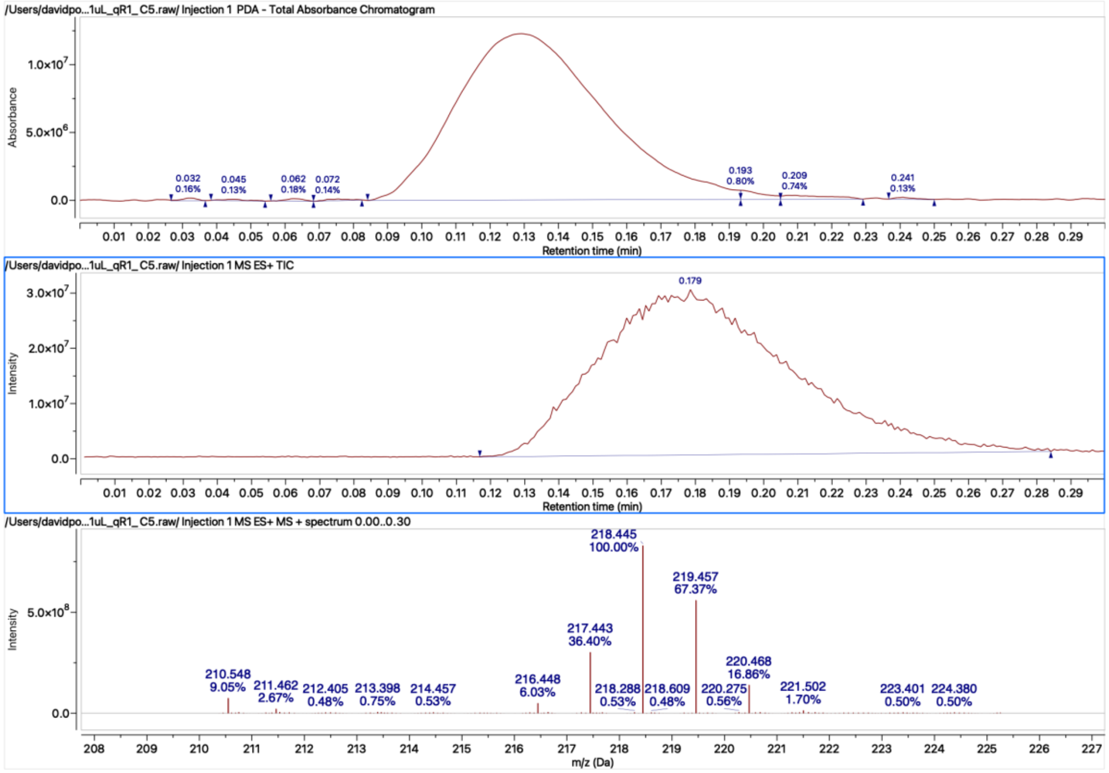

Fig. S112. *m/z* 210-225 Da used in MS file.

C5	R1	IV 1.0 μL
monosub	m/z	norm
M+1	217.37	36.40
M+2	218.36	100.00
M+3	219.41	67.37
M+4	220.44	16.86
M+5	221.48	1.70
TIC height	2.99E+07	

C5	R2	IV 1.0 μL
monosub	m/z	norm
M+1	217.37	37.09
M+2	218.36	100.00
M+3	219.41	67.70
M+4	220.44	16.70
M+5	221.48	1.57
TIC height	2.91E+07	

C5	R3	IV 1.0 μL
monosub	m/z	norm
M+1	217.37	36.75
M+2	218.36	100.00
M+3	219.41	68.02
M+4	220.44	16.83
M+5	221.48	1.87
TIC height	3.75E+07	

C5			
	AVG	SD	RSD
M+1	36.75	0.35	0.9%
M+2	100.00	0.00	0.0%
M+3	67.70	0.33	0.5%
M+4	16.80	0.09	0.5%
M+5	1.71	0.15	8.8%
TIC height	3.22E+07	4.64E+06	14.4%

Fig. S113. Numerical summary of triplicate runs for **C5**.

MS-only, C6

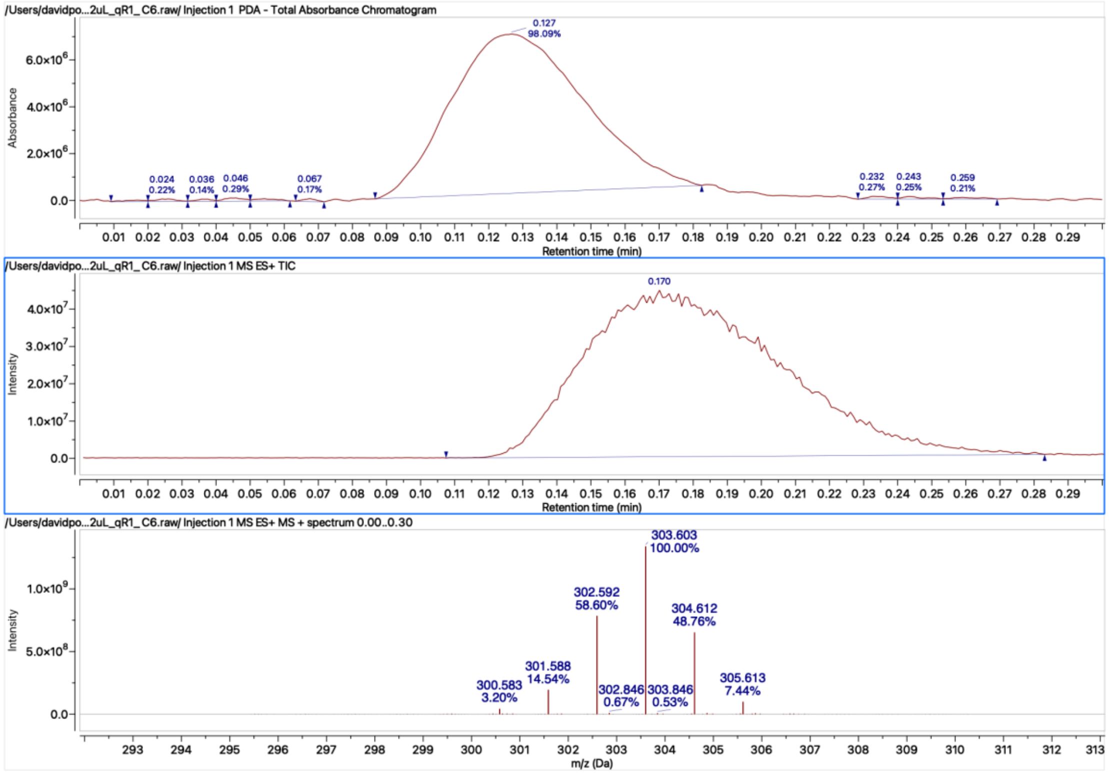

Fig. S114. *m/z* 295-310 Da used in MS file.

C6	R1	IV 0.2 μL
monosub	m/z	norm
M+1	301.53	14.54
M+2	302.55	58.60
M+3	303.55	100.00
M+4	304.54	48.76
M+5	305.53	7.44
TIC height	4.46E+07	

C6	R2	IV 0.2 μL
monosub	m/z	norm
M+1	301.53	14.30
M+2	302.55	58.31
M+3	303.55	100.00
M+4	304.54	48.57
M+5	305.53	7.55
TIC height	4.56E+07	

C6	R3	IV 0.2 μL
monosub	m/z	norm
M+1	301.53	14.38
M+2	302.55	57.82
M+3	303.55	100.00
M+4	304.54	49.02
M+5	305.53	7.78
TIC height	4.45E+07	

C6			
	AVG	SD	RSD
M+1	14.41	0.12	0.8%
M+2	58.24	0.39	0.7%
M+3	100.00	0.00	0.0%
M+4	48.78	0.23	0.5%
M+5	7.59	0.17	2.3%
TIC height	4.49E+07	6.08E+05	1.4%

Fig. S115. Numerical summary of triplicate runs for **C6**.

MS-only, D1

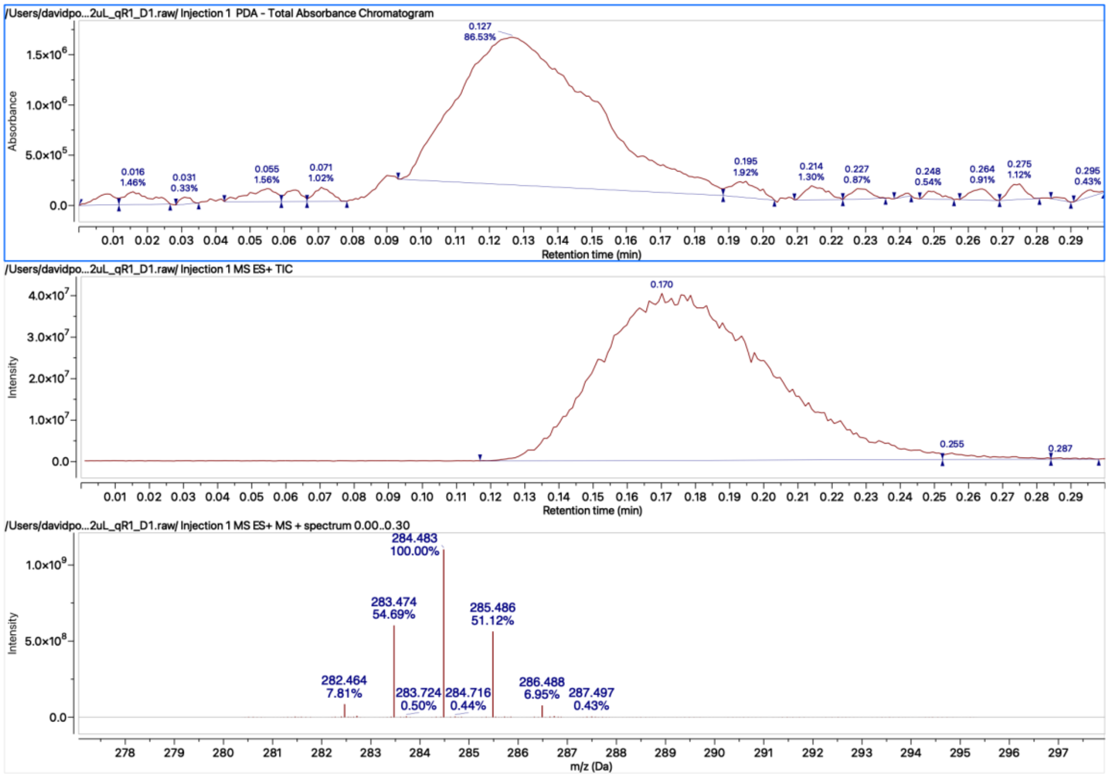

Fig. S116. *m/z* 280-295 Da used in MS file.

D1	qR1	0.2 uL
monosub	m/z	norm
M+1	282.43	7.81
M+2	283.47	54.69
M+3	284.46	100.00
M+4	285.46	51.12
M+5	286.49	6.95
TIC height	4.03E+07	

D1	qR2	
monosub	m/z	norm
M+1	282.43	8.09
M+2	283.47	55.03
M+3	284.46	100.00
M+4	285.46	51.16
M+5	286.49	6.59
TIC height	3.88E+07	

D1	qR3	
monosub	m/z	norm
M+1	282.43	8.07
M+2	283.47	54.91
M+3	284.46	100.00
M+4	285.46	51.44
M+5	286.49	6.51
TIC height	3.78E+07	

D1			
	AVG	SD	RSD
M+1	7.99	0.16	2.0%
M+2	54.88	0.17	0.3%
M+3	100.00	0.00	0.0%
M+4	51.24	0.17	0.3%
M+5	6.68	0.23	3.5%
TIC height	3.90E+07	1.26E+06	3.2%

Fig. S117. Numerical summary of triplicate runs for **D1**.

MS-only, D2

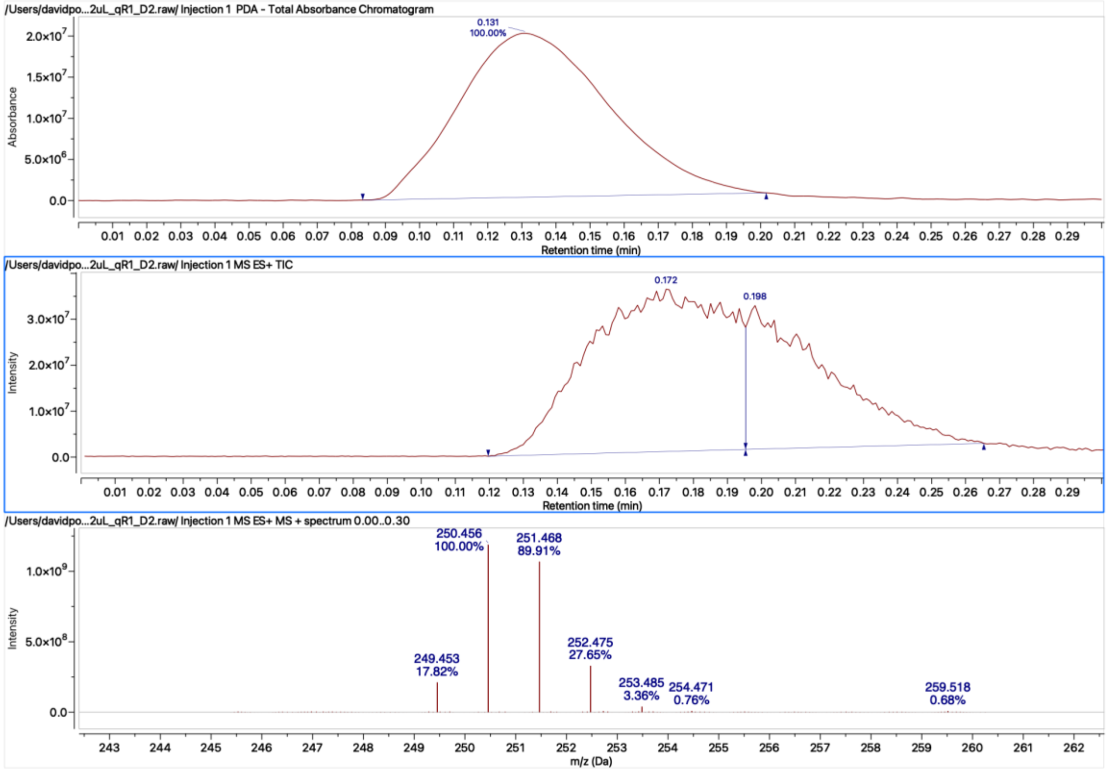

Fig. S118. *m/z* 245-260 Da used in MS file.

D2	qR1	2 uL
monosub	m/z	norm
M+1	249.42	17.82
M+2	250.43	100.00
M+3	251.43	89.91
M+4	252.46	27.65
M+5	253.30	3.36
TIC height	3.54E+07	

D2	qR2	
monosub	m/z	norm
M+1	249.42	17.89
M+2	250.43	100.00
M+3	251.43	90.83
M+4	252.46	28.03
M+5	253.30	3.17
TIC height	3.38E+07	

D2	qR3	
monosub	m/z	norm
M+1	249.42	18.01
M+2	250.43	100.00
M+3	251.43	90.48
M+4	252.46	27.63
M+5	253.30	3.32
TIC height	3.59E+07	

D2			
	AVG	SD	RSD
M+1	17.91	0.10	0.5%
M+2	100.00	0.00	0.0%
M+3	90.41	0.46	0.5%
M+4	27.77	0.23	0.8%
M+5	3.28	0.10	3.1%
TIC height	3.50E+07	1.10E+06	3.1%

Fig. S119. Numerical summary of triplicate runs for **D2**.

MS-only, D3

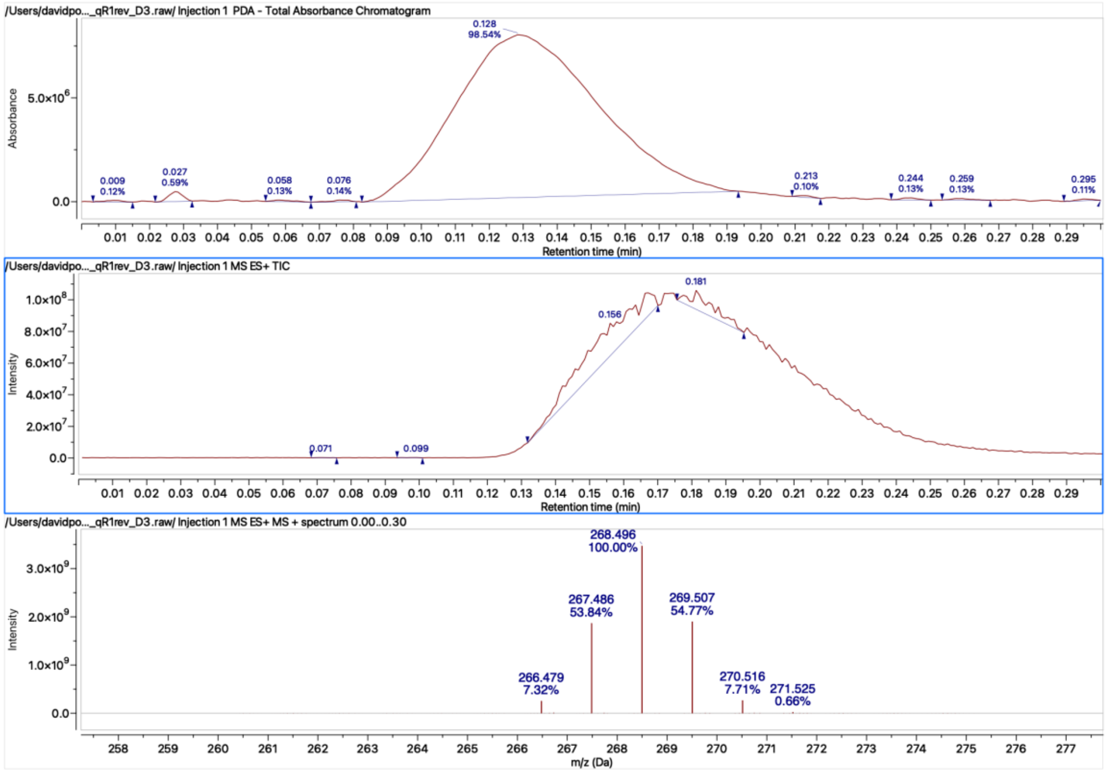

Fig. S120. *m/z* 260-275 Da used in MS file.

D3	qR1	1 uL
monosub	m/z	norm
M+1	266.43	7.32
M+2	267.47	53.84
M+3	268.46	100.00
M+4	269.47	54.77
M+5	270.47	7.71
TIC height	1.04E+08	

D3	qR2	
monosub	m/z	norm
M+1	266.43	7.38
M+2	267.47	54.05
M+3	268.46	100.00
M+4	269.47	54.81
M+5	270.47	7.83
TIC height	1.02E+08	

D3	qR3	
monosub	m/z	norm
M+1	266.43	7.41
M+2	267.47	53.92
M+3	268.46	100.00
M+4	269.47	54.77
M+5	270.47	7.82
TIC height	1.21E+08	

D3			
	AVG	SD	RSD
M+1	7.37	0.05	0.6%
M+2	53.94	0.11	0.2%
M+3	100.00	0.00	0.0%
M+4	54.78	0.02	0.0%
M+5	7.79	0.07	0.9%
TIC height	1.09E+08	1.04E+07	9.6%

Fig. S121. Numerical summary of triplicate runs for **D3**.

MS-only, D4

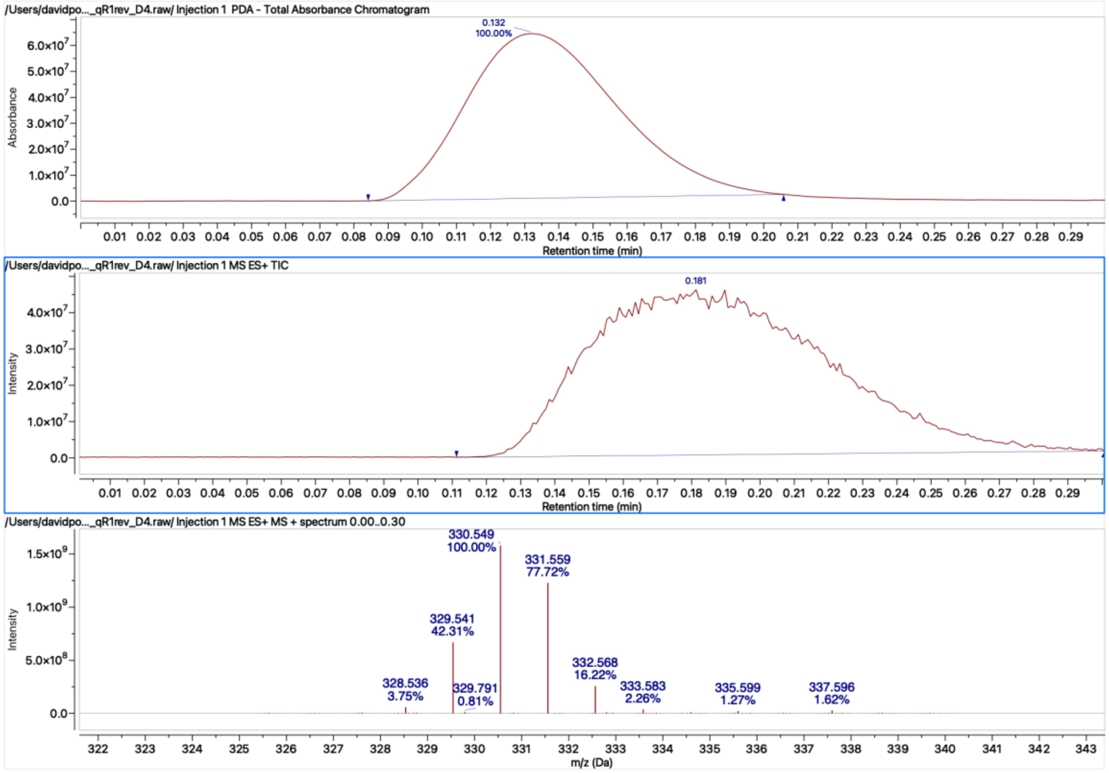

Fig. S122. *m/z* 325-340 Da used in MS file.

D4	qR1	2 uL
monosub	m/z	norm
M+1	328.37	3.75
M+2	329.46	42.31
M+3	330.47	100.00
M+4	331.53	77.72
M+5	332.57	16.22
TIC height	4.55E+07	

D4	qR2	
monosub	m/z	norm
M+1	328.37	3.89
M+2	329.46	42.89
M+3	330.47	100.00
M+4	331.53	77.58
M+5	332.57	16.45
TIC height	4.41E+07	

D4	qR3	
monosub	m/z	norm
M+1	328.37	3.73
M+2	329.46	42.27
M+3	330.47	100.00
M+4	331.53	77.57
M+5	332.57	16.54
TIC height	4.66E+07	

D4			
	AVG	SD	RSD
M+1	3.79	0.09	2.3%
M+2	42.49	0.35	0.8%
M+3	100.00	0.00	0.0%
M+4	77.62	0.08	0.1%
M+5	16.40	0.17	1.0%
TIC height	4.54E+07	1.25E+06	2.8%

Fig. S123. Numerical summary of triplicate runs for **D4**.

MS-only, D5

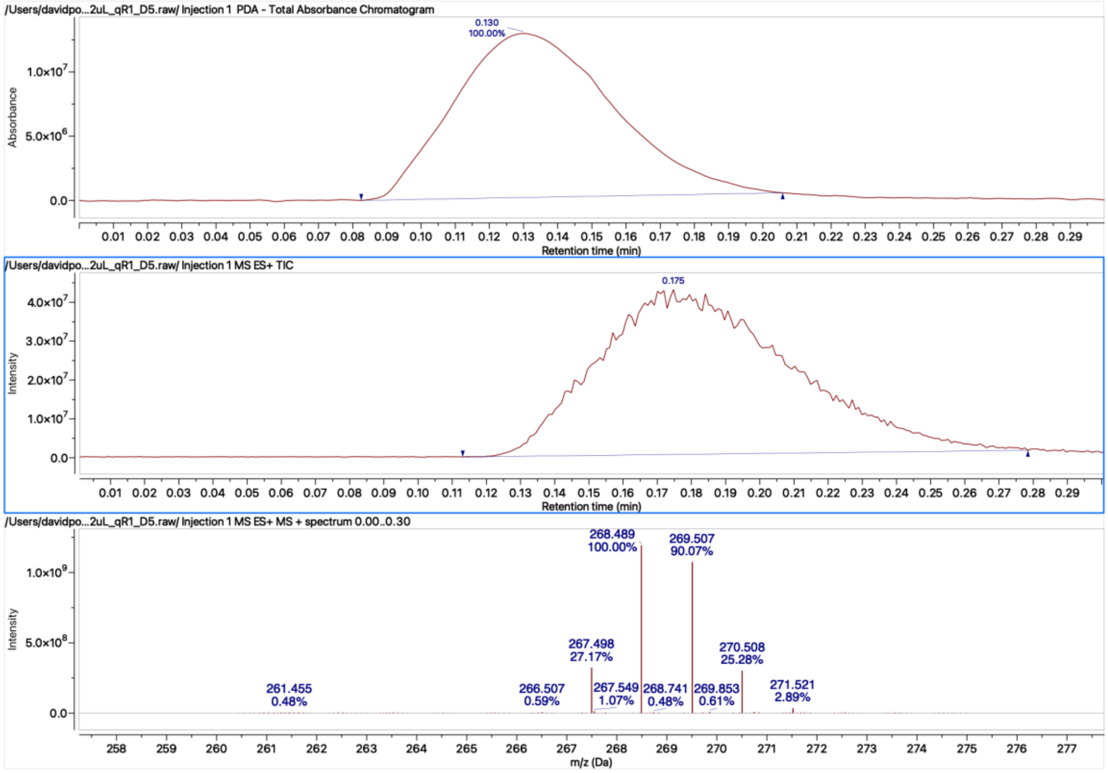

Fig. S124. *m/z* 260-275 Da used in MS file.

D5	qR1	2 uL
monosub	m/z	norm
M+1	267.51	27.17
M+2	268.48	100.00
M+3	269.51	90.07
M+4	270.44	25.28
M+5	271.43	2.89
TIC height	4.24E+07	

D5	qR2	
monosub	m/z	norm
M+1	267.51	27.21
M+2	268.48	100.00
M+3	269.51	89.98
M+4	270.44	25.17
M+5	271.43	2.84
TIC height	4.14E+07	

D5	qR3	
monosub	m/z	norm
M+1	267.51	26.98
M+2	268.48	100.00
M+3	269.51	89.21
M+4	270.44	25.01
M+5	271.43	3.01
TIC height	4.21E+07	

D5			
	AVG	SD	RSD
M+1	27.12	0.12	0.5%
M+2	100.00	0.00	0.0%
M+3	89.75	0.47	0.5%
M+4	25.15	0.14	0.5%
M+5	2.91	0.09	3.0%
TIC height	4.20E+07	5.13E+05	1.2%

Fig. S125. Numerical summary of triplicate runs for **D5**.

MS-only, D6

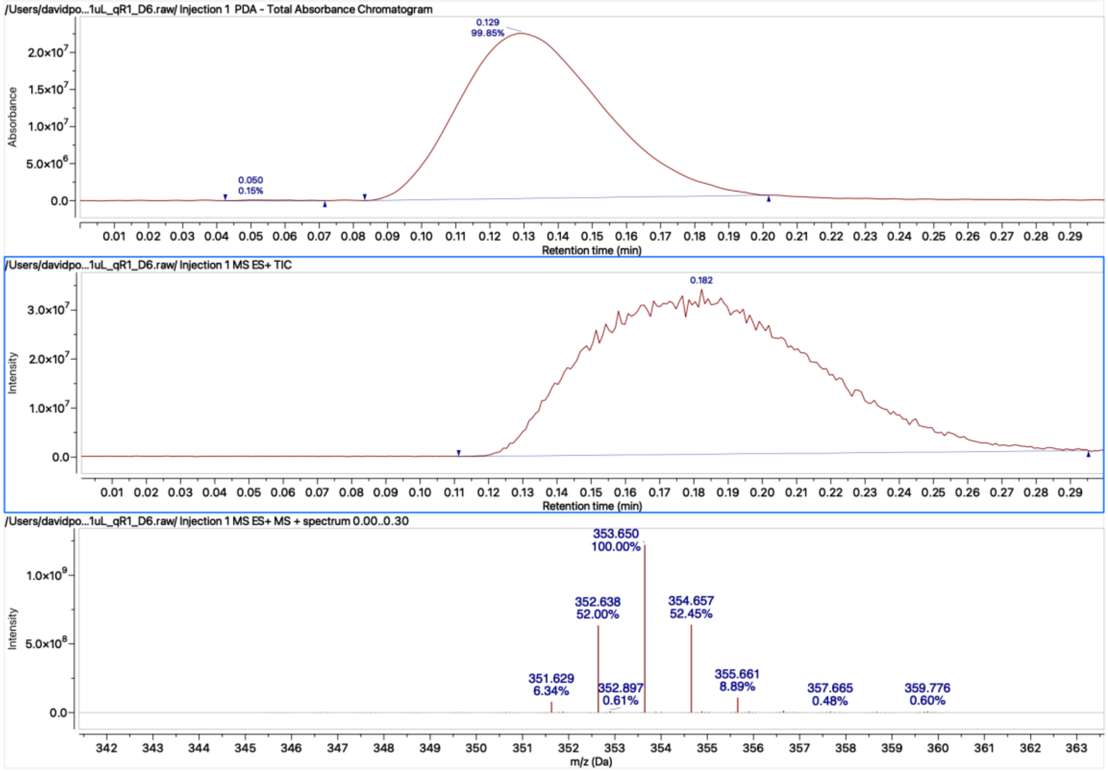

Fig. S126. *m/z* 345-360 Da used in MS file.

D6	qR1	1 uL
monosub	m/z	norm
M+1	351.54	6.34
M+2	352.58	52.00
M+3	353.58	100.00
M+4	354.58	52.45
M+5	355.55	8.89
TIC height	3.37E+07	

D6	qR2	
monosub	m/z	norm
M+1	351.54	6.28
M+2	352.58	51.45
M+3	353.58	100.00
M+4	354.58	52.10
M+5	355.55	8.82
TIC height	3.35E+07	

D6	qR3	
monosub	m/z	norm
M+1	351.54	6.27
M+2	352.58	51.78
M+3	353.58	100.00
M+4	354.58	52.97
M+5	355.55	8.73
TIC height	3.30E+07	

D6			
	AVG	SD	RSD
M+1	6.30	0.04	0.6%
M+2	51.74	0.28	0.5%
M+3	100.00	0.00	0.0%
M+4	52.51	0.44	0.8%
M+5	8.81	0.08	0.9%
TIC height	3.34E+07	3.61E+05	1.1%

Fig. S127. Numerical summary of triplicate runs for **D6**.

5.2.3.3 Variance in Isotope Patterns in MS-Only Measurements for 24-Well Plate

The average relative standard deviation (RSD) from triplicate measurements for the four non-base peak normalized intensities across all 24 wells (96 total peaks considered; 288 discrete values) was 1.0%. The average RSD from triplicate measurements for the maximum TIC heights across all 24 wells (24 discrete values) was 5.8%.

Even in extreme cases of TIC variance, the RSD of normalized intensities exhibited good precision. For example, well A2 exhibited a 21.7% RSD in the TIC height despite only a 1.3% RSD across four total peaks and 12 discrete values.

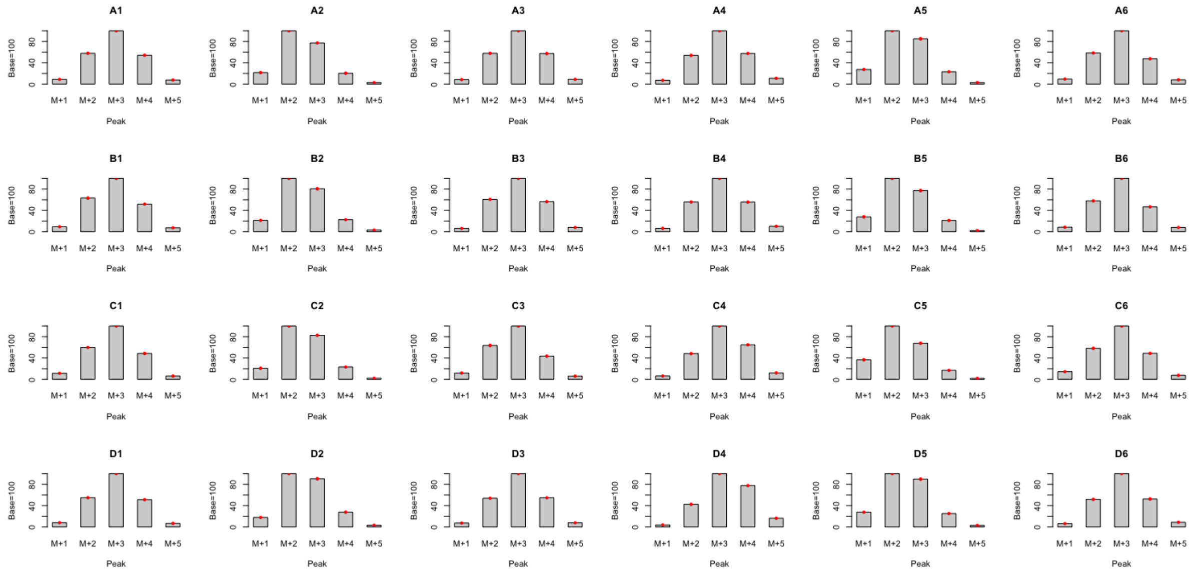

Fig. S128. Respective isotopic clusters for 24 reaction wells, with normalized intensities plotted for all spectra. Measurements shown are averages of triplicates; standard deviation bars are shown in red.

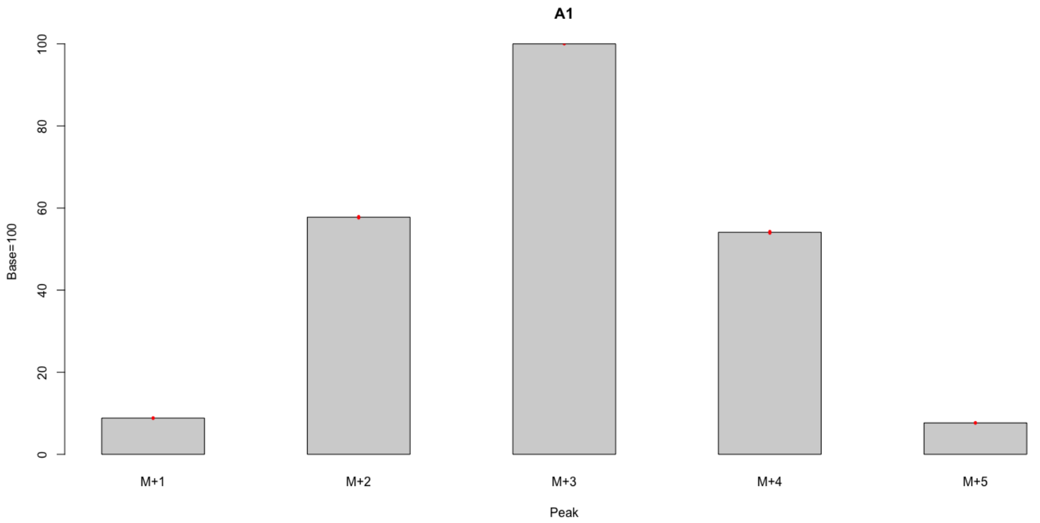

Fig. S129. Inset view of well **A1** from the above Fig..

5.2.3.4 MS-Only Deconvolution Methods for 24-Well Plate

The methods in **Sections 1.2.6** were used with the C_{NMR} matrix values (isotopic distributions predicted from labelling amounts obtained using the methods in **Section 2**.

The methods in **Sections 1.2.6** were used with the C_{LC} matrix values obtained from the separated products in the UPLC-MS analyses from **Section 5.2.2** Separation of multiple regioisomeric species was not always feasible for a given well in this 24-well plate. As a result, when sufficient separation was not achieved another $\mathbf{C_{LC}}$ matrix from the same substrate was used. This assumption is reasonable given that that the differences between NMR-expected isotopic distributions and LC-observed isotopic distributions should be solely a function of substrate labeling states, which are constant within each column. Furthermore, since this model case does not exhibit meaningful kinetic isotope effects (KIEs), reaction-to-reaction differences in conversion are not expected to materially impact these quantitative results.

Unless otherwise noted, deconvolution of regioisomers was performed using the $\mathbf{C_{LC}}$ matrix for each unique well. Noted below are wells which, owing to incomplete peak separation, was referenced to another well utilizing the same substrate:

B1; C1; and D1 use the $\mathbf{C_{LC}}$ of A1.

A3; B3; and D3 use the $\mathbf{C_{LC}}$ of C3.

For column 3, the C3 matrix was deemed preferable to the B3 matrix due to small yet consequential noise at the *m/z* 228 peak (M+1), and UPLC separation for A3 was not achieved. Otherwise, entries in the “A” row were selected by convention for $\mathbf{C_{LC}}$ deconvolution.

Both OLS and NNLS were undertaken with minimal differences except for wells A4-D4 where OLS generated negative values, likely due to the similarity between the isotopic distributions of the R4 and RPh product. Final deconvolution results for the 24-well plate are presented using NNLS regression.

Well	M+1	M+2	M+3	M+4	M+5
**A1**	8.83 ± 0.07	57.76 ± 0.17	100 ± 0	54.09 ± 0.21	7.66 ± 0.07
**A2**	21.55 ± 0.2	100 ± 0	77.21 ± 0.12	20.34 ± 0.37	2.78 ± 0.06
**A3**	8.46 ± 0.1	57.75 ± 0.27	100 ± 0	57.27 ± 0.35	8.88 ± 0.08
**A4**	7.06 ± 0.12	53.89 ± 0.32	100 ± 0	57.4 ± 0.14	10.79 ± 0.16
**A5**	27.4 ± 0.13	100 ± 0	84.96 ± 0.55	23.1 ± 0.09	2.75 ± 0.12
**A6**	9.41 ± 0.03	58.37 ± 0.12	100 ± 0	47.49 ± 0.11	7.99 ± 0.08
**B1**	9.37 ± 0.02	63.19 ± 0.36	100 ± 0	51.8 ± 0.04	7.36 ± 0.03
**B2**	21.27 ± 0.06	100 ± 0	80.56 ± 0.42	22.64 ± 0.02	3.2 ± 0.01
**B3**	6.24 ± 0.03	60.64 ± 0.16	100 ± 0	56.28 ± 0.14	7.95 ± 0.07
**B4**	6.31 ± 0.24	55.71 ± 0.07	100 ± 0	55.55 ± 0.26	10.15 ± 0.07
**B5**	27.86 ± 0.32	100 ± 0	77.11 ± 0.4	21.12 ± 0.09	2.03 ± 0.06
**B6**	8.42 ± 0.07	57.83 ± 0.23	100 ± 0	46.67 ± 0.14	7.94 ± 0.03
**C1**	11.4 ± 0.06	59.87 ± 0.07	100 ± 0	48.53 ± 0.34	6.22 ± 0.08
**C2**	20.81 ± 0.22	100 ± 0	82.6 ± 0.23	23.13 ± 0.08	2.07 ± 0.05
**C3**	11.85 ± 0.16	63.5 ± 0.51	100 ± 0	43.53 ± 0.31	6.03 ± 0.14
**C4**	6.39 ± 0.02	48.21 ± 0.19	100 ± 0	64.78 ± 0.18	12.08 ± 0.19
**C5**	36.75 ± 0.35	100 ± 0	67.7 ± 0.33	16.8 ± 0.09	1.71 ± 0.15
**C6**	14.41 ± 0.12	58.24 ± 0.39	100 ± 0	48.78 ± 0.23	7.59 ± 0.17
**D1**	7.99 ± 0.16	54.88 ± 0.17	100 ± 0	51.24 ± 0.17	6.68 ± 0.23
**D2**	17.91 ± 0.1	100 ± 0	90.41 ± 0.46	27.77 ± 0.23	3.28 ± 0.1
**D3**	7.37 ± 0.05	53.94 ± 0.11	100 ± 0	54.78 ± 0.02	7.79 ± 0.07
**D4**	3.79 ± 0.09	42.49 ± 0.35	100 ± 0	77.62 ± 0.08	16.4 ± 0.17
**D5**	27.12 ± 0.12	100 ± 0	89.75 ± 0.47	25.15 ± 0.14	2.91 ± 0.09
**D6**	6.3 ± 0.04	51.74 ± 0.28	100 ± 0	52.51 ± 0.44	8.81 ± 0.08

Table S2. Summary of LOOP injection values obtained for the 24-well plate, including the standard deviation of each M+x value. Because these results are normalized the standard deviation of the most abundant peak is 0.

OLS Deconvolution for the 24-well plate in R

See file: OLS_NNLS_Deconvolution.R

Terms were defined for a data array of predicted R2, R4, and R6 isotopic distributions for the 24-well plate (lines 3,7,9-12).

```
# Defining Terms #
peaknames = c("M+1","M+2","M+3","M+4","M+5")
regios = c("R2","R4","R6")
names_24w = c("A1","A2","A3","A4","A5","A6",
              "B1","B2","B3","B4","B5","B6",
              "C1","C2","C3","C4","C5","C6",
              "D1","D2","D3","D4","D5","D6")
```

Next, the **C_{NMR}** values calculated for each isomeric product are entered. The figure below shows values for wells A1 and A2 (lines 99-107) but this is done for every well in a similar format (lines 99-195).

```
C_NMR_ARR_noPh <- array(numeric(),c(5,3,24), dimnames=list(peaknames, regios, names_24w))

C_NMR_ARR_noPh[,1,1] <- c(0.4478, 50.118, 100.00, 19.938, 2.0508)
C_NMR_ARR_noPh[,2,1] <- c(0.1961, 22.384, 90.695, 100, 14.733)
C_NMR_ARR_noPh[,3,1] <- c(20.625, 86.974, 100, 19.164, 1.8829)

C_NMR_ARR_noPh[,1,2] <- c(1, 100, 70.065, 10.897, 0.7174)
C_NMR_ARR_noPh[,2,2] <- c(0.6827, 68.592, 100, 42.364, 5.0214)
C_NMR_ARR_noPh[,3,2] <- c(66.35, 100, 44.803, 6.2604, 0.3842)
```

For the OLS or NNLS analysis, unnormalized **C_{NMR}** values are generated (lines 200-206):

```
C_NMR_ARR_unnormalized <- array(numeric(), c(5, 3, 24), dimnames = list(peaknames, regios, names_24w))

# Loop through each slice of C_NMR_ARR_noPh
for (k in 1:24) {
  # Unnormalize each slice
  C_NMR_ARR_unnormalized[, , k] <- t(t(C_NMR_ARR_noPh[, , k]) / colSums(C_NMR_ARR_noPh[, , k])) * 100
}
```

A data array containing the measured LOOP injection m/z values is entered (lines 62-86).

```
HTE4_qAVG_LOOP <- array(numeric(),c(24,5),dimnames=list(names_24w, peaknames[1:5]))
HTE4_qAVG_LOOP["A1",1:5] <- c(8.83, 57.76, 100.00, 54.09, 7.66)
HTE4_qAVG_LOOP["A2",1:5] <- c(21.55, 100.00, 77.21, 20.34, 2.78)
HTE4_qAVG_LOOP["A3",1:5] <- c(8.46, 57.75, 100.00, 57.27, 8.88)
HTE4_qAVG_LOOP["A4",1:5] <- c(7.06, 53.89, 100.00, 57.40, 10.79)
HTE4_qAVG_LOOP["A5",1:5] <- c(27.40, 100.00, 84.96, 23.10, 2.75)
HTE4_qAVG_LOOP["A6",1:5] <- c(9.41, 58.37, 100.00, 47.49, 7.99)
HTE4_qAVG_LOOP["B1",1:5] <- c(9.37, 63.19, 100.00, 51.80, 7.36)
HTE4_qAVG_LOOP["B2",1:5] <- c(21.27, 100.00, 80.56, 22.64, 3.20)
HTE4_qAVG_LOOP["B3",1:5] <- c(6.24, 60.64, 100.00, 56.28, 7.95)
HTE4_qAVG_LOOP["B4",1:5] <- c(6.31, 55.71, 100.00, 55.55, 10.15)
HTE4_qAVG_LOOP["B5",1:5] <- c(27.86, 100.00, 77.11, 21.12, 2.03)
HTE4_qAVG_LOOP["B6",1:5] <- c(8.42, 57.83, 100.00, 46.67, 7.94)
HTE4_qAVG_LOOP["C1",1:5] <- c(11.40, 59.87, 100.00, 48.53, 6.22)
HTE4_qAVG_LOOP["C2",1:5] <- c(20.81, 100.00, 82.60, 23.13, 2.07)
HTE4_qAVG_LOOP["C3",1:5] <- c(11.85, 63.50, 100.00, 43.53, 6.03)
HTE4_qAVG_LOOP["C4",1:5] <- c(6.39, 48.21, 100.00, 64.78, 12.08)
HTE4_qAVG_LOOP["C5",1:5] <- c(36.75, 100.00, 67.70, 16.80, 1.71)
HTE4_qAVG_LOOP["C6",1:5] <- c(14.41, 58.24, 100.00, 48.78, 7.59)
HTE4_qAVG_LOOP["D1",1:5] <- c(7.99, 54.88, 100.00, 51.24, 6.68)
HTE4_qAVG_LOOP["D2",1:5] <- c(17.91, 100.00, 90.41, 27.77, 3.28)
HTE4_qAVG_LOOP["D3",1:5] <- c(7.37, 53.94, 100.00, 54.78, 7.79)
HTE4_qAVG_LOOP["D4",1:5] <- c(3.79, 42.49, 100.00, 77.62, 16.40)
HTE4_qAVG_LOOP["D5",1:5] <- c(27.72, 100.00, 89.75, 25.15, 2.91)
HTE4_qAVG_LOOP["D6",1:5] <- c(6.30, 51.74, 100.00, 52.51, 8.81)
```

The OLS analysis and extraction of the weights for the R2, R4, and R6 regioisomers and their corresponding p-values (lines 213-244).

```
results <- lapply(names_24w, function(name) {
  # Extract response vector
  response <- HTE4_qAVG_LOOP[name, ]

  # Extract predictors for this response vector
  predictors <- C_NMR_ARR_unnormalized[, 1:3, name]  # Select only columns R2, R4, R6

  # Fit OLS model
  model <- lm(response ~ predictors)

  # Get summary of the model
  summary_output <- summary(model)

  # Extract coefficients, R-squared, and p-values
  coefficients <- summary_output$coefficients
  r_squared <- summary_output$r.squared
  p_values <- summary_output$coefficients[, 4]  # Pr(>|t|) is in the 4th column

  # Return the results as a list
  # Create a list with predictor values, t-values, and R-squared
  result <- list(name = name,
                 R2_coef = coefficients[2, 1],
                 R2_p_value = p_values[2],
                 R4_coef = coefficients[3, 1],
                 R4_p_value = p_values[3],
                 R6_coef = coefficients[4, 1],
                 R6_p_value = p_values[4],
                 r_squared = r_squared)

  # Return the result
  return(result)
})
```

The weights obtained for R2, R4, and R6 are not normalized but the values can be normalized (lines 247-263).

```
# Convert the list of results to a dataframe
results_CNMR <- bind_rows(results)

# Print the dataframe
R2_values <- results_CNMR$R2_coef
R4_values <- results_CNMR$R4_coef
R6_values <- results_CNMR$R6_coef
# Create a matrix with these values
data_array <- matrix(c(R2_values, R4_values, R6_values), nrow = 24, byrow = FALSE, dimnames= list(NULL, c("R2","R4","R6")))
###############

###############
#GET NORMALIZED DATA ARRAY C_NMR###
norm_data_array_CNMR <- array(numeric(),c(24,3),dimnames=list(names_24w,regios))
for (i in 1:24) {
  # unnormalize each slice
  norm_data_array_CNMR[i,] <- round((data_array[i,] / sum(data_array[i,])),3)*100
}
```

The p-values for these weights are generated (lines 269-273)

```
## GET ARRAY OF P VALUES
R2_p_values <- results_CNMR$R2_p_value
R4_p_values <- results_CNMR$R4_p_value
R6_p_values <- results_CNMR$R6_p_value
# Create a matrix with these values
pvalue_array_NMR <- round(matrix(c(R2_p_values, R4_p_values, R6_p_values), nrow = 24, byrow = FALSE,dimnames= list(NULL, c("R2","R4","R6"))),3)
```

NNLS deconvolution of the 24-well plate in R

See file: OLS_NNLS_Deconvolution.R

With the data frames from above (HTE4_qAVG_LOOP and C_NMR_ARR_unnormalized), NNLS deconvolution can be performed using the nnls package in R (lines 288-292). The code below gives the relative product ratios out of 100 and also generates the residual sum of squares values (lines 295-304).

```
#### PERFORMS NNLS AND ROUNDS DATA TO 3 decimal places ######
wNNLS_C_NMR_ARR <- array(numeric(),c(24,3),dimnames=list(names_24w,regios))
for (i in 1:24) {
  wNNLS <- nnls(C_NMR_ARR_unnormalized[,,i], HTE4_qAVG_LOOP[i,])$x
  wNNLS_C_NMR_ARR[i,] <- round((wNNLS / sum(wNNLS[wNNLS > 0])),3) * 100
}

### PERFORM THE NNLS AND PULL OUT RESIDUAL SUM OF SQUARES ###
wNNLS_C_NMR_ARR_sumsquares <- array(numeric(),c(24,1),dimnames=list(names_24w,"sum of squares"))
for (i in 1:24) {
  wNNLS_C_NMR_ARR_sumsquares[i,] <- nnls(C_NMR_ARR_unnormalized[,,i], HTE4_qAVG_LOOP[i,])$deviance
}

#### ROUNDS SUM OF SQUARES TO 3 DECIMAL PLACES ######
round_C_NMR_sumsquares <- array(numeric(),c(24,1),dimnames=list(names_24w,"sum of squares"))
for (i in 1:24){
  round_C_NMR_sumsquares[i,] <- round(wNNLS_C_NMR_ARR_sumsquares[i,],3)
}
```

For wells A4, B4, C4, and D4 where arylation can occur on the phenyl ring, a similar analysis is done using the unnormalized predicted isotopic distributions of the R2, R4, R6, and RPh regioisomers.

5.2.3.5 MS-Only Deconvolution Data for 24-Well Plate

Well	R2	R4	R6
A1	11.9 (0.173)	42 (0.024)	46.1 (0.049)
A2	41.9 (0.01)	28 (0.015)	30.1 (0.011)
A3	10.9 (0.364)	46.2 (0.048)	42.9 (0.11)
A4	—	—	—
A5	19.3 (0.188)	43.9 (0.083)	36.8 (0.077)
A6	27.7 (0.058)	31.4 (0.024)	40.9 (0.041)
B1	5.1 (0.685)	38.5 (0.074)	56.4 (0.114)
B2	37.8 (0.008)	33.3 (0.009)	28.9 (0.008)
B3	6.1 (0.745)	45 (0.1)	48.9 (0.195)
B4	—	—	—
B5	26.9 (0.005)	34.1 (0.004)	39 (0.003)
B6	30.9 (0.02)	30.5 (0.01)	38.7 (0.017)
C1	13.9 (0.14)	36.5 (0.026)	49.5 (0.043)
C2	32.8 (0.018)	38.6 (0.015)	28.7 (0.016)
C3	17.9 (0.004)	31.2 (0.001)	50.9 (0.001)
C4	—	—	—
C5	23.3 (0.013)	24.6 (0.012)	52.1 (0.005)
C6	21.9 (0.458)	33.1 (0.169)	45 (0.269)
D1	21.1 (0.026)	39.9 (0.007)	38.9 (0.015)
D2	30.1 (0.006)	46.7 (0.004)	23.3 (0.006)
D3	21 (0.057)	44.9 (0.013)	34.2 (0.037)
D4	—	—	—
D5	13.7 (0.314)	50.1 (0.09)	36.3 (0.097)
D6	34.4 (0.06)	38.7 (0.025)	26.9 (0.081)

Table S3. Summary of product ratios as determined via OLS analysis. In parenthesis are the corresponding p-values obtained. Due to negative values obtained for wells A4, B4, C4, and D4 they are omitted from this table and are shown in the table summarizing NNLS results.

Well	R2	R4	R6	RPh	RSS
**A1**	12.5	41.9	45.6	NA	0.834
**A2**	39.3	29.5	31.2	NA	1.02
**A3**	10.1	46.3	43.5	NA	3.68
**A4**	20.7	15.3	37	27	0.409
**A5**	21.6	42.6	35.8	NA	6.53
**A6**	25.8	31.9	42.3	NA	0.569
**B1**	7.1	38.1	54.9	NA	7.42
**B2**	35.4	34.6	30	NA	1.007
**B3**	11.3	43.9	44.7	NA	20.8
**B4**	21.7	19.8	38.6	19.9	3.56
**B5**	27.8	33.6	38.7	NA	0.177
**B6**	29.8	30.7	39.4	NA	0.122
**C1**	12.5	36.9	50.7	NA	0.987
**C2**	32.9	38.5	28.6	NA	0.119
**C3**	15.3	31.9	52.7	NA	0.820
**C4**	19.3	23	28.5	29.3	0.219
**C5**	22.9	24.9	52.3	NA	0.077
**C6**	15.2	34.8	50	NA	22.2
**D1**	21.6	39.8	38.6	NA	0.073
**D2**	29.5	47	23.6	NA	0.092
**D3**	20.1	45	34.9	NA	0.334
**D4**	16.1	5.1	19.4	59.4	0.336
**D5**	16.6	48.5	34.9	NA	11.4
**D6**	34	38.8	27.2	NA	0.527

Table S4. Summary of NNLS calculated product ratios including the residual sum of squares (RSS).

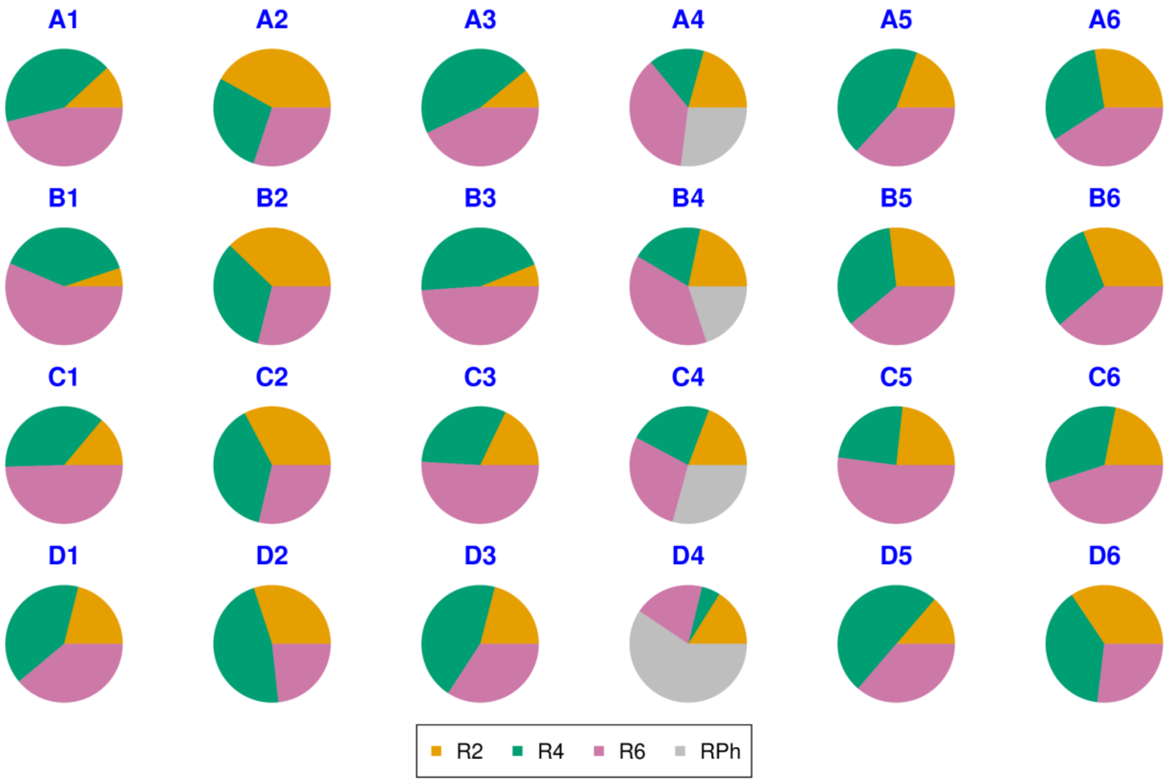

Fig. S130. Pie Chart showing R2:R4:R6:RPh ratios for the 24-well plate using NMR predicted isotopic distributions (**C_{NMR}**). Deconvolution was performed via OLS regression except for wells A4-D4 where NNLS was used.

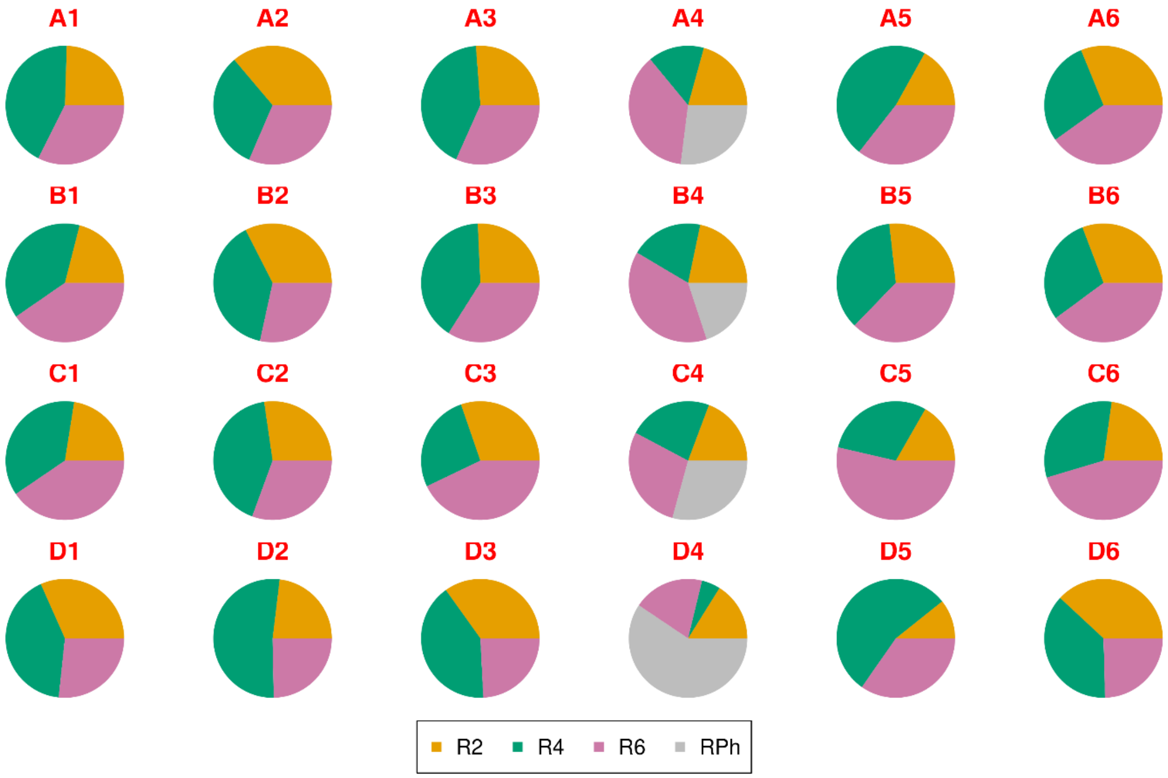

Fig. S131. Pie Chart showing R2:R4:R6:RPh ratios for the 24-well plate using experimentally observed LC distributions (**C**$_{LC}$). Deconvolution was performed via OLS regression except for wells A4-D4 where NNLS was used.

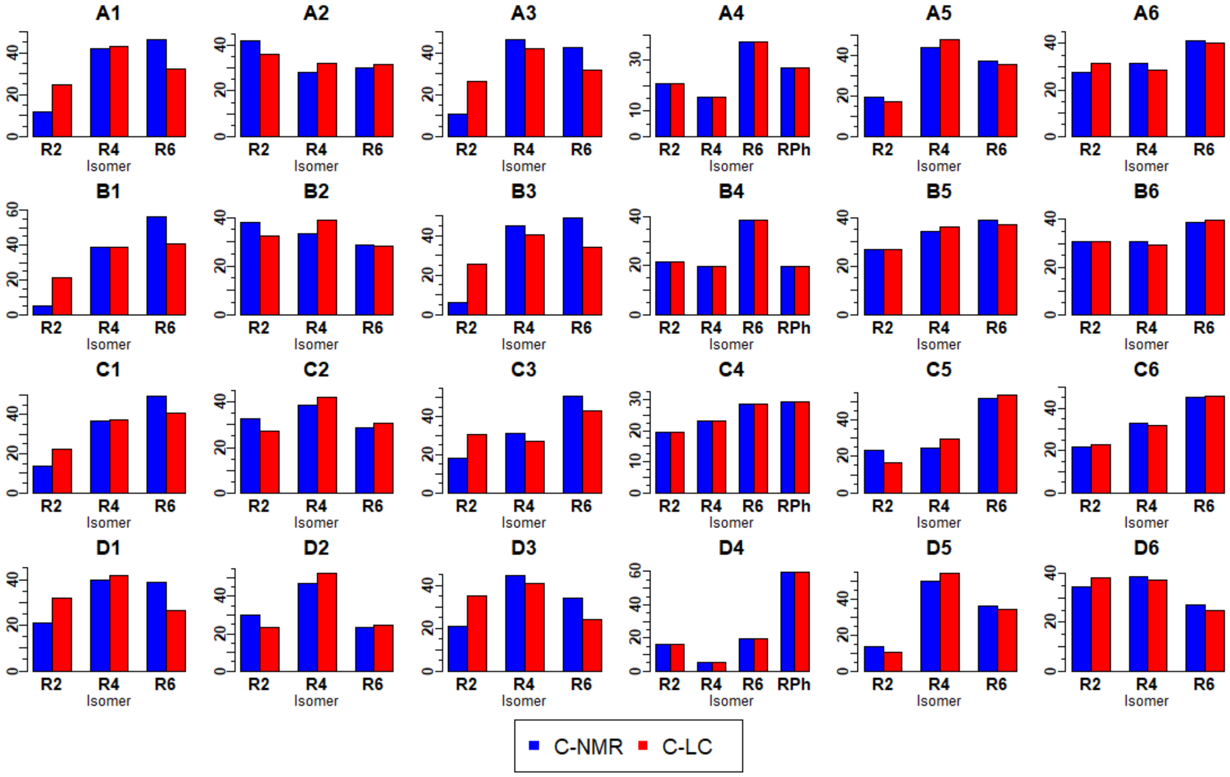

Fig. S132. Comparison of R2:R4:R6:RPh product ratios between C_{NMR} values and C_{LC} values.

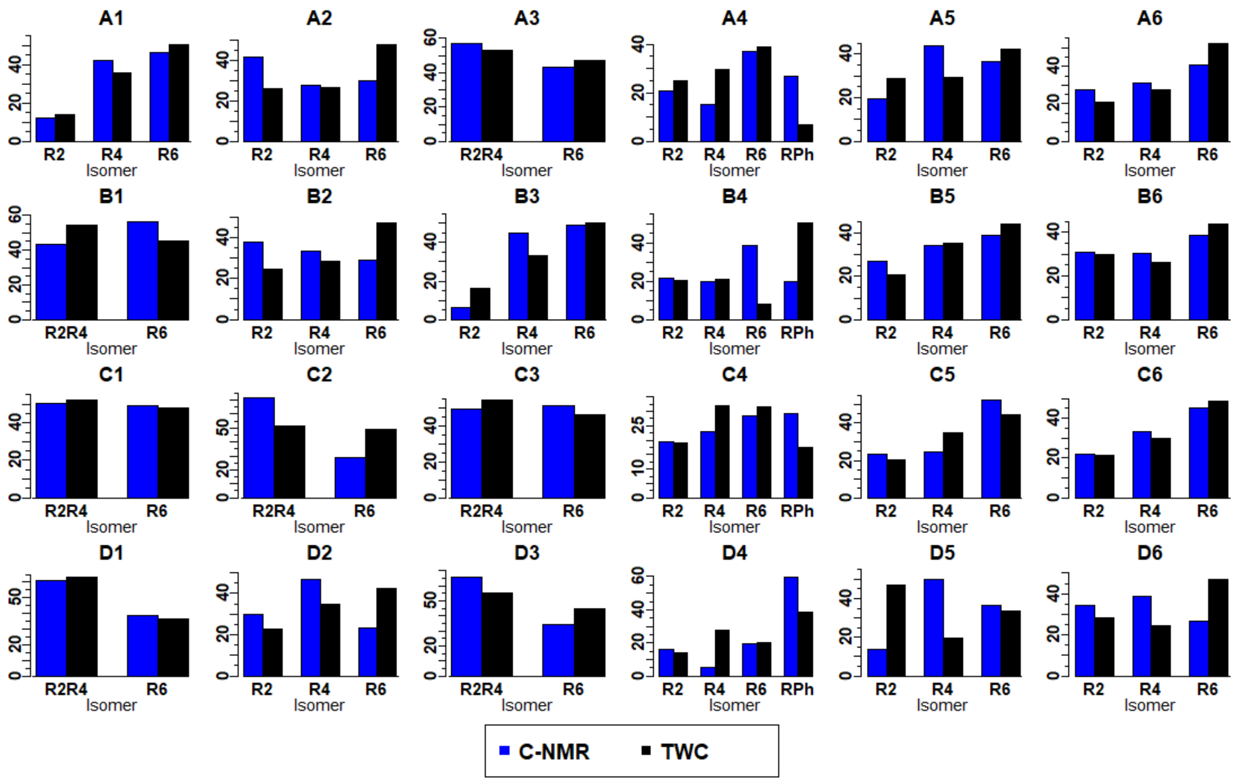

Fig. S133. Comparison of product ratios determined by UPLC separation and total wavelength chromatography (TWC) vs MS-Only spectral deconvolution using C_{NMR} values. Products that did not separate in the UPLC were compared as sums to the sums of the product percentages from spectral deconvolution even though the latter method generates individual isomer percentages.

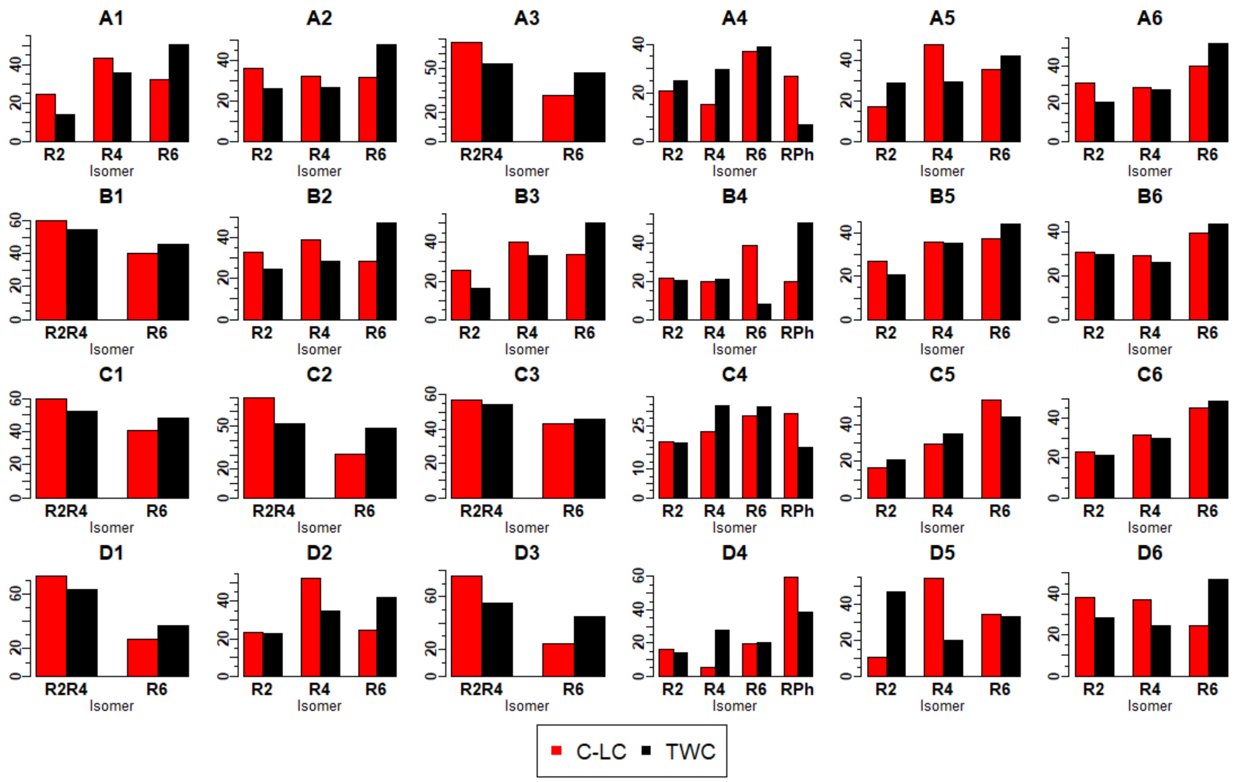

Fig. S134. Comparison of product ratios determined by UPLC separation and total wavelength chromatography (TWC) vs MS-Only spectral deconvolution using C_{LC} values. Products that did not separate in the UPLC were compared as sums to the sums of the product percentages from spectral deconvolution even though the latter method generates individual isomer percentages.

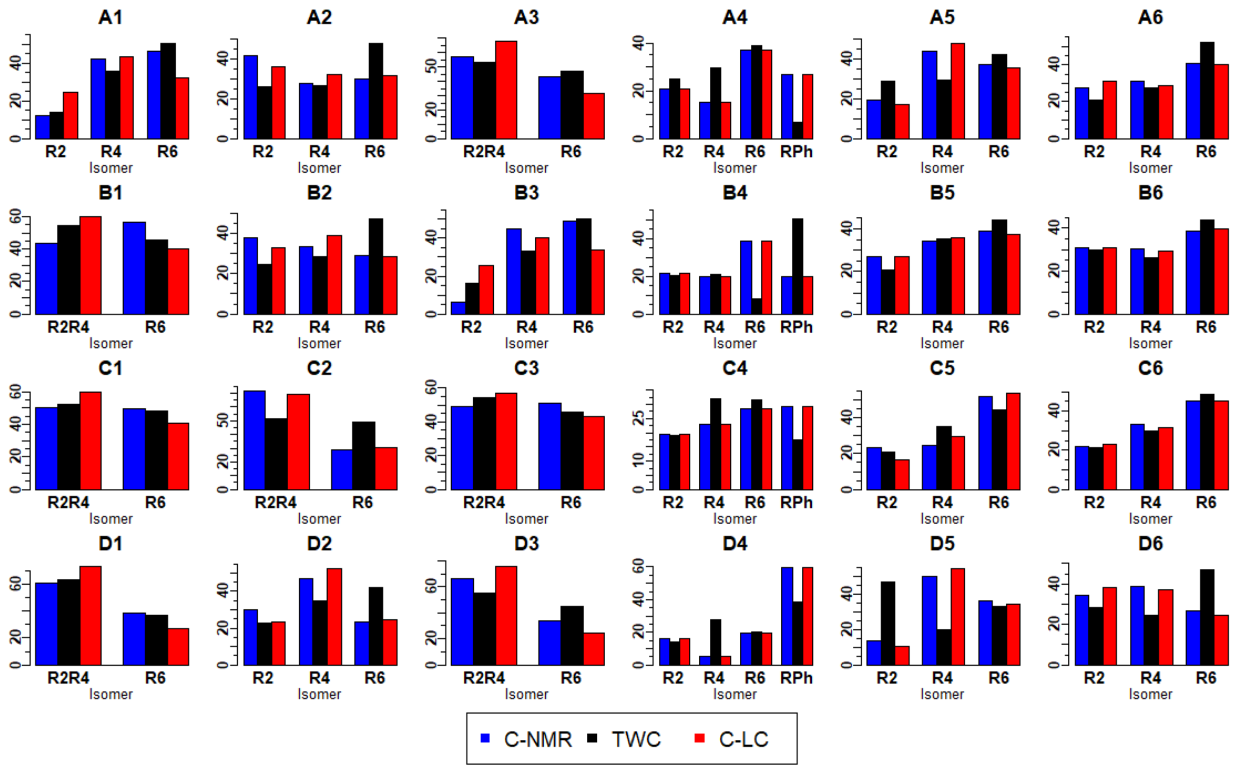

Fig. S135. Comparison of UPLC TWC product ratios with MS-Only C_{NMR} and C_{LC} values.

5.2.4 Validation of Regioisomer Identities in 24-Well Plate

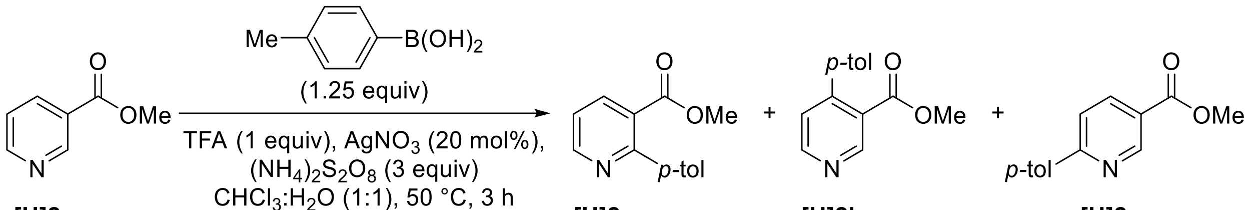

[H]2 **[H]2a** **[H]2b** **[H]2c**

Benchtop scale reaction of A1. To a 20 mL scintillation vial was added methyl nicotinate (**[H]2**) (34.3 mg, 250 µmol) followed by $CHCl_3$ (2.5 mL). To this solution was added TFA (19 µL, 250 µmol, 1.00 equiv) and the solution was left to stir open to air for ~2 min before adding *para*-tolylboronic acid (51.0 mg, 250 µmol, 1.5 equiv), water (2.5 mL), $AgNO_3$ 8.4 mg, 20 mol %), and $(NH_4)_2S_2O_8$ (171 mg, 0.75 mmol, 3.00 equiv). The vial was capped and heated to 50 °C in an aluminum heating block. After 3 h, the reaction was quenched 1 M NaOH (5 mL). The aqueous layer was extracted with $CHCl_3$ (3 x 10 mL) and the combined organic layers were dried over anhydrous Na_2SO_4, filtered, and concentrated. To the resultant mixture was added 1,3,5-Tribromobenzene (26.2 mg, 83.2 µmol, 0.33 equiv) as an internal standard and the mixture was dissolved in $CDCl_3$ for analysis via ^1H NMR spectroscopy. The NMR yields of **[H]2a**, **[H]2b**, and **[H]2c** were 14%, 16%, and 15% respectively.

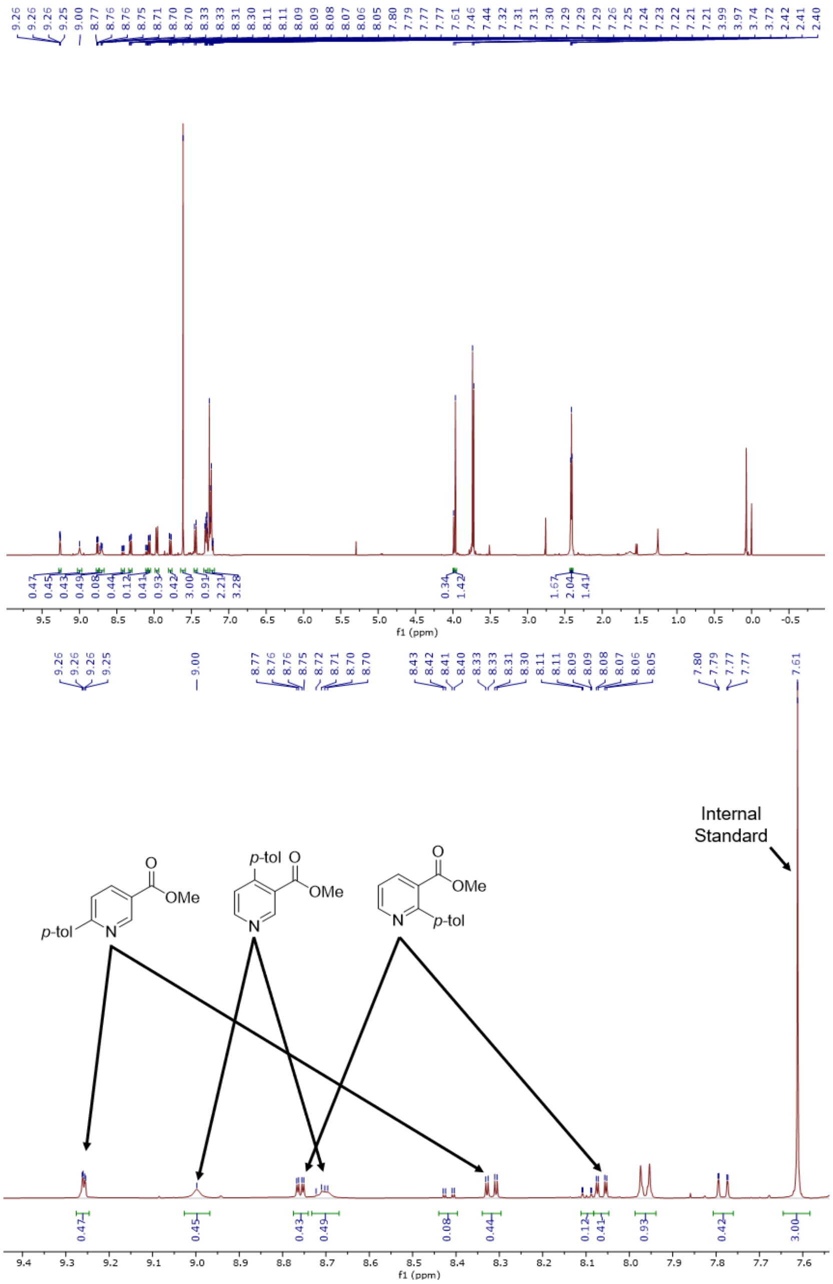

Fig. S136. ^{1}H NMR (600 MHz, $CDCl_3$) of reaction mixture of methyl 2-nicotinate (**[H]2**) with *para*-tolylboronic acid showing internal standard and peaks used to quantitate product amounts. Full width spectrum (top). Bottom: Expansion of aromatic region (bottom).

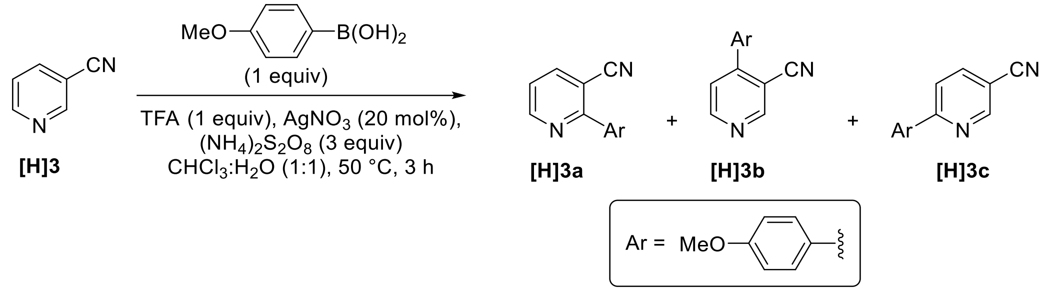

Benchtop scale reaction of B2. To a 20 mL scintillation vial was added 3-cyanopyridine (**[H]3**) (104 mg, 1.00 mmol) followed by $CHCl_3$ (5 mL). To this solution was added TFA (77 µL, 1.00 mmol, 1.00 equiv) and the solution was left to stir open to air for ~2 min before adding 4-methoxyphenylboronic acid (152 mg, 1.00 mmol, 1.00 equiv), water (5 mL), $AgNO_3$ (34 mg, 0.20 mmol, 20 mol%), and $(NH_4)_2S_2O_8$ (685 mg, 3.00 mmol, 3.00 equiv). The vial was capped and heated to 50 °C in an aluminum heating block. After 3 h, the reaction was quenched with satd $NaHCO_3$ (10 mL). The aqueous layer was extracted with $CHCl_3$ (3 x 10 mL) and the combined organic layers were dried over anhydrous Na_2SO_4, filtered, and concentrated. To the resultant mixture was added 1,4-dimethoxybenzene (34.5 mg, 0.250 mmol) as an internal standard and the mixture was dissolved in $CDCl_3$ for analysis via ^{1}H NMR spectroscopy. The NMR yields of **[H]3a**, **[H]3b**, and **[H]3c** were 31%, 20%, and 17%, respectively. Following this analysis, the material was purified via column chromatography using 20-40% EtOAc in hexanes to yield **[H]3a** (24.1 mg, 0.115 mmol, 11%), **[H]3b** (30.5 mg, 0.145 mmol, 14%), and **[H]3c** (29.0 mg, 0.138 mmol, 14%) all as light yellow amorphous solids.

[H]3a:
^{1}H NMR (400 MHz, $CDCl_3$) δ 8.83 (d, *J* = 4.7 Hz, 1H), 8.03 (d, *J* = 7.8 Hz, 1H), 7.93 (d, *J* = 8.4 Hz, 2H), 7.30 (dd, *J* = 7.9, 4.8 Hz, 1H), 7.04 (d, *J* = 8.5 Hz, 2H), 3.88 (s, 3H).

^{13}C{^{1}H} NMR (101 MHz, $CDCl_3$) δ 161.5, 160.6, 152.7, 142.0, 130.6, 129.8, 121.0, 118.1, 114.2, 106.8, 55.5.

[H]3b:
^{1}H NMR (400 MHz, $CDCl_3$) δ 8.91 (s, 1H), 8.76 (d, *J* = 5.3 Hz, 1H), 7.61 (d, *J* = 8.8 Hz, 2H), 7.45 (d, *J* = 5.2 Hz, 1H), 7.06 (d, *J* = 8.8 Hz, 2H), 3.88 (s, 3H).

^{13}C{^{1}H} NMR (101 MHz, $CDCl_3$) δ 161.5, 154.2, 152.7, 152.0, 130.0, 127.7, 123.5, 117.2, 114.8, 108.2, 55.6.

[H]3c:
^{1}H NMR (400 MHz, $CDCl_3$) δ 8.89 (dd, *J* = 2.2, 0.8 Hz, 1H), 8.02 (d, *J* = 8.9 Hz, 2H), 7.95 (dd, *J* = 8.4, 2.2 Hz, 1H), 7.78 (dd, *J* = 8.4, 0.9 Hz, 1H), 7.02 (d, *J* = 8.9 Hz, 2H), 3.89 (s, 3H).

^{13}C{^{1}H} NMR (101 MHz, $CDCl_3$) δ 161.9, 160.2, 152.5, 139.8, 130.0, 129.0, 119.2, 117.4, 114.6, 107.0, 55.6.

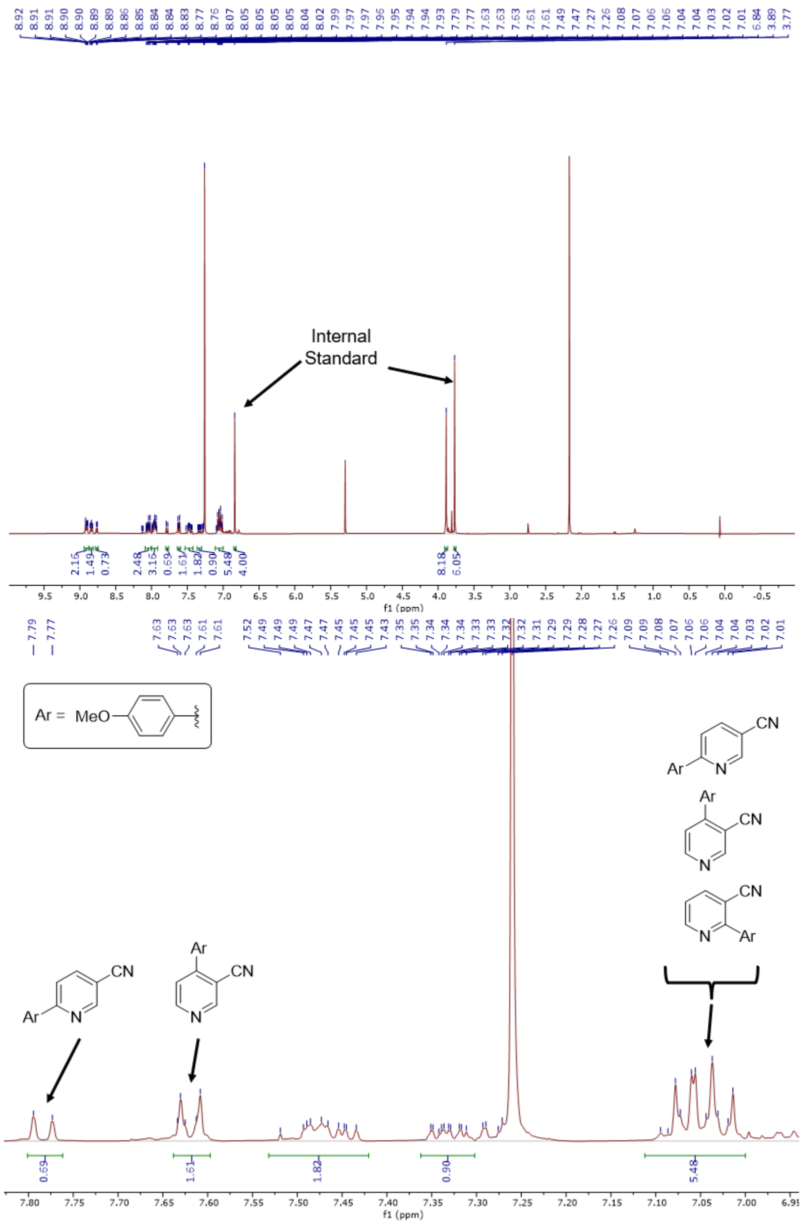

Fig. S137. ^{1}H NMR (600 MHz, $CDCl_3$) of reaction mixture of 3-cyanopyridine (**[H]3**) with *para*-methoxytolylboronic acid showing internal standard and peaks used to quantitate product amounts. Full width spectrum (top). Expansion of aromatic region (bottom).

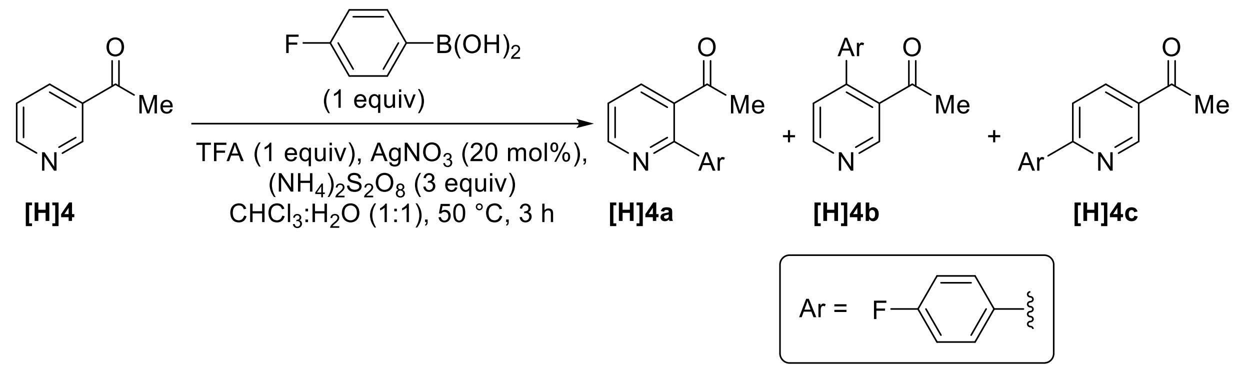

Benchtop scale reaction of C3. To a 20 mL scintillation vial was added 3-acetylpyridine (**[H]4**) (121 mg, 1.00 mmol) followed by $CHCl_3$ (5 mL). To this solution was added TFA (77 µL, 1.00 mmol, 1.00 equiv) and the solution was left to stir open to air for ~2 min before adding 4-fluorophenylboronic acid (140 mg, 1.00 mmol, 1.00 equiv), water (5 mL), $AgNO_3$ (34 mg, 0.20 mmol, 20 mol%), and $(NH_4)_2S_2O_8$ (685 mg, 3.00 mmol, 3.00 equiv). The vial was capped and heated to 50 °C in an aluminum heating block. After 3 h, the reaction was quenched with satd $NaHCO_3$ (10 mL). The aqueous layer was extracted with $CHCl_3$ (3 x 10 mL) and the combined organic layers were dried over anhydrous Na_2SO_4, filtered, and concentrated. To the resultant mixture was added 1,4-dimethoxybenzene (34.5 mg, 0.250 mmol) as an internal standard and the mixture was dissolved in $CDCl_3$ for analysis via ^{1}H NMR spectroscopy. The NMR yields of **[H]4a**, **[H]4b**, and **[H]4c** were 16%, 19%, and 37%, respectively. Following this analysis the material was purified via column chromatography using 10-40% EtOAc in hexanes to yield **[H]4a** (23.8 mg, 0.111 mmol, 11%) as a yellow oil, **[H]4b** (25.5 mg, 0.118 mmol, 12%) as a an off-white solid, and **[H]4c** (27.5 mg, 0.128 mmol, 12%) as a white solid.

[H]4a

^{1}H NMR (400 MHz, $CDCl_3$) δ 8.76 (dd, *J* = 4.8, 1.7 Hz, 1H), 7.87 (dd, *J* = 7.8, 1.7 Hz, 1H), 7.57 (dd, $^3J_{HH}$ = 8.7, $^4J_{HF}$ = 5.3 Hz, 2H), 7.37 (dd, *J* = 7.8, 4.8 Hz, 1H), 7.18 (dd, $^3J_{HH}$ = 8.6, $^3J_{HF}$ = 8.6 Hz, 2H), 2.11 (s, 3H).

^{13}C NMR{^{1}H} (101 MHz, $CDCl_3$) δ 203.4, 163.7 (d, *J* = 249.9 Hz), 156.0, 151.0, 136.4, 136.3, 135.8 (d, *J* = 3.3 Hz), 131.1 (d, *J* = 8.4 Hz), 122.1, 116.0 (d, *J* = 21.8 Hz), 30.3.

^{19}F NMR (376 MHz, $CDCl_3$) δ –111.5.

[H]4b

^{1}H NMR (400 MHz, $CDCl_3$) δ 8.77 (s, 1H), 8.72 (d, *J* = 5.1 Hz, 1H), 7.38 – 7.27 (m, 3H), 7.18 (dd, $^3J_{HH}$ = 8.5, $^3J_{HF}$ = 8.5 Hz, 2H), 2.13 (s, 3H).

^{13}C NMR{^{1}H} (101 MHz, $CDCl_3$) δ 202.0, 163.5 (d, *J* = 250.1 Hz), 151.5, 148.9, 147.1, 135.7, 134.0 (d, *J* = 3.6 Hz), 130.3 (d, *J* = 8.3 Hz), 124.5, 116.3 (d, *J* = 21.8 Hz), 30.5.

^{19}F NMR (376 MHz, $CDCl_3$) δ –111.0.

[H]4c

^{1}H NMR (400 MHz, $CDCl_3$) δ 9.22 (dd, *J* = 2.3, 0.6 Hz, 1H), 8.30 (dd, *J* = 8.3, 2.3 Hz, 1H), 8.08 (dd, $^3J_{HH}$ = 8.9, $^4J_{HF}$ = 5.4 Hz, 2H), 7.81 (dd, *J* = 8.4, 0.7 Hz, 1H), 7.19 (dd, $^3J_{HH}$ = 8.6, $^3J_{HF}$ = 8.6 Hz, 2H), 2.67 (s, 3H).

^{13}C NMR{^{1}H} (101 MHz, $CDCl_3$) δ 196.4, 164.2 (d, *J* = 250.7 Hz), 159.9, 150.2, 136.6, 134.4 (d, *J* = 3.2 Hz), 130.6, 129.4 (d, *J* = 8.7 Hz), 119.8, 116.0 (d, *J* = 21.7 Hz), 26.8.

^{19}F NMR (376 MHz, $CDCl_3$) δ –110.9.

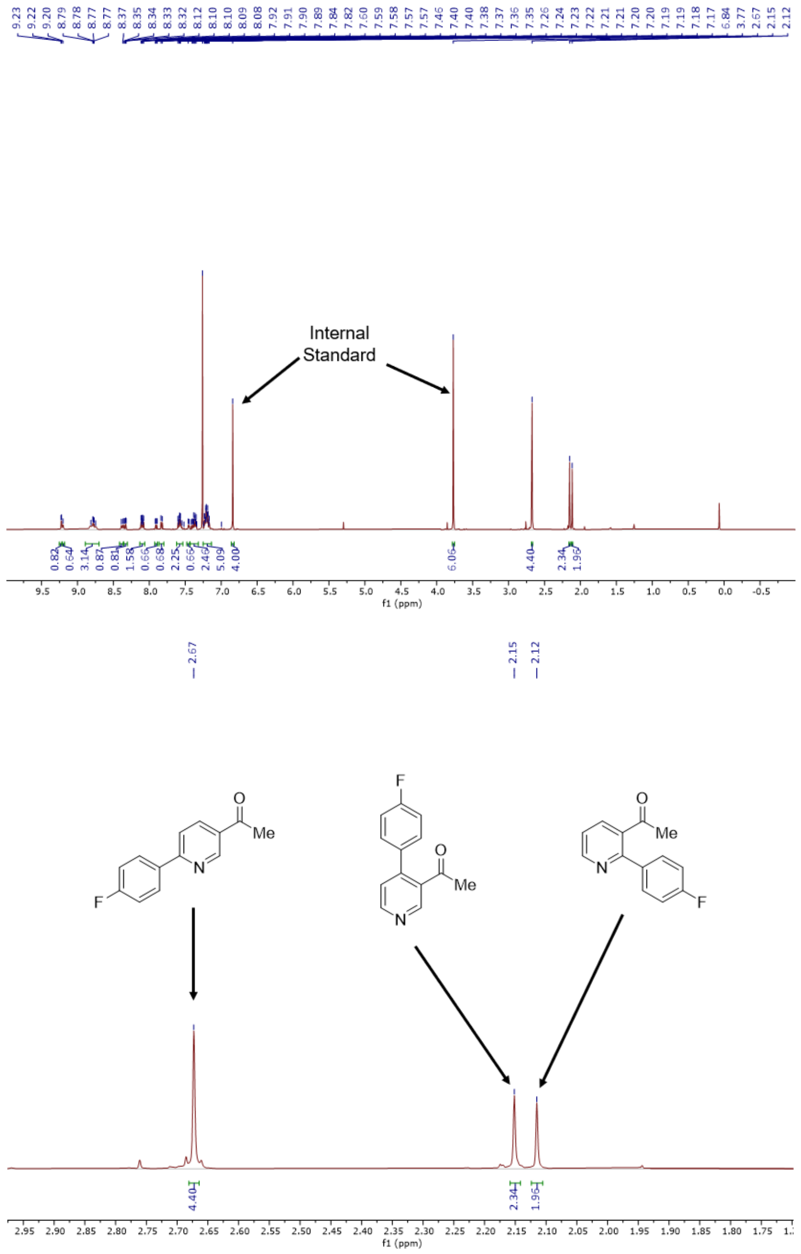

Fig. S138. ^{1}H NMR (600 MHz, $CDCl_3$) of reaction mixture of 3-acetylpyridine ([H]4) with *para*-methoxytolylboronic acid showing internal standard and peaks used to quantitate product amounts. Full width spectrum (top). Expansion of aromatic region (bottom).

6. Synthesis of Deuterated of Arenes

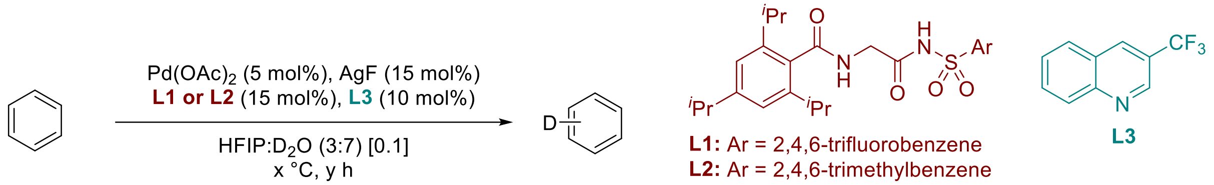

General Procedure A using $Pd(OAc)_2$. A 10 mL microwave vial was charged with: Protio substrate (1.0 equiv), $Pd(OAc)_2$ (5 mol%), AgF (15 mol%), **L1** or **L2** (15 mol%), and **L3** (10 mol%). HFIP:D_2O (3:7) was added to the vial to create a 0.1 M solution with respect to the substrate. The vial was heated at the indicated temperature using an aluminum heating block. After sufficient deuterium incorporation was achieved, the reaction mixture was allowed to cool to rt and was concentrated. The resultant material was chromatographed to obtain pure material. Small scale deuterations were performed initially and were monitored by taking aliquots of the mixture at multiple time points. Aliquots are directly concentrated and analyzed by NMR to monitor deuterium incorporation over time. This data was used to define the reaction time for a subsequent reaction performed at a larger scale.

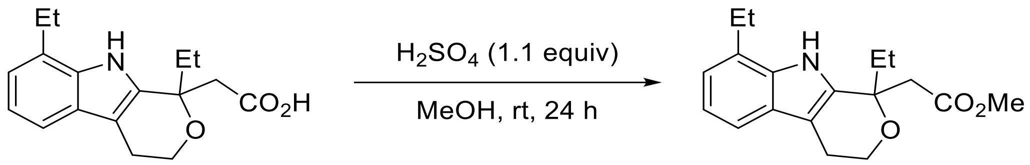

H-Etodolac methyl ester ([H]8). To a dry 250 mL rbf was added etodolac (2.30 g, 8.00 mmol) followed by MeOH (40 mL). With rapid stirring H_2SO_4 (9 drops, ~450 µL, 8.4 mmol, 1.1 equiv) was added and the solution was stirred at rt. After 24 h the reaction was deemed complete by TLC and satd $NaHCO_3$ (25 mL) was added to the flask slowly to avoid excessive foaming. The solution was concentrated to remove the MeOH and EtOAc (150 mL) was added. The aqueous layer was removed, and the organic layer was washed with satd $NaHCO_3$ (3 x 25 mL) followed by satd NaCl (50 mL). The organic phase was dried using anhydrous Na_2SO_4, filtered, and concentrated to yield pure **H-etodolac methyl ester** (**[H]8**) as an off-white powder (2.20 g, 7.30 mmol, 91%).

^{1}H NMR (600 MHz, $CDCl_3$) δ 9.06 (s, 1H), 7.37 (d, J = 7.7 Hz, 1H), 7.06 (t, J = 7.5 Hz, 1H), 7.01 (d, J = 7.2 Hz, 1H), 4.05 (ddd, J = 11.2, 4.8, 4.8 Hz, 1H), 3.94 (ddd, J = 11.6, 7.6, 4.3 Hz, 1H), 3.02 (d, J = 16.7 Hz, 1H), 2.93 (d, J = 16.5 Hz, 1H), 2.93 – 2.81 (m, 3H), 2.75 (ddd, J = 15.2, 4.5, 4.5 Hz, 1H), 2.17 (dq, J = 14.8, 7.4 Hz, 1H), 2.01 (dq, J = 14.6, 7.3 Hz, 1H), 1.38 (t, J = 7.6 Hz, 3H), 0.84 (t, J = 7.4 Hz, 3H).

^{13}C{^{1}H} NMR (151 MHz, $CDCl_3$) δ 173.4, 136.0, 134.6, 126.7, 126.3, 120.5, 119.7, 116.1, 108.5, 74.7, 60.8, 52.1, 42.9, 30.8, 24.3, 22.5, 13.9, 7.7.

Spectral data are of commercial material and agree with those reported.[1]

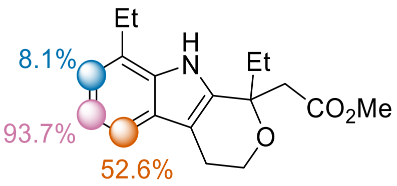

D-Etodolac methyl ester ([D]8). Following general procedure **A** using **H-etodolac methyl ester** (151 mg, 0.500 mmol), $Pd(OAc)_2$ (5.6 mg, 0.025 mmol, 5 mol %), AgF (9.2 mg, 0.073 mmol, 15 mol %), **L1** (37.4 mg, 0.0750 mmol, 15 mol %), **L3** (9.9 mg, 0.050 mmol, 10 mol%), and HFIP:D_2O (1.5 mL : 3.5 mL). The mixture was heated to 80 °C for 3 h. The material was concentrated and chromatographed using 0-10% EtOAc in hexanes to yield pure **D-etodolac methyl ester** (**[D]8**) as an off-white solid (125 mg, 0.415 mmol, 83%).

^{1}H NMR (600 MHz, d1=30s, $CDCl_3$) δ 9.05 (s, 1H), 7.36 (s, 0.474H), 7.08 – 7.03 (m, 0.063H), 7.01 (s, 0.919H), 4.04 (ddd, *J* = 11.4, 4.8, 4.8 Hz, 1H), 3.94 (ddd, *J* = 11.6, 7.6, 4.3 Hz, 1H), 3.72 (s, 3H), 3.02 (d, *J* = 16.6 Hz 1H), 2.92 (d, *J* = 16.6 Hz 1H), 2.90 – 2.80 (m, 3H), 2.75 (ddd, *J* = 15.2, 4.5, 4.5 Hz, 1H), 2.16 (dq, *J* = 14.8, 7.4 Hz, 1H), 2.01 (dq, *J* = 14.6, 7.3 Hz, 1H), 1.37 (t, *J* = 7.6 Hz, 3H), 0.83 (t, *J* = 7.4 Hz, 3H).

^{13}C{^{1}H} NMR (151 MHz, $CDCl_3$) δ 173.4, 136.0, 134.6, 126.74 – 126.66 (m, adjacent), 126.3 – 126.2 (m, adjacent), 120.5 – 120.4 (m, *labeled*), 119.7 – 119.2 (m, labeled), 116.1, 116.0, 115.7 (t, 24.1 Hz) (m, labeled), 108.6 – 108.5 (m, adjacent), 74.7, 60.8, 52.1, 42.9, 30.8, 24.34 – 24.28 (m, *adjacent*), 22.56 – 22.54 (m, *adjacent*), 13.9, 7.7.

MS (ESI, SQ): loop injection, concentration 100 µM, injection vol 1 µL, cone voltage 30 V.

Isopat: 1.494 D (NMR: 1.544 D)

%D Calculation: Method 2.1.

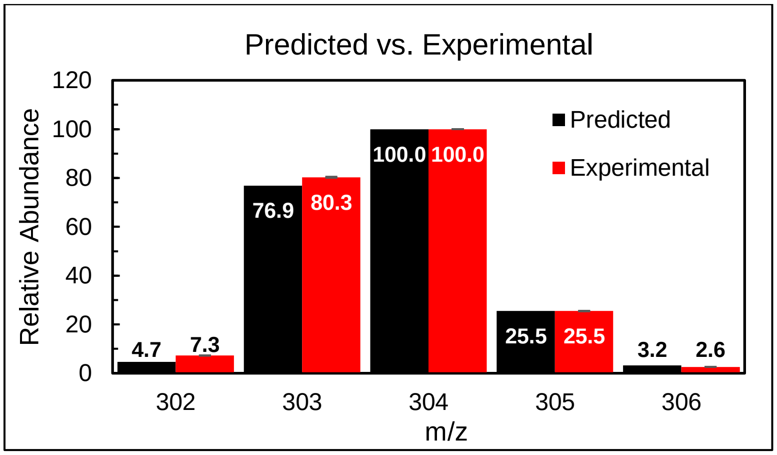

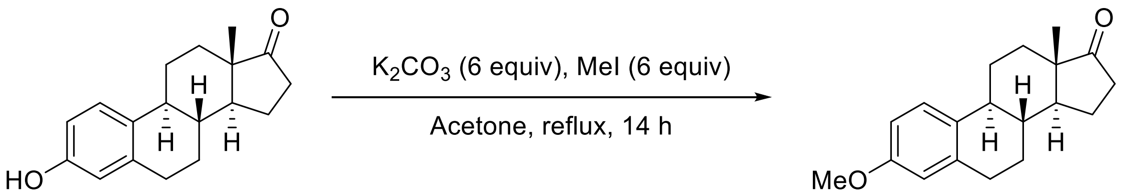

H-Estrone methyl ether ([H]9). To a dry 25 mL rbf was added estrone (0.200 g, 0.740 mmol) and K_2CO_3 followed by anhydrous acetone (7 mL). Methyl iodide (0.28 mL, 4.4 mmol) was added in one portion and the mixture was heated to reflux using an oil bath at 65 °C. After 14 h, the reaction was deemed complete by TLC and was allowed to cool to rt. After cooling, 1 M NaOH (10 mL) was added, and the mixture left to stir for 10 min. The resultant mixture was diluted with $CHCl_3$ (100 mL) and the aqueous layer removed. The organic phase was washed with 1 M NaOH (20 mL) and satd NaCl (20 mL), dried with anhydrous Na_2SO_4, filtered, and concentrated. The resultant material was chromatographed using 5-20% EtOAc in hexanes to yield pure **H-estrone methyl ether** (**[H]9**) as a white solid (0.190 g, 0.670 mmol, 91%).

^{1}H NMR (600 MHz, $CDCl_3$) δ 7.21 (d, *J* = 8.5 Hz, 1H), 6.72 (dd, *J* = 8.6, 2.9 Hz, 1H), 6.65 (d, *J* = 2.7 Hz, 1H), 3.78 (s, 3H), 3.05 – 2.78 (m, 2H), 2.50 (ddd, *J* = 19.1, 8.8, 1.1 Hz, 1H), 2.43 – 2.34 (m, 1H), 2.26 (td, *J* = 10.4, 4.5 Hz, 1H), 2.14 (dt, *J* = 19.1, 9.0 Hz, 1H), 2.09 – 1.98 (m, 2H), 1.98 – 1.92 (m, 1H), 1.68 – 1.39 (m, 6H), 0.91 (s, 3H).

^{13}C{^{1}H} NMR (151 MHz, $CDCl_3$) δ 221.1, 157.7, 137.9, 132.2, 126.5, 114.0, 111.7, 55.4, 50.6, 48.2, 44.1, 38.5, 36.0, 31.7, 29.8, 26.7, 26.1, 21.7, 14.0.

Spectral data are of commercial material and agree with those reported.[1]

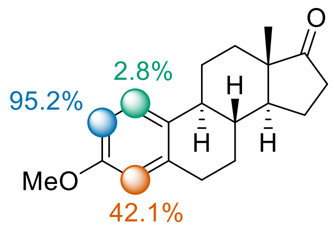

D-Estrone methyl ether ([D]9. Following general procedure **A** using **H-estrone methyl ether** (62.3 mg, 0.219mmol), $Pd(OAc)_2$ (2.5 mg, 0.011 mmol, 5 mol %), AgF (4.2 mg, 0.033 mmol, 15 mol %), **L1** (16.3 mg, 0.0329 mmol, 15 mol %), **L3** (4.3 mg, 0.022 mmol, 10 mol%), and HFIP:D_2O (0.6 mL : 1.5 mL). The mixture was heated to 90 °C for 1.5 h. The material was concentrated and chromatographed using 5-20% EtOAc in hexanes to yield pure **D-estrone methyl ether** (**[D]9**) as a white solid (32.3 mg, 0.114 mmol, 52%).

^{1}H NMR (600 MHz, d1=30s, $CDCl_3$) δ 7.21 (s, 0.972H), 6.72 (d, *J* = 8.9 Hz, 0.048H), 6.65 (s, 0.579H), 3.78 (s, 3H), 2.96 – 2.85 (m, 2H), 2.50 (dd, *J* = 19.1, 8.8 Hz, 1H), 2.43 – 2.34 (m, 1H), 2.26 (td, J = 11.3 Hz, 4.7 Hz, 1H), 2.14 (dt, *J* = 18.9, 9.0 Hz, 1H), 2.09 – 1.98 (m, 2H), 1.98 – 1.90 (m, 1H), 1.68 – 1.39 (m, 6H), 0.91 (s, 3H).

^{13}C{^{1}H} NMR (151 MHz, $CDCl_3$) δ 221.1, 157.73 – 157.65 (m, *adjacent*), 137.9 – 137.8 (m, *adjacent*), 132.15 – 132.14 (m, *adjacent*), 126.5 – 126.4 (m, *labeled*), 114.1, 114.0, 113.7 (t, 23.9 Hz), 111.75, 111.71, 111.44 (t, 24.2 Hz), 111.40 (t, 24.2 Hz), 55.4, 50.6, 48.2, 44.1, 38.5, 36.0, 31.7, 29.8 – 29.7 (m, *adjacent*), 26.7, 26.1, 21.7, 14.0.

MS (ESI, SQ): loop injection, concentration 100 µM, injection vol 1 µL, cone voltage 30 V.

Isopat: 1.337 D (NMR: 1.401 D)

%D Calculation: Method 2.1.

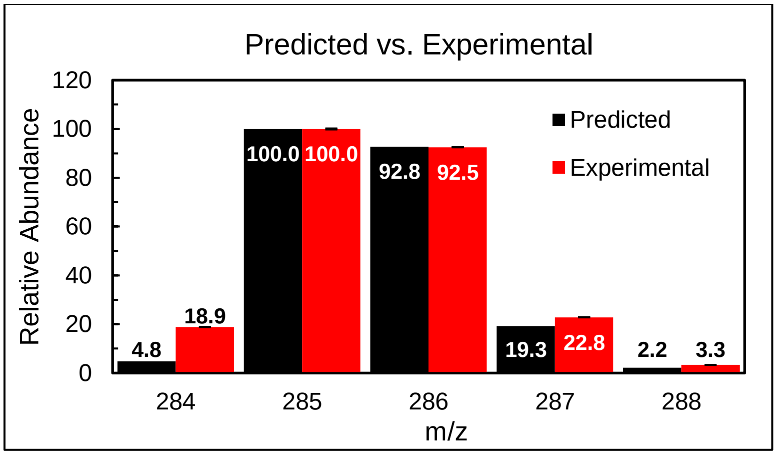

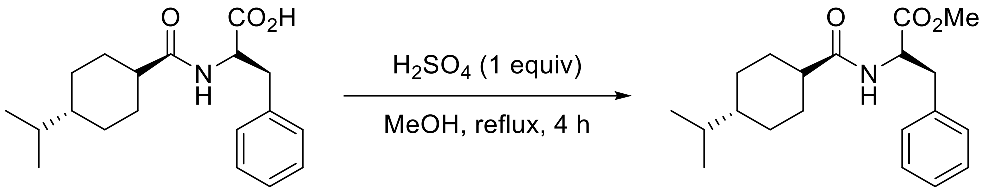

H-Nateglinide methyl ester. To a dry 100 mL rbf was added nateglinide (1.50 g, 4.72 mmol) followed by MeOH (25 mL). With rapid stirring H_2SO_4 (5 drops, ~250 µL, 4.7 mmol, 1 equiv) was added. A reflux condenser was attached and the mixture heated to reflux using an oil bath. After 4 h ,the reaction was deemed complete by TLC and the mixture was allowed to cool to rt before adding satd $NaHCO_3$ (25 mL) slowly to avoid excessive foaming. The solution was concentrated to remove the MeOH and subsequently EtOAc (100 mL) was added. The aqueous layer was removed, and the organics washed with satd $NaHCO_3$ (3 x 15 mL) followed by satd NaCl (25 mL). The organic phase was dried using anhydrous Na_2SO_4, filtered, and concentrated to yield pure **H-nateglinide methyl ester** as a white fluffy powder (1.46 g, 4.41 mmol, 93%).

^{1}H NMR (600 MHz, C_6D_6) δ 7.13 – 7.09 (m, 2H), 7.07 – 7.04 (m, 1H), 7.04 – 7.00 (m, 2H), 5.68 (d, *J* = 7.7 Hz, 1H), 5.05 (dt, *J* = 7.7, 5.7, 5.7 Hz, 1H), 3.23 (s, 3H), 3.18 (dd, *J* = 13.8, 6.0 Hz, 1H), 2.98 (dd, *J* = 13.8, 5.5 Hz, 1H), 1.85 – 1.69 (m, 2H), 1.58 – 1.44 (m, 5H), 1.30 – 1.20 (m, 1H), 0.91 – 0.81 (m, 1H), 0.79 (d, *J* = 6.8 Hz, 6H), 0.75 – 0.65 (m, 2H).

^{13}C{^{1}H} NMR (151 MHz, $CDCl_3$) δ 175.7, 172.4, 136.1, 129.5, 128.6, 127.2, 52.8, 52.4, 45.6, 43.4, 38.0, 32.9, 29.9, 29.6, 29.1, 29.0, 19.9.

Spectral data are of commercial material and agree with those reported.[1]

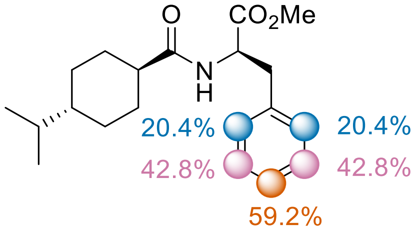

D-Nateglinide methyl ester ([D]10). Following general procedure **XXXX** using **H-nateglinide methyl ester** (249 mg, 0.750 mmol), $Pd(OAc)_2$ (8.4 mg, 0.037 mmol, 5 mol %), AgF (14.3 mg, 0.113 mmol, 15 mol %), **L2** (54.8 mg, 0.113 mmol, 15 mol %), **L3** (14.8 mg, 0.075 mmol, 10 mol%), and HFIP:D_2O (2.25 mL : 5.25 mL). The mixture was heated to 80 °C for 16 h. The material was concentrated and chromatographed using 5-15% EtOAc in hexanes to yield pure **D-nateglinide methyl ester** (**[D]10**) as a white solid (225 mg, 0.678 mmol, 90%).

^{1}H NMR (600 MHz, d1=30s, C_6D_6) δ 7.13 – 7.09 (m, 1.22H), 7.07 – 7.02 (m, 0.41H) 7.04 – 7.00 (m, 1.74H), 5.68 (d, J = 6.7 Hz, 1H), 5.05 (dt, J = 7.7, 5.7 Hz, 1H), 3.23 (s, 3H), 3.18 (dd, J = 13.8, 6.0 Hz, 1H), 2.98 (dd, J = 13.8, 5.5 Hz, 1H), 1.84 – 1.70 (m, 2H), 1.58 – 1.44 (m, 5H), 1.30 – 1.23 (m, 1H), 0.88 – 0.81 (m, 1H), 0.79 (d, J = 6.8 Hz, 6H), 0.75 – 0.63 (m, 2H)

^{13}C{^{1}H} NMR (151 MHz, $CDCl_3$) δ 175.7, 172.4, 136.0 – 135.9 (m, *adjacent*), 129.4 – 129.0 (m, *labeled*), 128.6 – 127.9 (m, *labeled*), 127.2 – 126.6 (m, *labeled*), 52.8, 52.4, 45.6, 43.3, 38.0 – 37.9 (m, *adjacent*), 32.9, 29.9, 29.6, 29.1, 29.0, 19.8.

MS (ESI, SQ): loop injection, concentration 100 µM, injection vol 1 µL, cone voltage 30 V.

Isopat: 1.869 D (NMR: 1.856 D).

%D Calculation: Method 2.1 & 2.3.

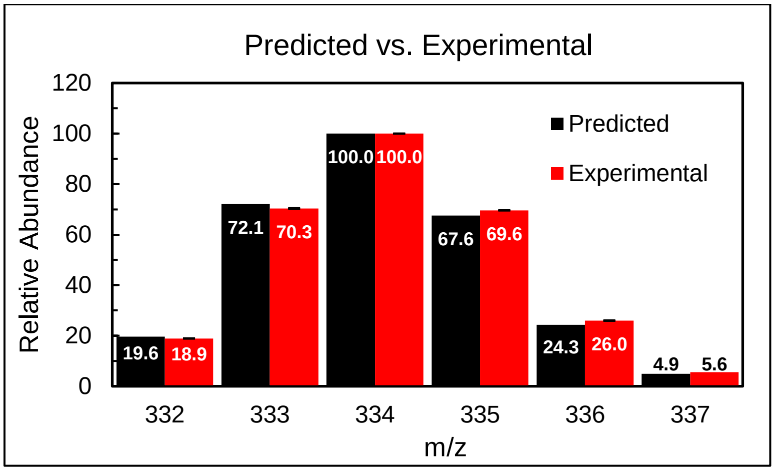

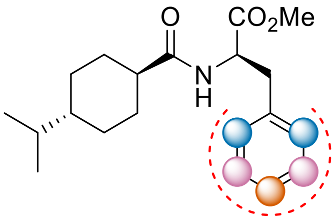

94.4% D (4.72 D)

d_5-Nateglinide methyl ester. Following general procedure **A** using H-nateglinide methyl ester (331 mg, 1.00 mmol), $Pd(OAc)_2$ (22.4 mg, 0.100 mmol, 10 mol %), AgF (38.1 mg, 0.300 mmol, 30 mol %), **L1** (99.7 mg, 0.200 mmol, 20 mol %), **L3** (59.1 mg, 0.300 mmol, 30 mol %), and HFIP:D_2O (2.25 mL : 5.25 mL). The mixture was heated to 80 °C for 16 h. The material was concentrated and chromatographed using 5-15% EtOAc in hexanes to yield pure **d_5-nateglinide methyl ester** as a white solid (282 mg, 0.850 mmol, 85%). The resultant material was resubjected to the same reaction conditions and following chromatography yielded pure **d_5-nateglinide methyl ester** as a white solid (170 mg, 0.512 mmol, 60%). The combined aromatic peaks integrate to 0.28H which corresponds to 94.4% D or 4.72 D incorporated.

^{1}H NMR (600 MHz, Acetone) δ 7.29 (s, 0.111H), 7.23 (d, J = 3.2 Hz, 0.169H), 7.09 (d, J = 8.1 Hz, 1H), 4.71 (td, J = 8.3, 8.2, 5.5 Hz, 1H), 3.67 (s, 3H), 3.14 (dd, J = 13.8, 5.5 Hz, 1H), 2.99 (dd, J = 13.8, 8.4 Hz, 1H), 2.16 – 2.08 (m, 1H), 1.89 – 1.70 (m, 4H), 1.43 – 1.28 (m, 3H), 1.09 – 0.93 (m, 3H), 0.86 (d, J = 6.8 Hz, 6H).

^{13}C{^{1}H} NMR (151 MHz, $CDCl_3$) δ 175.7, 172.3, 135.9 – 135.8 (m, adjacent), 129.3, 129.0 (t, 24.0 Hz), 128.4, 128.1 (t, 24.0 Hz), 127.0, 126.7 (t, 24.1 Hz), 52.8, 52.4, 45.6, 43.3, 37.9, 32.9, 29.9, 29.6, 29.1, 29.0, 19.8.

7. Reactions of Arenes

7.1.1 Bromination of Etodolac Methyl Ester

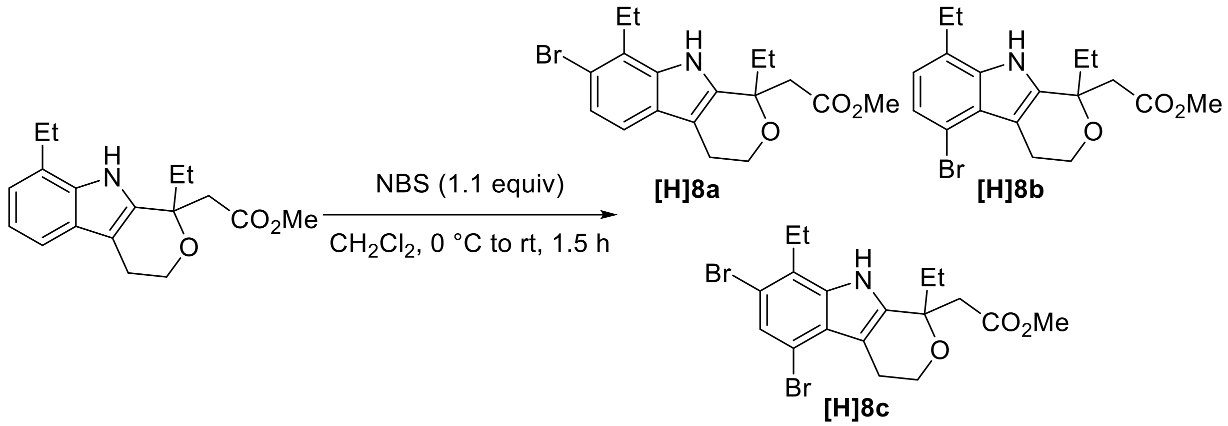

Bromination of H-etodolac methyl ester. To a tapered 10 mL microwave vial was added H-etodolac methyl ester (50.0 mg, 0.166 mmol) followed by CH_2Cl_2 (0.8 mL). The mixture was cooled to 0 °C using an ice bath and NBS (32.5 mg, 0.182 mmol) was added in one portion. After stirring for 30 min at 0 °C the vial was removed from the ice bath and allowed to warm to rt. After 1 h, the reaction was deemed complete by TLC and the mixture was directly concentrated and chromatographed using 0-10% EtOAc in hexanes to yield an inseparable mixture of products **A:B:C** (1:0.36:0.1) as a yellow tinted solid (61.2 mg total). Yields based off NMR ratios: 66% (**[H]8a**), 24% (**[H]8b**), 5% (**[H]8c**).

^{1}H NMR (600 MHz, Acetone) δ 10.21 (s, 0.03H, **[H]8c**), 9.97 (s, 0.24H, **[H]8b**), 9.93 (s, 0.77H, **[H]8a**), 7.36 (s, 0.10H, **[H]8c**), 7.23 (d, J = 8.4 Hz, 1.0H, **[H]8a**), 7.19 (d, J = 8.4 Hz, 1.0H, **[H]8a**), 7.11 (d, J = 7.7 Hz, 0.36H, **[H]8b**), 6.83 (dt, J = 7.7, 0.8 Hz, 0.36H, **[H]8b**), 4.12 – 3.87 (m, 3H, **[H]8a/[H]8b/[H]8c**), 3.62 (s, 1.13H, **[H]8b**), 3.61 (s, 3.35H, **[H]8a**/**[H]8c**), 3.12 (dt, J = 15.6, 3.9 Hz, 0.45H, **[H]8b**), 3.08 – 2.95 (m, 4.46H, **[H]8a/[H]8b/[H]8c**), 2.94 – 2.86 (m, 1.76H, **[H]8a/[H]8b/[H]8c**), 2.86 – 2.73 (m, 2.72H, **A**/**B**/**C**), 2.66 (ddd, J = 15.1, 3.9, 3.4 Hz, 1.07H, **A**), 2.13 – 2.00 (m, 4.79H, **[H]8a/[H]8b/[H]8c**), 1.27 (t, J = 7.6 Hz, 1.59H, **[H]8b**), 1.22 – 1.16 (m, 3.59H, **[H]8a**/**[H]8c**), 0.74 – 0.68 (m, 4.62H, **[H]8a/[H]8b/[H]8c**).

^{13}C{^{1}H} NMR (151 MHz, Acetone) δ 171.5, 171.4, 138.3, 137.9, 136.9, 136.4, 127.3, 127.0, 126.9, 125.9, 124.2, 123.8, 122.0, 118.0, 116.7, 111.5, 109.9, 109.7, 76.14, 76.10, 61.09, 61.06, 61.0, 51.80, 51.78, 51.75, 43.5, 43.4, 43.3, 31.83, 31.80, 30.6, 25.1, 25.0, 24.3, 14.4, 13.8, 13.6, 8.1, 8.03, 7.98.

HRMS (EI, TOF) m/z: $[M]^+$ calculated for $C_{18}H_{22}BrNO_3$ 379.0783, observed 379.0803.

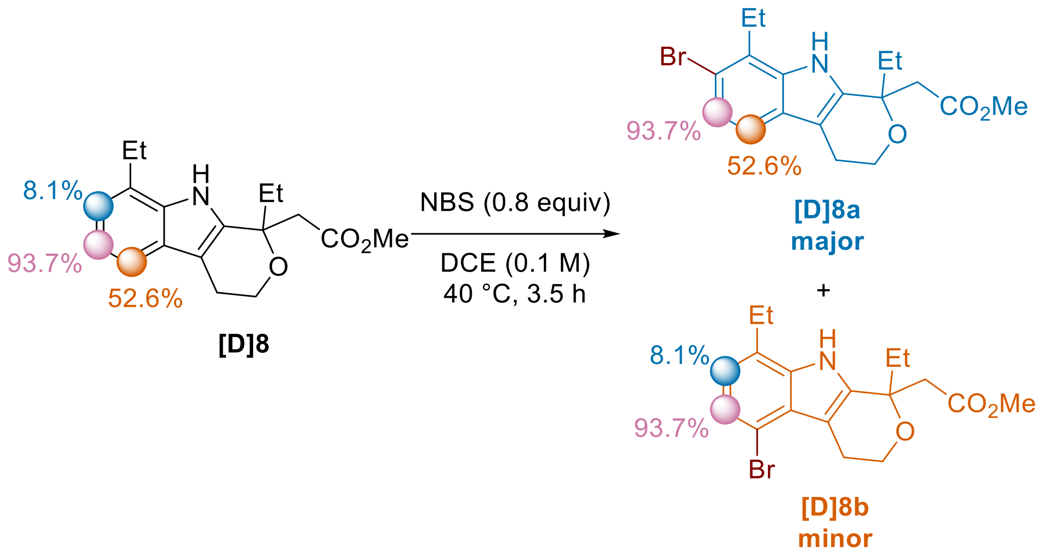

Bromination of D-etodolac methyl ester. D-Etodolac methyl ester (89.1 mg, 0.296 mmol) was dissolved in $ClCH_2CH_2Cl$ (3.2 mL) making a 92.5 mM stock solution. The stock solution (151 µL, 4.2 mg, 0.014 mmol) was added to a 1 mL glass vial (8 x 30 mm) equipped with a stir bar. The solvent was removed with a Genevac (vacuum centrifugation) 1 h cycle at 25 °C. A stock solution was prepared containing NBS (72.2 mg, 0.406 mmol) dissolved in $ClCH_2CH_2Cl$ (5.0 mL) resulting in an 81.2 mM solution. The NBS stock solution (140 µL, 2.0 mg, 0.011 mmol) was added to the glass vial and the glass vial was sealed in an aluminum reaction block and stirred using a tumble stirrer, the heat generated from the tumble stirrer resulted in a reaction temperature of 40 °C. After 3.5 h, the reaction was removed from stirring, concentrated with a Genevac (vacuum centrifugation) 1 h cycle at 25 °C. The reaction mixture was dissolved in MeCN (400 µL) and a portion of this solution of this solution (28.5 µL) was diluted in MeCN (5 mL) resulting in an approximately 200 µM solution (with respect to initial starting material). This solution was then analyzed using a Waters ACQUITY SQ. The reaction was performed in duplicate.

UPLC (Acquity SQ): Premier BEH C18; 2.4 µm; 50mm x 2.1mm; Column T = 40 °C; 0.75 mL/min. Gradient: 0.55 min at 50% MeCN/H_2O (0.1% FA); 3 min gradient to 55% MeCN/H_2O (0.1% FA); 1.85 min gradient to 90% MeCN/H_2O (0.1% FA); 0.05 min gradient to 5% MeCN/H_2O (0.1% FA); 0.1 min at 5% MeCN/H_2O (0.1% FA). Detection: PDA (TWC 210 – 400 nm); MS (ESI+, *m/z* = 375 – 390, 10 V, 20 Hz).

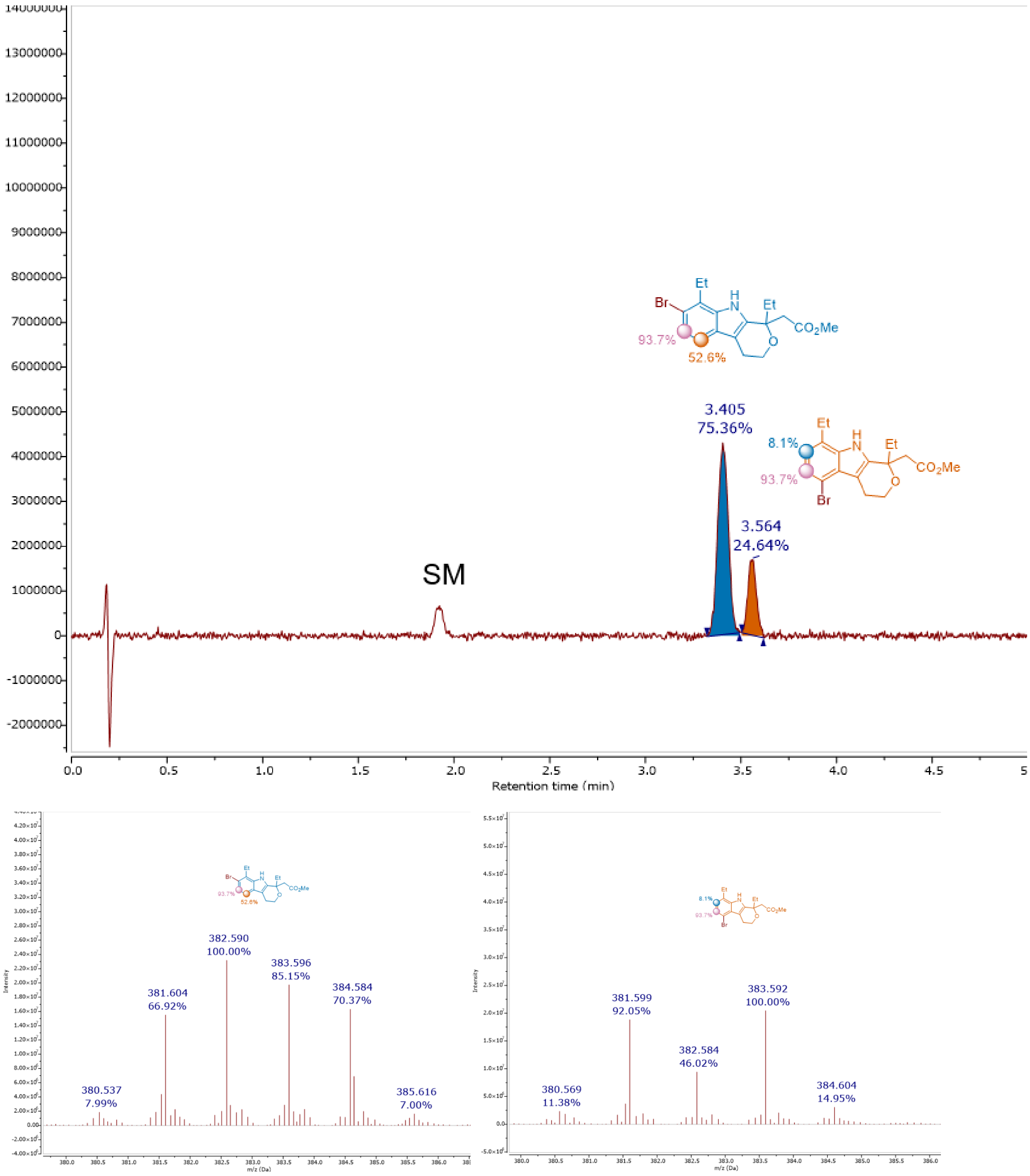

Fig. S139. D-Etodolac methyl ester bromination HPLC trace (top) and isotopic patterns for the of major (bottom left) and minor products (bottom right) (Trial 1).

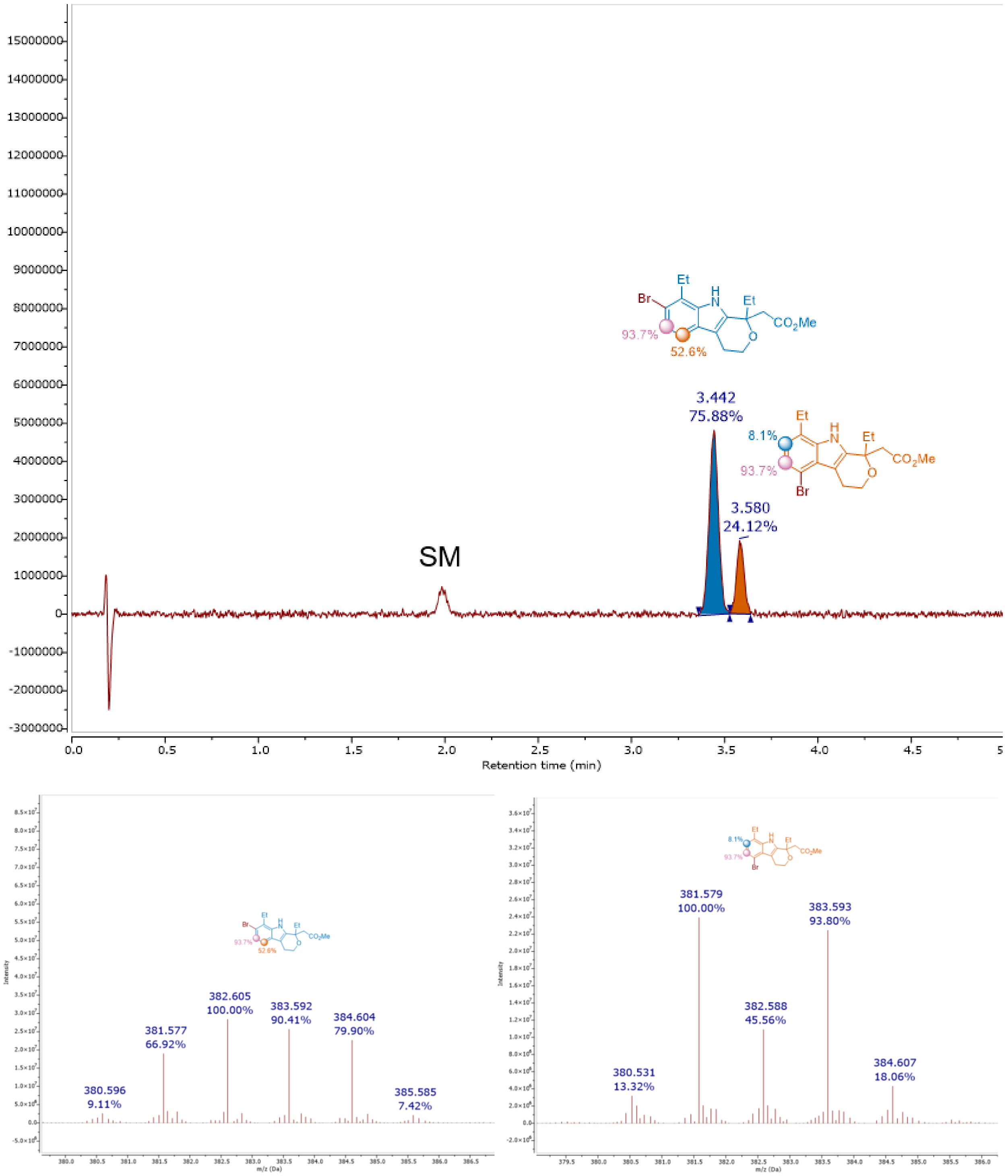

Fig. S140. D-Etodolac methyl ester bromination HPLC trace (top) and isotopic patterns for the of major (bottom left) and minor products (bottom right) (Trial 2).

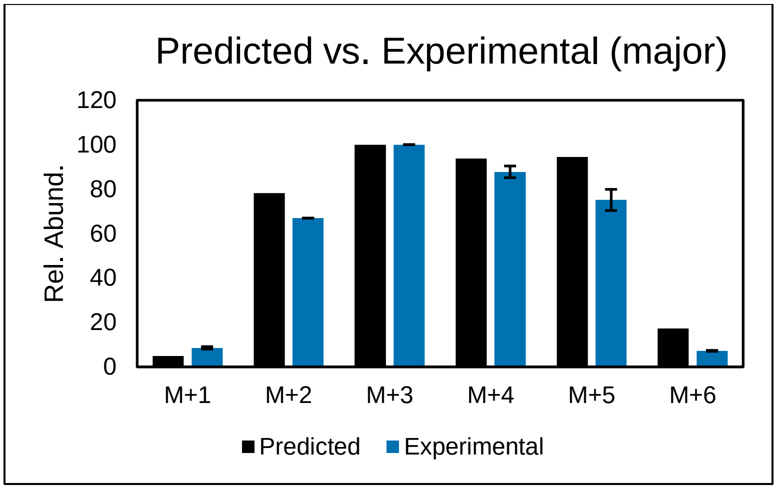

m/z	M+1	M+2	M+3	M+4	M+5	M+6
**Pred.**	4.83	78.17	100	93.72	94.46	17.28
**Trial 1**	9.11	66.92	100	90.41	79.90	7.42
**Trial 2**	7.99	66.92	100	85.15	70.37	7.00
**Avg**	**8.55**	**66.92**	**100**	**87.78**	**75.13**	**7.21**
**SD**	0.56	0	0	2.63	4.76	0.21

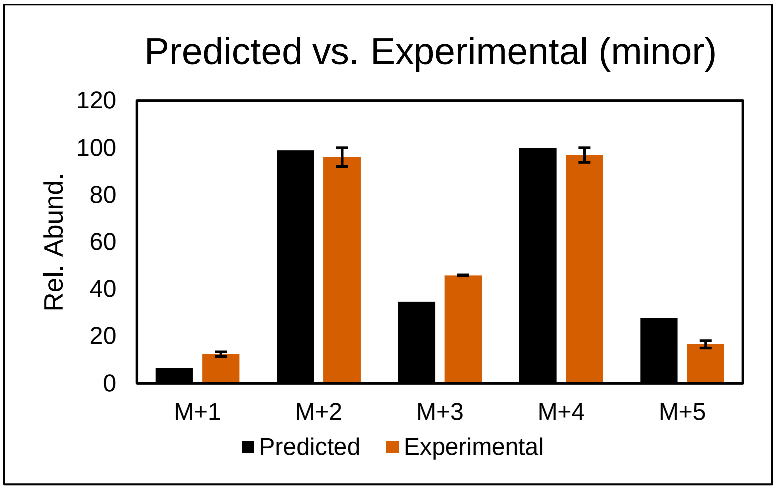

m/z	M+1	M+2	M+3	M+4	M+5
**Pred.**	6.52	98.89	34.6	100	27.74
**Trial 1**	13.32	100	45.56	93.80	18.06
**Trial 2**	11.38	92.05	46.02	100	14.95
**Avg**	**12.35**	**96.025**	**45.79**	**96.9**	**16.505**
**SD**	0.97	3.975	0.23	3.1	1.555

Fig. S141. Graphical representation of isotopic distribution for the major (top) and minor (bottom) bromination products of D-etodolac methyl ester. A table summarizing this data is provided below each graph.

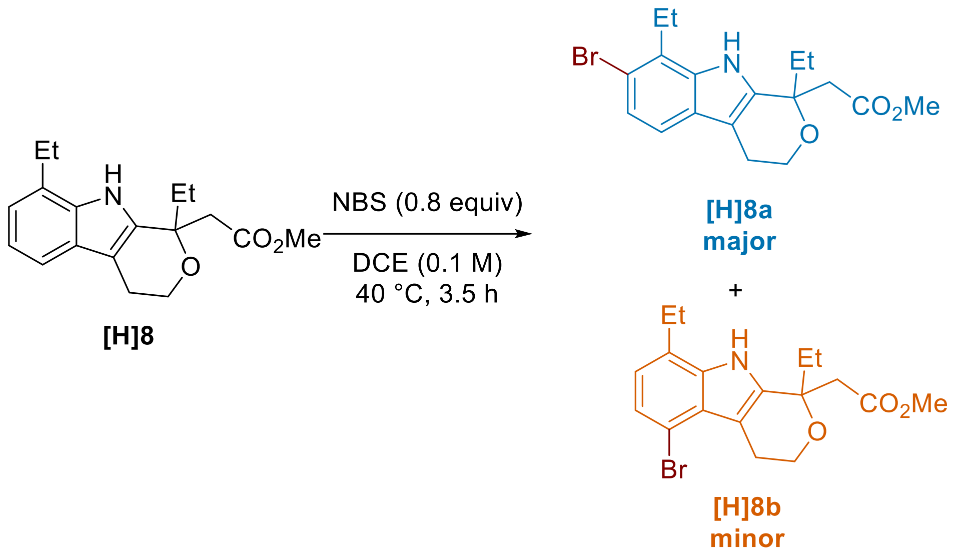

Bromination of H-etodolac methyl ester (control). This reaction was run under same conditions and on the same plate as D-etodolac methyl ester above. H-Etodolac methyl ester (28.3 mg, 0.0939 mmol) was dissolved in $ClCH_2CH_2Cl$ (1.0 mL) making a 93.8 mM stock solution. The stock solution (150 µL, 4.2 mg, 0.014 mmol) was added to a 1 mL glass vial (8 x 30 mm) equipped with a stir bar. The solvent was removed with a Genevac (vacuum centrifugation) 1 h cycle at 25 °C. A stock solution was prepared containing NBS (72.2 mg, 0.406 mmol) dissolved in $ClCH_2CH_2Cl$ (5.0 mL) resulting in an 81.2 mM solution. The NBS stock solution (140 µL, 2.0 mg, 0.011 mmol) was added to the glass vial and the glass vial was sealed in an aluminum reaction block and stirred using a tumble stirrer, the heat generated from the tumble stirrer resulted in a reaction temperature of 40 °C. After 3.5 h, the reaction was removed from stirring, concentrated with a Genevac (vacuum centrifugation) 1 h cycle at 25 °C. The reaction mixture was dissolved in MeCN (400 µL) and a portion of this solution of this solution (28.5 µL) was diluted in MeCN (5 mL) resulting in an approximately 200 µM solution (with respect to initial starting material). This solution was then analyzed using a Waters ACQUITY SQ. The reaction was performed in duplicate.

UPLC (Acquity SQ): Premier BEH C18; 2.4 µm; 50mm x 2.1mm; Column T = 40 °C; 0.75 mL/min. Gradient: 0.55 min at 50% MeCN/H_2O (0.1% FA); 3 min gradient to 55% MeCN/H_2O (0.1% FA); 1.85 min gradient to 90% MeCN/H_2O (0.1% FA); 0.05 min gradient to 5% MeCN/H_2O (0.1% FA); 0.1 min at 5% MeCN/H_2O (0.1% FA). Detection: PDA (TWC 210 – 400 nm); MS (ESI+, *m/z* = 375 – 390, 10 V, 20 Hz). Corrections factors for TWC were obtained from mixtures quantified by ^1H NMR spectroscopy. t_{major} = 3.46 min correction factor = 1.00 ; t_{minor} = 3.60 min, correction factor = 1.24.

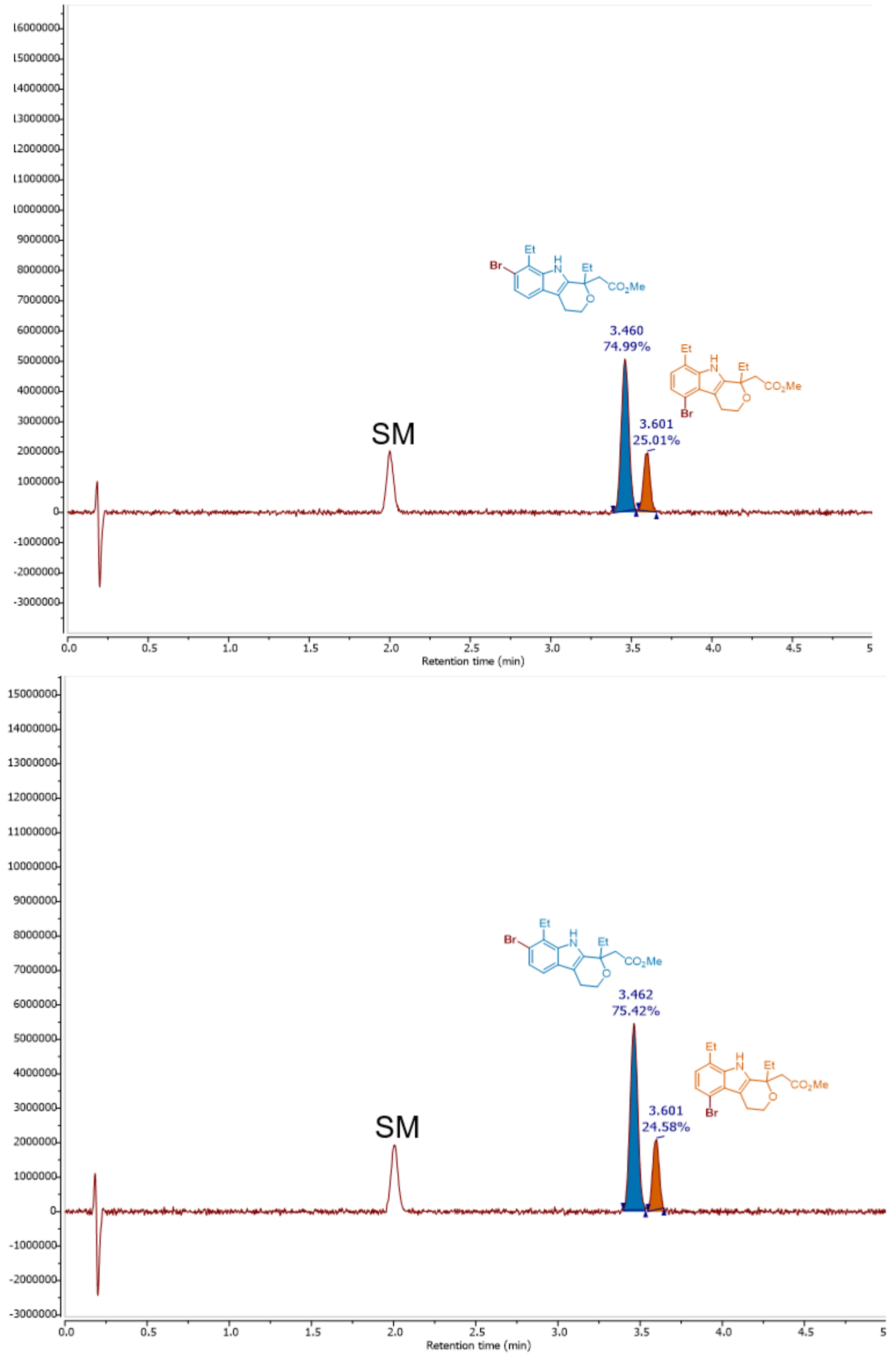

Fig. S142. HPLC trace for H-etodolac methyl ester bromination, trial 1 (top) and trial 2 (bottom).

Trial	Major[a]	Minor[a]	Major[b]	Minor[b]
**H-1**	75.0	25.0	75.0	29.8
**H-2**	75.4	24.6	75.4	29.2
**Avg**	75.2	24.8	**75.2**	**29.5**
**SD**	0.2	0.2	0.2	0.3

Trial	Major[a]	Minor[a]	Major[b]	Minor[b]
**D-1**	75.4	24.6	75.4	29.3
**D-2**	75.9	24.1	75.9	28.7
**Avg**	75.6	24.4	**75.6**	**29.0**
**SD**	0.3	0.3	0.3	0.3

Table S5. Summary of etodolac methyl ether major/minor Product ratios for protio (H-1 & H-2) and deutero (D-1 & D-2) trials. [a]Raw HPLC ratios. [b]Corrected ratios using response factors.

	Major	Minor
**[H]8**	71.8	28.1
**[D]8**	72.3	27.7

Table S6. Normalized product ratios for protio (**[H]8**) and deutero (**[D]8**) material.

7.1.2 MS-Only Analysis Etodolac Methyl Ester

One of the reaction mixtures above was analyzed via Loop injection to determine the ratio of **[D]8a** and **[D]8b** without LC separation. A 200 μM solution, with respect to initial starting material, was prepared in MeCN and was analyzed with a Waters AQUITY SQ using the following parameters: MS (ESI+, *m/z* = 375 – 390, 10 V, 20 Hz). Loop injections were performed in triplicate, and OLS deconvolution was performed to determine product ratios. A summary of the isotopic distribution and product ratios obtained are shown below.

m/z	M+1	M+2	M+3	M+4	M+5	M+6
**t1**	9.29	100	85.71	86.94	70.57	13.5
**t2**	9.44	100	87.68	88.36	72.82	12.93
**t3**	10.62	100	88.63	90.44	73.82	13.62
**Avg**	9.78	100	87.34	88.58	72.40	13.35
**SD**	0.59	0.00	1.22	1.44	1.36	0.30

	Major	Minor
**OLS**	73.0 (0.017)	27.0 (0.073)
**Calib. UV**	72.3	27.7

Table S7. (top) Summary of M+1–M+6 values for triplicate loop injections. (bottom) OLS deconvoluted product ratios vs calibrated UV of deutero trial, for the OLS values p-values are shown in parentheses.

7.2.1 Bromination of Estrone Methyl Ether

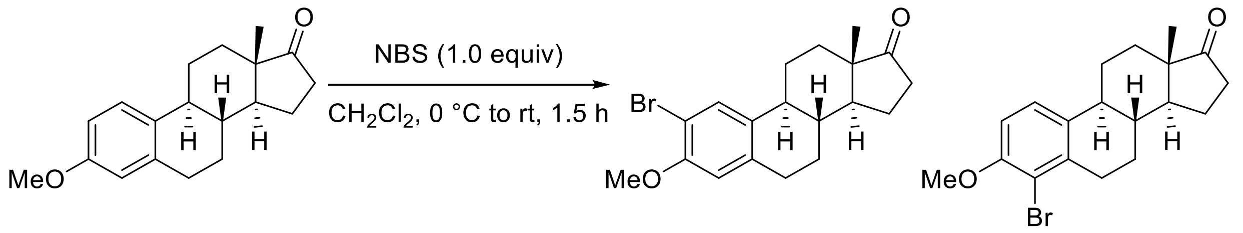

[H]9 **[H]9a** **[H]9b**

Bromination of H-estrone methyl ether. To a tapered 10 mL microwave vial was added H-estrone methyl ether (100 mg, 0.352 mmol) followed by CH_2Cl_2 (1.75 mL). The mixture was cooled to 0 °C using an ice bath and NBS (62.6 mg, 0.352 mmol) was added in one portion. After stirring for 30 min at 0 °C, the vial was removed from the ice bath and allowed to warm to rt. After 1 h, the reaction was deemed complete by TLC. After concentration and chromatography using 0-15% EtOAc in hexanes a mixture of products **[H]9a:[H]9b** (1:0.44) was obtained as an off-white solid (98.8 mg total, 0.272 mmol). Yields based off NMR ratios: 53% (**[H]9a**), 23% (**[H]9b**).

^{1}H NMR (600 MHz, Acetone) δ 7.41 (s, 1H, **[H]9a**), 7.31 (d, *J* = 8.6 Hz, 0.44H, **[H]9b**), 6.89 (d, *J* = 8.6 Hz, 0.44H, **[H]9b**), 6.79 (s, 1H, **[H]9a**), 3.85 (s, 1.39H, **[H]9b**), 3.83 (s, 3H, **[H]9a**), 2.98 (dd, *J* = 17.7, 5.8 Hz, 0.46H, **[H]9b**), 2.89 – 2.84 (m, 2.34H, **[H]9a/[H]9b**), 2.74 – 2.65 (m, 0.49H, **[H]9b**), 2.52 – 2.37 (m, 2H, **[H]9a**), 2.39 – 2.32 (m, 1H, **[H]9a**), 2.33 – 2.19 (m, 1.64H, **[H]9a/[H]9b**), 2.15 – 2.06 (m, 2.16H, **[H]9a/[H]9b**), 2.04 – 1.99 (m, 1.46H, **[H]9a/[H]9b**), 1.90 – 1.82 (m, 1.58H, **[H]9a/[H]9b**), 1.72 – 1.35 (m, 9.57H, **[H]9a/[H]9b**), 0.893 (s, 3H, **[H]9a**), 0.889 (s, 1.56H, **[H]9b**).

^{13}C{^{1}H} NMR (151 MHz, Acetone) δ 219.34 (**[H]9b**), 219.29 (**[H]9a**), 154.9 (**[H]9b**), 154.7 (**[H]9a**), 138.3 (**[H]9a**), 138.1 (**[H]9b**), 135.2 (**[H]9b**), 134.6 (**[H]9a**), 130.8, 126.0, 115.1, 113.5, 110.3, 109.0, 56.5 (**[H]9a**), 56.4 (**[H]9b**), 51.01 (**[H]9b**), 50.99 (**[H]9a**), 48.4 (**[H]9a**), 48.3 (**[H]9b**), 45.0 (**[H]9b**), 44.6 (**[H]9a**), 38.9 (**[H]9a**), 38.4 (**[H]9b**), 36.08 (**[H]9b**), 36.05 (**[H]9a**), 32.53 (**[H]9b**), 32.52 (**[H]9a**), 31.8 (2C), 27.4 (**[H]9b**), 27.1 (**[H]9a**), 26.9 (**[H]9b**), 26.6 (**[H]9a**), 22.10 (**[H]9a**), 22.09 (**[H]9b**), 14.10 (**[H]9a**), 14.06 (**[H]9b**).

HRMS (ESI, TOF) m/z: $[M]^+$ calculated for $C_{19}H_{23}BrO_2$ 362.0881, observed 362.0874.

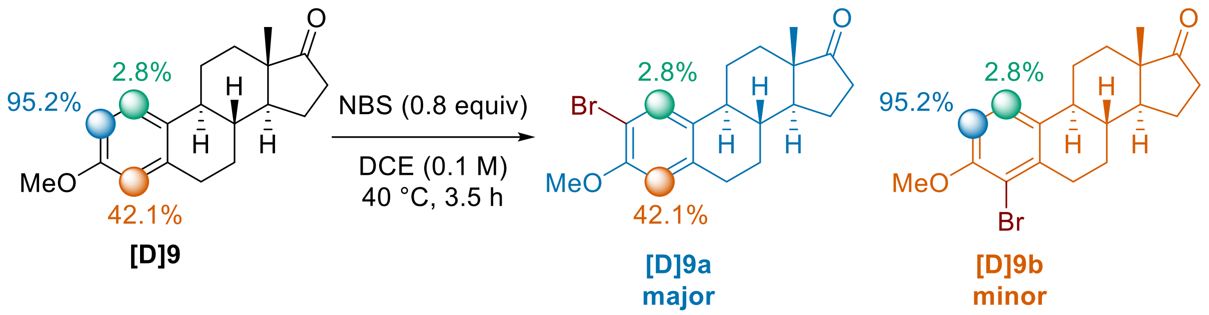

Bromination of D-estrone methyl ether. D-Estrone methyl ether (28.4 mg, 0.0998 mmol) was dissolved in $ClCH_2CH_2Cl$ (1.0 mL) making a 99.8 mM stock solution. The stock solution (141 µL, 4.0 mg, 0.014 mmol) was added to a 1 mL glass vial (8 x 30 mm) equipped with a stir bar. The solvent was removed with a Genevac (vacuum centrifugation) 1 h cycle at 25 °C. A stock solution was prepared containing NBS (72.2 mg, 0.406 mmol) dissolved in $ClCH_2CH_2Cl$ (5.0 mL) resulting in an 81.2 mM solution. The NBS stock solution (140 µL, 2.0 mg, 0.011 mmol) was added to the glass vial and the glass vial was sealed in an aluminum reaction block and stirred using a tumble stirrer, the heat generated from the tumble stirrer resulted in a reaction temperature of 40 °C. After 3.5 h the reaction was removed from stirring, concentrated with a Genevac (vacuum centrifugation) 1 h cycle at 25 °C. The reaction mixture was dissolved in MeCN (400 µL) and a portion of this solution (71 µL) was diluted in MeCN (5 mL) resulting in an approximately 500 µM solution (with respect to initial starting material). This solution was then analyzed using a Waters ACQUITY SQ. The reaction was performed in duplicate.

UPLC (Acquity SQ): Premier BEH C18; 2.4 µm; 50mm x 2.1mm; Column T = 40 °C; 0.75 mL/min. Gradient: 0.50 min at 50% $MeCN/H_2O$ (0.1% FA); 2.8 min gradient to 99% $MeCN/H_2O$ (0.1% FA); 0.55 min at 99% $MeCN/H_2O$ (0.1% FA); 0.05 min gradient to 5% $MeCN/H_2O$ (0.1% FA); 0.1 min at 5% $MeCN/H_2O$ (0.1% FA). Detection: PDA (TWC 210 – 400 nm); MS (ESI+, *m/z* = 355 – 370, 30 V, 20 Hz).

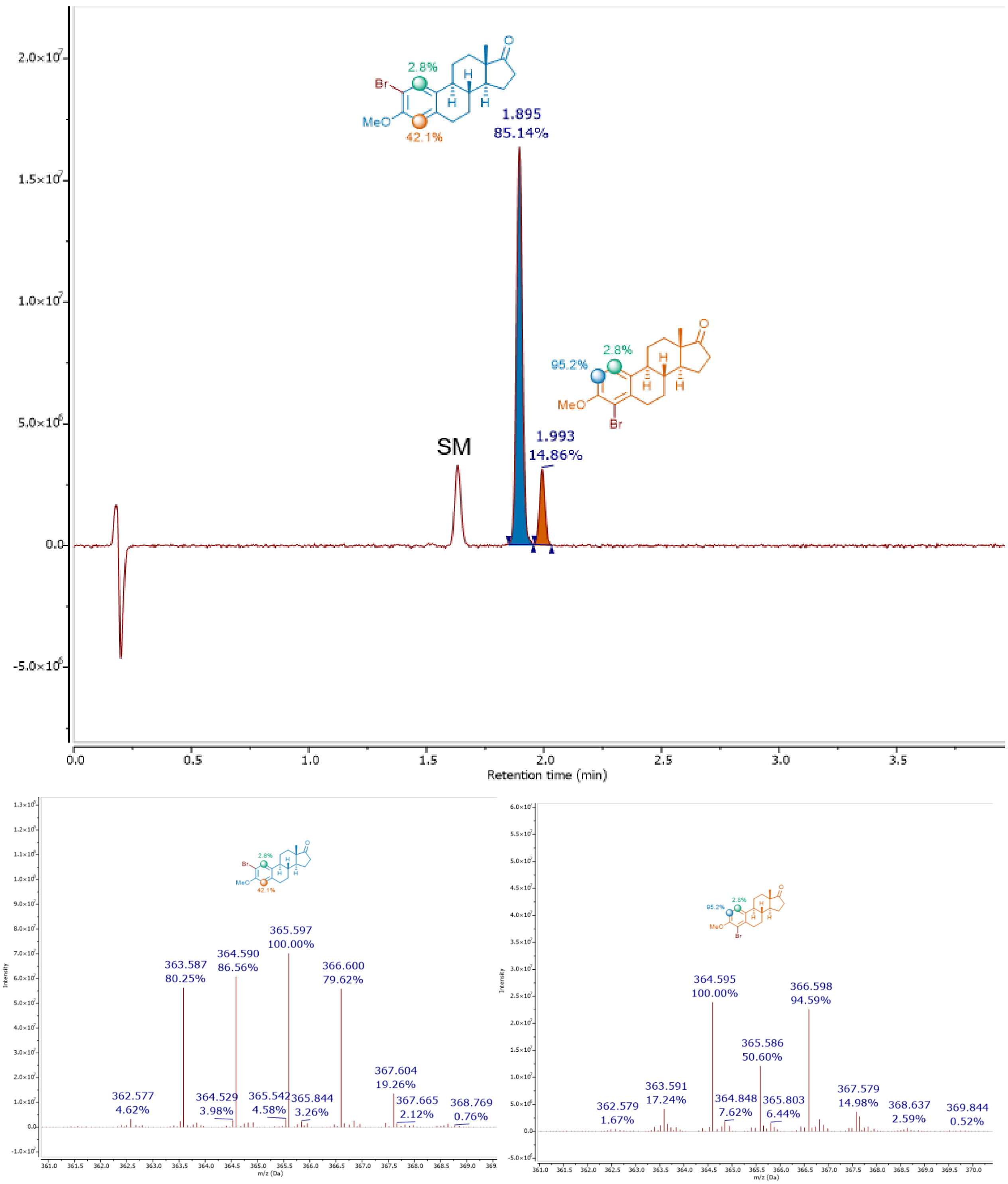

Fig. S143. D-Estrone methyl ether bromination HPLC trace (top) and isotopic patterns for the of major (bottom left) and minor products (bottom right) (Trial 1).

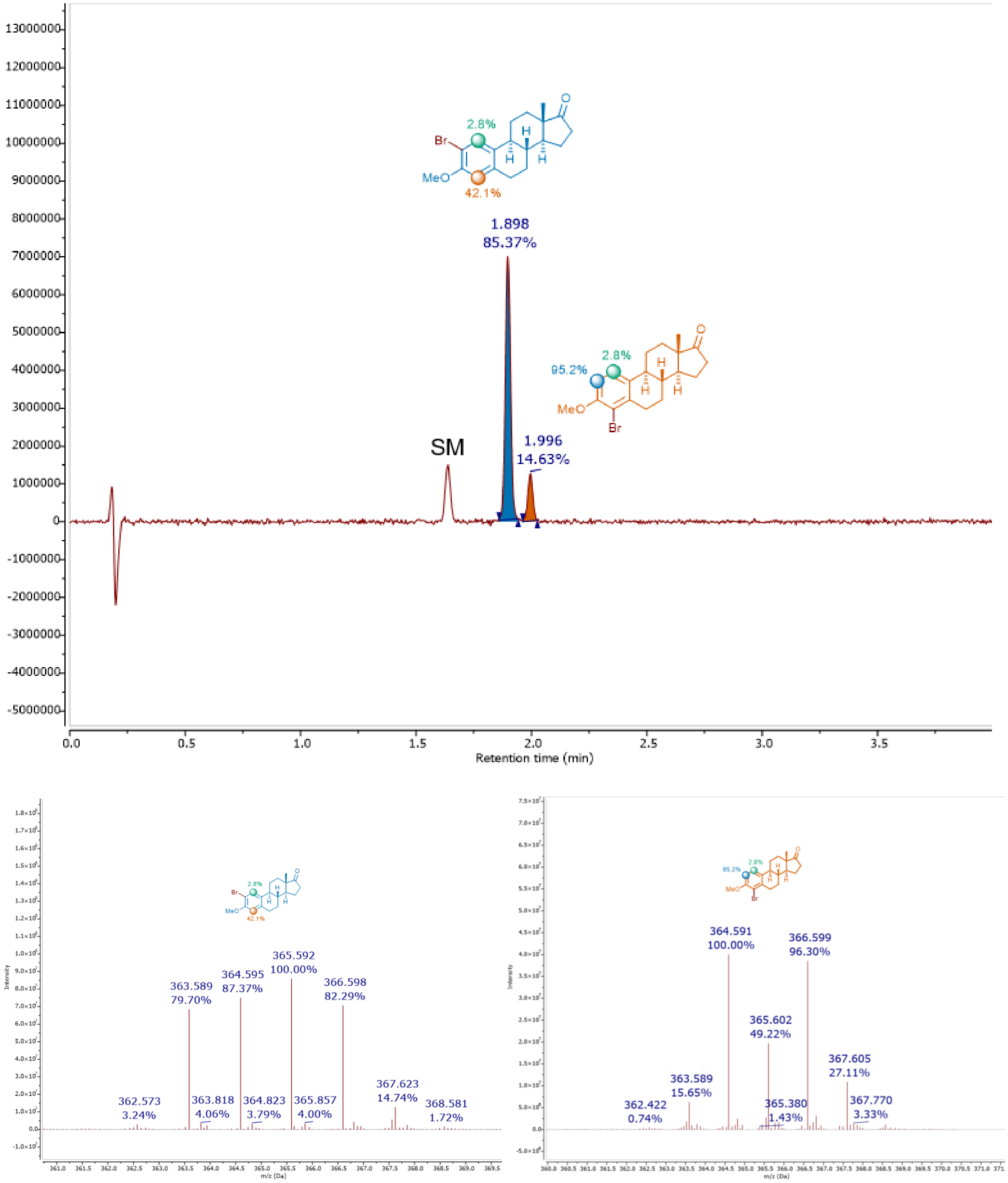

Fig. S144. D-Estrone methyl ether bromination HPLC trace (top) and isotopic patterns for the major (bottom left) and minor products (bottom right) (Trial 2).

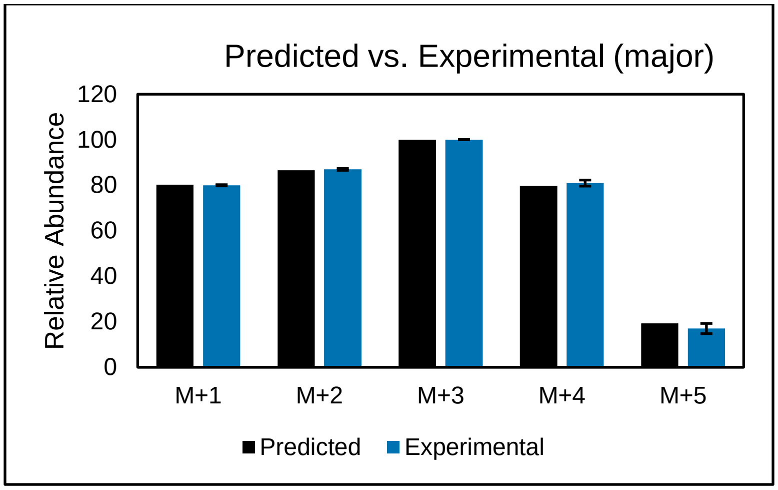

m/z	M+1	M+2	M+3	M+4	M+5
**Predicted**	85.11	82.03	100.00	81.76	16.74
**Trial 1**	80.25	86.56	100.00	79.62	19.26
**Trial 2**	79.7	87.37	100.00	82.29	14.74
**Avg**	79.97	86.96	100.00	80.95	17.00
**SD**	0.27	0.40	0	1.33	2.26

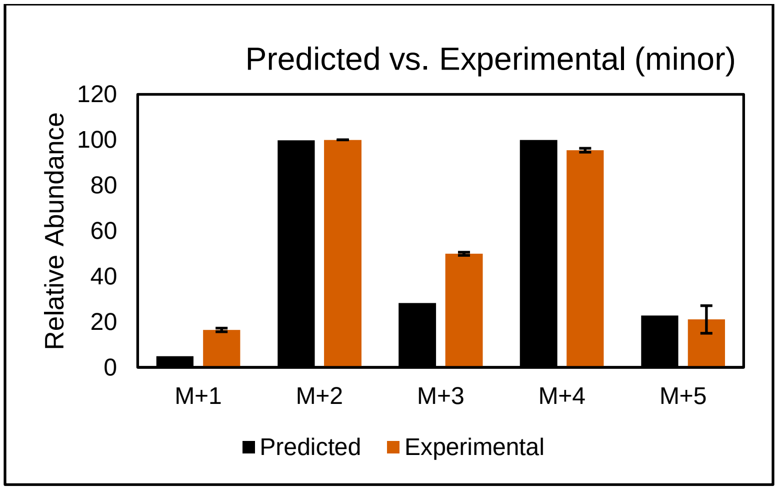

m/z	M+1	M+2	M+3	M+4	M+5
**Predicted**	4.97	99.75	28.27	100.00	22.82
**Trial 1**	17.24	100.00	50.6	94.59	14.98
**Trial 2**	15.65	100.00	49.22	96.3	27.11
**Avg**	16.445	100.00	49.91	95.445	21.045
**SD**	0.79	0	0.69	0.85	6.06

Fig. S145. Graphical representation of isotopic distribution for the major (top) and minor (bottom) bromination products of D-estrone methyl ether. A table summarizing this data is provided below each graph.

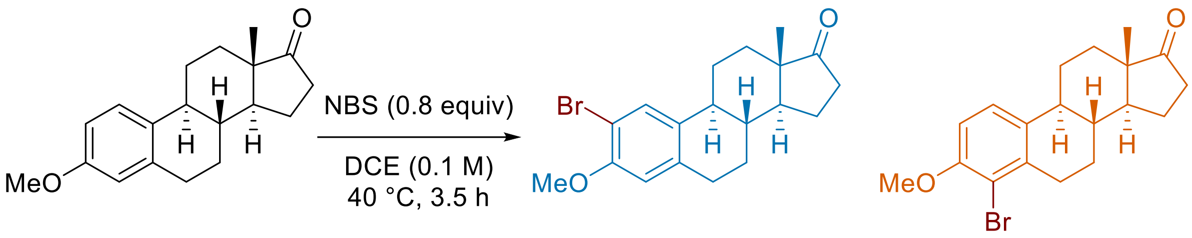

[H]9 **[H]9a major** **[H]9b minor**

Bromination of H-estrone methyl ether (control). This reaction was run under same conditions and on the same plate as D-etodolac above. H-Estrone methyl ether (25.0 mg, 0.0879 mmol) was dissolved in $ClCH_2CH_2Cl$ (1.0 mL) making an 87.9 mM stock solution. The stock solution (160 µL, 4.0 mg, 0.014 mmol) was added to a 1 mL glass vial (8 x 30 mm) equipped with a stir bar. The solvent was removed with a Genevac (vacuum centrifugation) 1 h cycle at 25 °C. A stock solution was prepared containing NBS (72.2 mg, 0.406 mmol) dissolved in $ClCH_2CH_2Cl$ (5.0 mL) resulting in an 81.2 mM solution. The NBS stock solution (140 µL, 2.0 mg, 0.011 mmol) was added to the glass vial and the glass vial was sealed in an aluminum reaction block and stirred using a tumble stirrer, the heat generated from the tumble stirrer resulted in a reaction temperature of 40 °C. After 3.5 h, the reaction was removed from stirring and concentrated with a Genevac (vacuum centrifugation) 1 h cycle at 25 °C. The reaction mixture was dissolved in MeCN (400 µL) and a portion of this solution (71 µL) was diluted in MeCN (5 mL) resulting in an approximately 500 µM solution (with respect to initial starting material). This solution was then analyzed using a Waters ACQUITY SQ. The reaction was performed in duplicate.

UPLC (Acquity SQ): Premier BEH C18; 2.4 µm; 50mm x 2.1mm; Column T = 40 °C; 0.75 mL/min. Gradient: 0.50 min at 50% MeCN/H_2O (0.1% FA); 2.8 min gradient to 99% MeCN/H_2O (0.1% FA); 0.55 min at 99% MeCN/H_2O (0.1% FA); 0.05 min gradient to 5% MeCN/H_2O (0.1% FA); 0.1 min at 5% MeCN/H_2O (0.1% FA). Detection: PDA (TWC 210 – 400 nm); MS (ESI+, *m/z* = 355 – 370, 30 V, 20 Hz). Corrections factors for TWC were obtained from mixtures quantified by ^1H NMR spectroscopy. t_{major} = 1.898 min correction factor = 1.00 ; t_{minor} = 1.997 min, correction factor = 1.16.

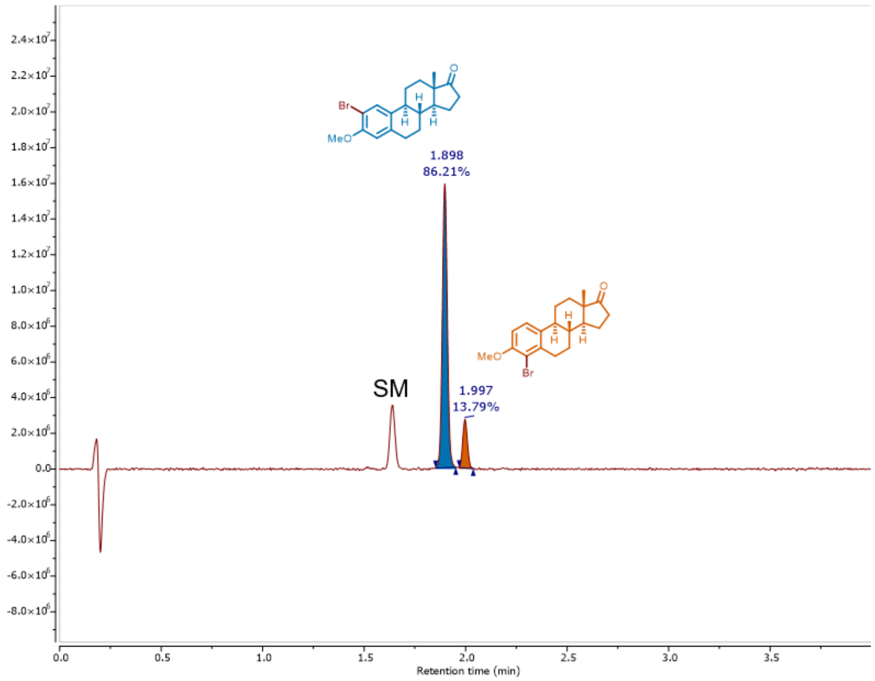

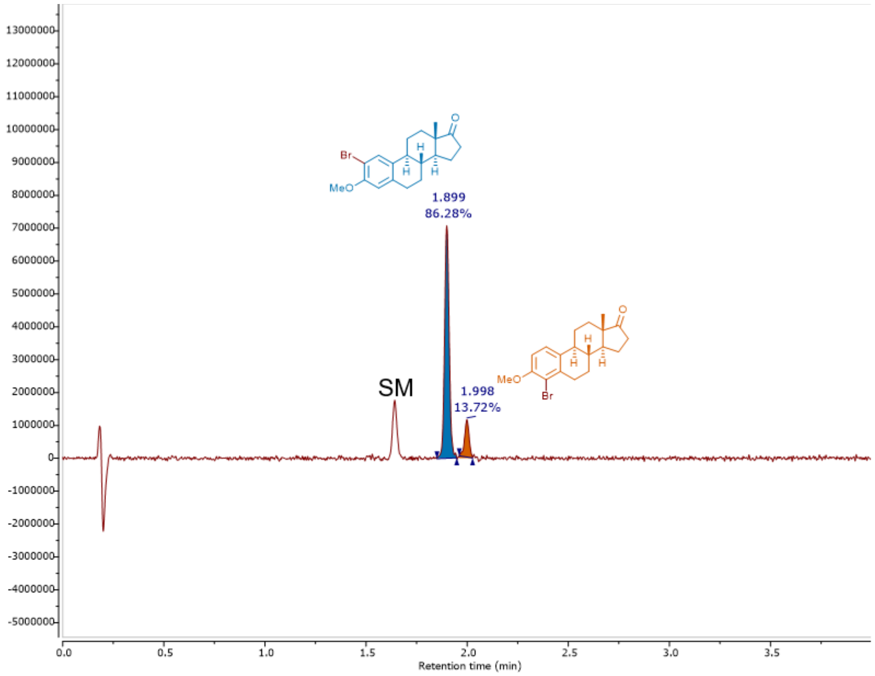

Fig. S146. H-Estrone methyl ether bromination HPLC Trace for both trial 1 (top) and trial 2 (bottom).

Trial	Major[a]	Minor[a]	Major[b]	Minor[b]
**H-1**	86.2	13.8	86.2	16.5
**H-2**	86.3	13.7	86.3	16.5
**Avg**	86.2	13.7	**86.2**	**16.5**
**SD**	0.03	0.03	0.03	0.04

Trial	Major[a]	Minor[a]	Major[b]	Minor[b]
**D-1**	85.1	14.9	85.1	17.8
**D-2**	85.4	14.6	85.4	17.6
**Avg**	85.2	14.7	**85.2**	**17.7**
**SD**	0.1	0.1	0.1	0.1

Table S8. Summary of estrone methyl ether major/minor product ratios for protio (H-1 & H-2) and deutero (D-1 & D-2) trials. [a]Raw HPLC ratios. [b]Corrected ratios using response factors.

	Major	Minor
**[H]9**	83.9	16.1
**[D]9**	82.8	17.2

Table S9. Normalized product ratios for protio (**[H]9**) and deutero (**[D]9**) material.

7.2.2 MS-Only Analysis Estrone Methyl Ether

One of the reaction mixtures above was analyzed via Loop injection to determine the ratio of major and minor without LC separation. A 4 mM solution, with respect to initial starting material, was prepared in MeCN and was analyzed with a Waters AQUITY SQ using the following parameters: MS (ESI+, *m/z* = 355 – 370, 30 V, 20 Hz). Loop injections were performed in triplicate, and OLS deconvolution was performed to determine product ratios. A summary of the isotopic distribution and product ratios obtained are shown below.

m/z	M+1	M+2	M+3	M+4	M+5	M+6
**t1**	71.47	95.8	100	96.31	29.31	4.76
**t2**	72.37	96.43	100	97.62	29.19	5
**t3**	71.51	95.51	100	96.80	29.34	4.95
**Avg**	71.78	95.91	100	96.91	29.28	4.90
**SD**	0.42	0.38	0.00	0.54	0.06	0.10

	Major	Minor
**OLS**	82.4 (0.013)	17.6 (0.066)
**Calib. UV**	82.8	17.2

Table S10. Summary of M+1–M+6 values for triplicate loop injections (top). OLS deconvoluted product ratios vs calibrated UV of deutero trial, for the OLS values p-values are shown in parentheses (bottom).

7.3 Cyanation of Nateglinide Methyl Ester

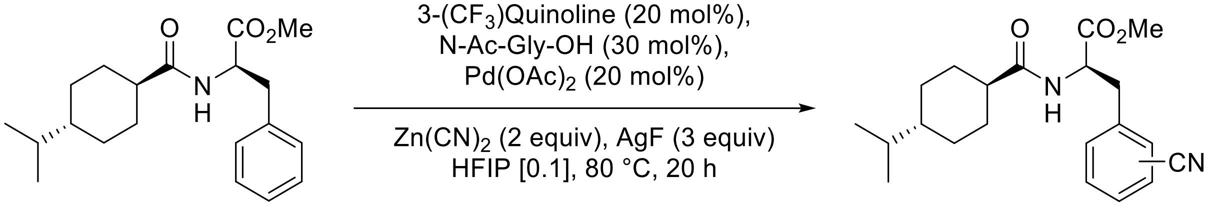

23% yield
o:m:p = (1:0.91:0.70)
ratios from isolated material

H-Nateglinide-methyl ester cyanation. To a 10 mL microwave vial was added **H-nateglinide-methyl ester** (49.7 mg, 0.150 mmol), AgF (57.1 mg, 0.450 mmol), and 3-trifluoromethylquinoline (5.9 mg, 0.030 mmol), N-Ac-Gly-OH (5.3 mg, 0.045 mmol), $Zn(CN)_2$ (35.2 mg, 0.300 mmol), and $Pd(OAc)_2$ (6.7 mg, 0.030 mmol). HFIP (1.5 mL) was added and the vial was sealed with a crimp cap. After heating to 80 °C in an aluminum heating block for 20 h, the reaction was allowed to cool to rt before filtering the mixture through a silica plug and rinsing with EtOAc (15 mL). The filtrate was concentrated and chromatographed using 0-30% EtOAc in hexanes. The *ortho*-substituted product was isolated as an off-white solid (4.7 mg, 0.013 mmol, 9% yield). Fractions containing the *meta/para*-products were separated in two. The first set of fractions contained 3.4 mg of material with a ratio of *meta:para* = 1:0.285; the second half of fractions contained 4.3 mg of material with a ratio of *meta:para* = 1:1.5. The yield for the *meta*-substituted product using the NMR ratios was determined to be (4.3 mg, 0.012 mmol, 8% yield) while the yield for the *para*-substituted product was (3.3 mg, 0.009 mmol, 6% yield).

***ortho*-Product:**
^{1}H NMR (600 MHz, $CDCl_3$) δ 7.62 (dd, *J* = 7.7, 1.4 Hz, 1H), 7.54 (td, *J* = 7.7, 1.4 Hz, 1H), 7.39 (d, *J* = 7.8 Hz, 1H), 7.35 (td, *J* = 7.6, 1.2 Hz, 1H), 6.03 (d, *J* = 8.1 Hz, 1H), 4.94 (td, *J* = 7.8, 5.6 Hz, 1H), 3.79 (s, 3H), 3.41 (dd, *J* = 14.1, 5.7 Hz, 1H), 3.23 (dd, *J* = 14.1, 7.6 Hz, 1H), 2.02 (ddd, *J* = 15.8, 12.3, 3.6 Hz, 1H), 1.93 – 1.81 (m, 2H), 1.81 – 1.73 (m, 3H), 1.45 – 1.30 (m, 3H), 1.09 – 0.91 (m, 2H), 0.85 (d, *J* = 6.8 Hz, 6H).

$^{13}C\{^1H\}$ NMR (151 MHz, $CDCl_3$) δ 175.9, 171.8, 140.6, 133.0, 132.9, 130.8, 127.8, 118.2, 113.5, 53.0, 52.6, 45.6, 43.3, 37.0, 32.9, 29.7, 29.6, 29.1, 29.0, 19.9.

HRMS (ESI, TOF) m/z: $[M+Na]^+$ calculated for $C_{21}H_{28}N_2O_3Na$ 379.1992, observed 379.2016.

***meta*- and *para*-Products:**
^{1}H NMR (600 MHz, $CDCl_3$) δ 7.58 (d, *J* = 8.0 Hz, 0.58H, ***para***), 7.55 (d, *J* = 8.1 Hz, 1H, ***meta***), 7.40 (t, *J* = 7.7 Hz, 1H, ***meta***), 7.37 (s, 1H, ***meta***), 7.35 (d, *J* = 7.8 Hz, 1H, ***meta***), 7.21 (d, *J* = 7.9 Hz, 0.63H, ***para***), 5.98 – 5.85 (m, 1.31H, ***mix***), 4.93 – 4.85 (m, 1.35H, ***mix***), 3.76 (s, 3H, ***meta***), 3.75 (s, 0.94H, ***para***), 3.29 – 3.22 (m, 1.38H, ***mix***), 3.16 – 3.07 (m, 1.38H, ***mix***), 2.06 – 1.98 (m, 1.15H, ***mix***), 1.92 – 1.83 (m, 2.56H, ***mix***), 1.82 – 1.76 (m, 2.98H, ***mix***), 1.46 – 1.34 (m, 4.24H, ***mix***), 1.09 – 0.93 (m, 4.46H, ***mix***), 0.86 (d, *J* = 6.9 Hz, 8.95H, ***mix***).

$^{13}C\{^1H\}$ NMR (151 MHz, $CDCl_3$) δ 175.8 (***mix***), 171.9 (***mix***), 141.9 (***para***), 137.9 (***meta***), 134.0 (***meta***), 133.0 (***meta***), 132.4 (***para***), 131.0 (***meta***), 130.3 (***para***), 129.4 (***meta***), 118.8 (***para***), 118.7 (***meta***), 112.7 (***meta***), 111.3 (***para***), 52.8 (***meta***), 52.7 (***mix***), 52.6 (***para***), 45.7 (***meta***), 45.6 (***para***), 43.34 (***para***), 43.33 (***meta***), 38.2 (***para***), 37.7 (***meta***), 32.9 (***mix***), 30.04 (***meta***), 30.00 (***para***), 29.66 (***meta***), 29.65 (***para***), 29.09 (***meta***), 29.08 (***para***), 29.00 (***mix***), 19.9 (***mix***).

The spectral data for the *meta/para*-products agree with those reported.[25]

Calibration curves were done using the following UPLC conditions:

UPLC (Acquity SQ): ACQUITY UPLC HSS C18; 1.8 μm; 150 mm x 2.1 mm; Column T = 40 °C; 0.350 mL/min. Gradient: 5.00 min at 54% MeCN/H_2O (0.1% FA); 2 min gradient to 58% MeCN/H_2O (0.1% FA); 1 min gradient to 95% MeCN/H_2O (0.1% FA); 0.85 min at 95% MeCN/H_2O (0.1% FA); 0.05 min to 5% MeCN/H_2O (0.1% FA); 0.1 min at 5% MeCN/H_2O (0.1% FA). Detection: PDA (TWC 190 – 260 nm); MS (ESI+, *m/z* = 320 – 380, 30 V, 20 Hz).

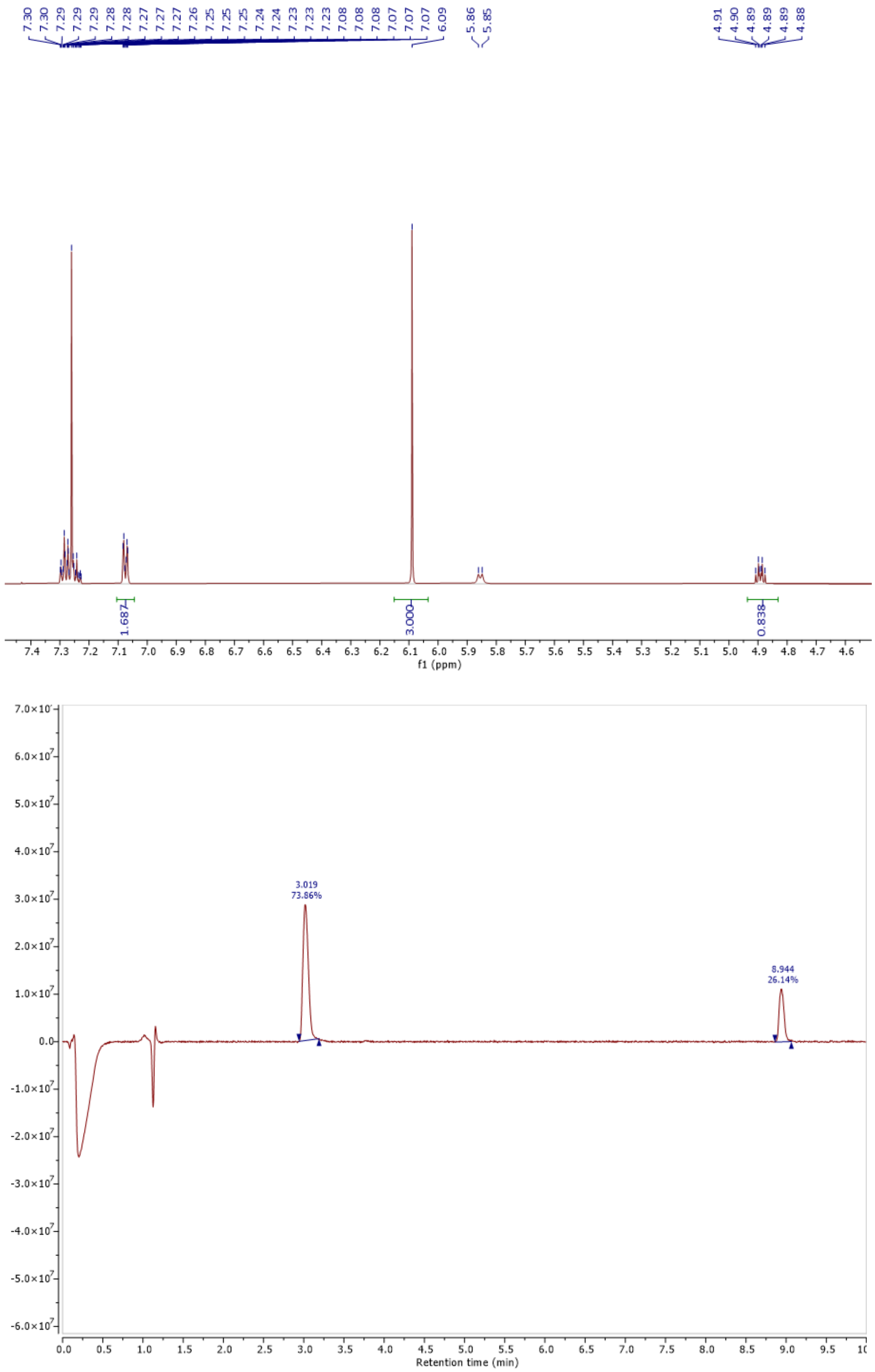

Fig. S147. Trial 1 to determine the correction factor of a mixture of H-nateglinide methyl ester (**[H]10**) and 1,3,5-trimethoxybenzene. ^{1}H NMR of a mixture of the two compounds (top). UPLC trace of the same mixture (bottom).

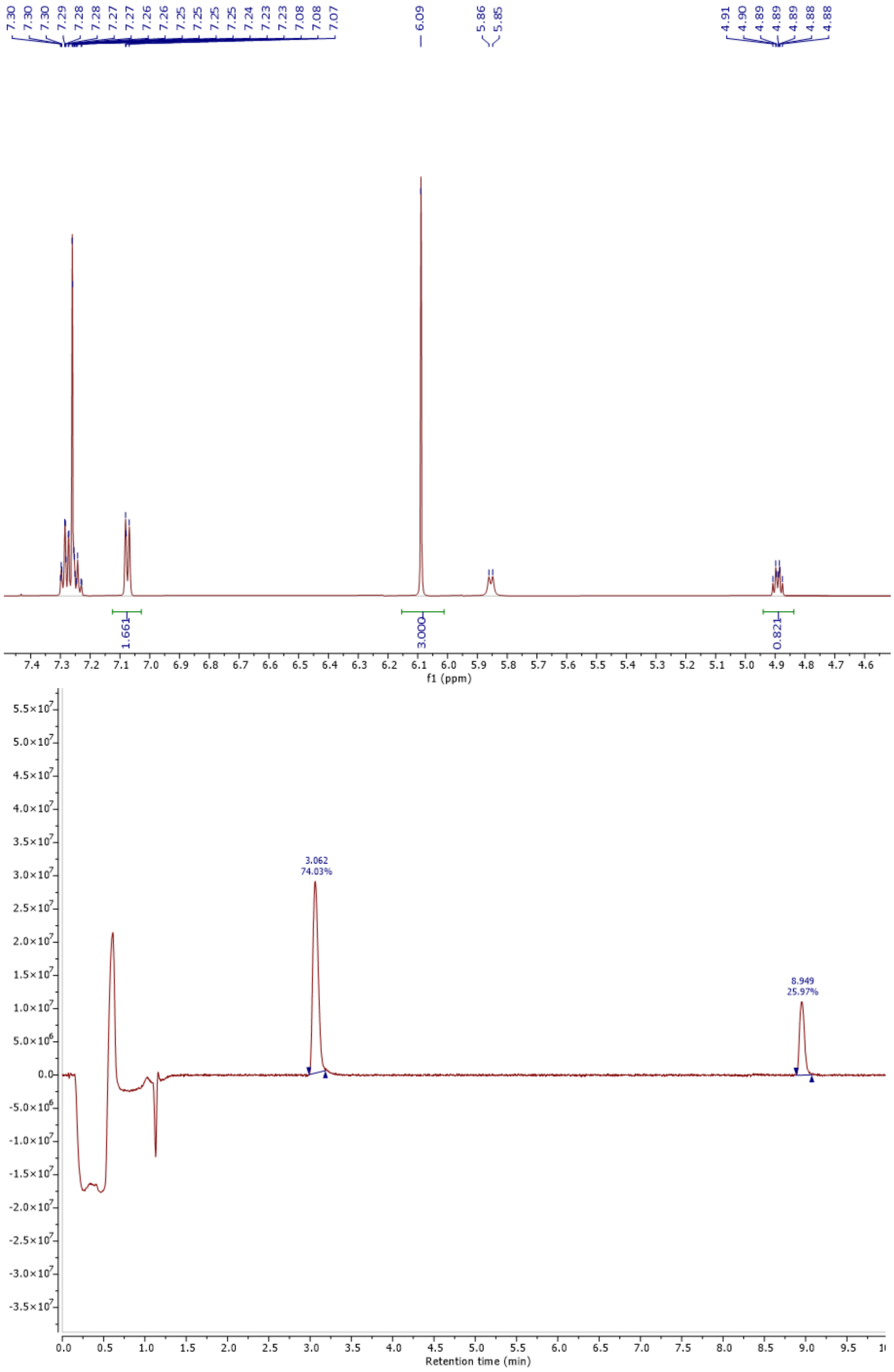

Fig. S148. Trial 2 to determine the correction factor of a mixture of H-nateglinide methyl ester (**[H]10**) and 1,3,5-trimethoxybenzene. ^{1}H NMR of a mixture of the two compounds (top). UPLC trace of the same mixture (bottom).

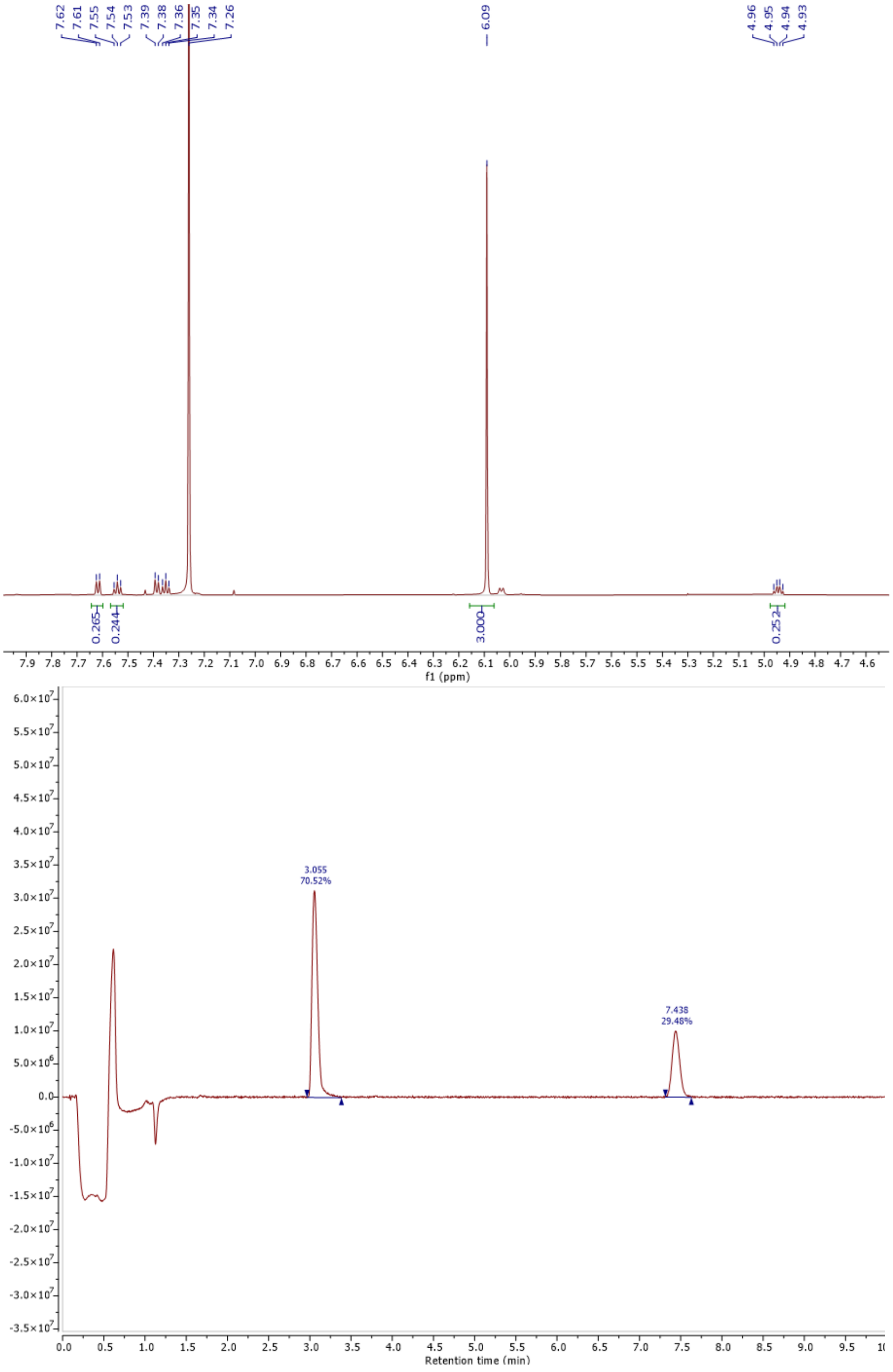

Fig. S149. Trial 1 to determine the correction factor of a mixture of *ortho*-cyano H-nateglinide methyl ester (**[H]10a**) and 1,3,5-trimethoxybenzene. ^{1}H NMR of a mixture of the two compounds (top). UPLC trace of the same mixture (bottom).

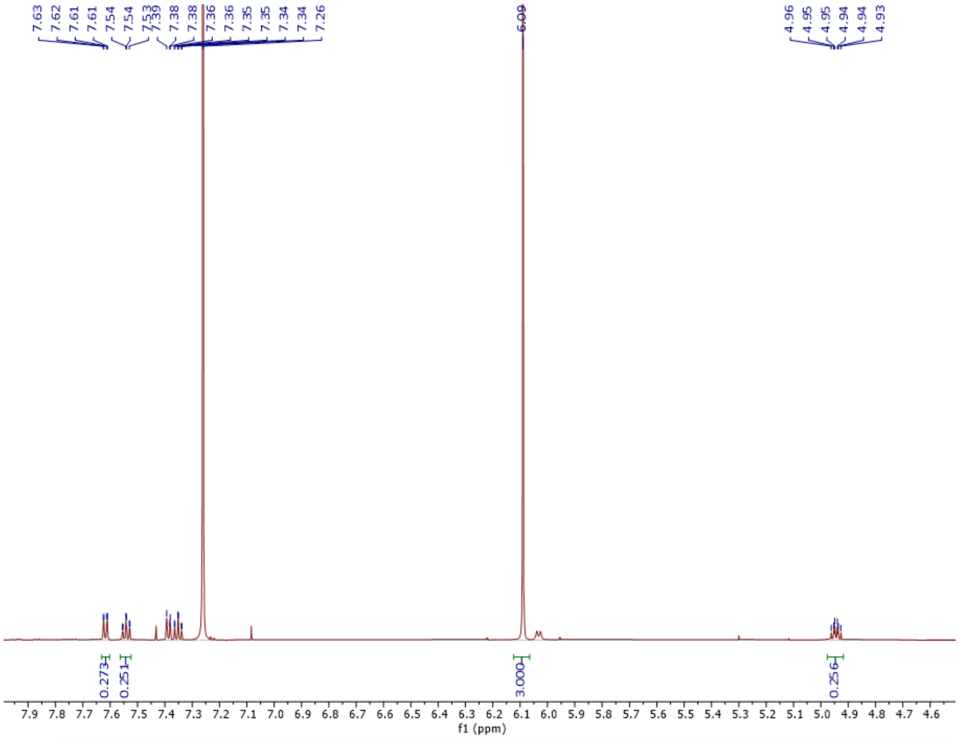

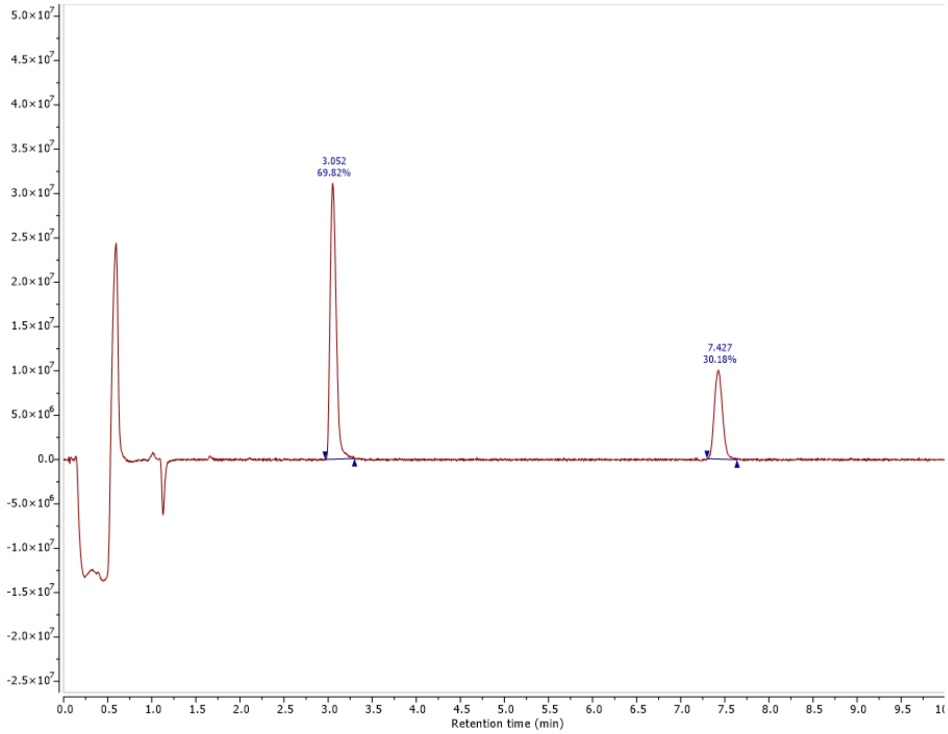

Fig. S150. Trial 2 to determine the correction factor of a mixture of *ortho*-cyano H-nateglinide methyl ester (**[H]10a**) and 1,3,5-trimethoxybenzene. ^{1}H NMR of a mixture of the two compounds (top). UPLC trace of the same mixture (bottom).

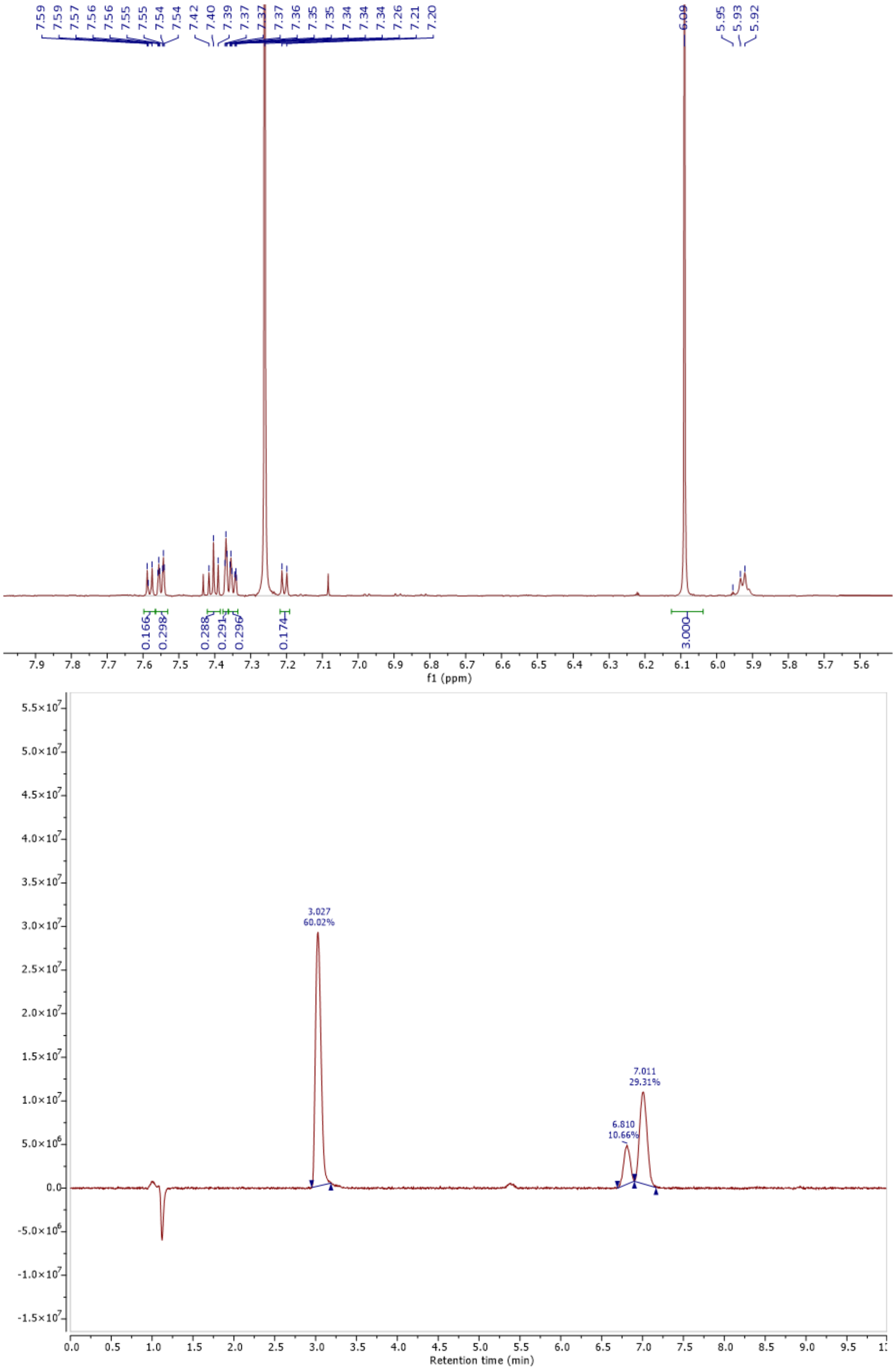

Fig. S151. Trial 1 to determine the correction factor of a mixture of *meta/para*-cyano H-nateglinide methyl ester (**[H]10b/**(**[H]10c**) and 1,3,5-trimethoxybenzene. ^{1}H NMR of a mixture of the two compounds (top). UPLC trace of the same mixture, the para isomer elutes at 6.810 min and the meta isomer at 7.011 min (bottom).

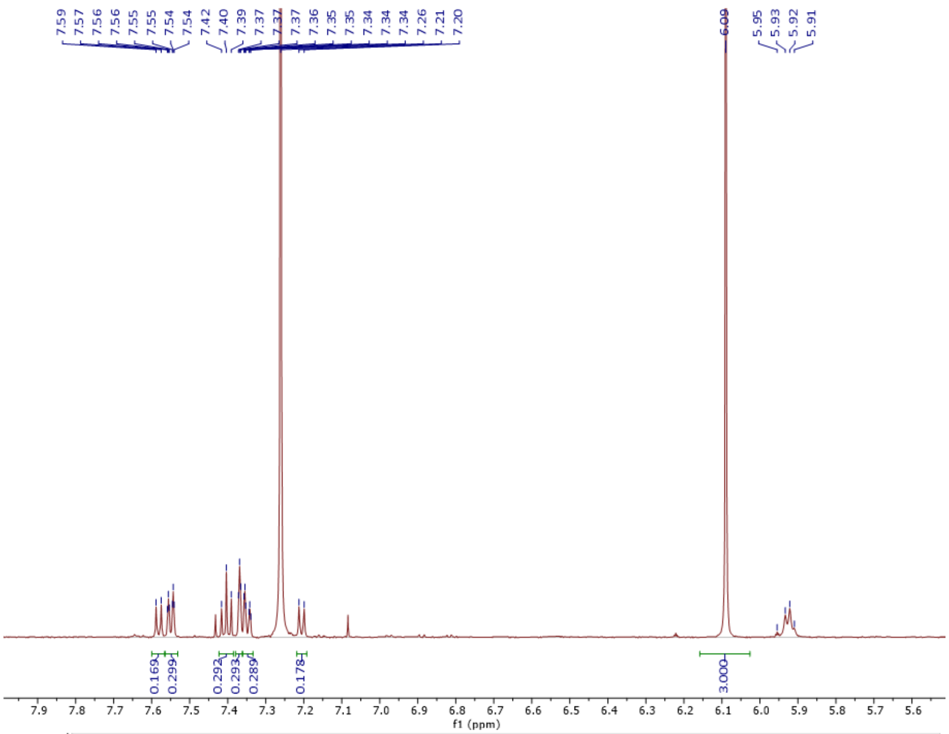

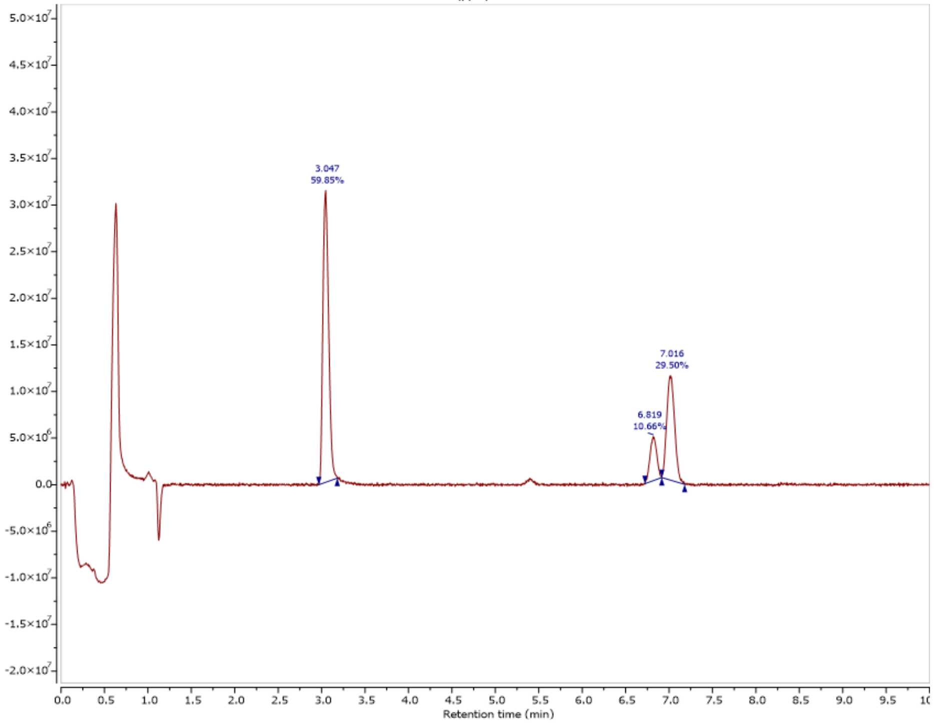

Fig. S152. Trial 2 to determine the correction factor of a mixture of *meta/para*-cyano H-nateglinide methyl ester (**[H]10b/**(**[H]10c**) and 1,3,5-trimethoxybenzene. ^{1}H NMR of a mixture of the two compounds (top). UPLC trace of the same mixture, the para isomer elutes at 6.810 min and the meta isomer at 7.011 min (bottom).

Substrate	Correction Factor[a]
[H]-Nateglinide methyl ester	2.365 ± 0.011
Ortho Product (**[H]10a**)	0.604 ± 0.003
Meta Product (**[H]10b**)	0.599 ± 0.0001
Para Product (**[H]10c**)	0.483 ± 0.004

[a]Average of duplicates shown above.

Table S11. Summary of the correction factors determined for H-Nateglinide methyl ester (**[H]10**) as well as the *ortho* (**[H]10a**), *meta* (**[H]10b**), and *para* (**[H]10c**) cyanation products.

8. Deuteration of sp^3 Centers

8.1 Deuteration of 1-(4-Methoxybenzyl)indoline

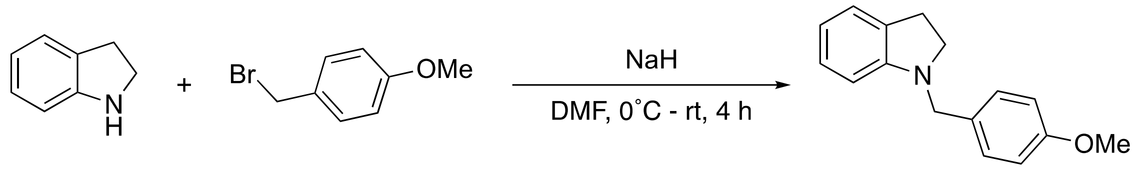

[**Caution:** Sodium hydride is extremely reactive with water. It produces hydrogen gas in an exothermic reaction which can be sufficient to cause a fire or explosion. Excess sodium hydride should be carefully quenched with alcoholic solvents at lower than ambient temperatures.]

1-(4-Methoxybenzyl)indoline ([H]11). To a dry 100 mL rbf was added NaH (293 mg, 90 wt%, 11.0 mmol) in a glovebox. The flask was cooled to 0 °C, before adding DMF (30.3 mL) and indoline (1.19 g, 10.0 mmol) under Ar. After stirring for 1 h, 1-(bromomethyl)-4-methoxybenzene (2.01 g, 10.0 mmol) was added and the flask was warmed to rt. After 4 h, the reaction mixture was cooled to 0 °C and quenched with water (60 mL). The aqueous layer was washed with EtOAc (3 x 30 mL), and the combined organics were washed with satd NaCl (60 mL), dried using anhydrous Na_2SO_4, filtered, and concentrated. The resultant material was purified via column chromatography using 0-5% EtOAc in hexanes to yield pure **1-(4-methoxybenzyl)indoline** as a white amorphous solid (2.00 g, 8.35 mmol, 84%).

^{1}H NMR (600 MHz, Acetone) δ 7.30 (d, *J* = 8.6, 2H), 7.03 (d, *J* =7.2 Hz, 1H), 6.98 (t, *J* = 7.7 Hz 1H), 6.90 (d, *J* =8.6 Hz, 2H), 6.58 (t, *J* = 7.4, Hz, 1H), 6.55 (d, *J* =7.8 Hz, 1H), 4.19 (s, 2H), 3.78 (s, 3H), 3.24 (t, *J* = 8.3 Hz, 2H), 2.89 (t, *J* = 8.3 Hz, 2H).

^{13}C{^{1}H} NMR (151 MHz, Acetone) δ 160.0, 153.7, 131.5, 131.0, 130.3, 128.1, 125.3, 118.5, 114.7, 108.1, 55.6, 54.1, 53.6, 29.2.

Spectral data agree with those reported.[26]

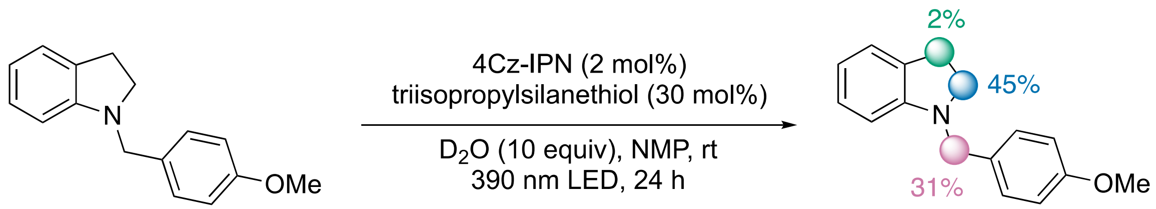

D-1-(4-Methoxybenzyl)indoline ([D]11). To a tapered microwave vial was added **1-(4-methoxybenzyl)indoline** (100 mg, 0.418 mmol), 4CzIPN (6.59 mg, 8.36 µmol), dry NMP (1.67 mL), D_2O (83.7 mg, 75.6 µL, 4.18 mmol), and triisopropylsilanethiol (23.9 mg, 0.30 Eq, 125 µmol). The vial was sealed with a crimp cap, then degassed via 3 cycles of vacuum evacuation (2 min with stirring)/Ar backfill. The vial was sealed with parafilm and irradiated with a 390 nm Kessil lamp for 24 h. The reaction mixture was diluted with EtOAc (5 mL) and washed with satd NaCl (3 x 7 mL). The organic layer was dried with anhydrous Na_2SO_4, filtered and concentrated. The resultant material was purified via column chromatography using 30% toluene in hexanes to yield pure **D-1-(4-methoxybenzyl)indoline** as an off white solid (40.8 mg, 0.170 mmol, 41%).

^{1}H NMR (600 MHz, Acetone) δ 7.29 (d, *J* = 8.5 Hz, 2H), 7.03 (d, *J* = 7.2 Hz, 1H), 6.98 (t, *J* = 7.7 Hz, 1H), 6.90 (d, *J* = 8.6 Hz, 2H), 6.59 (t, *J* = 7.3 Hz, 1H), 6.57 (d, *J* = 7.7 Hz, 1H), 4.21 – 4.14 (m, 1.36H), 3.78 (s, 3H), 3.28 – 3.18 (m, 1.09H), 2.91 – 2.84 (d, *J* = 8.4 Hz, 1.96H).

^{13}C{^{1}H} NMR (151 MHz, Acetone) δ 159.9, 153.61 – 153.57 (m, *adjacent*), 131.4 – 131.2 (m, *adjacent*), 130.85 – 130.84 (m, *adjacent*), 130.14 – 130.11 (m, *adjacent*), 127.9, 125.1, 118.3 – 118.2 (m, *adjacent*), 114.6, 108.00 – 107.96 (m, *adjacent*), 55.5, 54.0 – 53.5 (m, *labeled*), 53.4 – 53.37 (m, *labeled*), 53.05 (t, 20.8 Hz, 0 D adjacent), 53.03 (t, 20.8 Hz, 1 D adjacent), 53.01 (t, 20.7 Hz, 2 D adjacent), 29.0 – 28.8 (m, *labeled*).

MS (ESI, SQ): loop injection, concentration 1 mM, injection vol 0.1 µL, cone voltage 10 V.

Isopat: 1.627 D (NMR: 1.560)

%D Calculation: Method 2.1 (the deuterium incorporation was calculated using the following equation $\%D = 100 * (integration\ of\ protio\ NMR - integration\ of\ deuterated\ NMR)$. The NMR for the protio material was taken in triplicate and the integrations were averaged to get the integration of protio NMR value.)

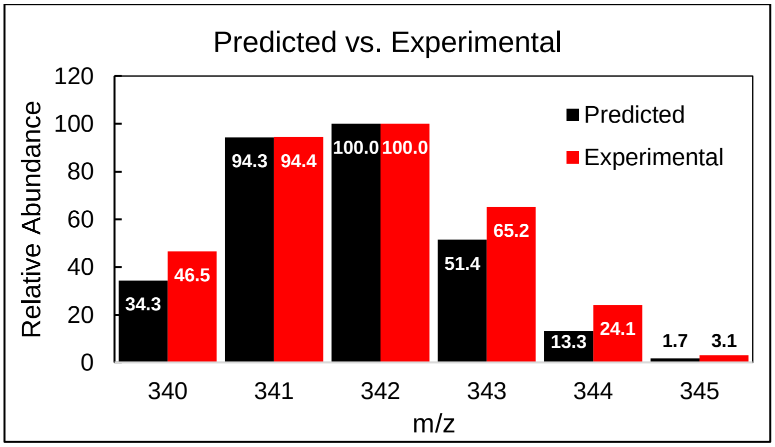

9. Reaction at sp^3 Centers

9.1 Reaction of 1-(4-Methoxybenzyl)indoline

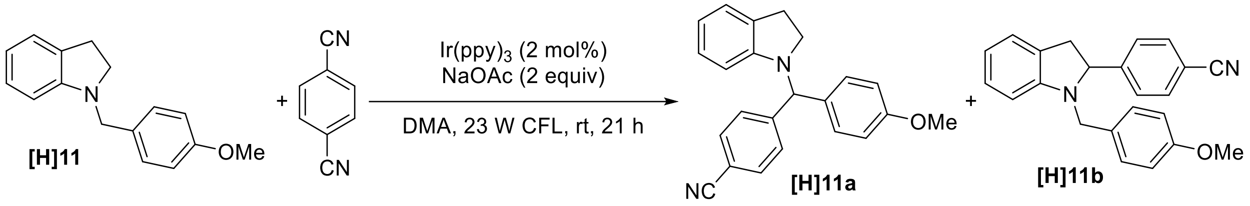

[**Caution**: The reaction releases NaCN and can produce HCN gas. Conduct reaction in a well-ventilated fume hood. Keep aqueous cyanide-containing waste basic and dispose of in accord with institutional guidelines.]

4-(Indolin-1-yl(4-methoxyphenyl)methyl)benzonitrile and 4-(1-(4-methoxybenzyl)indolin-2-yl)benzonitrile. To a tapered 10 mL microwave vial was added **1-(4-methoxybenzyl)indoline** (100 mg, 418 µmol), tris[2-(2-pyridyl)phenyl]iridium (2.19 mg, 3.34 µmol), 1,4-dicyanobenzene (21.4 mg, 167 µmol) and sodium acetate (27.4 mg, 334 µmol). The vial was sealed with a septum and purged with Ar for 10 min before adding dry DMA (669 µL). The septum was quickly replaced with a crimp cap and the vial was irradiated with a 23 W CFL for 21 h. The reaction mixture was diluted with EtOAc (5 mL) and satd Na_2CO_3 (10 mL). The layers were separated, and the aqueous layer was washed with EtOAc (3 x 5 mL). The combined organic layers were washed with satd NaCl (10 mL), dried with Na_2SO_4, filtered, and concentrated. The resultant material was purified via column chromatography using 0-5% EtOAc in hexanes to afford an inseparable mixture of **[H]11a** and **[H]11b** as a colorless oil (1 : 3.94 (**[H]11a**:**[H]11b**), 20.6 mg, 60.5 µmol, 15% combined yield).

^{1}H NMR (600 MHz, $CDCl_3$) δ 7.64 – 7.59 (m, 9H) (**[H]11b**), 7.51 (d, *J* = 8.0 Hz, 9H) (**[H]11a** & **[H]11b**), 7.17 (d, *J* = 8.6 Hz, 2H) (**[H]11a**), 7.11 – 7.03 (m, 16H) (**[H]11a** & **[H]11b**), 6.93 (t, J = 7.5 Hz, 1H) (**[H]11a**), 6.86 (d, J = 8.7 Hz, 2H) (**[H]11a**), 6.80 (d, J = 8.17 Hz, 8H) (**[H]11b**), 6.73 (t, *J* = 7.4 Hz, 4H) (**[H]11b**), 6.69 – 6.64 (m, 1H) (**[H]11a**), 6.53 (d, *J* = 7.8 Hz, 4H) (**[H]11b**), 6.11 (d, *J* = 7.9 Hz, 1H) (**[H]11a**), 5.49 (s, 1H) (**[H]11a**), 4.64 (t, *J* = 9.6 Hz, 4H) (**[H]11b**), 4.33 (d, *J* = 15.4 Hz, 4H) (**[H]11b**), 3.91 (d, *J* = 15.4 Hz, 4H) (**[H]11b**), 3.80 (s, 3H) (**[H]11a**), 3.78 (s, 11H) (**[H]11b**), 3.42 (dd, *J* = 15.8, 9.3 Hz, 4H) (**[H]11b**), 3.23 – 3.19 (m, 1H) (**[H]11a**), 3.10 (q, *J* = 8.9 Hz, 1H) (**[H]11a**), 3.01 – 2.95 (m, 1H) (**[H]11a**), 2.92 (dd, *J* = 15.9, 9.9 Hz, 5H) (**[H]11b**).

Spectral data agree with those reported.[27]

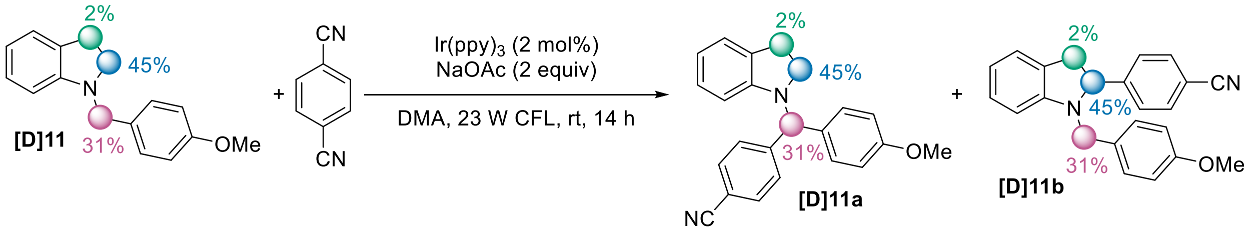

D-4-(Indolin-1-yl(4-methoxyphenyl)methyl)benzonitrile and D_4-(1-(4-methoxybenzyl)indolin-2-yl)benzonitrile. To a tapered 10 mL microwave vial was added **D- 1-(4-methoxybenzyl)indoline** (10.3 mg, 42.9 µmol) and tris[2-(2-pyridyl)phenyl]iridium (225 µg, 0.343 µmol) as stock solutions in acetone. The acetone was removed by rotary evaporation. To the vial was then added 1,4-dicyanobenzene (2.20 mg, 17.2 µmol) and sodium acetate (2.82 mg,

34.3 μmol). The vial was sealed with a septum and purged with Ar for 10 min before adding dry DMA (172 μL). The septum was quickly replaced with a crimp cap and the vial was irradiated with a 23 W CFL for 14 h. The reaction mixture was diluted with EtOAc (2 mL) and saturated aqueous Na_2CO_3. The layers were separated and the aqueous layer was washed with EtOAc (3 x 2 mL). The organic layer was washed with satd NaCl (2 mL), dried with Na_2SO_4, filtered, and concentrated to yield a yellow oil. A ~1 mM solution, with respect to initial starting material, was prepared and the crude mixture was then analyzed using a Waters ACQUITY SQ. The ratio of product **[D]11a** to **[D]11b** as determined by NMR was 1:1.24. Due to excessive fragmentation spectral deconvolution of unseparated crude material was unsuccessful.

UPLC (Acquity SQ): ACQUITY UPLC HSS C18; 1.8 μm; 150 mm x 2.1 mm; Column T = 22 °C; 0.350 mL/min. Gradient: 43.0 min from 1% MeCN/H_2O (0.1% FA) to 55% MeCN/H_2O (0.1% FA) at gradient curve = 5; 4 min gradient to 70% MeCN/H_2O (0.1% FA); 2.3 min gradient to 99% MeCN/H_2O (0.1% FA); 0.55 min at 99% MeCN/H_2O (0.1% FA); 0.15 min to 5% MeCN/H_2O (0.1% FA); 0.1 min at 5% MeCN/H_2O (0.1% FA). Detection: PDA (TWC 210 – 400 nm); MS (ESI+, *m/z* = 335 – 350, 10 V, 20 Hz).

Note: MS patterns for each regioisomer were obtained by taking a portion of each peak centroid rather than the entire peak due to poor regioisomer overlap

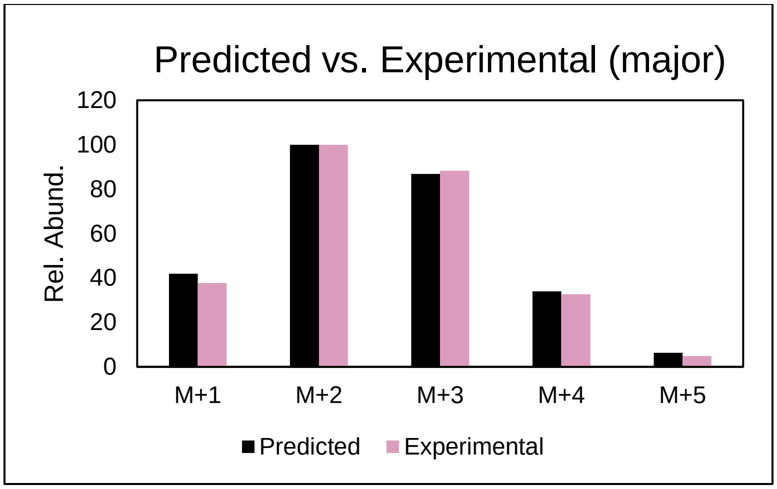

m/z	M+1	M+2	M+3	M+4	M+5
**Pred.**	41.94	100	86.83	33.94	6.32
**Exp.**	37.74	100	88.24	32.71	4.86

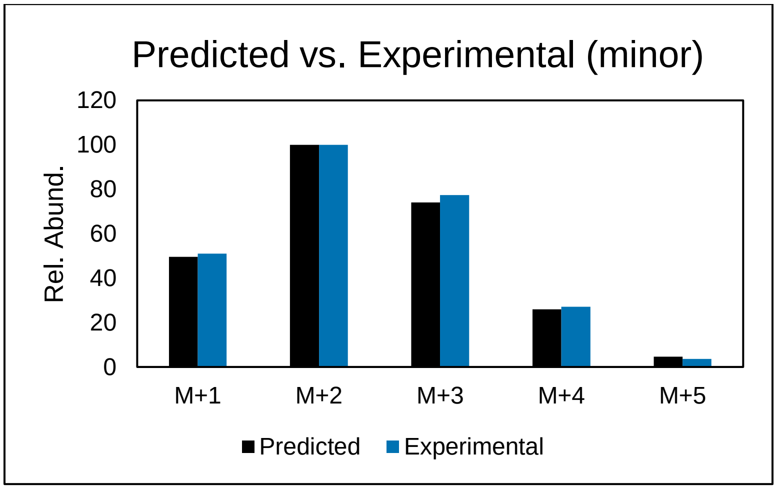

m/z	M+1	M+2	M+3	M+4	M+5
**Pred.**	49.62	100	74.10	25.95	4.67
**Exp.**	51.05	100	77.39	27.06	3.55

Fig. S153. Graphical representation of isotopic distribution for the major (**[D]11a**) (top) and minor (**[D]11b**) (bottom) arylation products **of D-1-(4-methoxybenzyl)indoline**. A table summarizing this data is provided below each graph.

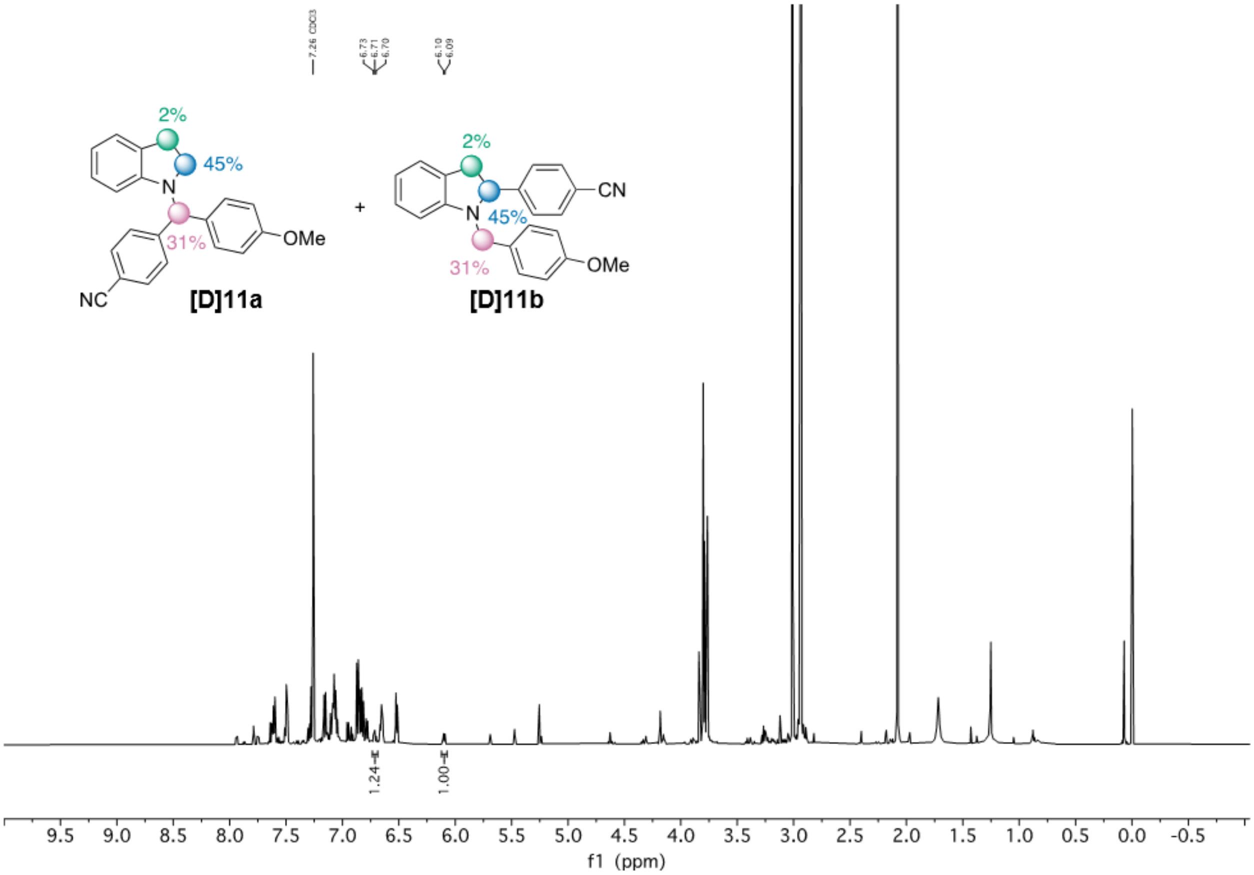

Fig. S154. **^{1}H NMR (600 MHz, Acetone)** of **[D]11a** and **[D]11b** showing product ratios.

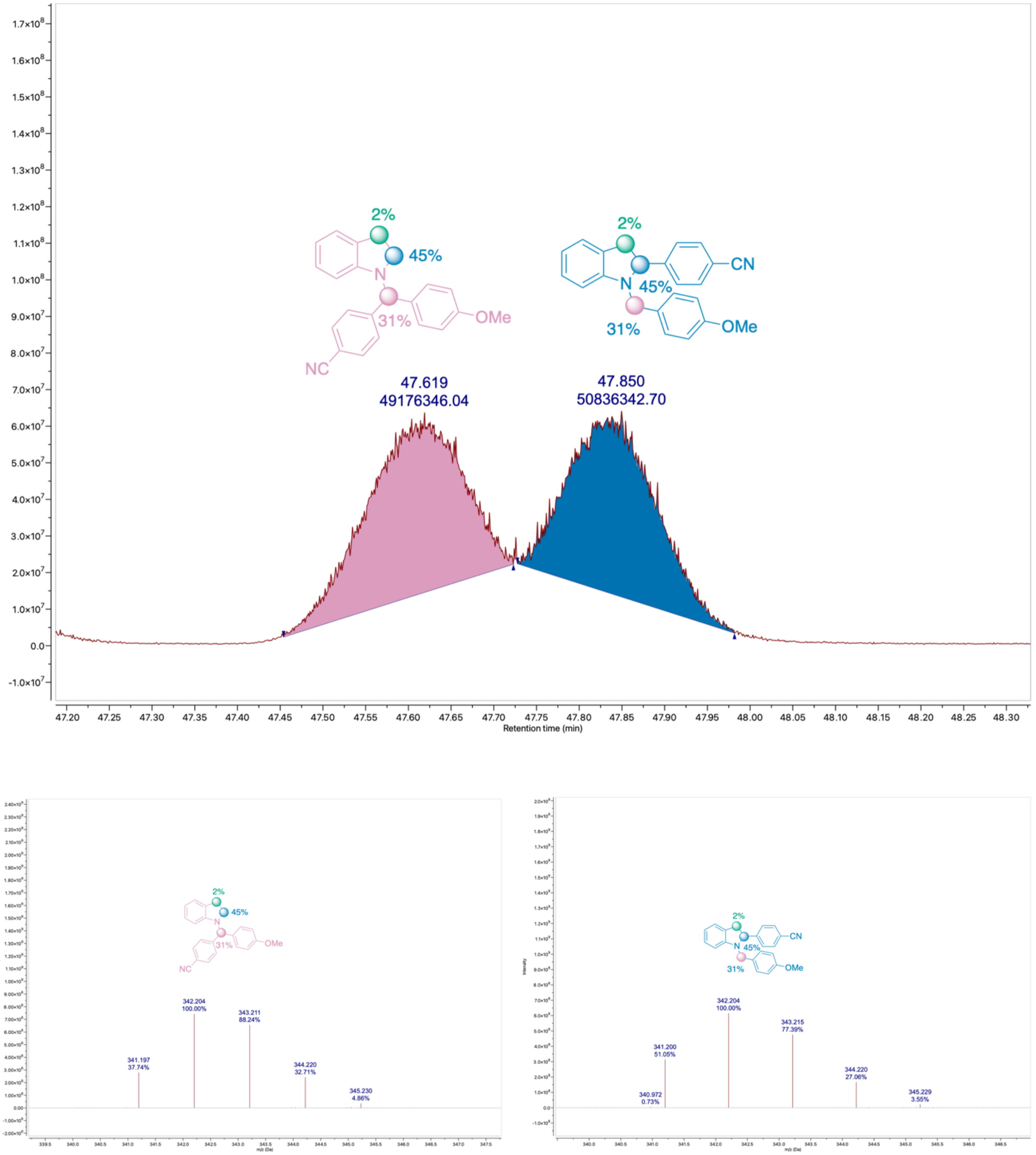

Fig. S155. D-1-(4-methoxybenzyl)indoline arylation TIC trace (top) and isotopic patterns for the of major (bottom left) and minor products (bottom right).

10. Kinetic Isotope Effects

10.1 Pyridine Kinetic Isotope Effects

10.1.1 Synthesis of Pyridine Substrates

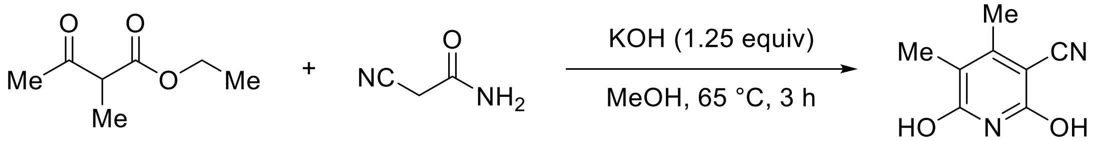

2,6-Dihydroxy-4,5-dimethylnicotinonitrile. Ethyl 2-methyl-3-oxobutanoate (7.00 g, 48.6 mmol) and 2-cyanoacetamide (4.08 g, 48.6 mmol, 1.00 equiv) was added to a 50 mL round bottom flask. Potassium hydroxide (KOH) (3.41 g, 60.7 mmol, 1.25 equiv) was dissolved in MeOH (24.5 mL) (dissolution can be exothermic, make sure solution is at room temperature before adding into starting material). The methanolic KOH solution was added dropwise over 20 min to the reaction vessel. The mixture was left to stir at rt for ~ 60 min before attaching a reflux condenser and heating the mixture to 65 ºC using an oil bath. After 3 h, a creamy white solid formed, and the reaction was removed from the oil bath. Once the reaction has cooled to rt, the slurry is filtered, and solids washed with Et_2O. The solid material is collected to yield 2,6-dihydroxy-4,5-dimethylnicotinonitrile (6.70 g, 40.8 mmol, 84% yield) as a creamy solid. The product was taken forward to the next step without further purification.

^{1}H NMR (600 MHz, DMSO) δ 2.17 (s, 3H), 1.85 (s, 3H).

Spectral data agree with those reported.[28]

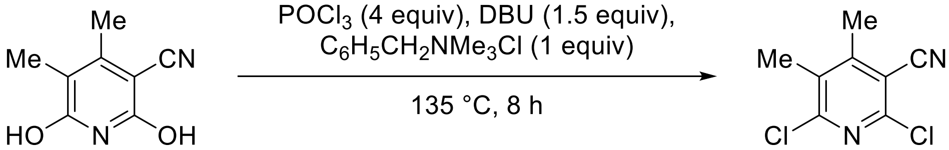

2,6-Dichloro-4,5-dimethylnicotinonitrile. 2,6-Dihydroxy-4,5-dimethylnicotinonitrile (5.00 g, 30.5 mmol, 1.00 equiv) was added to a dry 50 mL Schlenk flask fitted with a reflux condenser. 1,8-Diazabicyclo[5.4.0]undec-7-ene (DBU) (6.90 mL, 46.0 mmol,1.50 equiv) was added and the mixture was allowed to stir for 10 min resulting in the formation of a brown paste. Once the brown paste formed, benzyltrimethylammonium chloride (5.66 g, 30.5 mmol, 1.00 equiv) was added and the reflux condenser was sealed with a rubber septum, attached to a pressure equalizing line, and the system was flushed with argon. Phosphorus oxytrichloride ($POCl_3$) (11.4 mL, 122 mmol, 4.00 equiv) was injected through the sidearm and the reaction flask was heated to 135 ºC in an oil bath. After 8 h, the reaction mixture was removed from the oil bath and allowed to cool to rt. The reaction mixture was poured over ice and neutralized with 1 M KOH solution to a pH of > 8 as determined using pH paper. The aqueous mixture was extracted with CH_2Cl_2 (3 x 25 mL) and the combined organics were washed with satd NaCl (100mL), dried with anhydrous $MgSO_4$, filtered, and concentrated. The resultant material was chromatographed using 10% EtOAc in hexanes to yield pure 2,6-dichloro-4,5-dimethylnicotinonitrile (1.53 g, 30.5 mmol, 25% yield) as a white solid.

^{1}H NMR (400 MHz, $CDCl_3$) δ 2.58 (s, 3H), 2.39 (s, 3H).

Spectral data agree with those reported.[28]

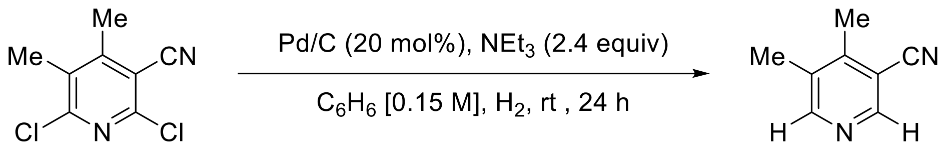

[H]12

4,5-Dimethylnicotinonitrile*.* 2,6-Dichloro-4,5-dimethylnicotinonitrile (503 mg, 2.50 mmol, 1.00 equiv) was added to a dry 25 mL round bottom flask. Dry benzene (12.5 mL, 0.2 M) and freshly distilled triethylamine (0.84 mL, 6.00 mmol, 2.40 equiv) were added. To the solution was added 10% Pd/C (53.0 mg, 0.050 mmol, 20 mol%) before sealing the reaction with a rubber septum and purging with argon. The flask was then backfilled with H_2 using a balloon and the balloon was left on the flask during the reaction. After 24 h, the reaction was deemed complete as determined by LCMS. The mixture was filtered through Celite™ and washed with CH_2Cl_2 (45 mL). The filtrate was concentrated and diluted with Et_2O (15 mL) and once again filtered through Celite™, washing with Et_2O (15 mL). The filtrate was concentrated, and the resultant material chromatographed using 0–20% EtOAc in hexanes to yield pure **4,5-dimethylnicotinonitrile** (300 mg, 1.51 mmol, 91% yield) as a colorless oil.

^{1}H NMR (600 MHz, $CDCl_3$) δ 8.61 (s, 1H), 8.48 (s, 1H), 2.46 (s, 3H), 2.30 (s, 3H).

^{13}C{^{1}H} NMR (151 MHz, $CDCl_3$) δ 153.2, 150.7, 149.1, 132,9, 116.5, 110.8, 17.7, 16.7.

HRMS (EI, TOF) m/z: $[M]^+$ calculated for $C_8H_8N_2$ 132.0687, observed 132.0695.

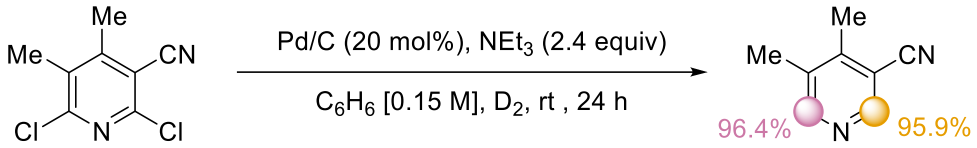

[D]12

4,5-Dimethylnicotinonitrile-2,6-*d*$_2$*.* 2,6-Dichloro-4,5-dimethylnicotinonitrile (201 mg, 1.00 mmol, 1.00 equiv) was added to a dry 25 mL round bottom flask. Dry benzene (5 mL, 0.2 M) and freshly distilled triethylamine (0.67 mL, 4.80 mmol, 4.80 equiv) were added to the flask. To the solution was added 10% Pd/C (21 mg, 0.020 mmol, 20 mol%) before sealing the reaction with a rubber septum and purging with argon. The flask was then backfilled with D_2 using a balloon and the balloon was left on the flask during the reaction. After 48 h, the reaction was deemed complete as determined by NMR spectroscopy. The mixture was subsequently filtered through Celite™ and washed with CH_2Cl_2 (45 mL). The filtrate was concentrated and diluted with Et_2O (15 mL) and once again filtered through Celite™, washing with Et_2O (15 mL). The filtrate was concentrated, and the resultant material chromatographed using 0–20% EtOAc in hexanes to yield pure **4,5-dimethylnicotinonitrile-2,6-*d*$_2$** (107 mg, 0.797 mmol, 80% yield) as a colorless oil.

^{1}H NMR (600 MHz, $CDCl_3$) δ 8.63 (s, 0.041H), 8.49 (0.036H), 2.49 (s, 3H), 2.33 (s, 3H).

^{13}C{^{1}H} NMR (151 MHz, $CDCl_3$) δ 153.2, 152.8 (t, 27.0 Hz), 150.8, 150.5 (t, 28.3 Hz), 149.3, 132.9, 116.5, 110.8, 17.7, 16.7.

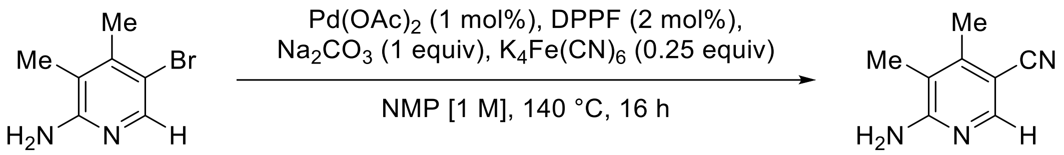

6-Amino-4,5-dimethylnicotinonitrile. To a dry 10 mL microwave vial was added sodium carbonate (158 mg, 1.49 mmol, 1.00 equiv), potassium ferrocyanide (137 mg, 0.37 mmol, 0.25 equiv), $Pd(OAc)_2$ (0.34 mg, 0.015 mmol, 1.0 mol%) and DPPF (1.7 mg, 0.03 mmol, 2.0 mol%). The vial was sealed with a crimp cap before vacuum purging 3x with argon. 5-Bromo-3,4-dimethylpyridin-2-amine (300 mg, 1.49 mmol) was weighed into a separate vial and anhydrous NMP was added (1.49 mL, 1 M). This solution was transferred to the reaction mixture and the vial was heated to 140 ºC in an aluminum heating block. After 16 h, the vial was allowed to cool to room temperature before diluting with water (15 mL). The aqueous solution was extracted with EtOAc (3 x 15 mL) and the combined organics were washed with 20% LiCl solution (3 x 10 mL), dried with anhydrous $MgSO_4$, filtered, and concentrated. The resultant material was chromatographed using 0–50% EtOAc in hexanes with 5% added NEt_3 yielding pure 6-amino-4,5-dimethylnicotinonitrile (140 mg, 0.95 mmol, 64% yield) as an off white solid.

^{1}H NMR (600 MHz, MeOD) δ 8.08 (s, 1H), 2.39 (s, 3H), 2.08 (s, 3H).

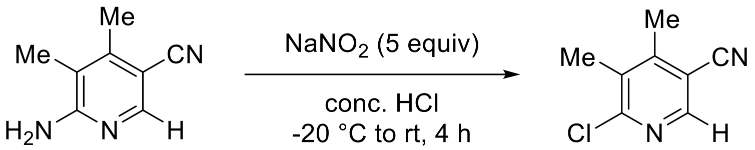

6-Chloro-4,5-dimethylnicotinonitrile. 6-Amino-4,5-dimethylnicotinonitrile (242 mg, 1.6 mmol) was added to a 40 mL scintillation vial. The vial was cooled to –20 °C using a dry ice and xylene bath before adding concentrated HCl (37%, 4.9 mL) over the course of ~2 min. Sodium nitrite (567 mg, 8.2 mmol, 5 equiv) was added in 3 portions over the course of ~10 min after which the reaction was left to warm to room temperature. After 4 h, the reaction mixture was neutralized to a pH >8 using 1 M NaOH. The neutralized solution was extracted with EtOAc (3 x 30 mL) and the combined organics were washed with satd NaCl (20 mL), dried over anhydrous $MgSO_4$, filtered, and concentrated. The resultant crude material was purified via column chromatography using 20% EtOAc in hexanes to yield 6-chloro-4,5-dimethylnicotinonitrile (144 mg, 0.86 mmol, 53% yield) as a white solid.

^{1}H NMR (400 MHz, $CDCl_3$) δ 8.45 (s, 1H), 2.56 (s, 3H), 2.42 (s, 3H).

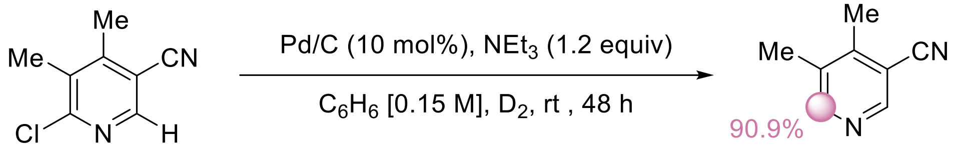

[D]12'

4,5-Dimethylnicotinonitrile-6-*d*. 6-Chloro-4,5-dimethylnicotinonitrile (189 mg, 1.13 mmol, 1.00 equiv) was added to a dry 25 mL round bottom flask with a magnetic stir bar. Dry benzene (5.7 mL, 0.2 M) and freshly distilled triethylamine (0.19 mL, 1.36 mmol, 1.20 equiv) were added to the flask. To the solution was added 10% Pd/C (12 mg, 0.11 mmol, 10 mol%) before sealing the flask with a rubber septum and purging with argon. The flask was then backfilled with D_2 using a balloon

and the balloon was left on the flask during the reaction. After 24 h, the reaction was deemed complete as determined by LCMS and the reaction mixture was subsequently filtered through Celite™ and washed with CH_2Cl_2 (45 mL). The filtrate was concentrated and diluted with Et_2O (15 mL) and once again filtered through Celite™, washing with Et_2O (15 mL). The filtrate was concentrated, and the resultant material was chromatographed using 0–20% EtOAc in hexanes to yield pure **4,5-dimethylnicotinonitrile-6-*d*** (92 mg, 0.69 mmol, 61% yield) as a colorless oil.

^{1}H NMR (600 MHz, $CDCl_3$) δ 8.65 (s, 1H), 8.50 (s, 0.091H), 2.50 (s, 3H), 2.33 (s, 3H).

^{13}C{^{1}H} NMR (151 MHz, $CDCl_3$) δ 153.1, 152.8 (t, J = 27.0 Hz), 150.7, 149.2, 133.0 – 132.9 (m, adjacent), 116.5, 110.8, 17.7, 16.6.

Spectral data correspond with that of **4,5-dimethylnicotinonitrile** shown above.

10.1.2 Synthesis of Pyridine Product Standards

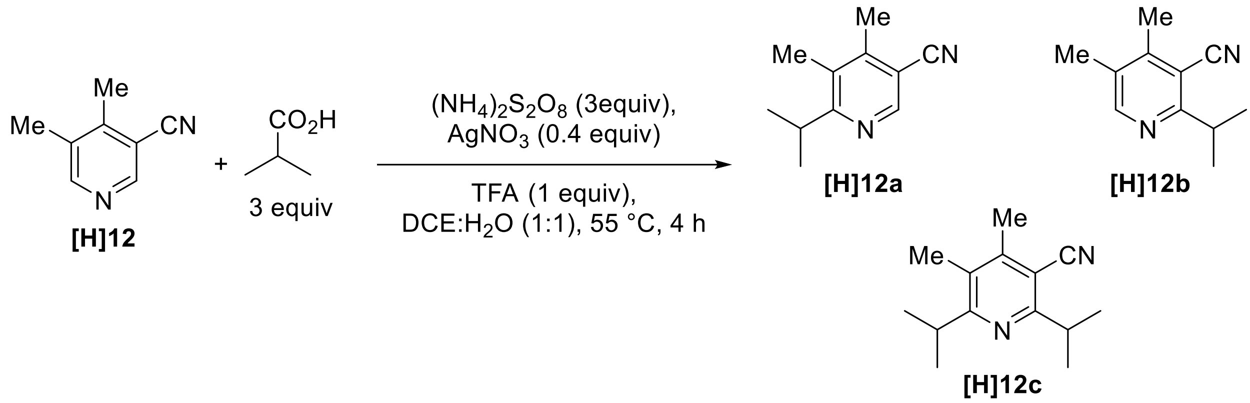

Minisci reaction of [H]12 with isobutyric acid. 4,5-Dimethylnicotinonitrile (132 mg, 1 mmol, 1 equiv) was added to a 25 mL rbf followed by $AgNO_3$ (69 mg, 0.41 mmol, 0.41 equiv) and Ammonium persulfate (709 mg, 3.00 mmol, 3.00 equiv). The solids were dispersed in water (5 mL) and $ClCH_2CH_2Cl$ (5 mL) before adding TFA (78.5 μl, 1.02 mmol, 1.00 equiv) and isobutyric acid (0.19 mL, 2.0 mmol, 2.0 equiv) with stirring. A septum with a needle outlet was attached to the flask to reduce pressure buildup (CO_2 released during reaction) and the mixture was heated to 55 °C using an oil bath. After 4 h, the reaction was deemed complete by TLC and the reaction mixture was removed from the oil bath and allowed to cool down to room temperature. Once cooled, the solution was neutralized using a 1 M NaOH solution to a pH >8. The mixture was extracted with CH_2Cl_2 (3 x 15 mL), and the combined organics were washed with satd NaCl, dried with anhydrous $MgSO_4$, filtered, and concentrated. The resultant material was chromatographed using 0-10% EtOAc in hexanes affording **[H]12a** (15 mg, 0.09 mmol, 8%) as a white solid, **[H]12b** (26 mg, 0.15 mmol, 15% yield) as a white solid, and **[H]12c** (25 mg, 0.12 mmol, 11% yield) as a white solid.

[H]12a:

^{1}H NMR (400 MHz, $CDCl_3$) δ 8.43 (s, 1H), 3.51 (hept, J = 6.7 Hz, 1H), 2.47 (s, 3H), 2.27 (s, 3H), 1.32 (d, J = 6.8 Hz, 6H).

^{13}C{^{1}H} NMR (101 MHz, $CDCl_3$) δ 167.8, 152.3, 149.9, 129.9, 116.6, 108.9, 34.5, 21.9, 18.0, 16.5.

HRMS (EI, TOF) m/z: $[M]^+$ calculated for $C_{11}H_{14}N_2$ 174.1157, observed 174.1162.

[H]12b:

^{1}H NMR (400 MHz, $CDCl_3$) δ 8.60 (s, 1H), 3.33 (hept, J = 6.7 Hz, 1H), 2.48 (s, 3H), 2.30 (s, 3H), 1.25 (d, J = 6.8 Hz, 6H).

^{13}C{^{1}H} NMR (101 MHz, $CDCl_3$) δ 168.7, 149.7, 148.7, 129.7, 117.3, 108.5, 32.1, 21.6, 18.6, 14.3.

HRMS (EI, TOF) m/z: $[M]^+$ calculated for $C_{11}H_{14}N_2$ 174.1157, observed 174.1169.

[H]12c:

^{1}H NMR (400 MHz, $CDCl_3$) δ 3.45 (hept, J = 6.8 Hz, 1H), 3.30 (hept, J = 6.7 Hz, 1H), 2.46 (s, 3H), 2.24 (s, 3H), 1.29 (d, J = 6.7 Hz, 6H), 1.24 (d, J = 6.7 Hz, 6H).

^{13}C{^{1}H} NMR (101 MHz, $CDCl_3$) δ 167.3, 166.1, 148.9, 126.3, 117.5, 106.0, 34.6, 32.3, 22.0, 21.7, 18.8, 14.0.

HRMS (EI, TOF) m/z: $[M]^+$ calculated for $C_{14}H_{20}N_2$ 216.1626, observed 216.1633.

10.1.3 Intermolecular Kinetic Isotope Experiment [D]12

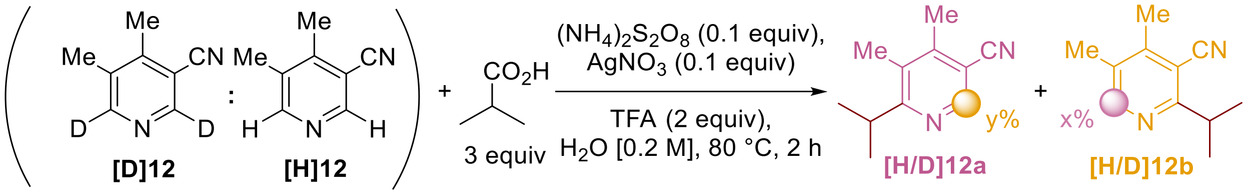

To a 5 mL scintillation vial was added stock solutions of **[D]12** (0.50 M in $CHCl_3$, 100 µL, 50 µmol) and **[H]12** (0.50 M in $CHCl_3$, 100 µL, 50 µmol) to obtain a 100 µmol of a 50:50 mixture of **[D]12** and **[H]12**. The solution was concentrated to remove the $CHCl_3$ before adding water (0.3 mL), isobutyric acid (27.2 µL, 0.300 mmol), TFA (15.4 µL, 0.200 mmol), $AgNO_3$ (0.1 M in H_2O, 100 µL, 0.01 mmol, 10 mol%), and $(NH_4)_2S_2O_8$ (0.1 M in H_2O, 100 µL, 0.01 mmol) to obtain a solution with a final volume of 0.5 mL H_2O. The vial was capped and heated to 80 °C using an aluminum heating block for 2 h. Once cooled to room temperature, 1,2,4,5-tetramethylbenzene (1.0 M in MeCN, 50 µL, 0.050 mmol) was added to each vial as an internal standard. A 1 mM sample was prepared, with respect to the starting concentration of starting material, by taking 5.5 µL of the crude solution and diluting with 995.5 µL of MeCN. The reaction was performed in duplicate with <10% conversion. Samples were analyzed using a Waters ACQUITY TQ. The isotopic distribution of **[H/D]12a** and **[H/D]12b** was used to calculate the amount of deuterium incorporated in the products using IsoPat-2 and this data was used to calculate the KIE for **12a** and **12b**. A summary of these results is provided below. Because the deuterium incorporation at both positions in **[D]12** is >95% it was treated as if fully deuterated for the below calculations.

UPLC (Acquity TQ): Aquity UPLC HSS C18; 1.8 µm; 50 mm x 2.1 mm; Column T = 35 °C; 0.500 mL/min. Gradient: 0.50 min at 5% MeCN/H_2O (0.1% FA); 0.50 min gradient to 50% MeCN/H_2O (0.1% FA); 1.60 min at 50% MeCN/H_2O (0.1% FA); 0.10 min to 70% MeCN/H_2O (0.1% FA); 0.55 min to 90% MeCN/H_2O (0.1% FA); 1 min to 95% MeCN/H_2O (0.1% FA). Detection: PDA (TWC 215 – 254 nm); MS (ESI+, m/z = 100 – 300, 30 V, 20 Hz).

[H/D]12a	M+1	M+2	M+3	#D
**t1**	100	68.65	7.26	0.358
**t2**	100	68.12	7.45	0.356

Table S12. Summary of isotopic distribution for **[H/D]12a** and the amount of deuterium as determined by IsoPat-2.

[H/D]12b	M+1	M+2	M+3	#D
**t1**	100	60.93	6.70	0.325
**t2**	100	62.51	5.95	0.332

Table S13. Summary of isotopic distribution for **[H/D]12b** and the amount of deuterium as determined by IsoPat-2.

Because products obtained by reacting with **[D]12** will contain 1 deuterium, and products reacting with **[H]12** have 0 deuterium, the amount of deuterium determined by IsoPat-2 represents the fraction of deutero product formed. As such 1-#D represents the fraction of protio product formed, the ratio of protio product over deutero product yields the KIE. A sample calculation is shown below, followed by a table summarizing the obtained KIE values.

$$\#D\ Observed = 0.358$$

$$\#D\ of\ deutero\ product = 1$$

$$\frac{\#D\ Observed}{\#D\ of\ deutero\ product} = deutero\ product\ fraction = \frac{0.358}{1} = 0.358$$

$$fraction\ protio\ product = 1 - 0.358 = 0.642$$

$$KIE = \frac{0.642}{0.358} = 1.79$$

12a	t1	t2	Avg.
**KIE**	1.79	1.81	**1.80 ± 0.01**

12b	t1	t2	Avg.
**KIE**	2.08	2.01	**2.04 ± 0.03**

Table S14. Summary of KIE data obtained for **12a** and **12b**.

10.1.4 Reactions with Singly Deuterated Pyridine

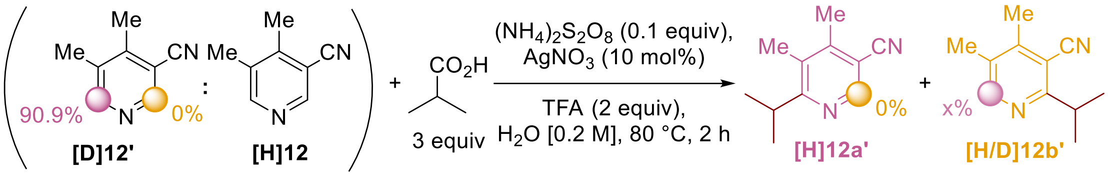

A series of five experiments were performed sequentially diluting **[D]12'** with **[H]12** to obtain ratios of 100:0, 75:25, 50:50, 25:75, and 0:100 (**[D]12'**:**[H]12**). Experiments were performed in 5 mL scintillation vials under air. Stock solutions were prepared of **[D]12'** in (0.50 M, in $CHCl_3$) and **[H]12** (0.50 M, in $CHCl_3$). Following the table below an appropriate amount of each stock solution was added to each reaction vial to obtain 0.100 mmol of combined starting materials in the appropriate ratio.

Ratio [D]12':[H]12	µL [D]12'	µL [H]12
100:0	200	0
75:25	150	50
50:50	100	100
25:75	50	150
0:100	0	200

Following the addition of substrates, each reaction vial was concentrated to remove the $CHCl_3$ before adding water (0.3 mL), isobutyric acid (27.2 µL, 0.300 mmol), TFA (15.4 µL, 0.200 mmol), $AgNO_3$ (0.1 M in H_2O, 100 µL, 0.01 mmol, 10 mol%), and $(NH_4)_2S_2O_8$ (0.1 M in H_2O, 100 µL, 0.01 mmol) to obtain a solution with a final volume of 0.5 mL H_2O. After the addition of $(NH_4)_2S_2O_8$ the vial was capped and heated to 80 °C on an aluminum heating block for 2 h. After 2 h, the vials were removed from the heating block and allowed to cool to room temperature. Once cooled to rt, 1,2,4,5-tetramethylbenzene (1.0 M in MeCN, 50 µL, 0.050 mmol) was added to each vial as an internal standard. A 5 mM sample was prepared, with respect to the starting concentration of starting material, by taking 27.5 µL of the crude solution and diluting with 972.5 µL of MeCN. These samples were analyzed using Waters ACQUITY TQ. Relative UV correction factors were used to compare molar ratios of **[H]12a** and **[H]12b** and are 1.33 and 1.00 respectively.

UPLC (Acquity TQ): Aquity UPLC HSS C18; 1.8 µm; 50 mm x 2.1 mm; Column T = 35 °C; 0.500 mL/min. Gradient: 0.50 min at 5% MeCN/H_2O (0.1% FA); 0.50 min gradient to 50% MeCN/H_2O (0.1% FA); 1.60 min at 50% MeCN/H_2O (0.1% FA); 0.10 min to 70% MeCN/H_2O (0.1% FA); 0.55 min to 90% MeCN/H_2O (0.1% FA); 1 min to 95% MeCN/H_2O (0.1% FA). Detection: PDA (TWC 215 – 254 nm); MS (ESI+, *m/z* = 100 – 300, 30 V, 20 Hz).

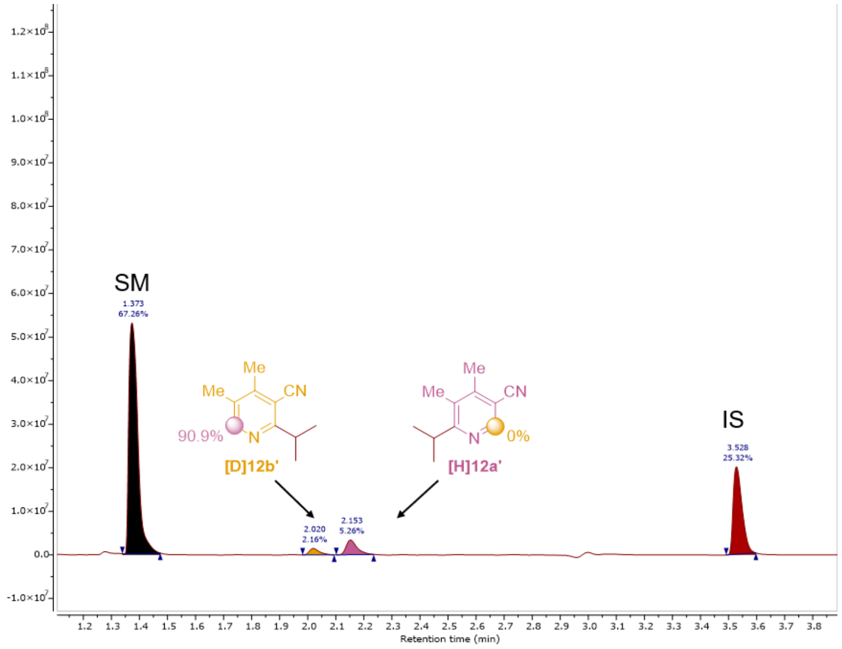

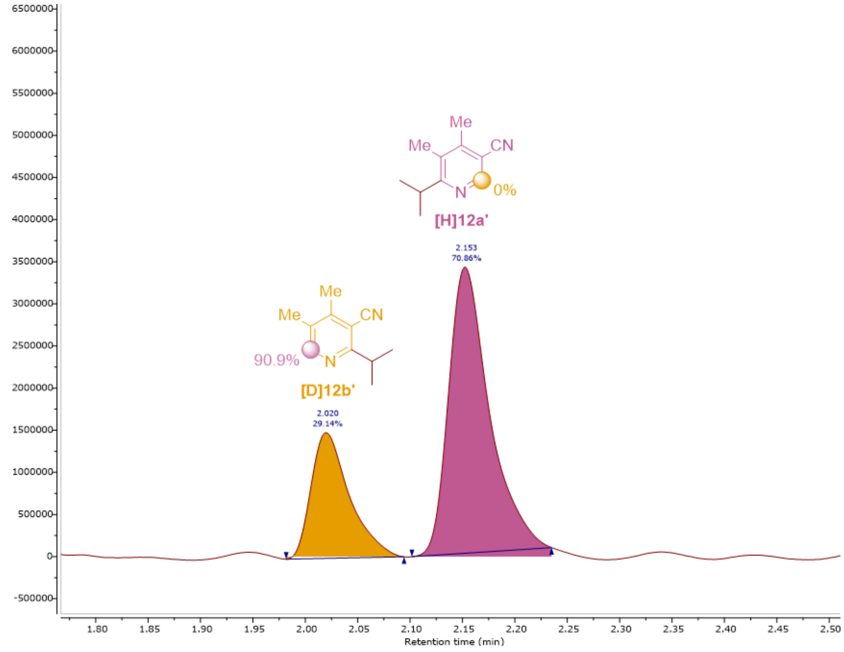

Fig. S156. Full UPLC trace for 25:75 (**[D]12'** : **[H]12**) reaction (top). Zoom in showing just product ratios for **[D]12b'** and **[H]12a'** (bottom).

D:H	Corrected Rel. % [H/D]12b’	Corrected Rel. % [H]12a’
**100:0**	49.61	62.13
**75:25**	44.71	65.87
**50:50**	40.09	69.40
**25:75**	38.17	70.86
**0:100**	34.18	73.91

Table S15. Corrected relative % of **[H/D]12b’** and **[H]12a’**

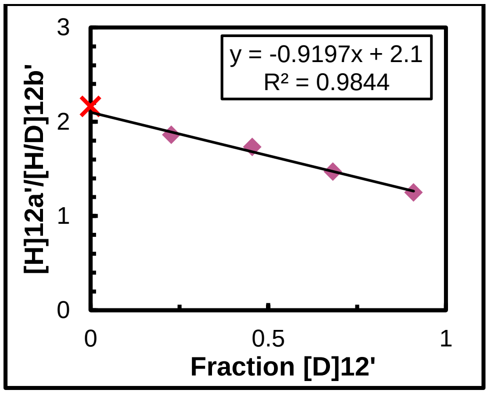

Fraction [D]12’	[H]12a’/[H/D]12b’
**1**	1.25
**0.75**	1.47
**0.50**	1.73
**0.25**	1.86
**0**	2.16

Fig. S157. (top) Plot of Fraction **[D]12’** vs. product ratio of **[H]12a’/[H/D]12b’**. (bottom) Summary of plotted data.

10.1.5 Reactions with Doubly Deuterated Pyridine

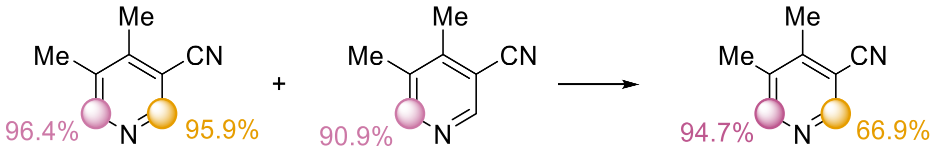

[D]12 **[D]12'** **[D]12"**

Generation of desired doubly labeled material. To obtain material with sufficient deuteration at both the C6 and C2 positions, stock solutions of **4,5-dimethylnicotinonitrile-2,6-*d*₂** (0.50 M in $CHCl_3$, 261 μL, 130 μmol) and **4,5-dimethylnicotinonitrile-6-*d*** (0.50 M in $CHCl_3$, 113 μL, 56.3 μmol) were mixed together to give **[D]12”** containing 94.7% D at C6 and 66.9% D at C2. This material was used for the subsequent dilution experiments.

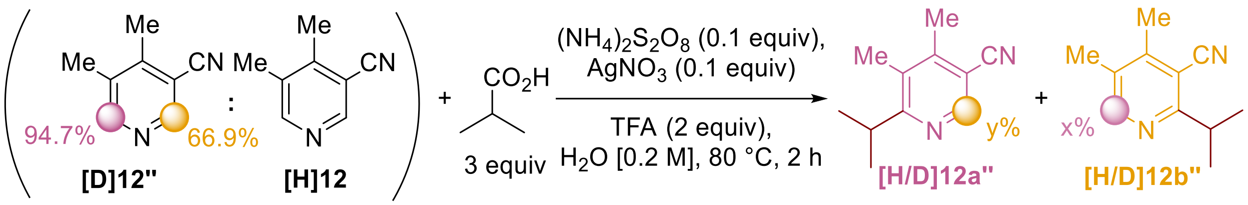

A series of four experiments were performed by sequentially diluting **[D]12”** with **[H]12** to obtain ratios of 100:0, 75:25, 50:50, 25:75 (**[D]12”**:**[H]12**). Experiments were performed in 5 mL scintillation vials under air. Stock solutions were prepared of **[D]12”** (0.50 M, in $CHCl_3$) and **[H]12** (0.50 M, in $CHCl_3$). Following the table below an appropriate amount of each stock solution was added to each reaction vial to obtain 0.100 mmol of combined starting materials in the appropriate ratio.

Ratio [D]12”:[H]12	µL [D]12”	µL [H]12
100:0	200	0
75:25	150	50
50:50	100	100
25:75	50	150
0:100	0	200

Following the addition of substrates each reaction vial was concentrated to remove the $CHCl_3$ before adding water (0.3 mL), isobutyric acid (27.2 µL, 0.300 mmol), TFA (15.4 µL, 0.200 mmol), $AgNO_3$ (0.1 M in H_2O, 100 µL, 0.01 mmol, 10 mol%), and $(NH_4)_2S_2O_8$ (0.1 M in H_2O, 100 µL, 0.01 mmol) to obtain a solution with a final volume of 0.5 mL H_2O. After the addition of $(NH_4)_2S_2O_8$ the vial was capped and heated to 80 °C on an aluminum heating block for 2 h. After 2 h, the vials were removed from the heating block and allowed to cool to rt. Once cooled to rt, 1,2,4,5-tetramethylbenzene (1.0 M in MeCN, 50 µL, 0.050 mmol) was added to each vial as an internal standard. A 5 mM sample was prepared, with respect to the starting concentration of starting material, by taking 27.5 µL of the crude solution and diluting with 972.5 µL of MeCN. These samples were analyzed using Waters ACQUITY TQ. Relative UV correction factors were used to compare molar ratios of **[H]12a** and **[H]12b** and are 1.33 and 1.00 respectively.

UPLC (Acquity TQ): Aquity UPLC HSS C18; 1.8 µm; 50 mm x 2.1 mm; Column T = 35 °C; 0.500 mL/min. Gradient: 0.50 min at 5% MeCN/H_2O (0.1% FA); 0.50 min gradient to 50% MeCN/H_2O (0.1% FA); 1.60 min at 50% MeCN/H_2O (0.1% FA); 0.10 min to 70% MeCN/H_2O (0.1% FA); 0.55 min to 90% MeCN/H_2O (0.1% FA); 1 min to 95% MeCN/H_2O (0.1% FA). Detection: PDA (TWC 215 – 254 nm); MS (ESI+, *m/z* = 100 – 300, 30 V, 20 Hz).

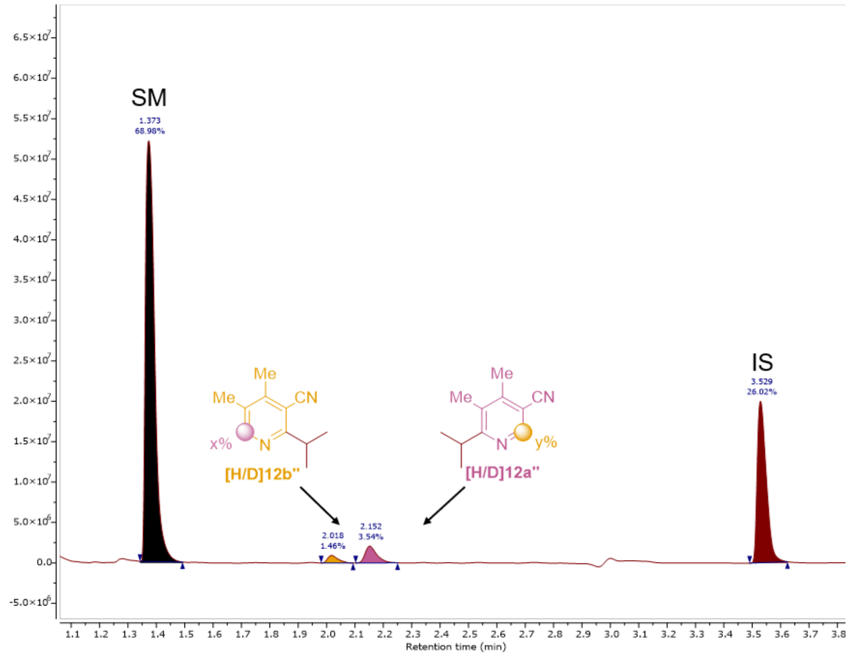

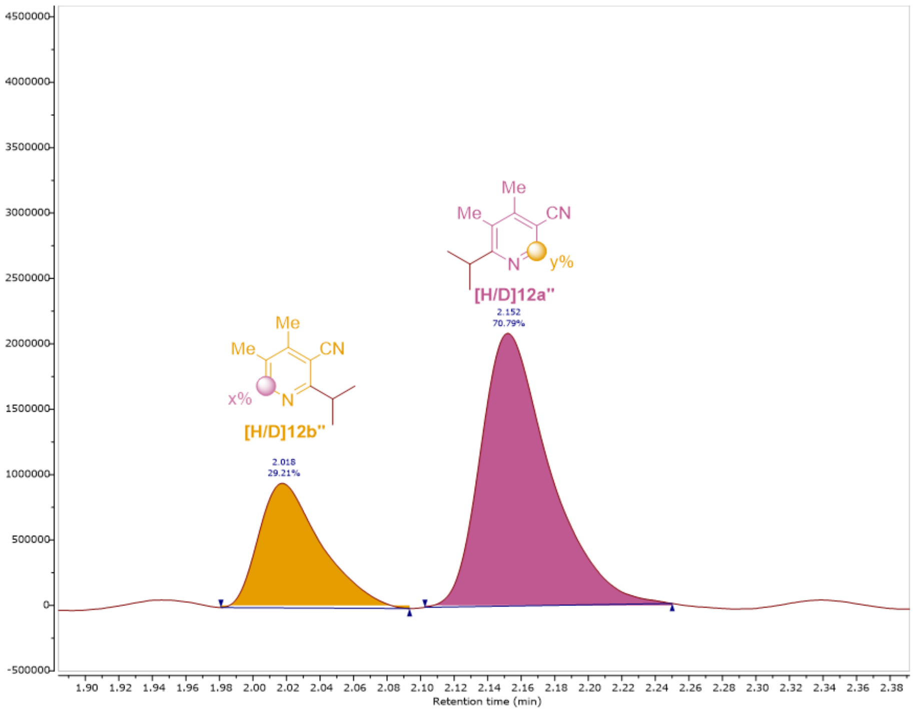

Fig. S158. Full UPLC trace for 100:0 (**[D]12"** : **[H]12**) reaction (top). Zoom in showing just product ratios for **[H/D]12b"** and **[H/D]12a"** (bottom).

[D]12”:[H]12	Corrected Rel. % [H/D]12b”	Corrected Rel. % [H]12a”
**100:0**	34.90	70.79
**75:25**	36.06	72.12
**50:50**	37.16	72.95
**25:75**	38.94	73.82

Table S16. Corrected relative % of **[H/D]12b’** and **[H]12a’**

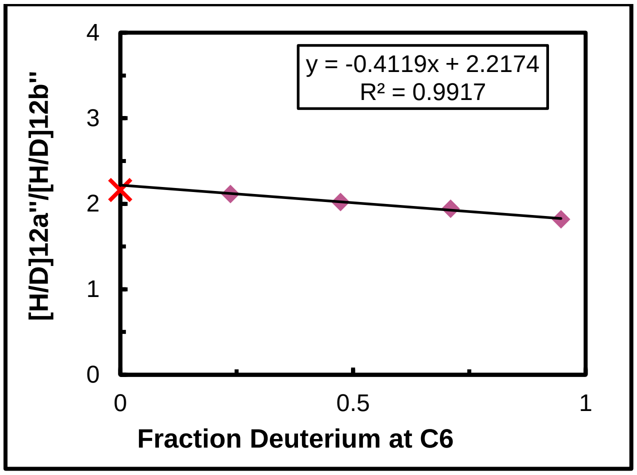

Fraction D at C6	[H/D]12a”/[H/D]12b”
0.947	1.817
0.710	1.941
0.473	2.021
0.237	2.115

Fig. S159. Plot of Fraction D at C6 vs. product ratio of **[H/D]12a”/[H/D]12b”** (top). Summary of plotted data (bottom).

10.1.6 Derivations of KIE with Singly Deuterated Pyridine

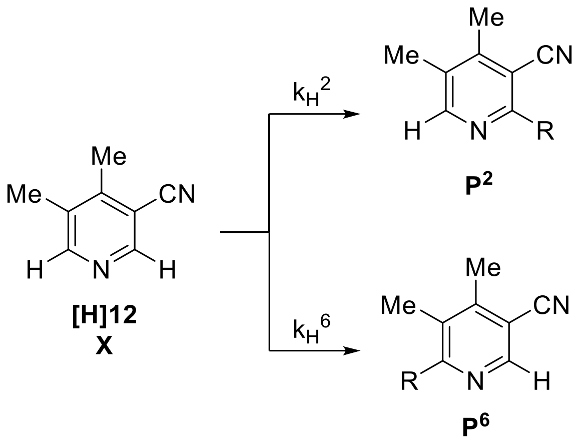

Fig. S160. Representative reaction of protio material.

In the above reaction, **[H]12** can form two possible products: P^2 and P^6. All reactions were run to low conversion (<10%) to allow the use of initial rates. Within a given reaction vial, t is the same for both products allowing canceling. By using the same reaction time for a series of reactions, t will cancel similarly which allow ratios from different reaction vials to be compared. The rate of formation of each of these products is thus approximated with equations (1) and (2) below.

$$[P^2] = k_H^2[X]t \tag{1}$$

$$[P^6] = k_H^6[X]t \tag{2}$$

$$\frac{[P^6]}{[P^2]} = \frac{k_H^6[X]t}{k_H^2[X]t} = \frac{k_H^6}{k_H^2} \tag{3}$$

From equations (1) and (2) we obtain equation (3) which shows that the ratio of each product formed is equal to the ratio of the rates of their formation.

When moving towards partially labeled material multiple species are involved in the formation of both products shown below in **Fig. S161.**

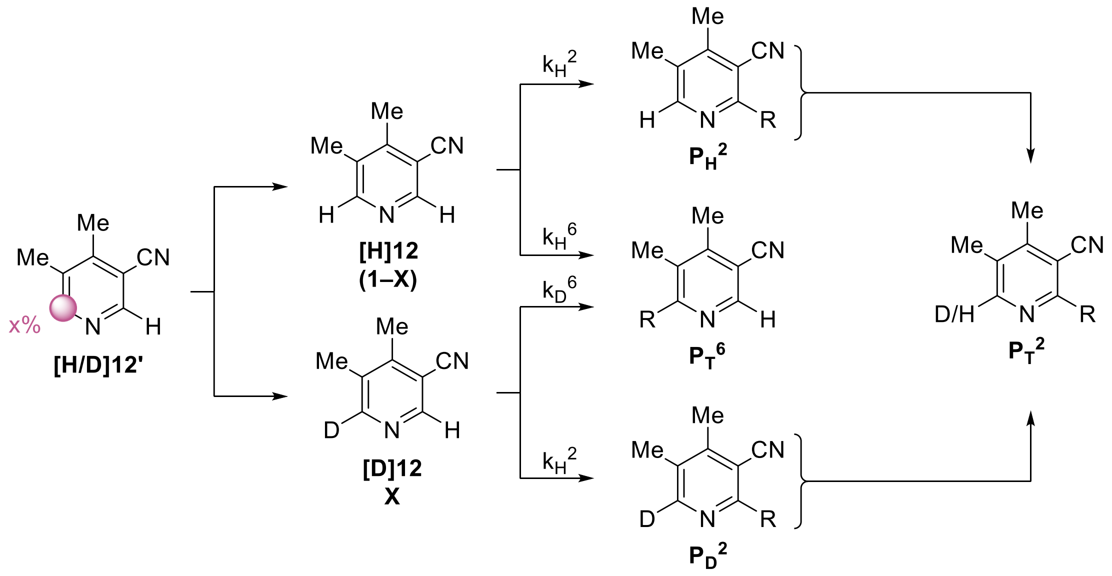

Fig. S161. Reaction scheme depicting the speciation of **[H/D]12'** and subsequent rates of product formation.

In the figure above partially labeled compound **[H/D]12'** consists of **[D]12** (labeled X) and **[H]12** (labeled 1-X). Both **[D]12** and **[H]12** can form $\mathbf{P_T^6}$ at rates k_D^6 and k_H^6 respectively, however because the deuterium label is lost it is not possible to tell where this product originated from. Alternatively, **[D]12** and **[H]12** can go on to form P_T^2 both at a rate equal to k_H^2 (assuming no secondary kinetic isotope effect from the deuterium label at C6). Modifying equations (1) and (2) generates equations (4) and (5). The ratio of these two equations equation (6), the analog of equation (3). The time component cancels which is reasonable as these values arise from one reaction. In order to generate sufficient data for fitting, a series of reactions is conducted (all for the same amount of time) with the same overall concentration of **[H/D]12'** but with different ratios of **[H]12' : [D]12'**.

$$[P_T^2] = k_H^2[(1-X)]t + k_H^2[(X)]t = k_H^2 t \tag{4}$$

$$[P_T^6] = k_H^6[(1-X)]t + k_D^6[(X)]t \tag{5}$$

$$\frac{[P_T^6]}{[P_T^2]} = \frac{k_H^6[(1-X)]}{k_H^2} + \frac{k_D^6[(X)]}{k_H^2} \tag{6}$$

Rearrangement of equation (6) yields equation (7) which can be further rearranged to the form of a linear equation (y = mx + b). Here, x = X which is the fraction of deutero material (**[D]12**) in the dilution (from **Fig. S159**.). And, y to the experimentally observed ratio of P_T^6/P_T^2. The slope of the line m is equal to $\frac{k_D^6 - k_H^6}{k_H^2}$ while the y-intercept is equal to $\frac{k_H^6}{k_H^2}$ or the product ratio obtained using only protio material. With the values for obtained for m and b it is now possible to solve for the KIE at C6 or $\frac{k_H^6}{k_D^6}$.

$$\frac{[P_T^6]}{[P_T^2]} = \frac{k_H^6}{k_H^2} - \frac{k_H^6[(X)]}{k_H^2} + \frac{k_D^6[(X)]}{k_H^2} \tag{7}$$

$$\frac{[P_T^6]}{[P_T^2]} = [(X)]\left(\frac{k_D^6 - k_H^6}{k_H^2}\right) + \frac{k_H^6}{k_H^2} \tag{8}$$

Plotting the data as shown in **Fig. S157** for a series of reactions all conducted for the same amount of time above generates m = –1 and b = 2.145 (R^2 0.943). Using these values yields equations (9) and (10). Substituting the value from equation (10) into equation (9) generates the term shown in equation (11). Multiplying equation (10) by the inverse of this term as in equation (12) provides the KIE for the 6-position.

$$\frac{k_D^6 - k_H^6}{k_H^2} = -0.9197 \tag{9}$$

$$\frac{k_H^6}{k_H^2} = \mathbf{2.100} \tag{10}$$

$$\frac{k_D^6}{k_H^2} - 2.100 = -0.9197 \therefore \frac{k_D^6}{k_H^2} = 1.180 \tag{11}$$

$$\frac{k_H^6}{k_H^2}\frac{k_H^2}{k_D^6} = \frac{2.100}{1.180} = \frac{k_H^6}{k_D^6} = \mathbf{1.78} \tag{12}$$

10.1.7 Derivations of KIE with Doubly Deuterated Pyridine

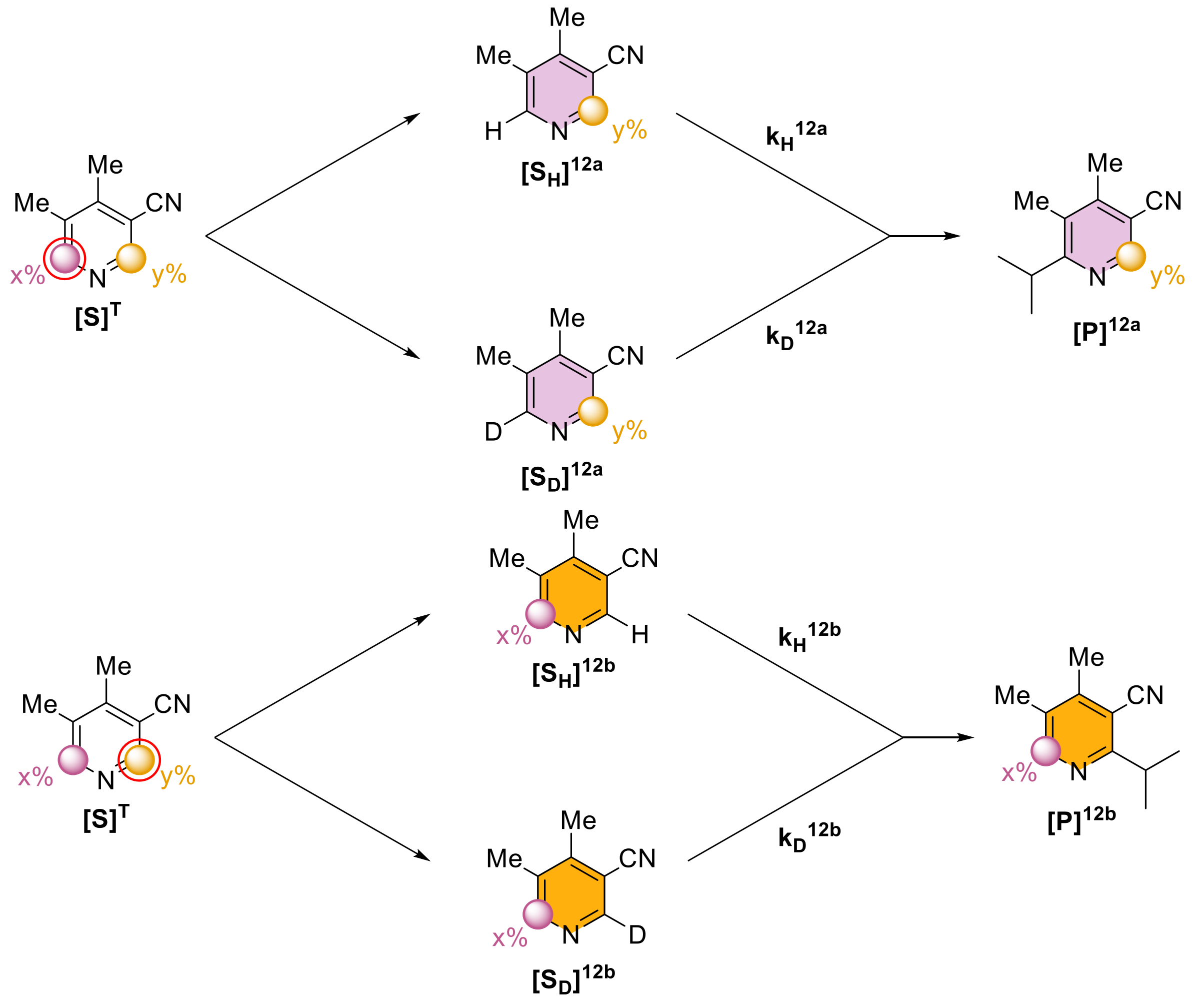

Fig. S162. Derivation of rate equations for a mixture of **4,5-Dimethylnicotinonitrile-2,6-*d*$_2$** and **4,5-Dimethylnicotinonitrile.**

When focusing on one position, such as the C2 position, the starting material **[S]^T** can be represented as protio (**[S$_H$]^{12a}**) and deutero (**[S$_D$]^{12a}**) species, both of which form product **[P]^{12a}** at rates **k$_H$^{12a}** and **k$_D$^{12a}** respectively. As such the concentration of **[P]^{12a}** at time t is defined by equation (13). Performing a similar type of analysis on the C6 position yields equation (14).

$$[P]^{12a} = (k_H^{12a}[S_H]^{12a} + k_D^{12a}[S_D]^{12a})t \quad (13)$$

$$[P]^{12b} = \left(k_H^{12b}[S_H]^{12b} + k_D^{12b}[S_D]^{12b}\right)t \quad (14)$$

When observing the ratio of C6 (**[H/D]12a**) product over C2 (**[H/D]12b**) product across a series of dilutions halted at the same time we form equation (15) from equations (13) and (14). Equation (15) can be rearranged to yield equation (16) which can be expressed in matrix format (equation (17)) when the reaction is performed *n* times by diluting the isotopically labeled starting material with **[S$_H$]** while maintaining the same overall concentration of **[S]^T**.

$$\frac{[P]^{12a}}{[P]^{12b}} = \frac{k_H^{12a}[S_H]^{12a} + k_D^{12a}[S_D]^{12a}}{k_H^{12b}[S_H]^{12b} + k_D^{12b}[S_D]^{12b}} \tag{15}$$

$$\frac{[P]^{12a}[S_H]^{12b}}{[P]^{12b}} = \frac{k_H^{12a}}{k_H^{12b}}[S_H]^{12a} + \frac{k_D^{12a}}{k_H^{12b}}[S_D]^{12a} - \frac{k_D^{12b}}{k_H^{12b}}\frac{[P]^{12a}[S_D]^{12b}}{[P]^{12b}} \tag{16}$$

$$\begin{bmatrix} ([S_H]^{12a})^1 & ([S_D]^{12a})^1 & \left(-\frac{[P]^{12a}[S_D]^{12b}}{[P]^{12b}}\right)^1 \\ \vdots & \vdots & \vdots \\ ([S_H]^{12a})^n & ([S_D]^{12a})^n & \left(-\frac{[P]^{12a}[S_D]^{12b}}{[P]^{12b}}\right)^n \end{bmatrix} \begin{bmatrix} \frac{k_H^{12a}}{k_H^{12b}} \\ \frac{k_D^{12a}}{k_H^{12b}} \\ \frac{k_D^{12b}}{k_H^{12b}} \end{bmatrix} = \begin{bmatrix} \left(\frac{[P]^{12a}[S_H]^{12b}}{[P]^{12b}}\right)^1 \\ \vdots \\ \left(\frac{[P]^{12a}[S_H]^{12b}}{[P]^{12b}}\right)^n \end{bmatrix} \tag{17}$$

Creating multiple equations (16) at the series of dilutions performed and using the solver function in Excel as discussed previously will solve for the values of $\frac{k_H^{12a}}{k_H^{12b}}$, $\frac{k_D^{12a}}{k_H^{12b}}$ and $\frac{k_D^{12b}}{k_H^{12b}}$. Alternatively, equation (17) can be solved using OLS regression as previously discussed. The inverse of the value obtained for $\frac{k_D^{12b}}{k_H^{12b}}$ is the KIE for **12b**, the value obtained for $\frac{k_H^{12a}}{k_H^{12b}}$ is the predicted protio regioselctivity of **12a**/**12b,** and dividing the value obtained for $\frac{k_H^{12a}}{k_H^{12b}}$ by the value of $\frac{k_D^{12a}}{k_H^{12b}}$ yields the KIE for **12a**. Because this equation utilizes product ratios, the relative ratio of **[H/D]12a** and **[H/D]12b** can be used instead of concentrations. A summary of the relevant values as well as the constructed matrix using equation (17) is shown below.

[H/D]12a Dilution (D:H)	$[P]^{12a}$	$[S_H]^{12a}$	$[S_D]^{12a}$
**100:0**	0.355	0.066	0.134
**75:25**	0.34	0.1	0.1
**50:50**	0.331	0.133	0.067
**25:75**	0.321	0.167	0.033

[H/D]12b Dilution (D:H)	$[P]^{12a}$	$[S_H]^{12a}$	$[S_D]^{12a}$
**100:0**	0.645	0.011	0.189
**75:25**	0.66	0.058	0.142
**50:50**	0.669	0.105	0.095
**25:75**	0.679	0.153	0.047

$$\begin{bmatrix} 0.0110 & 0.1890 & -0.2435 \\ 0.058 & 0.1420 & -0.1941 \\ 0.1050 & 0.0950 & -0.1354 \\ 0.1530 & 0.0470 & -0.0698 \end{bmatrix} \begin{bmatrix} \frac{k_H^{12a}}{k_H^{12b}} \\ \frac{k_D^{12a}}{k_H^{12b}} \\ \frac{k_D^{12b}}{k_H^{12b}} \end{bmatrix} = \begin{bmatrix} 0.1199 \\ 0.1940 \\ 0.2688 \\ 0.3532 \end{bmatrix}$$

Fig. S163. Summary of relevant values for **[H/D]12a** (top). Summary of relevant values for **[H/D]12b** (middle). Constructed matrix using equation (17) and values obtained from the tables (bottom).

	$\frac{k_H^{12a}}{k_H^{12b}}$	$\frac{k_D^{12a}}{k_H^{12b}}$	$\frac{k_D^{12b}}{k_H^{12b}}$
**OLS**	2.18	1.21	0.541
**Solver**	2.21	1.54	0.804

Table S17. Summary of values obtained through OLS regression (OLS), and the Excel Solver (Solver).

	KIE (12a)	KIE (12b)	12a/12b
**OLS**	1.81	1.85	2.18
**Solver**	1.43	1.24	2.21
**True Values**	1.80	2.04	2.16

Table S18. Summary KIE and regioselectivity values obtained through OLS regression (OLS), Excel Solver (Solver), and experimentally obtained values (True Values).

10.2 Nateglinide Methyl Ester Kinetic Isotope Effects

10.2.1 Intermolecular Kinetic Isotope Experiment Nateglinide Methyl Ester

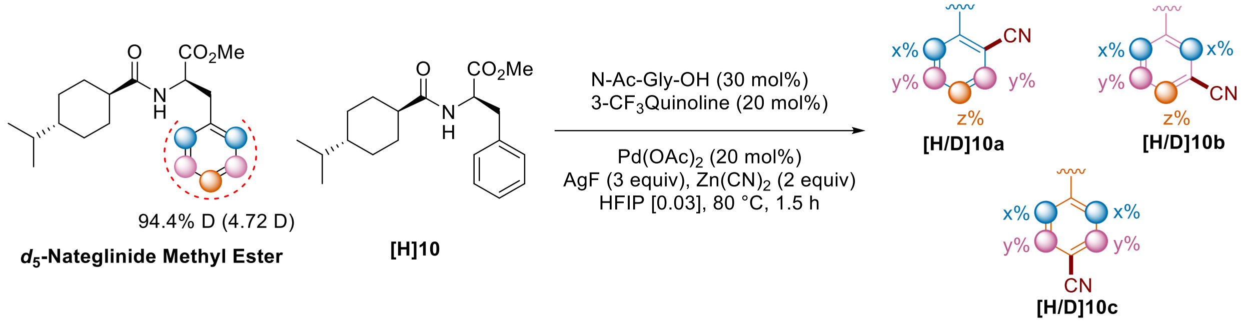

Experiments were set up inside a hood under air and performed in triplicate. Reactions were performed in three 1 mL glass vial (8 x 30 mm) equipped with a stir bar. To each 1 mL vial was added stock solutions of d_5-nateglinide methyl ester in $ClCH_2CH_2Cl$ (35 µL, 0.074 mg, 2.2 µmol) and H-nateglinide methyl ester in $ClCH_2CH_2Cl$ (35 µL, 0.73 mg, 2.2 µmol). Each reaction vial was subsequently charged with 3-CF_3-quinoline (9.74 mM in $ClCH_2CH_2Cl$, 92.9 µL, 0.178 mg, 0.905 µmol), N-Ac-Gly-OH (15.7 mM in MeOH, 86.6 µL, 0.159 mg, 1.36 µmol). AgF and $Zn(CN)_2$ were added via slurry addition as follows: AgF (70.3 mg/mL in $ClCH_2CH_2Cl$, 24.5 µL, 1.72 mg, 13.6 µmol); $Zn(CN)_2$ (68.4 mg/mL in $ClCH_2CH_2Cl$, 15.5 µL, 1.06 mg, 9.05 µmol). The vials were concentrated with a Genevac (vacuum centrifugation) with a 1 h cycle at 25 °C. The vials were placed in the center of a 96-well aluminum block and the vials were covered with a PTFA film and sealed with an aluminum top plate containing a rubber gasket with the use of 9 screws. After sealing the plate, it was placed on a tumble stirrer heated to 80 °C. After 1.5 h, the plate was removed from the tumble stirrer and placed between two similarly sized 96-well aluminum blocks to assist in cooling the plate. Once cooled to room temperature, the top plate was removed and each vial was dosed with a stock solution of 1,3,5-trimethoxybenzene (10.49 mM in MeCN, 172 µL, 1.80 µmol). The vials were concentrated with a Genevac (vacuum centrifugation) with a 1 h cycle at 25 °C and the vials were diluted with MeCN (400 µL). For each vial, the resultant solution (212 µL) was transferred to a 96-well plastic well plate and further diluted with MeCN (588 µL). The plate was then centrifuged for 30 min to allow any precipitate to settle before analysis. This solution was analyzed using a Waters ACQUITY SQ. Reactions were run to <10% conversion. Each triplicate was analyzed twice by UPLC, and the total #D of each isomer was calculated using IsoPat-2. These results are summarized below.

UPLC (Acquity SQ): ACQUITY UPLC HSS C18; 1.8 µm; 150 mm x 2.1 mm; Column T = 40 °C; 0.350 mL/min. Gradient: 5.00 min at 54% MeCN/H_2O (0.1% FA); 2 min gradient to 58% MeCN/H_2O (0.1% FA); 1 min gradient to 95% MeCN/H_2O (0.1% FA); 0.85 min at 95% MeCN/H_2O (0.1% FA); 0.05 min to 5% MeCN/H_2O (0.1% FA); 0.1 min at 5% MeCN/H_2O (0.1% FA). Detection: PDA (TWC 190 – 260 nm); MS (ESI+, m/z = 320 – 380, 30 V, 20 Hz).

[H/D]10a	M+1	M+2	M+3	M+4	M+5	M+6	#D
**t1-1**	100	22.07	2.42	8.28	38.4	6.82	1.177
**t1-2**	100	21.78	2.73	8.3	38.88	7.06	1.189
**t2-1**	100	21.79	2.39	8.62	40.14	7.35	1.214
**t2-2**	100	21.53	2.46	9.33	40.33	7.15	1.224

**t3-1**	100	21.54	2.81	9.3	42.5	8.18	1.267
**t3-2**	100	21.98	2.69	9.74	43.38	8.46	1.285

[H/D]10b	M+1	M+2	M+3	M+4	M+5	M+6	#D
**t1-1**	100	22.03	2.75	7.3	21.19	3.44	0.784
**t1-2**	100	22.23	2.57	7.2	22.35	3.74	0.811
**t2-1**	100	21.68	2.8	7.31	23.07	3.74	0.831
**t2-2**	100	21.8	2.57	7.17	23.19	3.79	0.831
**t3-1**	100	21.76	3.04	7.88	24.33	4.11	0.87
**t3-2**	100	21.64	2.72	6.97	24.41	4.29	0.861

[H/D]10c	M+1	M+2	M+3	M+4	M+5	M+6	#D
**t1-1**	100	21.69	2.62	8.19	30.18	4.34	1.006
**t1-2**	100	21.44	2.21	7.65	29.9	4.25	0.993
**t2-1**	100	21.43	2.43	7.14	32.95	4.38	1.053
**t2-2**	100	21.8	2.6	7.59	32.26	5.18	1.047
**t3-1**	100	22.01	2.27	8.77	34.98	5.35	1.111
**t3-2**	100	21.97	2.72	8.47	34.34	5.69	1.099

Table S19. Summary of M+1–M+6 values for **[H/D]10a-c**. Calculated #D for each trial is shown on the right of each row.

Because the starting d_5-nateglinide methyl ester contained 4.72 D, as determined by IsoPat-2, products formed from reacting with all deutero material are assumed to contain 3.72 D. Dividing the #D observed experimentally by 3.72 yields the product fraction of deutero material which can then be used to determine the ratio of protio/deutero and thus the KIE. A sample calculation is shown below followed by a summary of KIE values.

$$\#D\ Observed = 1.177$$

$$\#D\ of\ deutero\ product = 3.72$$

$$\frac{\#D\ Observed}{\#D\ of\ deutero\ product} = deutero\ product\ fraction = \frac{1.177}{3.72} = 0.316$$

$$fraction\ protio\ product = 1 - 0.316 = 0.684$$

$$KIE = \frac{0.684}{0.316} = 2.16$$

10a	t1-1	t1-2	t2-1	t2-2	t3-1	t3-2	Avg.
**KIE**	2.16	2.13	2.06	2.04	1.94	1.89	**2.04 ± 0.10**

10b	t1-1	t1-2	t2-1	t2-2	t3-1	t3-2	Avg.
KIE	3.74	3.59	3.48	3.48	3.28	3.32	**3.48 ± 0.16**

10c	t1-1	t1-2	t2-1	t2-2	t3-1	t3-2	Avg.
KIE	2.70	2.75	2.53	2.55	2.35	2.38	**2.54 ± 0.15**

Table S20. Summary of KIE data for **10a**, **10b**, and **10c.**

10.2.2 Reactions of Triply Deuterated Nateglinide Methyl Ester

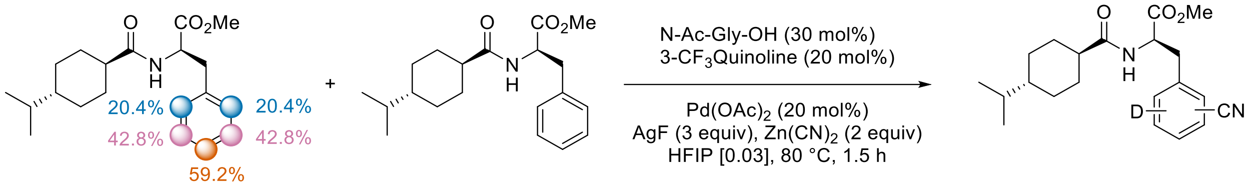

A series of five experiments were performed, each in triplicate, sequentially diluting D-nateglinide methyl ester with the H-nateglinide methyl ester. The ratios of D:H were: 100:0; 75:25; 50:50; 25:75; 0:100. For each reaction, the same total concentration of H/D-nateglinide methyl ester was used.

Trial	100:0 (D:H)	75:25 (D:H)	50:50 (D:H)	25:75 (D:H)	0:100 (D:H)
1	A1-x[a]	A2-x[a]	A3-x[a]	A4-x[a]	A5-x[a]
2	B1-x[a]	B2-x[a]	B3-x[a]	B4-x[a]	B5-x[a]
3	C1-x[a]	C2-x[a]	C3-x[a]	C4-x[a]	C5-x[a]

[a]Duplicates of each trial were conducted, trial 1 x=1, trial 2, x=2.

Table S21. Summary of plate layout.

Experiments were conducted inside a hood under air. Stock solutions were prepared of D-nateglinide methyl ester in $ClCH_2CH_2Cl$ (4.5 µmol per 93.2 µL), and H-nateglinide methyl ester in $ClCH_2CH_2Cl$ (4.5 µmol per 70.0 µL). Each vial was dosed with the appropriate ratio of D-nateglinide methyl ester and H-nateglinide methyl ester shown by the table below.

Ratio D:H	µL (D)	µL (H)
100:0	93.2	0
75:25	69.9	17.5
50:50	46.6	35.0
25:75	23.3	52.5
0:100	0	70.0

After the addition of both deutero and protio substrates each vial contained (1.5 mg, 4.5 µmol) of D/H-nateglinide methyl ester. Each reaction vial was subsequently charged with 3-CF_3-quinoline (9.74 mM in $ClCH_2CH_2Cl$, 92.9 µL, 0.178 mg, 0.905 µmol), N-Ac-Gly-OH (15.7 mM in MeOH, 86.6 µL, 0.159 mg, 1.36 µmol). AgF and $Zn(CN)_2$ were added via slurry addition as follows: AgF (70.3 mg/mL in $ClCH_2CH_2Cl$, 24.5 µL, 1.72 mg, 13.6 µmol); $Zn(CN)_2$ (68.4 mg/mL in $ClCH_2CH_2Cl$, 15.5 µL, 1.06 mg, 9.05 µmol). The vials were concentrated with a Genevac (vacuum centrifugation) with a 1 h cycle at 25 °C. The vials were placed in the center of a 96-well aluminum

block and the vials were covered with a PTFA film and sealed with an aluminum top plate containing a rubber gasket with the use of 9 screws. After sealing the plate, it was placed on a tumble stirrer heated to 80 °C. After 1.5 h, the plate was removed from the tumble stirrer and placed between two similarly sized 96-well aluminum blocks to assist in cooling the plate. Once cooled to room temperature, the top plate was removed and each vial was dosed with a stock solution of 1,3,5-trimethoxybenzene (10.49 mM in MeCN, 172 µL, 1.80 µmol). The vials were concentrated with a Genevac (vacuum centrifugation) with a 1 h cycle at 25 °C and the vials were diluted with MeCN (400 µL). For each vial, the resultant solution (212 µL) was transferred to a 96-well plastic well plate and further diluted with MeCN (588 µL). The plate was then centrifuged for 30 min to allow any precipitate to settle before analysis. This solution was analyzed using a Waters ACQUITY SQ.

UPLC (Acquity SQ): ACQUITY UPLC HSS C18; 1.8 µm; 150 mm x 2.1 mm; Column T = 40 °C; 0.350 mL/min. Gradient: 5.00 min at 54% MeCN/H_2O (0.1% FA); 2 min gradient to 58% MeCN/H_2O (0.1% FA); 1 min gradient to 95% MeCN/H_2O (0.1% FA); 0.85 min at 95% MeCN/H_2O (0.1% FA); 0.05 min to 5% MeCN/H_2O (0.1% FA); 0.1 min at 5% MeCN/H_2O (0.1% FA). Detection: PDA (TWC 190 – 260 nm); MS (ESI+, *m/z* = 320 – 380, 30 V, 20 Hz).

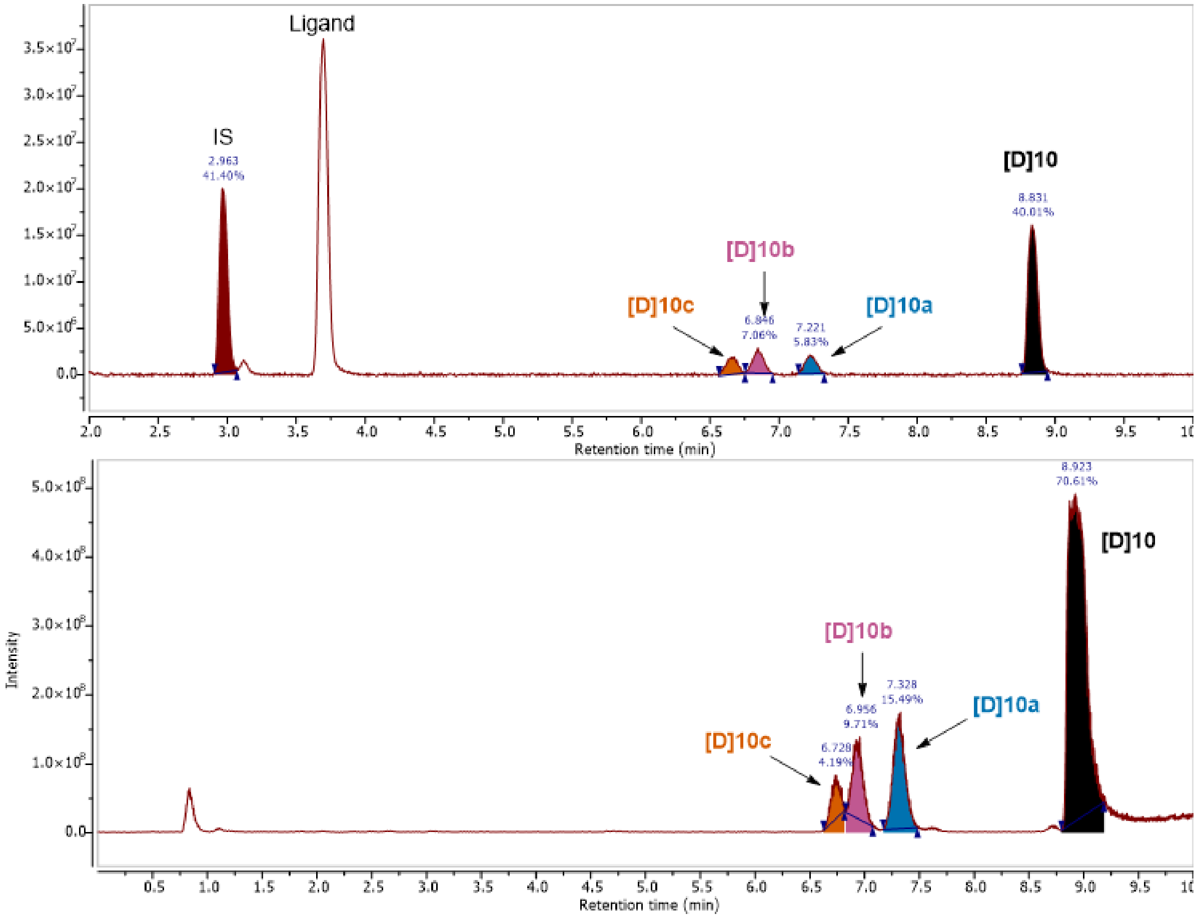

Fig. S164. Representative UPLC–MS trace for reaction A1-1.

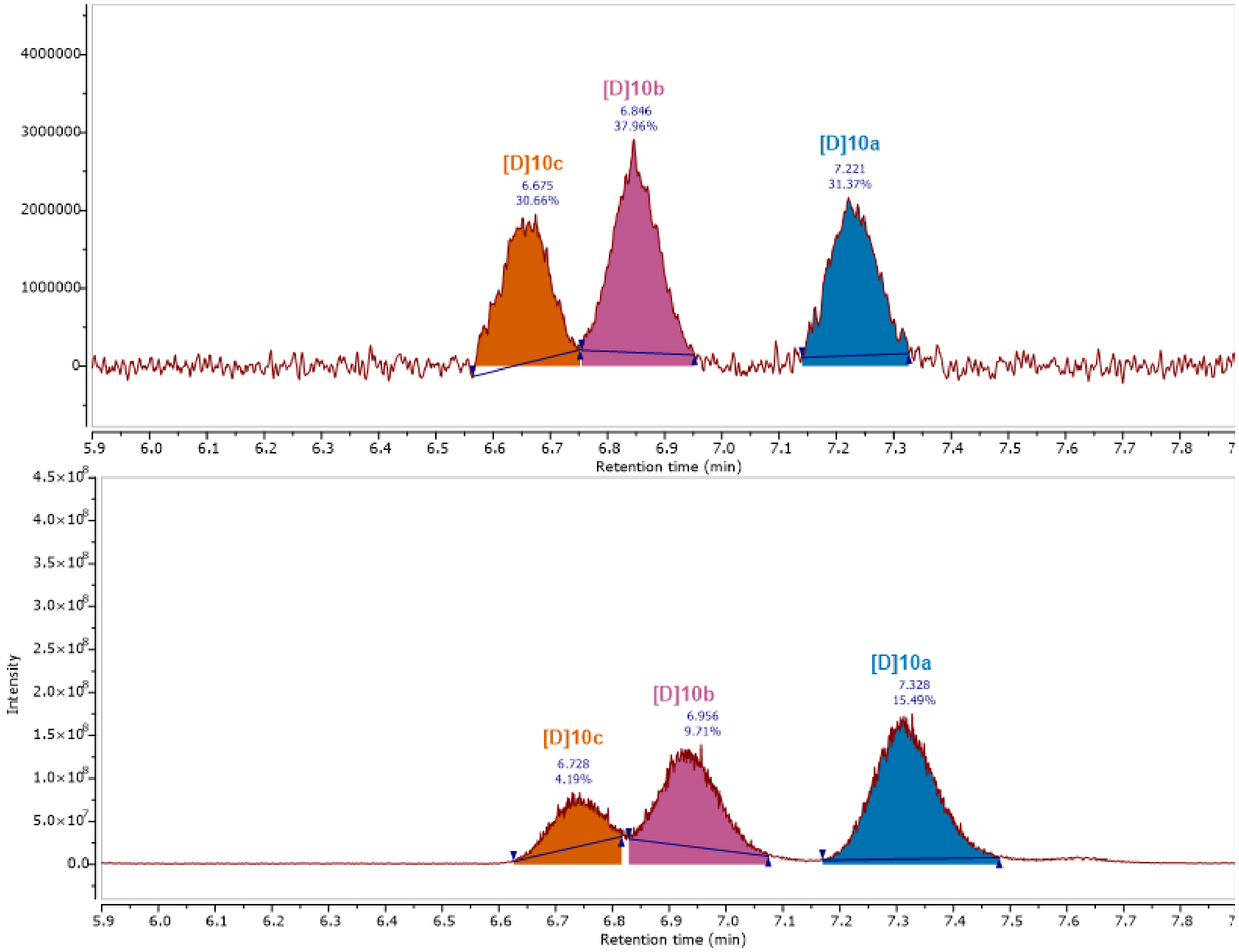

Fig. S165. Zoomed in portion of A1-1 depicting uncorrected product ratios.

Prod. Ratio	A1-C1[a]	A2-C2[a]	A3-C3[a]	A4-C4[a]	A5-C5[a]
[H/D]10a	33.9 ± 0.7	32.2 ± 0.3	30.8 ± 0.7	29.3 ± 0.3	27.8 ± 0.3
[H/D]10b	40.7 ± 0.6	41.5 ± 0.5	42.3 ± 0.7	42.6 ± 0.6	42.9 ± 0.4
[H/D]10c	25.4 ± 0.8	26.3 ± 0.5	26.9 ± 0.8	28.0 ± 0.6	29.3 ± 0.2

[a]Average of all six runs.

Table S22. Summary of the corrected product ratios across dilutions.

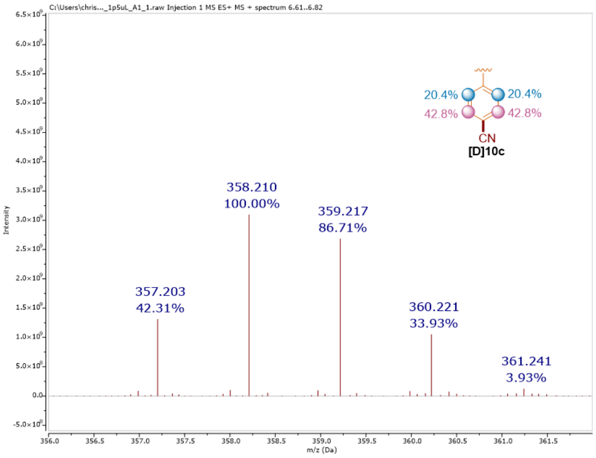

Fig. S166. Observed isotopic pattern for **[D]10c** in A1-1.

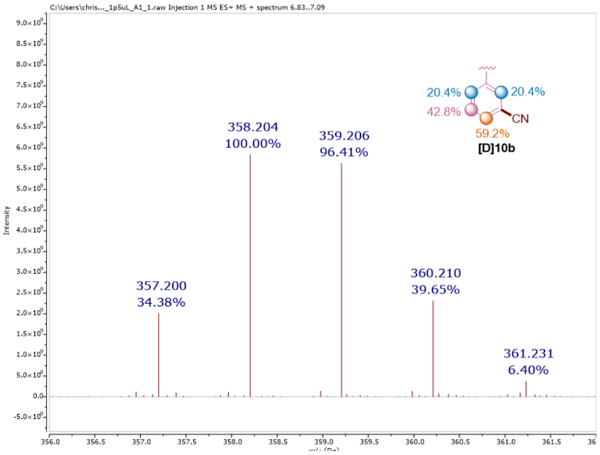

Fig. S167. Observed isotopic pattern for **[D]10b** in A1-1.

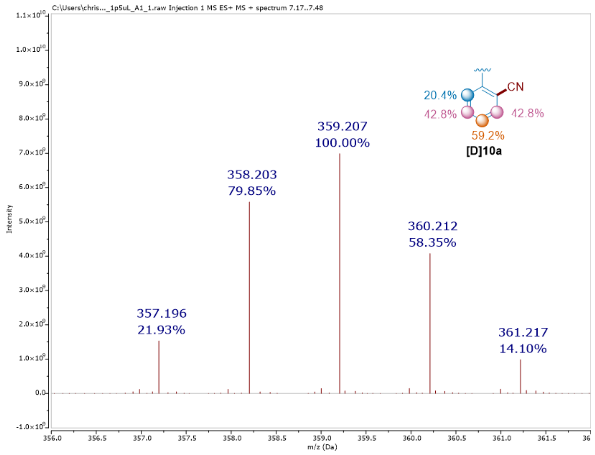

Fig. S168. Observed isotopic pattern for **[D]10a** in A1-1.

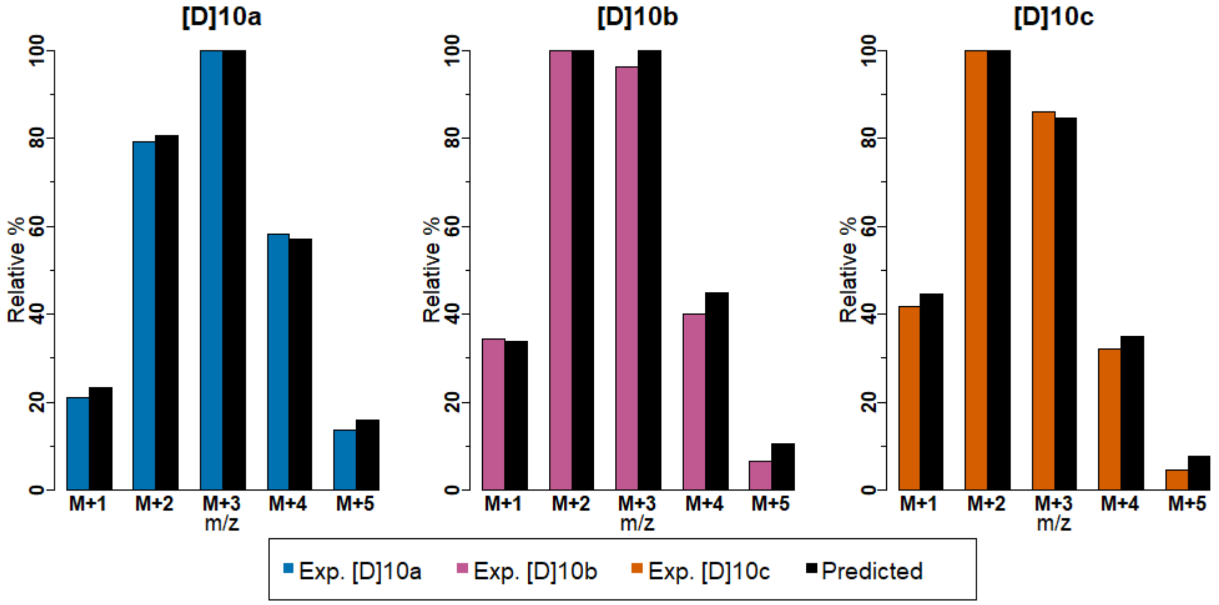

A1-C1	M+1[a]	M+2[a]	M+3[a]	M+4[a]	M+5[a]
Exp. [D]10a	20.9 ± 0.8	79.2± 0.5	100 ± 0	58.1 ± 0.8	4.4 ± 0.9
Pred. [D]10a	23.4	80.5	100	57.1	15.9
Exp. [D]10b	34.3 ± 0.5	100 ± 0	96.3 ± 0.9	40.0 ± 0.4	6.6 ± 0.3
Pred. [D]10b	33.8	99.8	100	44.9	10.6
Exp. [D]10c	41.7 ± 0.4	100 ± 0	86.0 ± 0.7	32.0 ± 1.4	4.4 ± 0.5
Pred. [D]10c	44.5	100	84.7	34.8	7.7

[a]Experimentally observed values represent the average relative abundance observed between wells A1-C1 over their duplicate trials (six total).

Fig. S169. Barplots depicted experimentally observed isotopic distribution (colored bars) and predicted isotopic distribution (black) (top). Table summarizing experimentally observed and predicted isotopic distribution (bottom).

10.2.3 MS-Only Analysis Nateglinide Methyl Ester

The three reactions performed using entirely deutero material (**[D]10**) were analyzed for MS-Only deconvolution of regioisomers. Due to the presence of a sodium adduct of the starting material overlapping with the products LOOP injections were not feasible. Instead, data from the separated mixture was used by taking the average across all three product peaks combined which should be analogous to a loop injection. This data is summarized below.

m/z	M+1	M+2	M+3	M+4	M+5	
**t1**	31.66	94.74	100	48.24	9.91	0.82
**t2**	31.18	95.89	100	48.74	10.09	0.69
**t3**	31.09	95.85	100	48.06	8.92	0.67
**Avg**	31.31	95.49	100	48.35	9.64	0.73
**SD**	0.25	0.53	0	0.29	0.51	0.07

	[D]10a	[D]10b	[D]10c
**OLS**	32.9 (0.108)	54.2 (0.151)	12.9 (0.451)
**Calib. UV**	33.9	40.7	25.4

Table S23. (top) Summary of M+1–M+6 values for triplicate loop injections. (bottom) OLS deconvoluted product ratios vs calibrated UV of deutero trial, for the OLS values p-values are shown in parentheses.

10.2.4 Derivations of KIE with Triply Deuterated Nateglinide Methyl Ester

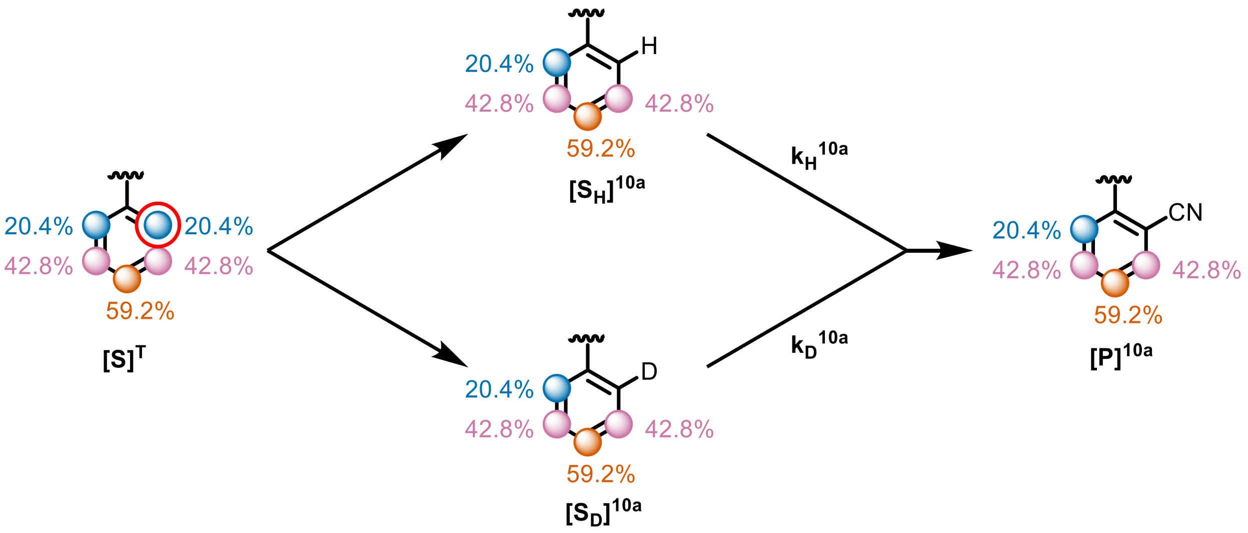

When focusing on one position, such as the *ortho* position, the starting material $[S]^T$ can be represented as two products, protio ($[S_H]^A$) and deutero ($[S_D]^A$), both leading to the formation of the same product $[P]^A$ at rates k_H^A and k_D^A respectively. As such the concentration of $[P]^A$ at time t is defined by equation (17).

$$[P]^{10a} = (k_H^{10a}[S_H]^{10a} + k_D^{10a}[S_D]^{10a})t \quad (17)$$

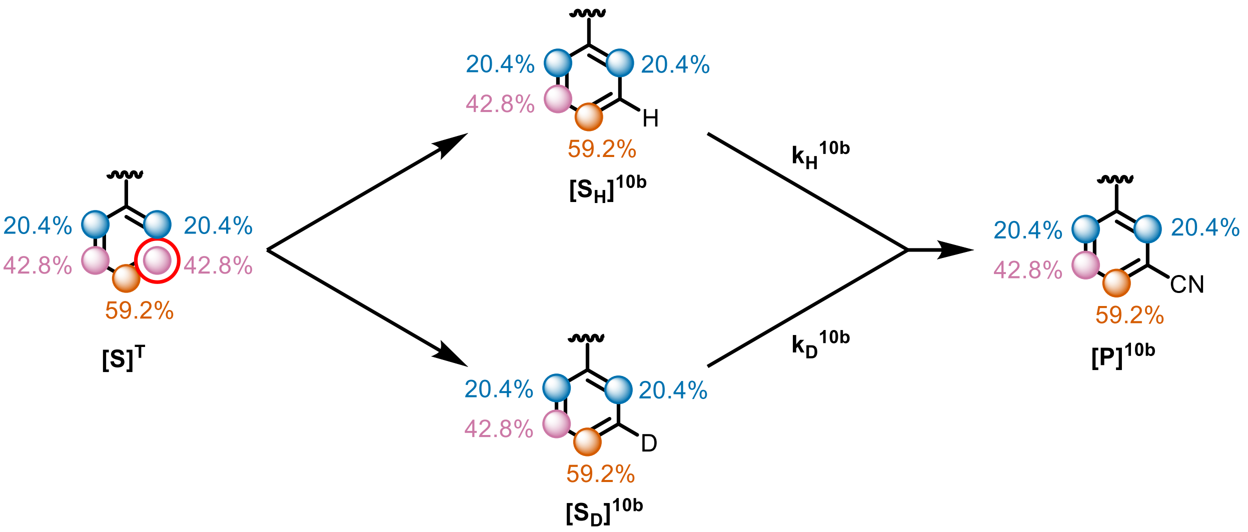

Performing a similar type of analysis on the *meta* position results in equation (18). When observing the ratio of *ortho* product over *meta* product across a series of dilutions stopped at the same time equation (19) results. Rearranging equation (19) yields equation (20). Performing the reaction *n* times at different ratios of protio and deutero material allows for the construction of the matrix shown below in equation (21).

$$[P]^{10b} = \left(k_H^{10b}[S_H]^{10b} + k_D^{10b}[S_D]^{10b}\right)t \tag{18}$$

$$\frac{[P]^{10a}}{[P]^{10b}} = \frac{k_H^{10a}[S_H]^{10a} + k_D^{10a}[S_D]^{10a}}{k_H^{10b}[S_H]^{10b} + k_D^{10b}[S_D]^{10b}} \tag{19}$$

$$\frac{[P]^{10a}[S_H]^{10b}}{[P]^{10b}} = \frac{k_H^{10a}}{k_H^{10b}}[S_H]^{10a} + \frac{k_D^{10a}}{k_H^{10b}}[S_D]^{10a} - \frac{k_D^{10b}}{k_H^{10b}}\frac{[P]^{10a}[S_D]^{10b}}{[P]^{10b}} \tag{20}$$

$$\begin{bmatrix} ([S_H]^{10a})^1 & ([S_D]^{10a})^1 & \left(-\frac{[P]^{10a}[S_D]^{10b}}{[P]^{10b}}\right)^1 \\ \vdots & \vdots & \vdots \\ ([S_H]^{10a})^n & ([S_D]^{10a})^n & \left(-\frac{[P]^{10a}[S_D]^{10b}}{[P]^{10b}}\right)^n \end{bmatrix} \begin{bmatrix} \frac{k_H^{10a}}{k_H^{10b}} \\ \frac{k_D^{10a}}{k_H^{10b}} \\ \frac{k_D^{10b}}{k_H^{10b}} \end{bmatrix} = \begin{bmatrix} \left(\frac{[P]^{10a}[S_H]^{10b}}{[P]^{10b}}\right)^1 \\ \vdots \\ \left(\frac{[P]^{10a}[S_H]^{10b}}{[P]^{10b}}\right)^n \end{bmatrix} \tag{21}$$

Equation (20) can be solved using the solver function in Excel as discussed previously, while equation (21) can be solved using OLS or NNLS linear regression.

The inverse of the value obtained for $\frac{k_D^{10b}}{k_H^{10b}}$ is the KIE for 10b and by dividing the value obtained for $\frac{k_H^{10a}}{k_H^{10b}}$ by the value of $\frac{k_D^{10a}}{k_H^{10b}}$ gives the KIE for 10a.

A summary of relevant values is shown below for the *ortho*, *meta*, and *para* products are shown below. The concentrations of products are substituted for their relative product ratio.

[H/D]10a Dilution (D:H)	$[P]^{10a}$	$[S_H]^{10a}$	$[S_D]^{10a}$
**100:0**	33.93	47.44	12.16
**75:25**	32.24	50.48	9.12
**50:50**	30.82	53.52	6.08
**25:75**	29.34	56.56	3.04

Table S24. Summary of relevant values for **[H/D]10a**.

[H/D]10b Dilution (D:H)	$[P]^{10b}$	$[S_H]^{10b}$	$[S_D]^{10b}$
**100:0**	40.67	34.09	25.51
**75:25**	41.50	40.47	19.13
**50:50**	42.32	46.85	12.75
**25:75**	42.63	53.22	6.38

Table S25. Summary of relevant values for **[H/D]10b**.

[H/D]10c Dilution (D:H)	$[P]^{10c}$	$[S_H]^{10c}$	$[S_D]^{10c}$
**100:0**	25.40	12.16	17.64
**75:25**	26.27	16.57	13.23
**50:50**	26.86	20.98	8.82
**25:75**	28.03	25.39	4.41

Table S26. Summary of relevant values for **[H/D]10c**.

OLS solution:

To solve using OLS analysis the matrix shown in equation (20) was used with the ratio of **[H/D]10a/[H/D]10b** to solve for the KIE at the *ortho* and *meta* position. The ratio of **[H/D]10c/[H/D]10b** was used to solve for the KIE of the *para* position. A summary of the obtained KIE values compared to those from conventional intermolecular KIE experiments are shown below.

	**10a (*ortho*)**	**10b (*meta*)**	**10c (*para*)**
**KIE (OLS)**	1.82 (0.062)	3.06 (0.075)	2.36 (0.459)
**KIE (intermolecular)**	2.04	3.48	2.54

Table S27. Summary of KIE values obtained through OLS analysis of dilution experiments (KIE(OLS)) and through traditional intermolecular experiments (KIE(intermolecular)). For KIE (OLS), p-values from the regression are shown in parentheses.

Excel Solver Solution:

Alternatively, the Excel solver function, as previously discussed, can be used on equation (19) over a series of *n* dilutions to solve for KIE values. In this instance, the product ratio of **[H/D]10a/[H/D]10b** was used to solve for the KIE of **10a** and **10b**, and the product ratio of

[H/D]10a/[H/D]10c was used to solve for the KIE of **10c**. A summary of the KIE values obtained compared to those from conventional intermolecular experiments are shown below.

	**10a (*ortho*)**	**10b (*meta*)**	**10c (*para*)**
**KIE (solver)**	2.18	3.39	1.95
**KIE (intermolecular)**	2.04	3.48	2.54

Table S28. Summary of KIE values obtained through the excel solver (KIE(solver)) and through traditional intermolecular experiments (KIE(intermolecular)).

10.3 Effect of Conversion on Regioselectivity

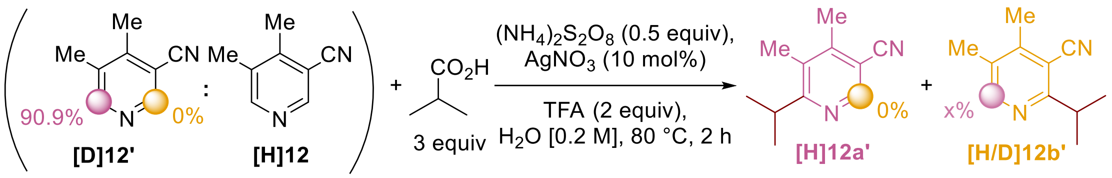

Two experiments were performed using ratios of 100:0 and 75:25 **[D]12'**:**[H]12** with the goal of analyzing outcomes at higher conversion. Experiments were performed in 5 mL scintillation vials under air. Stock solutions were prepared of **[D]12'** in (0.50 M, in $CHCl_3$) and **[H]12** (0.50 M, in $CHCl_3$). Following the table below an appropriate amount of each stock solution was added to each reaction vial to obtain 0.100 mmol of combined starting materials in the appropriate ratio.

**Ratio [D]12':[H]12**	**µL [D]12'**	**µL [H]12**
100:0	200	0
75:25	150	50

Following the addition of substrates, each reaction vial was concentrated to remove the $CHCl_3$ before adding water (0.3 mL), isobutyric acid (27.2 µL, 0.300 mmol), TFA (15.4 µL, 0.200 mmol), $AgNO_3$ (0.1 M in H_2O, 100 µL, 0.01 mmol, 10 mol%), and $(NH_4)_2S_2O_8$ (0.5 M in H_2O, 100 µL, 0.05 mmol) to obtain a solution with a final volume of 0.5 mL H_2O. After the addition of $(NH_4)_2S_2O_8$ the vial was capped and heated to 80 °C on an aluminum heating block. After 2 h, the vials were removed from the heating block and allowed to cool to room temperature. Once cooled to rt, 1,2,4,5-tetramethylbenzene (1.0 M in MeCN, 50 µL, 0.050 mmol) was added to each vial as an internal standard. A 1 mM sample was prepared, with respect to the starting concentration of starting material, by taking 5.5 µL of the solution and diluting with 994.5 µL of MeCN. These samples were analyzed using Waters ACQUITY TQ. Relative UV correction factors were used to compare molar ratios of **[H]12a** and **[H]12b** and are 1.33 and 1.00 respectively.

UPLC (Acquity TQ): Aquity UPLC HSS C18; 1.8 µm; 50 mm x 2.1 mm; Column T = 35 °C; 0.500 mL/min. Gradient: 0.50 min at 5% MeCN/H_2O (0.1% FA); 0.50 min gradient to 50% MeCN/H_2O (0.1% FA); 1.60 min at 50% MeCN/H_2O (0.1% FA); 0.10 min to 70% MeCN/H_2O (0.1% FA); 0.55 min to 90% MeCN/H_2O (0.1% FA); 1 min to 95% MeCN/H_2O (0.1% FA). Detection: PDA (TWC 215 – 254 nm); MS (ESI+, m/z = 100 – 300, 30 V, 20 Hz).

Data from this experiment was compared to the corresponding dilutions performed in section **10.1.4** and a summary of product ratios (**12a/12b**) and conversions are shown below.

Ratio [D]12':[H]12	Conv (%)	12a/12b
100:0	15	1.25
100:0	33	1.13
75:25	14	1.47
75:25	22	1.18

Table S29. Summary of conversion and product ratio data for mixture of **[D]12'** and **[H]12**.

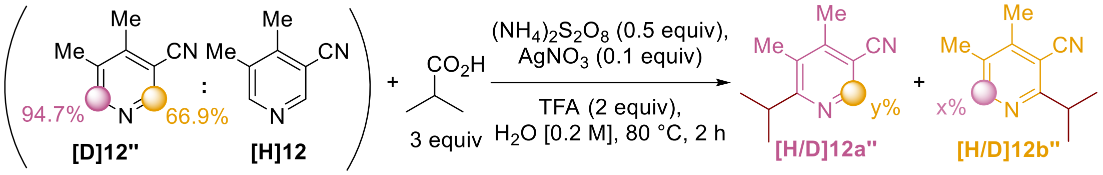

Two experiments were performed using ratios of 100:0 and 75:25 **[D]12":[H]12** with the goal of analyzing outcomes at higher conversion. Experiments were performed in 5 mL scintillation vials under air. Stock solutions were prepared of **[D]12"** in (0.50 M, in $CHCl_3$) and **[H]12** (0.50 M, in $CHCl_3$). Following the table below an appropriate amount of each stock solution was added to each reaction vial to obtain 0.100 mmol of combined starting materials in the appropriate ratio.

Ratio [D]12":[H]12	µL [D]12"	µL [H]12
100:0	200	0
75:25	150	50

Following the addition of substrates, each reaction vial was concentrated to remove the $CHCl_3$ before adding water (0.3 mL), isobutyric acid (27.2 µL, 0.300 mmol), TFA (15.4 µL, 0.200 mmol), $AgNO_3$ (0.1 M in H_2O, 100 µL, 0.01 mmol, 10 mol%), and $(NH_4)_2S_2O_8$ (0.5 M in H_2O, 100 µL, 0.05 mmol) to obtain a solution with a final volume of 0.5 mL H_2O. After the addition of $(NH_4)_2S_2O_8$ the vial was capped and heated to 80 °C on an aluminum heating block. After 2 h, the vials were removed from the heating block and allowed to cool to room temperature. Once cooled to rt, 1,2,4,5-tetramethylbenzene (1.0 M in MeCN, 50 µL, 0.050 mmol) was added to each vial as an internal standard. A 1 mM sample was prepared, with respect to the starting concentration of starting material, by taking 5.5 µL of the solution and diluting with 994.5 µL of MeCN. These samples were analyzed using Waters ACQUITY TQ. Relative UV correction factors were used to compare molar ratios of **[H]12a** and **[H]12b** and are 1.33 and 1.00 respectively.

UPLC (Acquity TQ): Aquity UPLC HSS C18; 1.8 µm; 50mm x 2.1mm; Column T = 35 °C; 0.500 mL/min. Gradient: 0.50 min at 5% MeCN/H_2O (0.1% FA); 0.50 min gradient to 50% MeCN/H_2O (0.1% FA); 1.60 min at 50% MeCN/H_2O (0.1% FA); 0.10 min to 70% MeCN/H_2O (0.1% FA); 0.55 min to 90% MeCN/H_2O (0.1% FA); 1 min to 95% MeCN/H_2O (0.1% FA). Detection: PDA (TWC 215 – 254 nm); MS (ESI+, *m/z* = 100 – 300, 30 V, 20 Hz).

Data from this experiment was compared to the corresponding dilutions performed in section **10.1.5** and a summary of product ratios (**12a/12b**) and conversions are shown below.

Ratio [D]12”:[H]12	Conv. (%)	12a/12b
100:0	16	1. 82
100:0	30	1. 26
75:25	19	1. 94
75:25	33	1. 56

Table S30. Summary of conversion and product ratio data for mixture of **[D]12”** and **[H]12**.

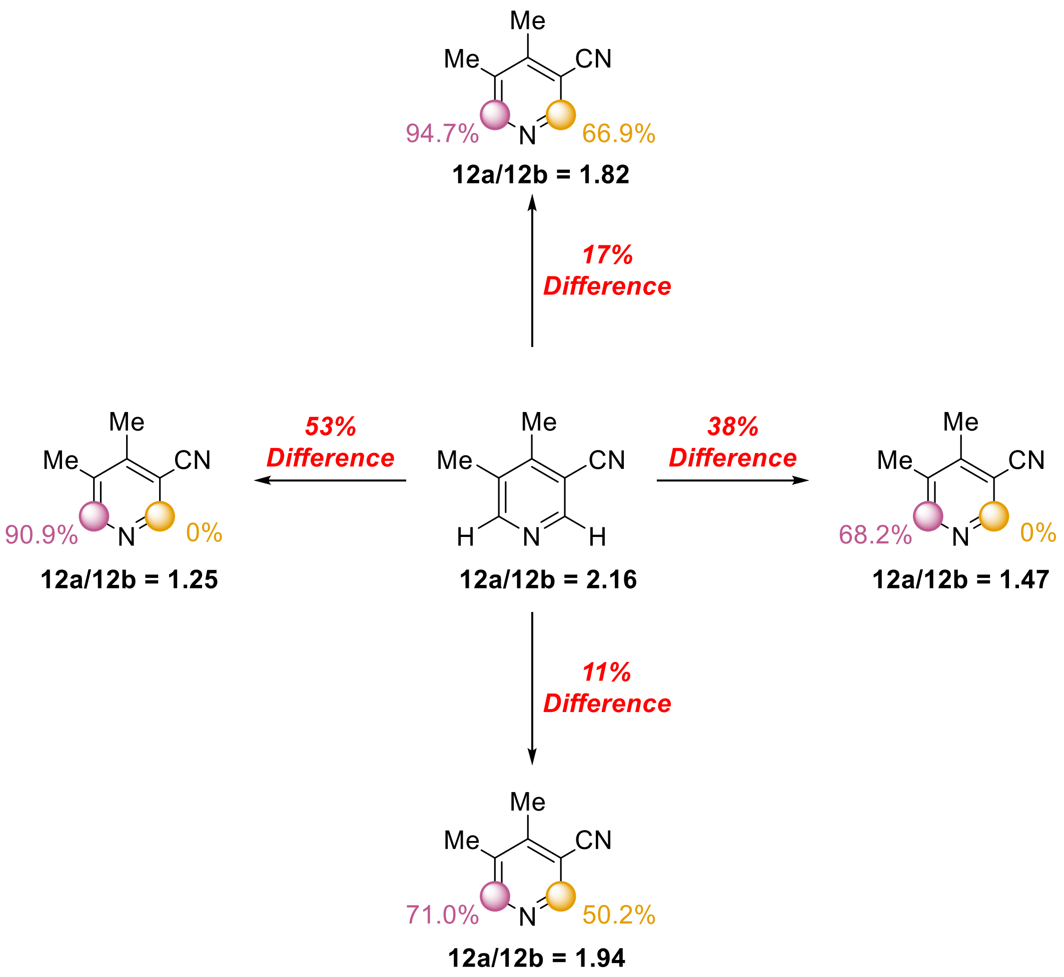

Fig. S170. Change in product ratios with respect to deuterium incorporation at low conversions.

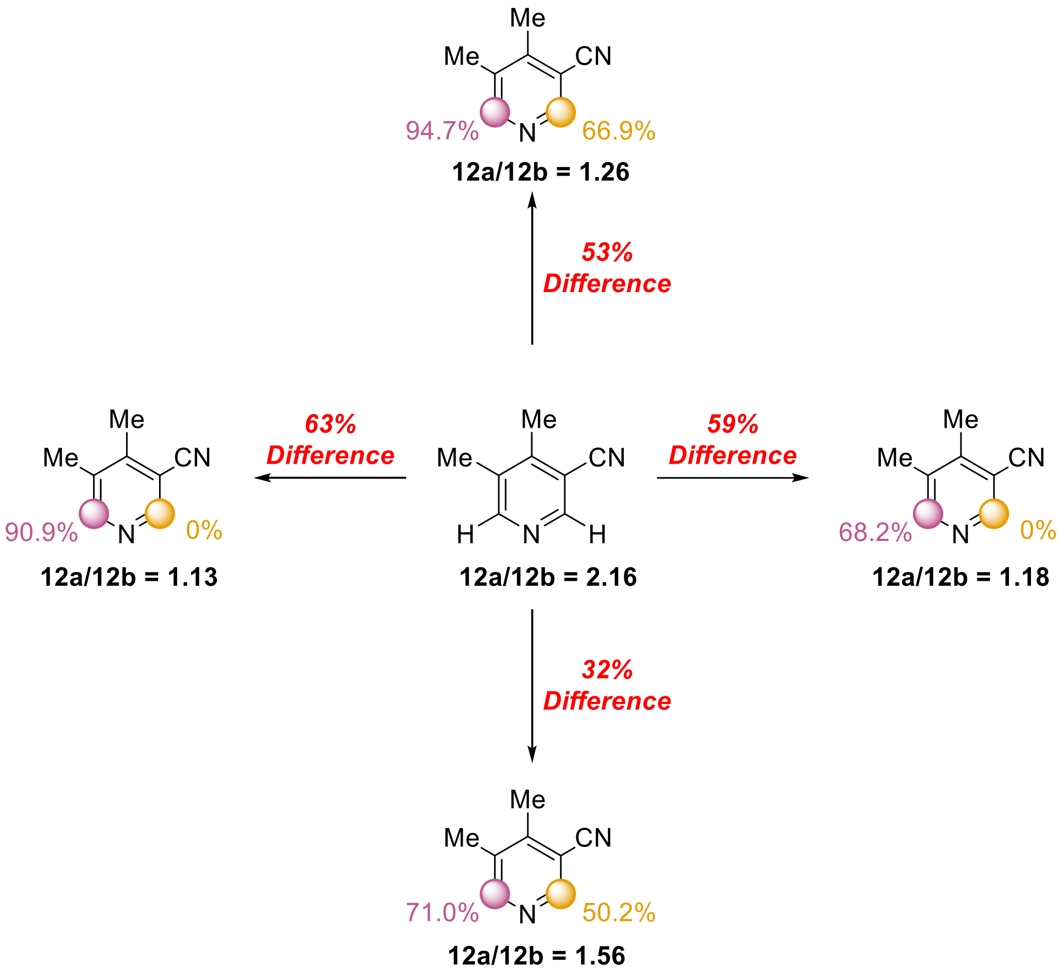

Fig. S171. Change in product ratios with respect to deuterium incorporation at high conversions.

From the above results we can observe that when a KIE is present greater differences in regioselectivity occur at higher conversions, and when there are larger differences in deuterium incorporation.

11. NMR Spectra

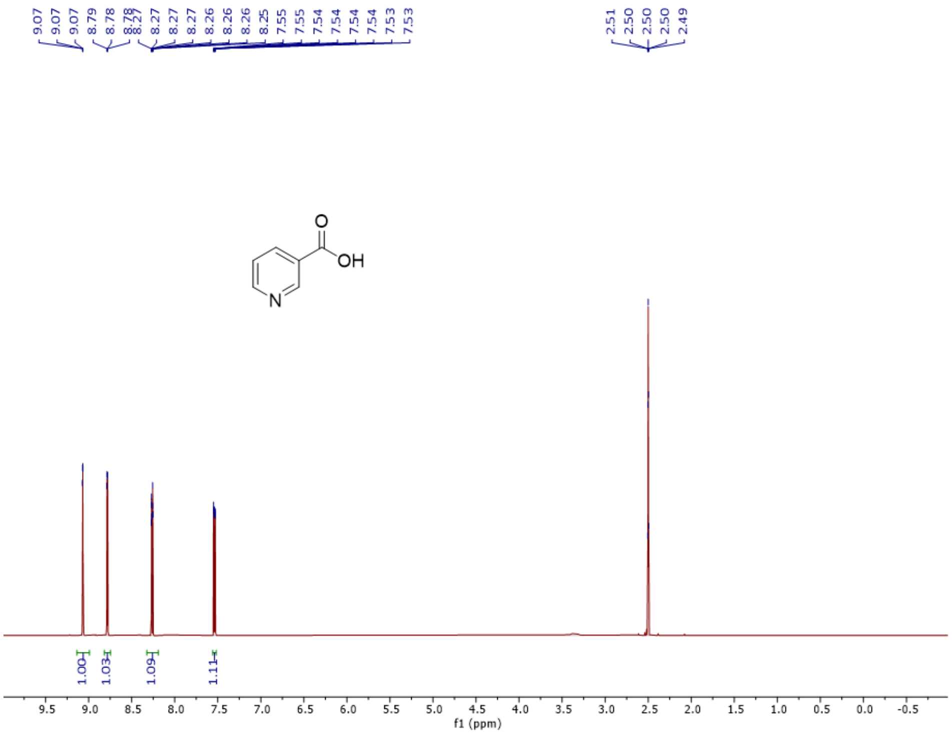

Fig. S172. ^{1}H NMR (600 MHz, DMSO-d6) of H-nicotinic acid ([H]1).

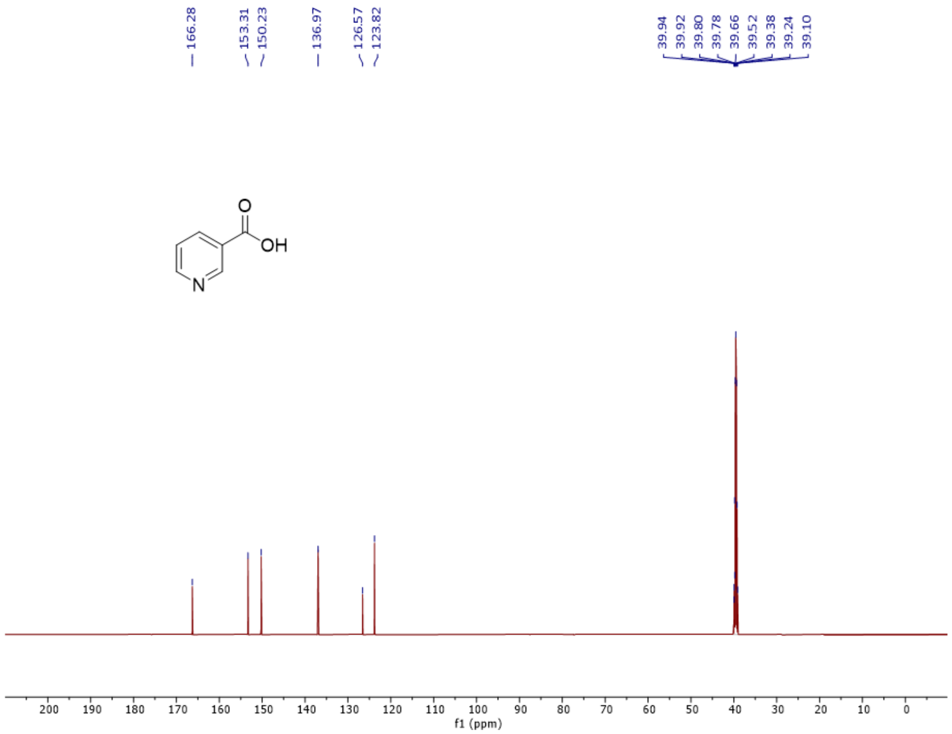

Fig. S173. ^{13}C{^{1}H} NMR (151 MHz, DMSO-d6) of H-nicotinic acid ([H]1).

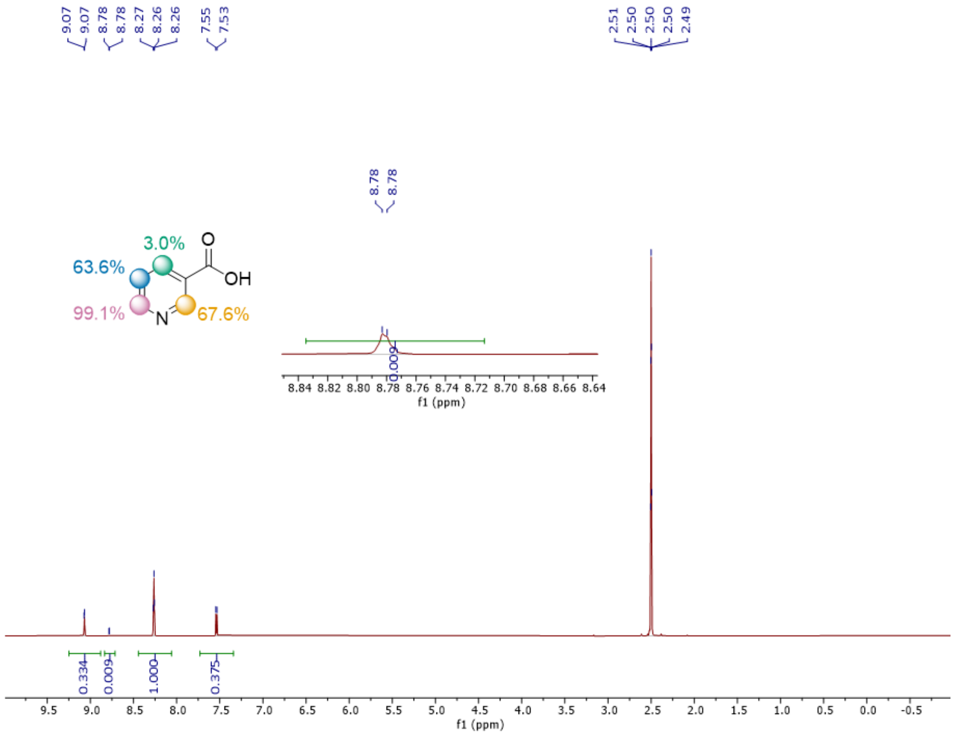

Fig. S174. ^{1}H **NMR (600 MHz, d1=30s, DMSO-d6) of D-nicotinic acid ([D]1).**

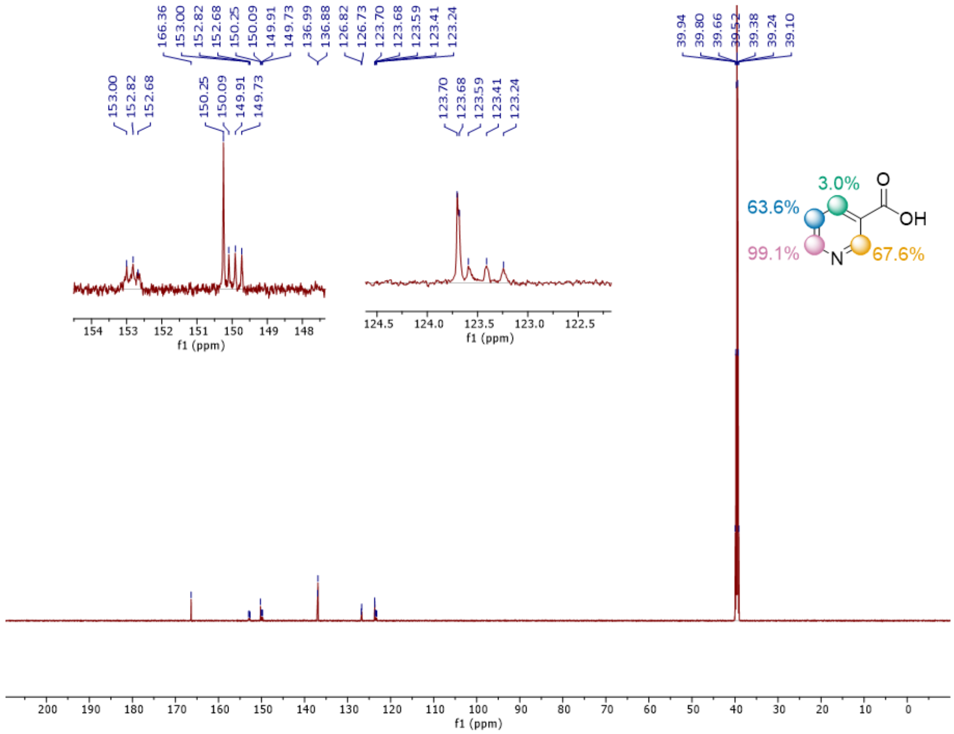

Fig. S175. $^{13}C\{^{1}H\}$ **NMR (151 MHz, DMSO-d6) of D-nicotinic acid ([D]1).**

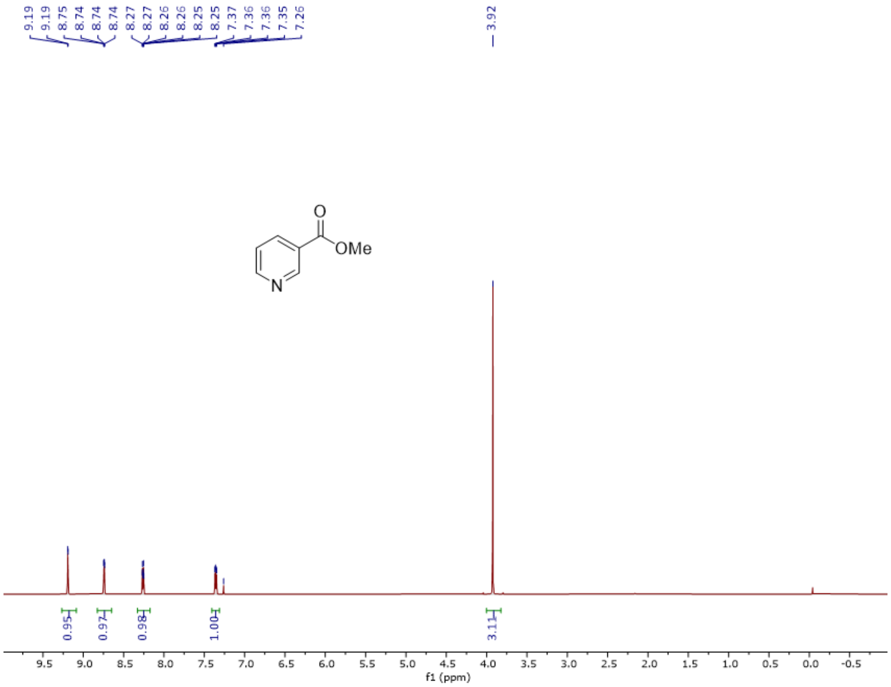

Fig. S176. **^{1}H NMR (600 MHz, $CDCl_3$) of H-methyl nicotinate ([H]2).**

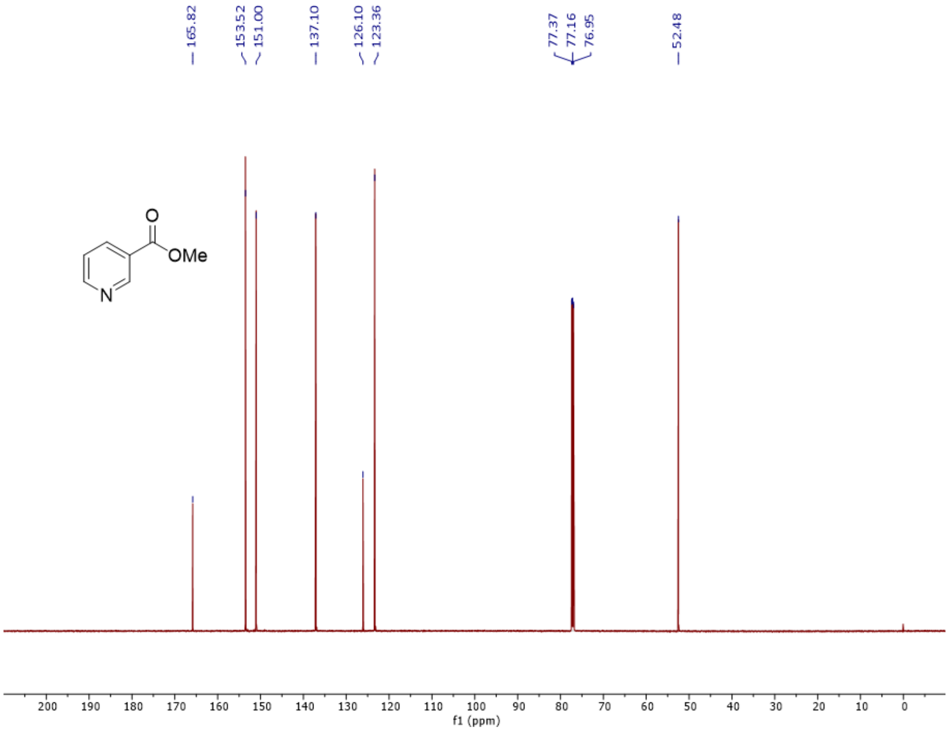

Fig. S177. **$^{13}C\{^1H\}$ NMR (151 MHz, $CDCl_3$) of H-methyl nicotinate ([H]2).**

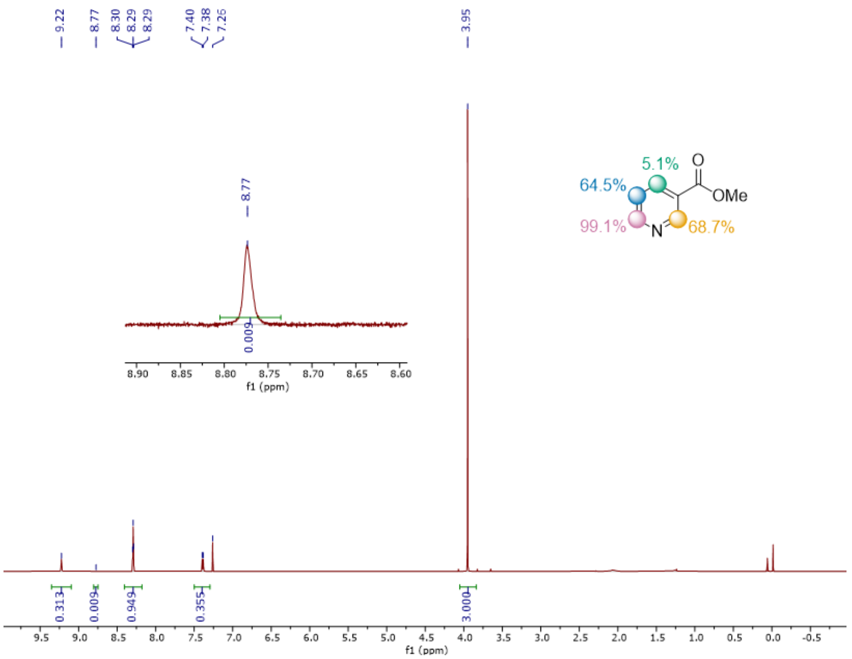

Fig. S178. ^{1}H NMR (600 MHz, d1=30s, $CDCl_3$) of D-methyl nicotinate ([D]2).

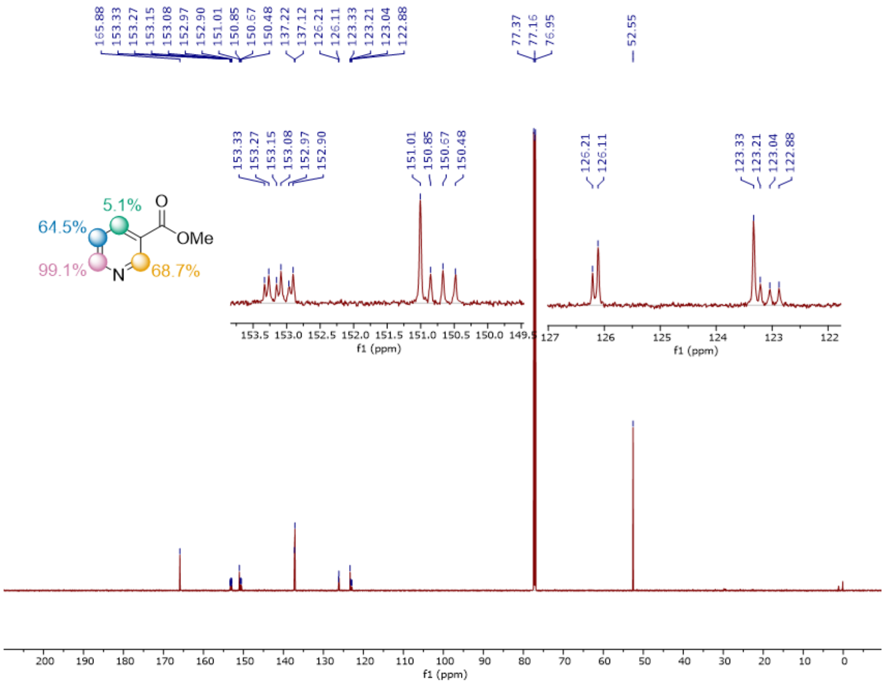

Fig. S179. $^{13}C\{^{1}H\}$ NMR (151 MHz, $CDCl_3$) of D-methyl nicotinate ([D]2).

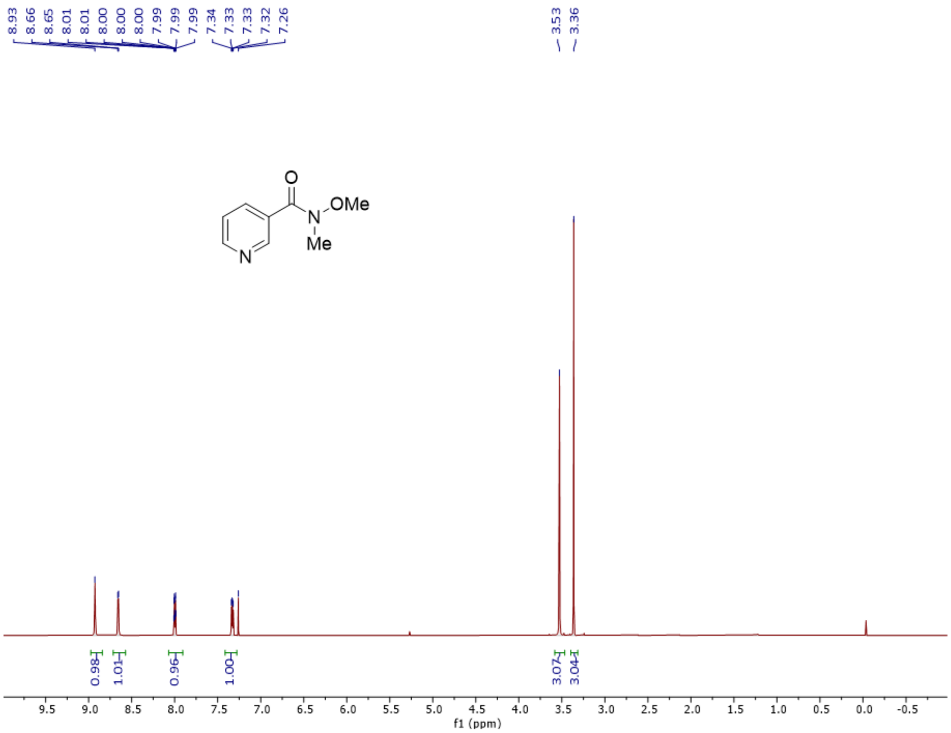

Fig. S180. ^{1}H NMR (600 MHz, $CDCl_3$) of H-*N*-Methoxy-*N*-methylnicotinamide.

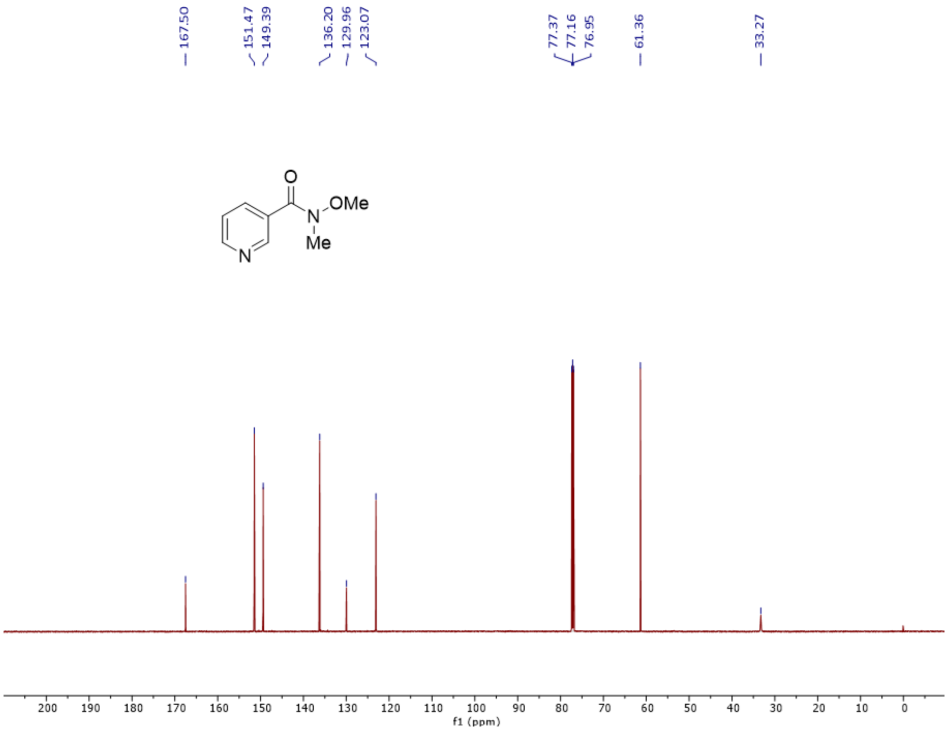

Fig. S181. $^{13}C\{^1H\}$ NMR (151 MHz, $CDCl_3$) of H-*N*-Methoxy-*N*-methylnicotinamide.

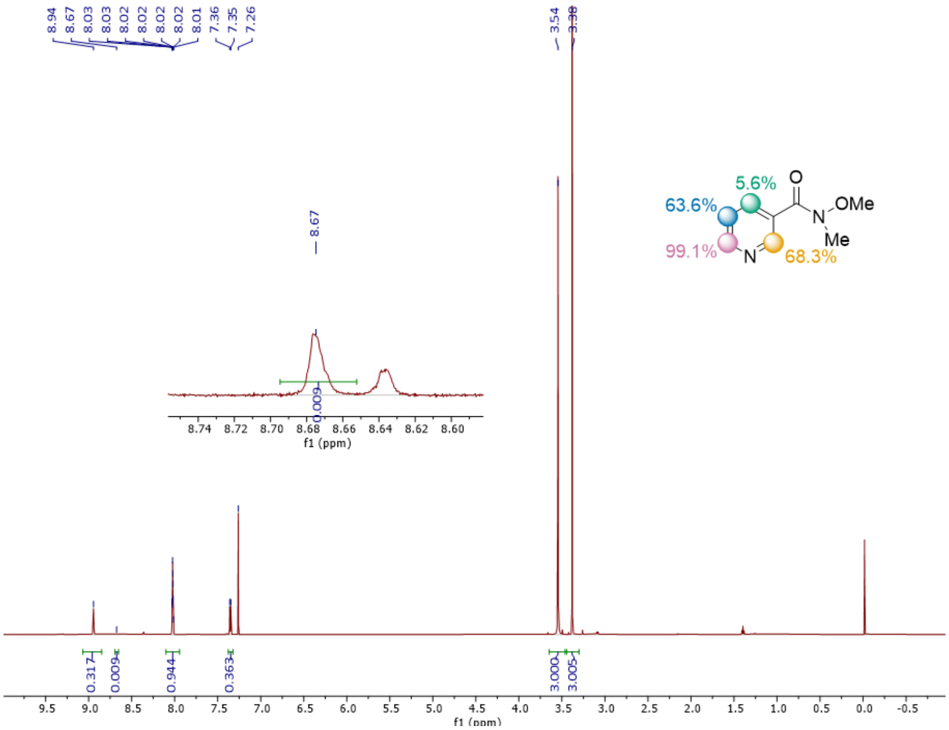

Fig. S182. ^{1}H **NMR (600 MHz, d1= 30s,** $CDCl_3$**) of D-*N*-Methoxy-*N*-methylnicotinamide.**

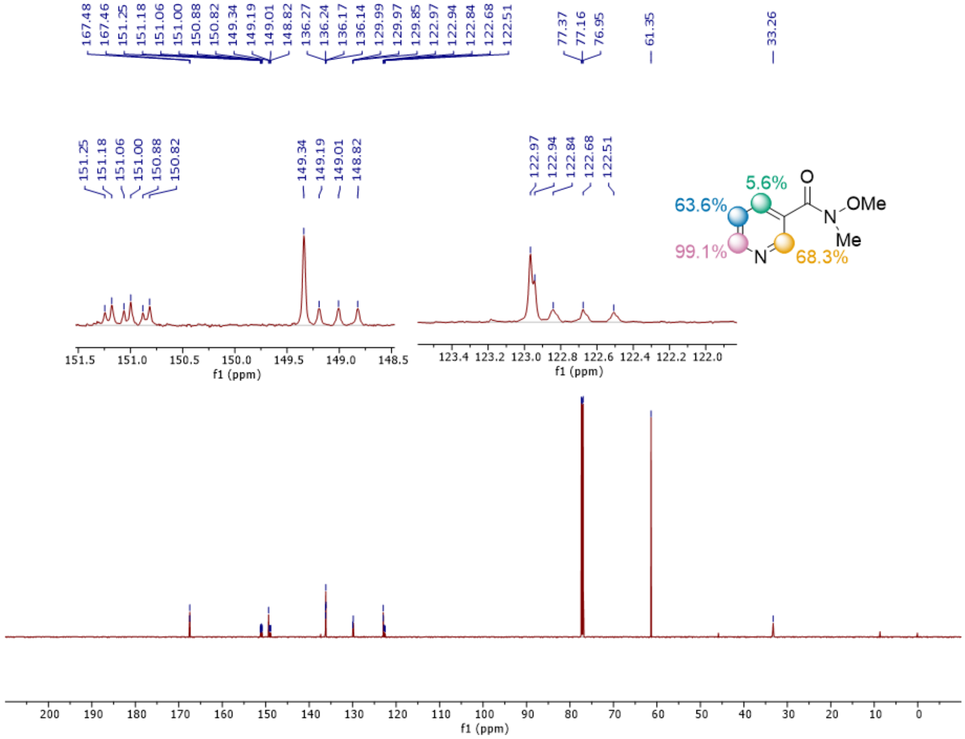

Fig. S183. $^{13}C\{^{1}H\}$ **NMR (151 MHz,** $CDCl_3$**) of D-*N*-Methoxy-*N*-methylnicotinamide.**

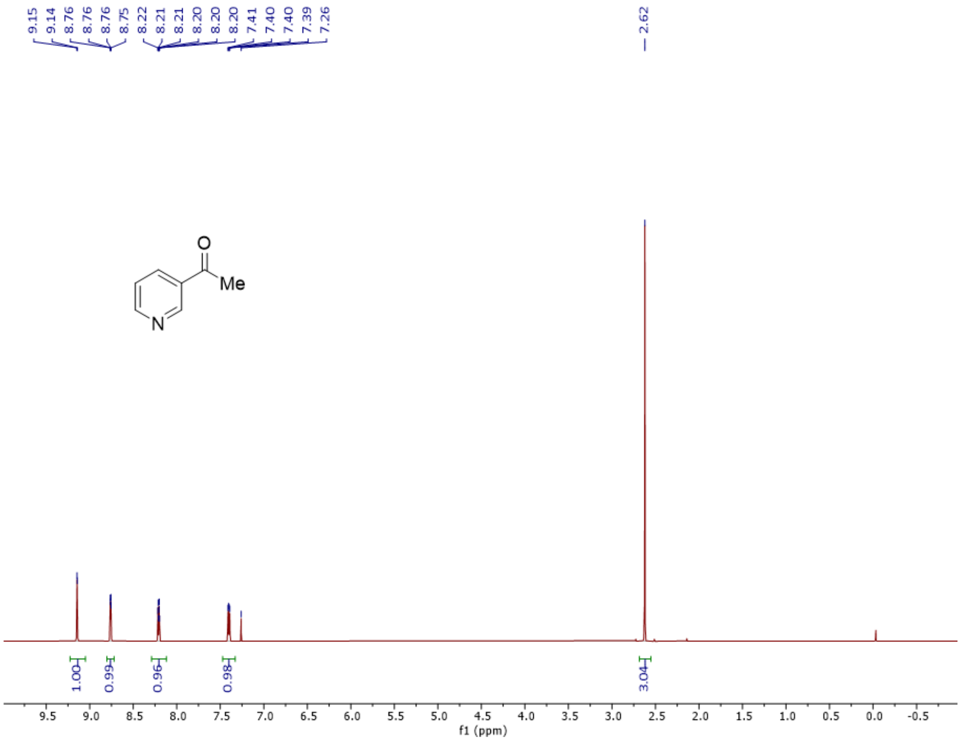

Fig. S184. **^{1}H NMR (600 MHz, $CDCl_3$) of H-3-acetylpyridine ([H]4).**

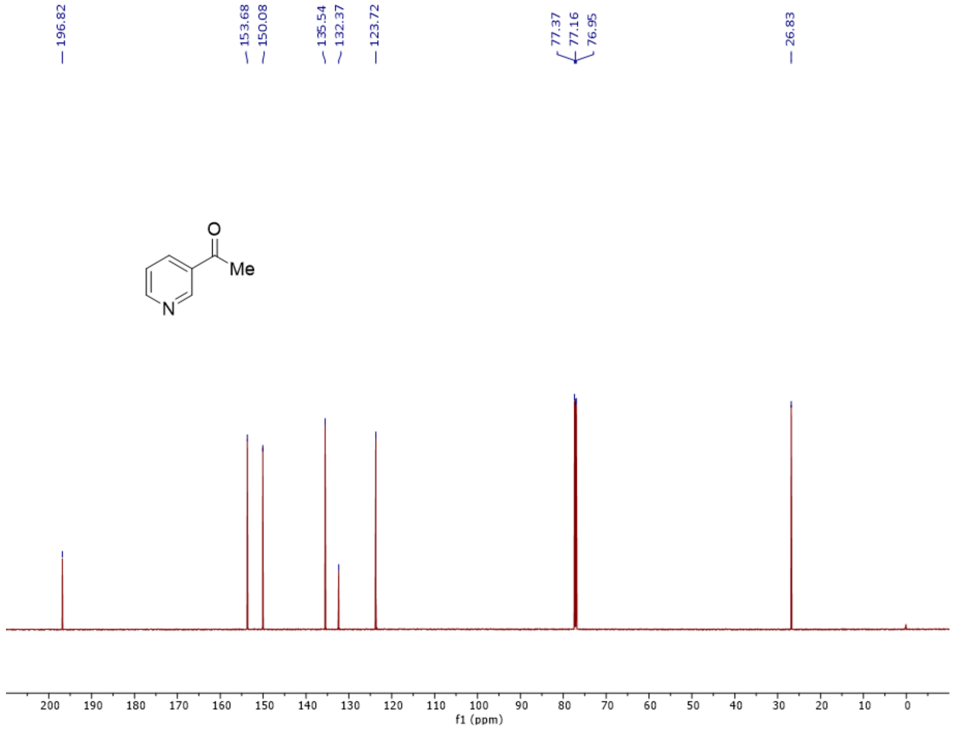

Fig. S185. **^{13}C{^{1}H} NMR (151 MHz, $CDCl_3$) of H-3-acetylpyridine ([H]4).**

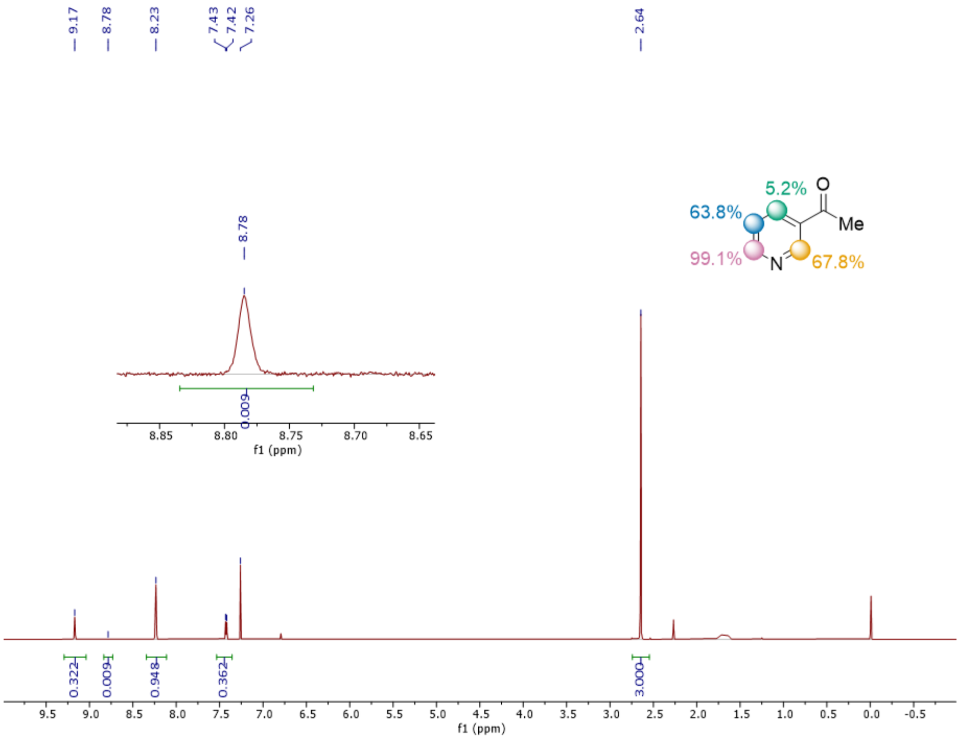

Fig. S186. ^{1}H NMR (600 MHz, d1=30s, $CDCl_3$) of D-3-acetylpyridine ([D]4).

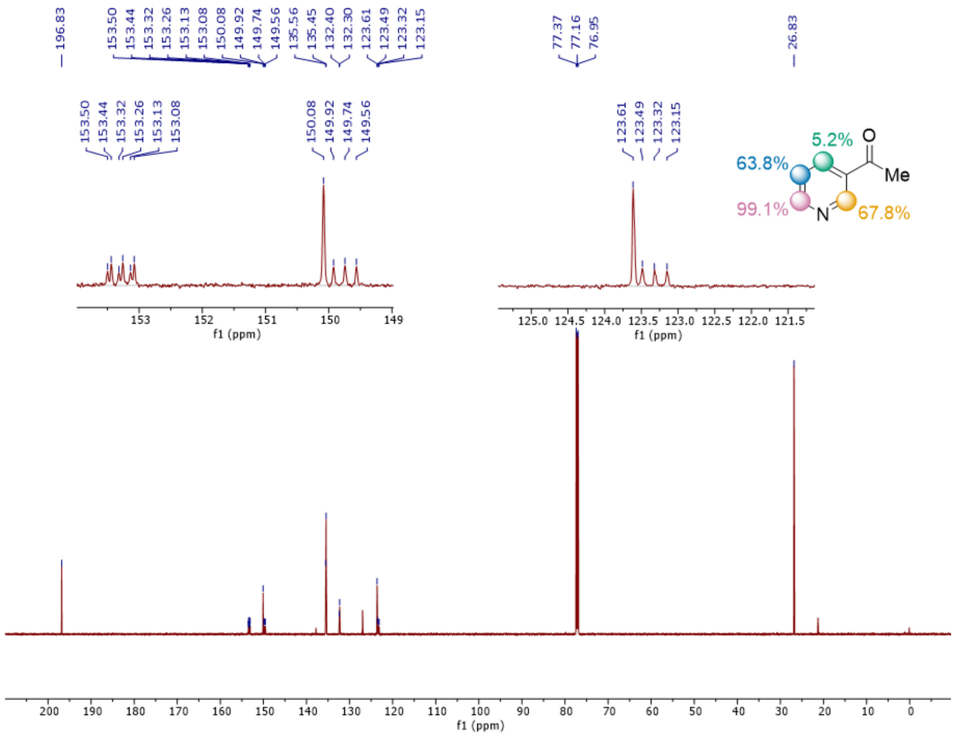

Fig. S187. $^{13}C\{^{1}H\}$ NMR (151 MHz, $CDCl_3$) of D-3-acetylpyridine ([D]4).

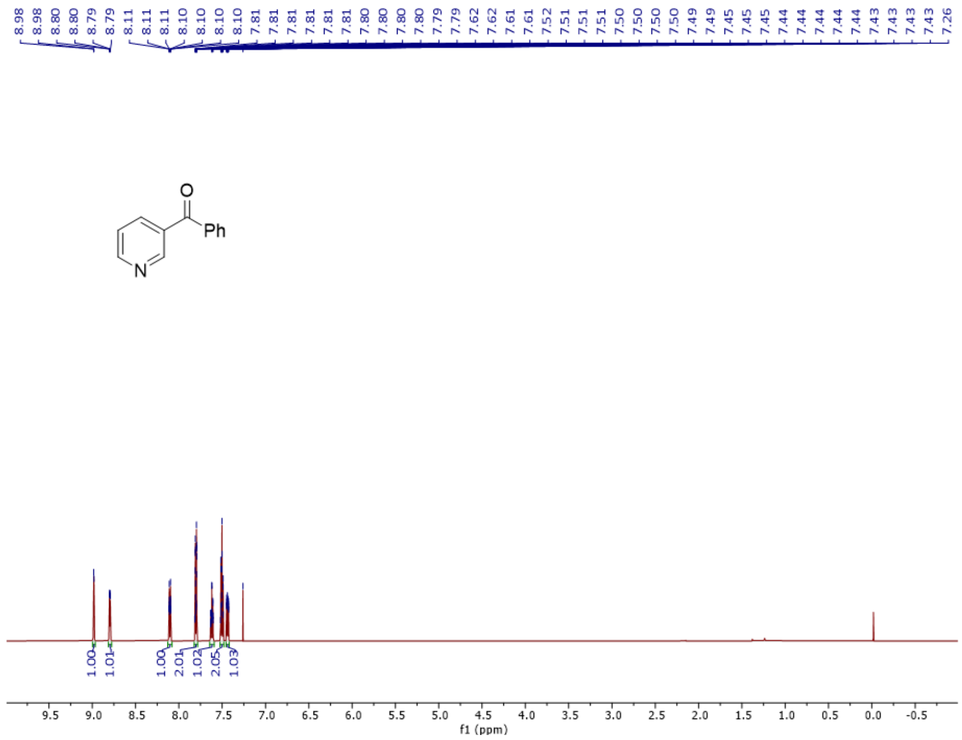

Fig. S188. ^{1}H **NMR (600 MHz,** $CDCl_3$**) of H-3-benzoylpyridine ([H]5).**

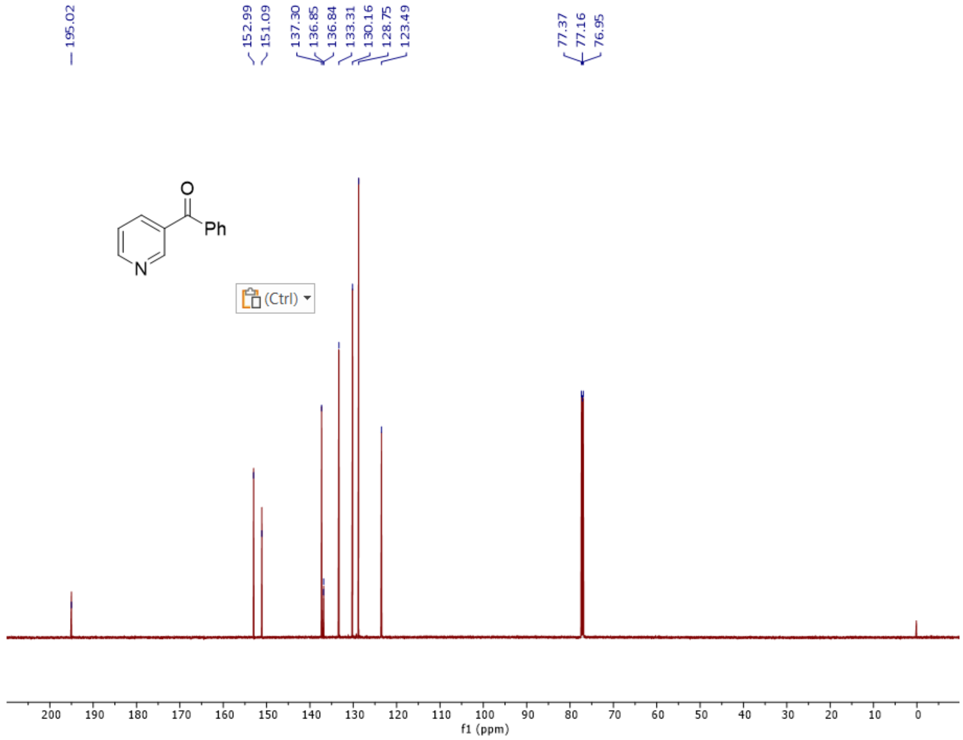

Fig. S189. $^{13}C\{^{1}H\}$ **NMR (151 MHz,** $CDCl_3$**) of H-3-benzoylpyridine ([H]5).**

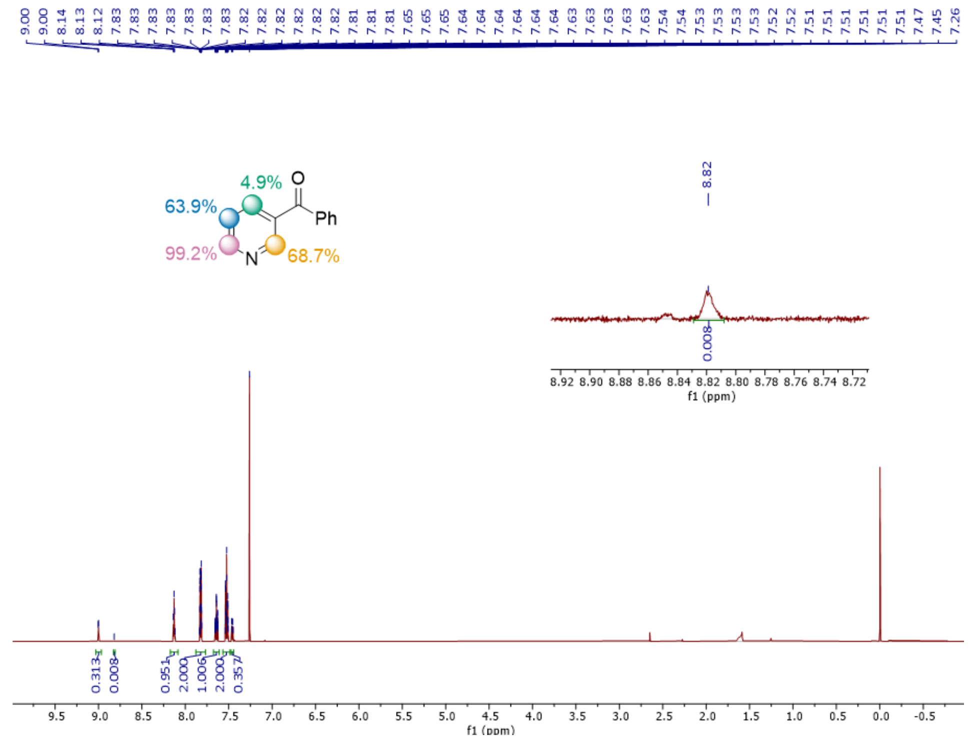

Fig. S190. [1]**H NMR (600 MHz, d1=30s, $CDCl_3$) of D-3-benzoylpyridine ([D]5).**

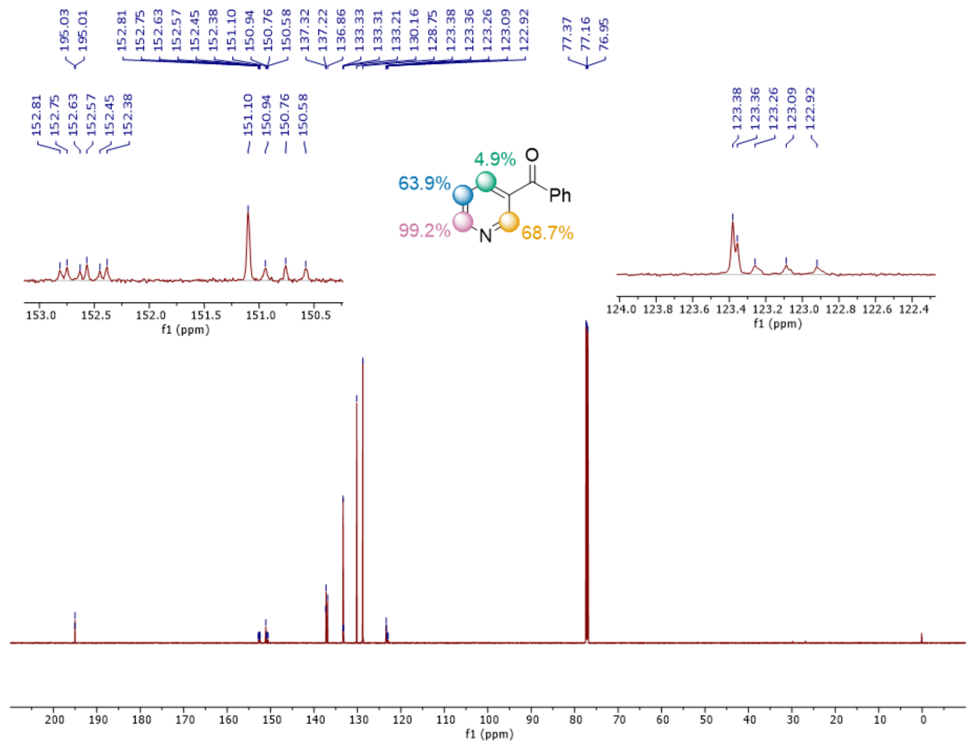

Fig. S191. ^{13}**C{**^{1}**H} NMR (151 MHz, $CDCl_3$) of D-3-benzoylpyridine ([D]5).**

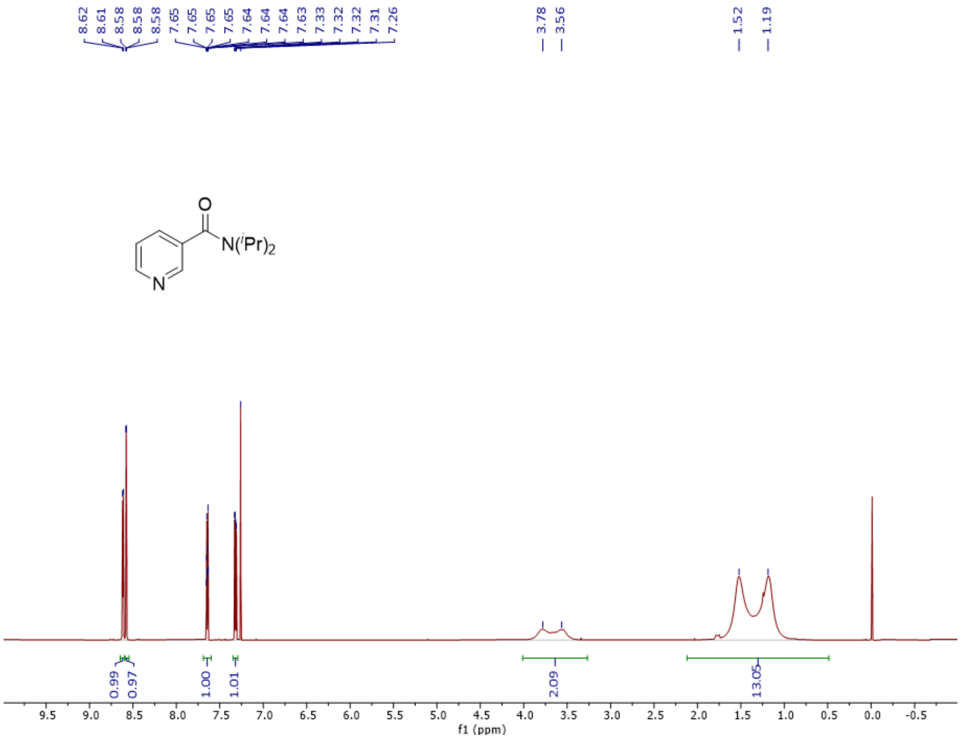

Fig. S192. ^{1}H **NMR (600 MHz,** $CDCl_3$**) of H-*N,N*-diisopropylnicotinamide ([H]7).**

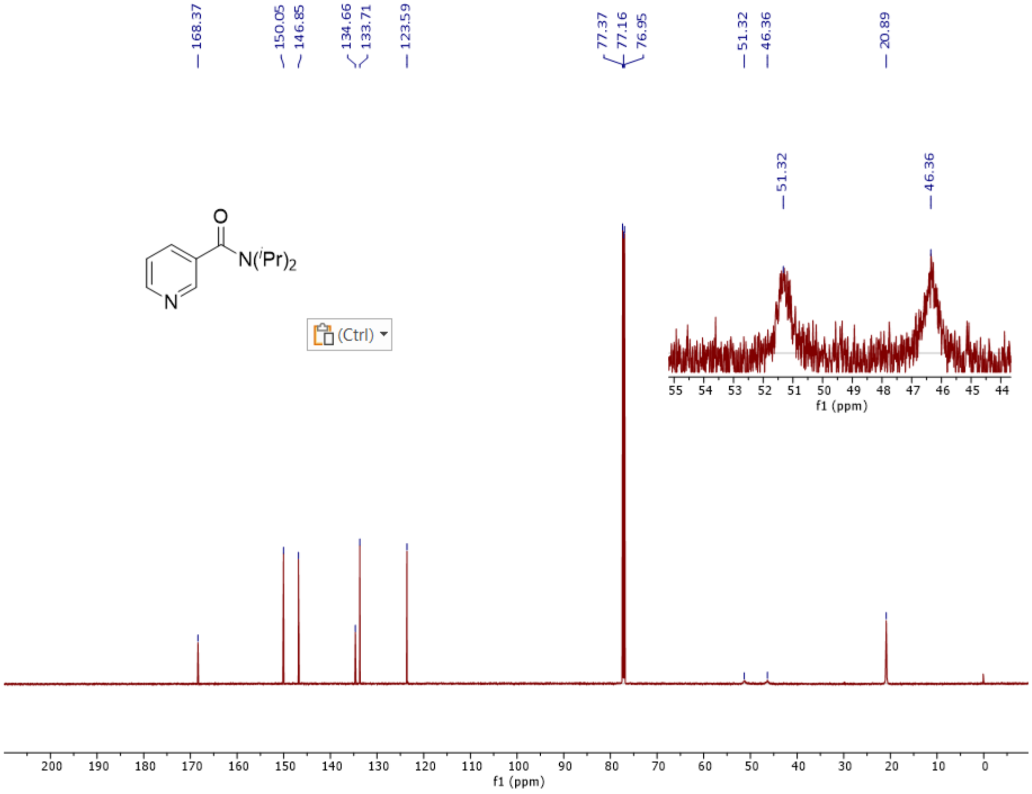

Fig. S193. $^{13}C\{^{1}H\}$ **NMR (151 MHz,** $CDCl_3$**) of H-*N,N*-diisopropylnicotinamide ([H]7).**

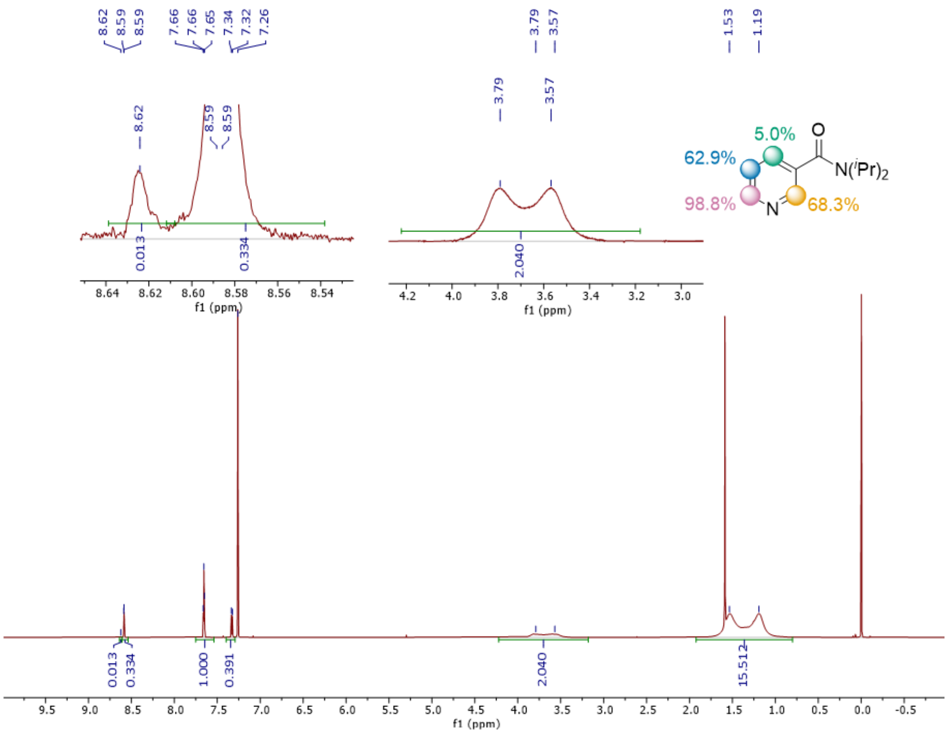

Fig. S194. ^{1}H NMR (600 MHz, d1=30s, $CDCl_3$) of D-*N,N*-diisopropylnicotinamide ([D]7).

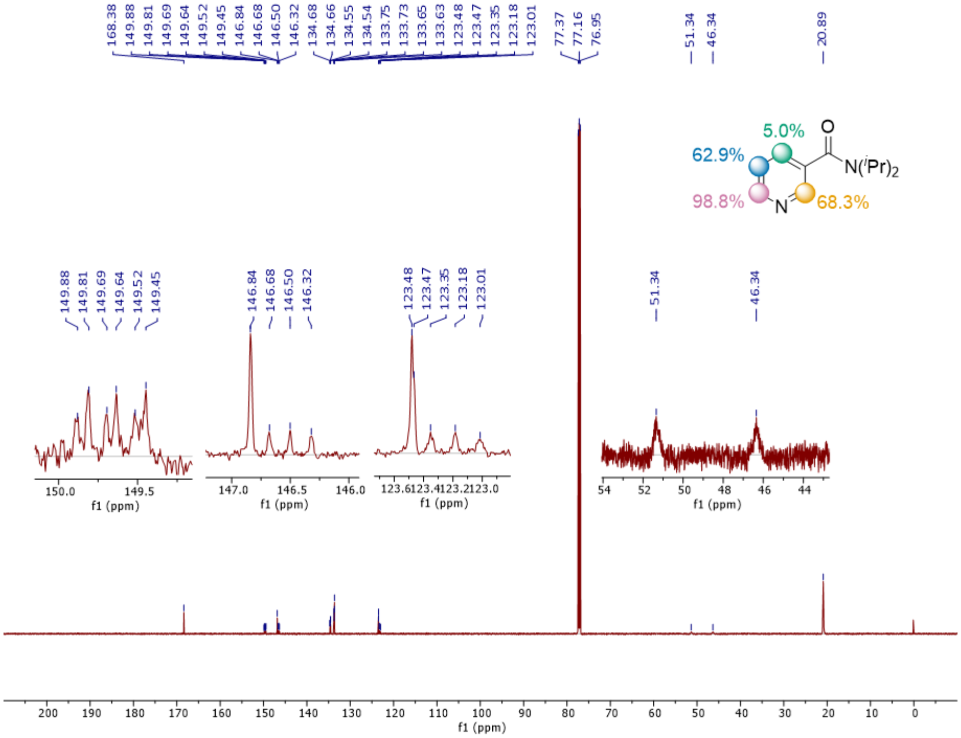

Fig. S195. $^{13}C\{^1H\}$ NMR (151 MHz, $CDCl_3$) of D-*N,N*-diisopropylnicotinamide ([D]7).

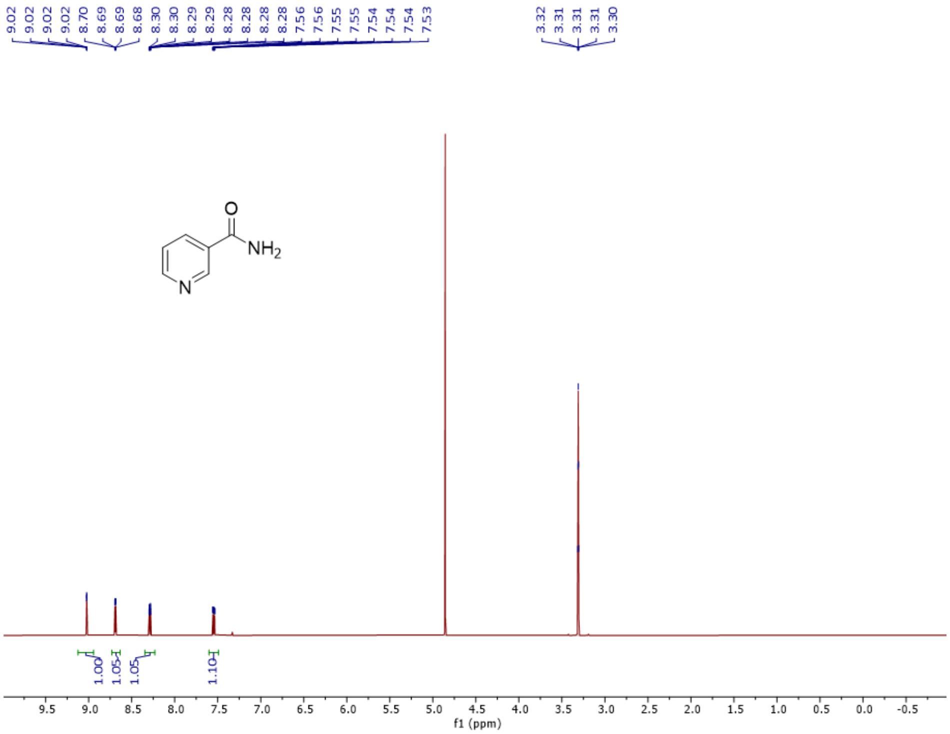

Fig. S196. ^{1}H **NMR (600 MHz, MeOD) of H-nicotinamide ([H]6).**

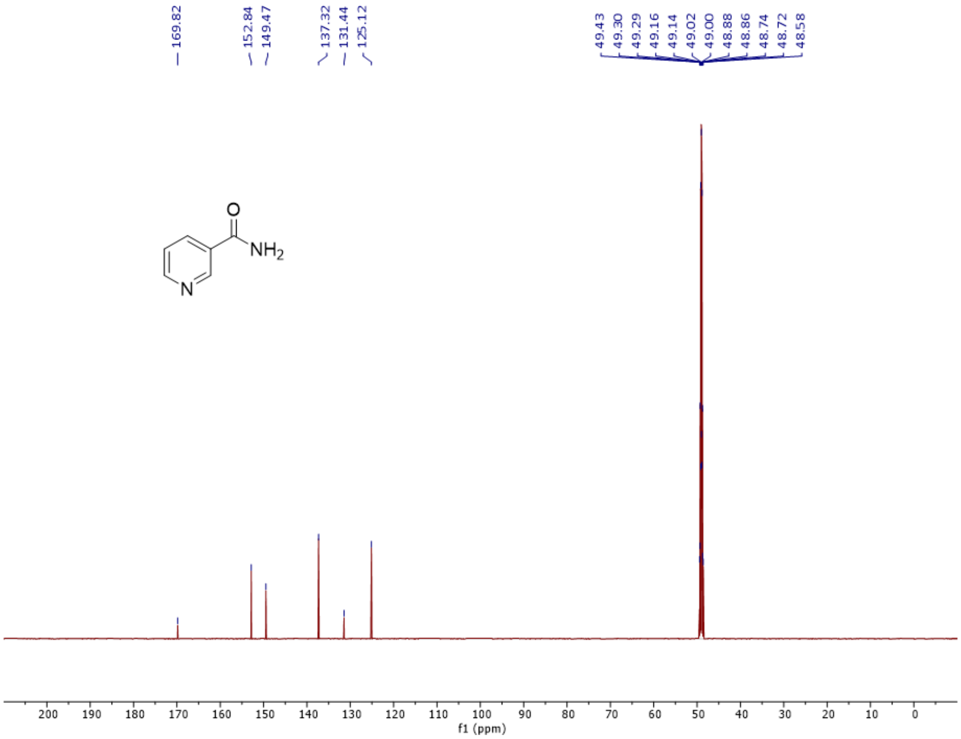

Fig. S197. $^{13}C\{^{1}H\}$ **NMR (151 MHz, MeOD) of H-nicotinamide ([H]6).**

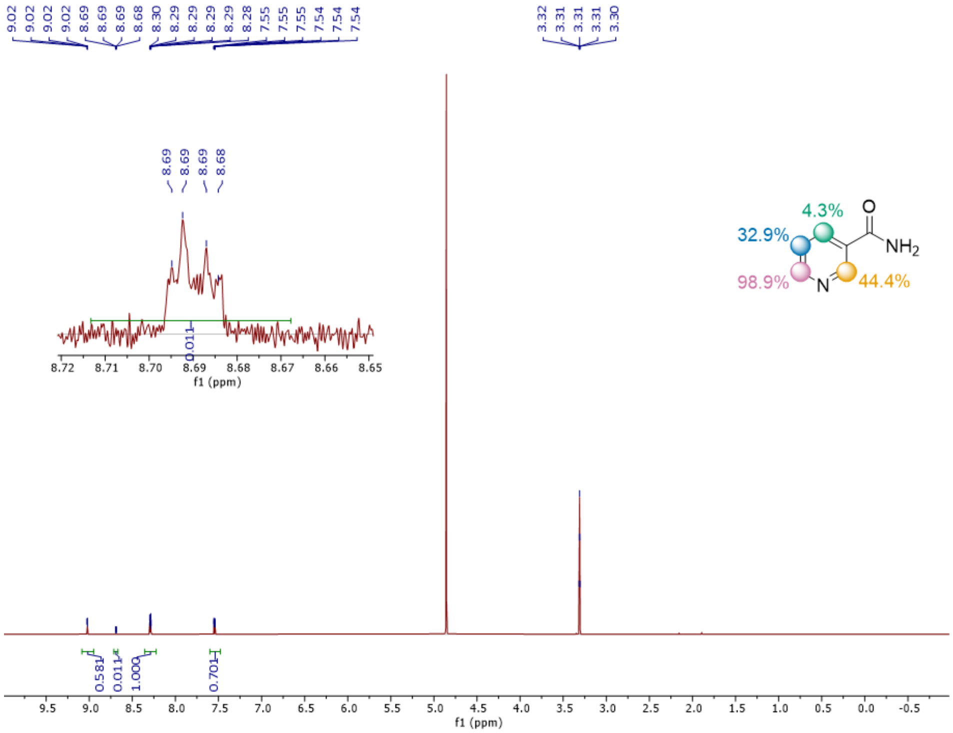

Fig. S198. ^{1}H **NMR (600 MHz, d1=30s, MeOD) of D-nicotinamide ([D]6).**

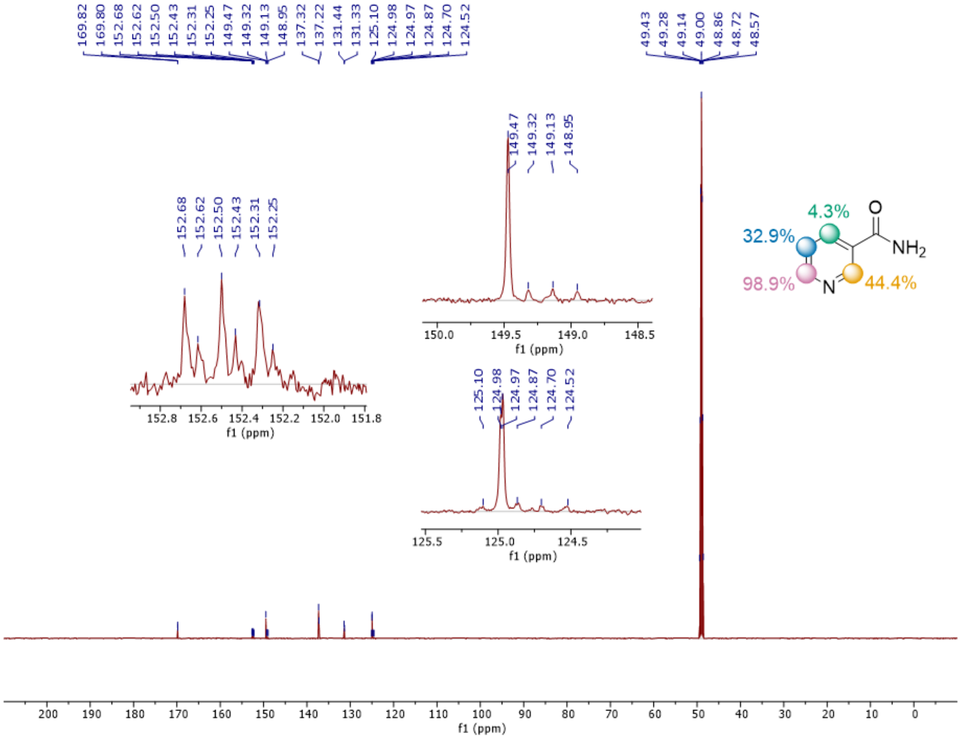

Fig. S199. $^{13}C\{^{1}H\}$ **NMR (151 MHz, MeOD) of D-nicotinamide ([D]6).**

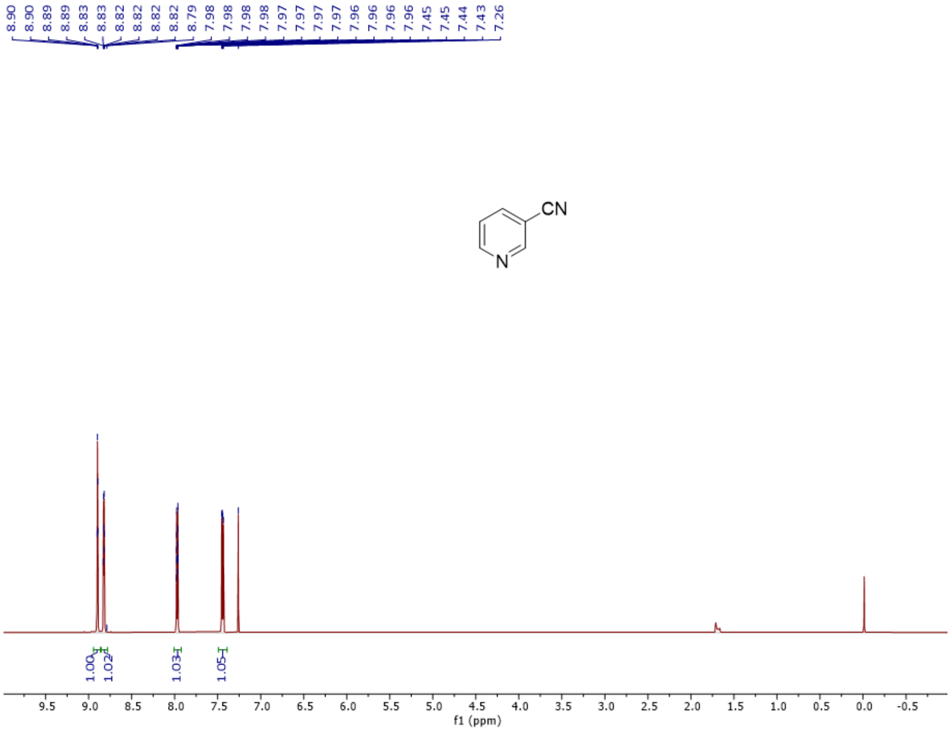

Fig. S200. ^{1}H **NMR (600 MHz, $CDCl_3$) of H-3-cyanopyridine ([H]3).**

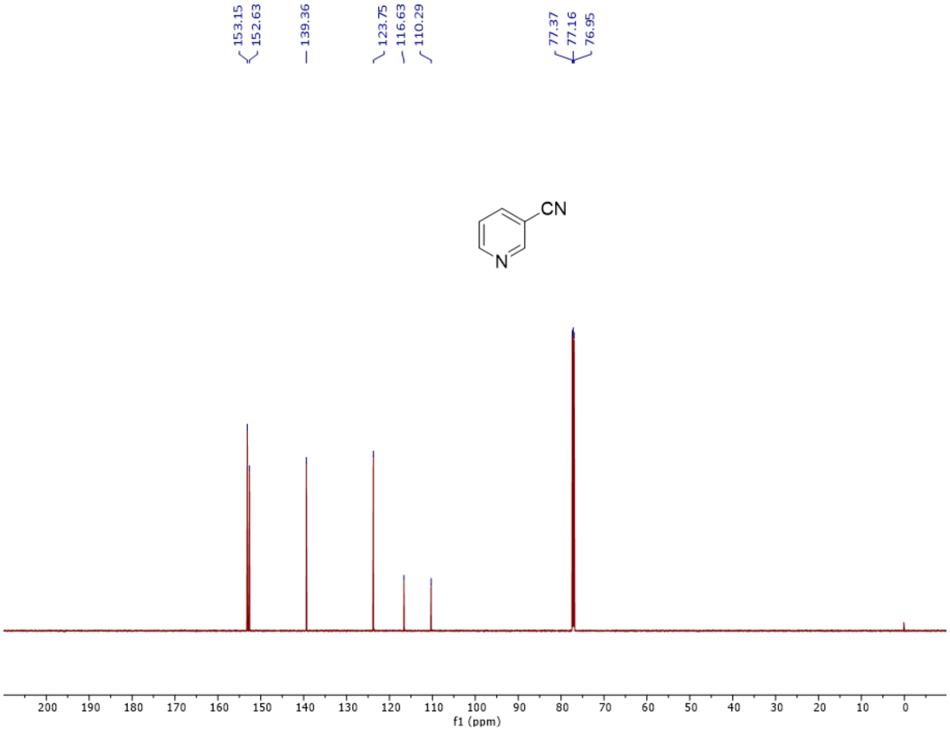

Fig. S201. $^{13}C\{^{1}H\}$ **NMR (151 MHz, $CDCl_3$) of H-3-cyanopyridine ([H]3).**

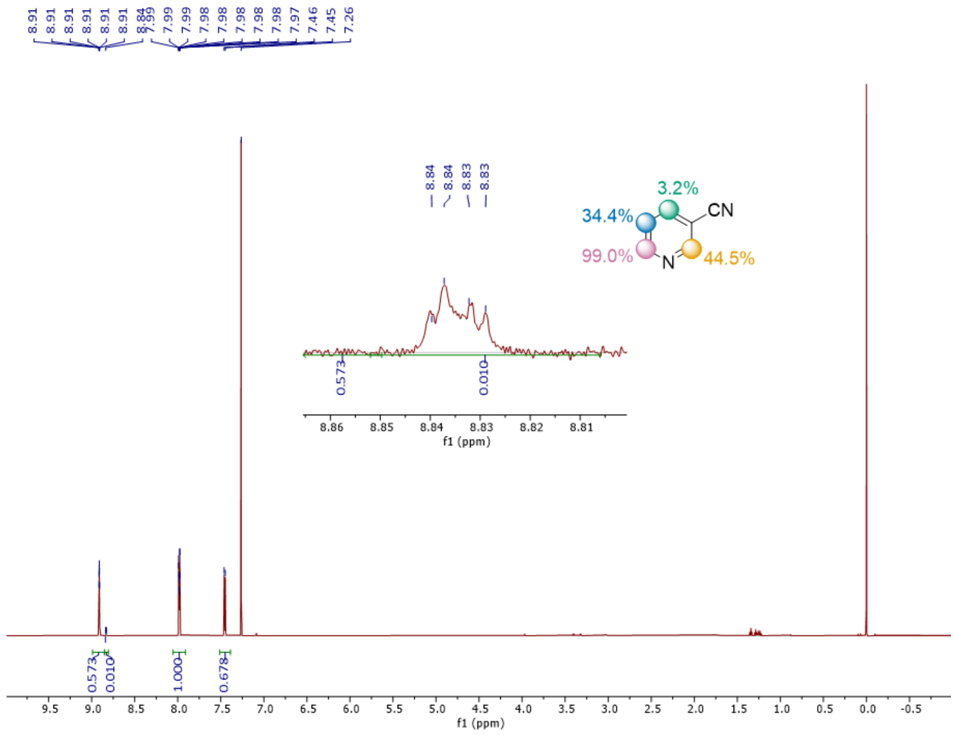

Fig. S202. ^{1}H **NMR (600 MHz, d1=30s,** $CDCl_3$**) of D-3-cyanopyridine ([D]3).**

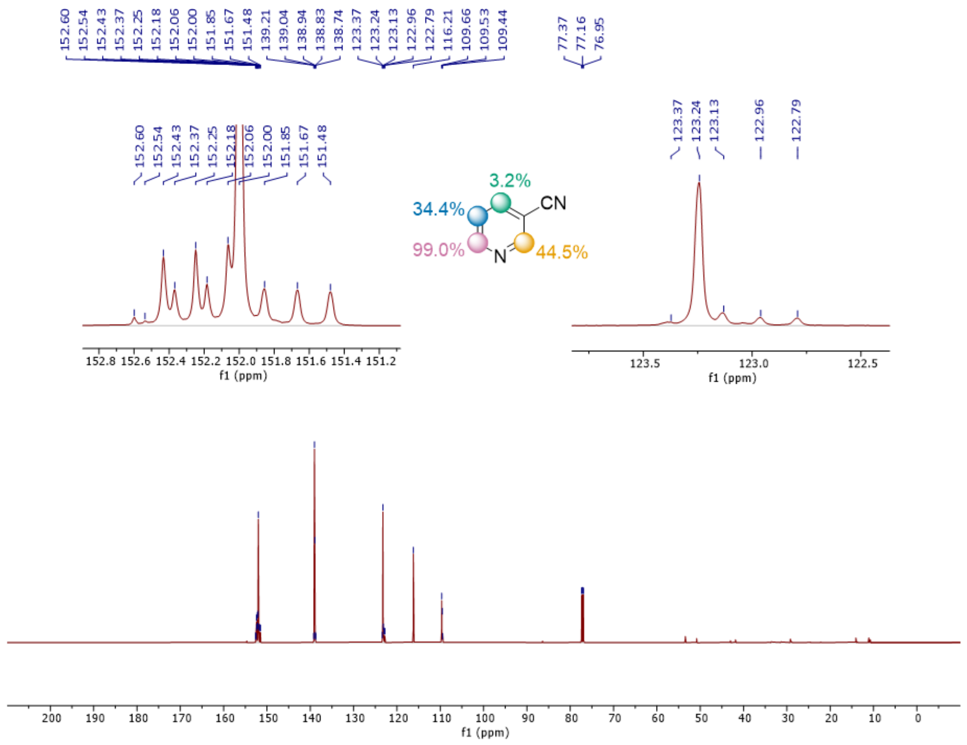

Fig. S203. $^{13}C\{^{1}H\}$ **NMR (151 MHz,** $CDCl_3$**) of D-3-cyanopyridine ([D]3).**

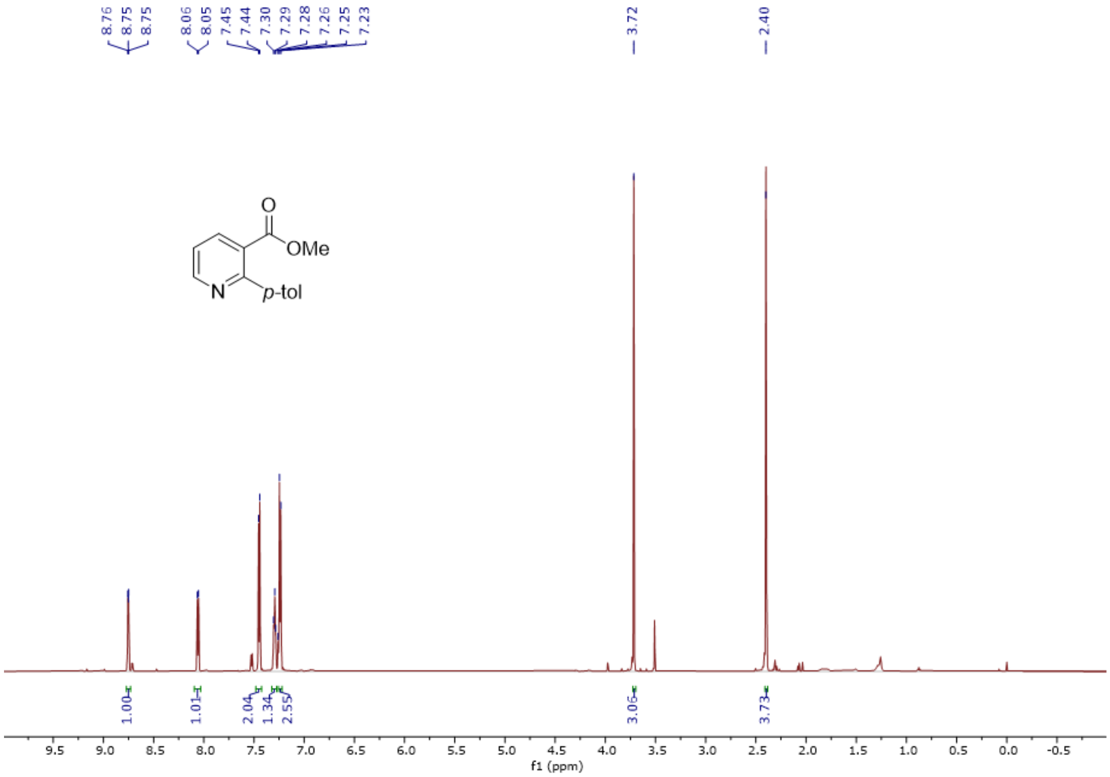

Fig. S204. ^{1}H NMR (600 MHz, $CDCl_3$) of methyl 2-(*para*-tolyl)nicotinate ([H]2a).

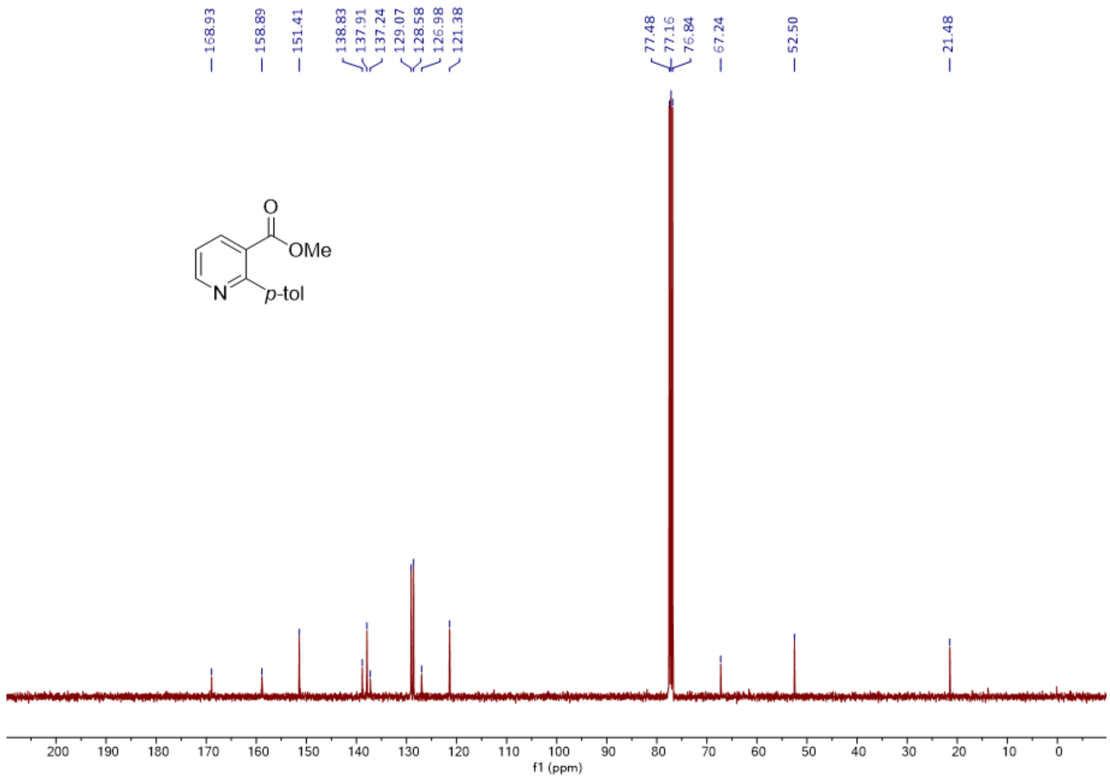

Fig. S205. $^{13}C\{^1H\}$ NMR (101 MHz, $CDCl_3$) of methyl 2-(*para*-tolyl)nicotinate ([H]2a).

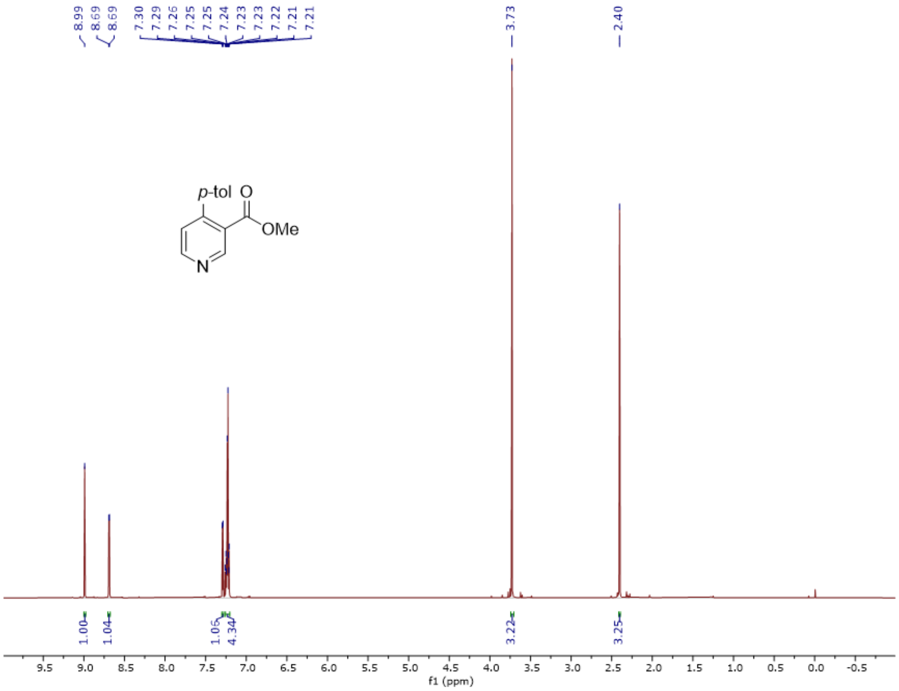

Fig. S206. ^{1}H **NMR (600 MHz, $CDCl_3$) of methyl 4-(*para*-tolyl)nicotinate ([H]2b).**

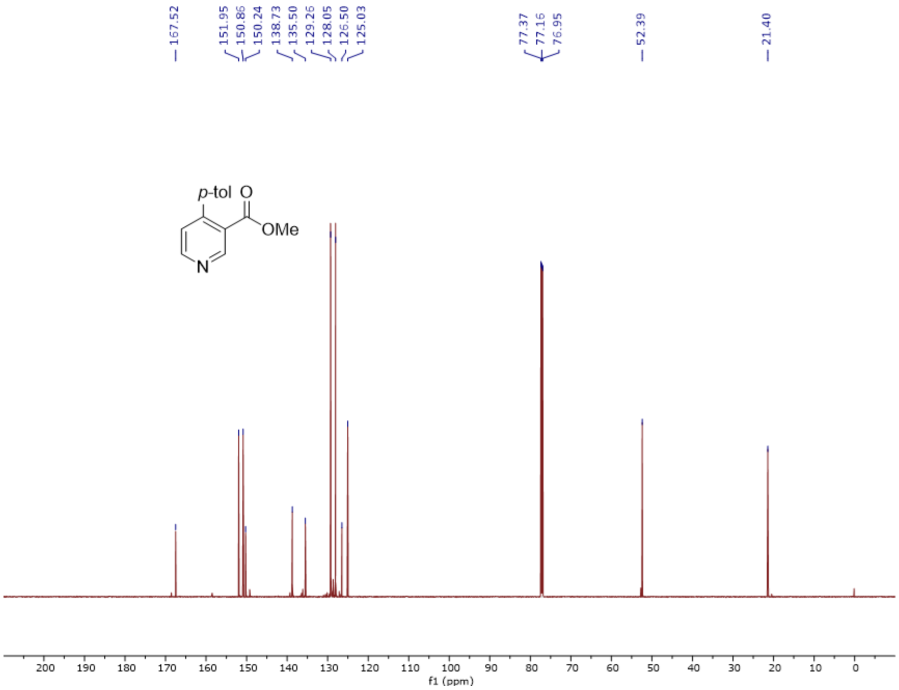

Fig. S207. $^{13}C\{^{1}H\}$ **NMR (151 MHz, $CDCl_3$) of methyl 4-(*para*-tolyl)nicotinate ([H]2b).**

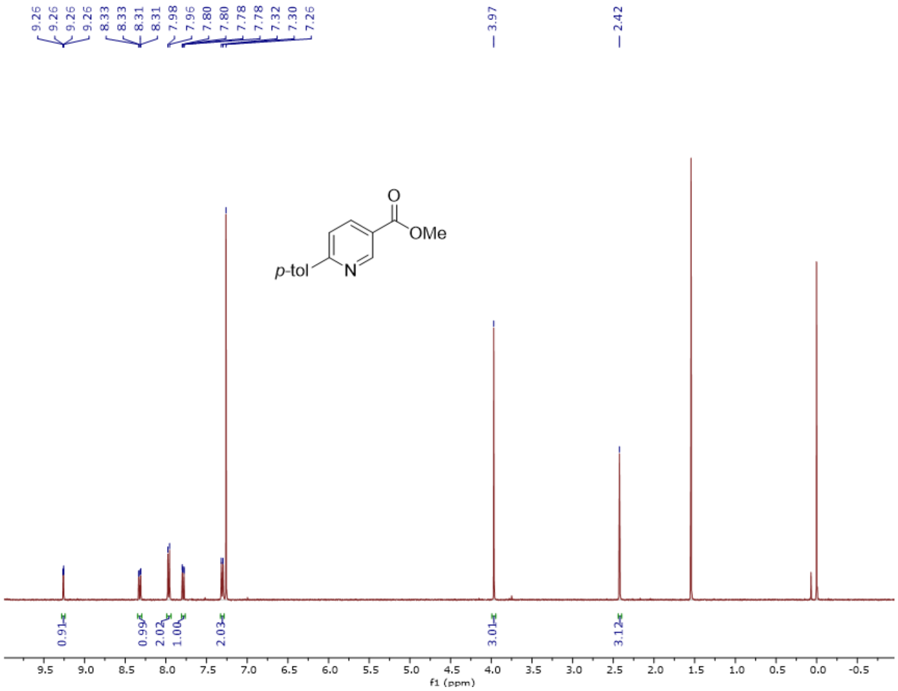

Fig. S208. ^{1}H NMR (600 MHz, $CDCl_3$) of methyl 6-(*para*-tolyl)nicotinate ([H]2c).

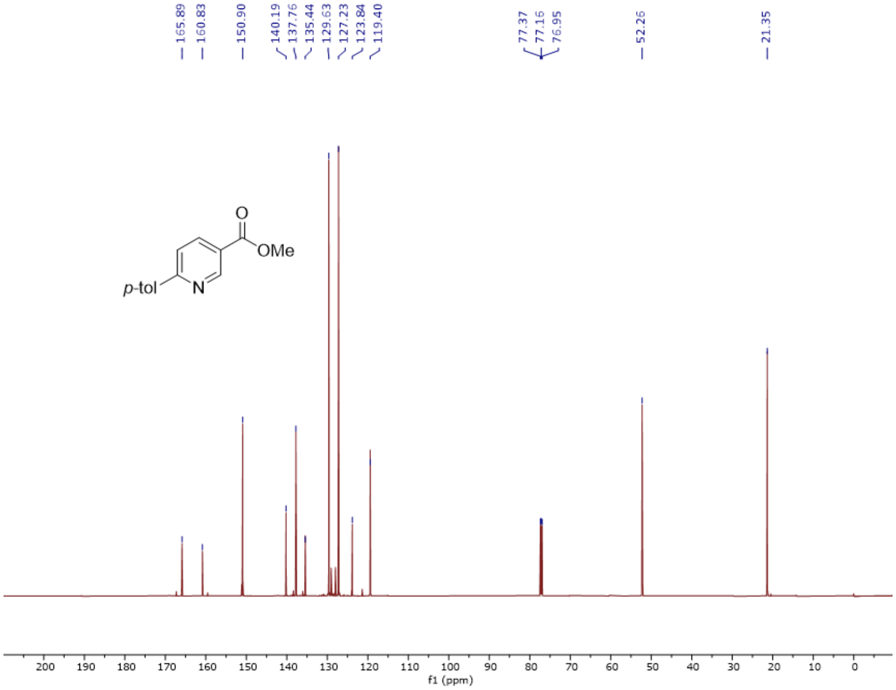

Fig. S209. $^{13}C\{^{1}H\}$ NMR (151 MHz, $CDCl_3$) of methyl 6-(*para*-tolyl)nicotinate ([H]2c).

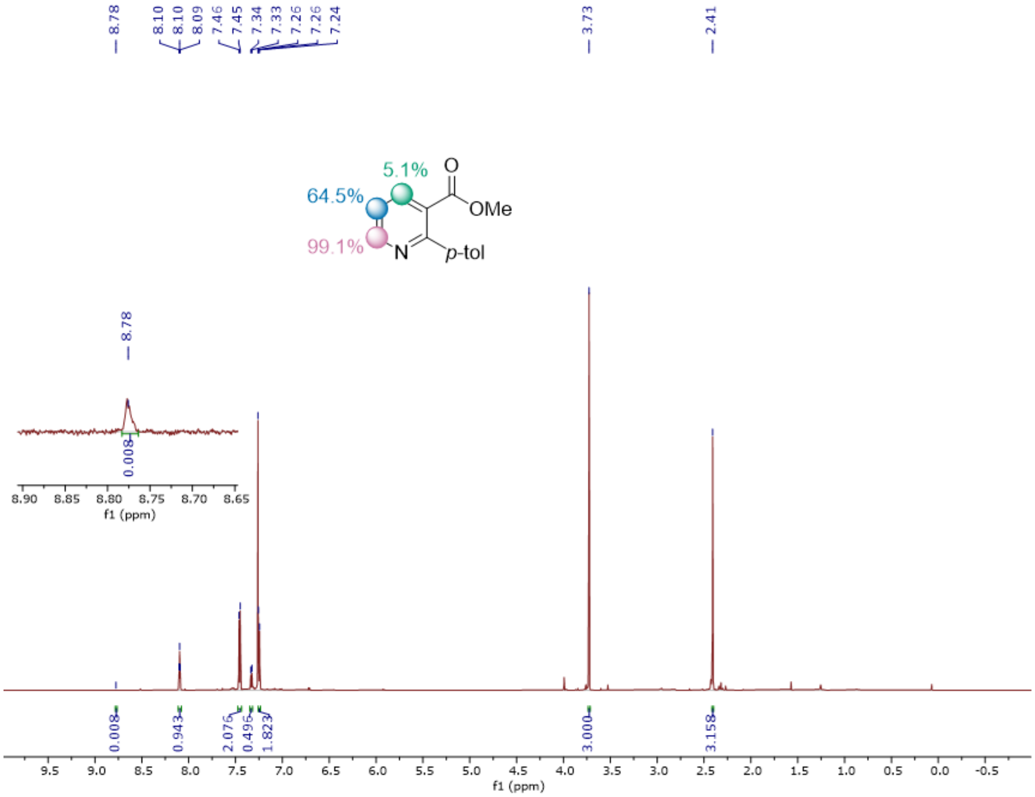

Fig. S210. ^{1}H NMR (600 MHz, $CDCl_3$) of [D]2a.

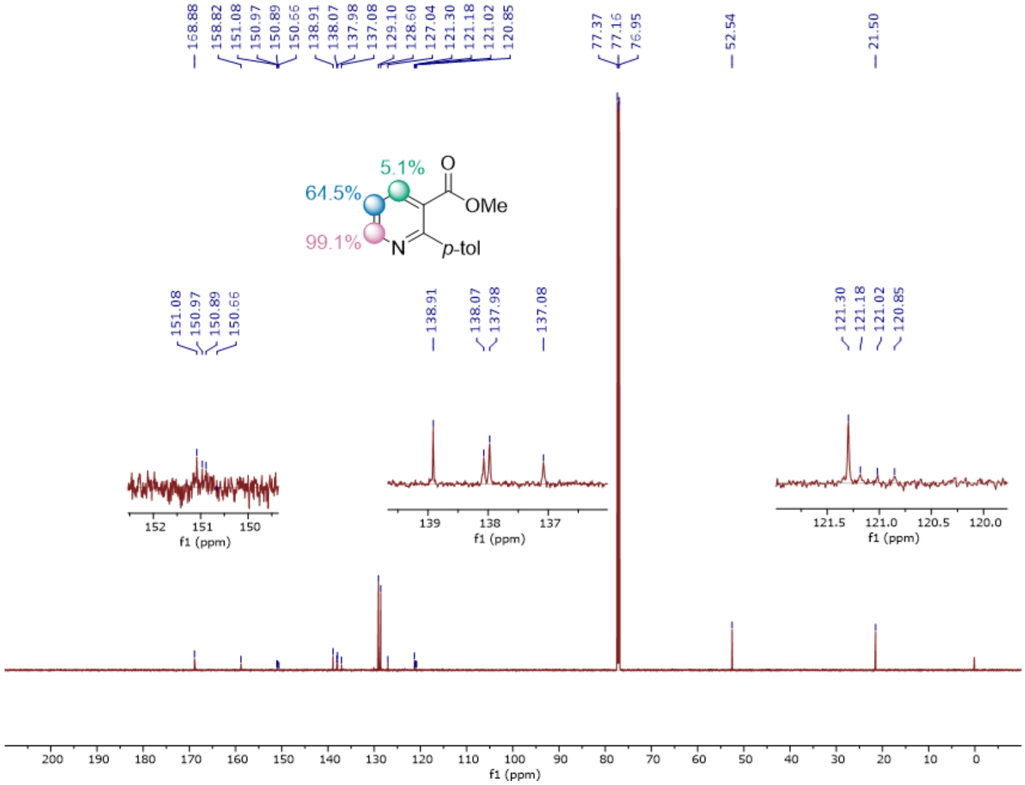

Fig. S211. $^{13}C\{^1H\}$ NMR (151 MHz, $CDCl_3$) of [D]2a.

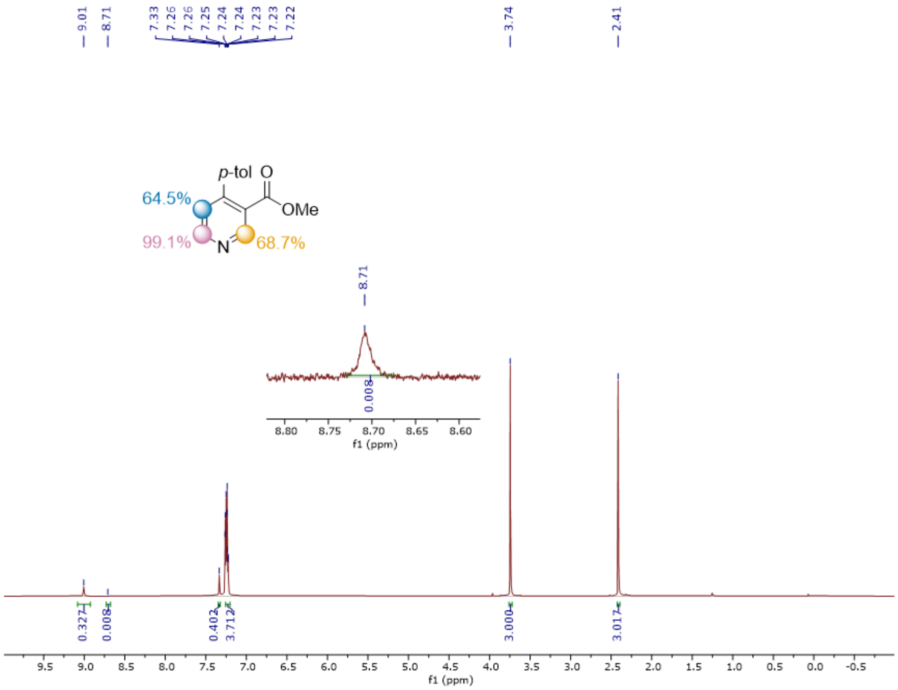

Fig. S212. ^{1}**H NMR (600 MHz, CDCl$_3$) of [D]2b.**

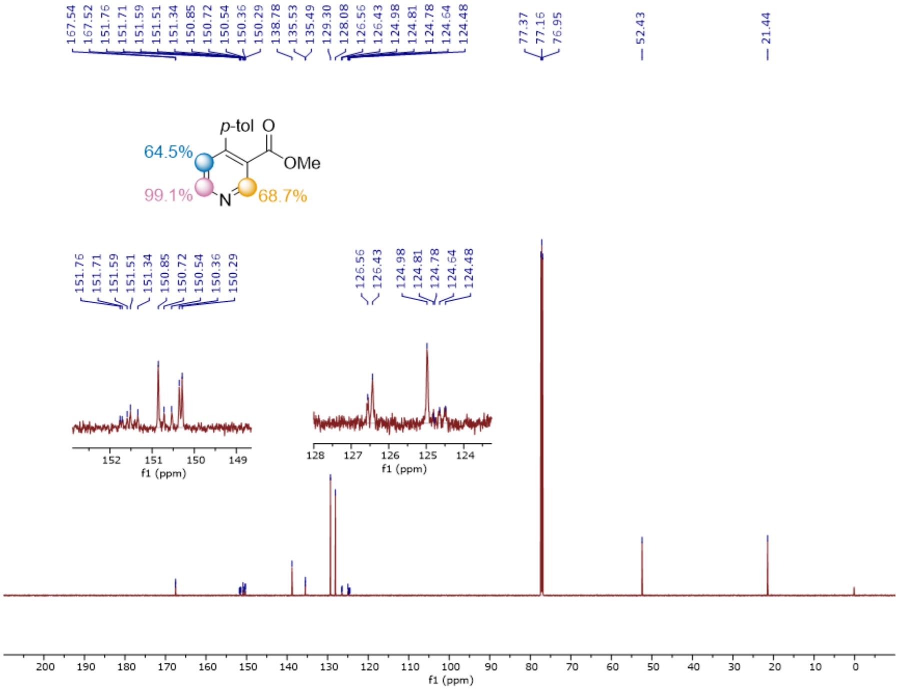

Fig. S213. ^{13}**C{^{1}H} NMR (151 MHz, CDCl$_3$) of [D]2b.**

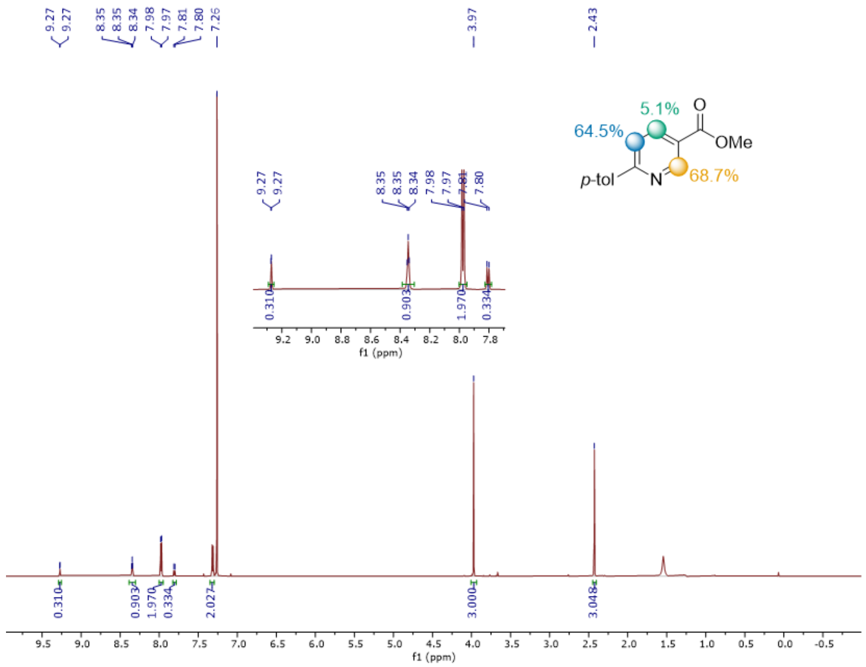

Fig. S214. ^{1}H NMR (600 MHz, $CDCl_3$) of [D]2c.

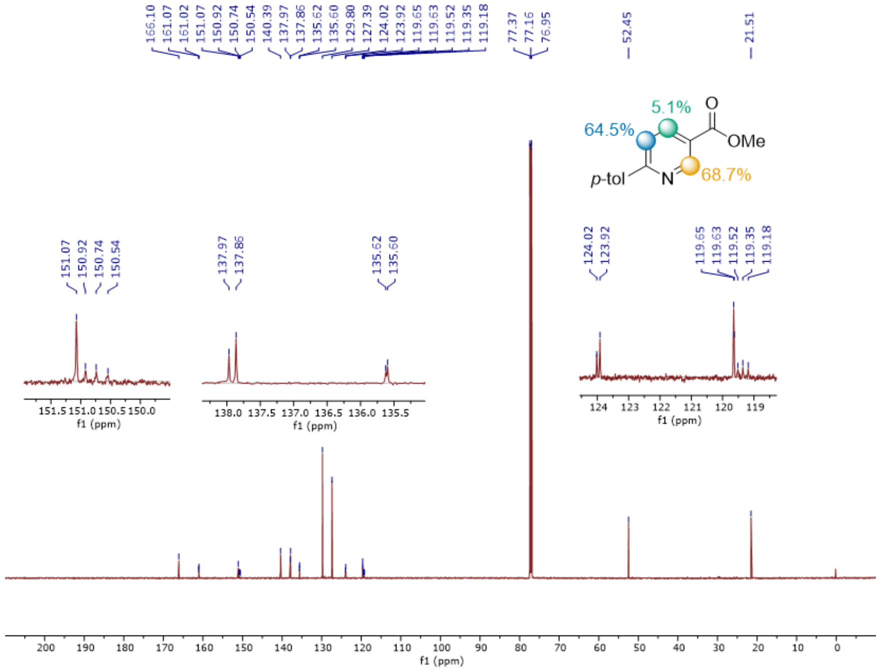

Fig. S215. $^{13}C\{^{1}H\}$ NMR (151 MHz, $CDCl_3$) of [D]2c.

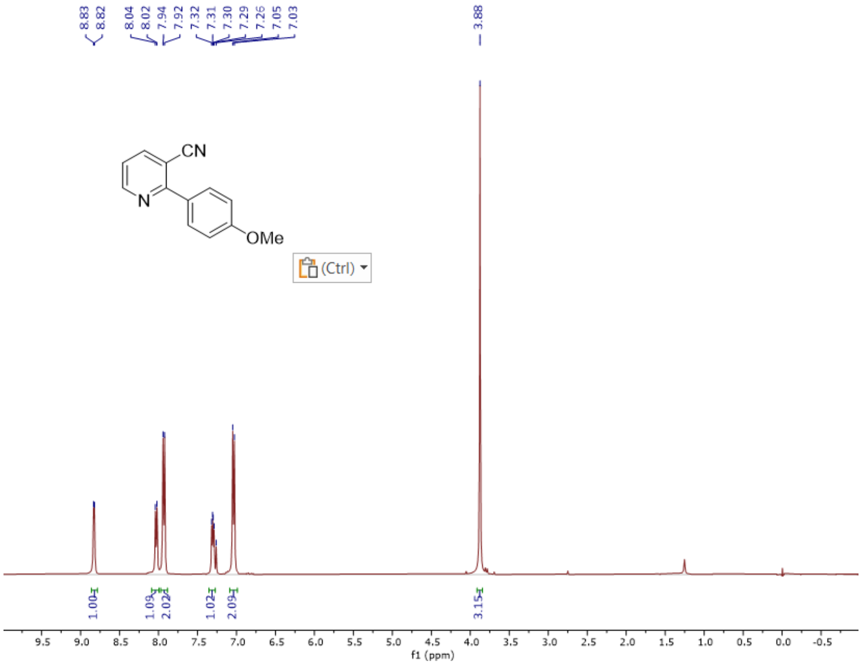

Fig. S216. ^{1}H NMR (400 MHz, $CDCl_3$) of [H]3a.

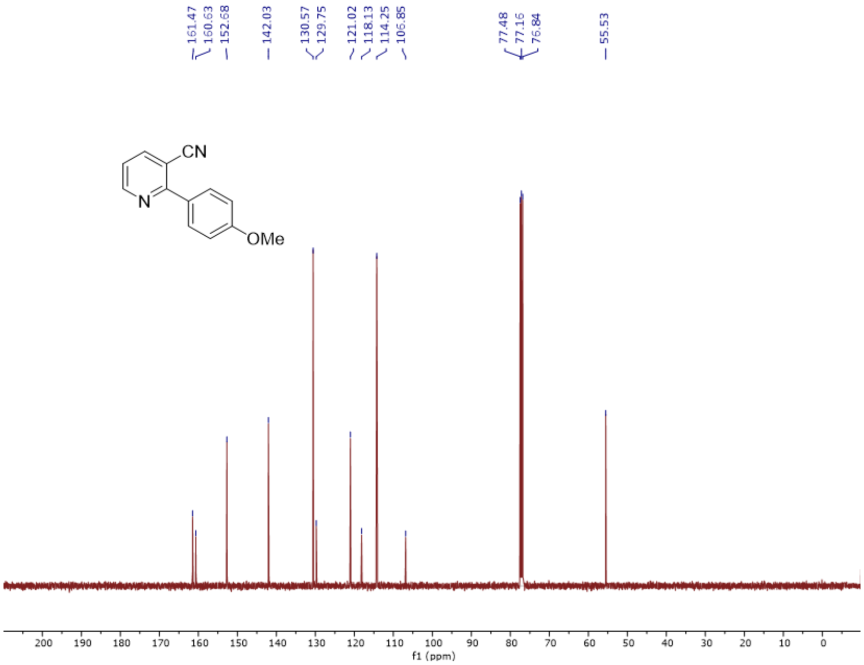

Fig. S217. $^{13}C\{^{1}H\}$ NMR (101 MHz, $CDCl_3$) of [H]3a.

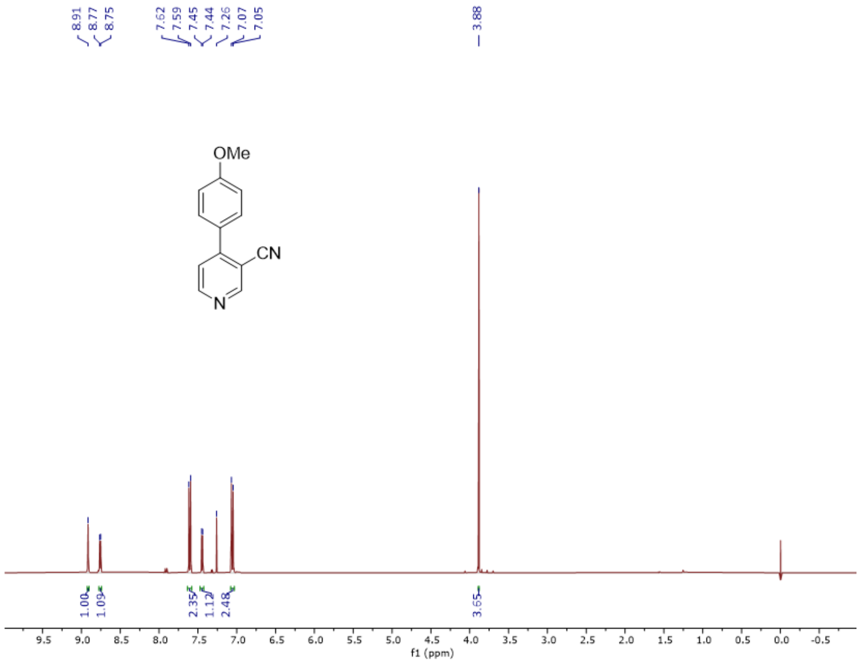

Fig. S218. ^{1}H **NMR (400 MHz,** $CDCl_3$**) of [H]3b.**

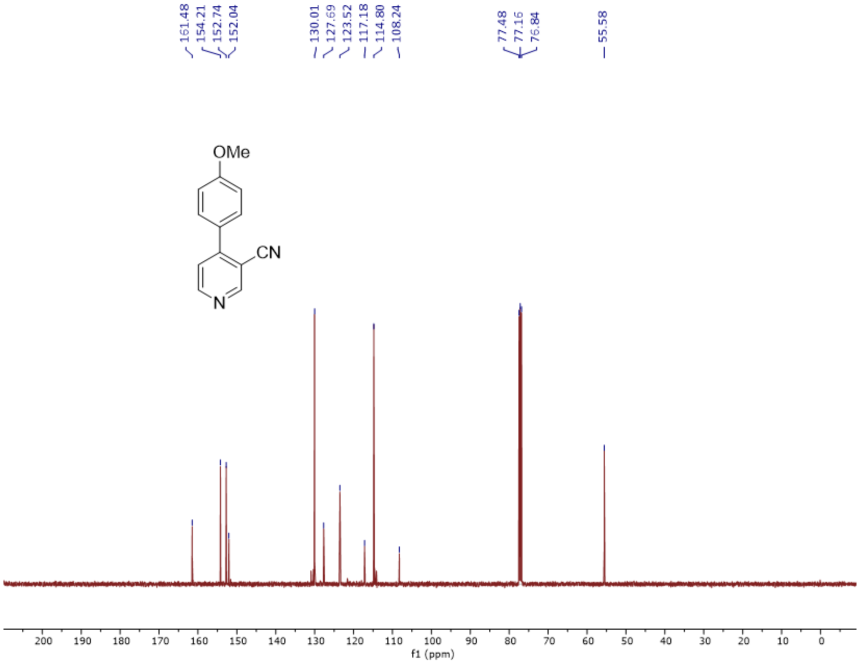

Fig. S219. $^{13}C\{^{1}H\}$ **NMR (101 MHz,** $CDCl_3$**) of [H]3b.**

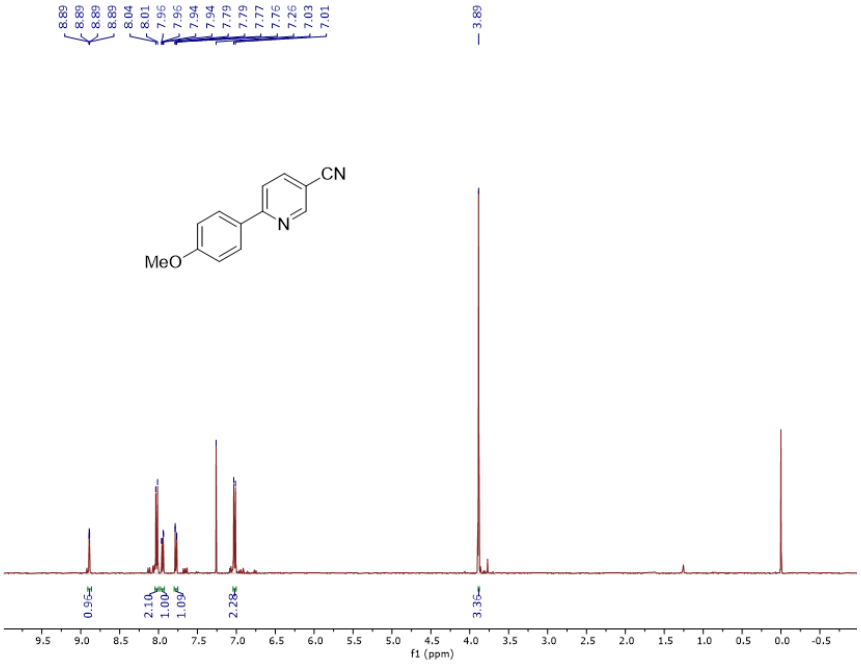

Fig. S220. ^{1}**H NMR (400 MHz, CDCl$_3$) of [H]3c.**

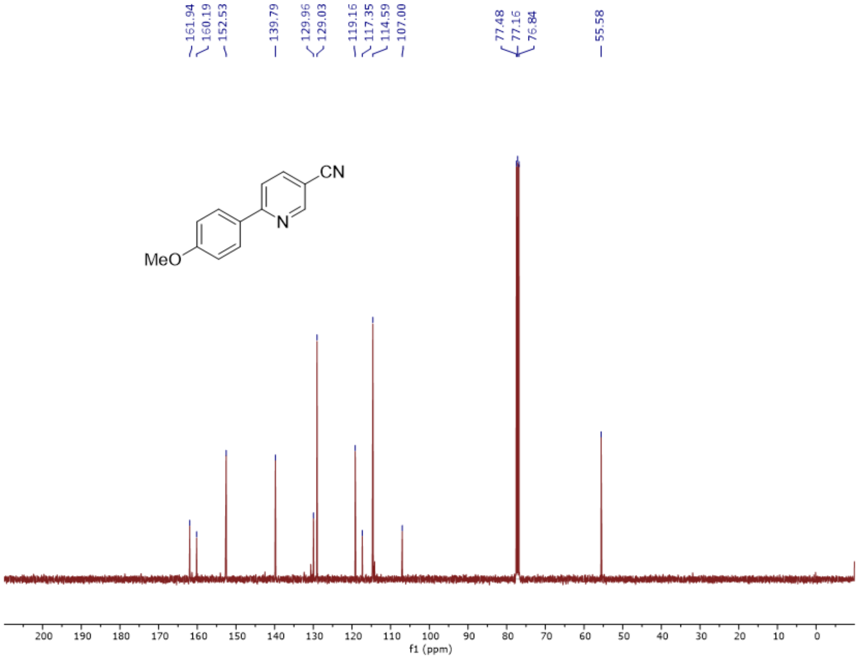

Fig. S221. ^{13}**C{^{1}H} NMR (101 MHz, CDCl$_3$) of [H]3c.**

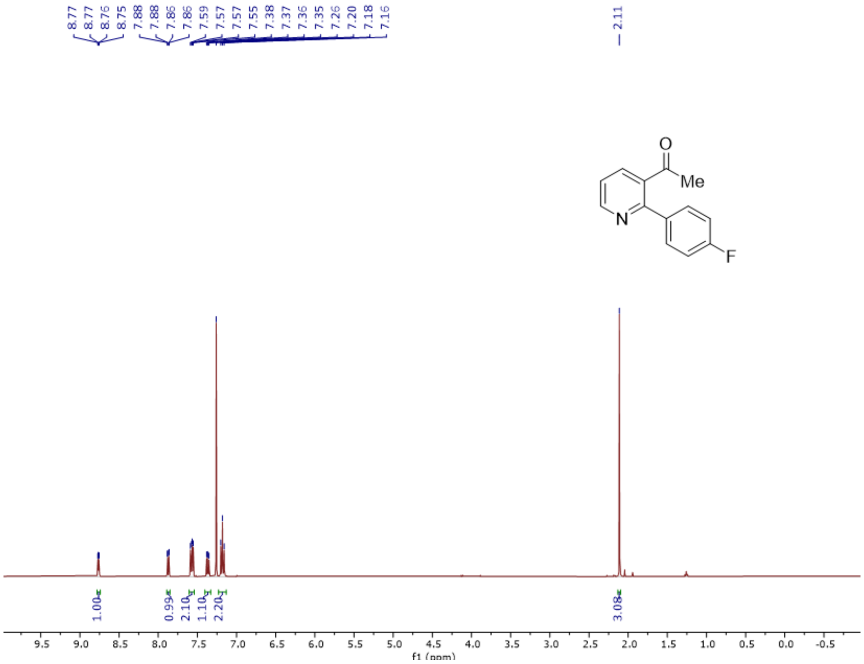

Fig. S222. ^{1}H **NMR (400 MHz, $CDCl_3$) of [H]4a.**

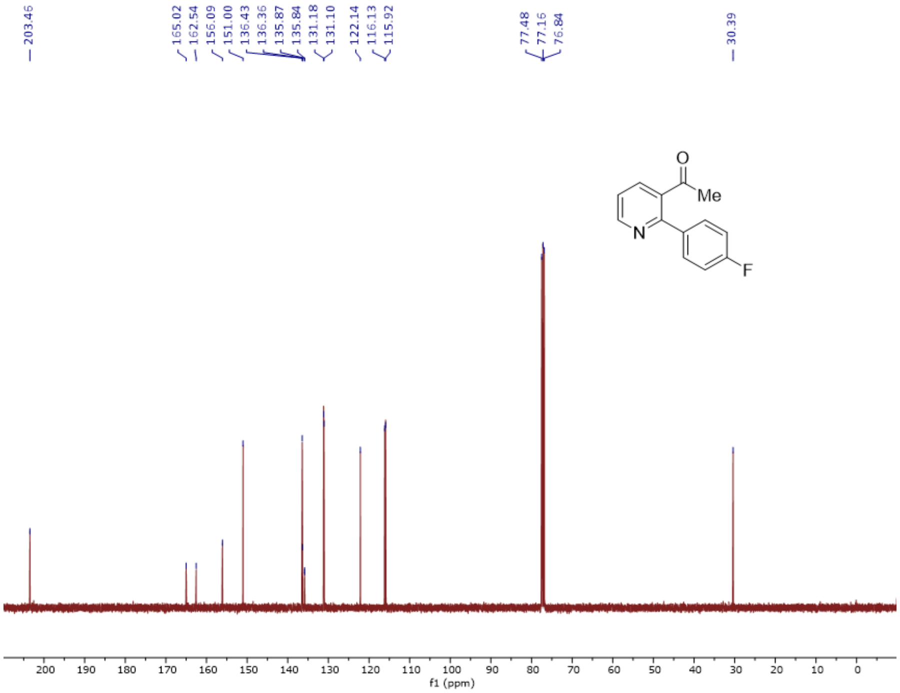

Fig. S223. ^{13}C **NMR{^{1}H} (101 MHz, $CDCl_3$) of [H]4a.**

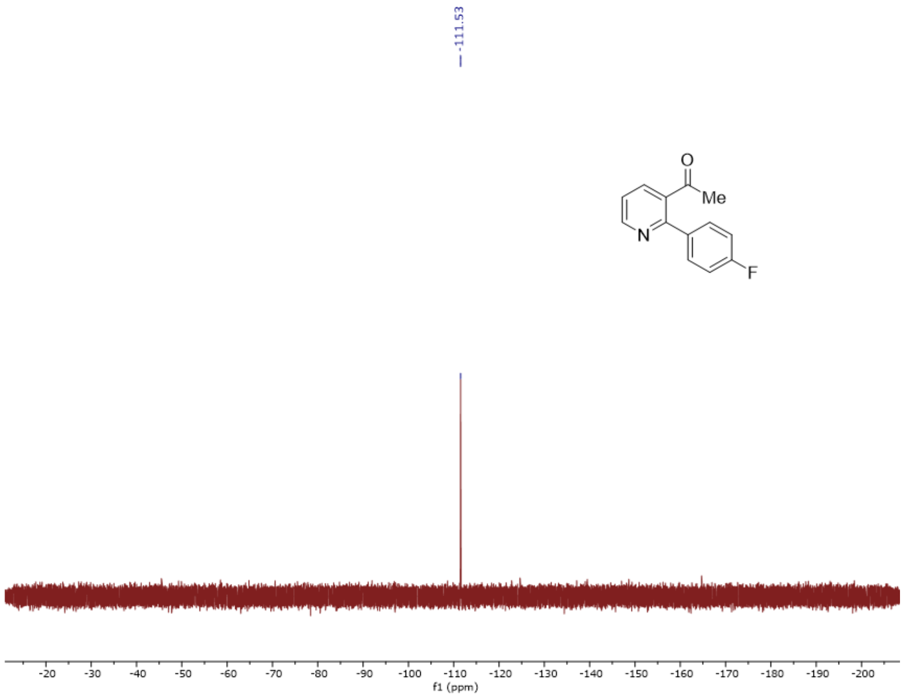

Fig. S224. ^{19}F NMR (376 MHz, $CDCl_3$) of [H]4a.

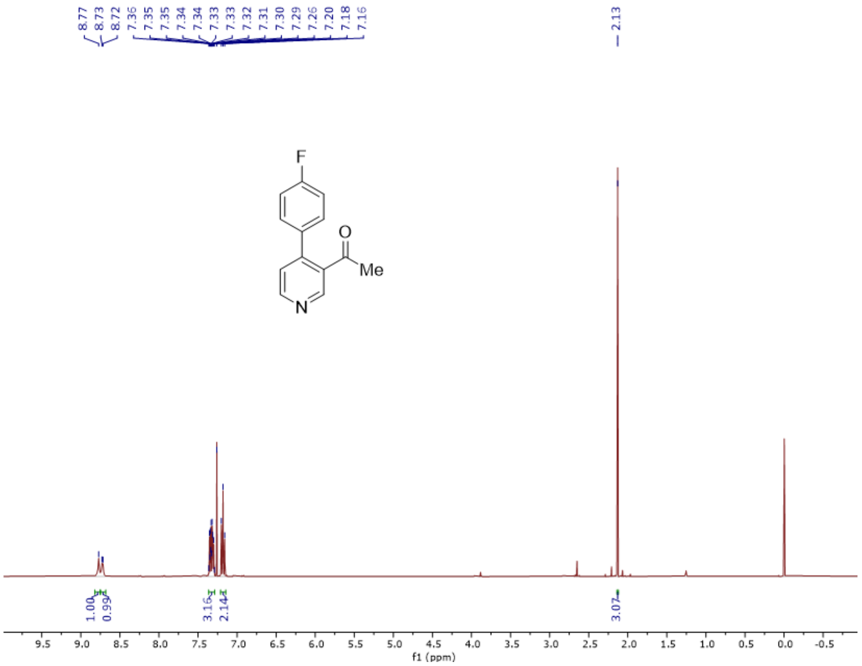

Fig. S225. ^{1}H NMR (400 MHz, $CDCl_3$) of [H]4b.

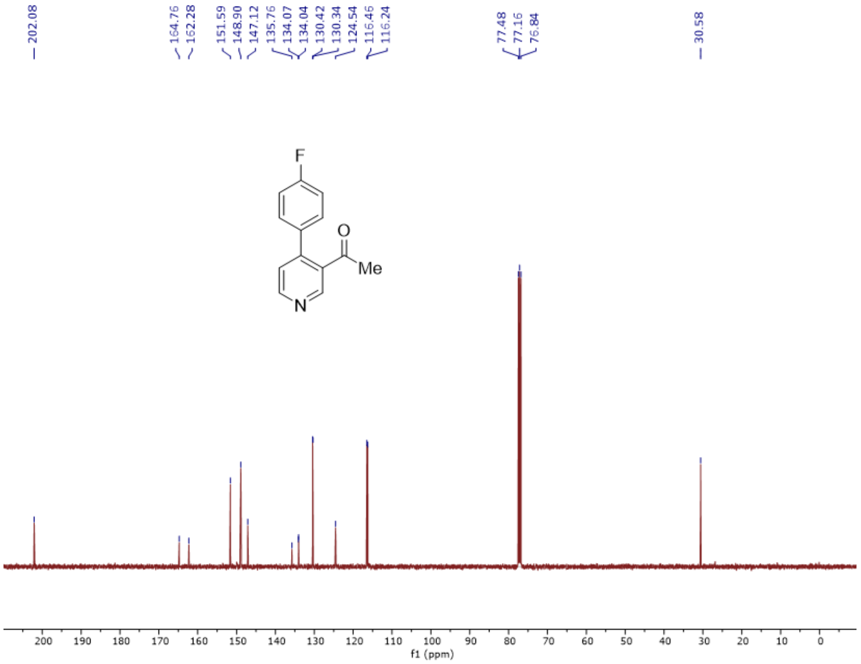

Fig. S226. ^{13}C NMR{^{1}H} (101 MHz, $CDCl_3$) of [H]4b.

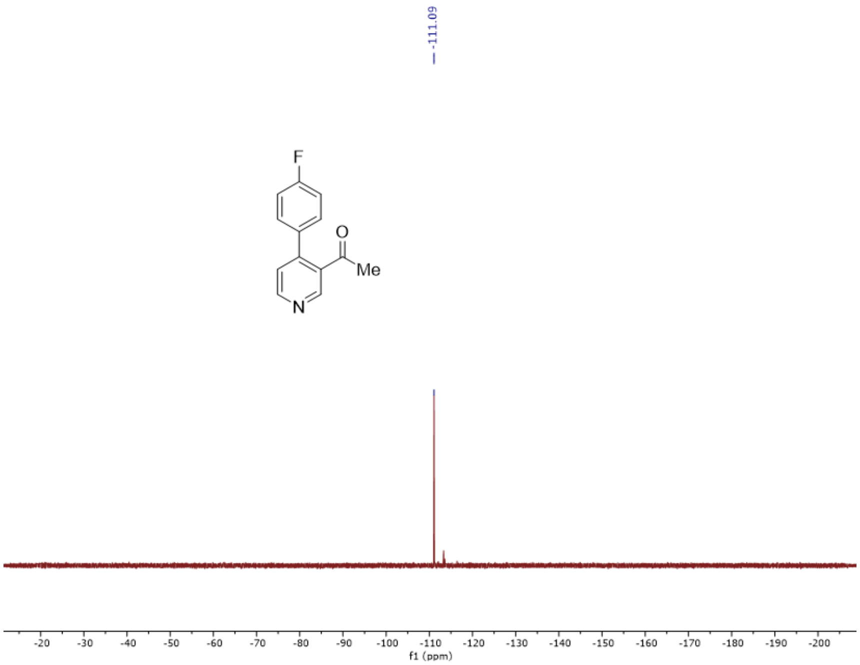

Fig. S227. ^{19}**F NMR (376 MHz,** $CDCl_3$**) of [H]4b.**

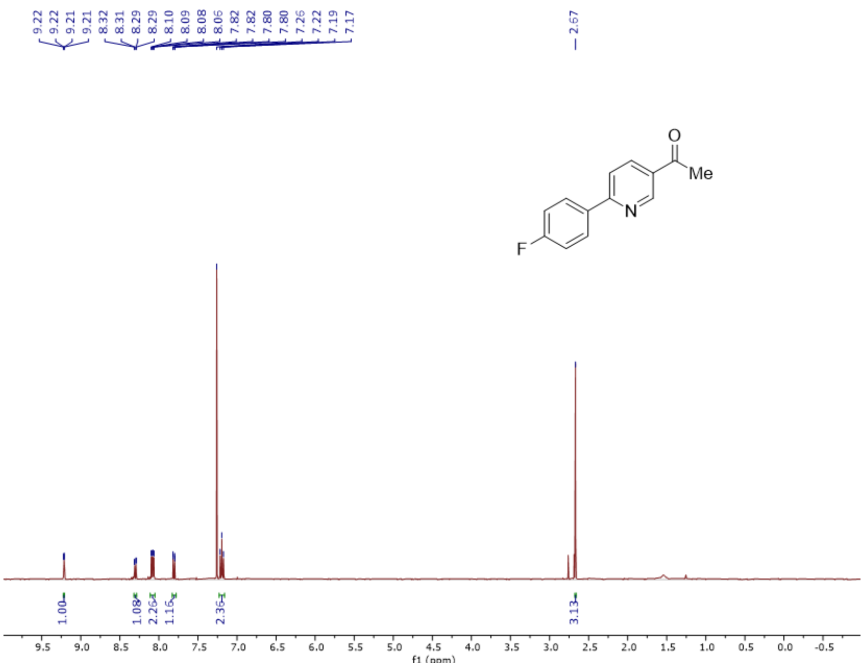

Fig. S228. ^{1}H NMR (400 MHz, $CDCl_3$) of [H]4c.

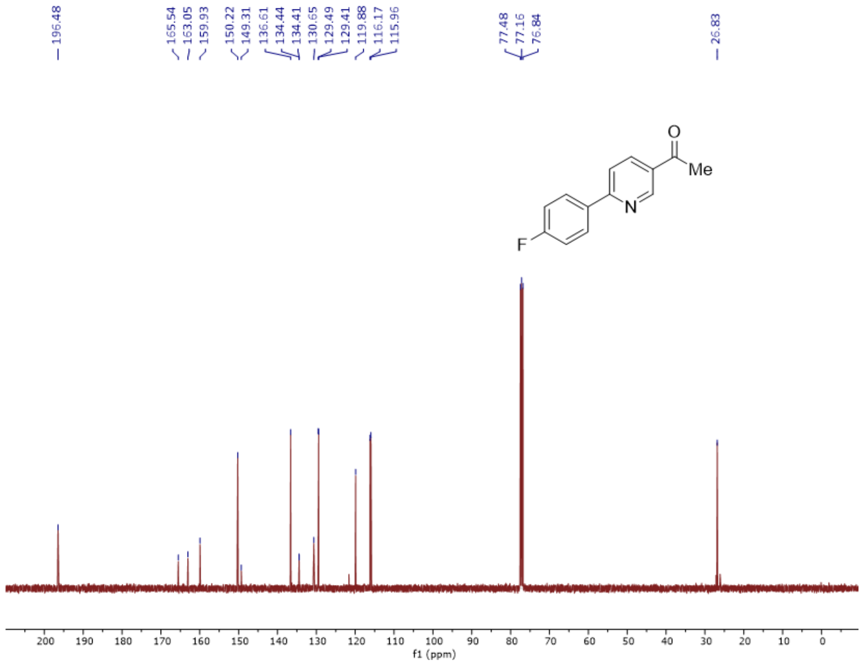

Fig. S229. ^{13}C NMR{^{1}H} (101 MHz, $CDCl_3$) of [H]4c.

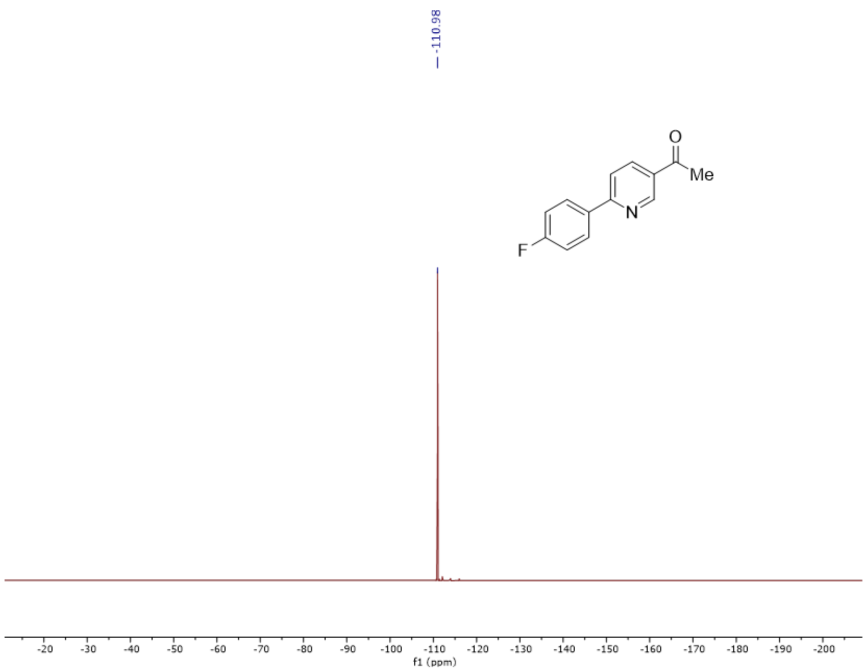

Fig. S230. ^{19}F NMR (376 MHz, $CDCl_3$) of [H]4c.

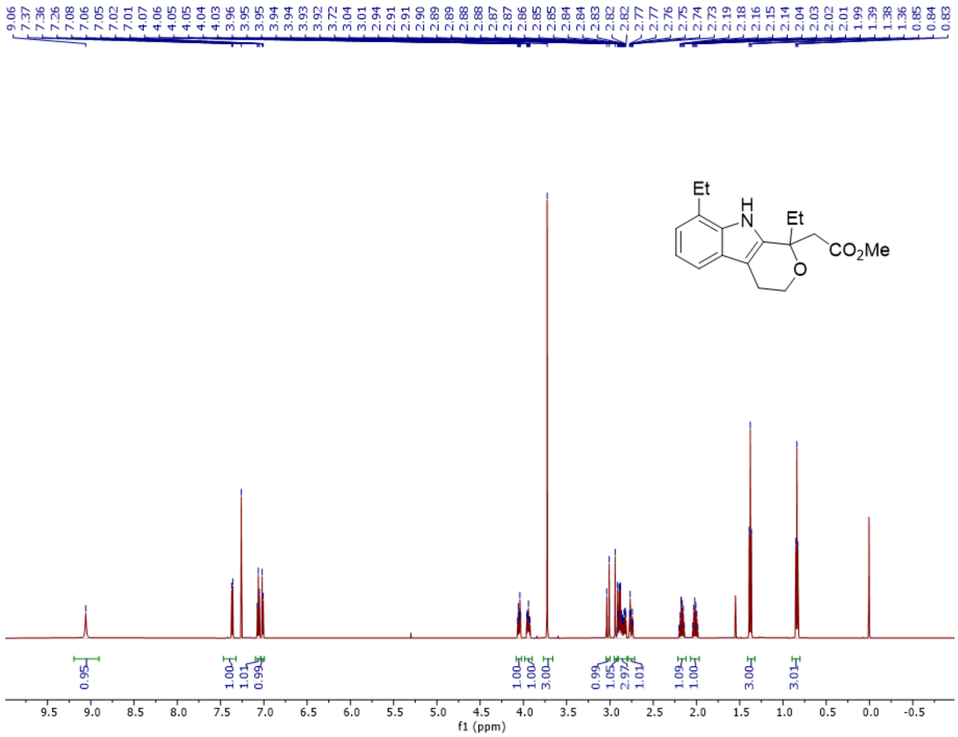

Fig. S231. **^{1}H NMR (600 MHz, $CDCl_3$) of H-etodolac methyl ester ([H]8.**

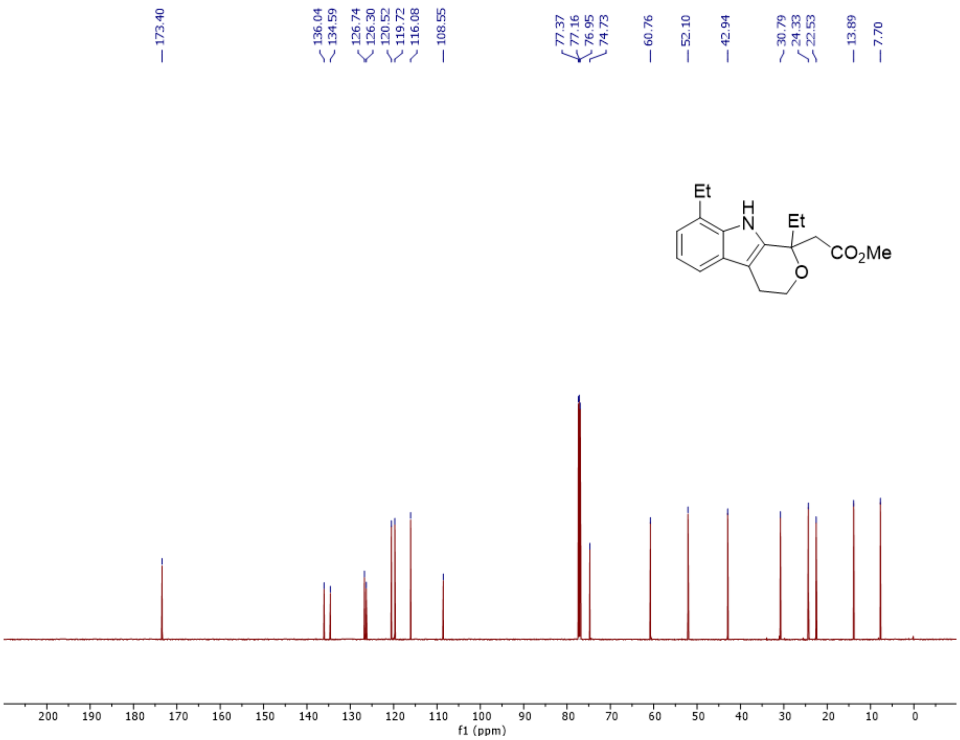

Fig. S232. **$^{13}C\{^{1}H\}$ NMR (151 MHz, $CDCl_3$) of H-etodolac methyl ester ([H]8).**

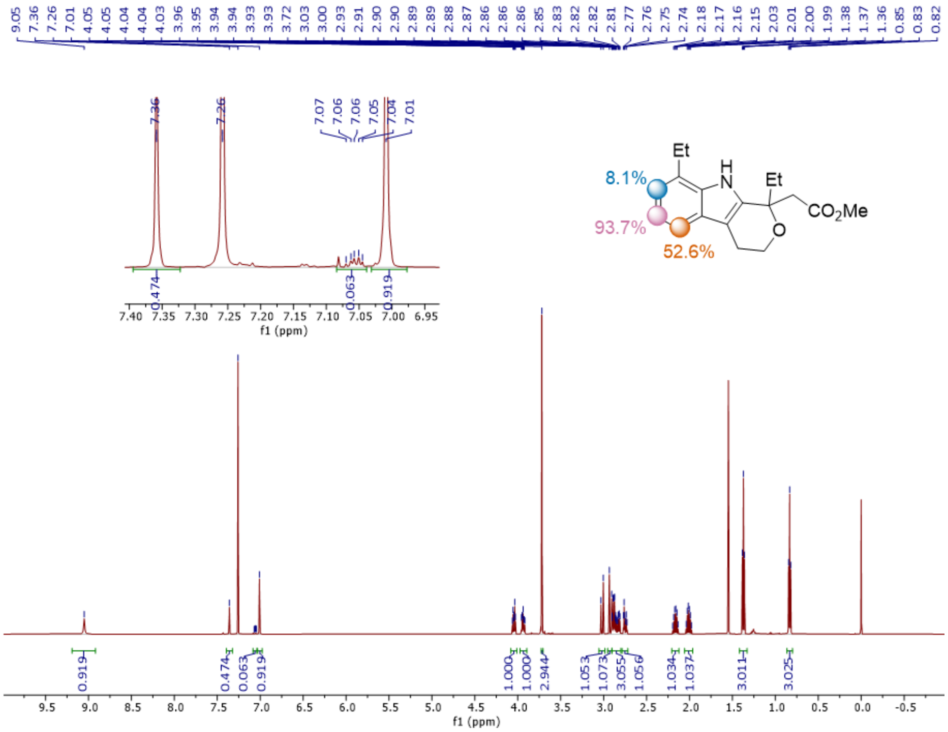

Fig. S233. ^{1}H NMR (600 MHz, d1=30s, $CDCl_3$) of D-etodolac methyl ester ([D]8).

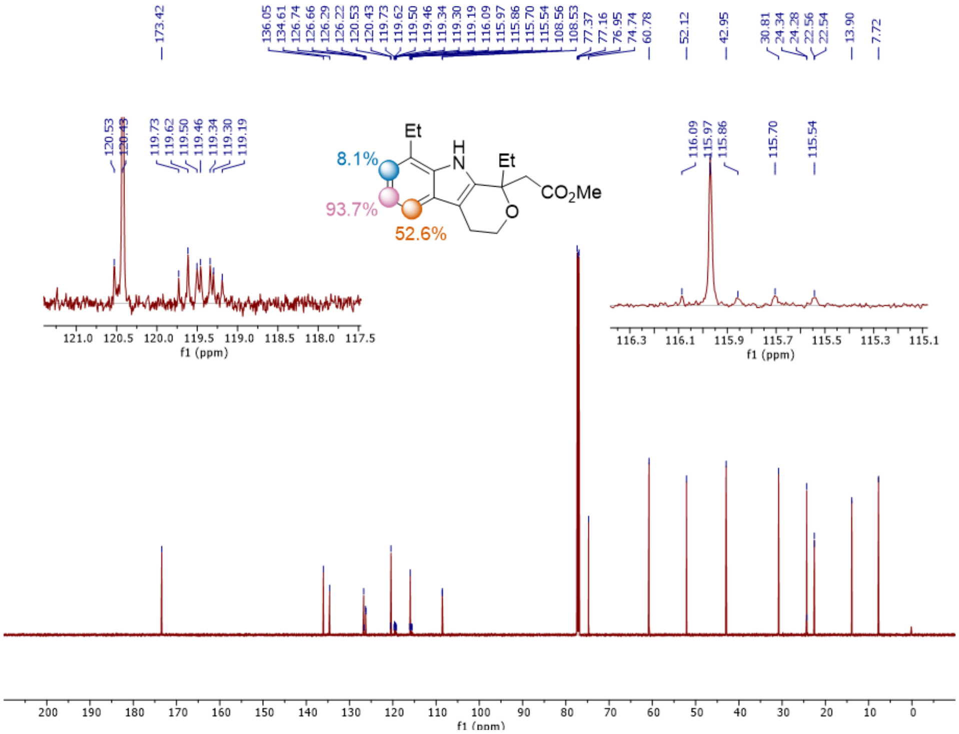

Fig. S234. $^{13}C\{^{1}H\}$ NMR (151 MHz, $CDCl_3$) of D-etodolac methyl ester ([D]8).

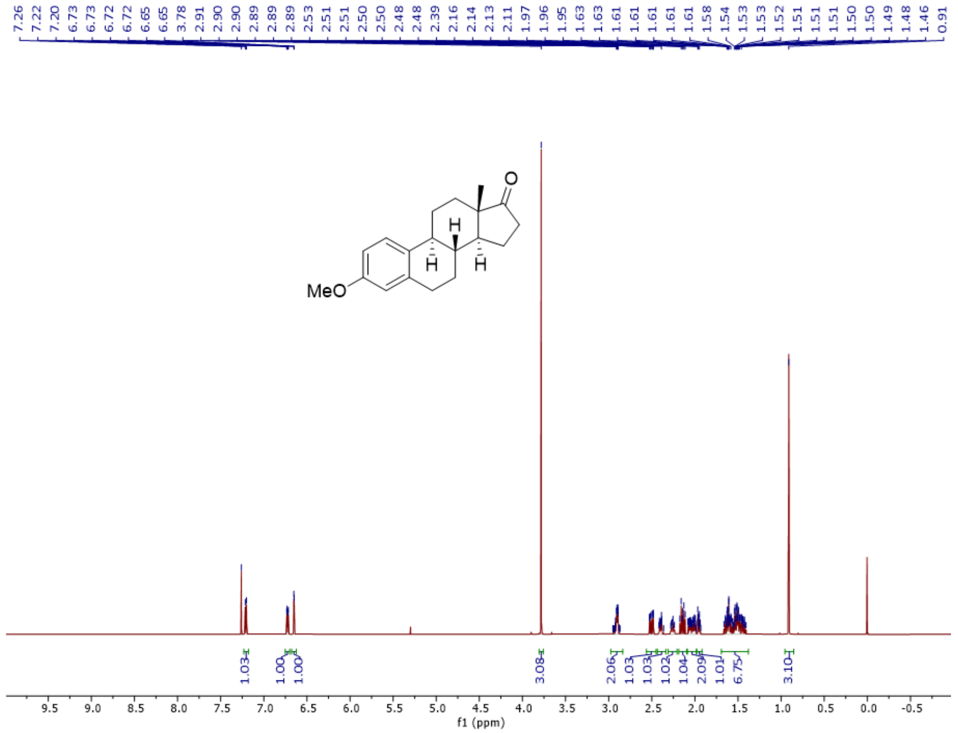

Fig. S235. ^{1}H NMR (600 MHz, $CDCl_3$) of H-estrone methyl ether ([H]9).

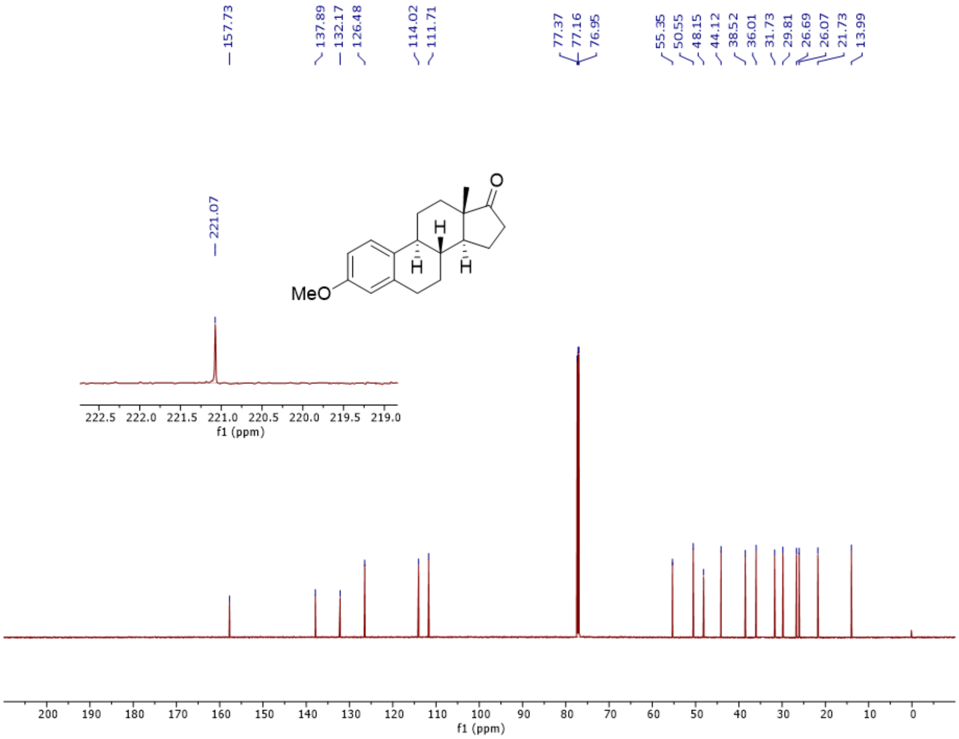

Fig. S236. $^{13}C\{^{1}H\}$ NMR (151 MHz, $CDCl_3$) of H-estrone methyl ether ([H]9).

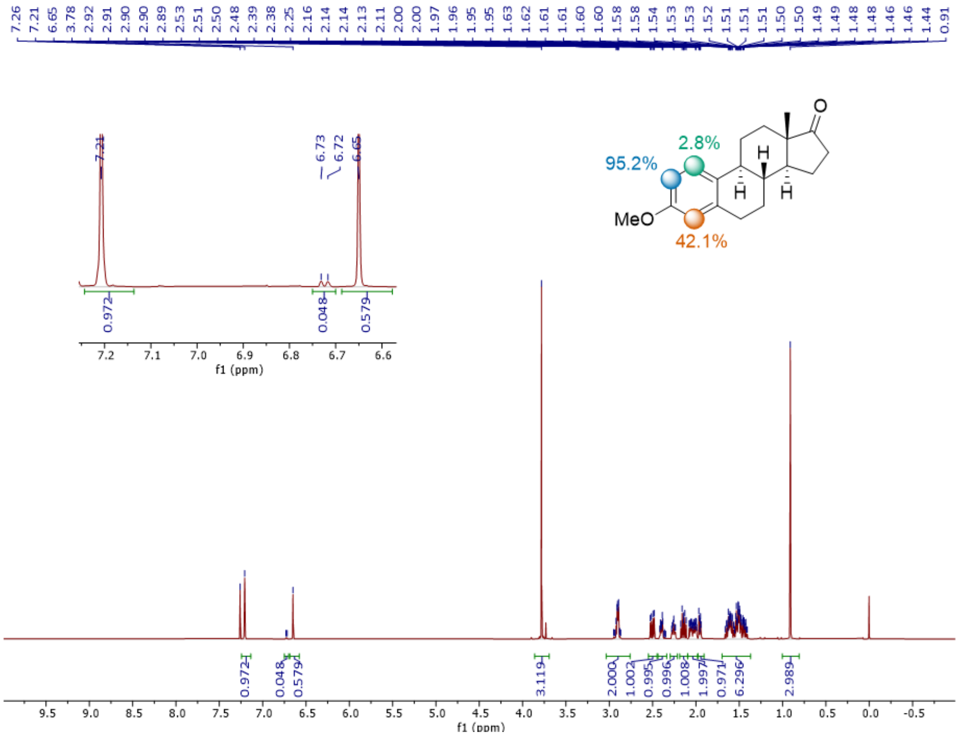

Fig. S237. ^{1}H NMR (600 MHz, d1=30s, $CDCl_3$) of D-estrone methyl ether ([D]9).

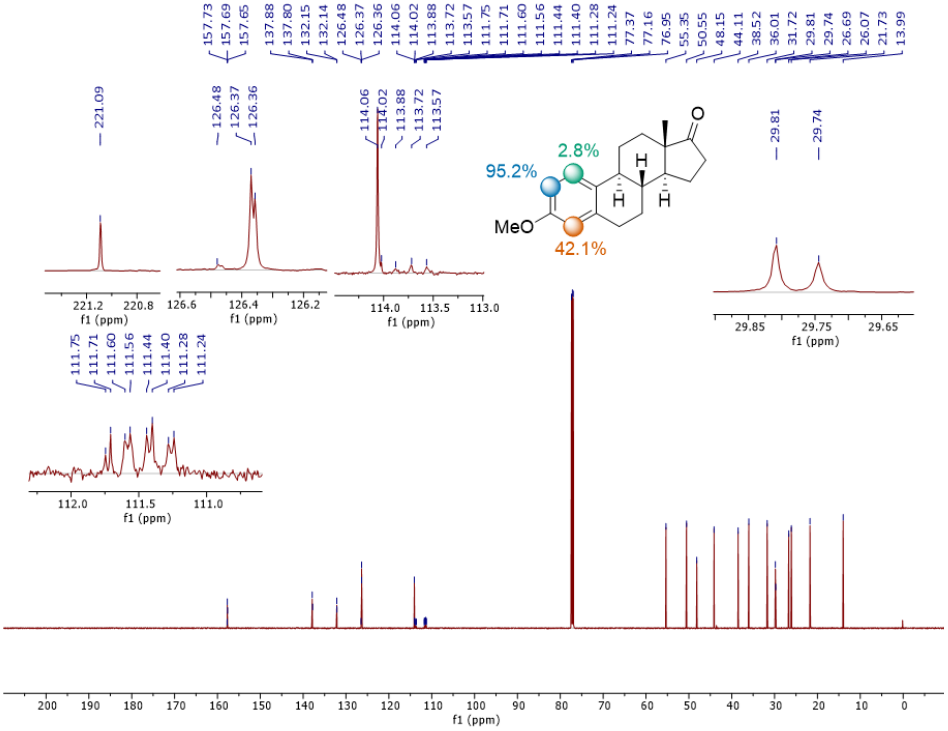

Fig. S238. $^{13}C\{^{1}H\}$ NMR (151 MHz, $CDCl_3$) of D-estrone methyl ether ([D]9).

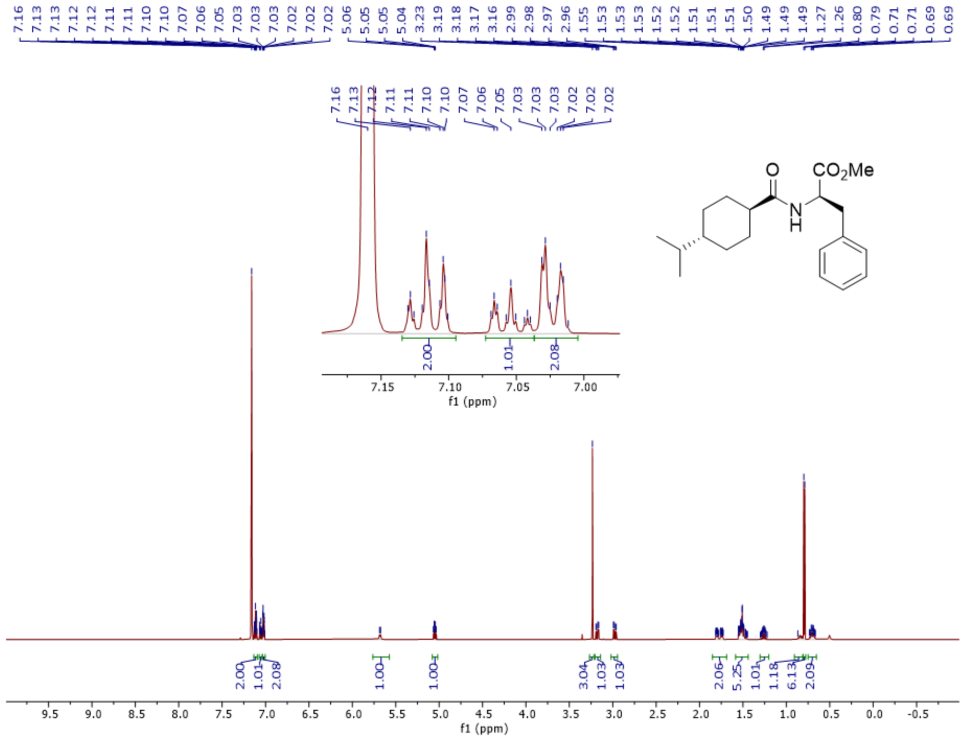

Fig. S239. ^{1}H NMR (600 MHz, C_6D_6) of H-nateglinide methyl ester ([H]10).

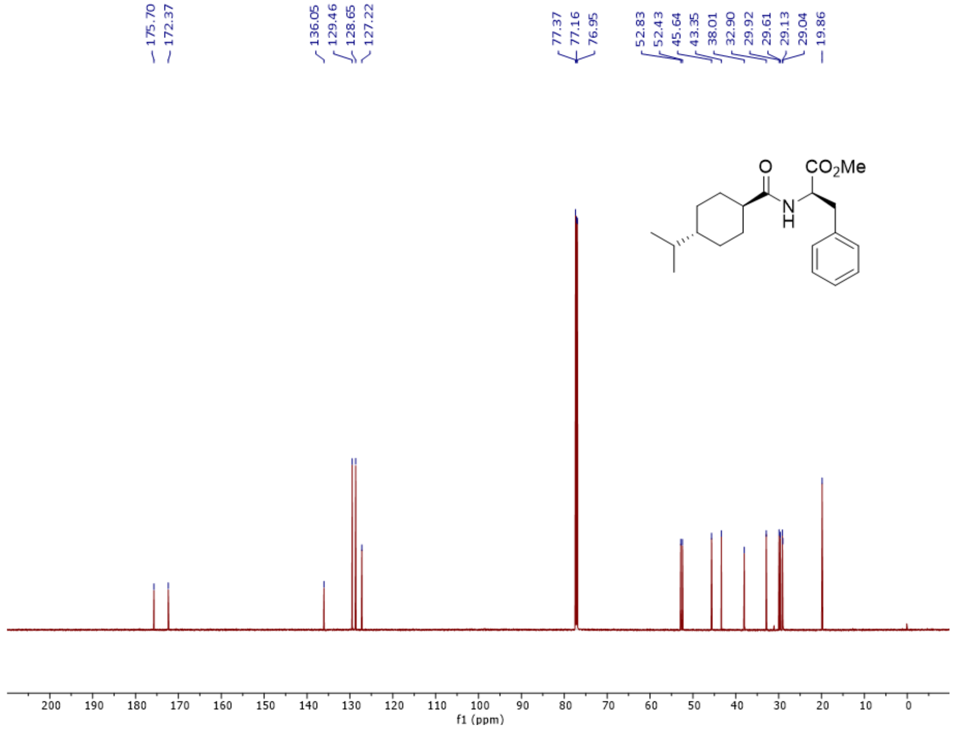

Fig. S240. $^{13}C\{^1H\}$ NMR (151 MHz, $CDCl_3$) of H-nateglinide methyl ester ([H]10.

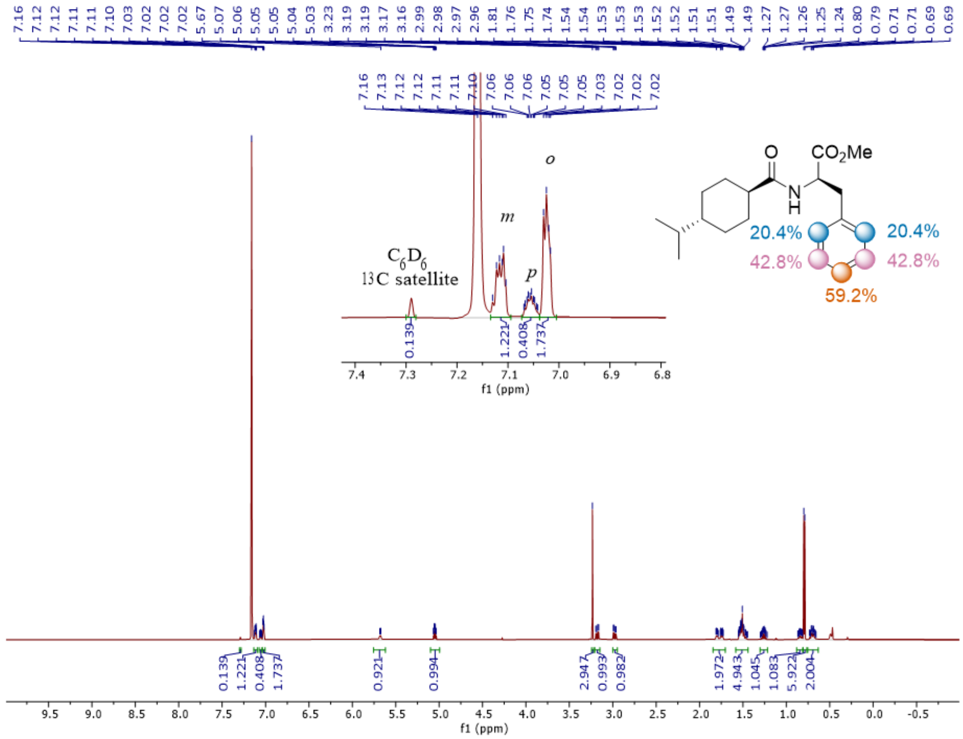

Fig. S241. ^{1}H NMR (600 MHz, d1=30s, C_6D_6) of D-nateglinide methyl ester ([D]10).

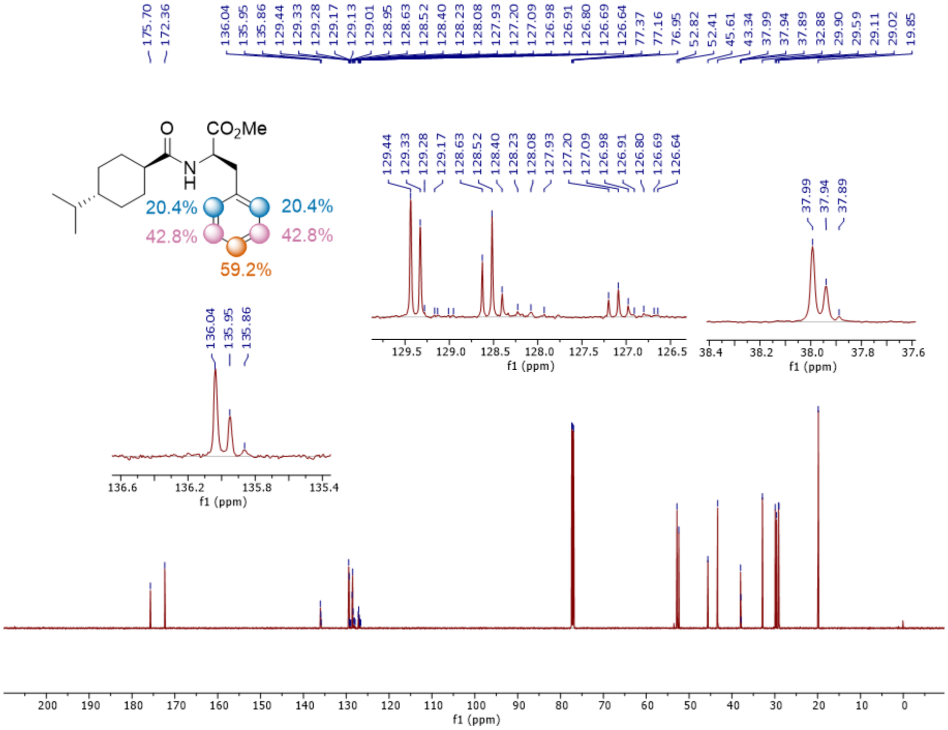

Fig. S242. $^{13}C\{^{1}H\}$ NMR (151 MHz, $CDCl_3$) of D-nateglinide methyl ester ([D]10).

Determining Deuterium Incorporation for D-nateglinide methyl ester:
Method 2.1: ^{1}H NMR, 600 MHz, d1=30s, C_6D_6

Ortho: correcting for ^{13}C satellite peak of benzene (0.139): $100 \times \left(1 - (\frac{1.737-0.139}{2H}\right) = \mathbf{\sim 20.1\%\ D}$

Meta: *inaccurate due to merging with incomplete separation with solvent peak.*

Para: $100 \times \left(1 - (\frac{0.408}{1H})\right) = \mathbf{59.2\%\ D}$

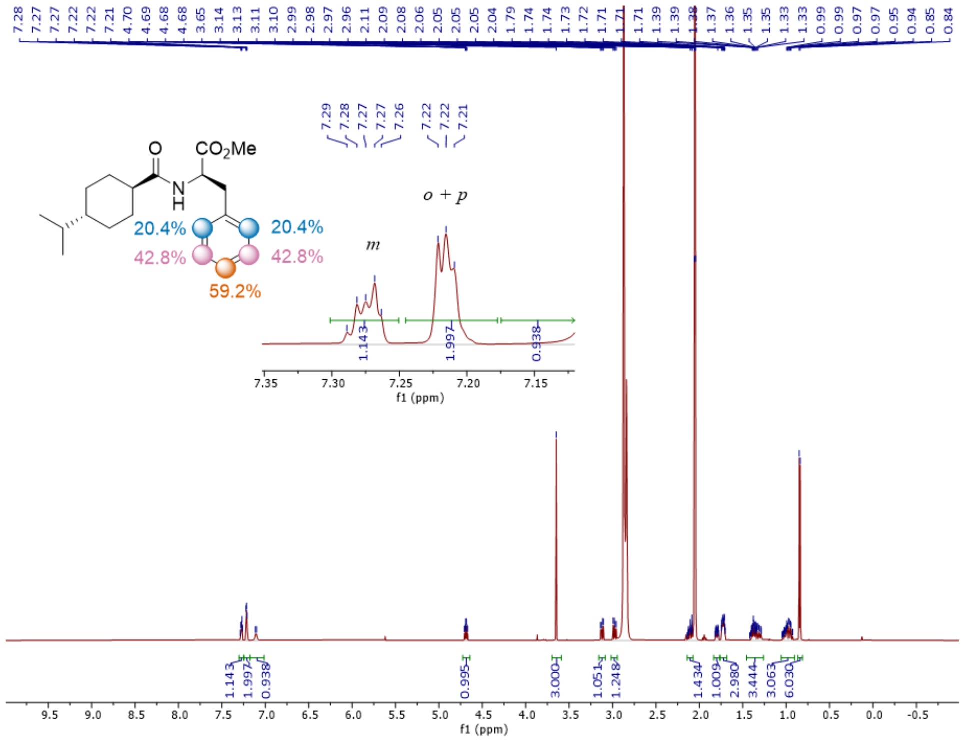

Fig. S243. **^{1}H NMR (600 MHz, acetone-d6) of D-nateglinide methyl ester ([D]10)**

Method 2.1: ^{1}H NMR, 600 MHz, d1=30s, acetone-d6
Ortho + Para (#H for para position determined from **^{1}H NMR (600 MHz, d1=30s, C_6D_6)**):

$$100 - \left(\frac{1.997 - 0.408\ H_{para}}{2\ H} \times 100\right) = \mathbf{20.5\%\ D\ (ortho)}$$

Meta: $100 \times \left(1 - (\frac{1.143}{2H}\right) = \mathbf{42.8\%\ D}$

Method 2.3: $^{13}C\{^1H\}$ NMR, 151 MHz, $CDCl_3$

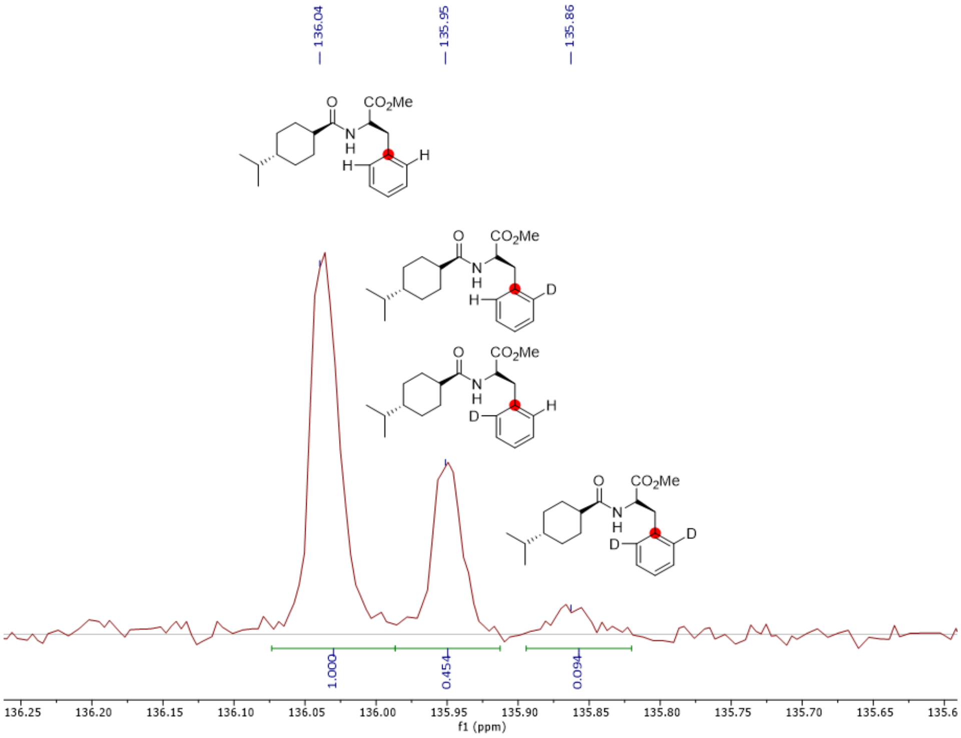

Fig. S244. **Zoomed in portion of $^{13}C\{^1H\}$ NMR of D-nateglinide methyl ester ([D]10) showing the ipso carbon of the aryl ring.**

$$100-\left[\frac{(1.000\times 2)+(0.454\times 1)}{(1.000\times 2)+(0.454\times 1)+(0.454\times 1)+(0.094\times 2)}\times 100\right]=\mathbf{20.7\%\ \mathit{D}}$$

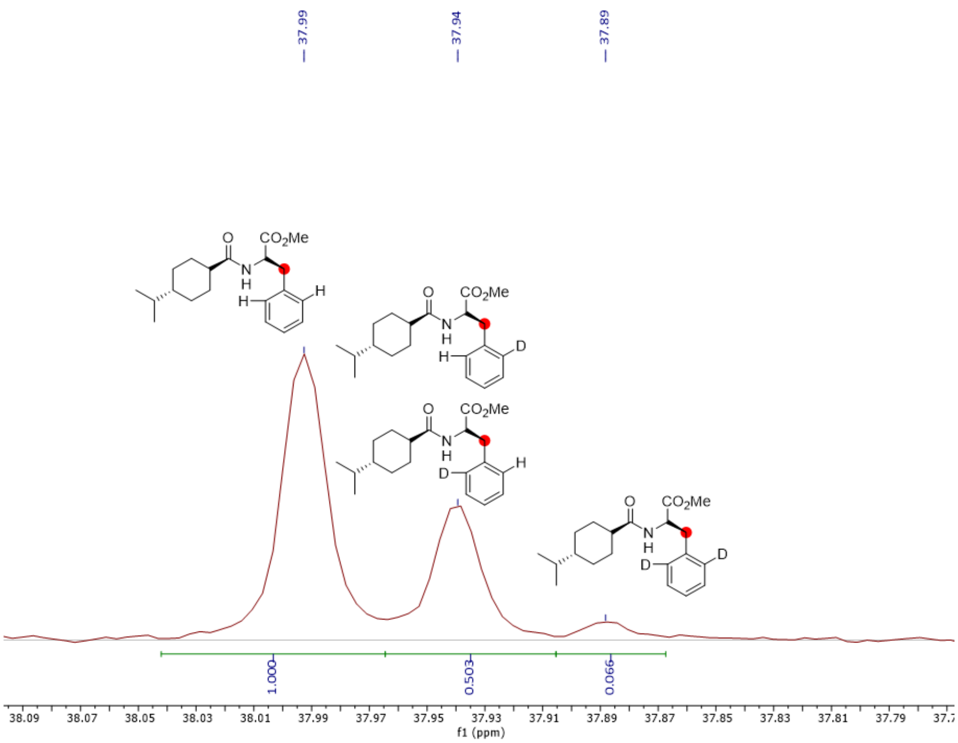

Fig. S245. Zoomed in portion of the $^{13}C\{^1H\}$ NMR of D-nateglinide methyl ester showing the benzylic carbon.

$$100 - \left[\frac{(1.000 \times 2) + (0.503 \times 1)}{(1.000 \times 2) + (0.503 \times 1) + (0.503 \times 1) + (0.066 \times 2)} \times 100\right] = \mathbf{20.2\%\ D}$$

Average %D at ortho position of D-nateglinide methyl ester.

$$\%D_{ortho} = \frac{20.1\% + 20.5\% + 20.7\% + 20.2\%}{4} = \mathbf{20.4\%\ D}$$

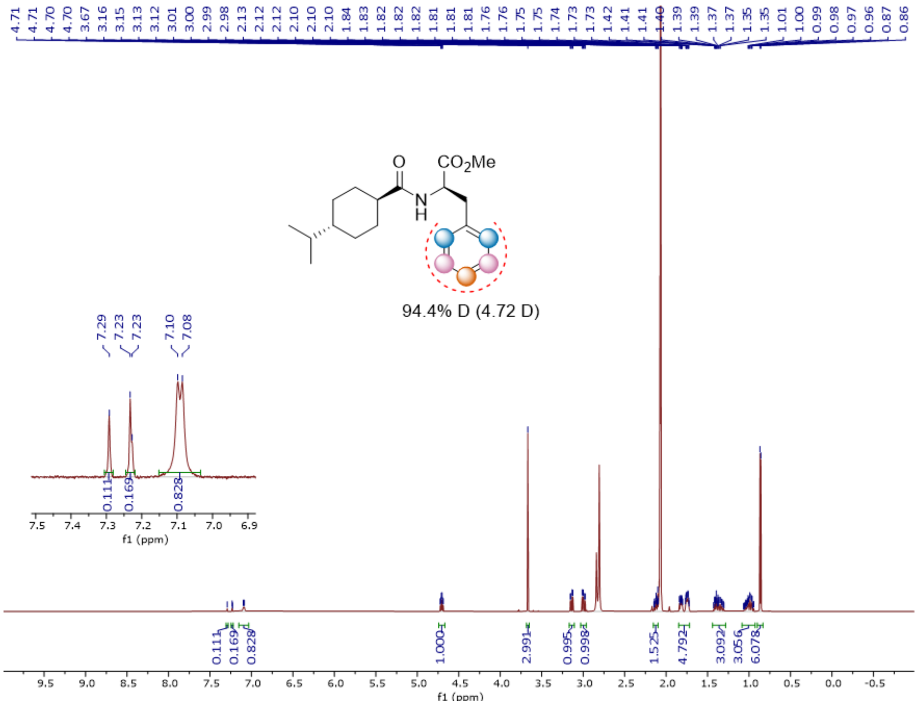

Fig. S246. ^{1}H NMR (600 MHz, Acetone) of d_5-Nateglinide methyl ester.

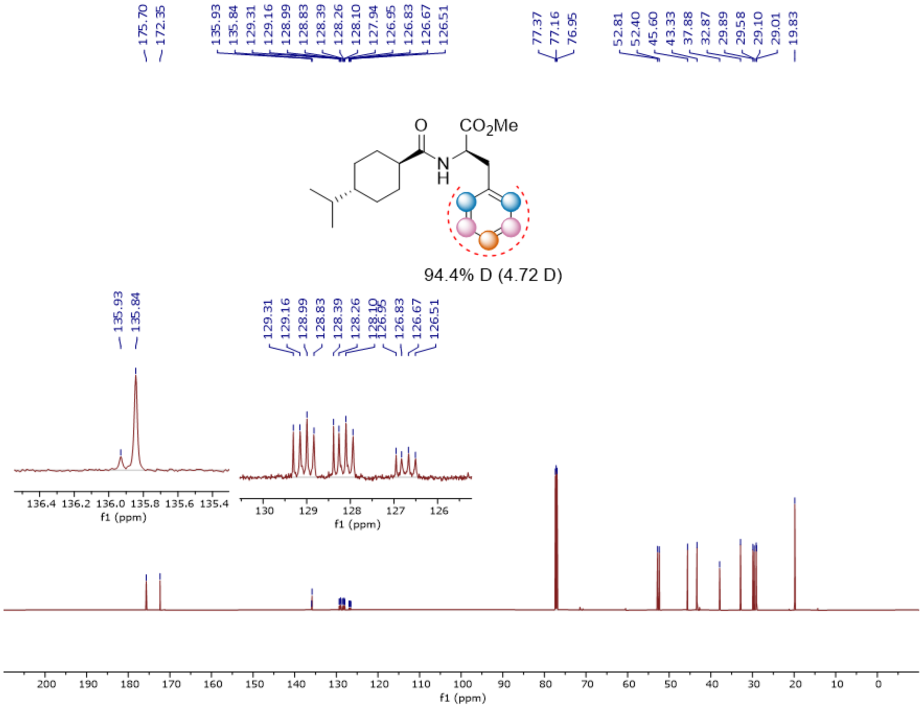

Fig. S247. $^{13}C\{^{1}H\}$ NMR (151 MHz, $CDCl_3$) of d_5-Nateglinide methyl ester

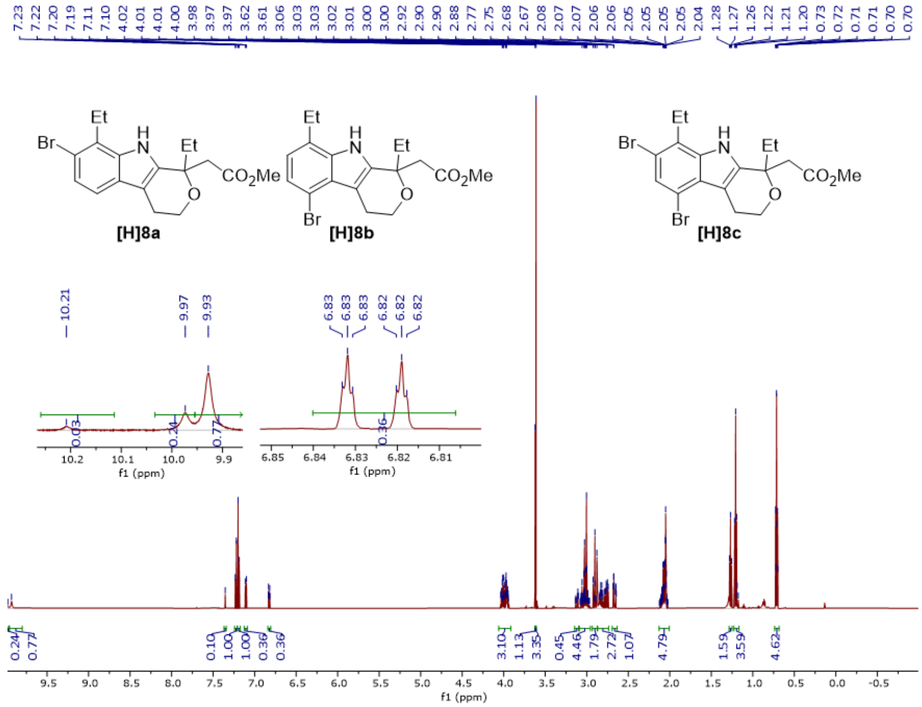

Fig. S248. ^{1}H NMR (600 MHz, Acetone) of [H]8a, [H]8b, [H]8c).

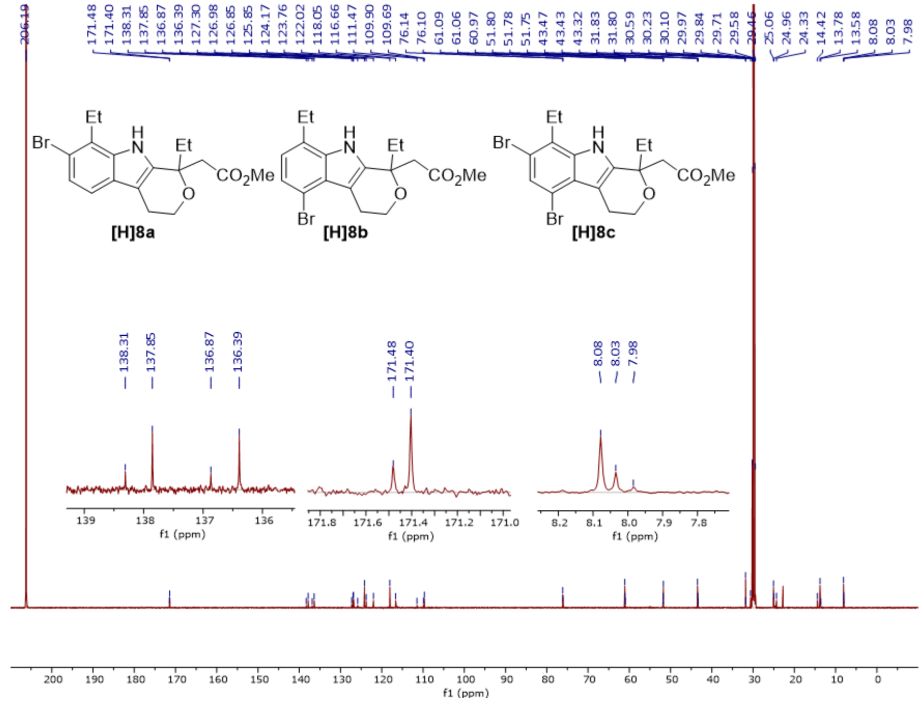

Fig. S249. $^{13}C\{^{1}H\}$ NMR (151 MHz, Acetone) of [H]8a, [H]8b, and [H]8c.

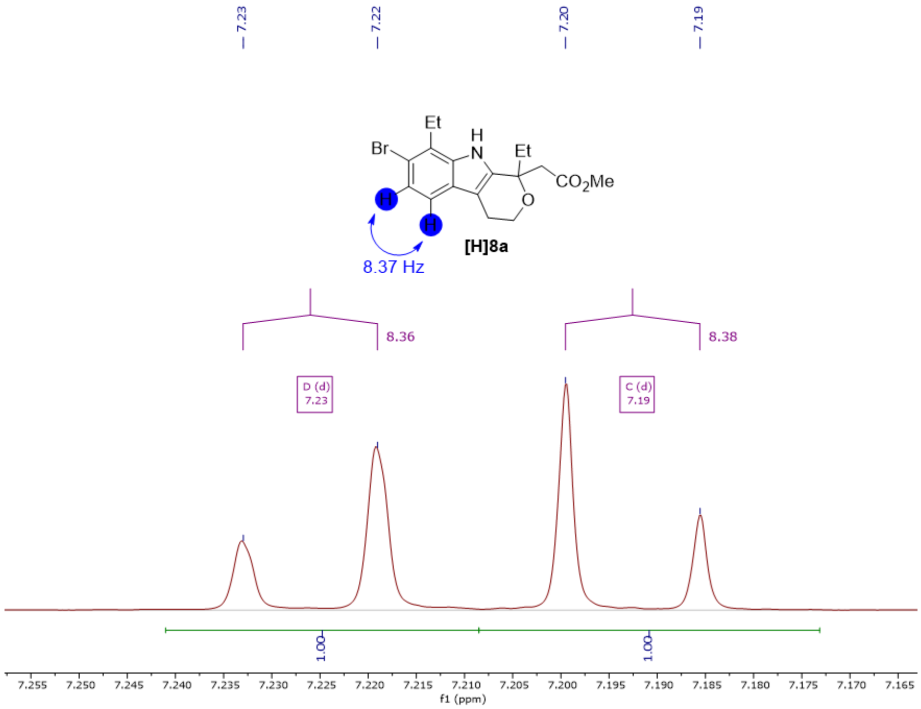

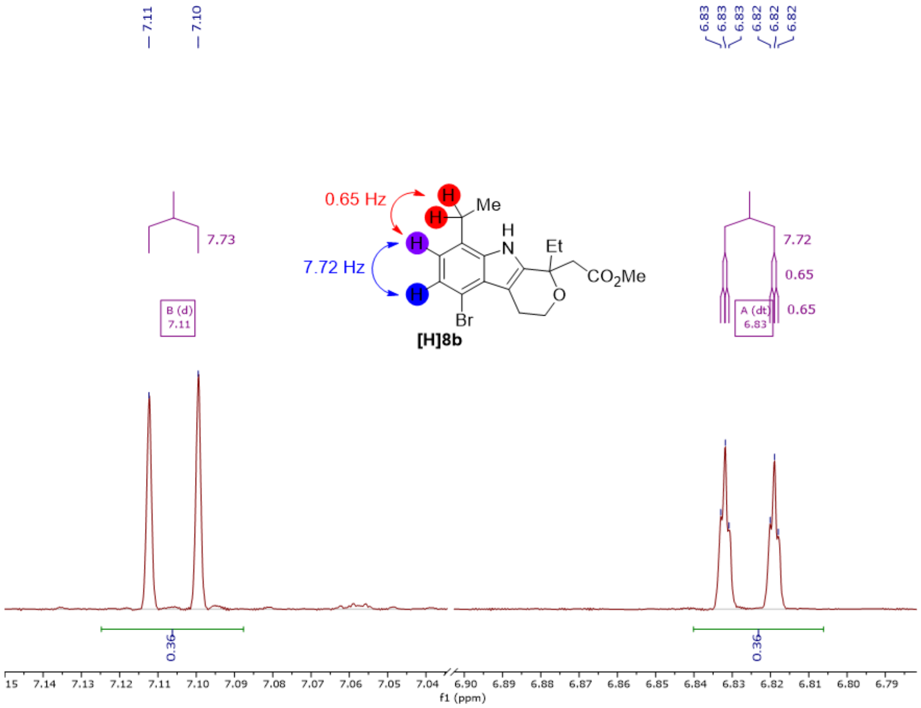

Fig. S250. Diagnostic peaks in ^{1}H NMR (600 MHz, Acetone) spectrum of [H]8a and [H]8b product mixture.

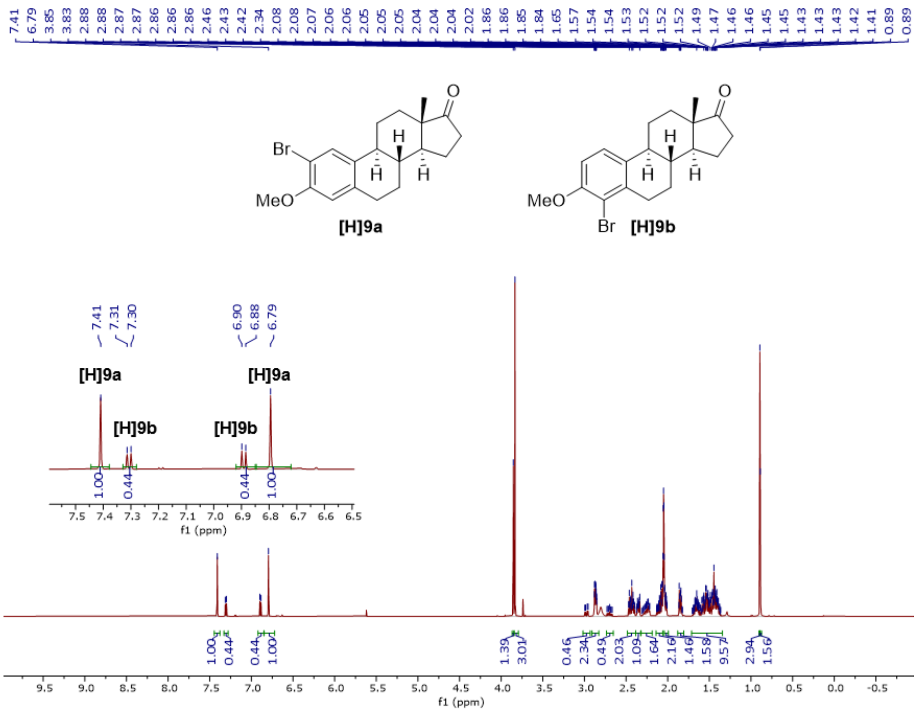

Fig. S251. ^{1}H NMR (600 MHz, Acetone) of product mixture of [H]9a and [H]9b.

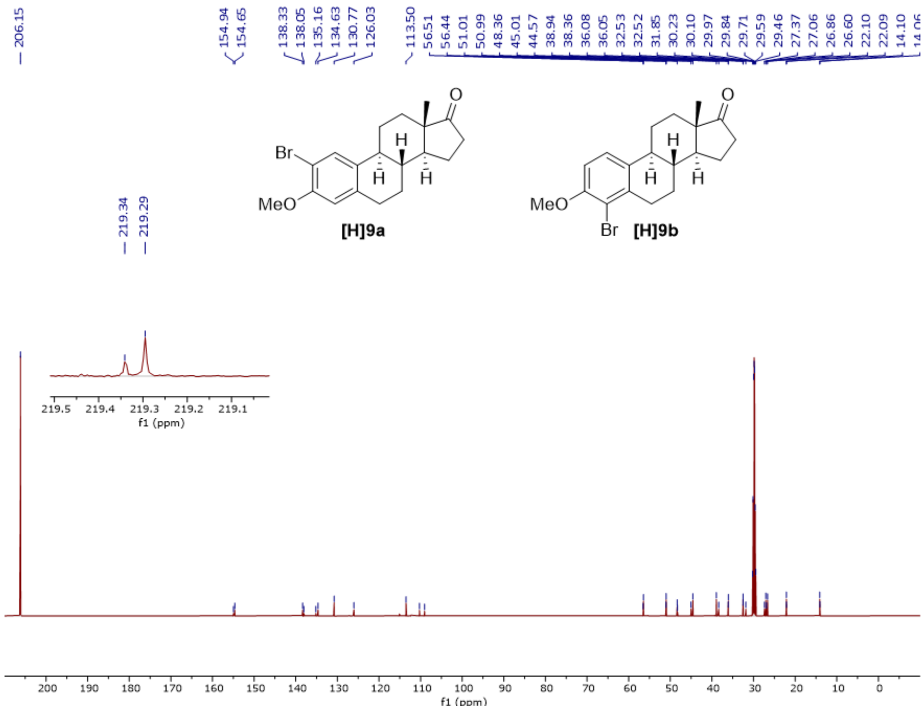

Fig. S252. $^{13}C\{^{1}H\}$ NMR (151 MHz, Acetone) of product mixture of [H]9a and [H]9b.

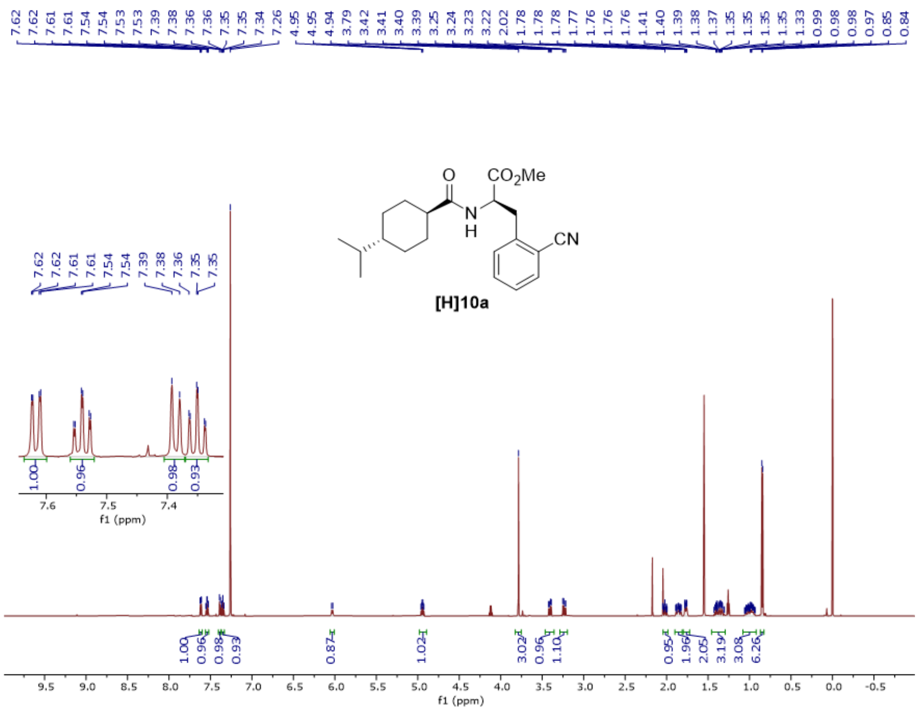

Fig. S253. ^{1}H NMR (600 MHz, $CDCl_3$) of [H]10a.

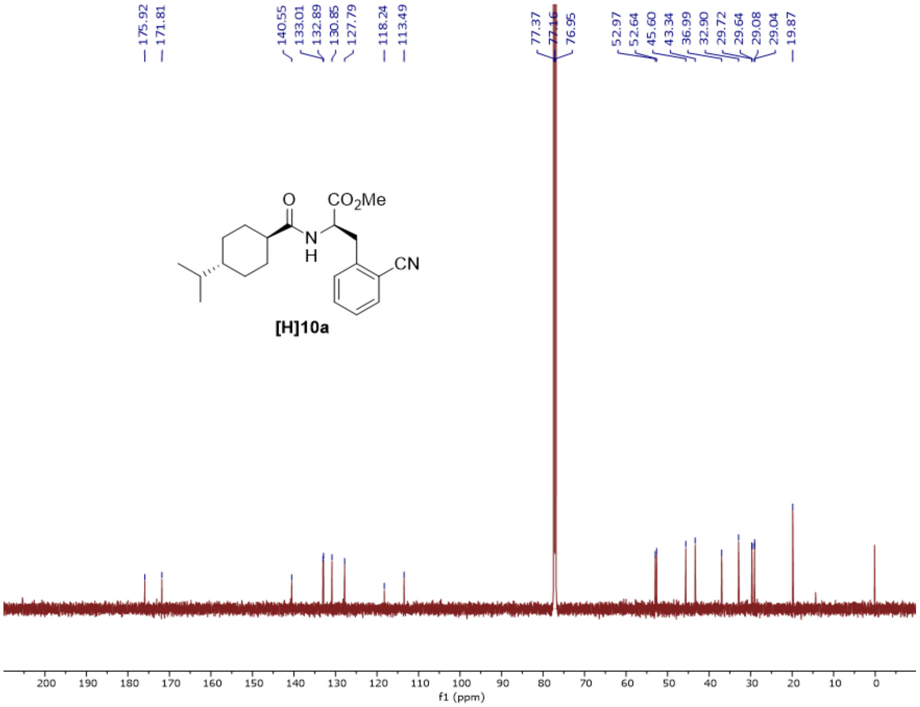

Fig. S254. $^{13}C\{^1H\}$ NMR (151 MHz, $CDCl_3$) of [H]10a.

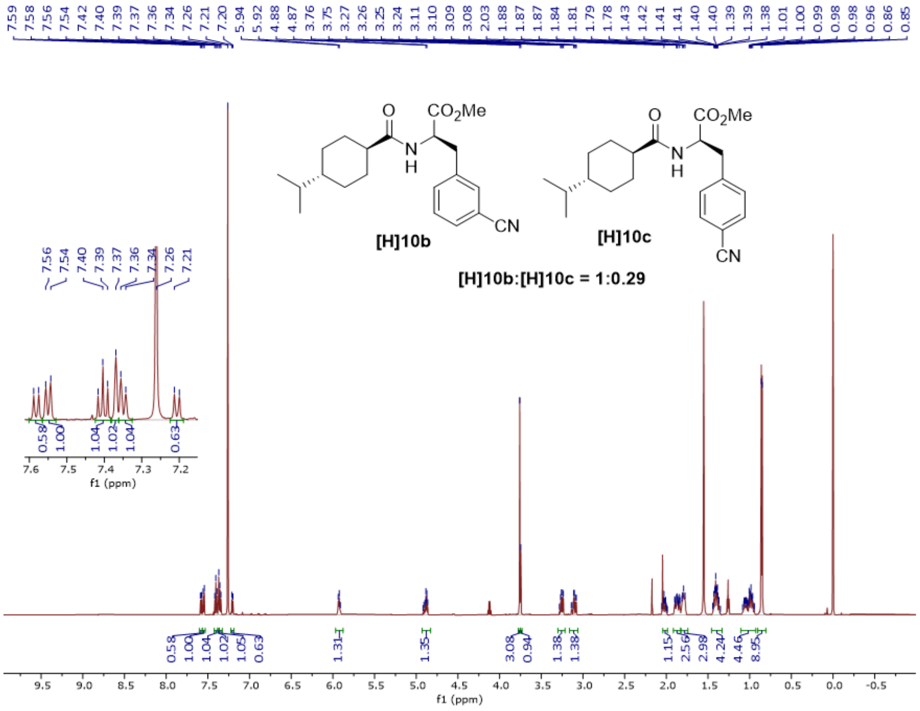

Fig. S255. ^{1}H NMR (600 MHz, $CDCl_3$) of [H]10b and [H]10c ([H]10b:[H]10c=1:0.29).

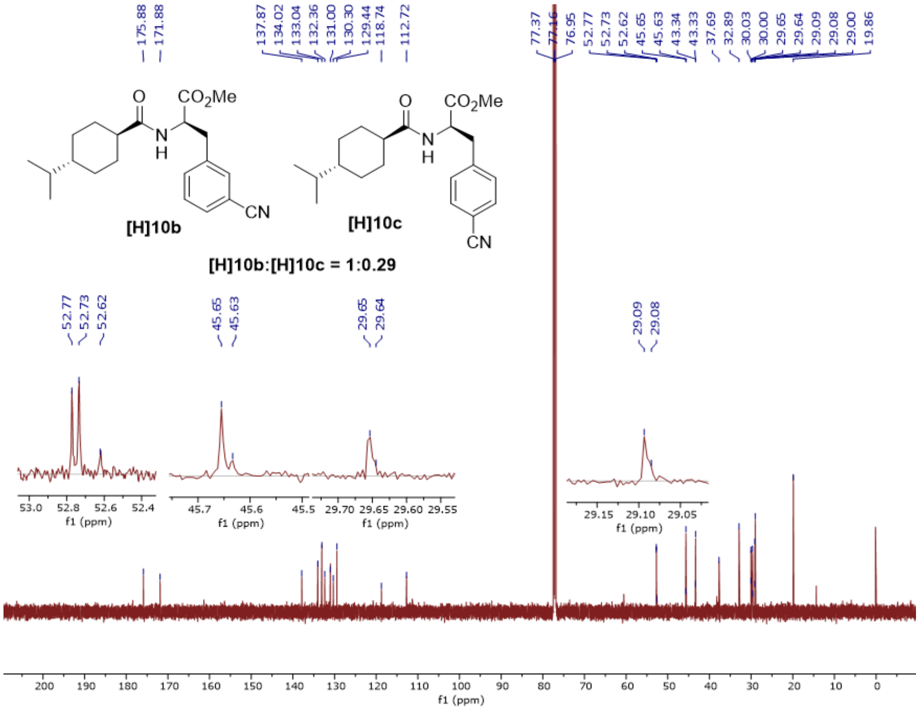

Fig. S256. $^{13}C\{^{1}H\}$ NMR (151 MHz, $CDCl_3$) of [H]10b and [H]10c ([H]10b:[H]10c=1:0.29).

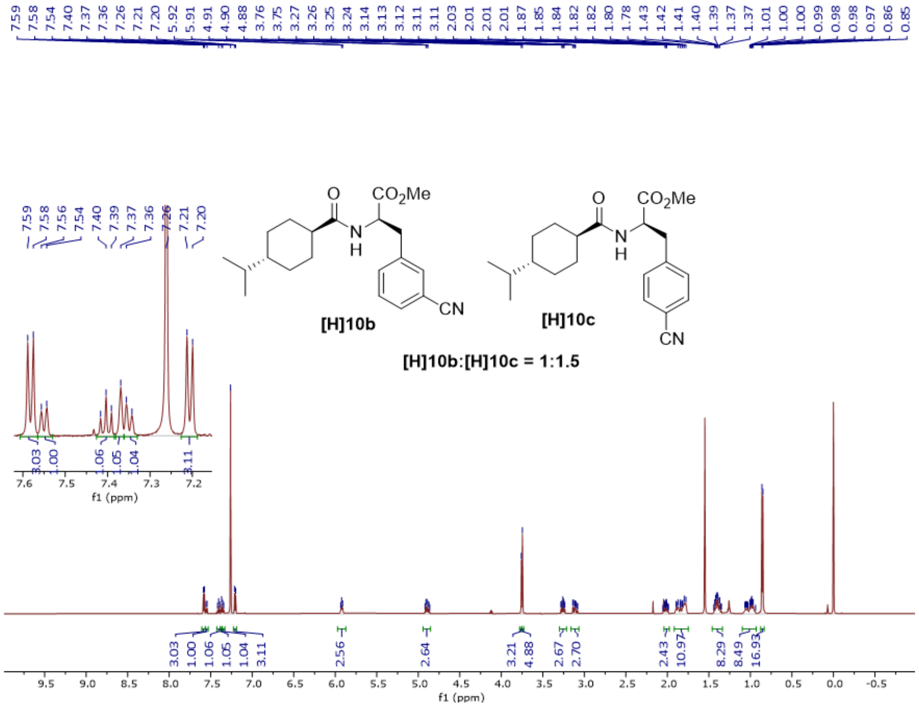

Fig. S257. ^{1}H NMR (600 MHz, $CDCl_3$) of [H]10b and [H]10c ([H]10b:[H]10c=1:1.5).

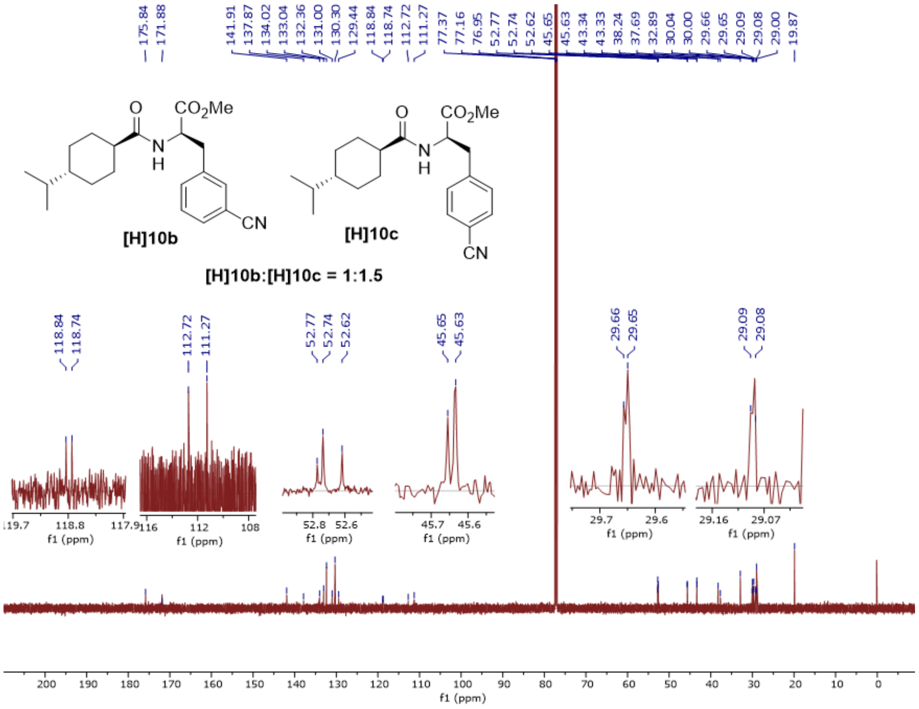

Fig. S258. $^{13}C\{^{1}H\}$ NMR (151 MHz, $CDCl_3$) of [H]10b and [H]10c ([H]10b:[H]10c=1:1.5).

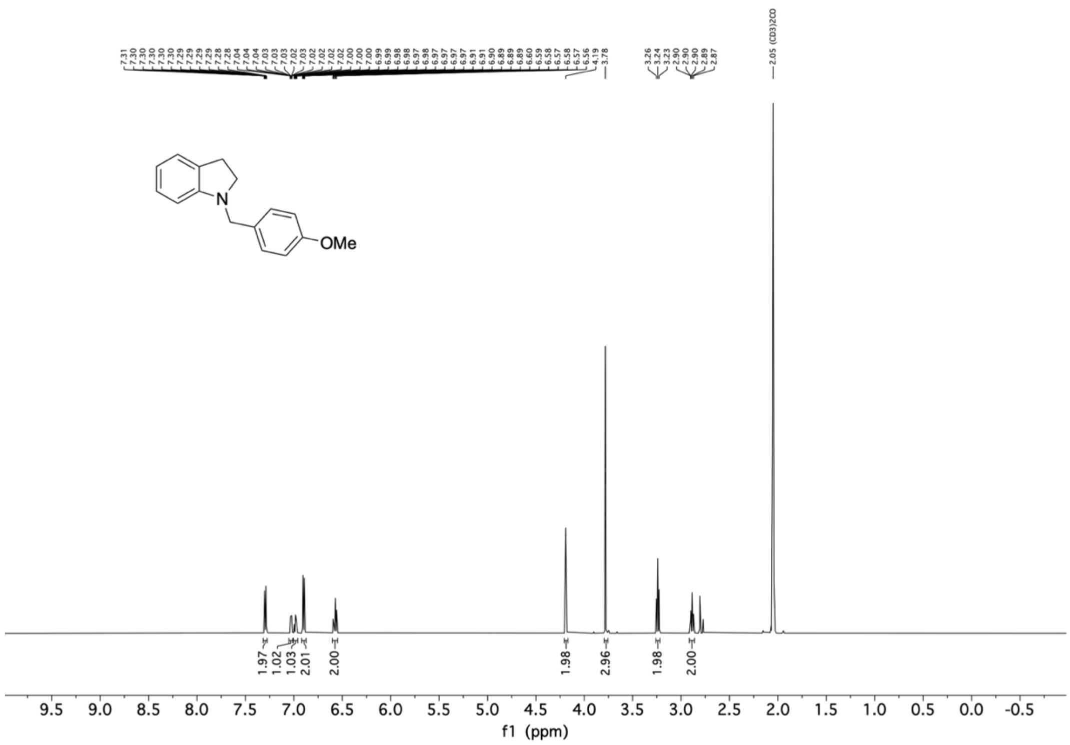

Fig. S259. ^{1}H **NMR (600 MHz, Acetone) of 1-(4-methoxybenzyl)indoline ([H]11).**

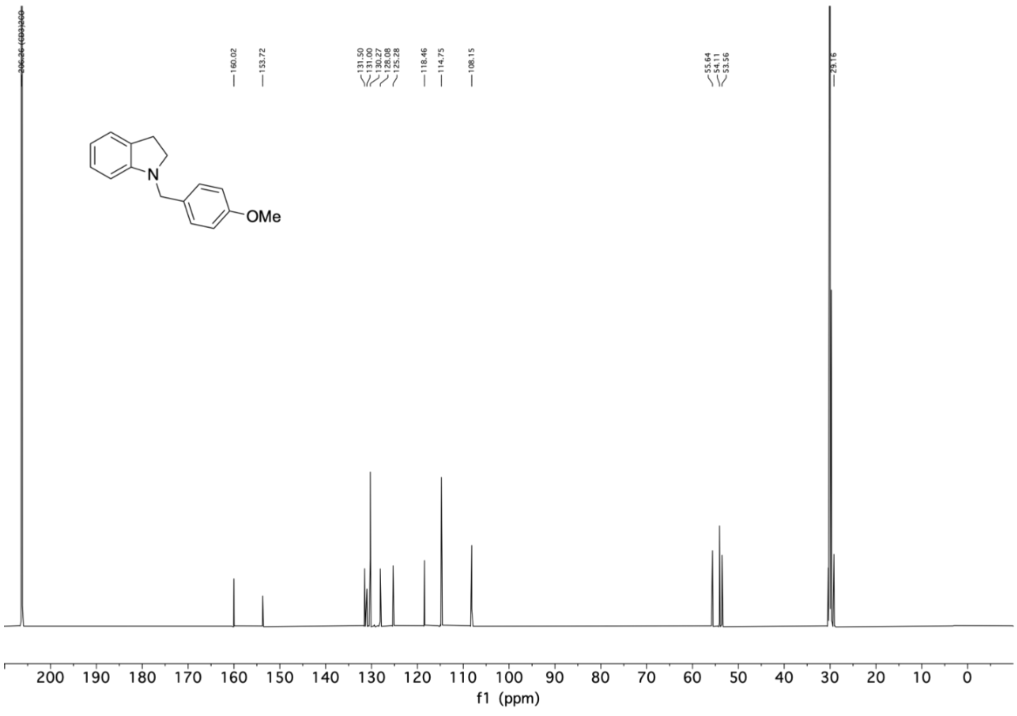

Fig. S260. $^{13}C\{^{1}H\}$ **NMR (600 MHz, Acetone) of 1-(4-methoxybenzyl)indoline ([H]11).**

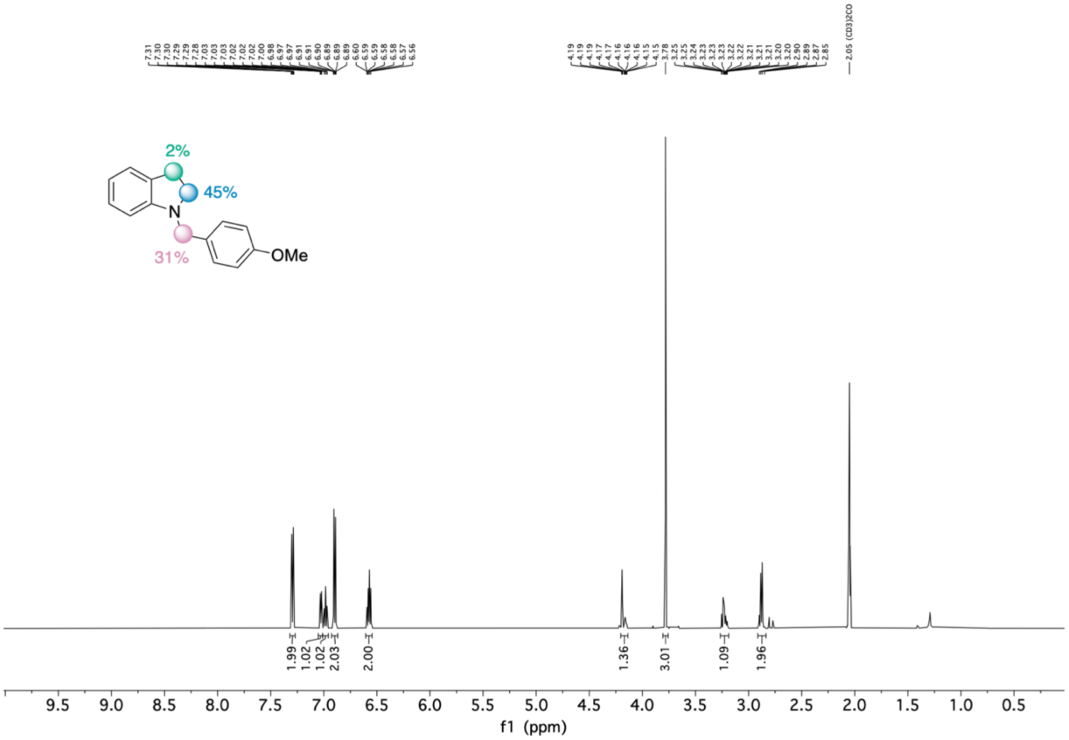

Fig. S261. **^{1}H NMR (600 MHz, Acetone) of D-1-(4-methoxybenzyl)indoline ([D]11).**

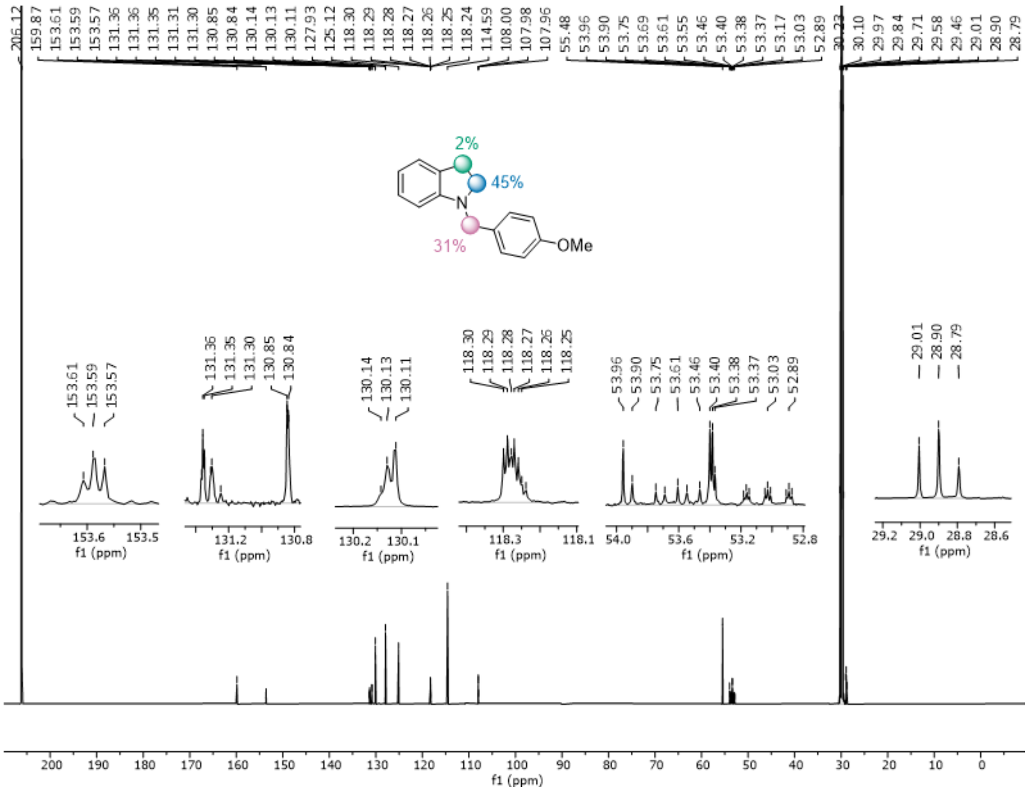

Fig. S262. **$^{13}C\{^{1}H\}$ NMR (600 MHz, Acetone) of D-1-(4-methoxybenzyl)indoline ([D]11).**

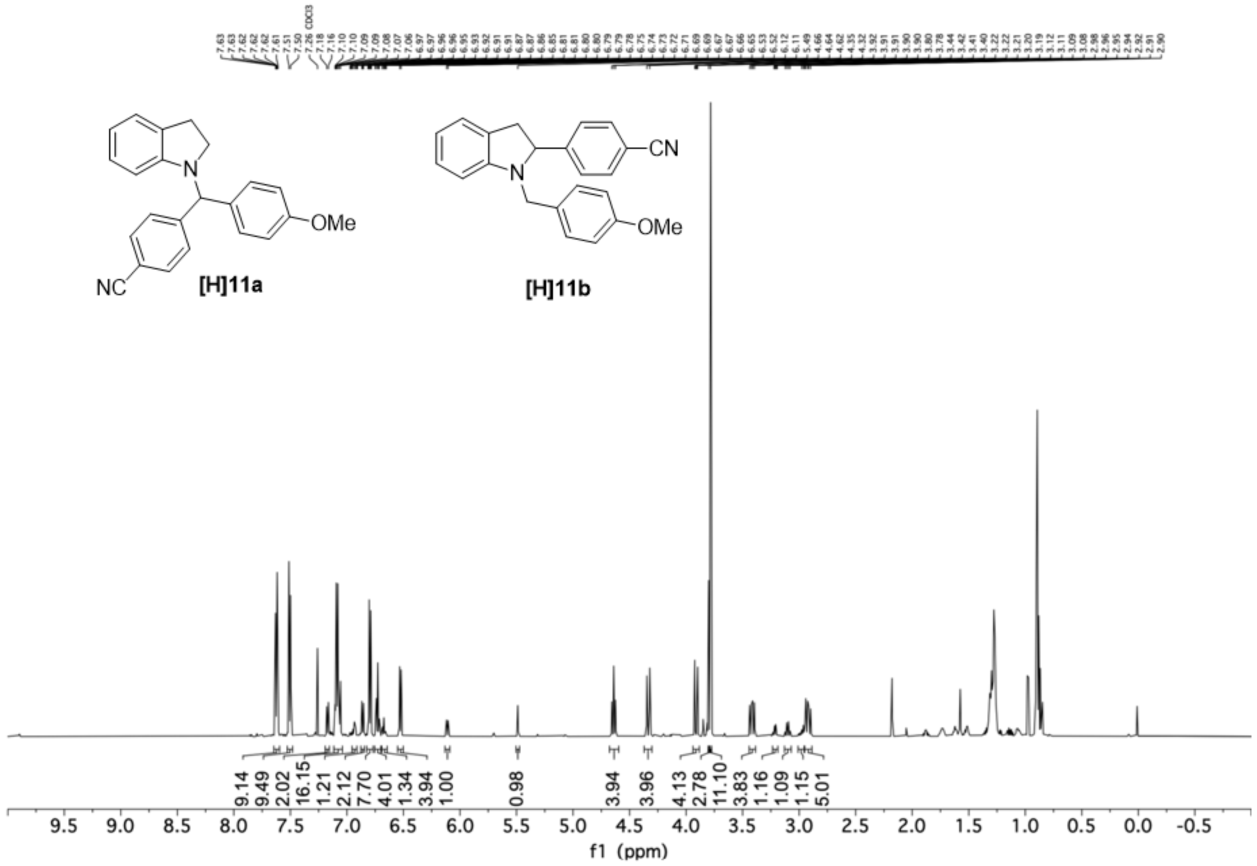

Fig. S263. ^{1}H NMR (600 MHz, Acetone) of [H]11a and [H]11b.

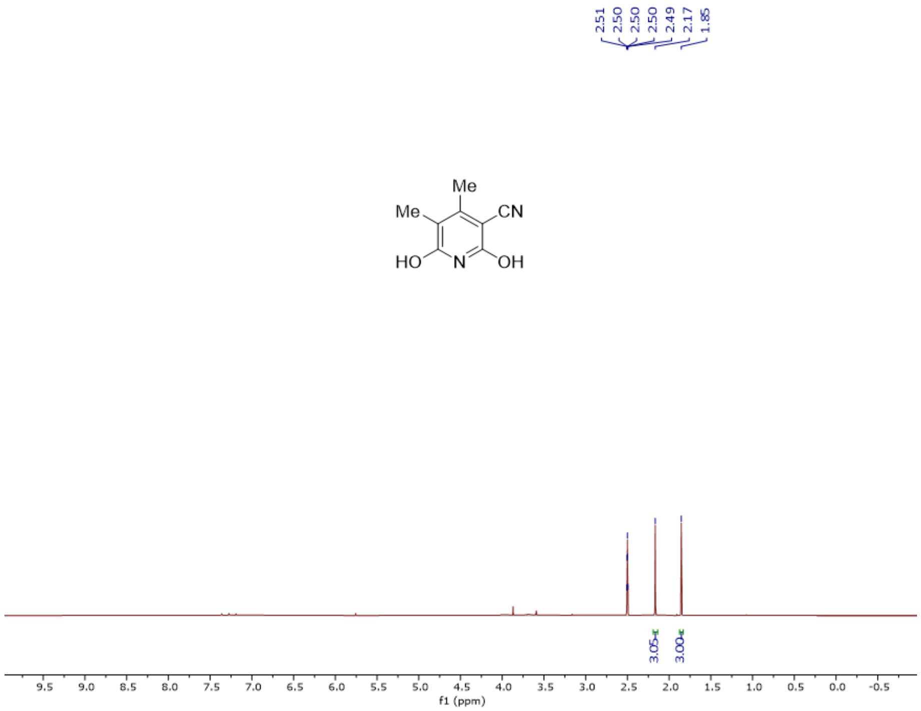

Fig. S264. **^{1}H NMR (600 MHz, DMSO) of 2,6-Dihydroxy-4,5-dimethylnicotinonitrile.**

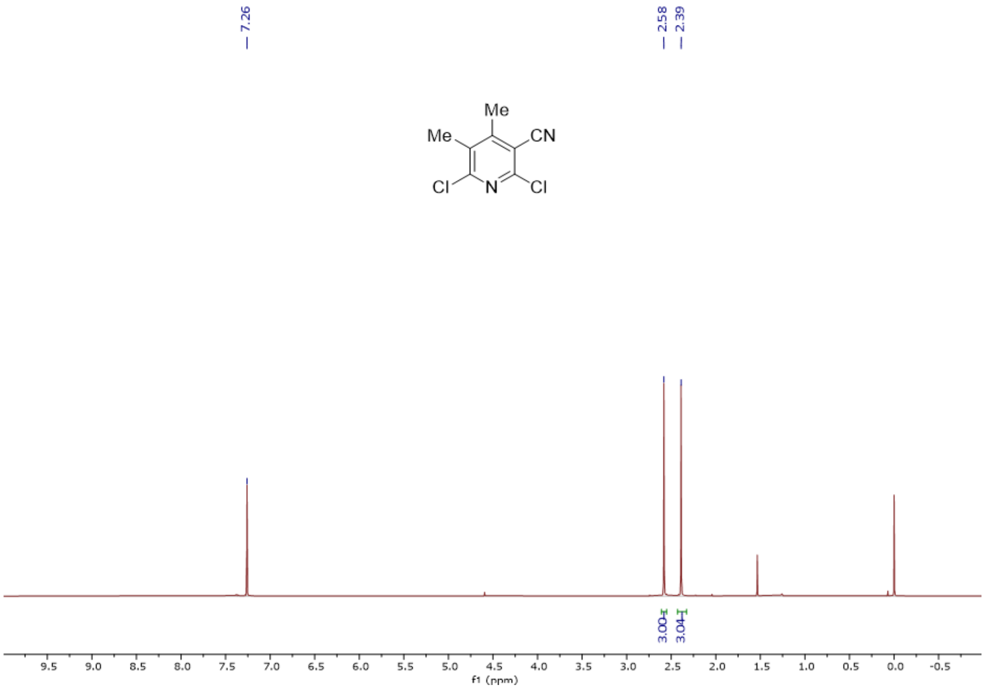

Fig. S265. **^{1}H NMR (400 MHz, $CDCl_3$) of 2,6-Dichloro-4,5-dimethylnicotinonitrile.**

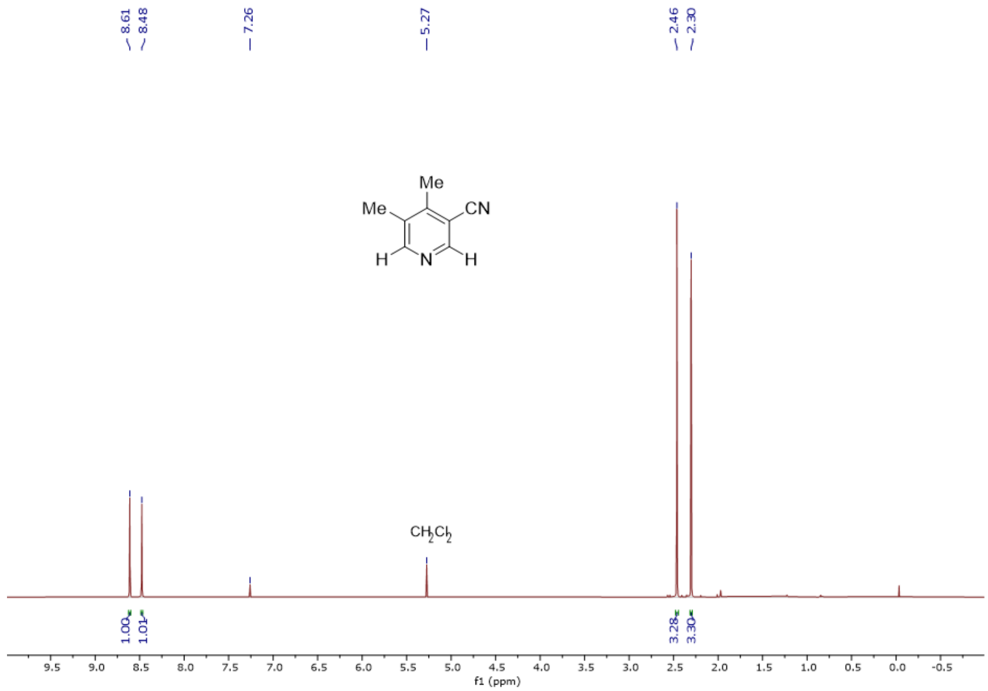

Fig. S266. ^{1}H NMR (600 MHz, $CDCl_3$) of 4,5-Dimethylnicotinonitrile ([H]12).

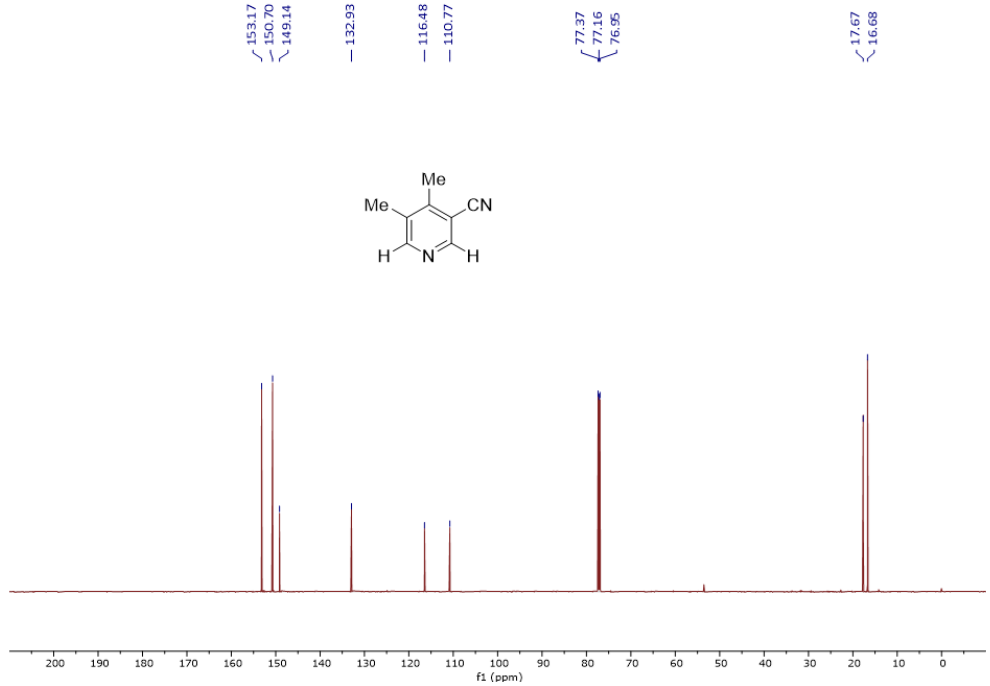

Fig. S267. $^{13}C\{^1H\}$ NMR (151 MHz, $CDCl_3$) of 4,5-Dimethylnicotinonitrile ([H]12).

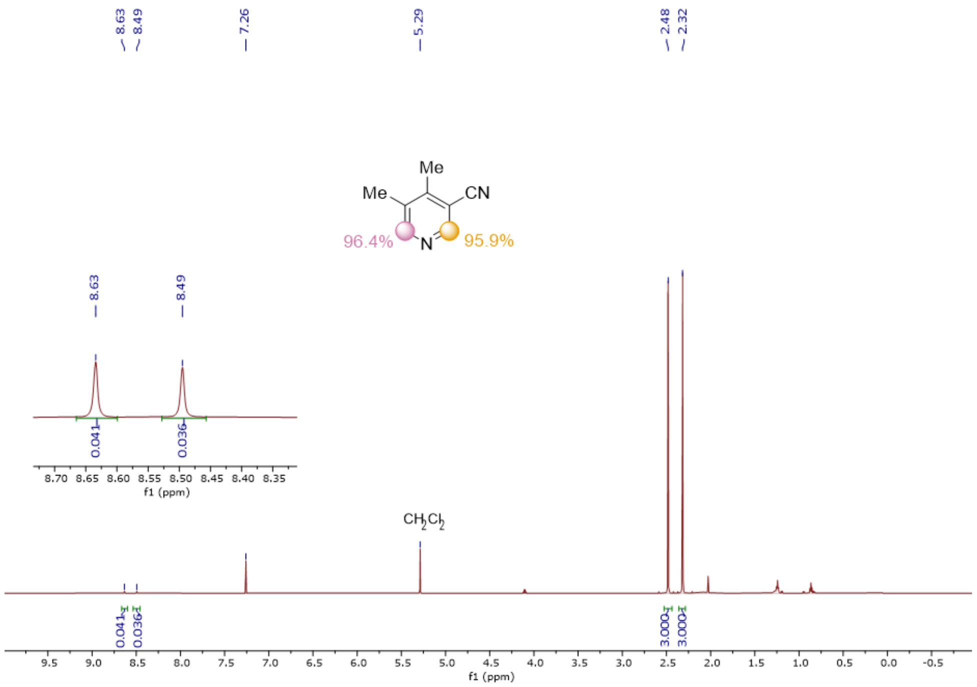

Fig. S268. ^{1}H NMR (600 MHz, $CDCl_3$) of 4,5-Dimethylnicotinonitrile-2,6-d_2 ([D]12).

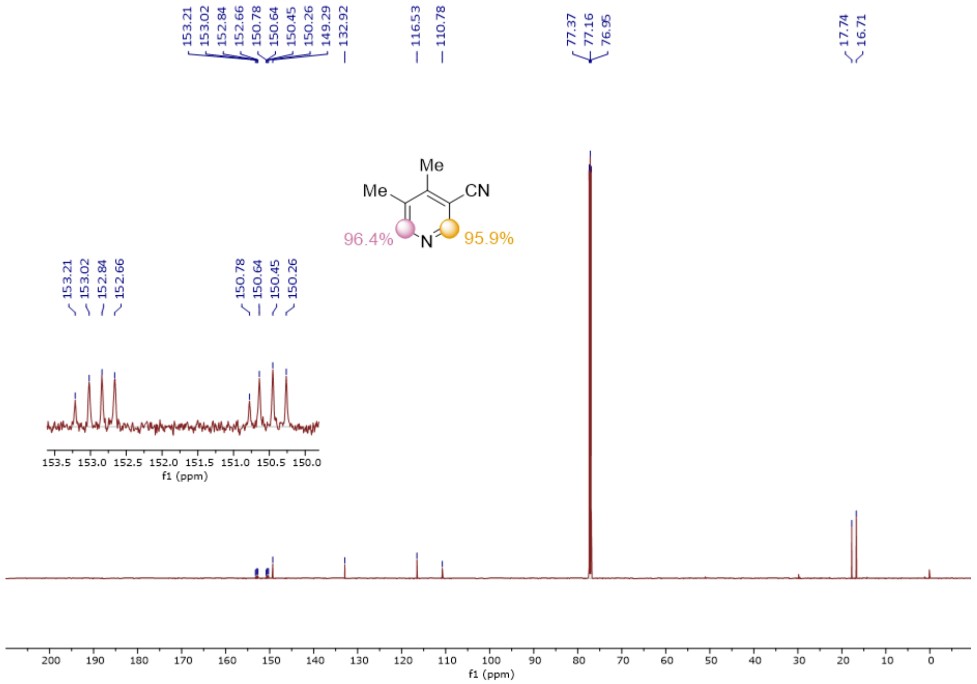

Fig. S269. $^{13}C\{^{1}H\}$ NMR (151 MHz, $CDCl_3$) of 4,5-Dimethylnicotinonitrile-2,6-d_2 ([D]12).

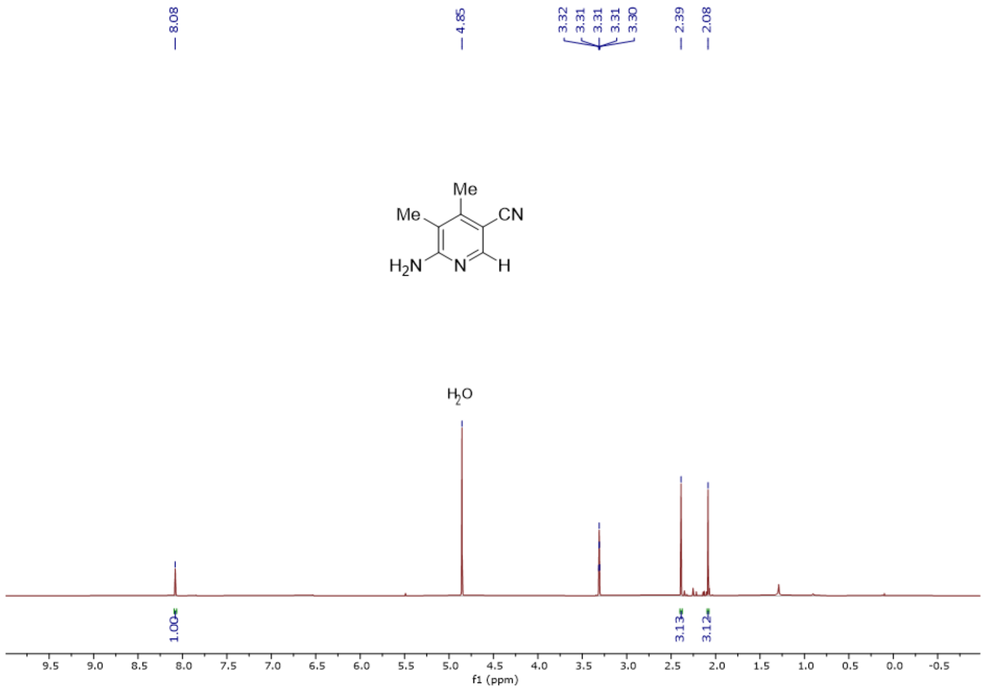

Fig. S270. ^{1}**H NMR (600 MHz, MeOD) of 6-Amino-4,5-dimethylnicotinonitrile.**

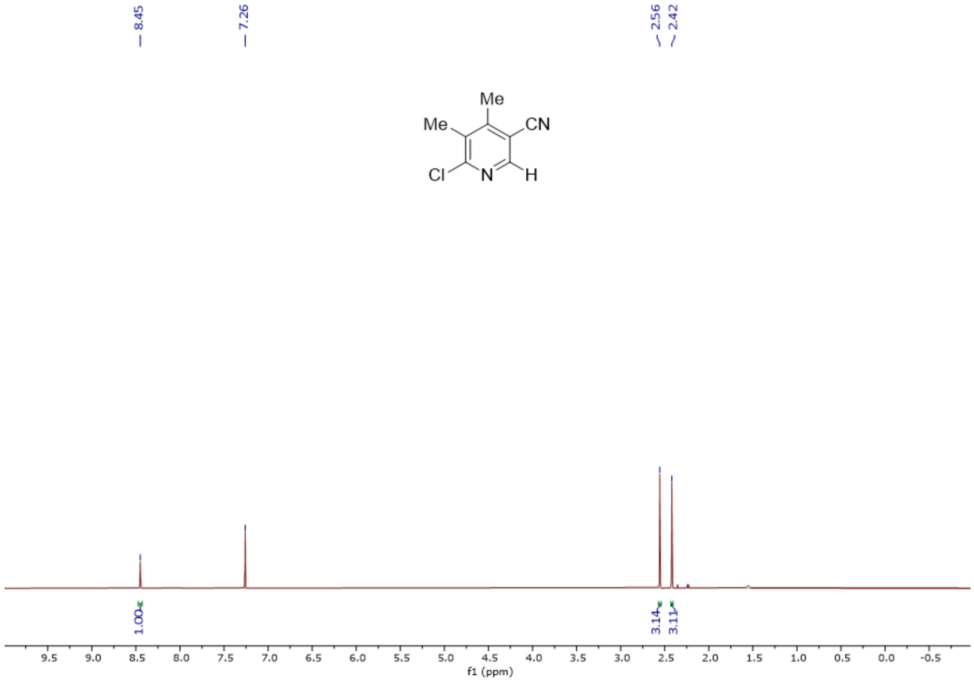

Fig. S271. ^{1}**H NMR (400 MHz, $CDCl_3$) of 6-Chloro-4,5-dimethylnicotinonitrile.**

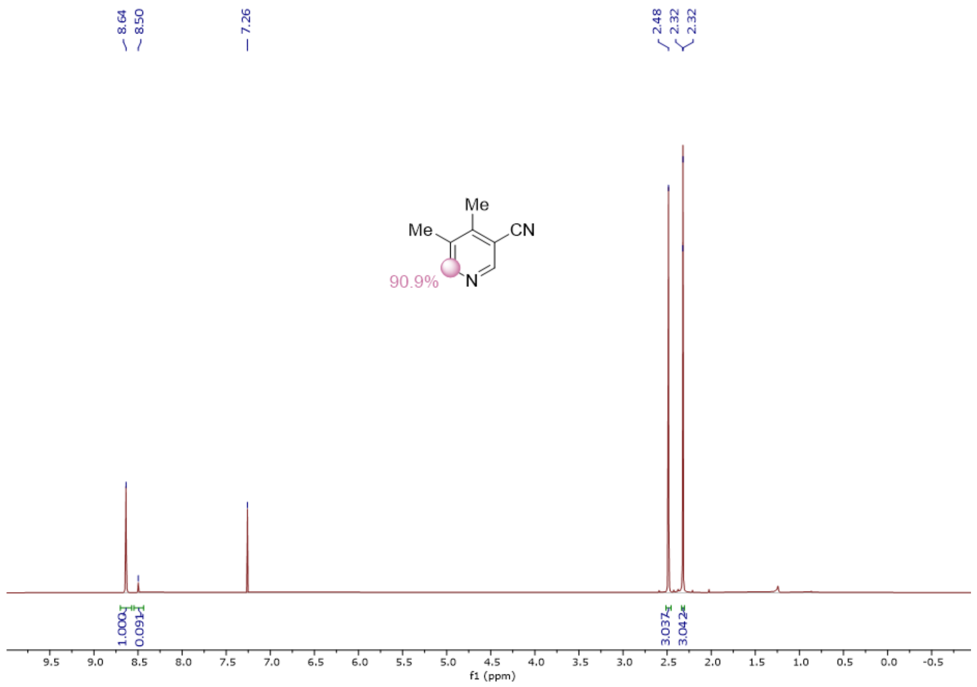

Fig. S272. **^{1}H NMR (600 MHz, $CDCl_3$) of 4,5-Dimethylnicotinonitrile-6-*d* ([D]12'.**

.

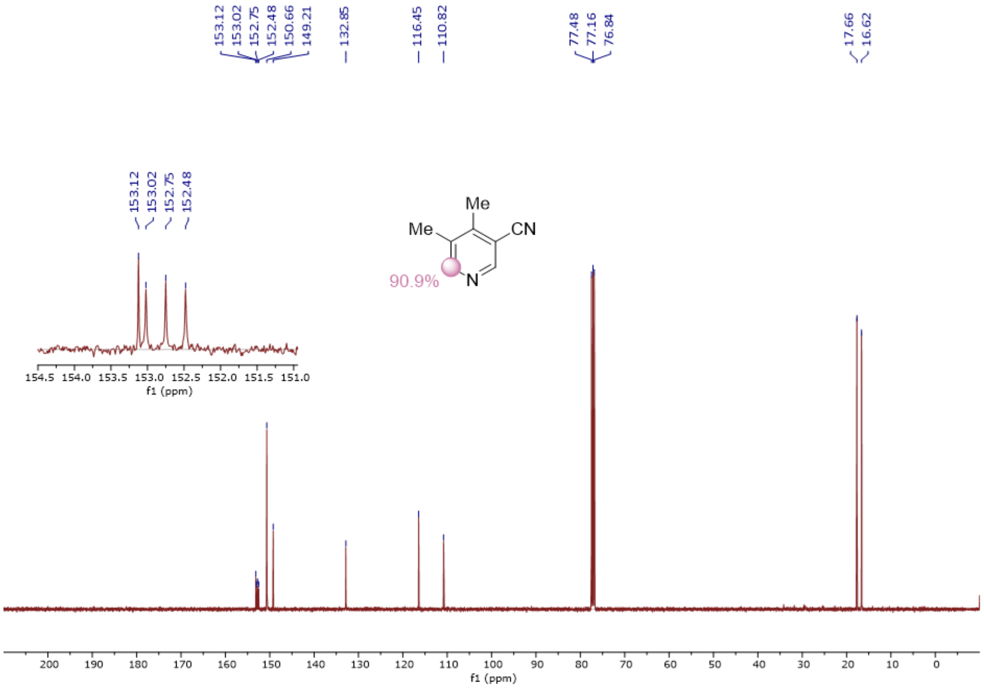

Fig. S273. **$^{13}C\{^1H\}$ NMR (151 MHz, $CDCl_3$) of 4,5-Dimethylnicotinonitrile-6-*d* ([D]12'.**

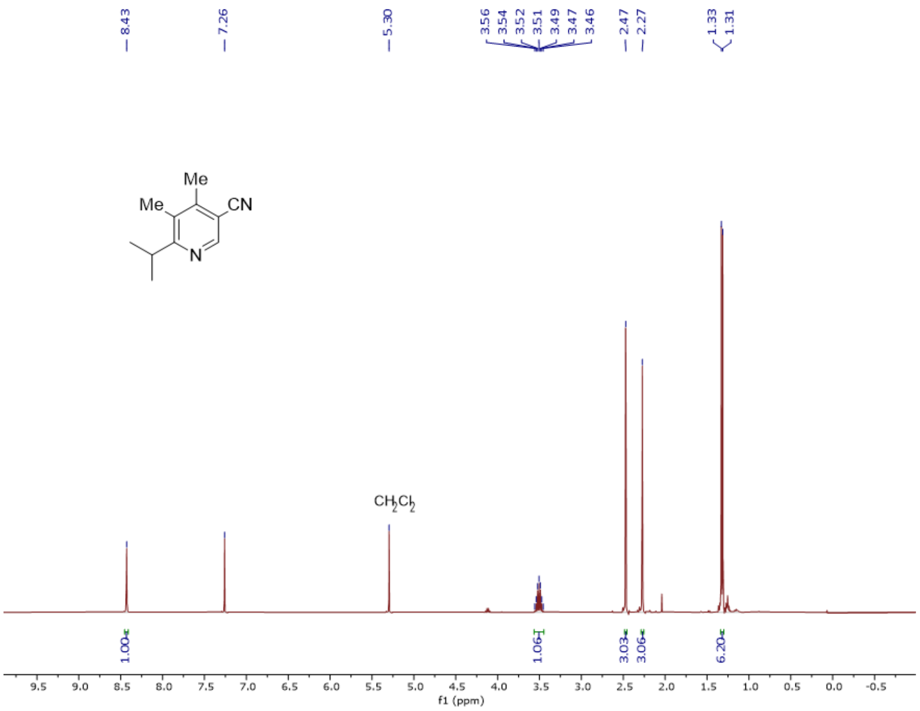

Fig. S274. ^{1}H NMR (400 MHz, $CDCl_3$) of [H]12a

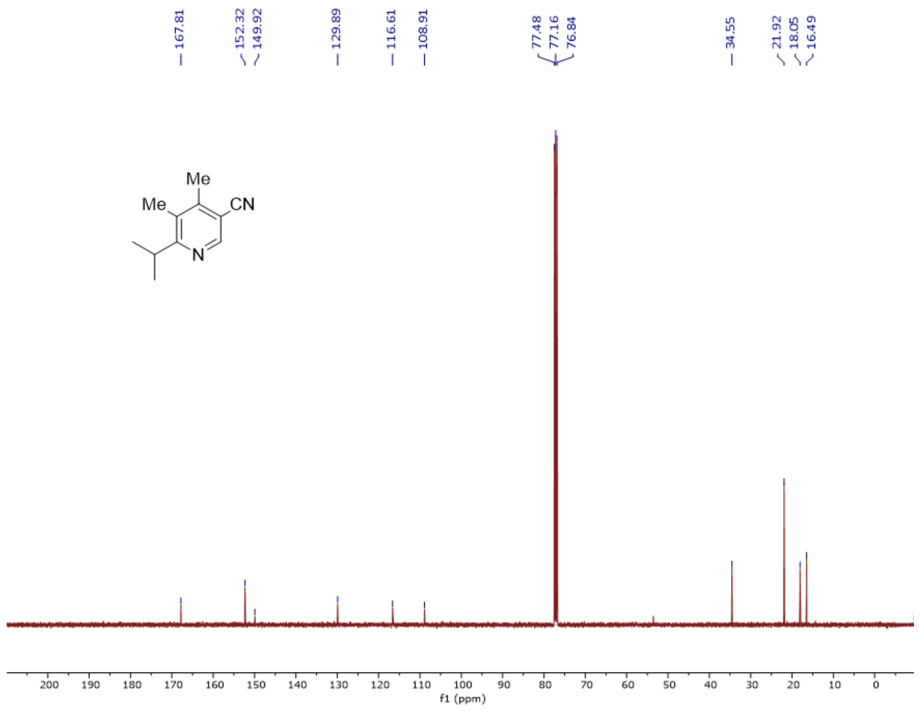

Fig. S275. $^{13}C\{^1H\}$ NMR (101 MHz, $CDCl_3$) of [H]12a

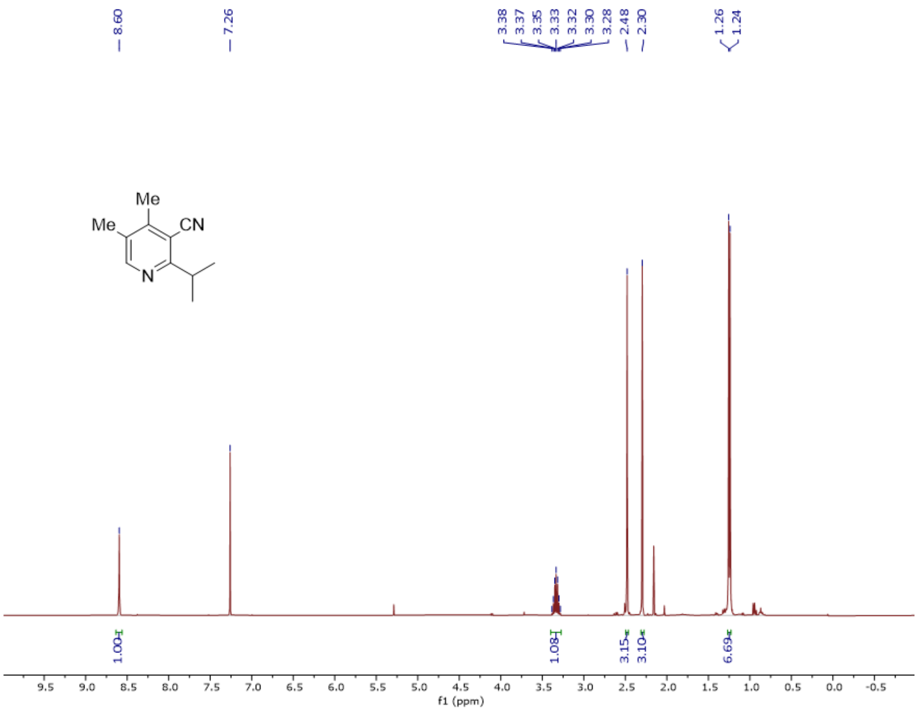

Fig. S276. ^{1}H NMR (400 MHz, $CDCl_3$) of [H]12b.

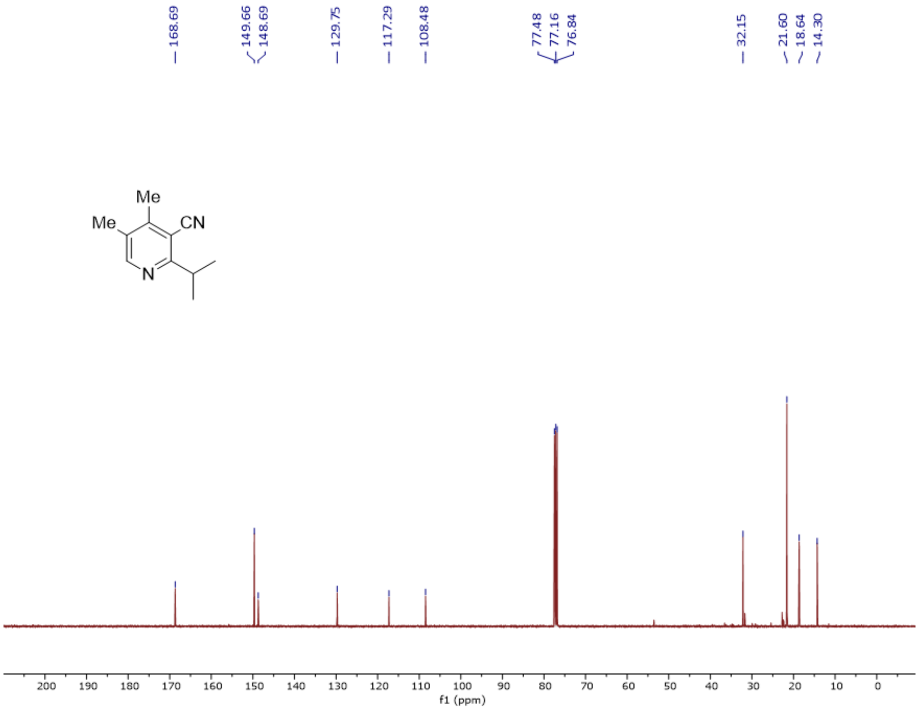

Fig. S277. $^{13}C\{^{1}H\}$ NMR (101 MHz, $CDCl_3$) of [H]12b.

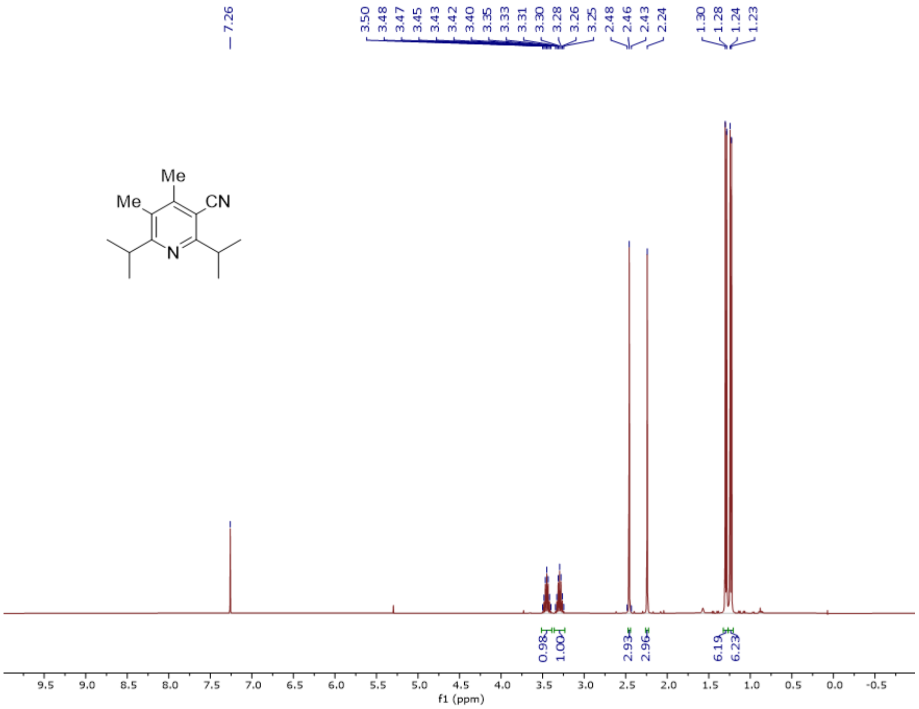

Fig. S278. ¹H NMR (400 MHz, $CDCl_3$) of [H]12c.

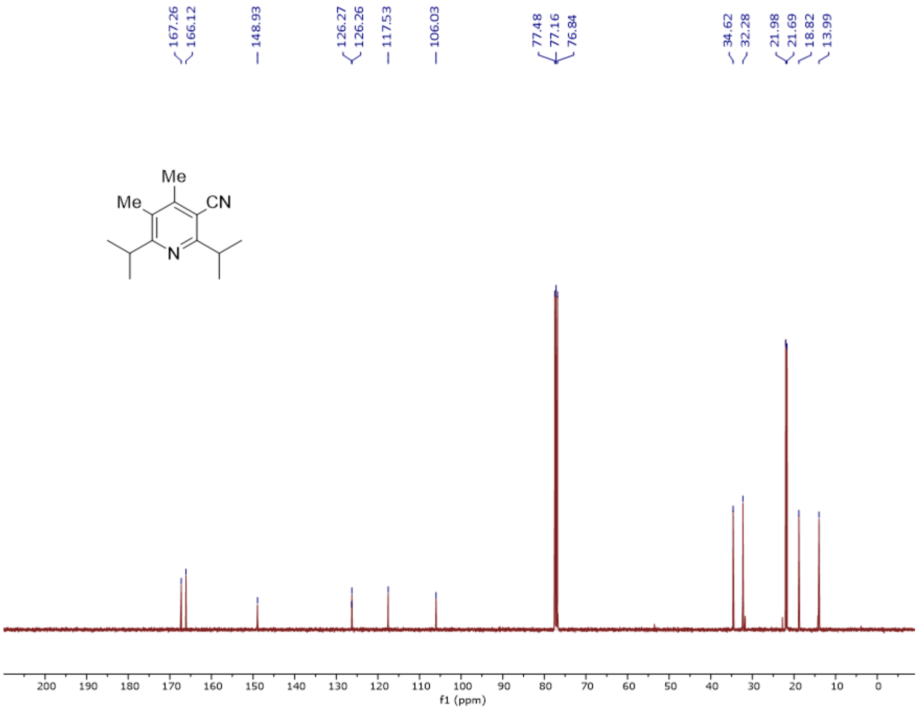

Fig. S279. $^{13}C\{^{1}H\}$ NMR (101 MHz, $CDCl_3$) of [H]12c.

References

1. Farizyan, M.; Mondal, A.; Mal, S.; Deufel, F.; van Gemmeren, M., Palladium-Catalyzed Nondirected Late-Stage C–H Deuteration of Arenes. *J. Am. Chem. Soc.* **2021,** *143* (40), 16370-16376, 10.1021/jacs.1c08233.
2. Loos, M.; Gerber, C.; Corona, F.; Hollender, J.; Singer, H., Accelerated Isotope Fine Structure Calculation Using Pruned Transition Trees. *Anal. Chem.* **2015,** *87* (11), 5738-5744, 10.1021/acs.analchem.5b00941.
3. Turowski, M.; Yamakawa, N.; Meller, J.; Kimata, K.; Ikegami, T.; Hosoya, K.; Tanaka, N.; Thornton, E. R., Deuterium Isotope Effects on Hydrophobic Interactions: The Importance of Dispersion Interactions in the Hydrophobic Phase. *J. Am. Chem. Soc.* **2003,** *125* (45), 13836-13849, 10.1021/ja036006g.
4. Oss, M.; Kruve, A.; Herodes, K.; Leito, I., Electrospray Ionization Efficiency Scale of Organic Compounds. *Anal. Chem.* **2010,** *82* (7), 2865-2872, 10.1021/ac902856t.
5. Martens, L.; Chambers, M.; Sturm, M.; Kessner, D.; Levander, F.; Shofstahl, J.; Tang, W. H.; Römpp, A.; Neumann, S.; Pizarro, A. D.; Montecchi-Palazzi, L.; Tasman, N.; Coleman, M.; Reisinger, F.; Souda, P.; Hermjakob, H.; Binz, P.-A.; Deutsch, E. W., mzML—a Community Standard for Mass Spectrometry Data*. *Molecular & Cellular Proteomics* **2011,** *10* (1), R110.000133, https://doi.org/10.1074/mcp.R110.000133.
6. Chambers, M. C.; Maclean, B.; Burke, R.; Amodei, D.; Ruderman, D. L.; Neumann, S.; Gatto, L.; Fischer, B.; Pratt, B.; Egertson, J.; Hoff, K.; Kessner, D.; Tasman, N.; Shulman, N.; Frewen, B.; Baker, T. A.; Brusniak, M.-Y.; Paulse, C.; Creasy, D.; Flashner, L.; Kani, K.; Moulding, C.; Seymour, S. L.; Nuwaysir, L. M.; Lefebvre, B.; Kuhlmann, F.; Roark, J.; Rainer, P.; Detlev, S.; Hemenway, T.; Huhmer, A.; Langridge, J.; Connolly, B.; Chadick, T.; Holly, K.; Eckels, J.; Deutsch, E. W.; Moritz, R. L.; Katz, J. E.; Agus, D. B.; MacCoss, M.; Tabb, D. L.; Mallick, P., A cross-platform toolkit for mass spectrometry and proteomics. *Nat. Biotechnol.* **2012,** *30* (10), 918-920, 10.1038/nbt.2377.
7. Kessner, D.; Chambers, M.; Burke, R.; Agus, D.; Mallick, P., ProteoWizard: open source software for rapid proteomics tools development. *Bioinformatics* **2008,** *24* (21), 2534-2536, 10.1093/bioinformatics/btn323.
8. DOI: 10.18129/B9.bioc.mzR.
9. DOI: 10.18129/B9.bioc.msdata
10. Gatto, L.; Lilley, K. S., MSnbase-an R/Bioconductor package for isobaric tagged mass spectrometry data visualization, processing and quantitation. *Bioinformatics* **2012,** *28* (2), 288-289, 10.1093/bioinformatics/btr645.
11. Gruber, C. C.; Oberdorfer, G.; Voss, C. V.; Kremsner, J. M.; Kappe, C. O.; Kroutil, W., An Algorithm for the Deconvolution of Mass Spectrosopic Patterns in Isotope Labeling Studies. Evaluation for the Hydrogen−Deuterium Exchange Reaction in Ketones. *J. Org. Chem.* **2007,** *72* (15), 5778-5783, 10.1021/jo070831o.
12. http://biocatalysis.uni-graz.at/pdf/IsoPat2.zip.
13. Tulloch, A. P., Deuterium isotope effects and assignment of 13C chemical shifts in spectra of methyl octadecanoate and the sixteen isomeric oxooctadecanoates. *Can. J. Chem.* **1977,** *55* (7), 1135-1142, 10.1139/v77-160.
14. Hansen, P. E.; Kamounah, F. S.; Gryko, D. T. Deuterium Isotope Effects on 13C-NMR Chemical Shifts of 10-Hydroxybenzo[h]quinolines *Molecules* [Online], 2013, p. 4544-4560.
15. Darwish, T. A.; Yepuri, N. R.; Holden, P. J.; James, M., Quantitative analysis of deuterium using the isotopic effect on quaternary 13C NMR chemical shifts. *Anal. Chim. Acta* **2016,** *927*, 89-98, https://doi.org/10.1016/j.aca.2016.05.003.
16. Pal, S.; Kumar, P.; Ramakrishna, E.; Kumar, S.; Porwal, K.; Kumar, B.; Arya, K. R.; Maurya, R.; Chattopadhyay, N., Extract and fraction of Cassia occidentalis L. (a synonym of Senna occidentalis) have

osteogenic effect and prevent glucocorticoid-induced osteopenia. *J. Ethnopharmacol.* **2019,** *235*, 8-18, https://doi.org/10.1016/j.jep.2019.01.029.

17. Kawabata, T.; Matsubara, H., Safe and facile evolution of diazomethane using the phase-vanishing method. *Tetrahedron Lett.* **2023,** *123*, 154554, https://doi.org/10.1016/j.tetlet.2023.154554.

18. Chouhan, K. K.; Chowdhury, D.; Mukherjee, A., Cyclotrimetaphosphate-assisted ruthenium catalyst for the hydration of nitriles and oxidation of primary amines to amides under aerobic conditions in water. *Organic & Biomolecular Chemistry* **2023,** *21* (11), 2429-2439, 10.1039/D3OB00062A.

19. Weinhold, T. D.; Reece, N. A.; Ribeiro, K.; Lopez Ocasio, M.; Watson, N.; Hanson, K.; Longstreet, A. R., Assessing Carbazole Derivatives as Single-Electron Photoreductants. *J. Org. Chem.* **2022,** *87* (24), 16928-16936, 10.1021/acs.joc.2c02312.

20. O'Brien, L.; Argent, S. P.; Ermanis, K.; Lam, H. W., Gold(I)-Catalyzed Nucleophilic Allylation of Azinium Ions with Allylboronates. *Angew. Chem. Int. Ed.* **2022,** *61* (22), e202202305, https://doi.org/10.1002/anie.202202305.

21. Joshi, A.; Kumari, S.; Kundu, S., Photoredox (NN)Mn(I) Catalysed Acceptorless Dehydrogenation: Synthesis of Amides, Aldehydes and Ketones. *Adv. Synth. Catal.* **2022,** *364* (24), 4371-4383, https://doi.org/10.1002/adsc.202201024.

22. Wu, H.; Sumita, A.; Otani, Y.; Ohwada, T., Friedel–Crafts Acylation of Aminocarboxylic Acids in Strong Brønsted Acid Promoted by Lewis Base P4O10. *J. Org. Chem.* **2022,** *87* (22), 15224-15249, 10.1021/acs.joc.2c01761.

23. Robinson, D. J.; Ortiz, K. G.; O'Hare, N. P.; Karimov, R. R., Dearomatization of Heteroarenium Salts with ArBpin Reagents. Application to the Total Synthesis of a Nuphar Alkaloid. *Org. Lett.* **2022,** *24* (19), 3445-3449, 10.1021/acs.orglett.2c00976.

24. Korch, K. M.; Hayes, J. C.; Kim, R. S.; Sampson, J.; Kelly, A. T.; Watson, D. A., Selected Ion Monitoring Using Low-Cost Mass Spectrum Detectors Provides a Rapid, General, and Accurate Method for Enantiomeric Excess Determination in High-Throughput Experimentation. *ACS Catalysis* **2022,** *12* (11), 6737-6745, 10.1021/acscatal.2c01628.

25. Zhao, D.; Xu, P.; Ritter, T., Palladium-Catalyzed Late-Stage Direct Arene Cyanation. *Chem* **2019,** *5* (1), 97-107, 10.1016/j.chempr.2018.09.027.

26. Fleury-Brégeot, N.; Raushel, J.; Sandrock, D. L.; Dreher, S. D.; Molander, G. A., Rapid and Efficient Access to Secondary Arylmethylamines. *Chem. Eur. J.* **2012,** *18* (31), 9564-9570, https://doi.org/10.1002/chem.201200831.

27. McNally, A.; Prier, C. K.; MacMillan, D. W. C., Discovery of an α-Amino C–H Arylation Reaction Using the Strategy of Accelerated Serendipity. *Science* **2011,** *334* (6059), 1114-1117, 10.1126/science.1213920.

28. CLAUSEN Dane James; FELLS, J. K., Joseph; LIU, Ping; MAZZOLA, Robert AZABICYCLO[4.1.0]HEPTANE ALLOSTERIC MODULATORS OF THE M4 MUSCARINIC ACETYLCHOLINE RECEPTOR. December 13, 2018.
