# Supplementary material for: Direct (LC-)MS Identification of Regioisomers from C–H Functionalization by Partial Isotopic Labeling
Source: ACS Cent Sci. 2025 Feb 14;11(2):272–8. doi: 10.1021/acscentsci.4c01765 (PMC11868960; doi:10.1021/acscentsci.4c01765)
Supplement: Supplementary file 3 — oc4c01765_si_003.zip [file oc4c01765_si_003.zip › Spectra Raw Files/NMR_SPECTRA.zip!/NMR_SPECTRA/[D]1_13C/5/pdata/1/email_CS-III-150-1-prod-C_5_1.pdf]

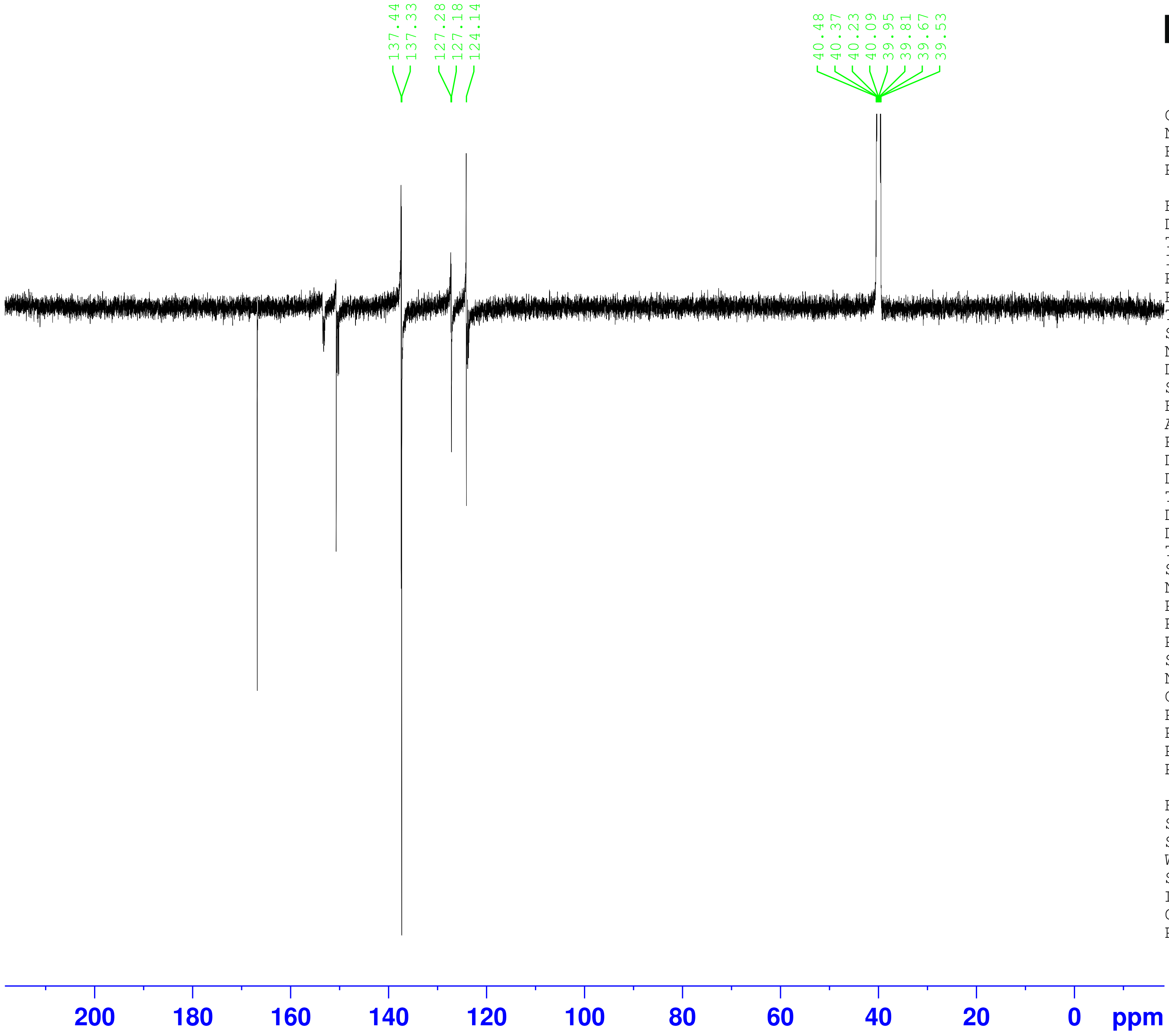

BRUKER

```
Current Data Parameters
NAME      CS-III-150-1-prod-C
EXPNO                       5
PROCNO                      1

F2 - Acquisition Parameters
Date_              20230125
Time                   0.25 h
INSTRUM   Avance 4 NEO 600
PROBHD    Z168773_0007 (
PULPROG            zgpg30
TD                  65536
SOLVENT              DMSO
NS                    256
DS                      4
SWH             35714.285 Hz
FIDRES           1.089913 Hz
AQ              0.9175040 sec
RG                    101
DW                 14.000 usec
DE                  18.00 usec
TE                  298.0 K
D1             2.00000000 sec
D11            0.03000000 sec
TD0                     1
SFO1          150.9178988 MHz
NUC1                  13C
P0                   3.33 usec
P1                  10.00 usec
PLW1          68.97799683 W
SFO2          600.1324005 MHz
NUC2                   1H
CPDPRG[2          waltz65
PCPD2               70.00 usec
PLW2          18.62700081 W
PLW12          0.54741001 W
PLW13          0.27533999 W

F2 - Processing parameters
SI                  32768
SF            150.9028085 MHz
WDW                    EM
SSB                     0
LB                   1.00 Hz
GB                      0
PC                   1.40
```
