# Supplementary material for: Direct (LC-)MS Identification of Regioisomers from C–H Functionalization by Partial Isotopic Labeling
Source: ACS Cent Sci. 2025 Feb 14;11(2):272–8. doi: 10.1021/acscentsci.4c01765 (PMC11868960; doi:10.1021/acscentsci.4c01765)
Supplement: Supplementary file 3 — oc4c01765_si_003.zip [file oc4c01765_si_003.zip › Spectra Raw Files/NMR_SPECTRA.zip!/NMR_SPECTRA/[D]10_1H_benzene/13/pdata/1/email_CS-III-223-2-exchange-d1-20s-16scans_13_1.pdf]

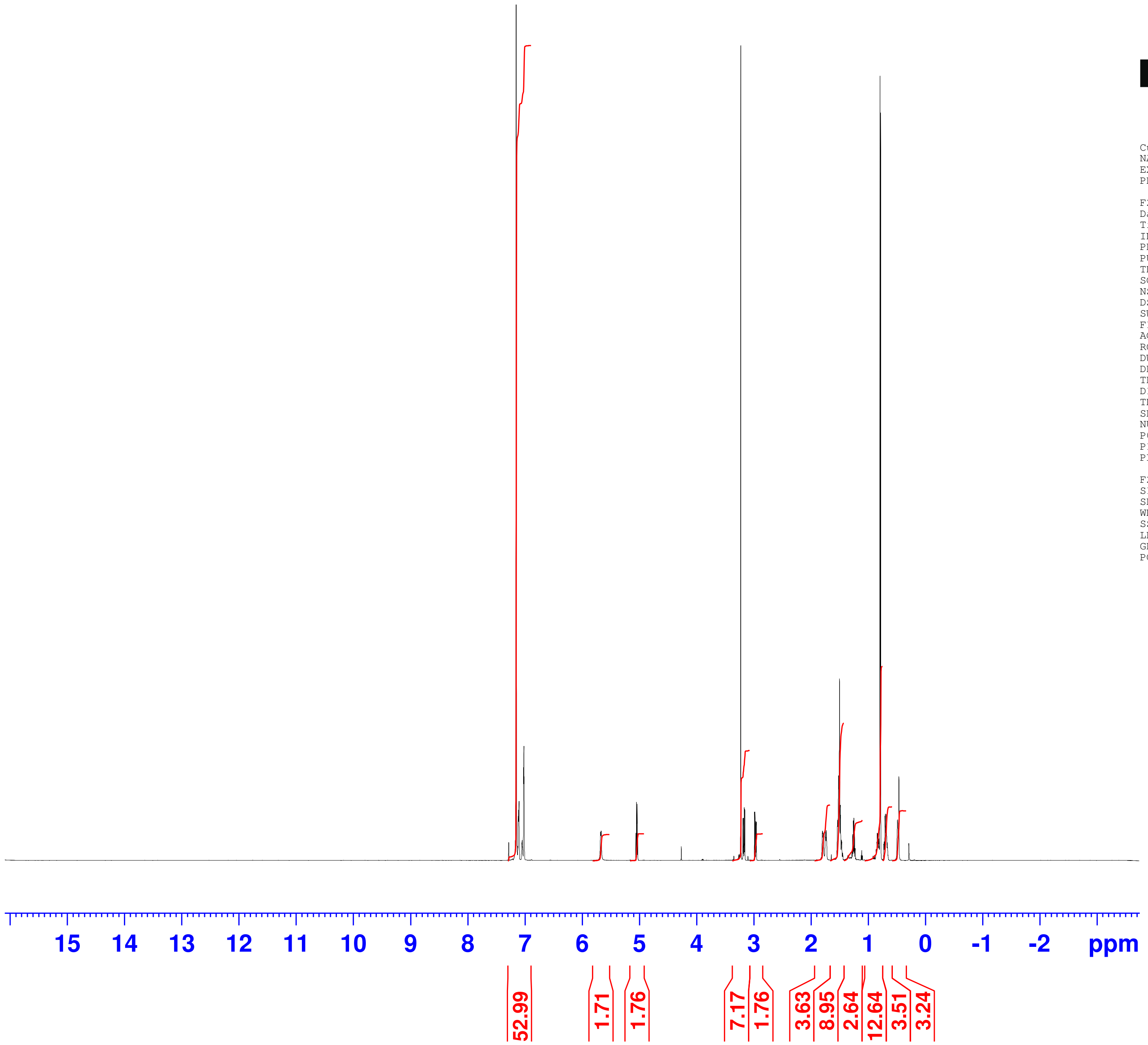


```
Current Data Parameters
NAME      CS-III-223-2-exchange-d1-20s-16scans
EXPNO                    13
PROCNO                    1

F2 - Acquisition Parameters
Date_              20230613
Time                  13.19 h
INSTRUM   Avance 4 NEO 600
PROBHD    Z168773_0007 (
PULPROG                zg30
TD                    65536
SOLVENT                C6D6
NS                       16
DS                        2
SWH               11904.762 Hz
FIDRES             0.363304 Hz
AQ                2.7525120 sec
RG                      101
DW                   42.000 usec
DE                    24.20 usec
TE                    298.0 K
D1              20.00000000 sec
TD0                       1
SFO1            600.1337058 MHz
NUC1                     1H
P0                     4.00 usec
P1                    12.00 usec
PLW1            18.62700081 W

F2 - Processing parameters
SI                    65536
SF              600.1300000 MHz
WDW                      EM
SSB                       0
LB                     0.30 Hz
GB                        0
PC                     1.00
```
