# Supplementary material for: Direct (LC-)MS Identification of Regioisomers from C–H Functionalization by Partial Isotopic Labeling
Source: ACS Cent Sci. 2025 Feb 14;11(2):272–8. doi: 10.1021/acscentsci.4c01765 (PMC11868960; doi:10.1021/acscentsci.4c01765)
Supplement: Supplementary file 3 — oc4c01765_si_003.zip [file oc4c01765_si_003.zip › Spectra Raw Files/NMR_SPECTRA.zip!/NMR_SPECTRA/[D]2_13C/4/pdata/1/email_CS-III-140-3-30min-C_4_1.pdf]

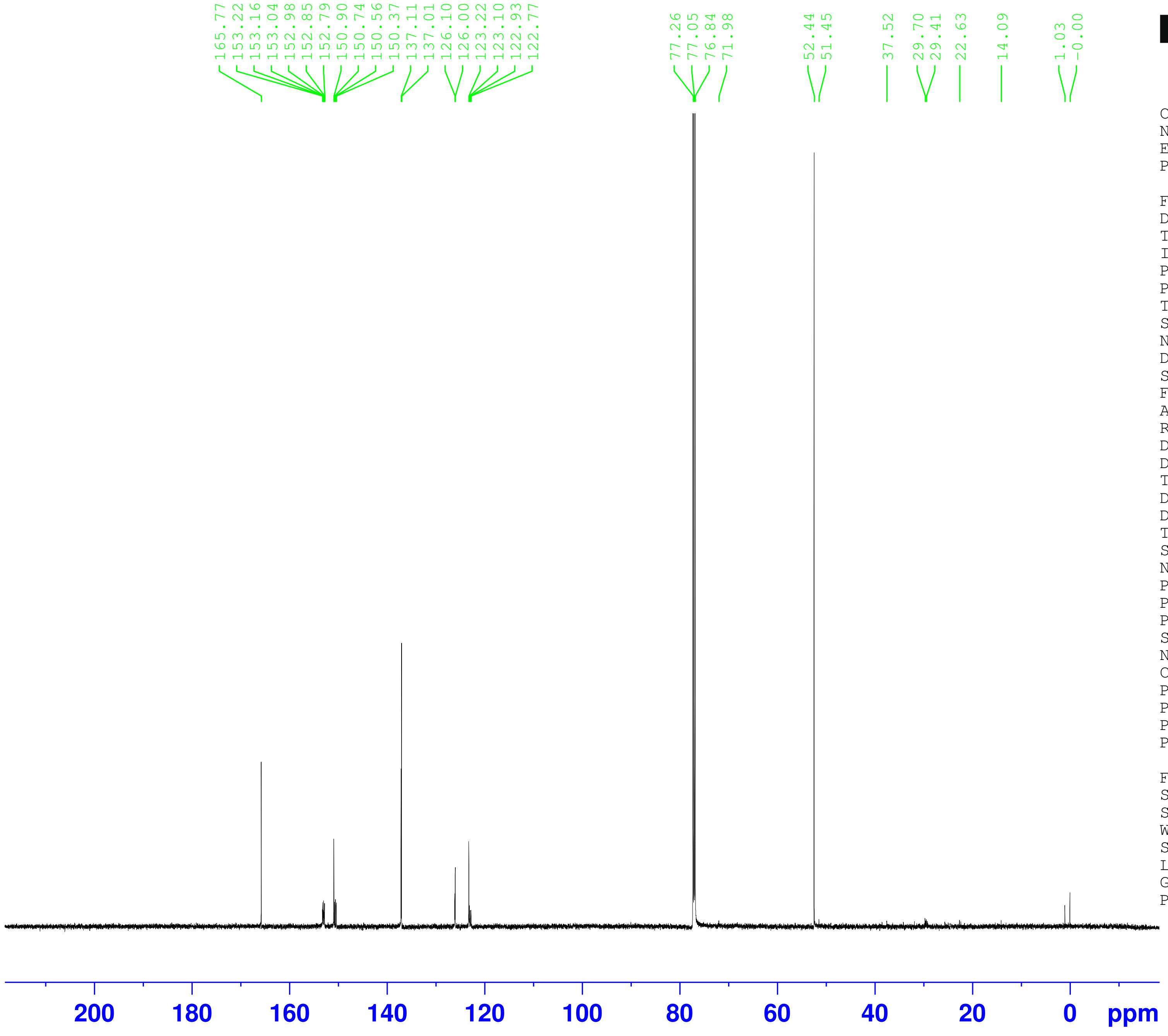

BRUKER

Current Data Parameters
NAME CS-III-140-3-30min-C
EXPNO 4
PROCNO 1

F2 - Acquisition Parameters

Date_	20230905	
Time	6.45	h
INSTRUM	Avance 4 NEO 600	
PROBHD	Z168773_0007 (	
PULPROG	zgpg30	
TD	65536	
SOLVENT	CDCl3	
NS	512	
DS	4	
SWH	35714.286	Hz
FIDRES	1.089913	Hz
AQ	0.9175040	sec
RG	101	
DW	14.000	usec
DE	18.00	usec
TE	298.0	K
D1	2.00000000	sec
D11	0.03000000	sec
TD0	1	
SFO1	150.9178988	MHz
NUC1	13C	
P0	3.50	usec
P1	10.50	usec
PLW1	68.97799683	W
SFO2	600.1324005	MHz
NUC2	1H	
CPDPRG[2	waltz65	
PCPD2	70.00	usec
PLW2	18.62700081	W
PLW12	0.54741001	W
PLW13	0.27533999	W

F2 - Processing parameters

SI	32768	
SF	150.9028069	MHz
WDW	EM	
SSB	0	
LB	1.00	Hz
GB	0	
PC	1.40	
