# Supplementary material for: Direct (LC-)MS Identification of Regioisomers from C–H Functionalization by Partial Isotopic Labeling
Source: ACS Cent Sci. 2025 Feb 14;11(2):272–8. doi: 10.1021/acscentsci.4c01765 (PMC11868960; doi:10.1021/acscentsci.4c01765)
Supplement: Supplementary file 3 — oc4c01765_si_003.zip [file oc4c01765_si_003.zip › Spectra Raw Files/NMR_SPECTRA.zip!/NMR_SPECTRA/[D]2_1H/6/pdata/1/email_CS-III-140-3-d1-30s-16scans-H_6_1.pdf]

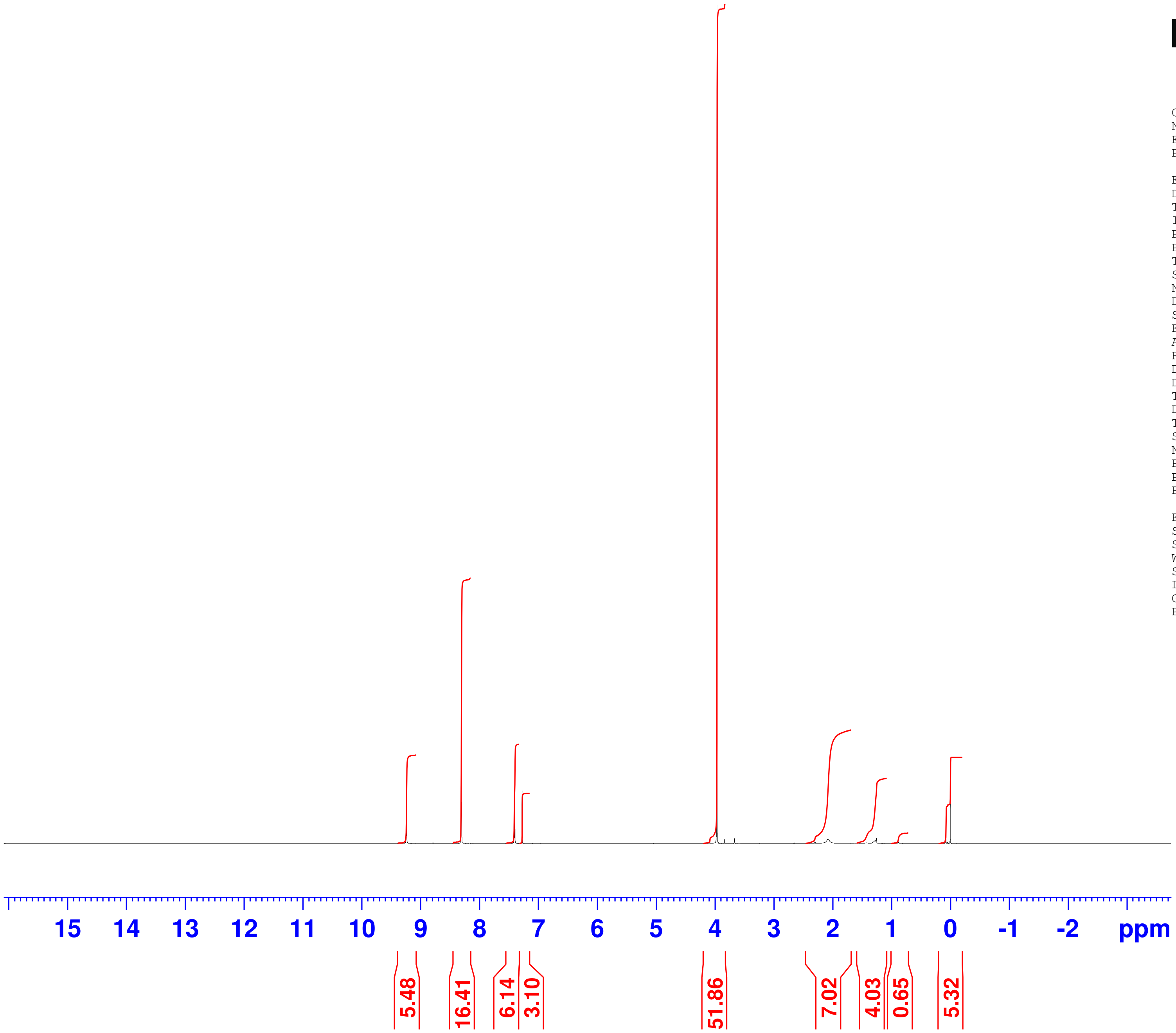


```
Current Data Parameters
NAME     CS-III-140-3-d1-30s-16scans-H
EXPNO                 6
PROCNO                1

F2 - Acquisition Parameters
Date_          20230905
Time               8.03 h
INSTRUM  Avance 4 NEO 600
PROBHD   Z168773_0007 (
PULPROG            zg30
TD                65536
SOLVENT           CDCl3
NS                   16
DS                    2
SWH           11904.762 Hz
FIDRES         0.363304 Hz
AQ            2.7525120 sec
RG                  101
DW               42.000 usec
DE                24.20 usec
TE                298.0 K
D1          30.00000000 sec
TD0                   1
SFO1        600.1337058 MHz
NUC1                 1H
P0                 4.00 usec
P1                12.00 usec
PLW1        18.62700081 W

F2 - Processing parameters
SI                65536
SF          600.1300054 MHz
WDW                  EM
SSB                   0
LB                 0.30 Hz
GB                    0
PC                 1.00
```
