# Supplementary material for: Direct (LC-)MS Identification of Regioisomers from C–H Functionalization by Partial Isotopic Labeling
Source: ACS Cent Sci. 2025 Feb 14;11(2):272–8. doi: 10.1021/acscentsci.4c01765 (PMC11868960; doi:10.1021/acscentsci.4c01765)
Supplement: Supplementary file 3 — oc4c01765_si_003.zip [file oc4c01765_si_003.zip › Spectra Raw Files/NMR_SPECTRA.zip!/NMR_SPECTRA/[D]7_1H/10/pdata/1/email_CS-III-222-1-d1-30s-16scans-H_10_1.pdf]

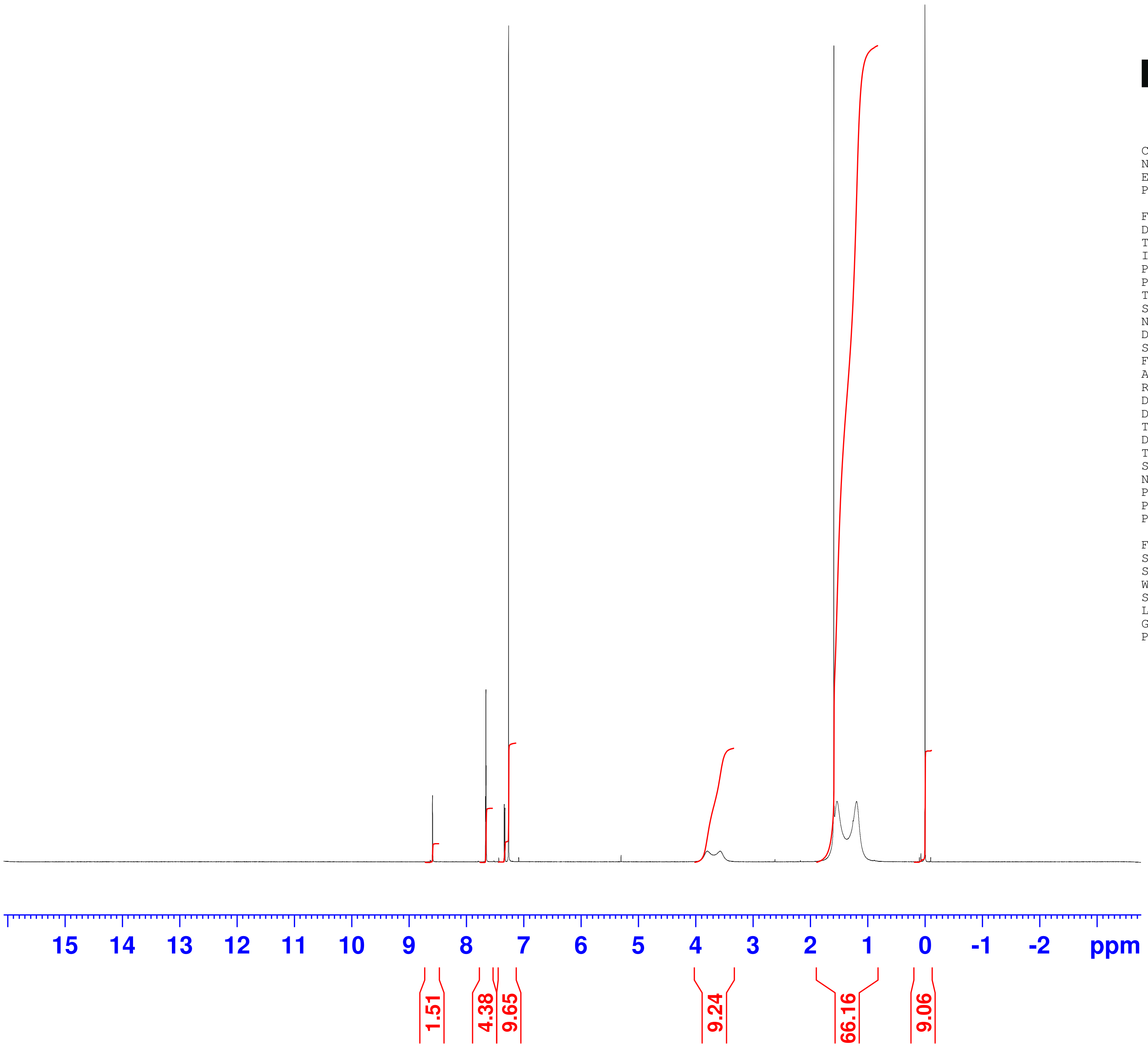

BRUKER

Current Data Parameters
NAME   CS-III-222-1-d1-30s-16scans-H
EXPNO   10
PROCNO   1

F2 - Acquisition Parameters
Date_   20230706
Time   18.48 h
INSTRUM   Avance 4 NEO 600
PROBHD   Z168773_0007 (
PULPROG   zg30
TD   65536
SOLVENT   CDCl3
NS   16
DS   2
SWH   11904.762 Hz
FIDRES   0.363304 Hz
AQ   2.7525120 sec
RG   101
DW   42.000 usec
DE   24.20 usec
TE   298.0 K
D1   30.00000000 sec
TD0   1
SFO1   600.1337058 MHz
NUC1   1H
P0   4.00 usec
P1   12.00 usec
PLW1   18.62700081 W

F2 - Processing parameters
SI   65536
SF   600.1300140 MHz
WDW   EM
SSB   0
LB   0.30 Hz
GB   0
PC   1.00
