# Supplementary material for: Direct (LC-)MS Identification of Regioisomers from C–H Functionalization by Partial Isotopic Labeling
Source: ACS Cent Sci. 2025 Feb 14;11(2):272–8. doi: 10.1021/acscentsci.4c01765 (PMC11868960; doi:10.1021/acscentsci.4c01765)
Supplement: Supplementary file 3 — oc4c01765_si_003.zip [file oc4c01765_si_003.zip › Spectra Raw Files/NMR_SPECTRA.zip!/NMR_SPECTRA/[D]8_13C/10/pdata/1/email_CS-III-218-2-exchange-col-C_10_1.pdf]

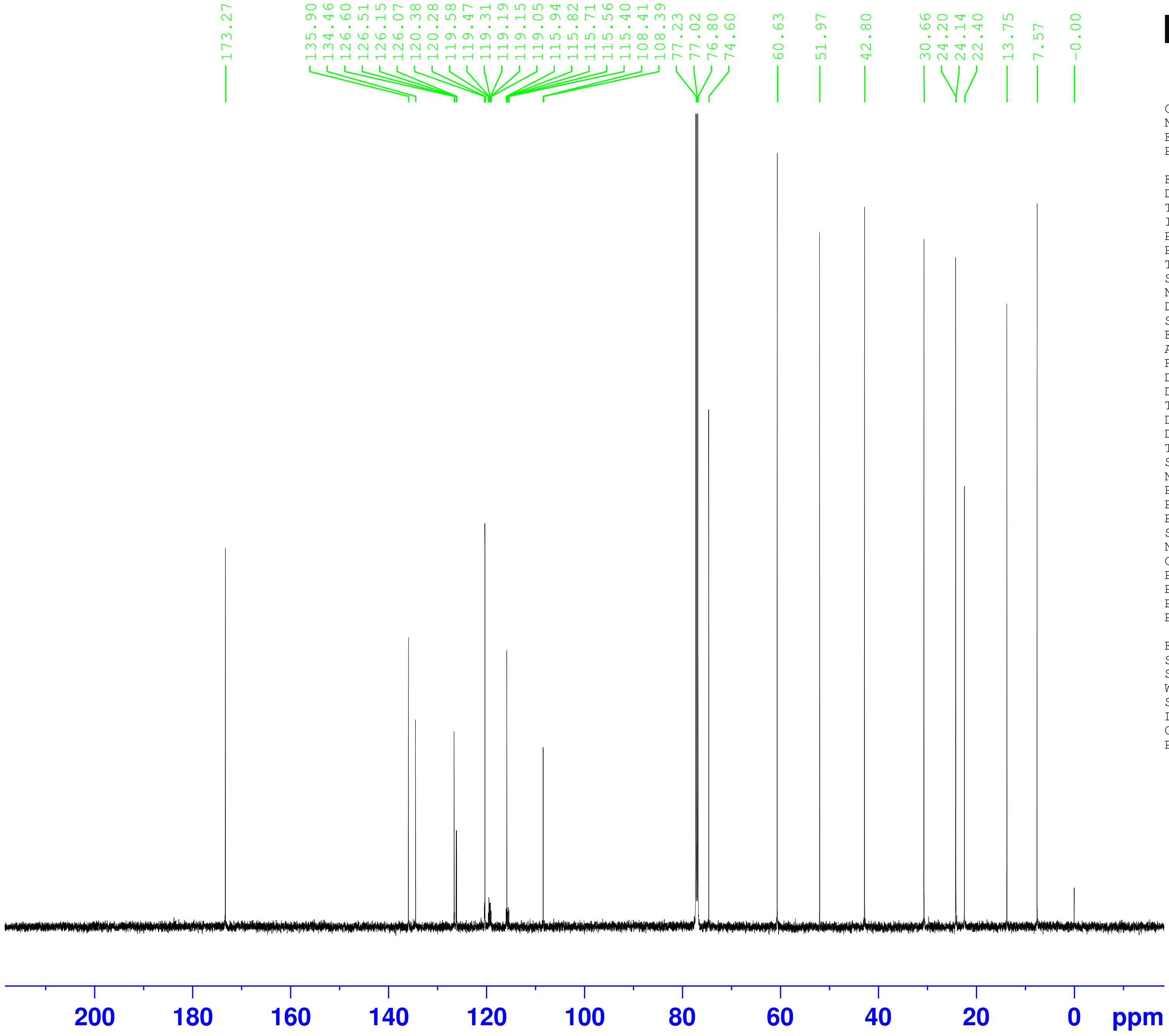


BRUKER

Current Data Parameters

| | |
|---|---|
| NAME | CS-III-218-2-exchange-col-C |
| EXPNO | 10 |
| PROCNO | 1 |

F2 - Acquisition Parameters

| | |
|---|---|
| Date_ | 20230614 |
| Time | 22.52 h |
| INSTRUM | Avance 4 NEO 600 |
| PROBHD | Z168773_0007 ( |
| PULPROG | zgpg30 |
| TD | 65536 |
| SOLVENT | CDCl3 |
| NS | 256 |
| DS | 4 |
| SWH | 35714.285 Hz |
| FIDRES | 1.089913 Hz |
| AQ | 0.9175040 sec |
| RG | 101 |
| DW | 14.000 usec |
| DE | 18.00 usec |
| TE | 298.0 K |
| D1 | 2.00000000 sec |
| D11 | 0.03000000 sec |
| TD0 | 1 |
| SFO1 | 150.9178988 MHz |
| NUC1 | 13C |
| P0 | 3.50 usec |
| P1 | 10.50 usec |
| PLW1 | 68.97799683 W |
| SFO2 | 600.1324005 MHz |
| NUC2 | 1H |
| CPDPRG[2 | waltz65 |
| PCPD2 | 70.00 usec |
| PLW2 | 18.62700081 W |
| PLW12 | 0.54741001 W |
| PLW13 | 0.27533999 W |

F2 - Processing parameters

| | |
|---|---|
| SI | 32768 |
| SF | 150.9028122 MHz |
| WDW | EM |
| SSB | 0 |
| LB | 1.00 Hz |
| GB | 0 |
| PC | 1.40 |
