# Supplementary material for: Direct (LC-)MS Identification of Regioisomers from C–H Functionalization by Partial Isotopic Labeling
Source: ACS Cent Sci. 2025 Feb 14;11(2):272–8. doi: 10.1021/acscentsci.4c01765 (PMC11868960; doi:10.1021/acscentsci.4c01765)
Supplement: Supplementary file 3 — oc4c01765_si_003.zip [file oc4c01765_si_003.zip › Spectra Raw Files/NMR_SPECTRA.zip!/NMR_SPECTRA/[D]9_1H/12/pdata/1/email_CS-IV-35-1-d1-20s-16scans-H_12_1.pdf]

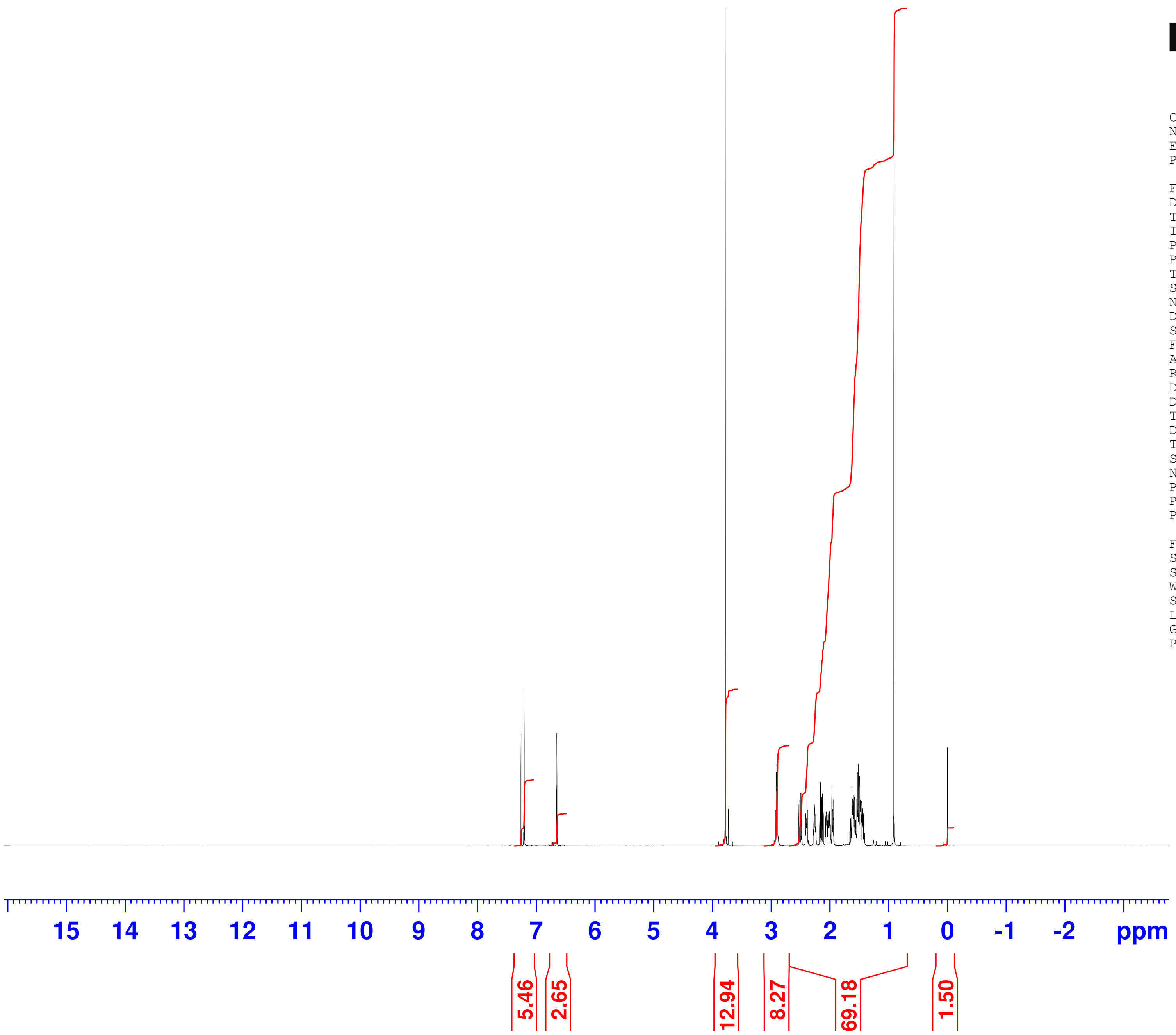

Current Data Parameters
NAME CS-IV-35-1-d1-20s-16scans-H
EXPNO 12
PROCNO 1

F2 - Acquisition Parameters

Date_	20230906
Time	16.48 h
INSTRUM	Avance 4 NEO 600
PROBHD	Z168773_0007 (
PULPROG	zg30
TD	65536
SOLVENT	CDCl3
NS	16
DS	2
SWH	11904.762 Hz
FIDRES	0.363304 Hz
AQ	2.7525120 sec
RG	101
DW	42.000 usec
DE	24.20 usec
TE	298.0 K
D1	20.00000000 sec
TD0	1
SFO1	600.1337058 MHz
NUC1	1H
P0	4.00 usec
P1	12.00 usec
PLW1	18.62700081 W

F2 - Processing parameters

SI	65536
SF	600.1300164 MHz
WDW	EM
SSB	0
LB	0.30 Hz
GB	0
PC	1.00
