# Supplementary material for: Direct (LC-)MS Identification of Regioisomers from C–H Functionalization by Partial Isotopic Labeling
Source: ACS Cent Sci. 2025 Feb 14;11(2):272–8. doi: 10.1021/acscentsci.4c01765 (PMC11868960; doi:10.1021/acscentsci.4c01765)
Supplement: Supplementary file 3 — oc4c01765_si_003.zip [file oc4c01765_si_003.zip › Spectra Raw Files/NMR_SPECTRA.zip!/NMR_SPECTRA/[H]10b_[H]10c_1to1.5_13C/21/pdata/1/email_CS-IV-197-1-s1-s1-C_21_1.pdf]

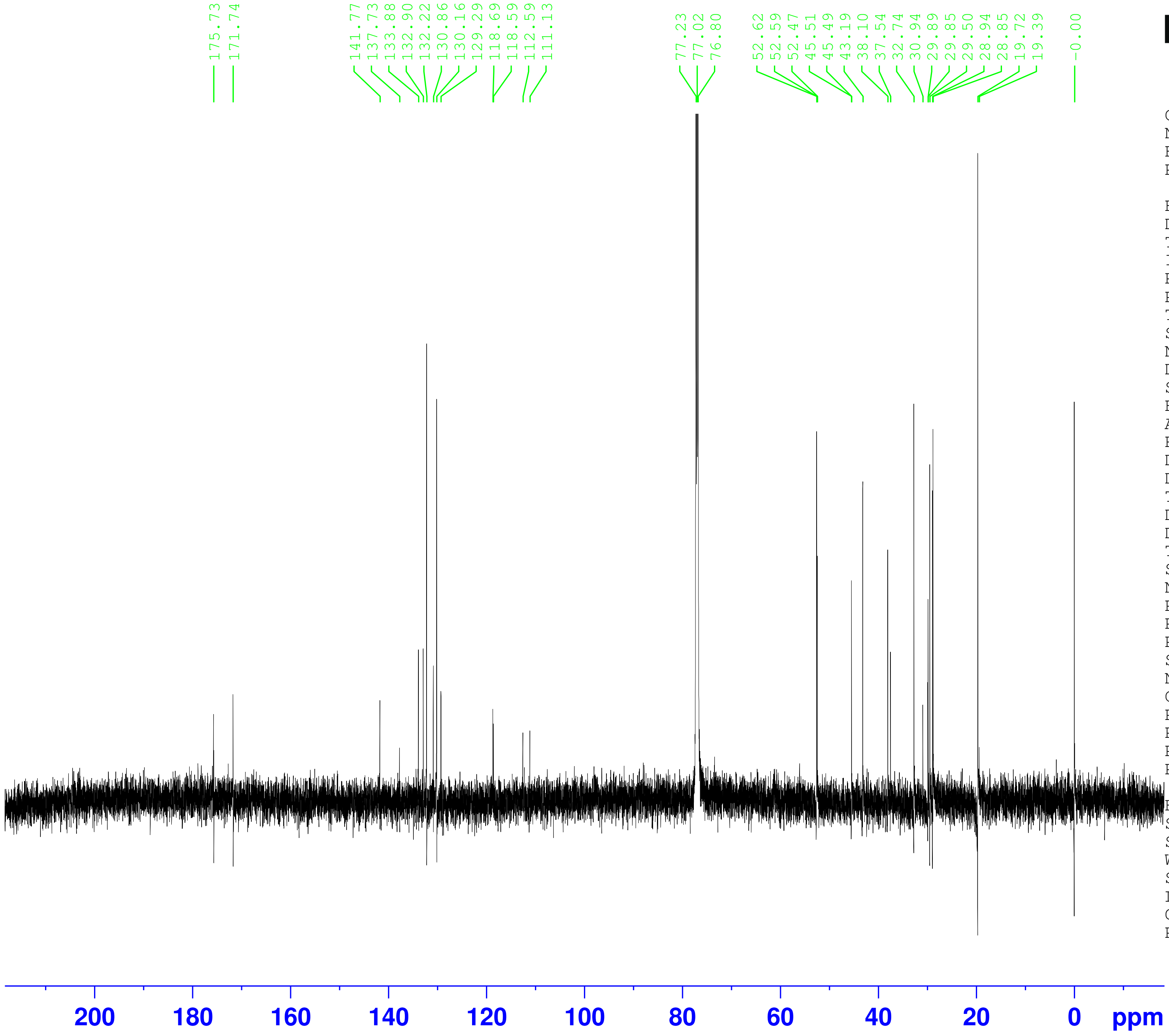

BRUKER

Current Data Parameters
NAME CS-IV-197-1-s1-s1-C
EXPNO 21
PROCNO 1

F2 - Acquisition Parameters
Date_ 20240220
Time 20.52 h
INSTRUM Avance 4 NEO 600
PROBHD Z168773_0007 (
PULPROG zgpg30
TD 65536
SOLVENT CDCl3
NS 1024
DS 4
SWH 35714.286 Hz
FIDRES 1.089913 Hz
AQ 0.9175040 sec
RG 101
DW 14.000 usec
DE 18.00 usec
TE 298.0 K
D1 2.00000000 sec
D11 0.03000000 sec
TD0 1
SFO1 150.9178988 MHz
NUC1 13C
P0 3.50 usec
P1 10.50 usec
PLW1 68.97799683 W
SFO2 600.1324005 MHz
NUC2 1H
CPDPRG[2 waltz65
PCPD2 70.00 usec
PLW2 18.62700081 W
PLW12 0.54741001 W
PLW13 0.27533999 W

F2 - Processing parameters
SI 32768
SF 150.9028087 MHz
WDW EM
SSB 0
LB 1.00 Hz
GB 0
PC 1.40
