# Supplementary material for: Direct (LC-)MS Identification of Regioisomers from C–H Functionalization by Partial Isotopic Labeling
Source: ACS Cent Sci. 2025 Feb 14;11(2):272–8. doi: 10.1021/acscentsci.4c01765 (PMC11868960; doi:10.1021/acscentsci.4c01765)
Supplement: Supplementary file 3 — oc4c01765_si_003.zip [file oc4c01765_si_003.zip › Spectra Raw Files/NMR_SPECTRA.zip!/NMR_SPECTRA/[H]11_1H/CDV-I-272-251-1_a/3/pdata/1/email_CDV-I-272-251-1_a_3_1.pdf]

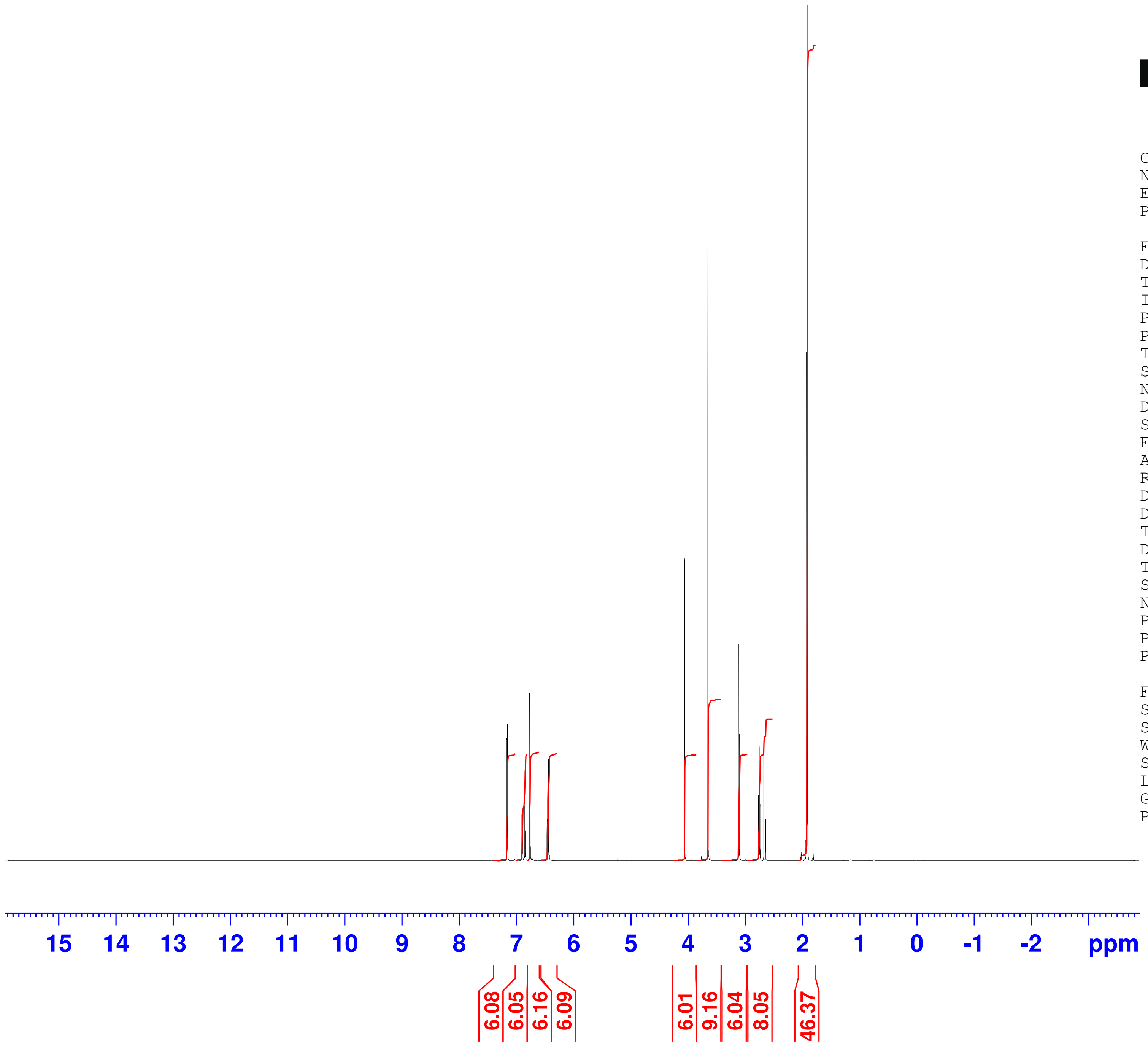


```
Current Data Parameters
NAME       CDV-I-272-251-1_a
EXPNO                      3
PROCNO                     1

F2 - Acquisition Parameters
Date_              20240411
Time                  12.05 h
INSTRUM   Avance 4 NEO 600
PROBHD     Z168773_0007 (
PULPROG               zg30
TD                   65536
SOLVENT            Acetone
NS                      16
DS                       2
SWH              11904.762 Hz
FIDRES            0.363304 Hz
AQ               2.7525120 sec
RG                     101
DW                  42.000 usec
DE                   24.20 usec
TE                   298.0 K
D1             20.00000000 sec
TD0                      1
SFO1           600.1337058 MHz
NUC1                    1H
P0                    4.00 usec
P1                   12.00 usec
PLW1           18.62700081 W

F2 - Processing parameters
SI                   65536
SF             600.1300897 MHz
WDW                     EM
SSB                      0
LB                    0.30 Hz
GB                       0
PC                    1.00
```
