# Supplementary material for: Direct (LC-)MS Identification of Regioisomers from C–H Functionalization by Partial Isotopic Labeling
Source: ACS Cent Sci. 2025 Feb 14;11(2):272–8. doi: 10.1021/acscentsci.4c01765 (PMC11868960; doi:10.1021/acscentsci.4c01765)
Supplement: Supplementary file 3 — oc4c01765_si_003.zip [file oc4c01765_si_003.zip › Spectra Raw Files/NMR_SPECTRA.zip!/NMR_SPECTRA/[H]2_1H/10/pdata/1/email_CS-IV-methylnicotinate-H_10_1.pdf]

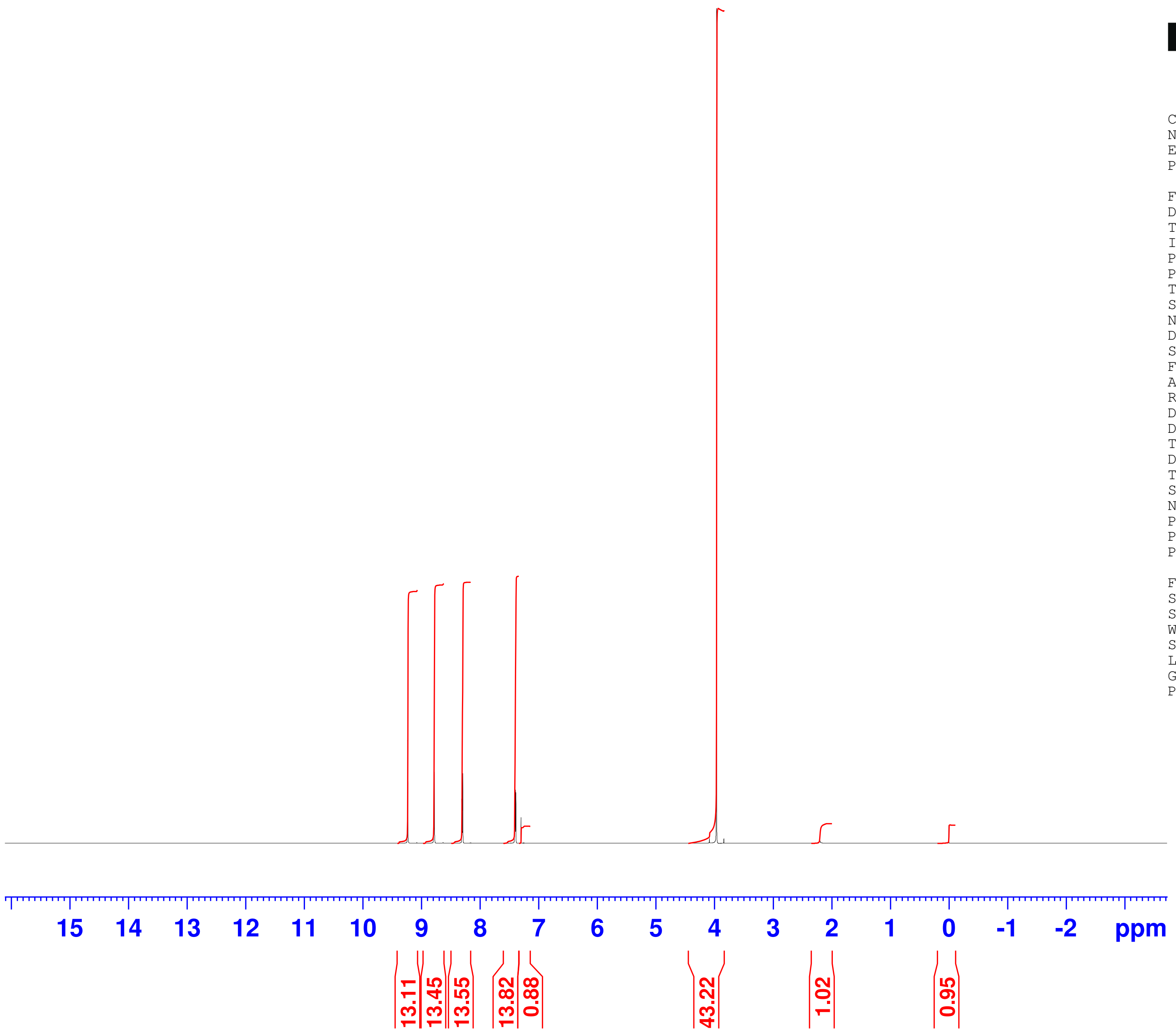

Current Data Parameters
NAME CS-IV-methylnicotinate-H
EXPNO 10
PROCNO 1

F2 - Acquisition Parameters
Date_ 20230915
Time 6.34 h
INSTRUM Avance 4 NEO 600
PROBHD Z168773_0007 (
PULPROG zg30
TD 65536
SOLVENT CDCl3
NS 16
DS 2
SWH 11904.762 Hz
FIDRES 0.363304 Hz
AQ 2.7525120 sec
RG 101
DW 42.000 usec
DE 24.20 usec
TE 298.0 K
D1 1.00000000 sec
TD0 1
SFO1 600.1337058 MHz
NUC1 1H
P0 4.00 usec
P1 12.00 usec
PLW1 18.62700081 W

F2 - Processing parameters
SI 65536
SF 600.1299903 MHz
WDW EM
SSB 0
LB 0.30 Hz
GB 0
PC 1.00
