# Supplementary material for: Direct (LC-)MS Identification of Regioisomers from C–H Functionalization by Partial Isotopic Labeling
Source: ACS Cent Sci. 2025 Feb 14;11(2):272–8. doi: 10.1021/acscentsci.4c01765 (PMC11868960; doi:10.1021/acscentsci.4c01765)
Supplement: Supplementary file 3 — oc4c01765_si_003.zip [file oc4c01765_si_003.zip › Spectra Raw Files/NMR_SPECTRA.zip!/NMR_SPECTRA/[H]3_1H/10/pdata/1/email_CS-IV-3cyanopyridine-H_10_1.pdf]

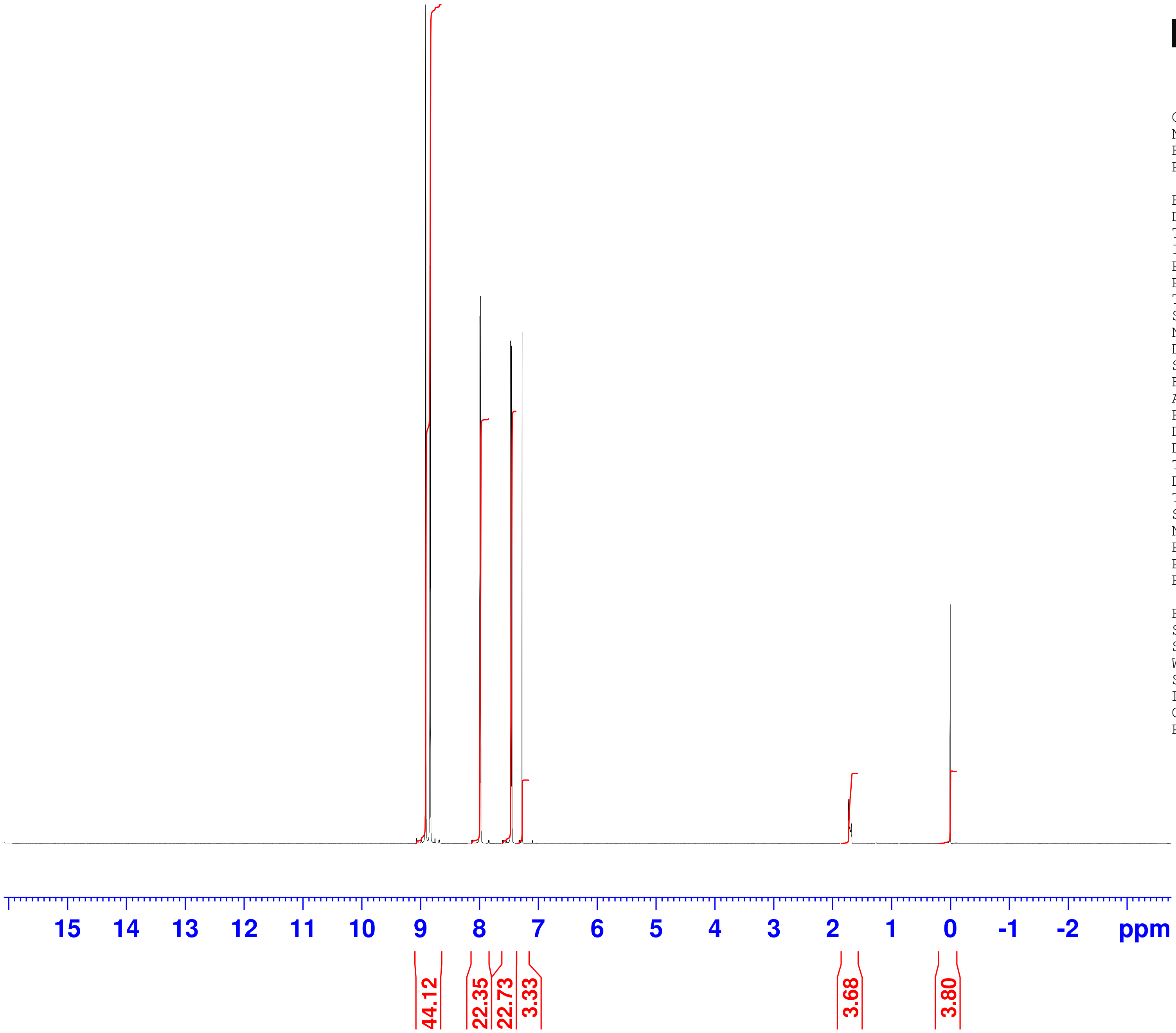

BRUKER

Current Data Parameters
NAME CS-IV-3cyanopyridine-H
EXPNO 10
PROCNO 1

F2 - Acquisition Parameters

Date_	20230914	
Time	6.51	h
INSTRUM	Avance 4 NEO 600	
PROBHD	Z168773_0007 (	
PULPROG	zg30	
TD	65536	
SOLVENT	CDCl3	
NS	16	
DS	2	
SWH	11904.762	Hz
FIDRES	0.363304	Hz
AQ	2.7525120	sec
RG	101	
DW	42.000	usec
DE	24.20	usec
TE	298.0	K
D1	1.00000000	sec
TD0	1	
SFO1	600.1337058	MHz
NUC1	1H	
P0	4.00	usec
P1	12.00	usec
PLW1	18.62700081	W

F2 - Processing parameters

SI	65536	
SF	600.1300058	MHz
WDW	EM	
SSB	0	
LB	0.30	Hz
GB	0	
PC	1.00	
