# Supplementary material for: Direct (LC-)MS Identification of Regioisomers from C–H Functionalization by Partial Isotopic Labeling
Source: ACS Cent Sci. 2025 Feb 14;11(2):272–8. doi: 10.1021/acscentsci.4c01765 (PMC11868960; doi:10.1021/acscentsci.4c01765)
Supplement: Supplementary file 3 — oc4c01765_si_003.zip [file oc4c01765_si_003.zip › Spectra Raw Files/NMR_SPECTRA.zip!/NMR_SPECTRA/[H]8a_[H]8b_mix_1H/12/pdata/1/email_CS-IV-26-1-prodmix-H_12_1.pdf]

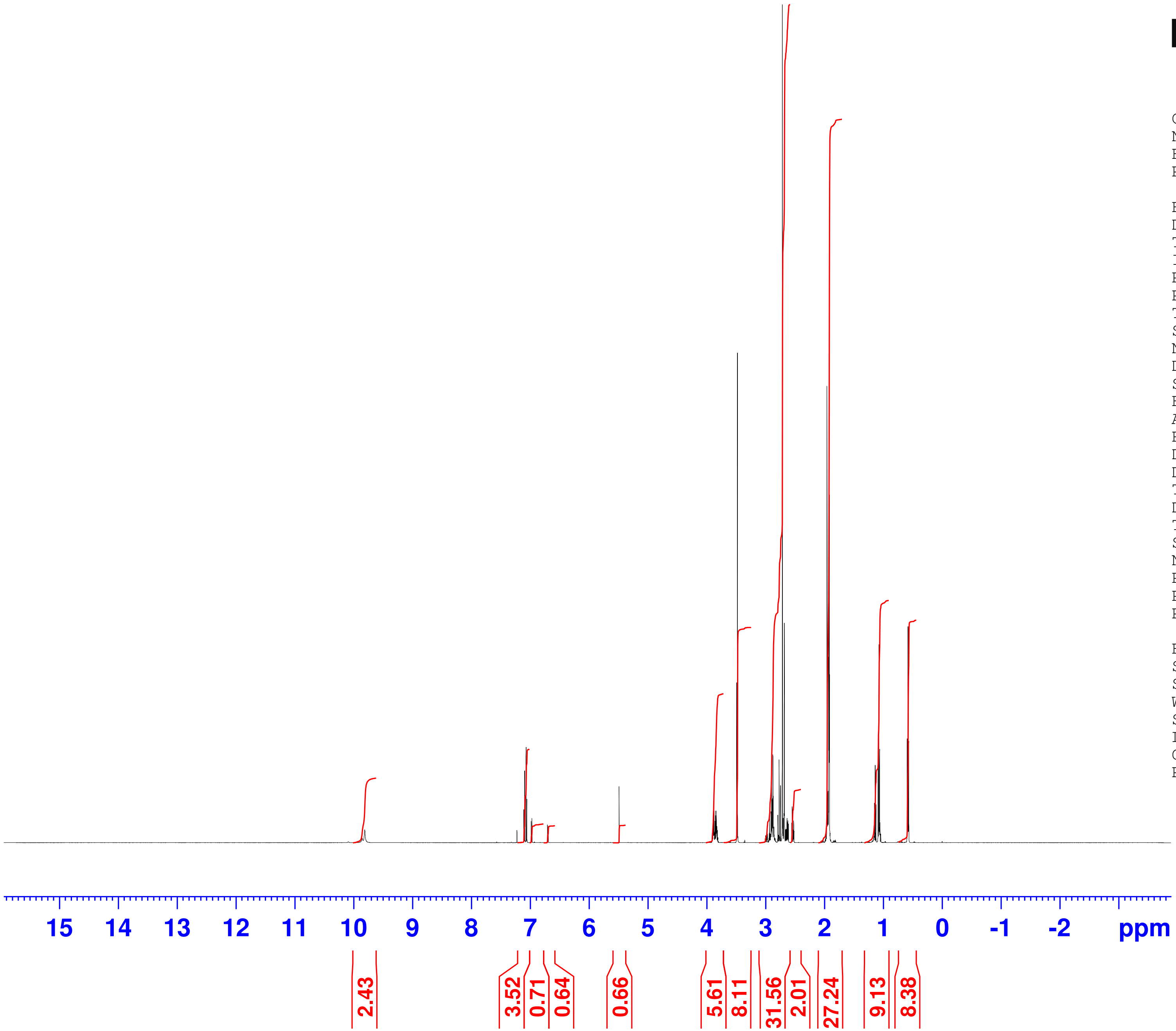

BRUKER

```
Current Data Parameters
NAME      CS-IV-26-1-prodmix-H
EXPNO                  12
PROCNO                  1

F2 - Acquisition Parameters
Date_            20230524
Time                15.59 h
INSTRUM   Avance 4 NEO 600
PROBHD    Z168773_0007 (
PULPROG              zg30
TD                  65536
SOLVENT           Acetone
NS                     16
DS                      2
SWH             11904.762 Hz
FIDRES           0.363304 Hz
AQ              2.7525120 sec
RG                    101
DW                 42.000 usec
DE                  24.20 usec
TE                  298.0 K
D1             1.00000000 sec
TD0                     1
SFO1          600.1337058 MHz
NUC1                   1H
P0                   4.00 usec
P1                  12.00 usec
PLW1          18.62700081 W

F2 - Processing parameters
SI                  65536
SF            600.1300881 MHz
WDW                    EM
SSB                     0
LB                   0.30 Hz
GB                      0
PC                   1.00
```
