# Supplementary material for: Direct (LC-)MS Identification of Regioisomers from C–H Functionalization by Partial Isotopic Labeling
Source: ACS Cent Sci. 2025 Feb 14;11(2):272–8. doi: 10.1021/acscentsci.4c01765 (PMC11868960; doi:10.1021/acscentsci.4c01765)
Supplement: Supplementary file 3 — oc4c01765_si_003.zip [file oc4c01765_si_003.zip › Spectra Raw Files/NMR_SPECTRA.zip!/NMR_SPECTRA/4,5-Dimethylnicotinonitrile-2,6-d2_13C/30/pdata/1/email_PJA-I-149- Pure 13C_30_1.pdf]

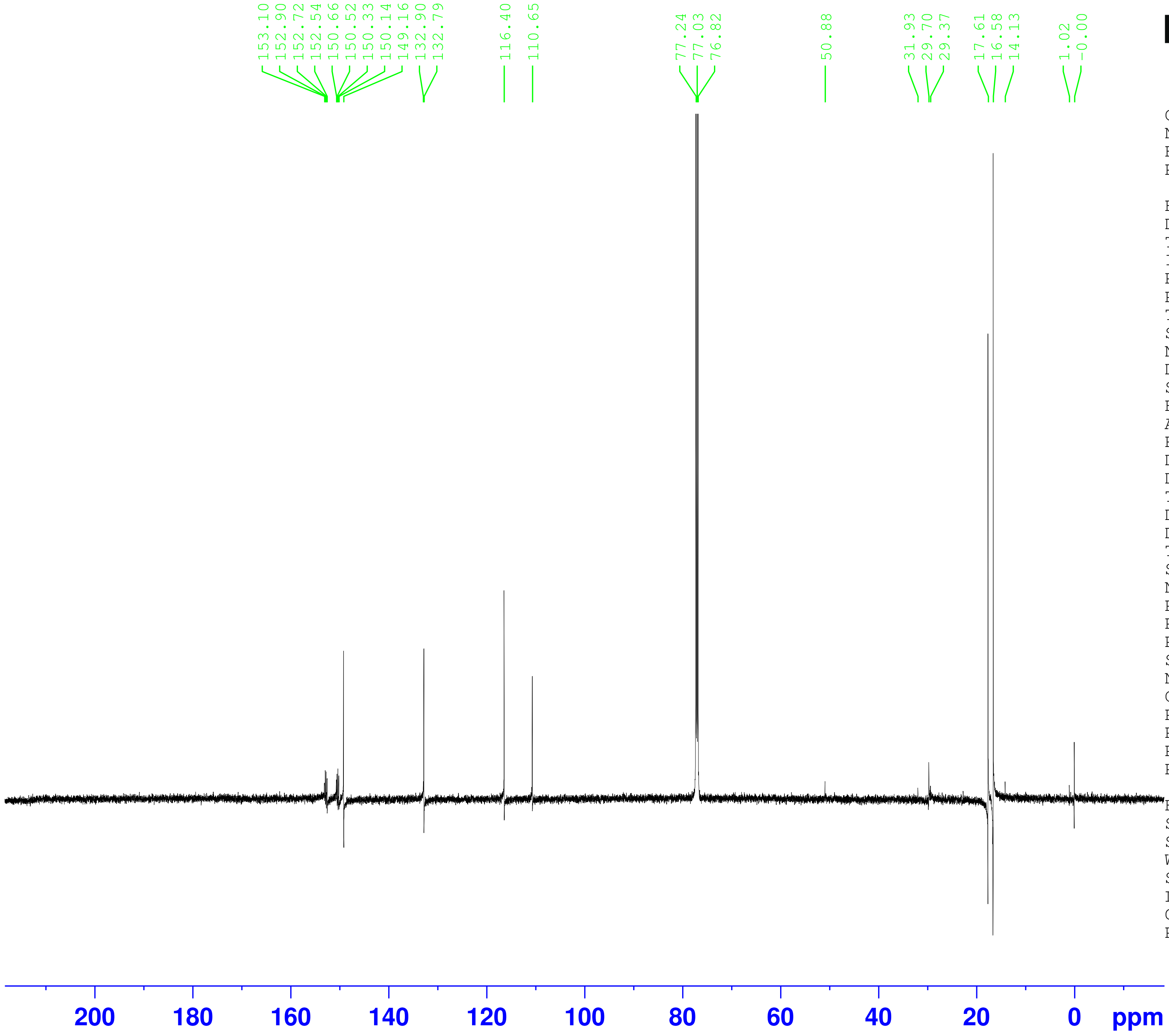

Current Data Parameters

NAME	PJA-I-149- Pure 13C
EXPNO	30
PROCNO	1

F2 - Acquisition Parameters

Date_	20230212
Time	1.00 h
INSTRUM	Avance 4 NEO 600
PROBHD	Z168773_0007 (
PULPROG	zgpg30
TD	65536
SOLVENT	CDCl3
NS	1024
DS	4
SWH	35714.285 Hz
FIDRES	1.089913 Hz
AQ	0.9175040 sec
RG	101
DW	14.000 usec
DE	18.00 usec
TE	298.0 K
D1	2.00000000 sec
D11	0.03000000 sec
TD0	1
SFO1	150.9178988 MHz
NUC1	13C
P0	3.33 usec
P1	10.00 usec
PLW1	68.97799683 W
SFO2	600.1324005 MHz
NUC2	1H
CPDPRG[2	waltz65
PCPD2	70.00 usec
PLW2	18.62700081 W
PLW12	0.54741001 W
PLW13	0.27533999 W

F2 - Processing parameters

SI	32768
SF	150.9028082 MHz
WDW	EM
SSB	0
LB	1.00 Hz
GB	0
PC	1.40
