# Supplementary material for: Direct (LC-)MS Identification of Regioisomers from C–H Functionalization by Partial Isotopic Labeling
Source: ACS Cent Sci. 2025 Feb 14;11(2):272–8. doi: 10.1021/acscentsci.4c01765 (PMC11868960; doi:10.1021/acscentsci.4c01765)
Supplement: Supplementary file 3 — oc4c01765_si_003.zip [file oc4c01765_si_003.zip › Spectra Raw Files/NMR_SPECTRA.zip!/NMR_SPECTRA/Proof_of_Concept_products/[D]2b_13C/7/pdata/1/email_DAP-I-151-3-13C_7_1.pdf]

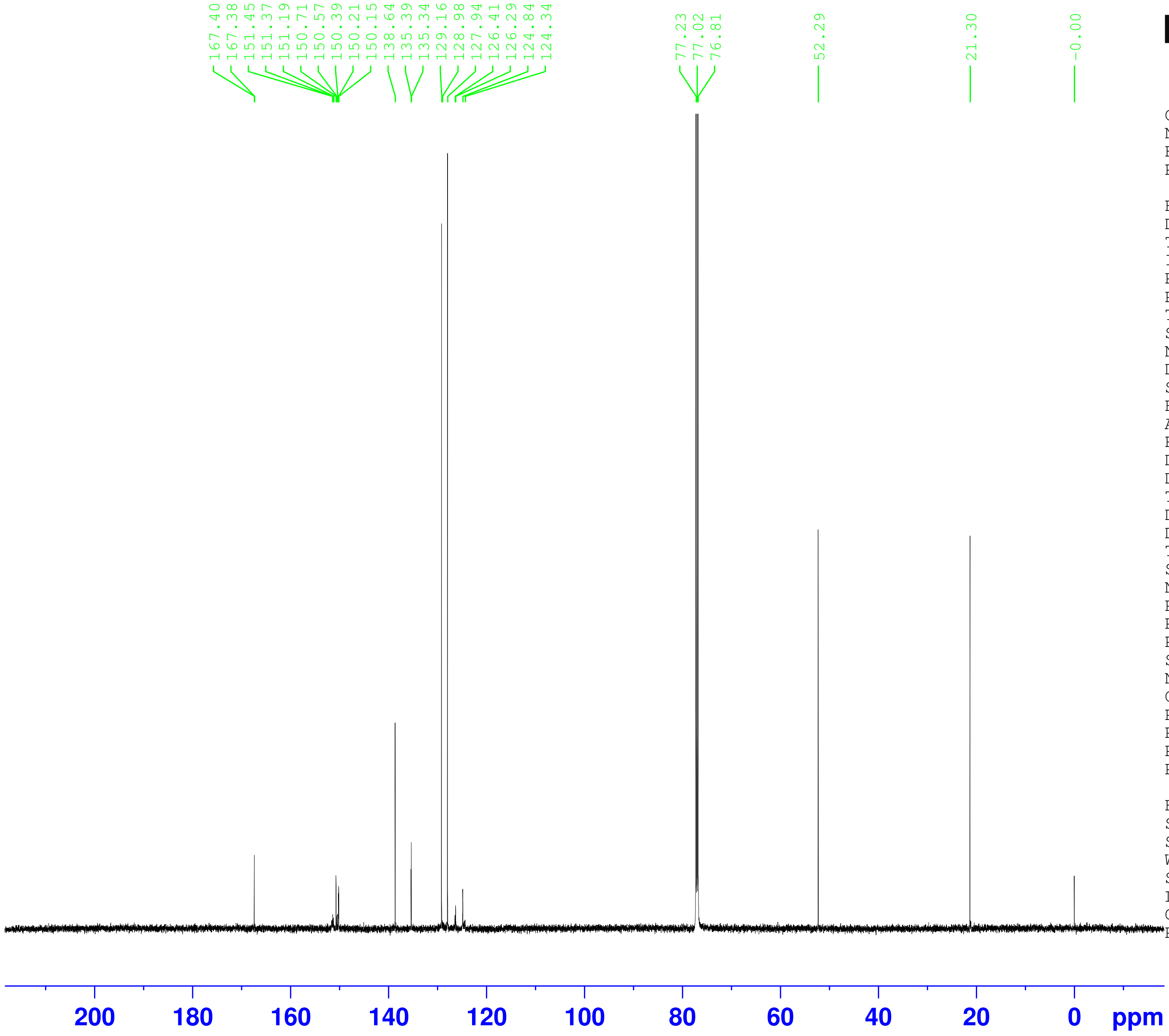


```
Current Data Parameters
NAME      DAP-I-151-3-13C
EXPNO                   7
PROCNO                  1

F2 - Acquisition Parameters
Date_            20230615
Time               8.05 h
INSTRUM   Avance 4 NEO 600
PROBHD    Z168773_0007 (
PULPROG           zgpg30
TD                 65536
SOLVENT            CDCl3
NS                   512
DS                     4
SWH            35714.285 Hz
FIDRES          1.089913 Hz
AQ             0.9175040 sec
RG                   101
DW                14.000 usec
DE                 18.00 usec
TE                 298.0 K
D1            2.00000000 sec
D11           0.03000000 sec
TD0                    1
SFO1         150.9178988 MHz
NUC1                 13C
P0                  3.50 usec
P1                 10.50 usec
PLW1         68.97799683 W
SFO2         600.1324005 MHz
NUC2                  1H
CPDPRG[2         waltz65
PCPD2              70.00 usec
PLW2         18.62700081 W
PLW12         0.54741001 W
PLW13         0.27533999 W

F2 - Processing parameters
SI                 32768
SF           150.9028098 MHz
WDW                   EM
SSB                    0
LB                  1.00 Hz
GB                     0
PC                  1.40
```
