# Supplementary material for: Direct (LC-)MS Identification of Regioisomers from C–H Functionalization by Partial Isotopic Labeling
Source: ACS Cent Sci. 2025 Feb 14;11(2):272–8. doi: 10.1021/acscentsci.4c01765 (PMC11868960; doi:10.1021/acscentsci.4c01765)
Supplement: Supplementary file 3 — oc4c01765_si_003.zip [file oc4c01765_si_003.zip › Spectra Raw Files/NMR_SPECTRA.zip!/NMR_SPECTRA/Protio_Well_A1_Validation_NMRs/[H]2a_1H/10/pdata/1/email_DAP-I-98-4B2-R2_10_1.pdf]

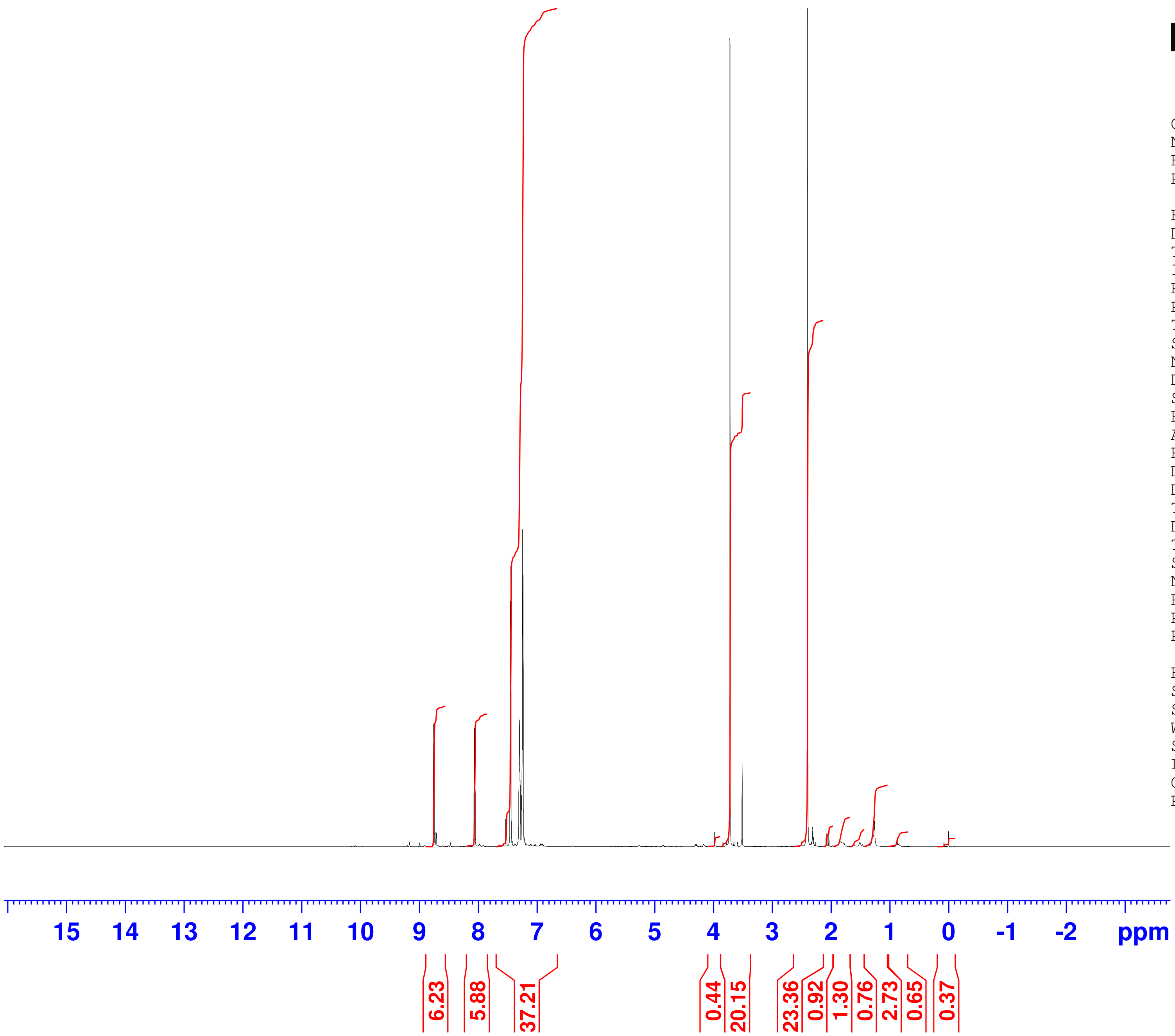

BRUKER

```
Current Data Parameters
NAME       DAP-I-98-4B2-R2
EXPNO                   10
PROCNO                   1

F2 - Acquisition Parameters
Date_             20230228
Time                 17.46 h
INSTRUM   Avance 4 NEO 600
PROBHD    Z168773_0007 (
PULPROG               zg30
TD                   65536
SOLVENT              CDCl3
NS                      16
DS                       2
SWH              11904.762 Hz
FIDRES            0.363304 Hz
AQ               2.7525120 sec
RG                    71.8
DW                  42.000 usec
DE                   24.20 usec
TE                   298.0 K
D1              1.00000000 sec
TD0                      1
SFO1        600.1337058 MHz
NUC1                    1H
P0                    4.00 usec
P1                   12.00 usec
PLW1           18.62700081 W

F2 - Processing parameters
SI                   65536
SF          600.1300153 MHz
WDW                     EM
SSB                      0
LB                    0.30 Hz
GB                       0
PC                    1.00
```
